# Analytical validation and sequencing coverage studies suggest that performance of a liquid biopsy assay is tumor agnostic (DNA-is-DNA)

**DOI:** 10.1371/journal.pone.0329392

**Published:** 2025-08-01

**Authors:** Wei Meng, Russell Petry, Norberto Pantoja Galicia, Allison van den Hout, Jessie Yu, Siliang Gong, Dhara Shah, Daokun Sun, Cui Guo, Shannon Bailey, Daniela Munafo, Ryan Woodhouse, Elizabeth Mansfield, Varun Pattani, Steven Perrault, Jun Zhou, Christine Vietz, Meijuan Li, Richard S.P. Huang

**Affiliations:** Foundation Medicine, Inc., Boston, Massachusetts, United States of America; University of Arkansas for Medical Sciences, UNITED STATES OF AMERICA

## Abstract

Per regulatory and standard requirements (e.g., Clinical & Laboratory Standards Institute (CLSI) guidelines, United States Food and Drug Administration (FDA) correspondence), analytical validation (AV) for each companion diagnostic (CDx) biomarker should be repeated using a clinical sample set for each cancer type listed as an indication in labelling for a CDx. Using data from AV studies and Foundation Medicine (FMI)’s clinical database, we evaluated the hypothesis that analytical performance of the FoundationOne^®^Liquid CDx (F1LCDx) assay is not impacted by cancer type and that large sets of clinical, tumor-specific samples might not be necessary for analytical validation of specific CDx biomarkers. We retrospectively evaluated all liquid biopsy samples from F1LCDx assay AV studies that were executed between April 2019 and November 2021 and clinical samples processed by F1LCDx between September 2020 and October 2021. For the samples from AV studies, we evaluated the precision and concordance performance by F1LCDx between tumor types; and for the clinical samples, we performed analyses comparing the distribution of coverage between tumor types. A total of 31,247 F1LCDx clinical samples and 579 samples from F1LCDx AV studies with a total of 335 disease ontologies (DOs) were included in this study. For precision: the median absolute pairwise difference of mean reproducibility between any pairs of two tumor types is 0.94% [0.01%−2.63%] and the median absolute pairwise difference of mean repeatability between any pairs of two tumor types is 0.91% [0.03%−2.98%]. For concordance: the median absolute $\partial_{PPA}$ between any pairs of two tumor types is 1.39% [0.1%−4.1%] and the median absolute $\partial_{NPA}$ between any pairs of two tumor types is 0.05% [0%−1%]. For coverage, a similar distribution was observed between tumor types using F1LCDx clinical samples. Herein, the results based on the extensive cohort of 31,826 liquid biopsy samples sequenced by the F1LCDx assay demonstrated that both analytical assessment of precision and concordance and coverage are comparable among tumor types (i.e., deoxyribonucleic acid (DNA) is DNA). The tumor type that circulating tumor DNA (ctDNA) was derived from is therefore not a vital consideration for AV studies for F1LCDx assay.

## Introduction

Companion diagnostic (CDx) approvals with oncologic drugs has accelerated in the past decade showcasing the increasing importance of CDx for oncology treatment [1]. CDx tests provide clinicians with critical information by identifying genomic alterations within a patient’s cancer and can potentially match these with highly specific targeted therapies. Oncology CDx tests have evolved significantly since the first approval for HER2 immunohistochemistry testing in breast carcinoma tissue specimens [2]. Since then, a multitude of tissue based CDx tests have been approved by the United States Food and Drug Administration (FDA) in single tumor types such as *BRAF* V600E in melanoma and *EGFR* exon 19 deletions in non-small cell lung cancer (NSCLC) [1]. The advent of next generation sequencing (NGS) technology led to the development and approval of CDx assays that simultaneously evaluate tens or hundreds of biomarkers from a single tissue biopsy. This includes low prevalence biomarkers such as *NTRK1*, *NTRK2*, and *NTRK3* (*NTRK1/2/3)* fusions as well as higher prevalence complex biomarkers such as tumor mutational burden-High (TMB-H).

FDA-approved NGS-based assays such as FoundationOne^®^CDx (F1CDx®) were developed as pan-solid tumor profiling assays. This is remarkable due to the heterogeneity of tumor structure and microenvironments, as well as the complexity of NGS workflows. Although specimen procurement may be specifically tailored for different sample matrix types, the pre-analytical workflow (i.e., library construction, hybridization capture, and sequencing) can remain consistent in a robust comprehensive genome profiling (CGP) assay. More recent advances have produced CDx tests capable of measuring and analyzing deoxyribonucleic acid (DNA) shed from tumors into the blood (circulating tumor DNA (ctDNA)), a phenomenon first characterized and demonstrated in 1977 [3]. These “liquid biopsy” assays are minimally invasive and offer other advantages, such as allowing frequent sampling, providing a more complete picture of the genomics driving a patient’s heterogenic cancer, and estimating disease burden, whereas a tissue biopsy is limited to a specific tumor sample [4]. Liquid biopsy assays have been successfully demonstrated blood as a viable source for clinical testing in multiple tumor types [5–7].

Similar to tumor tissue CDx tests, the evolution of liquid biopsy-based oncology CDx development has been moving towards multi- or pan-cancer assays (e.g., F1LCDx). Liquid biopsies are being used increasingly in many clinical practices such as early detection, identify intended use (IU) cancer population to help healthcare providers make treatment decisions, predict prognosis in monitoring, etc.. Yet considerable differences exist in the molecular biology behind solid and liquid-based assays. The cell free DNA (cfDNA) extracted from blood typically includes only a small fraction of ctDNA, with the remainder being shed as a normal process by the turnover of healthy tissues, primarily endothelial and immune cells. Considering the circulatory system as a “sink” that captures nucleic acids and other debris shed during cell death from the entire body, the population of cfDNA and ctDNA can be quite heterogeneous. The processes leading to cell shedding include apoptosis (primary mechanism of healthy cells), necrosis (primary mechanism of tumor cells), and active secretion from cells throughout the body. This variability and other factors such as epigenetic state and nuclease activity lead to DNA fragment sizes that are variable and specific to the source cells. Once in the blood, there are a variety of clearance mechanisms that are dependent on fragment size, and which lead to different rates of clearance [8]. On a more granular level, differences in single-strand overhangs, and damage such as single-strand breaks may exist. Despite these challenges, FDA approval of two multi-cancer liquid biopsy NGS CDx tests (i.e., F1LCDx and Guardant360^®^ CDx) suggests that a well-developed, pre-analytical workflow can be robust enough to overcome ctDNA heterogeneity.

NGS-based oncology CDx tests require highly stringent analytical validation (AV) and quality control (QC) during development and during clinical testing to ensure reliable results. Per Clinical & Laboratory Standards Institute (CLSI) guidelines and FDA expectations, AV for each individual CDx biomarker necessitates essential studies including precision and orthogonal concordance using a clinical sample set for each cancer type listed as an indication on the CDx technical label [9]. Certain targetable genomic alterations such as *NTRK1/2/3* fusions have an extremely low overall prevalence (~0.3% of all solid tumors) [10]. These low prevalence biomarkers pose a tremendous challenge if independent AV is required in each tumor type in which the biomarker may be relevant for therapy, particularly in rare cancer types. To overcome this, we undertook a study to measure the analytical performance of F1LCDx across 335 disease ontologies (DOs), with the hypothesis that the robustness of the entire analytical workflow can overcome ctDNA heterogeneity challenges. Using data from AV studies conducted at Foundation Medicine, Inc. (FMI), and FMI’s clinical database, we tested the hypothesis that F1LCDx assay performance is not impacted by cancer type and large sets of clinical, tumor-specific samples might not be necessary for future AV studies.

## Materials and methods

### Sample Selection

Samples used to evaluate assay performance across tumor types consisted of samples processed within F1LCDx assay AV studies (i.e., precision and concordance) (denoted as AV samples in the following context) as well as clinically tested samples from FMI’s clinical database (denoted as clinical samples in the following context). Institutional Review Board (IRB) approval was obtained from the WIRB-Copernicus Group (WCG ®) IRB prior to use of samples in the described validation studies. All samples were sequenced with the F1LCDx assay as previously described [11]. F1LCDx assay AV studies were executed between April 2019 (i.e., the AV studies conducted for the F1LCDx pre-market approval (PMA) submission) and October 2021 (i.e., the time of analysis execution for this manuscript). F1LCDx clinical samples included data from samples processed by F1LCDx between September 2020 (i.e., the launch of F1LCDx assay after FDA’s approval) and October 2021 (i.e., the time of analysis execution for this manuscript). The data were accessed on Oct 13, 2021 for the evaluation purpose of this manuscript.

### Precision analysis of AV samples

The precision analysis evaluated pairwise difference in mean reproducibility and repeatability calculated by percent agreement between two different tumor types for short variants (SVs). The 95% 2-sided confidence interval (CI) for pairwise difference based on bootstrap methodology was computed for both mean reproducibility and mean repeatability.

In addition, the pairwise difference along with the 95% 2-sided CIs based on bootstrap methodology in mean coefficients of variance (%CV) of repeatability and reproducibility using the underlying variant allele frequency (VAF) values of the SVs calculated from variance component analysis were calculated between all pairs of tumor types. Tumor types with a pre-set minimum number of unique variants (≥30) were included in the analysis.

### Concordance analysis of AV samples

The concordance assessment was performed by performing subgroup analysis of each tumor type. The purpose of the analysis was to examine the magnitude of the effect of tumor type on orthogonal concordance. This magnitude can be used to determine clinical significance. For a given tumor type, the positive percent agreement (PPA) and the negative percent agreement (NPA) were calculated using other assays as the reference assay. And the pairwise difference of PPA and NPA between tumor types were calculated along with the 95% 2-sided Wilson score CIs for pairwise difference.

### Coverage analysis of clinical samples

Target-level coverage, variant-level coverage, and sample-level coverage were evaluated for F1LCDx between tumor types. Target-level coverage is the number of reads mapped to a given baited region. Regions of the baitset are separated into two categories: enhanced sensitivity or standard sensitivity, in which the enhanced sensitivity regions are sequenced more deeply. Variant-level coverage is defined as the number of reads at the position of a given variant. This analysis included analyses of reads containing the variant (i.e., mutant allele depth) as well as all reads at the position (i.e., total depth). Coverage values across these two categories are averaged, producing sample-level coverage. Specifically, test QC for sample-level coverage assesses the 15^th^ percentile for enhanced sensitivity and standard sensitivity respectively.

For each coverage metric, the descriptive statistics (minimum, Q1, median, mean, Q3, maximum, and SD) of coverage metrics for each tumor type were provided for clinical samples, separately, by post-sequencing QC status (i.e., QC = Pass/Qualified). The distribution was reported only for all tumor types that have meet a pre-set minimum number of data points (for sample-level and target-level coverage: ≥ 500 for QC = Pass and ≥100 for QC = Qualified; for variant-level coverage: ≥ 500 for QC = Pass or QC = Qualified).

Both clinical significance and statistical significance have the importance of their own. The statistically significant results may not be of clinical importance, and vice versa [12]. Statistical significance is heavily dependent on the study’s sample size; with large sample sizes, even small differences can appear statistically significant; therefore, it is important to interpret carefully whether this “significance” is clinically meaningful [13]. Therefore, this paper focuses on the clinical significance, no statistical testing was performed to compare the pairwise differences or the distribution of coverage metrics between tumor types.

## Results

### Sample cohort

A total of 31,247 clinical samples and a total of 579 AV samples with a total of 335 DOs that met the established criteria were included in this study for the above-described analyses.

### Precision results

A total of 3,302 valid data points were evaluated from a total of 16 DOs. Upon grouping these DOs per FMI’s genomics dataset grouping names, there were six (6) tumor types (Breast, Colorectal (CRC), Non-small cell lung carcinoma (NSCLC), Melanoma, Ovary, and Prostate) with at least 30 data points included in the assessment.

#### Pairwise difference of mean reproducibility and repeatability.

Of 3,302 data points, a total of 2,875 data points of reproducibility and repeatability for base substitutions and 427 data points of reproducibility and repeatability for indels from precision studies were included in this assessment.

The mean reproducibility and repeatability for each major tumor type group as well as the pairwise differences with 95% 2-sided CIs based on bootstrap methodology are presented in Tables 1 and 2. The results are summarized for substitutions and indels respectively. In summary, the median absolute pairwise difference of mean reproducibility is 0.94% and it ranges from 0.01% to 2.63%. And the median absolute pairwise difference of mean repeatability is 0.91% and it ranges from 0.03% to 2.98%. The consistent small difference values of mean reproducibility and mean repeatability suggest that the tumor type does not influence the results of precision studies.

**Table 1:** Pairwise difference of mean reproducibility (%) between tumor types for each SV type (substitution and indel).

Variant Type	1^st^ Tumor Type	# of Precision Data Points of 1^st^ Tumor Type	# of Unique Variants of 1^st^ Tumor Type	Mean Reproducibility (%) of 1^st^ Tumor Type	2^nd^ Tumor Type	# of Precision Data Points of 2^nd^ Tumor Type	# of Unique Variants of 2^nd^ Tumor Type	Mean Reproducibility (%) of 2^nd^ Tumor Type	Mean Difference in Reproducibility (%) between 1^st^ and 2^nd^ Tumor Type	Two-sided 95% CI for Mean Difference (%)
**Substitution**	**Breast**	188	185	98.55	**CRC**	251	249	98.08	0.47	[-0.35, 1.77]
	**Breast**	188	185	98.55	**NSCLC**	685	335	97.93	0.62	[0.78, 2.50]
	**Breast**	188	185	98.55	**Melanoma**	91	91	99.27	−0.72	[-1.49, 0.82]
	**Breast**	188	185	98.55	**Ovary**	877	238	98.15	0.40	[-0.54, 1]
	**Breast**	188	185	98.55	**Prostate**	782	290	99.24	−0.69	[-1.10, 0.41]
	**CRC**	251	249	98.08	**NSCLC**	685	335	97.93	0.15	[0.02, 1.85]
	**CRC**	251	249	98.08	**Melanoma**	91	91	99.27	−1.19	[-2.24, 0.16]
	**CRC**	251	249	98.08	**Ovary**	877	238	98.15	−0.07	[-1.31, 0.35]
	**CRC**	251	249	98.08	**Prostate**	782	290	99.24	−1.16	[-1.87, -0.24]
	**NSCLC**	685	335	97.93	**Melanoma**	91	91	99.27	−1.34	[-3.01, -0.95]
	**NSCLC**	685	335	97.93	**Ovary**	877	238	98.15	−0.22	[-1.96, -0.86]
	**NSCLC**	685	335	97.93	**Prostate**	782	290	99.24	−1.32	[-2.52, -1.46]
	**Melanoma**	91	91	99.27	**Ovary**	877	238	98.15	1.12	[-0.39, 1.52]
	**Melanoma**	91	91	99.27	**Prostate**	782	290	99.24	0.03	[-0.96, 0.93]
	**Ovary**	877	238	98.15	**Prostate**	782	290	99.24	−1.09	[-0.94, -0.22]
**Indel**	**CRC**	92	88	99.28	**NSCLC**	96	43	97.21	2.07	[1.16, 4.11]
	**CRC**	92	88	99.28	**Ovary**	123	34	96.29	2.98	[0.74, 3.37]
	**CRC**	92	88	99.28	**Prostate**	113	48	97.77	1.50	[0.06, 2.22]
	**NSCLC**	96	43	97.21	**Ovary**	123	34	96.29	0.91	[-2.50, 1.35]
	**NSCLC**	96	43	97.21	**Prostate**	113	48	97.77	−0.57	[-3.27, 0.29]
	**Ovary**	123	34	96.29	**Prostate**	113	48	97.77	−1.48	[-2.56, 0.73]

CI = confidence interval; Indel = insertions/deletions; CRC = colorectal; NSCLC = non-small cell lung carcinoma.

**Table 2:** Pairwise difference of mean repeatability (%) between tumor types for each SV type (substitution and indel).

Variant Type	1^st^ Tumor Type	# of Precision Data Points of 1^st^ Tumor Type	# of Unique Variants of 1^st^ Tumor Type	Mean Repeatability (%) of 1^st^ Tumor Type	2^nd^ Tumor Type	# of Precision Data Points of 2^nd^ Tumor Type	# of Unique Variants of 2^nd^ Tumor Type	Mean Repeatability (%) of 2^nd^ Tumor Type	Mean Difference in Repeatability (%) between 1^st^ and 2^nd^ Tumor Type	Two-sided 95% CI for Mean Difference (%)
**Substitution**	**Breast**	188	185	98.55	**CRC**	251	249	98.08	0.47	[-1.02, 1.96]
	**Breast**	188	185	98.55	**NSCLC**	685	335	97.93	0.62	[-0.67, 1.91]
	**Breast**	188	185	98.55	**Melanoma**	91	91	99.27	−0.72	[-2.3, 0.86]
	**Breast**	188	185	98.55	**Ovary**	877	238	98.15	0.40	[-0.80, 1.60]
	**Breast**	188	185	98.55	**Prostate**	782	290	99.24	−0.69	[-1.88, 0.50]
	**CRC**	251	249	98.08	**NSCLC**	685	335	97.93	0.15	[-1, 1.30]
	**CRC**	251	249	98.08	**Melanoma**	91	91	99.27	−1.19	[-2.66, 0.29]
	**CRC**	251	249	98.08	**Ovary**	877	238	98.15	−0.07	[-1.12, 0.98]
	**CRC**	251	249	98.08	**Prostate**	782	290	99.24	−1.16	[-2.20, -0.12]
	**NSCLC**	685	335	97.93	**Melanoma**	91	91	99.27	−1.34	[-2.61, -0.07]
	**NSCLC**	685	335	97.93	**Ovary**	877	238	98.15	−0.22	[-0.95, 0.51]
	**NSCLC**	685	335	97.93	**Prostate**	782	290	99.24	−1.32	[-2.03, -0.60]
	**Melanoma**	91	91	99.27	**Ovary**	877	238	98.15	1.12	[-0.06, 2.30]
	**Melanoma**	91	91	99.27	**Prostate**	782	290	99.24	0.03	[-1.15, 1.20]
	**Ovary**	877	238	98.15	**Prostate**	782	290	99.24	−1.09	[-1.63, -0.56]
**Indel**	**CRC**	92	88	99.28	**NSCLC**	96	43	97.21	2.07	[0.05, 4.09]
	**CRC**	92	88	99.28	**Ovary**	123	34	96.29	2.98	[1.25, 4.72]
	**CRC**	92	88	99.28	**Prostate**	113	48	97.77	1.50	[-0.23, 3.23]
	**NSCLC**	96	43	97.21	**Ovary**	123	34	96.29	0.91	[-1.63, 3.45]
	**NSCLC**	96	43	97.21	**Prostate**	113	48	97.77	−0.57	[-3.10, 1.97]
	**Ovary**	123	34	96.29	**Prostate**	113	48	97.77	−1.48	[-3.80, 0.84]

CI = confidence interval; Indel = insertions/deletions; CRC = colorectal; NSCLC = non-small cell lung carcinoma.

#### Pairwise difference of mean %CV of reproducibility and repeatability.

A total of 1,477 data points of %CV of reproducibility and %CV repeatability for base substitutions were included in this assessment.

The mean reproducibility %CV and mean repeatability %CV using the underlying VAF for each tumor type as well as the pairwise differences with 95% 2-sided CIs based on bootstrap method are presented in S1 and S2 Tables in S1 File. In summary, the median absolute pairwise differences of mean reproducibility %CV is 2.16%, and it ranges from 0.44% to 4.32%; and the median absolute pairwise difference of mean repeatability % CV is 1.91%, and it ranges from 0.32% to 3.82%.

### Concordance results

A total of 3,082 data points of SVs detection status by F1LCDx and the orthogonal reference assay from concordance studies were included in this assessment. There were 61 DOs from these samples. Similarly, upon grouping these DOs per FMI’s CGR dataset grouping names, there were five (5) tumor types (Breast, CRC, NSCLC, Prostate, and Unknown primary carcinoma (cup)) with at least 100 data points were included in the assessment. Tumor types with less than 100 data points were grouped into the “Other” category and was also included in the assessment.

#### Pairwise difference of PPA and NPA.

For each tumor type, the PPA and NPA at variant level were calculated. And for each pair of tumor type, the difference between PPA (i.e., $\partial_{PPA}$) and NPA (i.e., $\partial_{NPA}$) were calculated, along with the 95% 2-sided score CIs. The results are summarized in Table 3 below. In summary, the median absolute $\partial_{PPA}$ is 1.39%, ranges from 0.10% to 4.10%. And the median absolute $\partial_{NPA}$ is 0.05%, ranges from 0% to 1%. The consistent small difference values of PPA and NPA suggest that the tumor type does not influence the results of concordance studies.

**Table 3:** Summary of $\delta_{PPA}$ and $\delta_{NPA}$ between tumor types.

1^st^ Tumor Type	2^nd^ Tumor Type	$\partial_{PPA}$ (%)	95% 2-sided score CI for $\partial_{PPA}$ (%)	$\partial_{NPA}$ (%)	95% 2-sided score CI for $\partial_{NPA}$ (%)
**Breast**	**CRC**	0.79	(−3.79, 6.74)	−0.02	(−0.07, 0.02)
**Breast**	**NSCLC**	2.71	(−1.92, 7.14)	−0.03	(−0.07, 0.01)
**Breast**	**Prostate**	−0.10	(−4.59, 7.37)	0	(−0.05, 0.05)
**Breast**	**Unknown primary carcinoma (cup)**	0.67	(−3.67, 4.57)	0.93	(0.83, 1.03)
**Breast**	**Others**	−1.39	(−5.47, 2.26)	−0.07	(−0.11, −0.04)
**CRC**	**NSCLC**	1.92	(−4.27, 6.61)	−0.01	(−0.05, 0.04)
**CRC**	**Prostate**	−0.89	(−6.98, 6.74)	0.02	(−0.03, 0.08)
**CRC**	**Unknown primary carcinoma (cup)**	−0.12	(−6.1, 4.08)	0.95	(0.86, 1.06)
**CRC**	**Others**	2.18	(−1.79, 7.97)	0.05	(0.01, 0.09)
**NSCLC**	**Prostate**	−2.81	(−7.41, 4.85)	0.03	(−0.02, 0.08)
**NSCLC**	**Unknown primary carcinoma (cup)**	−2.04	(−6.5, 2.21)	0.96	(0.87, 1.06)
**NSCLC**	**Others**	4.10	(0.07, 8.31)	0.04	(0.01, 0.07)
**Prostate**	**Unknown primary carcinoma (cup)**	0.77	(−6.72, 4.87)	0.93	(0.83, 1.04)
**Prostate**	**Others**	1.29	(−2.57, 8.63)	0.07	(0.02, 0.11)
**Unknown primary carcinoma (cup)**	**Others**	2.06	(−1.63, 5.71)	1	(0.91, 1.10)

PPA = positive percent agreement; NPA = negative percent agreement; $\partial_{PPA}=difference\;between\;two\;tumor\;types\;in\;PPA$; $\partial_{NPA}=difference\;between\;two\;tumor\;types\;in\;NPA$; CI = confidence interval; CRC = colorectal; NSCLC = non-small cell lung carcinoma.

### Coverage results

Coverage analysis was performed for the 31,247 clinical samples and included target-level, variant-level, and sample-level coverage. Samples were mapped and aggregated into tumor type groups based on FMI’s genomics dataset grouping names as well as common histological characteristics as determined by board-certified pathologists at FMI. For sample-level and target-level coverage, tumor types with at least 500 samples with QC = Pass or at least 100 samples with QC = Qualified were included in the assessment; for variant-level coverage, tumor types with at least 500 samples with QC = Pass or at least 500 samples with QC = Qualified were included in the assessment.

#### Target-level coverage.

**Fig 1:**
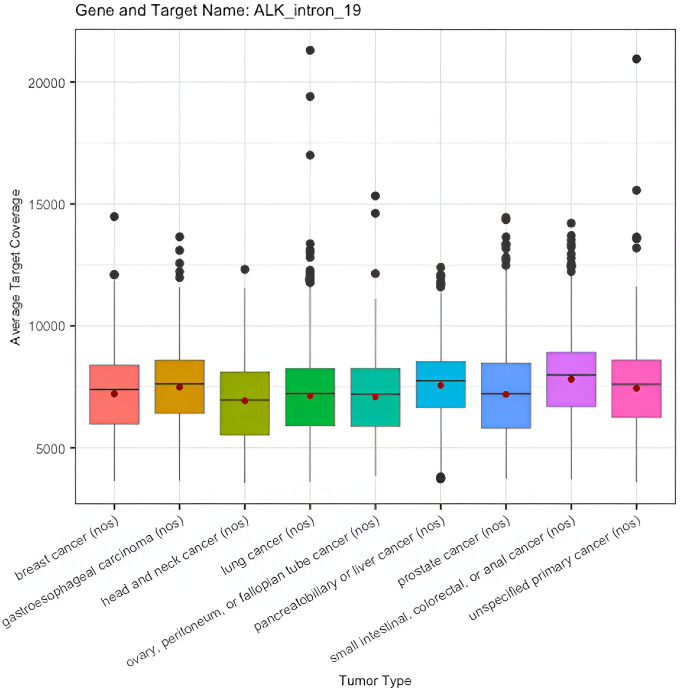
Distribution of target-level coverage of *ALK*_intron_19 for clinical samples (QC = Pass).

A total of 118 target regions for 13 genes (including *ALK*, *ATM*, *BRCA1*, *BRCA2*, *EGFR*, *ERBB2*, *FGFR2*, *MET*, *NTRK1*, *NTRK2*, *NTRK3*, *PIK3CA*, and *ROS1*) were evaluated for the target-level coverage distribution between tumor types among clinical samples. For example, in *ALK* intron 19, the boxplots of the target-level coverage distribution between tumor types are provided below (*ALK* intron 19 target-level coverage for clinical samples with QC status = Pass (Fig 1), clinical samples with QC status = Qualified (S1 Fig in S2 File). The descriptive statistics of the target-level coverage of ALK intron 19 for clinical samples with QC status pass or qualified is provided in S3 and S4 Tables in S1 File, respectively.

Similar distribution is observed between tumor types using clinical samples that had post-sequencing QC status pass or qualified. And similar performance is observed for other target regions for all genes (refer to S4 and S5 Figs in S2 File for the full box plots and S17 and S18 Tables in S1 File for the descriptive statistics of the target-level coverage distribution for all genes and target regions assessed).

#### Variant-level coverage.

**Fig 2:**
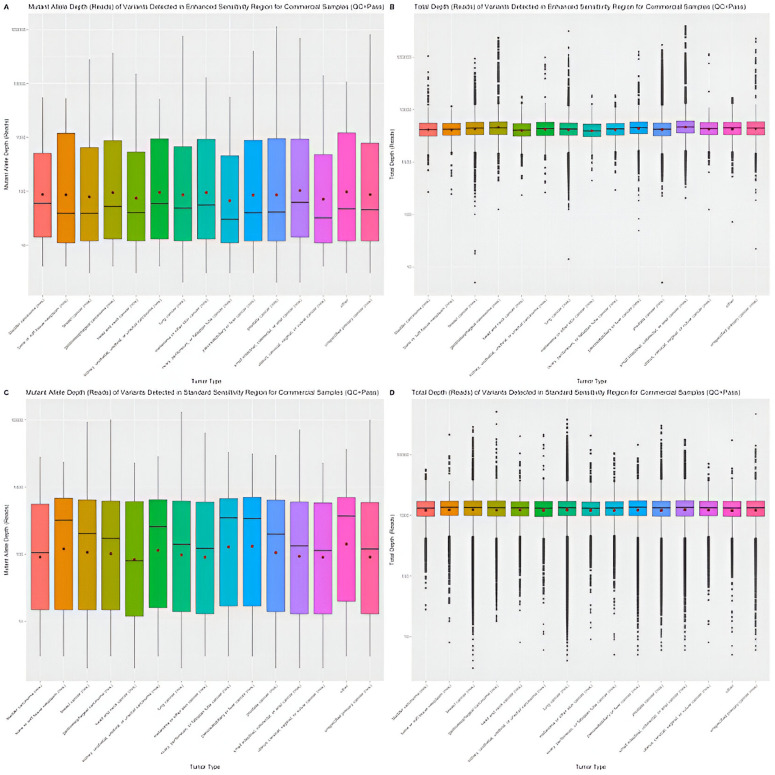
Distribution of variant-level coverage for clinical samples (QC  = Pass).

Distribution of variant-level coverage (mutant allele depth and total depth) are evaluated for clinical samples with QC status pass and qualified, separately. The descriptive statistics of the mutant allele depth and total depth of variants detected in enhanced sensitivity region or standard sensitivity region for clinical samples with QC status pass or qualified is provided in S5-S12 Tables in S1 File, respectively. Similar distribution of the distribution between tumor types is observed using clinical samples that had post-sequencing QC status pass (Fig 2) or qualified (S2 Fig in S2 File).

#### Sample-level coverage.

**Fig 3:**
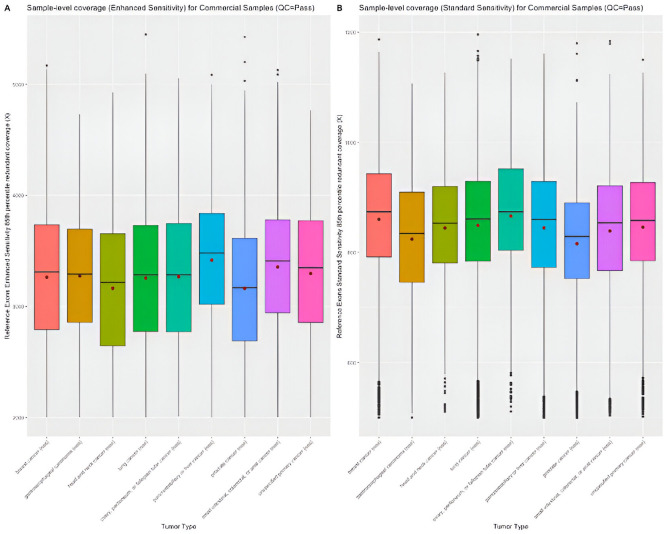
Distribution of sample-level coverage for clinical samples (QC= Pass).

Distribution of sample-level coverage at enhanced sensitivity region and standard sensitivity region is evaluated for clinical samples with QC status pass and qualified. The descriptive statistics of the sample-level coverage at enhanced sensitivity region or standard sensitivity region for clinical samples with QC status pass or qualified is provided in S13-S16 Tables in S1 File, respectively. Similar distribution of the sample-level coverage between tumor types is observed using clinical samples that had post-sequencing QC status pass (Fig 3) or qualified (S3 Fig in S2 File).

## Discussion

In this study, we provided evidence to demonstrate the F1LCDx assay performance is agnostic to tumor type and that large sets of clinical, tumor-specific samples would not be necessary for future AV studies. This evidence was found by studying a large cohort of clinical samples (n = 31,247) and AV samples (n = 579) across multiple tumor types. First, we examined the effect of tumor type on precision (reproducibility and repeatability) and concordance results. Here, we observed high mean reproducibility and mean repeatability results as well as PPA rates of 94%−98% and a NPA > 98% for each tumor type and small pairwise difference of mean reproducibility (≤2.63%), mean repeatability (≤2.98%), PPA (≤4.1%), and NPA (≤1%) between any pair of the tumor types. Consistently high concordance and precision values suggest that the tumor type does not influence the results of AV studies. Mechanistically, we hypothesize that the consistency of these endpoints is due to the use of the same procurement method for the liquid biopsies across all the different tumor types. For this assay, we use the same type of collection tube, method, and 14-day testing window once the blood specimen is procured regardless of tumor type. This contrasts with tissue biopsy where the methodology of procurement and time from sample procurement and testing can vary [14]. In addition, from a biological standpoint, once ctDNA is in the blood, the ctDNA is presumably no longer burdened with interferences due to local tumor environment and likely does not suffer from variability due to intratumoral heterogeneity. The strong and consistent analytical precision and concordance performance results across different tumor types support our hypothesis that once tumor DNA is shed into blood stream, the DNA is just DNA, and tumor type has little to no effect on the analytical precision or concordance performance of F1LCDx results.

In addition to examining precision and concordance from the AV studies, we also examined the coverage of sequencing across tumor types by examining a large clinical cohort of >30,000 cases as additional evidence. While it has been established that the tumor shedding levels vary in different tumor types, the analytic coverage analysis shows that the coverage across a large real-world data set is consistent despite this difference in tumor shedding [15,16]. Here we specifically examined target-level coverage of regions in 13 genes and across 577 target regions, variant-level coverage, and sample-level coverage, all with similar coverage across the tumor types.

One of the limitations of this study is that the data may not fully represent rare histologies, as these rare tumor groups were analyzed together as ‘Other’ tumor group for a larger sample size given the limitation of available data points in each rare histology group. Another limitation is that only 13 genes out of the 324 genes baited by F1LCDx were assessed in this study. They were selected because they are FDA approved CDx biomarkers on F1LCDx and hence they are the most clinically actionable biomarkers. In addition, various variant classes are covered by these genes, including short variants, rearrangements and fusions, and copy number alterations, which are representative of all variant classes that are reported by the F1LCDx assay.

Furthermore, all of the samples in this cohort were tested with a large targeted NGS liquid-based assay, which narrows the scope of our conclusions, meaning that it is uncertain if these results would have been the same with a different NGS based assay such as whole-genome sequencing. This is also one of the strengths of the study, because we were able to assess these various metrics on a cohort of samples that has consistent procurement and testing methodologies. However, more studies should be performed to see if this is applicable to other types of liquid biopsy-based assays.

## Conclusions

Herein, the results based on the extensive cohort of 31,826 liquid biopsy samples sequenced by the F1LCDx assay presented here demonstrated that both analytical assessment of precision and concordance and analytical coverage are comparable among tumor types. Performance was found to be agnostic to tumor types. These data show that for future NGS AV studies utilizing the F1LCDx assay, tumor type should not impact the assay performance and that tumor type is not an important consideration for pan-solid tumor AV studies.

## Supporting information

S1 File Supplement tables. (ZIP)

S2 File Supplement figures. (ZIP)
