# Supplementary material for: Analytical validation and sequencing coverage studies suggest that performance of a liquid biopsy assay is tumor agnostic (DNA-is-DNA)
Source: PLoS One. 2025 Aug 1;20(8):e0329392. doi: 10.1371/journal.pone.0329392 (PMC12316276; doi:10.1371/journal.pone.0329392)
Supplement: S1 File — (ZIP) [file pone.0329392.s001.zip › Supplement Tables/Supplement Tables.docx]

**Supplement Tables**

**S1 Table. Pairwise difference of mean %CV of reproducibility between tumor type for substitutions.**

| **Variant Type** | **1^st^ Tumor Type** | **# of Precision Data Points of 1^st^ Tumor Type** | **# of Unique Variants of 1^st^ Tumor Type** | **Mean Reproducibility (VAF %CV) of 1^st^ Tumor Type** | **2^nd^ Tumor Type** | **# of Precision Data Points of 2^nd^ Tumor Type** | **# of Unique Variants of 2^nd^ Tumor Type** | **Mean Reproducibility (VAF %CV) of 2^nd^ Tumor Type** | **Mean Difference in Reproducibility (VAF %CV) between 1^st^ and 2^nd^ Tumor Type** | **Two-sided 95% CI for Mean Difference (%)** |
| --- | --- | --- | --- | --- | --- | --- | --- | --- | --- | --- |
| **Substitution** | **Breast** | 135 | 135 | 23.20 | **NSCLC** | 163 | 161 | 18.86 | 4.35 | [1.55, 7.09] |
|  | **Breast** | 135 | 135 | 23.20 | **Ovary** | 528 | 151 | 21.83 | 1.37 | [-1.12, 3.85] |
|  | **Breast** | 135 | 135 | 23.20 | **Prostate** | 650 | 164 | 21.39 | 1.81 | [-0.65, 4.27] |
|  | **NSCLC** | 163 | 161 | 18.86 | **Ovary** | 528 | 151 | 21.83 | -2.98 | [-4.68, -1.22] |
|  | **NSCLC** | 163 | 161 | 18.86 | **Prostate** | 650 | 164 | 21.39 | -2.54 | [-4.2, -0.81] |
|  | **Ovary** | 528 | 151 | 21.83 | **Prostate** | 650 | 164 | 21.39 | 0.44 | [-0.71, 1.59] |

VAF=variant allele frequency; CV=coefficient of variation; CI=confidence interval; NSCLC=non-small cell lung carcinoma.

**S2 Table. Pairwise difference of mean %CV of repeatability between tumor type for substitutions.**

| **Variant Type** | **1^st^ Tumor Type** | **# of Precision Data Points of 1^st^ Tumor Type** | **# of Unique Variants of 1^st^ Tumor Type** | **Mean Repeatability (VAF %CV) of 1^st^ Tumor Type** | **2^nd^ Tumor Type** | **# of Precision Data Points of 2^nd^ Tumor Type** | **# of Unique Variants of 2^nd^ Tumor Type** | **Mean Repeatability (VAF %CV) of 2^nd^ Tumor Type** | **Mean Difference in Repeatability (VAF %CV) between 1^st^ and 2^nd^ Tumor Type** | **Two-sided 95% CI for Mean Difference (%)** |
| --- | --- | --- | --- | --- | --- | --- | --- | --- | --- | --- |
| **Substitution** | **Breast** | 135 | 135 | 20.89 | **NSCLC** | 163 | 161 | 17.03 | 3.86 | [1.27, 6.37] |
|  | **Breast** | 135 | 135 | 20.89 | **Ovary** | 528 | 151 | 19.65 | 1.24 | [-1.01, 3.49] |
|  | **Breast** | 135 | 135 | 20.89 | **Prostate** | 650 | 164 | 19.33 | 1.56 | [-0.68, 3.8] |
|  | **NSCLC** | 163 | 161 | 17.03 | **Ovary** | 528 | 151 | 19.65 | -2.62 | [-4.21, -0.96] |
|  | **NSCLC** | 163 | 161 | 17.03 | **Prostate** | 650 | 164 | 19.33 | -2.3 | [-3.87, -0.65] |
|  | **Ovary** | 528 | 151 | 19.65 | **Prostate** | 650 | 164 | 19.33 | 0.32 | [-0.74, 1.38] |

VAF=variant allele frequency; CV=coefficient of variation; CI=confidence interval; NSCLC=non-small cell lung carcinoma.

**S3 Table. Summary statistics of target level coverage of *ALK*_intron_19 for clinical samples (QC=Pass).**

| **Tumor Type** | **N of Samples** | **Min** | **Q1** | **Median** | **Mean** | **Q3** | **Max** | **SD** |
| --- | --- | --- | --- | --- | --- | --- | --- | --- |
| **Breast cancer (nos)** | 3654 | 3638.78 | 5979.11 | 7391.58 | 7215.21 | 8385.16 | 14482.64 | 1565.01 |
| **Gastroesophageal carcinoma (nos)** | 740 | 3653.67 | 6414.68 | 7622.95 | 7492.35 | 8592.82 | 13654.21 | 1571.73 |
| **Head and neck cancer (nos)** | 535 | 3563.49 | 5522.38 | 6956.02 | 6926.16 | 8104.70 | 12315.12 | 1621.52 |
| **Lung cancer (nos)** | 8102 | 3607.95 | 5900.51 | 7228.82 | 7133.07 | 8248.45 | 21299.16 | 1588.23 |
| **Ovary, peritoneum, or fallopian tube cancer (nos)** | 701 | 3842.48 | 5879.68 | 7191.99 | 7094.50 | 8252.25 | 15331.00 | 1599.36 |
| **Pancreatobiliary or liver cancer (nos)** | 3097 | 3724.97 | 6651.17 | 7751.61 | 7561.28 | 8538.47 | 12398.83 | 1410.72 |
| **Prostate cancer (nos)** | 4683 | 3736.46 | 5807.96 | 7217.70 | 7190.77 | 8469.44 | 14442.83 | 1694.76 |
| **Small intestinal, colorectal, or anal cancer (nos)** | 2774 | 3703.07 | 6695.75 | 7986.89 | 7810.95 | 8908.38 | 14209.97 | 1635.98 |
| **Unspecified primary cancer (nos)** | 1542 | 3605.98 | 6249.46 | 7602.28 | 7440.30 | 8596.99 | 20948.48 | 1668.51 |

Min=minimum; Q1=1^st^ quartile; Q3=3^rd^ quartile; Max=maximum; SD=standard deviation.

**S4 Table. Summary statistics of target level coverage of *ALK*_intron_19 for clinical samples (QC=Qualified).**

| **Tumor Type** | **N of Samples** | **Min** | **Q1** | **Median** | **Mean** | **Q3** | **Max** | **SD** |
| --- | --- | --- | --- | --- | --- | --- | --- | --- |
| **Breast cancer (nos)** | 374 | 380.48 | 3442.86 | 3933.65 | 4575.65 | 4927.22 | 13111.77 | 2003.18 |
| **Lung cancer (nos)** | 913 | 419.96 | 3449.64 | 3925.81 | 4491.94 | 4876.07 | 12783.00 | 1851.61 |
| **Pancreatobiliary or liver cancer (nos)** | 218 | 238.11 | 3576.38 | 4244.69 | 5143.94 | 6976.84 | 14128.18 | 2282.04 |
| **Prostate cancer (nos)** | 674 | 725.39 | 3475.15 | 4000.29 | 4686.21 | 5170.42 | 12302.04 | 2054.48 |
| **Small intestinal, colorectal, or anal cancer (nos)** | 228 | 507.80 | 3770.76 | 4423.05 | 5302.47 | 7233.97 | 12367.48 | 2321.57 |
| **Unspecified primary cancer (nos)** | 143 | 277.02 | 3531.98 | 4092.24 | 4565.93 | 5059.62 | 10433.20 | 1815.18 |

Min=minimum; Q1=1^st^ quartile; Q3=3^rd^ quartile; Max=maximum; SD=standard deviation.

**S5 Table. Summary statistics of mutant allele depth (reads) for variants detected in enhanced sensitivity region for clinical samples (QC=Pass).**

| **Tumor Type** | **N of Samples** | **Min** | **Q1** | **Median** | **Mean** | **Q3** | **Max** | **SD** |
| --- | --- | --- | --- | --- | --- | --- | --- | --- |
| **Bladder carcinoma (nos)** | 1794 | 4 | 14 | 59.50 | 515.13 | 505.75 | 5534 | 914.04 |
| **Bone or soft tissue neoplasm (nos)** | 1315 | 4 | 11 | 39 | 644.92 | 1184.50 | 5349 | 1019.02 |
| **Breast cancer (nos)** | 18614 | 0 | 12 | 39 | 566.44 | 640 | 28025 | 1038.28 |
| **Gastroesophageal carcinoma (nos)** | 3353 | 4 | 13 | 52 | 667.90 | 863 | 37210 | 1525.18 |
| **Head and neck cancer (nos)** | 2322 | 3 | 12 | 40 | 547.88 | 527.50 | 15247 | 1043.53 |
| **Kidney, urothelial, urethral, or urachal carcinoma (nos)** | 1295 | 4 | 13 | 59 | 625.35 | 940.50 | 5212 | 1014.79 |
| **Lung cancer (nos)** | 36841 | 2 | 12 | 49 | 597.62 | 664 | 76024 | 1271.60 |
| **Melanoma or other skin cancer (nos)** | 1432 | 3 | 13 | 55 | 628.67 | 920 | 13070 | 1090.84 |
| **Other** | 1378 | 3 | 12 | 47 | 694.25 | 1197.25 | 10885 | 1100.30 |
| **Ovary, peritoneum, or fallopian tube cancer (nos)** | 3069 | 3 | 11 | 30 | 537.33 | 449 | 5673 | 978.82 |
| **Pancreatobiliary or liver cancer (nos)** | 11425 | 3 | 12 | 40 | 651.73 | 880 | 40108 | 1144.11 |
| **Prostate cancer (nos)** | 19972 | 0 | 12 | 41 | 641.13 | 947.25 | 116475 | 1575.29 |
| **Small intestinal, colorectal, or anal cancer (nos)** | 17394 | 2 | 14 | 62 | 644.29 | 926.75 | 70311 | 1180.51 |
| **Unspecified primary cancer (nos)** | 7587 | 3 | 12 | 45 | 607.94 | 777.50 | 82344 | 1449.26 |
| **Uterus, cervical, vaginal, or vulvar cancer (nos)** | 2231 | 4 | 11 | 32 | 543.92 | 473.50 | 14291 | 1015.36 |

Min=minimum; Q1=1^st^ quartile; Q3=3^rd^ quartile; Max=maximum; SD=standard deviation.

**S6 Table. Summary statistics of total depth (reads) for variants detected in enhanced sensitivity region for clinical samples (QC=Pass).**

| **Tumor Type** | **N of Samples** | **Min** | **Q1** | **Median** | **Mean** | **Q3** | **Max** | **SD** |
| --- | --- | --- | --- | --- | --- | --- | --- | --- |
| **Bladder carcinoma (nos)** | 1794 | 267 | 3151.25 | 4147 | 4590.98 | 5424.75 | 104228 | 3575.29 |
| **Bone or soft tissue neoplasm (nos)** | 1315 | 244 | 3251 | 4209 | 4395.71 | 5383 | 11500 | 1596.22 |
| **Breast cancer (nos)** | 18614 | 5 | 3334 | 4398 | 4698.11 | 5671.75 | 94150 | 2416.54 |
| **Gastroesophageal carcinoma (nos)** | 3353 | 124 | 3363 | 4464 | 6216.73 | 5789 | 232604 | 12760.61 |
| **Head and neck cancer (nos)** | 2322 | 233 | 3121 | 4064.50 | 4295.73 | 5323.50 | 17703 | 1640.86 |
| **Kidney, urothelial, urethral, or urachal carcinoma (nos)** | 1295 | 261 | 3198 | 4344 | 4935.67 | 5684.50 | 98981 | 5552.67 |
| **Lung cancer (nos)** | 36841 | 14 | 3249 | 4239 | 4527.32 | 5472 | 312029 | 2981.26 |
| **Melanoma or other skin cancer (nos)** | 1432 | 443 | 3039.75 | 3920.50 | 4282.05 | 5252.75 | 18447 | 1768.81 |
| **Other** | 1378 | 72 | 3354.5 | 4460 | 4646.29 | 5647.75 | 16467 | 1900.28 |
| **Ovary, peritoneum, or fallopian tube cancer (nos)** | 3069 | 293 | 3269 | 4268 | 4452.71 | 5375 | 18617 | 1663.70 |
| **Pancreatobiliary or liver cancer (nos)** | 11425 | 49 | 3475 | 4495 | 4769.48 | 5761 | 125897 | 2929.59 |
| **Prostate cancer (nos)** | 19972 | 5 | 3179 | 4198.50 | 4723.46 | 5471 | 170875 | 4181.05 |
| **Small intestinal, colorectal, or anal cancer (nos)** | 17394 | 204 | 3560.25 | 4662 | 5375.55 | 6030.75 | 389308 | 9156.90 |
| **Unspecified primary cancer (nos)** | 7587 | 22 | 3316 | 4414 | 4837.55 | 5736 | 224363 | 5001.85 |
| **Uterus, cervical, vaginal, or vulvar cancer (nos)** | 2231 | 123 | 3347.50 | 4348 | 4666.78 | 5611 | 113381 | 3890.60 |

Min=minimum; Q1=1^st^ quartile; Q3=3^rd^ quartile; Max=maximum; SD=standard deviation.

**S7 Table. Summary statistics of mutant allele depth (reads) for variants detected in standard sensitivity region for clinical samples (QC=Pass).**

| **Tumor Type** | **N of Samples** | **Min** | **Q1** | **Median** | **Mean** | **Q3** | **Max** | **SD** |
| --- | --- | --- | --- | --- | --- | --- | --- | --- |
| **Bladder carcinoma (nos)** | 3650 | 3 | 15 | 105.50 | 296.71 | 557 | 2843 | 351.17 |
| **Bone or soft tissue neoplasm (nos)** | 3195 | 3 | 15 | 322 | 382.26 | 679 | 2386 | 378.86 |
| **Breast cancer (nos)** | 35177 | 2 | 15 | 203 | 351.16 | 645 | 9398 | 379.14 |
| **Gastroesophageal carcinoma (nos)** | 7109 | 3 | 15 | 172 | 334.79 | 618 | 10226 | 382.94 |
| **Head and neck cancer (nos)** | 5700 | 2 | 12 | 80 | 308.90 | 604 | 2328 | 361.75 |
| **Kidney, urothelial, urethral, or urachal carcinoma (nos)** | 3553 | 3 | 16 | 258 | 361.82 | 649 | 2892 | 372.35 |
| **Lung cancer (nos)** | 82670 | 0 | 14 | 141 | 329.76 | 617 | 13289 | 370.94 |
| **Melanoma or other skin cancer (nos)** | 3429 | 3 | 13 | 122 | 313.51 | 596 | 6516 | 372.32 |
| **Other** | 3467 | 0 | 20 | 370 | 406.04 | 697.50 | 3720 | 384.94 |
| **Ovary, peritoneum, or fallopian tube cancer (nos)** | 5769 | 3 | 17 | 351 | 387.16 | 672 | 3339 | 373.84 |
| **Pancreatobiliary or liver cancer (nos)** | 24763 | 3 | 17 | 342 | 396.43 | 702 | 3174 | 382.91 |
| **Prostate cancer (nos)** | 43682 | 2 | 14 | 199 | 349.24 | 640 | 3041 | 374.24 |
| **Small intestinal, colorectal, or anal cancer (nos)** | 30359 | 2 | 13 | 134 | 322.26 | 596 | 7186 | 375.16 |
| **Unspecified primary cancer (nos)** | 16744 | 3 | 13 | 120 | 316.59 | 588.25 | 10163 | 373.33 |
| **Uterus, cervical, vaginal, or vulvar cancer (nos)** | 4797 | 2 | 13 | 114 | 309.92 | 582 | 2328 | 352.36 |

Min=minimum; Q1=1^st^ quartile; Q3=3^rd^ quartile; Max=maximum; SD=standard deviation.

**S8 Table. Summary statistics of total depth (reads) for variants detected in standard sensitivity region for clinical samples (QC=Pass).**

| **Tumor Type** | **N of Samples** | **Min** | **Q1** | **Median** | **Mean** | **Q3** | **Max** | **SD** |
| --- | --- | --- | --- | --- | --- | --- | --- | --- |
| **Bladder carcinoma (nos)** | 3650 | 28 | 962.25 | 1315 | 1349.24 | 1680.75 | 5665 | 575.71 |
| **Bone or soft tissue neoplasm (nos)** | 3195 | 8 | 999 | 1355 | 1381.96 | 1702.50 | 21374 | 720.72 |
| **Breast cancer (nos)** | 35177 | 3 | 994 | 1345 | 1389.29 | 1711 | 28529 | 712.29 |
| **Gastroesophageal carcinoma (nos)** | 7109 | 18 | 973 | 1329 | 1383.97 | 1711 | 50988 | 998.01 |
| **Head and neck cancer (nos)** | 5700 | 8 | 983 | 1325 | 1348.86 | 1661 | 20178 | 622.09 |
| **Kidney, urothelial, urethral, or urachal carcinoma (nos)** | 3553 | 6 | 956 | 1315 | 1359.39 | 1682 | 21299 | 816.35 |
| **Lung cancer (nos)** | 82670 | 4 | 988 | 1330 | 1367.70 | 1690 | 37965 | 681.77 |
| **Melanoma or other skin cancer (nos)** | 3429 | 9 | 985 | 1308 | 1352.20 | 1648 | 20613 | 708.79 |
| **Other** | 3467 | 5 | 961 | 1319 | 1347.83 | 1682.50 | 17283 | 637.31 |
| **Ovary, peritoneum, or fallopian tube cancer (nos)** | 5769 | 5 | 993 | 1320 | 1347.37 | 1669 | 10590 | 619.63 |
| **Pancreatobiliary or liver cancer (nos)** | 24763 | 5 | 986 | 1362 | 1372.31 | 1728 | 14568 | 588.41 |
| **Prostate cancer (nos)** | 43682 | 6 | 967 | 1328 | 1359.97 | 1701 | 29967 | 686.07 |
| **Small intestinal, colorectal, or anal cancer (nos)** | 30359 | 6 | 977 | 1345 | 1386.48 | 1721 | 17624 | 671.53 |
| **Unspecified primary cancer (nos)** | 16744 | 6 | 965 | 1324 | 1362.53 | 1701 | 46702 | 711.95 |
| **Uterus, cervical, vaginal, or vulvar cancer (nos)** | 4797 | 8 | 987 | 1331 | 1352.42 | 1691 | 7043 | 550.72 |

Min=minimum; Q1=1^st^ quartile; Q3=3^rd^ quartile; Max=maximum; SD=standard deviation.

**S9 Table. Summary statistics of mutant allele depth (reads) for variants detected in enhanced sensitivity region for clinical samples (QC=Qualified).**

| **Tumor Type** | **N of Samples** | **Min** | **Q1** | **Median** | **Mean** | **Q3** | **Max** | **SD** |
| --- | --- | --- | --- | --- | --- | --- | --- | --- |
| **Breast cancer (nos)** | 1336 | 3 | 9 | 30.50 | 386.04 | 659.50 | 4093 | 632.84 |
| **Lung cancer (nos)** | 3504 | 2 | 9 | 32 | 357.13 | 424.50 | 16097 | 710.04 |
| **Pancreatobiliary or liver cancer (nos)** | 727 | 2 | 10 | 30 | 448.06 | 787.50 | 4120 | 753.12 |
| **Prostate cancer (nos)** | 2149 | 2 | 10 | 43 | 437.61 | 745 | 11639 | 788.41 |
| **Small intestinal, colorectal, or anal cancer (nos)** | 1201 | 3 | 13 | 60 | 503.13 | 518 | 49441 | 1892.37 |
| **Unspecified primary cancer (nos)** | 521 | 2 | 8 | 29 | 345.48 | 350 | 4028 | 631.34 |

Min=minimum; Q1=1^st^ quartile; Q3=3^rd^ quartile; Max=maximum; SD=standard deviation.

**S10 Table. Summary statistics of total depth (reads) for variants detected in enhanced sensitivity region for clinical samples (QC=Qualified).**

| **Tumor Type** | **N of Samples** | **Min** | **Q1** | **Median** | **Mean** | **Q3** | **Max** | **SD** |
| --- | --- | --- | --- | --- | --- | --- | --- | --- |
| **Breast cancer (nos)** | 1336 | 118 | 1754.75 | 2209.50 | 2703.92 | 3066.50 | 13913 | 1651.47 |
| **Lung cancer (nos)** | 3504 | 15 | 1723 | 2235 | 2659.59 | 3057 | 102471 | 2708.40 |
| **Pancreatobiliary or liver cancer (nos)** | 727 | 6 | 1793.50 | 2345 | 3057.26 | 3672.50 | 77884 | 3880 |
| **Prostate cancer (nos)** | 2149 | 148 | 1741 | 2278 | 2816.96 | 3233 | 97233 | 2808.16 |
| **Small intestinal, colorectal, or anal cancer (nos)** | 1201 | 144 | 2003 | 2722 | 4874.14 | 4374 | 190636 | 11734.38 |
| **Unspecified primary cancer (nos)** | 521 | 121 | 1769 | 2249 | 2527.94 | 2871 | 7696 | 1333.93 |

Min=minimum; Q1=1^st^ quartile; Q3=3^rd^ quartile; Max=maximum; SD=standard deviation.

**S11 Table. Summary statistics of mutant allele depth (reads) for variants detected in standard sensitivity region for clinical samples (QC=Qualified).**

| **Tumor Type** | **N of Samples** | **Min** | **Q1** | **Median** | **Mean** | **Q3** | **Max** | **SD** |
| --- | --- | --- | --- | --- | --- | --- | --- | --- |
| **Bladder carcinoma (nos)** | 565 | 3 | 10 | 33 | 175.49 | 368 | 1211 | 230.90 |
| **Breast cancer (nos)** | 3154 | 2 | 12 | 208.50 | 261.40 | 470 | 1607 | 257.60 |
| **Gastroesophageal carcinoma (nos)** | 633 | 2 | 21 | 164 | 247.31 | 412 | 1283 | 241.57 |
| **Head and neck cancer (nos)** | 551 | 2 | 14 | 242 | 263.58 | 472.50 | 1148 | 247.59 |
| **Lung cancer (nos)** | 8461 | 2 | 12 | 129 | 242.08 | 440 | 5197 | 270.17 |
| **Ovary, peritoneum, or fallopian tube cancer (nos)** | 603 | 2 | 17 | 209 | 253.14 | 444 | 1163 | 236.94 |
| **Pancreatobiliary or liver cancer (nos)** | 1606 | 3 | 18 | 270 | 284.13 | 482 | 2756 | 268.04 |
| **Prostate cancer (nos)** | 5582 | 2 | 13 | 199 | 263.61 | 470 | 3586 | 269.58 |
| **Small intestinal, colorectal, or anal cancer (nos)** | 2200 | 3 | 13 | 110 | 235.98 | 422 | 1576 | 265.08 |
| **Unspecified primary cancer (nos)** | 1268 | 2 | 11 | 106.50 | 240.31 | 446.25 | 1449 | 264.71 |

Min=minimum; Q1=1^st^ quartile; Q3=3^rd^ quartile; Max=maximum; SD=standard deviation.

**S12 Table. Summary statistics of total depth (reads) for variants detected in standard sensitivity region for clinical samples (QC=Qualified).**

| **Tumor Type** | **N of Samples** | **Min** | **Q1** | **Median** | **Mean** | **Q3** | **Max** | **SD** |
| --- | --- | --- | --- | --- | --- | --- | --- | --- |
| **Bladder carcinoma (nos)** | 565 | 11 | 671 | 870 | 892.98 | 1068 | 13500 | 648.32 |
| **Breast cancer (nos)** | 3154 | 10 | 676 | 928 | 944.01 | 1142 | 26301 | 691.04 |
| **Gastroesophageal carcinoma (nos)** | 633 | 18 | 639 | 910 | 954.08 | 1167 | 5519 | 535.89 |
| **Head and neck cancer (nos)** | 551 | 30 | 672.50 | 903 | 896.99 | 1144.50 | 2911 | 368.46 |
| **Lung cancer (nos)** | 8461 | 12 | 655 | 911 | 967.12 | 1158 | 74403 | 1291.13 |
| **Ovary, peritoneum, or fallopian tube cancer (nos)** | 603 | 49 | 576.50 | 878 | 878.48 | 1131.50 | 2847 | 405.29 |
| **Pancreatobiliary or liver cancer (nos)** | 1606 | 9 | 633 | 902.5 | 942.08 | 1144 | 12521 | 680.36 |
| **Prostate cancer (nos)** | 5582 | 9 | 682 | 933 | 963.32 | 1178 | 20333 | 698.66 |
| **Small intestinal, colorectal, or anal cancer (nos)** | 2200 | 9 | 632 | 912 | 976.99 | 1190.25 | 27293 | 883.08 |
| **Unspecified primary cancer (nos)** | 1268 | 23 | 640.50 | 905.5 | 932.15 | 1164.25 | 14011 | 574.58 |

Min=minimum; Q1=1^st^ quartile; Q3=3^rd^ quartile; Max=maximum; SD=standard deviation.

**S13 Table. Summary statistics of sample level coverage (enhanced sensitivity) for clinical samples (QC=Pass).**

| **Tumor Type** | **N of Samples** | **Min** | **Q1** | **Median** | **Mean** | **Q3** | **Max** | **SD** |
| --- | --- | --- | --- | --- | --- | --- | --- | --- |
| **Breast cancer (nos)** | 3654 | 2001 | 2793.50 | 3310.50 | 3264.09 | 3735.75 | 5169 | 622.18 |
| **Gastroesophageal carcinoma (nos)** | 740 | 2001 | 2859 | 3292 | 3275.54 | 3695.25 | 4730 | 579.42 |
| **Head and neck cancer (nos)** | 535 | 2001 | 2646.50 | 3217 | 3164.06 | 3654.50 | 4928 | 628.66 |
| **Lung cancer (nos)** | 8102 | 2000 | 2775.25 | 3286 | 3254.85 | 3730 | 5449 | 625.59 |
| **Ovary, peritoneum, or fallopian tube cancer (nos)** | 701 | 2010 | 2773 | 3285 | 3267.47 | 3747 | 5055 | 632 |
| **Pancreatobiliary or liver cancer (nos)** | 3097 | 2000 | 3020 | 3482 | 3417.38 | 3838 | 5085 | 580.48 |
| **Prostate cancer (nos)** | 4683 | 2000 | 2692 | 3170 | 3164.58 | 3614 | 5426 | 613.22 |
| **Small intestinal, colorectal, or anal cancer (nos)** | 2774 | 2003 | 2945 | 3410 | 3355.21 | 3780 | 5130 | 588.67 |
| **Unspecified primary cancer (nos)** | 1542 | 2002 | 2857.25 | 3350.50 | 3297.42 | 3773 | 4768 | 620.34 |

Min=minimum; Q1=1^st^ quartile; Q3=3^rd^ quartile; Max=maximum; SD=standard deviation.

**S14 Table. Summary statistics of sample level coverage (standard sensitivity) for clinical samples (QC=Pass).**

| **Tumor Type** | **N of Samples** | **Min** | **Q1** | **Median** | **Mean** | **Q3** | **Max** | **SD** |
| --- | --- | --- | --- | --- | --- | --- | --- | --- |
| **Breast cancer (nos)** | 3654 | 500 | 792 | 874 | 860.18 | 943 | 1187 | 116.10 |
| **Gastroesophageal carcinoma (nos)** | 740 | 500 | 746 | 834.50 | 824.13 | 909.25 | 1107 | 118.13 |
| **Head and neck cancer (nos)** | 535 | 511 | 781 | 853 | 844.50 | 919.5 | 1127 | 109.51 |
| **Lung cancer (nos)** | 8102 | 500 | 784 | 861 | 849.11 | 929 | 1196 | 113.13 |
| **Ovary, peritoneum, or fallopian tube cancer (nos)** | 701 | 511 | 804 | 874 | 866.18 | 952 | 1152 | 115.06 |
| **Pancreatobiliary or liver cancer (nos)** | 3097 | 500 | 773 | 860 | 844.73 | 929 | 1161 | 117.18 |
| **Prostate cancer (nos)** | 4683 | 500 | 753 | 829 | 815.82 | 890 | 1180 | 105.42 |
| **Small intestinal, colorectal, or anal cancer (nos)** | 2774 | 504 | 767 | 854 | 838.99 | 921 | 1184 | 116.34 |
| **Unspecified primary cancer (nos)** | 1542 | 502 | 785.25 | 858 | 845.88 | 927 | 1150 | 111.69 |

Min=minimum; Q1=1^st^ quartile; Q3=3^rd^ quartile; Max=maximum; SD=standard deviation.

**S15 Table. Summary statistics of sample level coverage (enhanced sensitivity) for clinical samples (QC=Qualified).**

| **Tumor Type** | **N of Samples** | **Min** | **Q1** | **Median** | **Mean** | **Q3** | **Max** | **SD** |
| --- | --- | --- | --- | --- | --- | --- | --- | --- |
| **Breast cancer (nos)** | 374 | 157 | 1610.50 | 1826 | 1957.97 | 1988.50 | 4249 | 709.71 |
| **Lung cancer (nos)** | 913 | 198 | 1608 | 1838 | 1952.57 | 1991 | 4742 | 705.89 |
| **Pancreatobiliary or liver cancer (nos)** | 218 | 8 | 1642.25 | 1920.50 | 2167.81 | 2687.25 | 4675 | 863.59 |
| **Prostate cancer (nos)** | 674 | 369 | 1596 | 1834.50 | 1974.25 | 1992.75 | 4785 | 699.19 |
| **Small intestinal, colorectal, or anal cancer (nos)** | 228 | 168 | 1692.75 | 1913.50 | 2112.92 | 2592.25 | 4570 | 836.91 |
| **Unspecified primary cancer (nos)** | 143 | 111 | 1673.50 | 1873 | 1941.93 | 1995 | 4593 | 702.53 |

Min=minimum; Q1=1^st^ quartile; Q3=3^rd^ quartile; Max=maximum; SD=standard deviation.

**S16 Table. Summary statistics of sample level coverage (standard sensitivity) for clinical samples (QC=Qualified).**

| **Tumor Type** | **N of Samples** | **Min** | **Q1** | **Median** | **Mean** | **Q3** | **Max** | **SD** |
| --- | --- | --- | --- | --- | --- | --- | --- | --- |
| **Breast cancer (nos)** | 374 | 54 | 562 | 707.50 | 660.37 | 777 | 1036 | 168.86 |
| **Lung cancer (nos)** | 913 | 21 | 548 | 679 | 644.11 | 756 | 1110 | 173.25 |
| **Pancreatobiliary or liver cancer (nos)** | 218 | 3 | 485.50 | 672 | 620.18 | 748.75 | 1042 | 190.63 |
| **Prostate cancer (nos)** | 674 | 54 | 533 | 663 | 631.64 | 735.75 | 1040 | 145.69 |
| **Small intestinal, colorectal, or anal cancer (nos)** | 228 | 35 | 469.75 | 663 | 612.61 | 744.25 | 1012 | 195.38 |
| **Unspecified primary cancer (nos)** | 143 | 72 | 490.50 | 677 | 627.36 | 757 | 1036 | 188.52 |

Min=minimum; Q1=1^st^ quartile; Q3=3^rd^ quartile; Max=maximum; SD=standard deviation.

**S17 Table. Summary statistics of target level coverage of all target regions of all genes for clinical samples (QC=Pass).**

**[S17 Table](https://foundationmedicine.sharepoint.com/:x:/s/R&DThinkTankforPDpapersandRAstrategy/Eaa-fTCtjSZHjuaY6BQsUZwBJ0Y8RrDfj0LjHtEbeoD4lQ?e=RmrcoA)**

**S18 Table. Summary statistics of target level coverage of all target regions of all genes for clinical samples (QC=Qualified).**

**[S18 Table](https://foundationmedicine.sharepoint.com/:x:/s/R&DThinkTankforPDpapersandRAstrategy/EQf5zO6g9tRIgB4m6kNFlYEB9_2PU7gvr7ubic-qVLyyTg?e=9LpKYp)**
