# Supplementary material for: Analytical validation and sequencing coverage studies suggest that performance of a liquid biopsy assay is tumor agnostic (DNA-is-DNA)
Source: PLoS One. 2025 Aug 1;20(8):e0329392. doi: 10.1371/journal.pone.0329392 (PMC12316276; doi:10.1371/journal.pone.0329392)

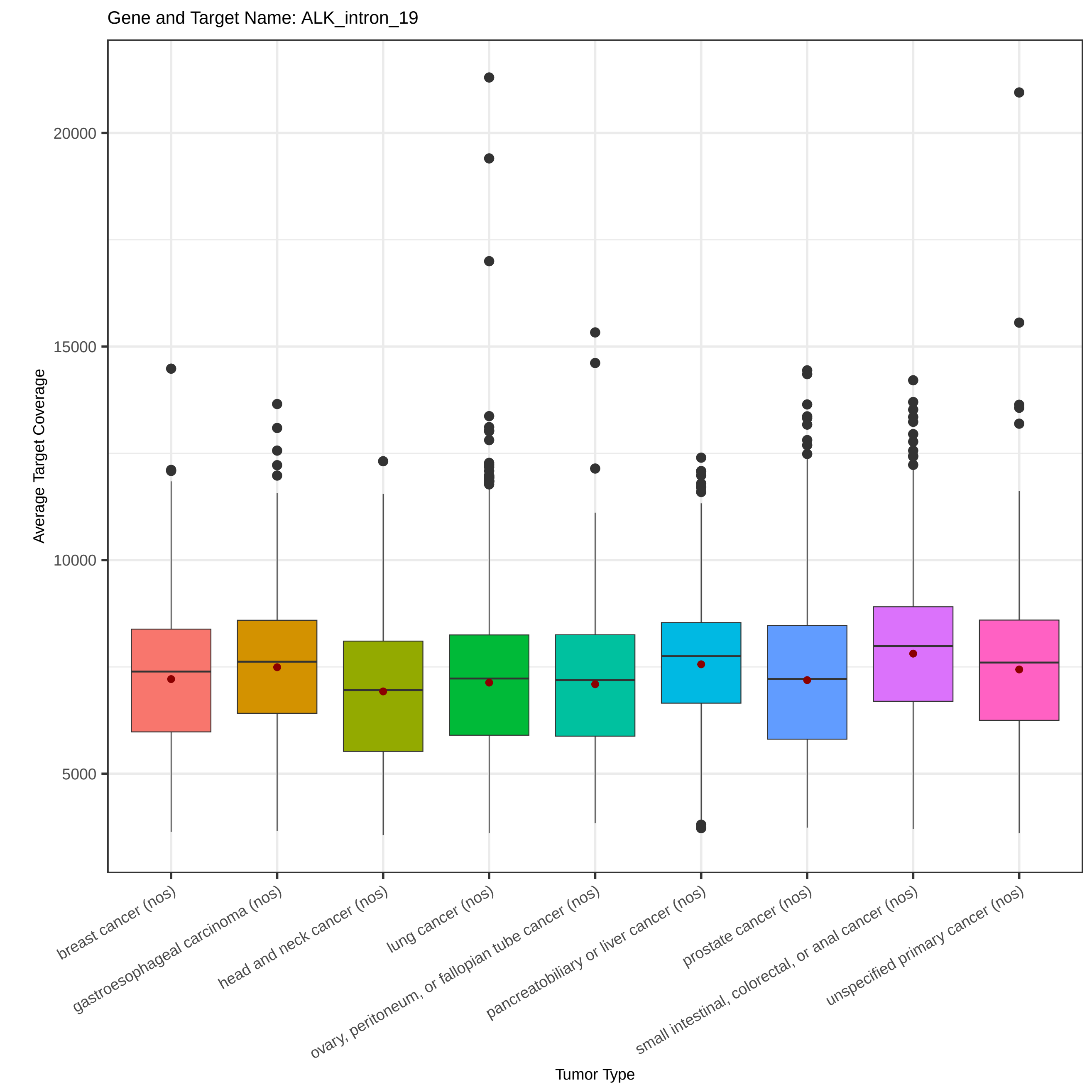

Gene and Target Name: ALK_intron_19
Average Target Coverage
20000
15000
10000
5000
breast cancer (nos)
gastroesophageal carcinoma (nos)
head and neck cancer (nos)
lung cancer (nos)
ovary, peritoneum, or fallopian tube cancer (nos)
pancreatobiliary or liver cancer (nos)
prostate cancer (nos)
small intestinal, colorectal, or anal cancer (nos)
unspecified primary cancer (nos)
Tumor Type

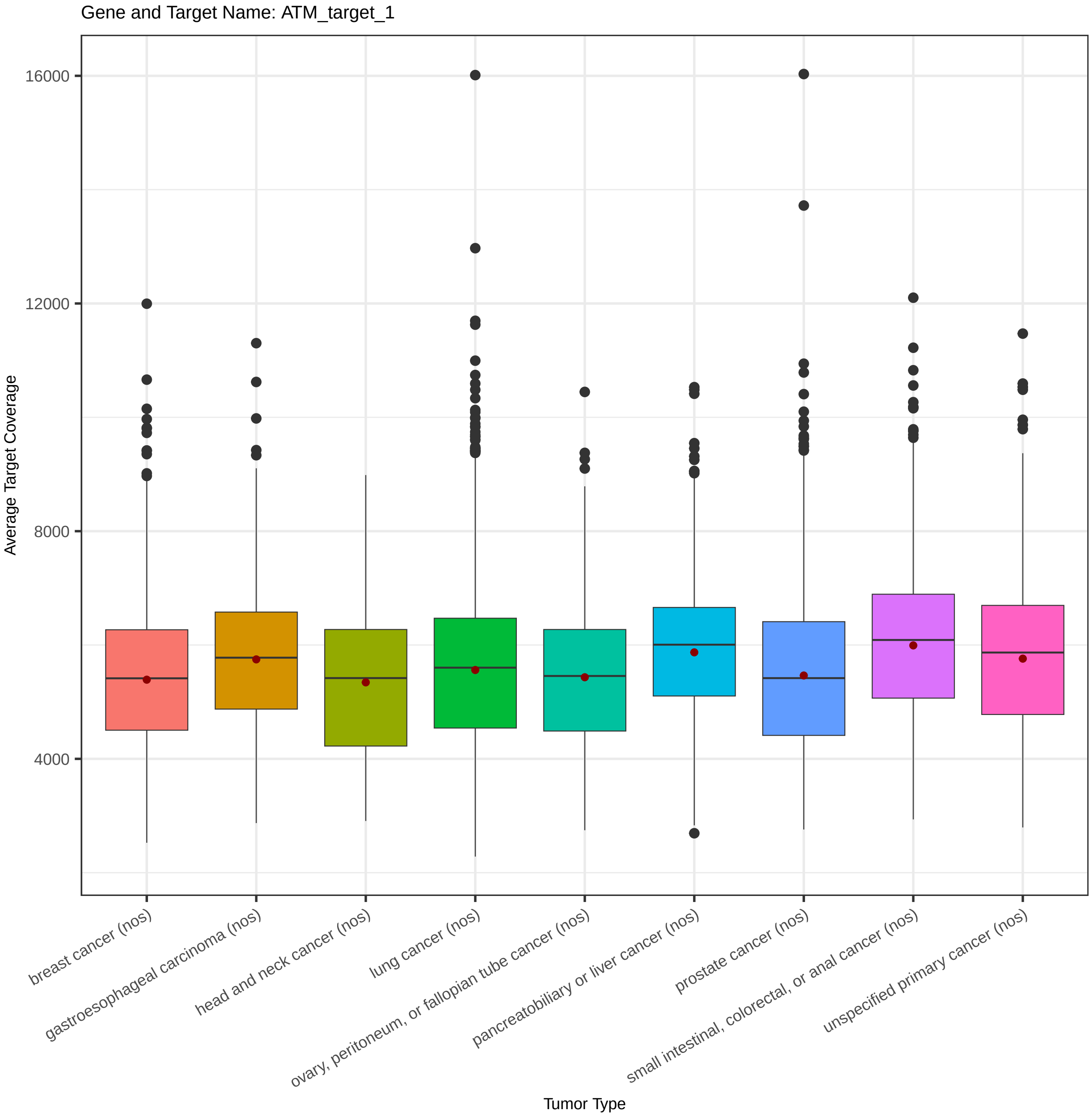

Gene and Target Name: ATM_target_1
Average Target Coverage
16000
12000
8000
4000
breast cancer (nos)
gastroesophageal carcinoma (nos)
head and neck cancer (nos)
lung cancer (nos)
ovary, peritoneum, or fallopian tube cancer (nos)
pancreatobiliary or liver cancer (nos)
prostate cancer (nos)
small intestinal, colorectal, or anal cancer (nos)
unspecified primary cancer (nos)
Tumor Type

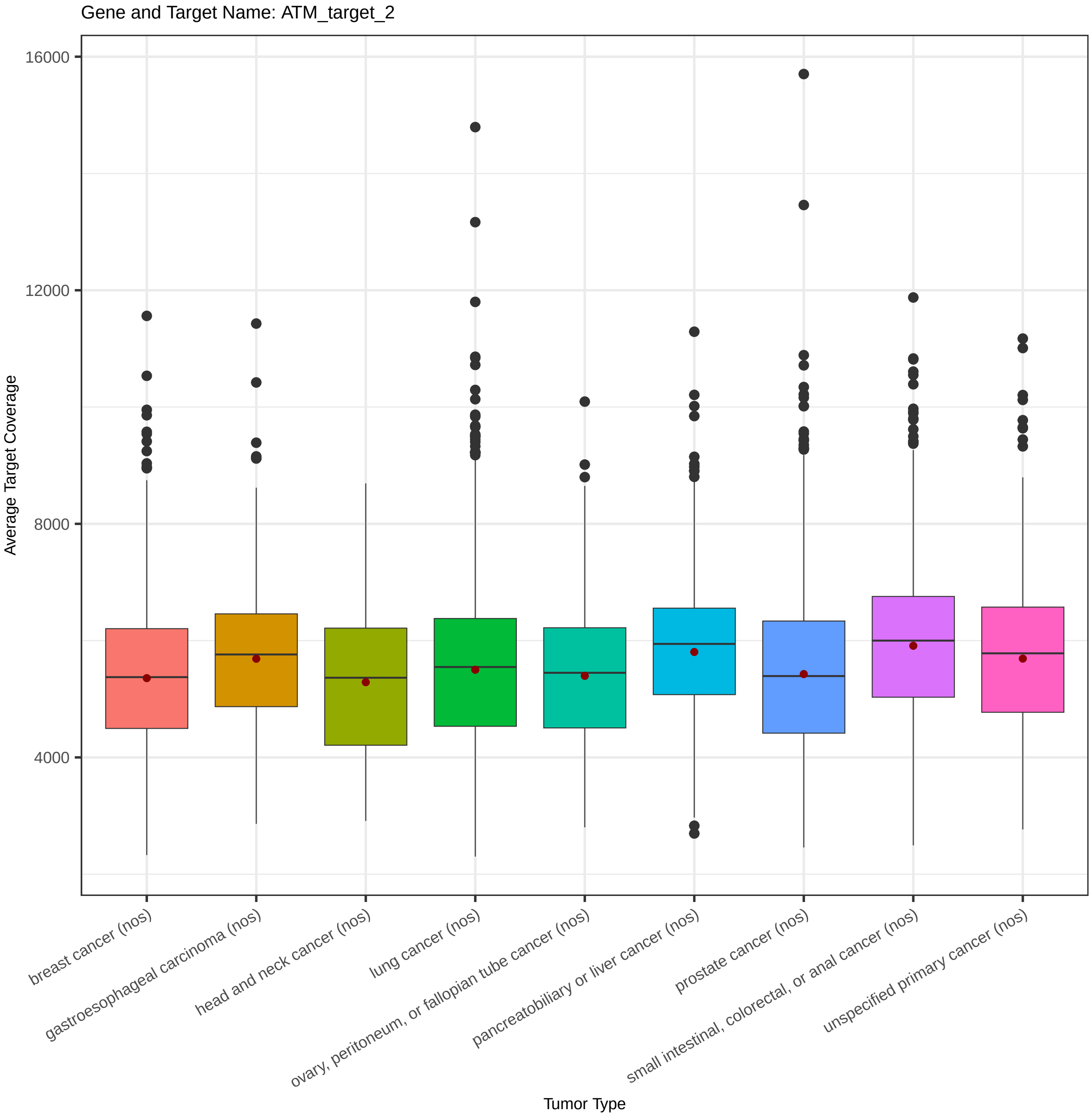

Gene and Target Name: ATM_target_2
Average Target Coverage
16000
12000
8000
4000
breast cancer (nos)
gastroesophageal carcinoma (nos)
head and neck cancer (nos)
lung cancer (nos)
ovary, peritoneum, or fallopian tube cancer (nos)
pancreatobiliary or liver cancer (nos)
prostate cancer (nos)
small intestinal, colorectal, or anal cancer (nos)
unspecified primary cancer (nos)
Tumor Type

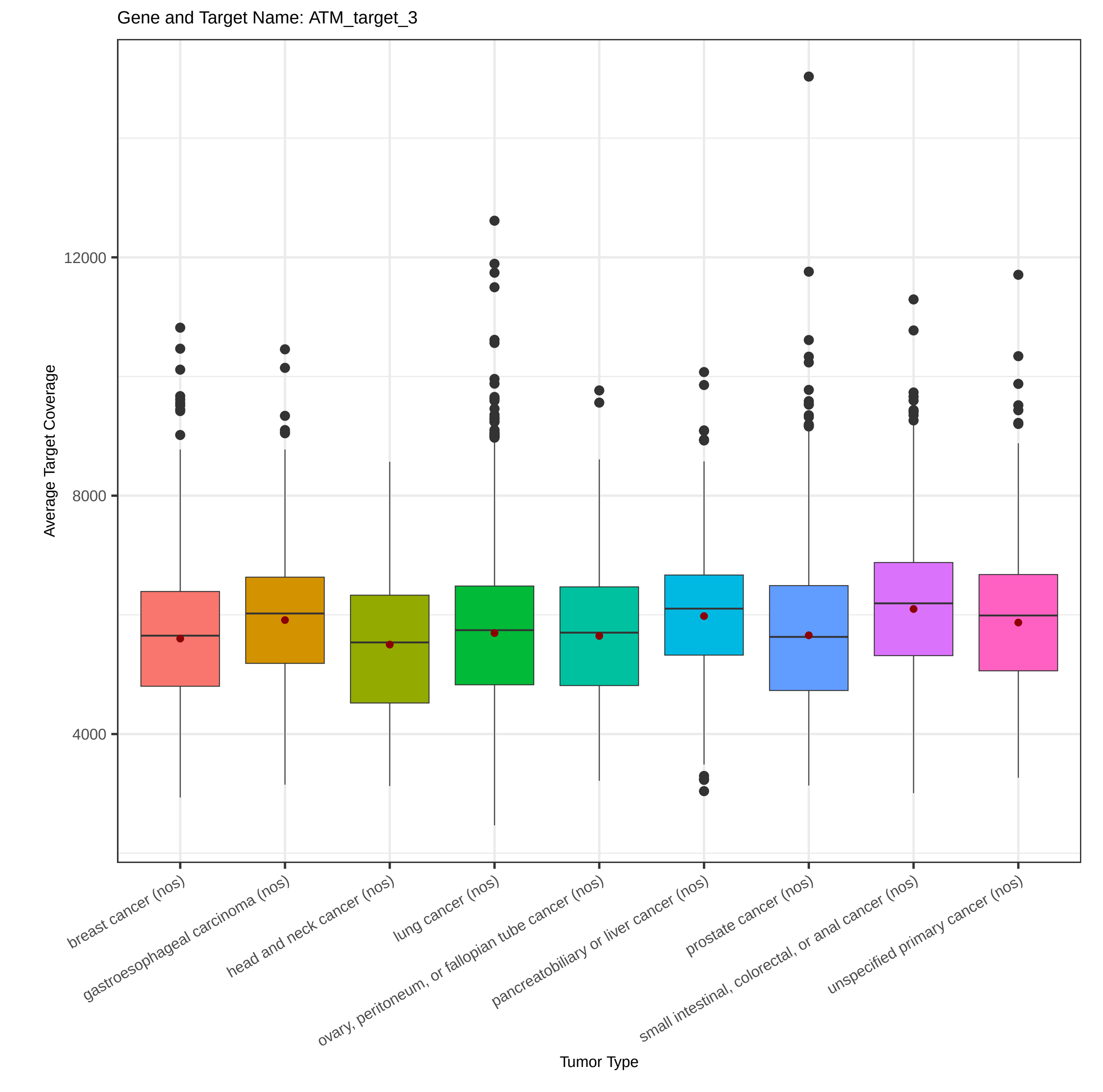

Gene and Target Name: ATM_target_3
Average Target Coverage
12000
8000
4000
Tumor Type
breast cancer (nos)
gastroesophageal carcinoma (nos)
head and neck cancer (nos)
lung cancer (nos)
ovary, peritoneum, or fallopian tube cancer (nos)
pancreatobiliary or liver cancer (nos)
prostate cancer (nos)
small intestinal, colorectal, or anal cancer (nos)
unspecified primary cancer (nos)

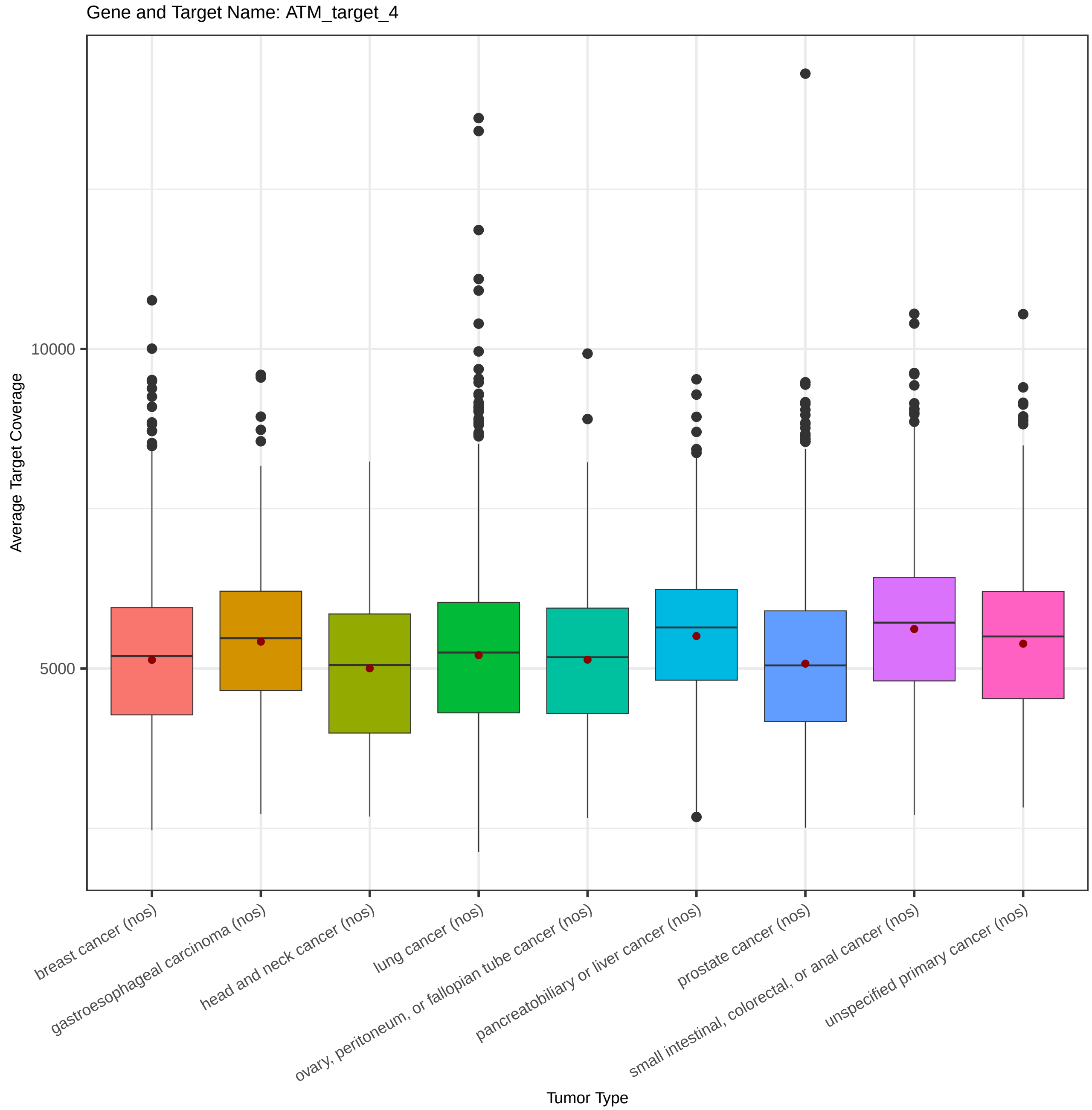

Gene and Target Name: ATM_target_4
Average Target Coverage
10000
5000
breast cancer (nos)
gastroesophageal carcinoma (nos)
head and neck cancer (nos)
lung cancer (nos)
ovary, peritoneum, or fallopian tube cancer (nos)
pancreatobiliary or liver cancer (nos)
prostate cancer (nos)
small intestinal, colorectal, or anal cancer (nos)
unspecified primary cancer (nos)
Tumor Type

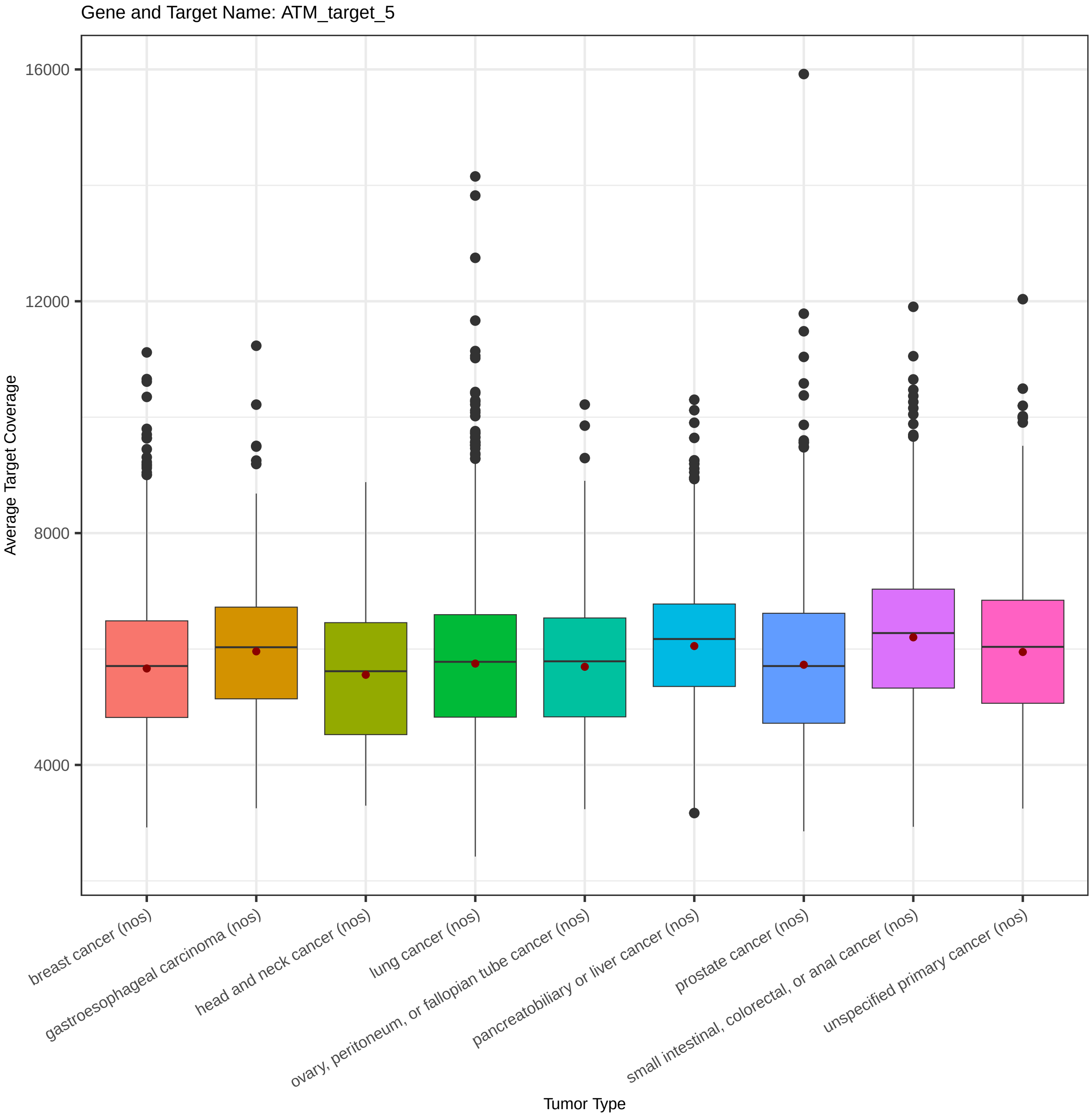
Gene and Target Name: ATM_target_5
Average Target Coverage
16000
12000
8000
4000
breast cancer (nos)
gastroesophageal carcinoma (nos)
head and neck cancer (nos)
lung cancer (nos)
ovary, peritoneum, or fallopian tube cancer (nos)
pancreatobiliary or liver cancer (nos)
prostate cancer (nos)
small intestinal, colorectal, or anal cancer (nos)
unspecified primary cancer (nos)
Tumor Type

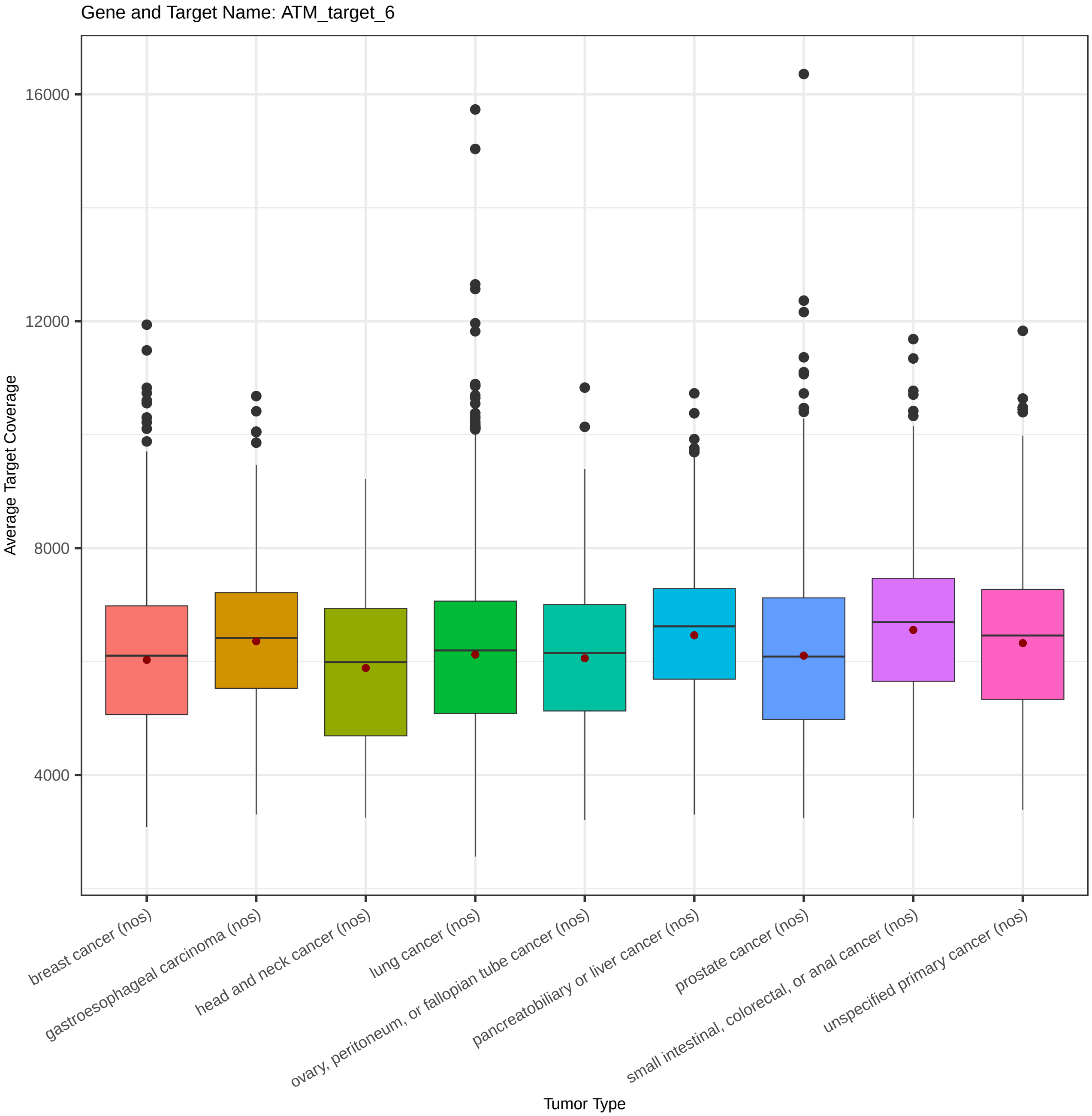

Gene and Target Name: ATM_target_6
Average Target Coverage
16000
12000
8000
4000
breast cancer (nos)
gastroesophageal carcinoma (nos)
head and neck cancer (nos)
lung cancer (nos)
ovary, peritoneum, or fallopian tube cancer (nos)
pancreatobiliary or liver cancer (nos)
prostate cancer (nos)
small intestinal, colorectal, or anal cancer (nos)
unspecified primary cancer (nos)
Tumor Type

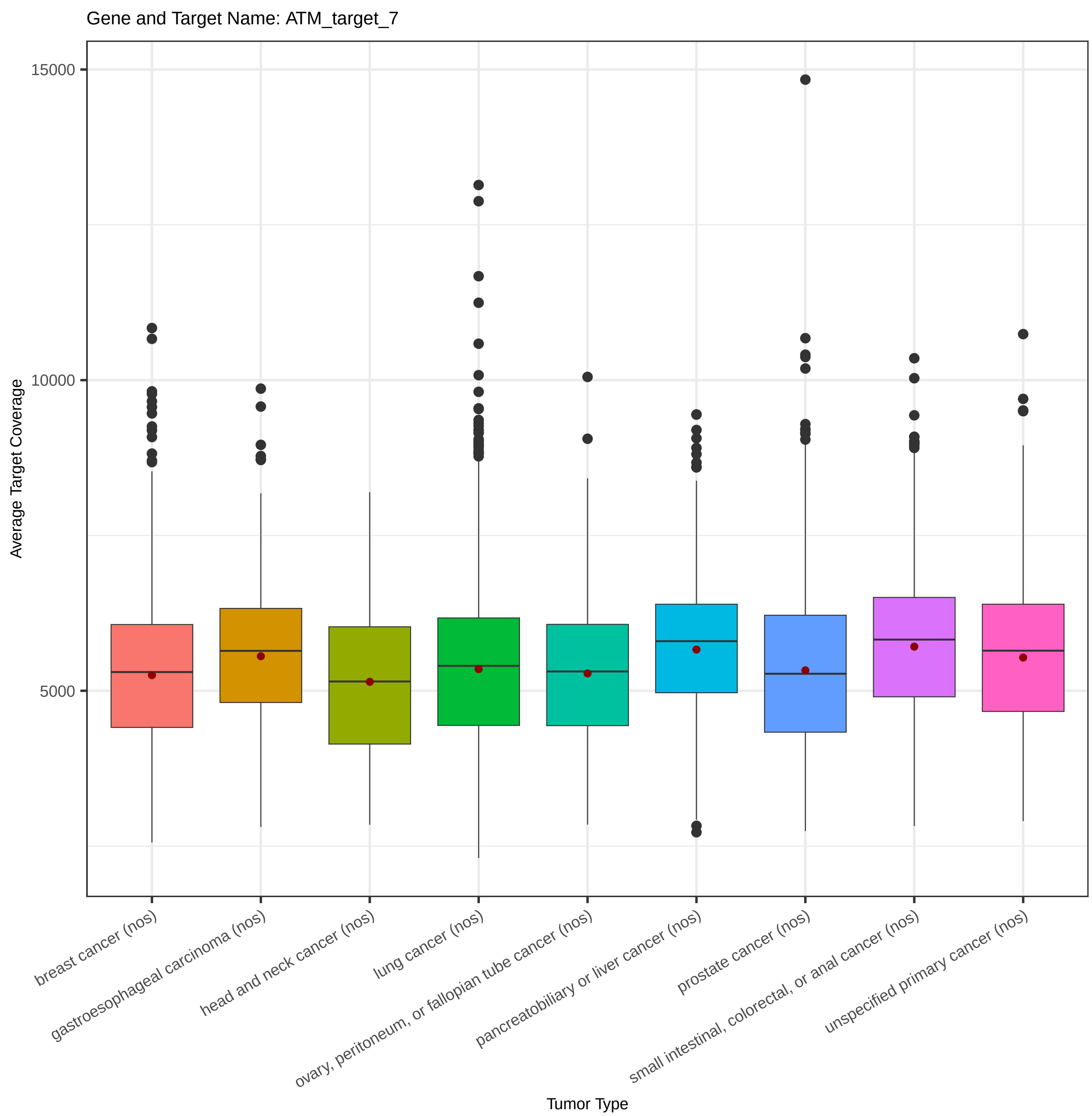
Gene and Target Name: ATM_target_7
Average Target Coverage
15000
10000
5000
breast cancer (nos)
gastroesophageal carcinoma (nos)
head and neck cancer (nos)
lung cancer (nos)
ovary, peritoneum, or fallopian tube cancer (nos)
pancreatobiliary or liver cancer (nos)
prostate cancer (nos)
small intestinal, colorectal, or anal cancer (nos)
unspecified primary cancer (nos)
Tumor Type

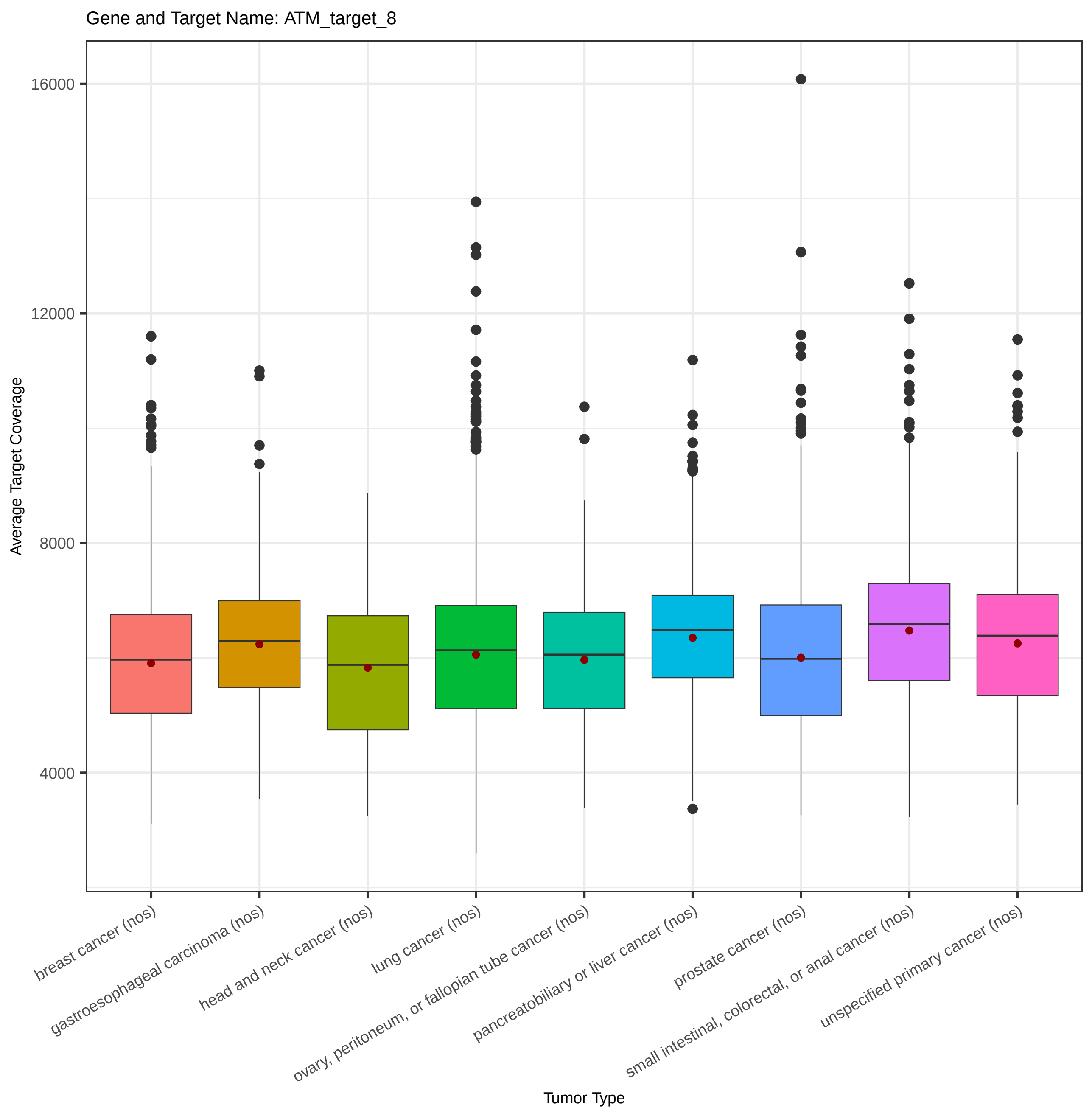

Gene and Target Name: ATM_target_8
Average Target Coverage
16000
12000
8000
4000
breast cancer (nos)
gastroesophageal carcinoma (nos)
head and neck cancer (nos)
lung cancer (nos)
ovary, peritoneum, or fallopian tube cancer (nos)
pancreatobiliary or liver cancer (nos)
prostate cancer (nos)
small intestinal, colorectal, or anal cancer (nos)
unspecified primary cancer (nos)
Tumor Type

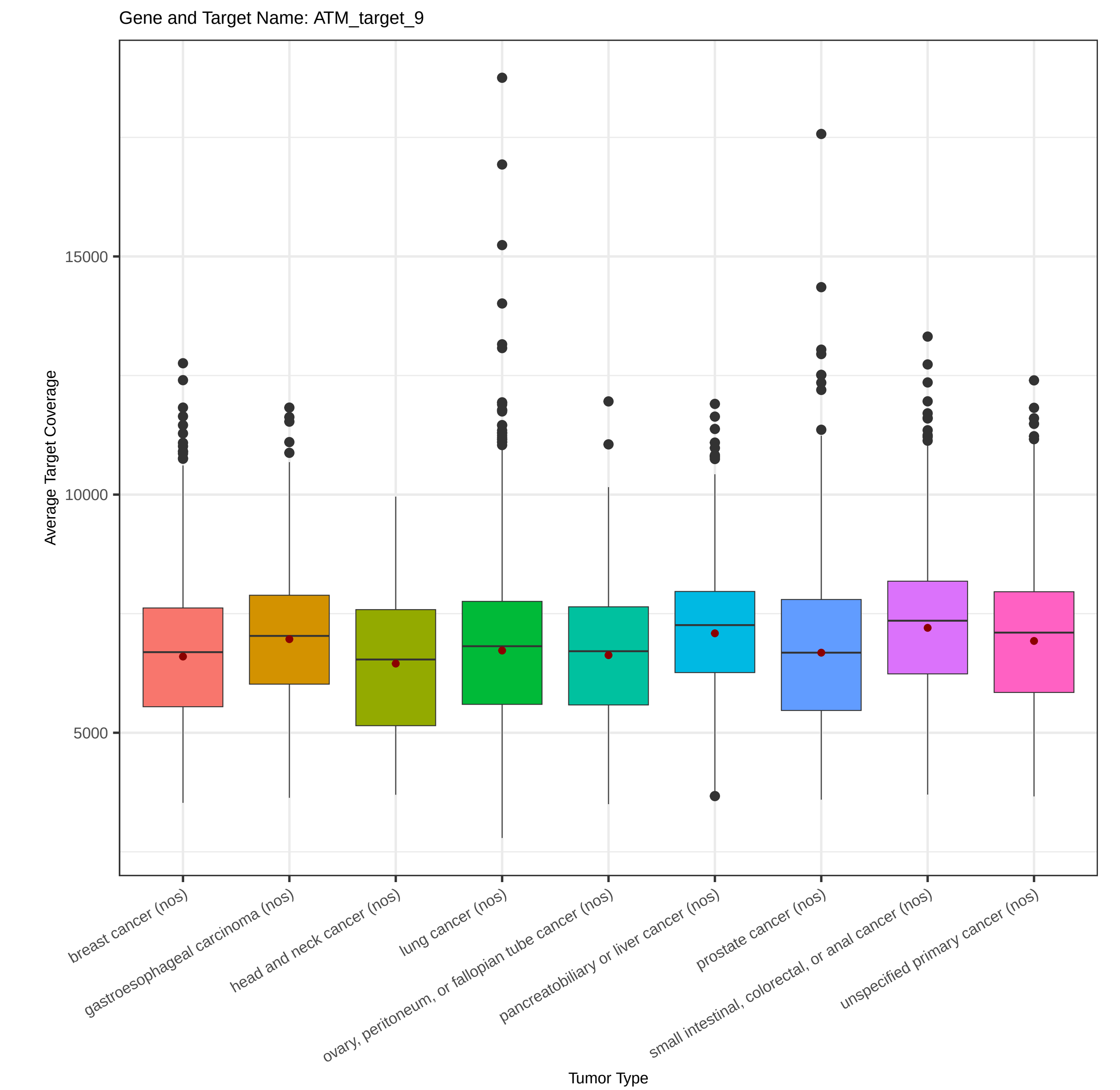
Gene and Target Name: ATM_target_9
Average Target Coverage
15000
10000
5000
Tumor Type
breast cancer (nos)
gastroesophageal carcinoma (nos)
head and neck cancer (nos)
lung cancer (nos)
ovary, peritoneum, or fallopian tube cancer (nos)
pancreatobiliary or liver cancer (nos)
prostate cancer (nos)
small intestinal, colorectal, or anal cancer (nos)
unspecified primary cancer (nos)

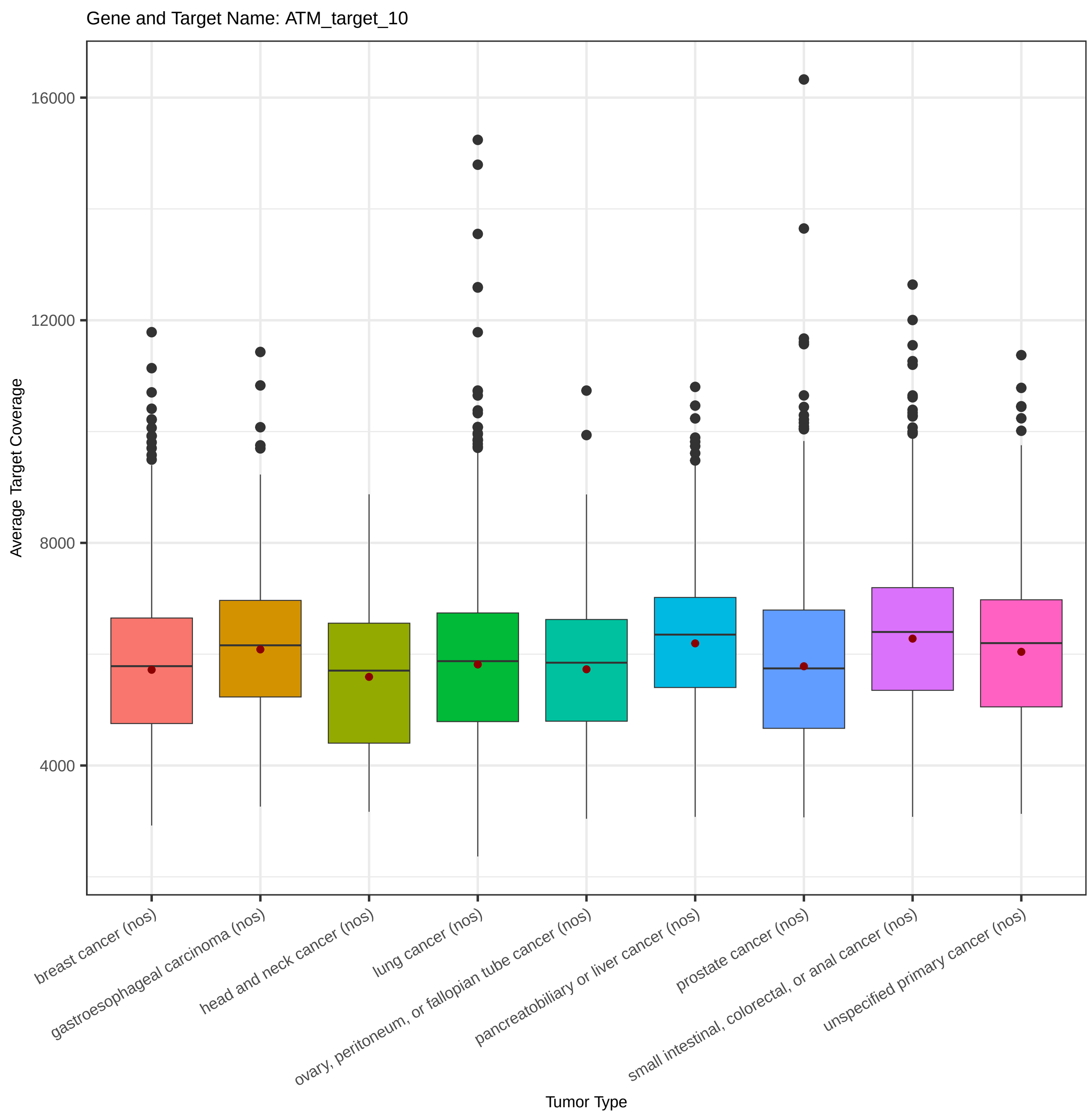

Gene and Target Name: ATM_target_10
Average Target Coverage
16000
12000
8000
4000
breast cancer (nos)
gastroesophageal carcinoma (nos)
head and neck cancer (nos)
lung cancer (nos)
ovary, peritoneum, or fallopian tube cancer (nos)
pancreatobiliary or liver cancer (nos)
prostate cancer (nos)
small intestinal, colorectal, or anal cancer (nos)
unspecified primary cancer (nos)
Tumor Type

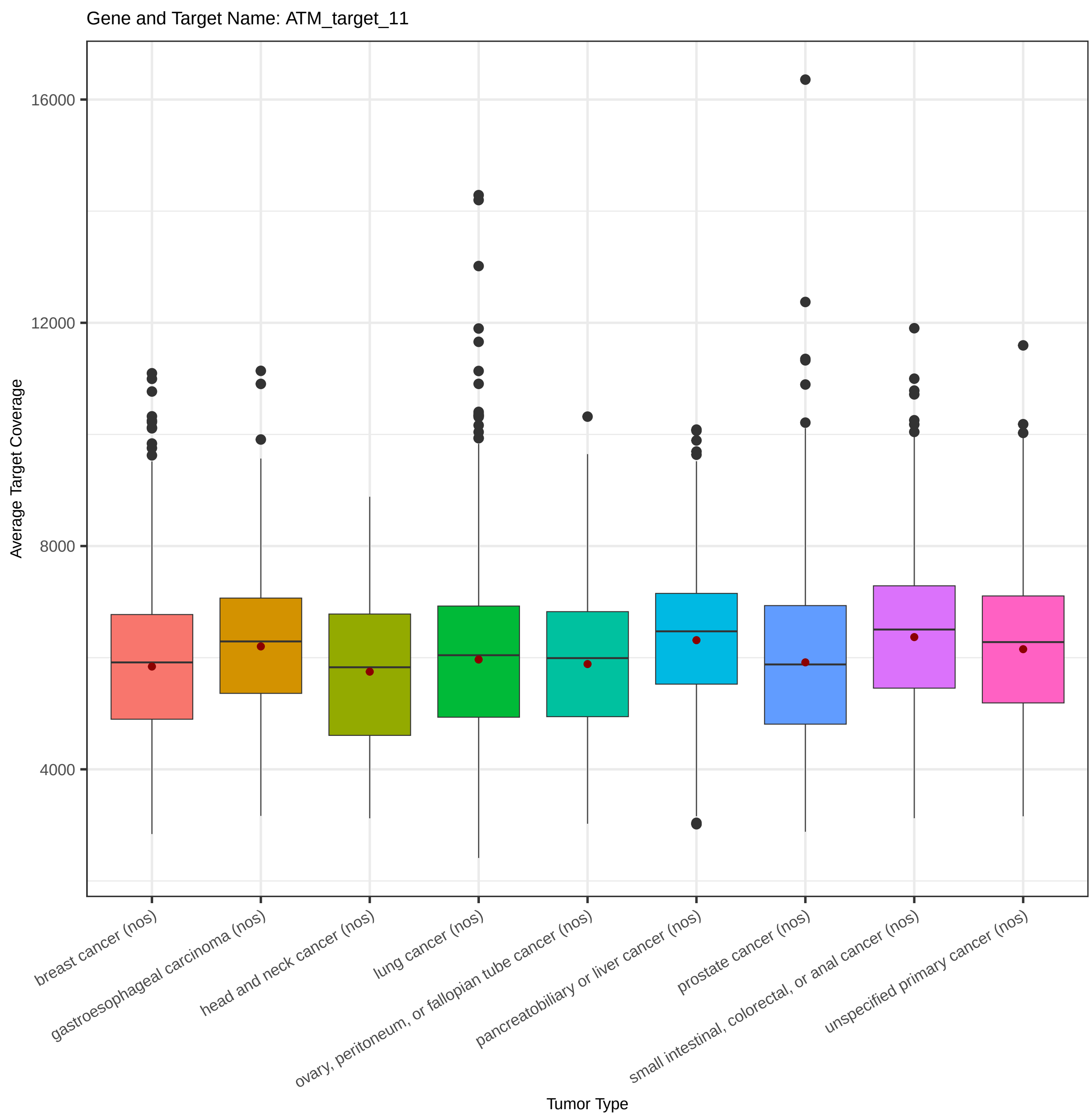
Gene and Target Name: ATM_target_11
Average Target Coverage
16000
12000
8000
4000
breast cancer (nos)
gastroesophageal carcinoma (nos)
head and neck cancer (nos)
lung cancer (nos)
ovary, peritoneum, or fallopian tube cancer (nos)
pancreatobiliary or liver cancer (nos)
prostate cancer (nos)
small intestinal, colorectal, or anal cancer (nos)
unspecified primary cancer (nos)
Tumor Type

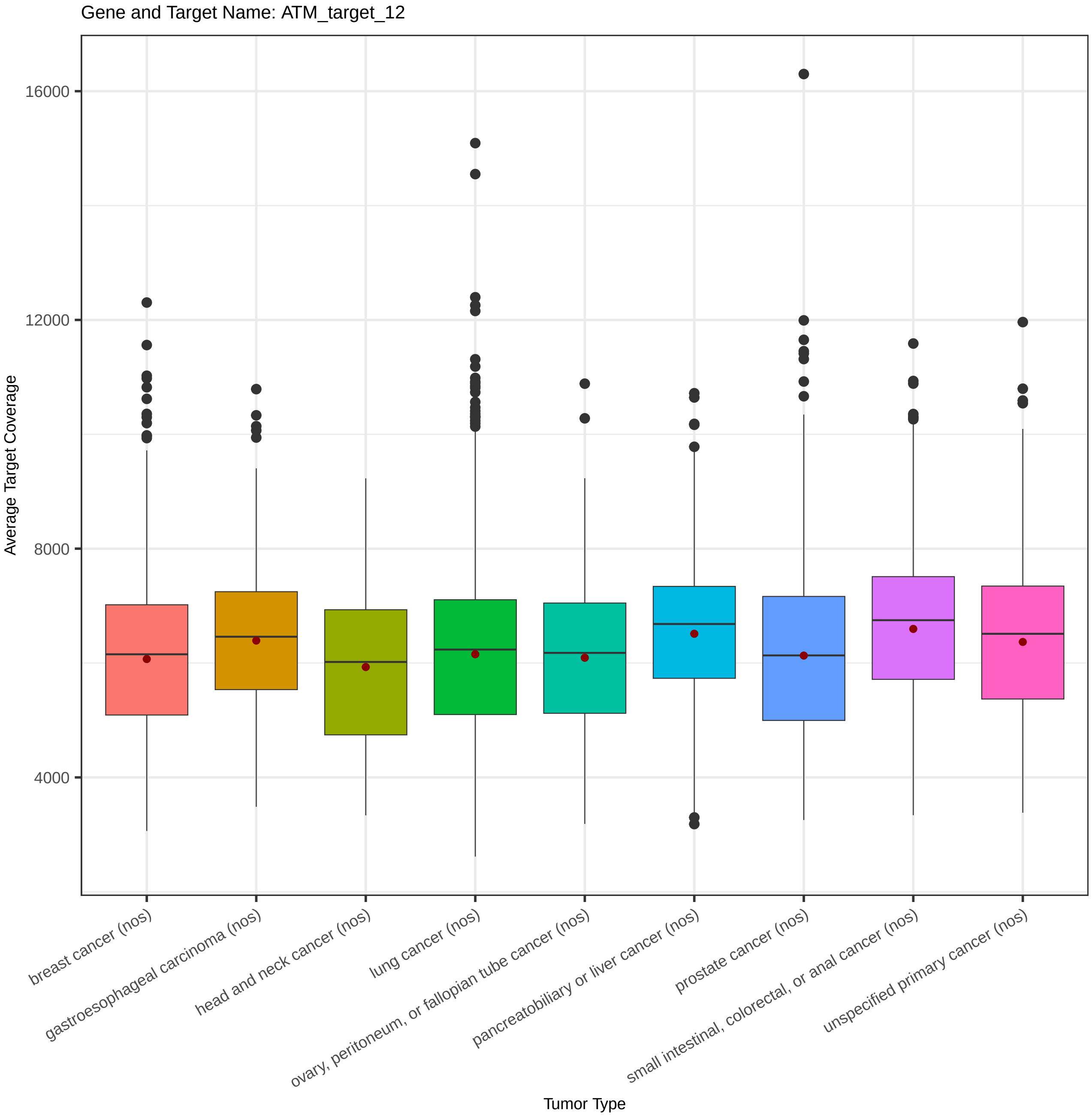
Gene and Target Name: ATM_target_12
Average Target Coverage
16000
12000
8000
4000
breast cancer (nos)
gastroesophageal carcinoma (nos)
head and neck cancer (nos)
lung cancer (nos)
ovary, peritoneum, or fallopian tube cancer (nos)
pancreatobiliary or liver cancer (nos)
prostate cancer (nos)
small intestinal, colorectal, or anal cancer (nos)
unspecified primary cancer (nos)
Tumor Type

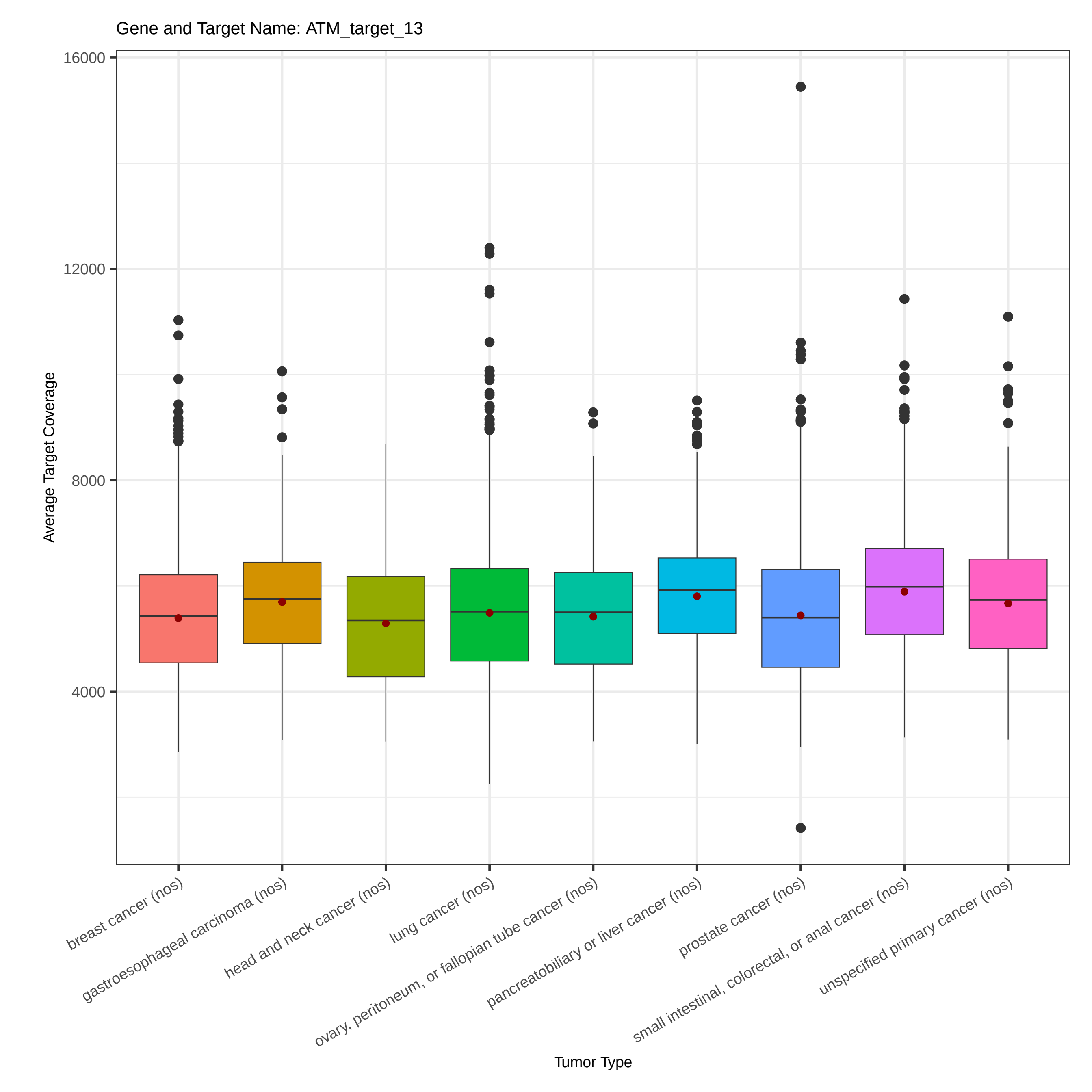
Gene and Target Name: ATM_target_13
Average Target Coverage
16000
12000
8000
4000
breast cancer (nos)
gastroesophageal carcinoma (nos)
head and neck cancer (nos)
lung cancer (nos)
ovary, peritoneum, or fallopian tube cancer (nos)
pancreatobiliary or liver cancer (nos)
prostate cancer (nos)
small intestinal, colorectal, or anal cancer (nos)
unspecified primary cancer (nos)
Tumor Type

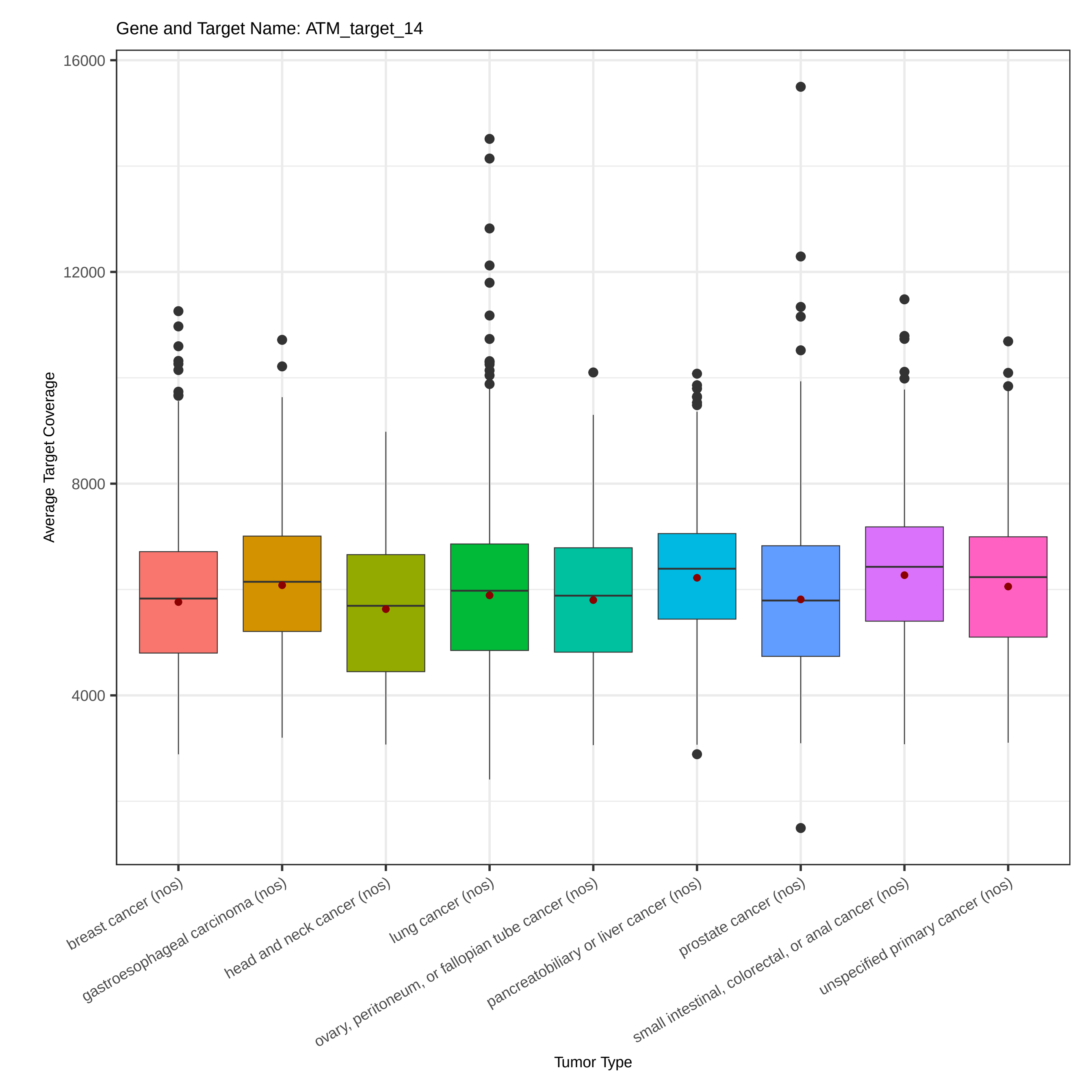
Gene and Target Name: ATM_target_14
Average Target Coverage
16000
12000
8000
4000
breast cancer (nos)
gastroesophageal carcinoma (nos)
head and neck cancer (nos)
lung cancer (nos)
ovary, peritoneum, or fallopian tube cancer (nos)
pancreatobiliary or liver cancer (nos)
prostate cancer (nos)
small intestinal, colorectal, or anal cancer (nos)
unspecified primary cancer (nos)
Tumor Type

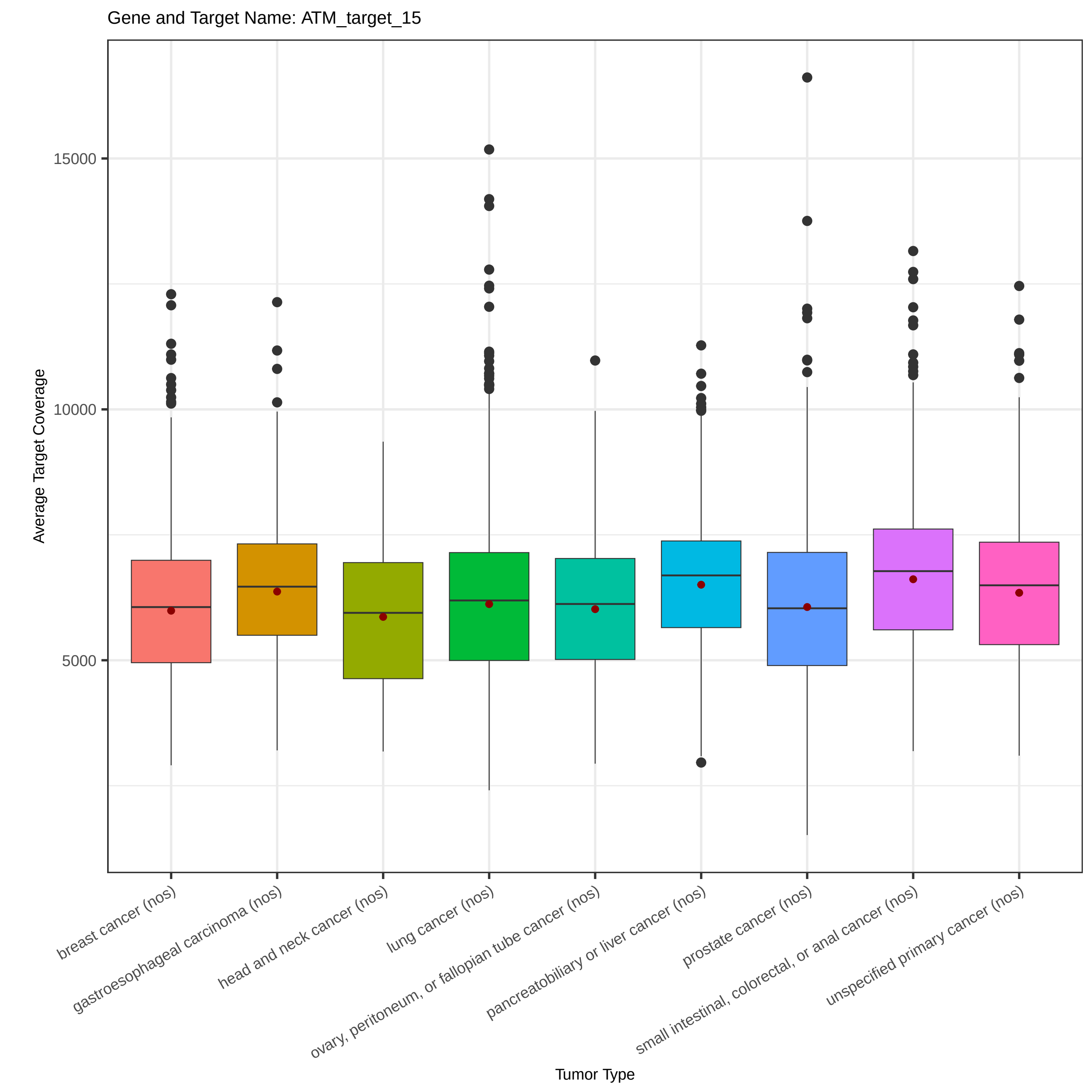
Gene and Target Name: ATM_target_15
Average Target Coverage
15000
10000
5000
breast cancer (nos)
gastroesophageal carcinoma (nos)
head and neck cancer (nos)
lung cancer (nos)
ovary, peritoneum, or fallopian tube cancer (nos)
pancreatobiliary or liver cancer (nos)
prostate cancer (nos)
small intestinal, colorectal, or anal cancer (nos)
unspecified primary cancer (nos)
Tumor Type

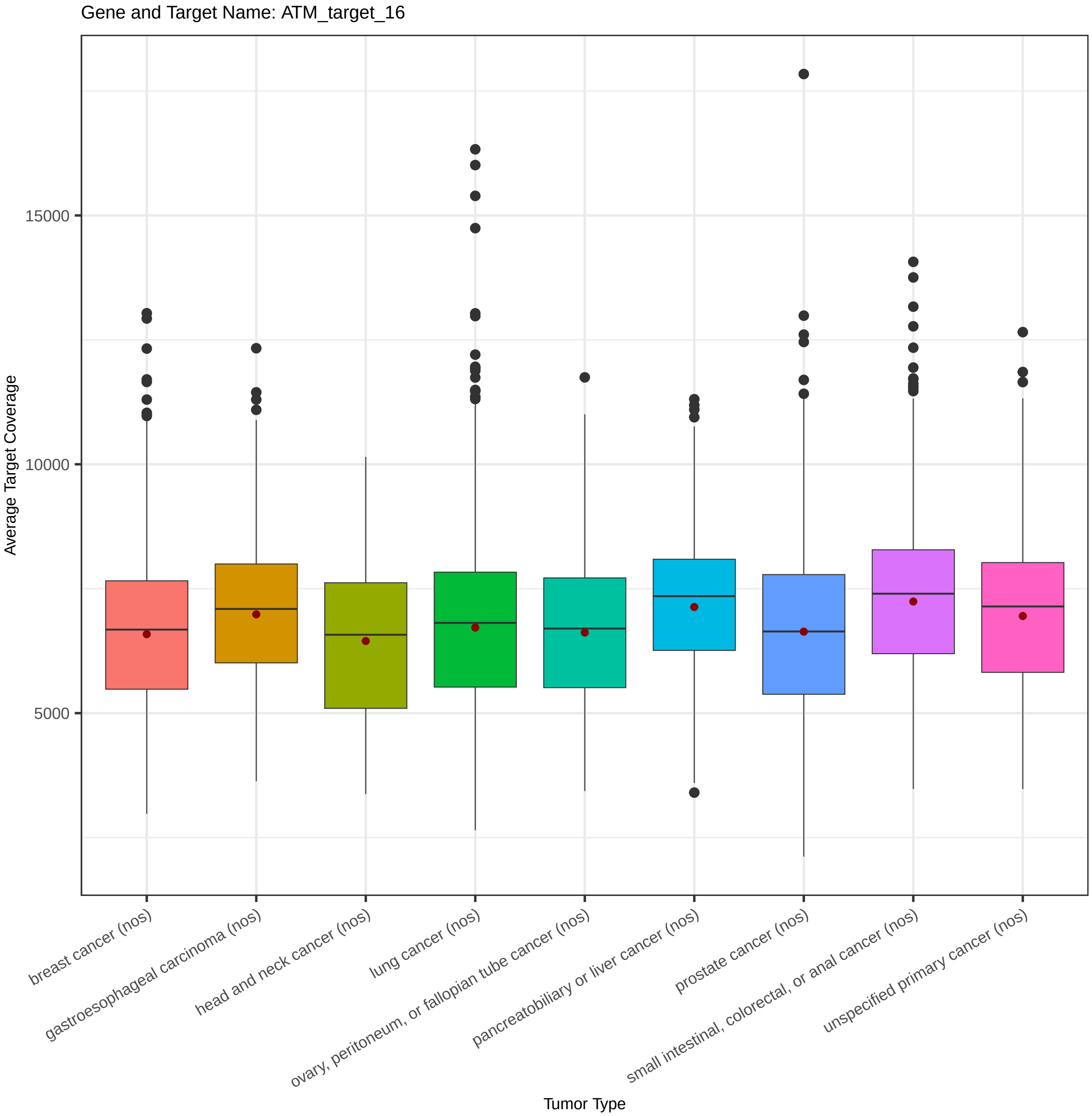

Gene and Target Name: ATM_target_16
Average Target Coverage
15000
10000
5000
breast cancer (nos)
gastroesophageal carcinoma (nos)
head and neck cancer (nos)
lung cancer (nos)
ovary, peritoneum, or fallopian tube cancer (nos)
pancreatobiliary or liver cancer (nos)
prostate cancer (nos)
small intestinal, colorectal, or anal cancer (nos)
unspecified primary cancer (nos)
Tumor Type

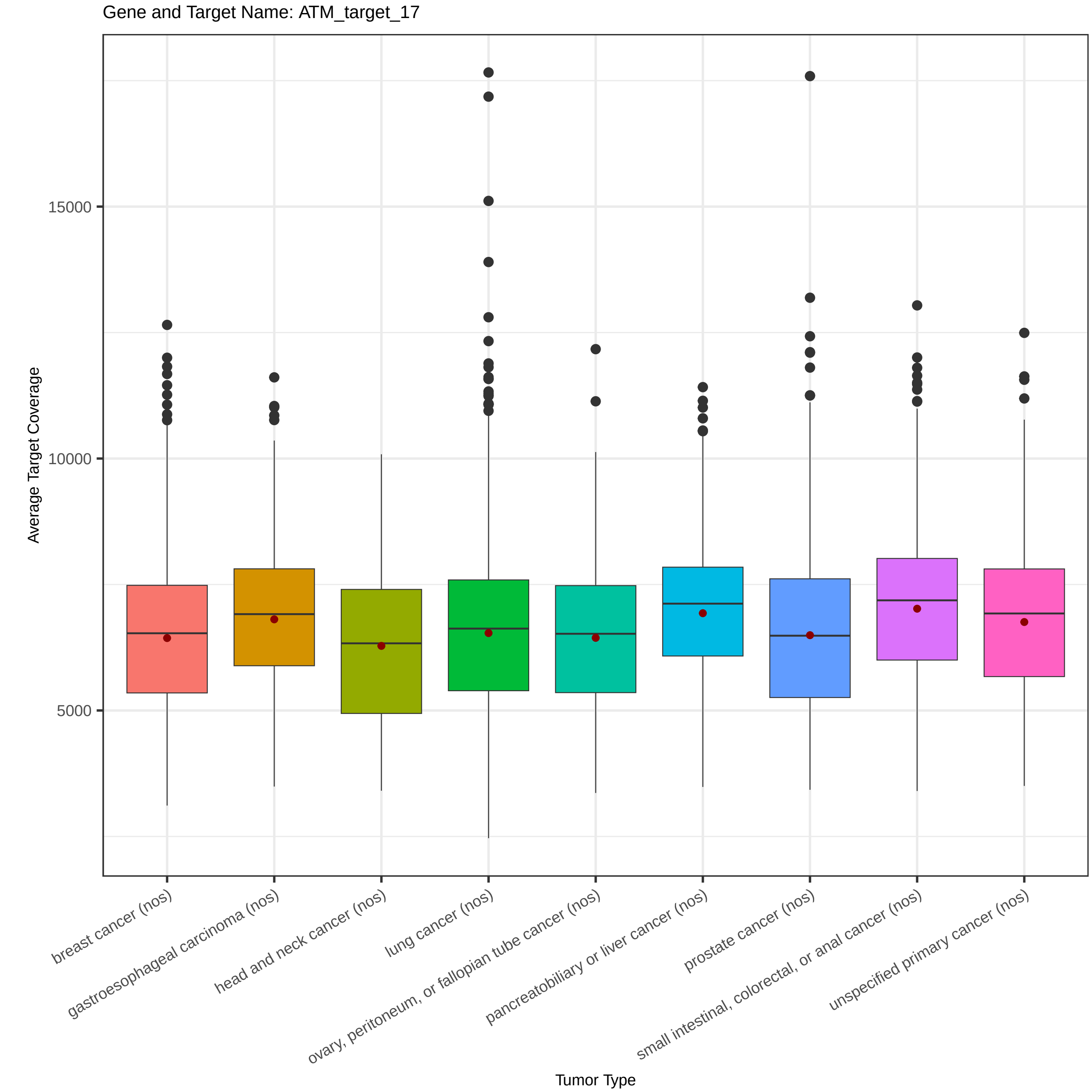

Gene and Target Name: ATM_target_17
Average Target Coverage
15000
10000
5000
Tumor Type
breast cancer (nos)
gastroesophageal carcinoma (nos)
head and neck cancer (nos)
lung cancer (nos)
ovary, peritoneum, or fallopian tube cancer (nos)
pancreatobiliary or liver cancer (nos)
prostate cancer (nos)
small intestinal, colorectal, or anal cancer (nos)
unspecified primary cancer (nos)

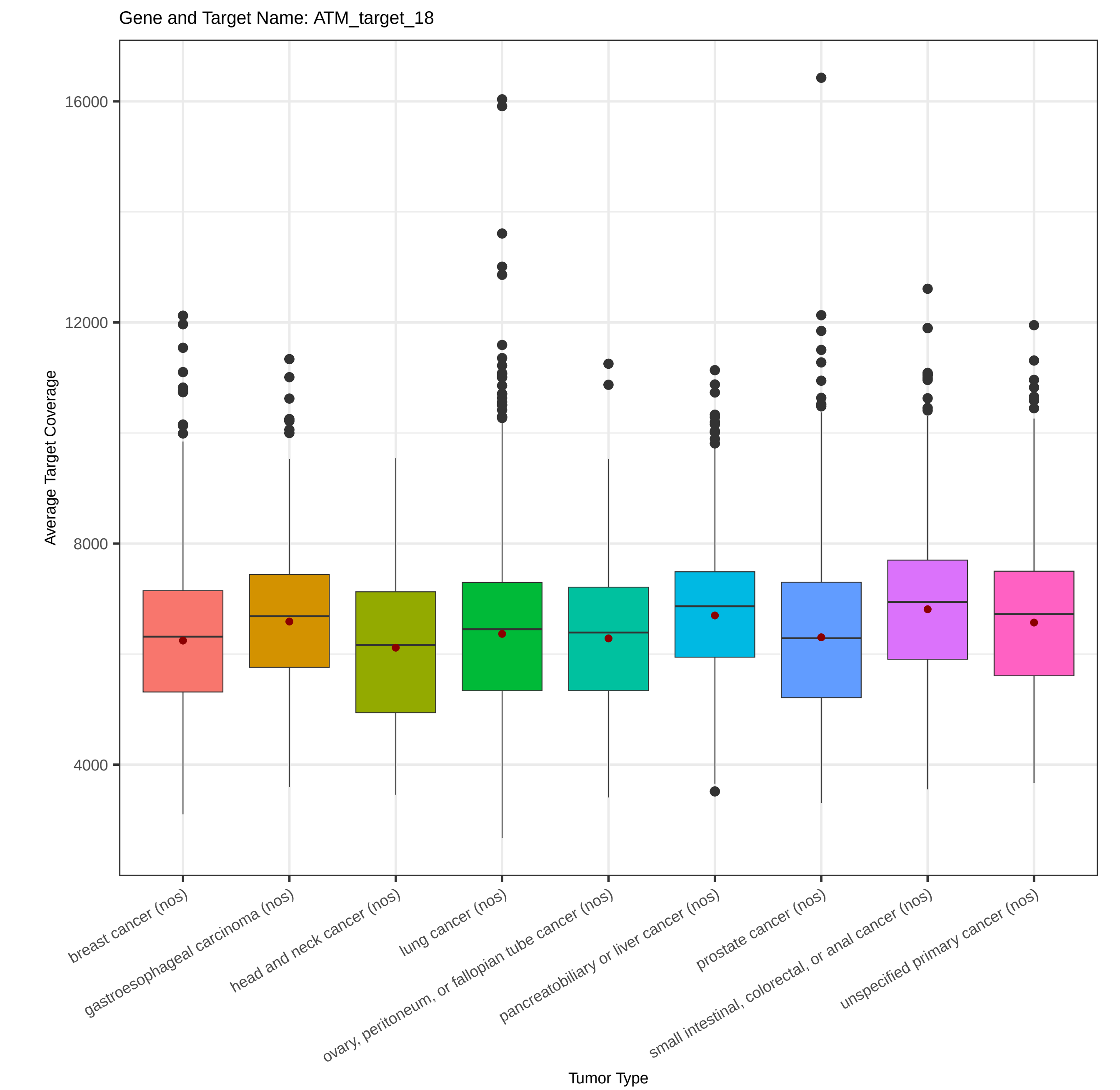
Gene and Target Name: ATM_target_18
Average Target Coverage
16000
12000
8000
4000
breast cancer (nos)
gastroesophageal carcinoma (nos)
head and neck cancer (nos)
lung cancer (nos)
ovary, peritoneum, or fallopian tube cancer (nos)
pancreatobiliary or liver cancer (nos)
prostate cancer (nos)
small intestinal, colorectal, or anal cancer (nos)
unspecified primary cancer (nos)
Tumor Type

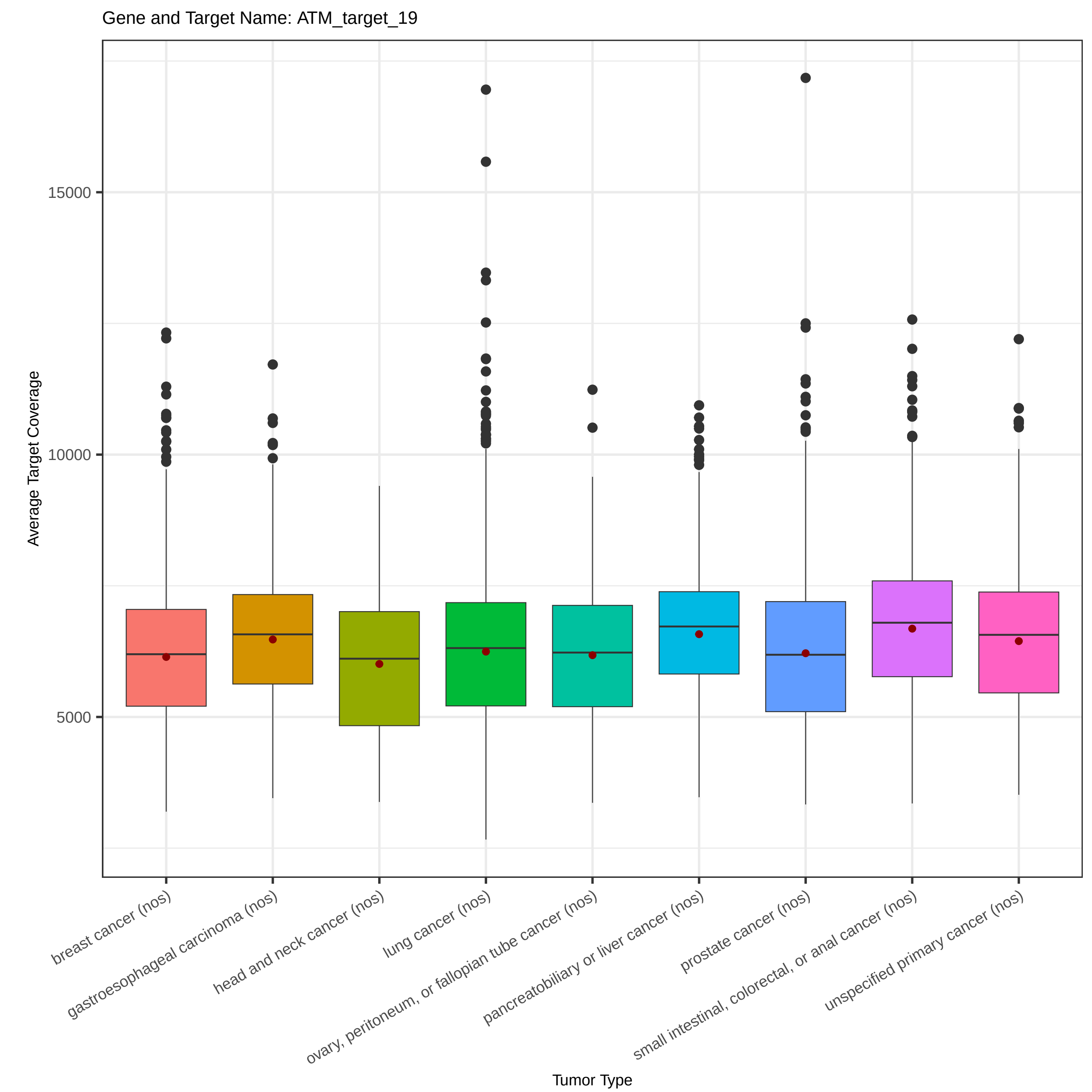

Gene and Target Name: ATM_target_19
Average Target Coverage
15000
10000
5000
breast cancer (nos)
gastroesophageal carcinoma (nos)
head and neck cancer (nos)
lung cancer (nos)
ovary, peritoneum, or fallopian tube cancer (nos)
pancreatobiliary or liver cancer (nos)
prostate cancer (nos)
small intestinal, colorectal, or anal cancer (nos)
unspecified primary cancer (nos)
Tumor Type

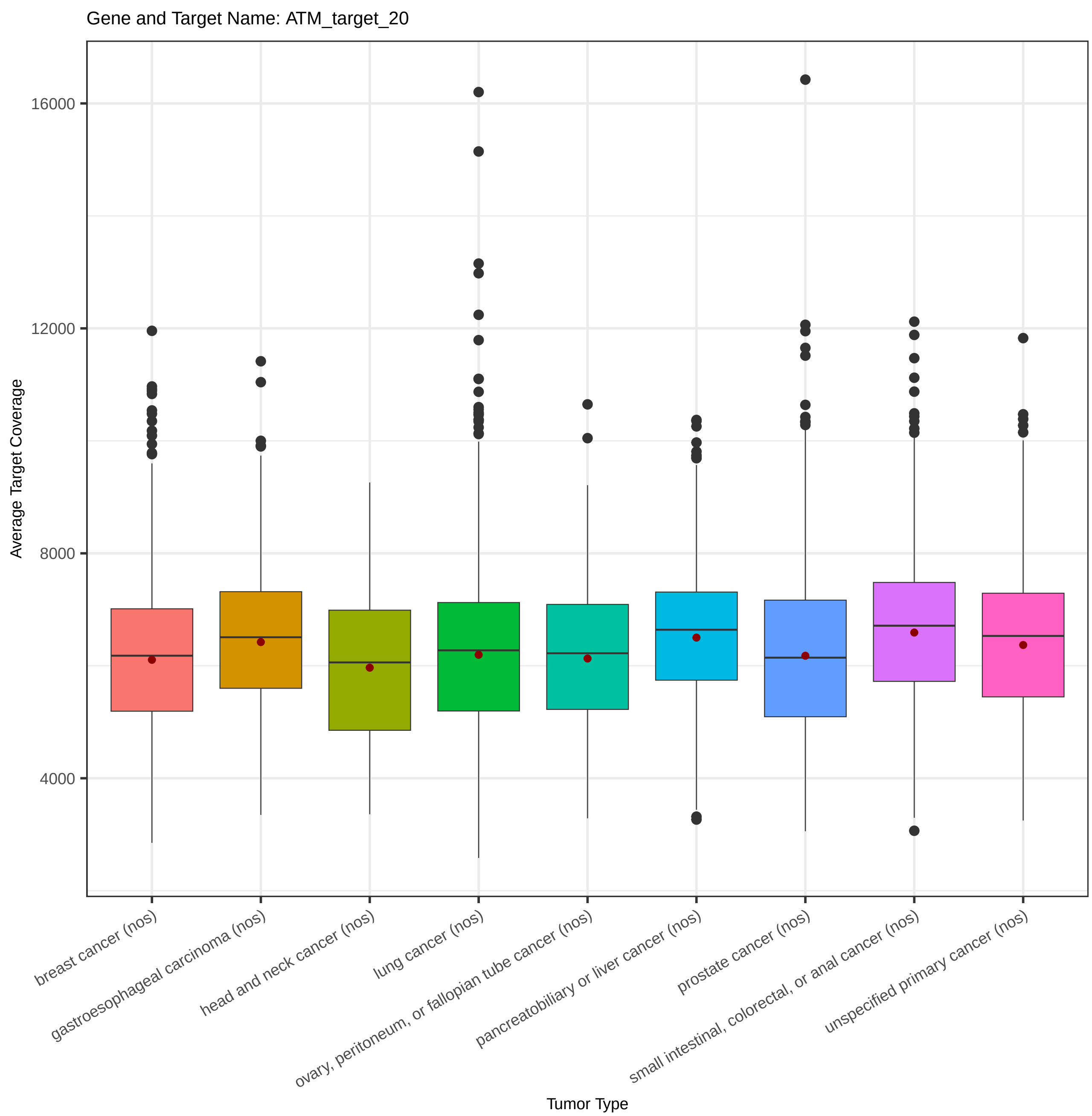

Gene and Target Name: ATM_target_20
Average Target Coverage
16000
12000
8000
4000
breast cancer (nos)
gastroesophageal carcinoma (nos)
head and neck cancer (nos)
lung cancer (nos)
ovary, peritoneum, or fallopian tube cancer (nos)
pancreatobiliary or liver cancer (nos)
prostate cancer (nos)
small intestinal, colorectal, or anal cancer (nos)
unspecified primary cancer (nos)
Tumor Type

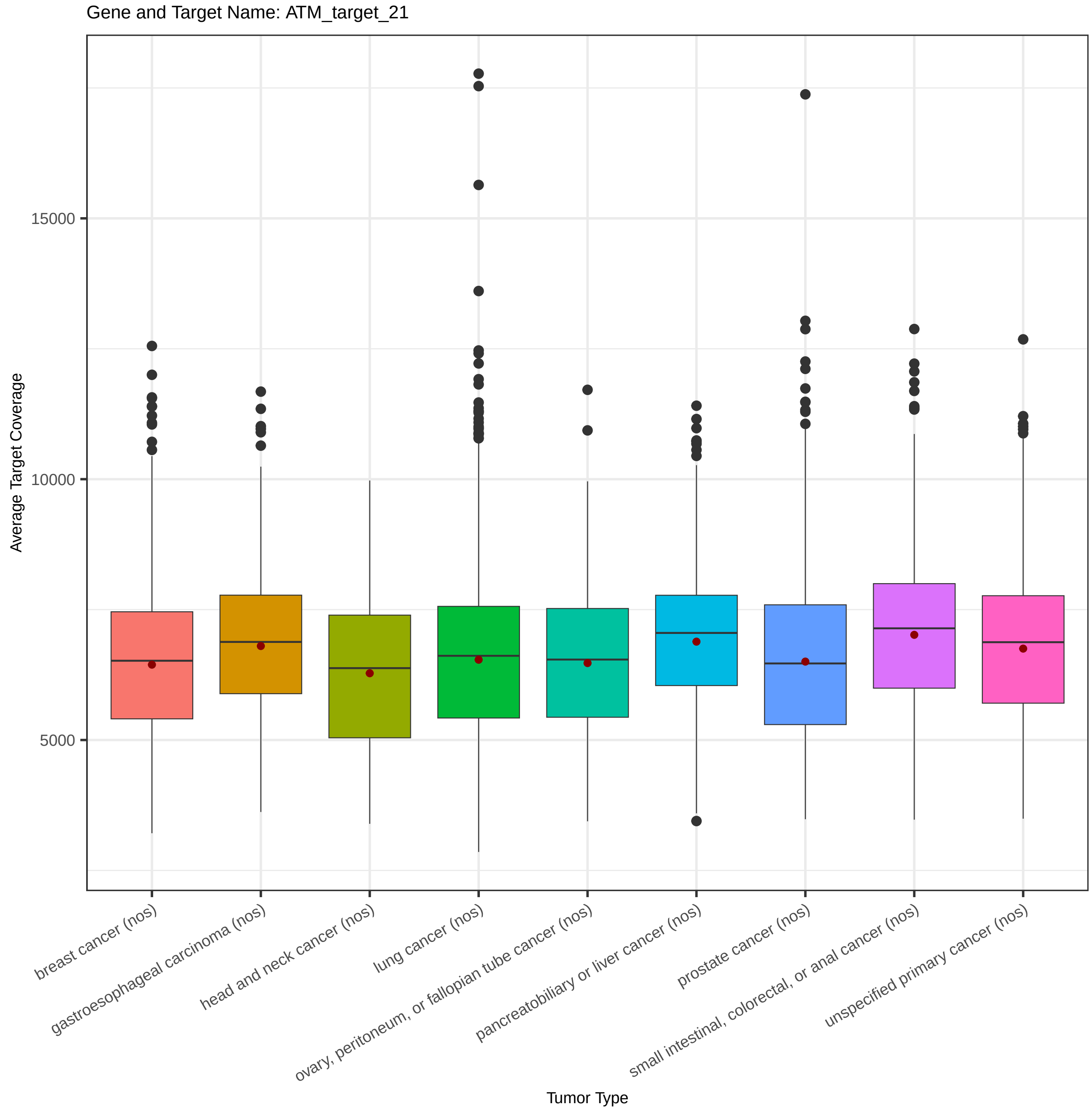

Gene and Target Name: ATM_target_21
Average Target Coverage
15000
10000
5000
breast cancer (nos)
gastroesophageal carcinoma (nos)
head and neck cancer (nos)
lung cancer (nos)
ovary, peritoneum, or fallopian tube cancer (nos)
pancreatobiliary or liver cancer (nos)
prostate cancer (nos)
small intestinal, colorectal, or anal cancer (nos)
unspecified primary cancer (nos)
Tumor Type

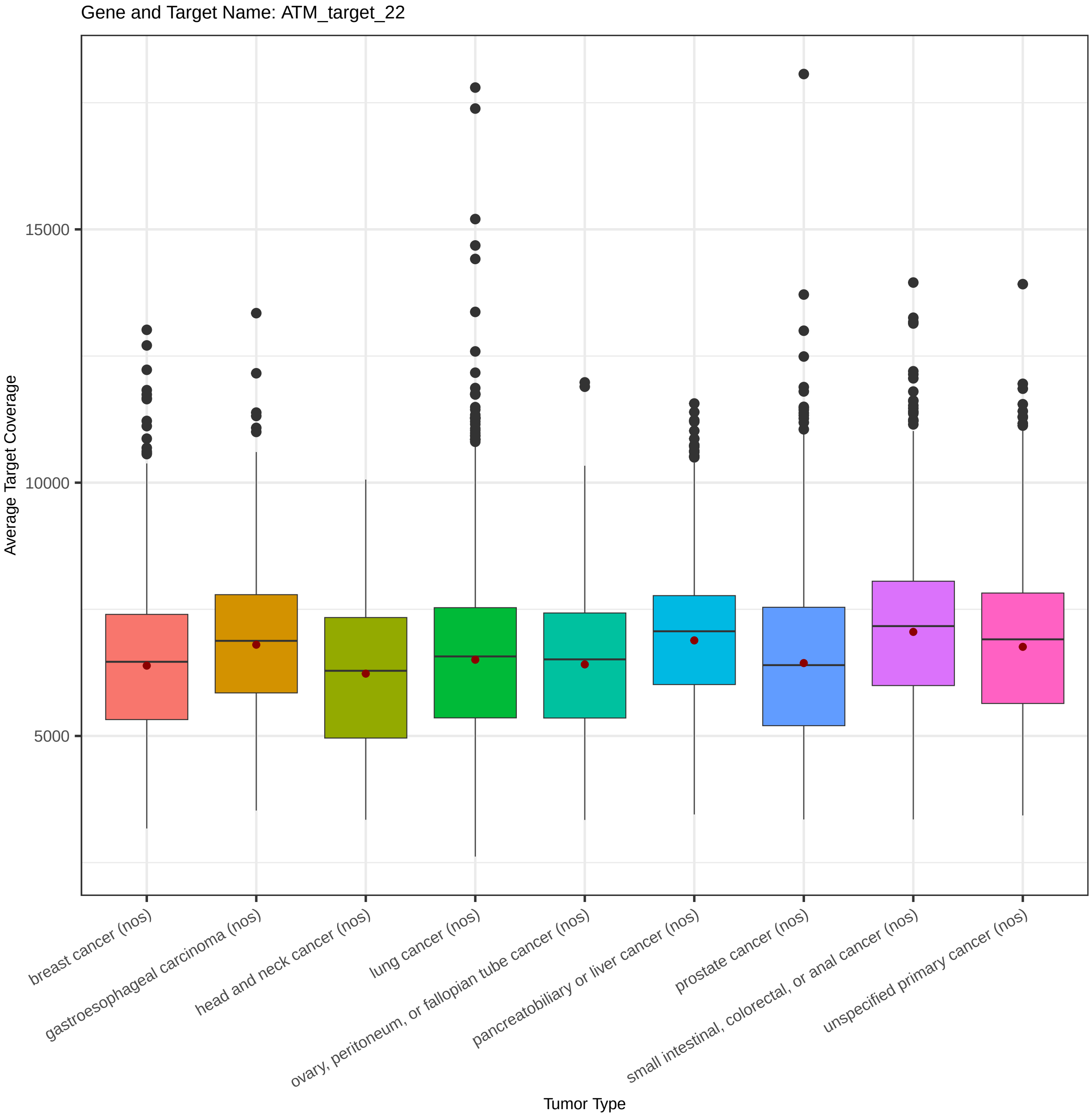

Gene and Target Name: ATM_target_22
Average Target Coverage
15000
10000
5000
Tumor Type
breast cancer (nos)
gastroesophageal carcinoma (nos)
head and neck cancer (nos)
lung cancer (nos)
ovary, peritoneum, or fallopian tube cancer (nos)
pancreatobiliary or liver cancer (nos)
prostate cancer (nos)
small intestinal, colorectal, or anal cancer (nos)
unspecified primary cancer (nos)

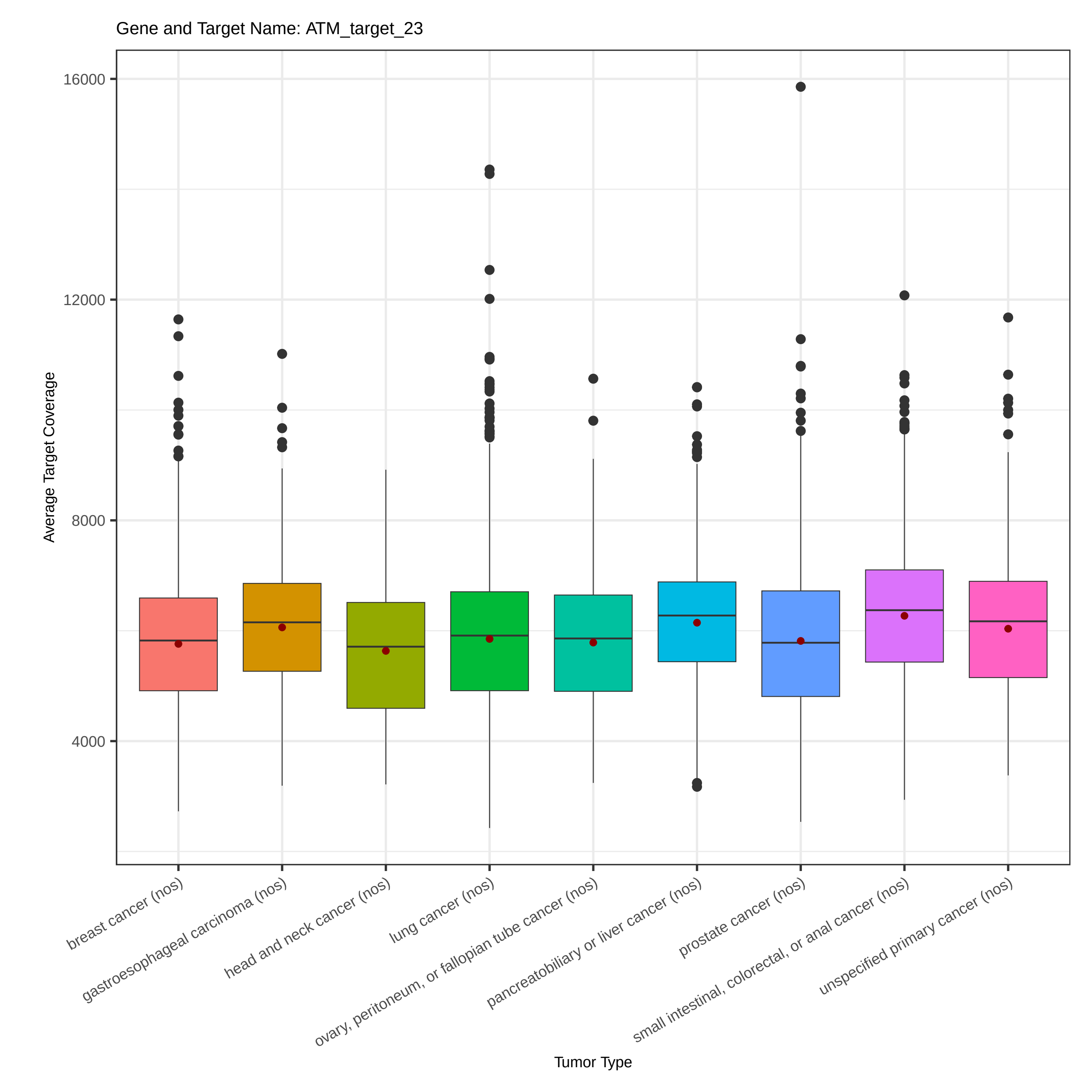
Gene and Target Name: ATM_target_23
Average Target Coverage
16000
12000
8000
4000
breast cancer (nos)
gastroesophageal carcinoma (nos)
head and neck cancer (nos)
lung cancer (nos)
ovary, peritoneum, or fallopian tube cancer (nos)
pancreatobiliary or liver cancer (nos)
prostate cancer (nos)
small intestinal, colorectal, or anal cancer (nos)
unspecified primary cancer (nos)
Tumor Type

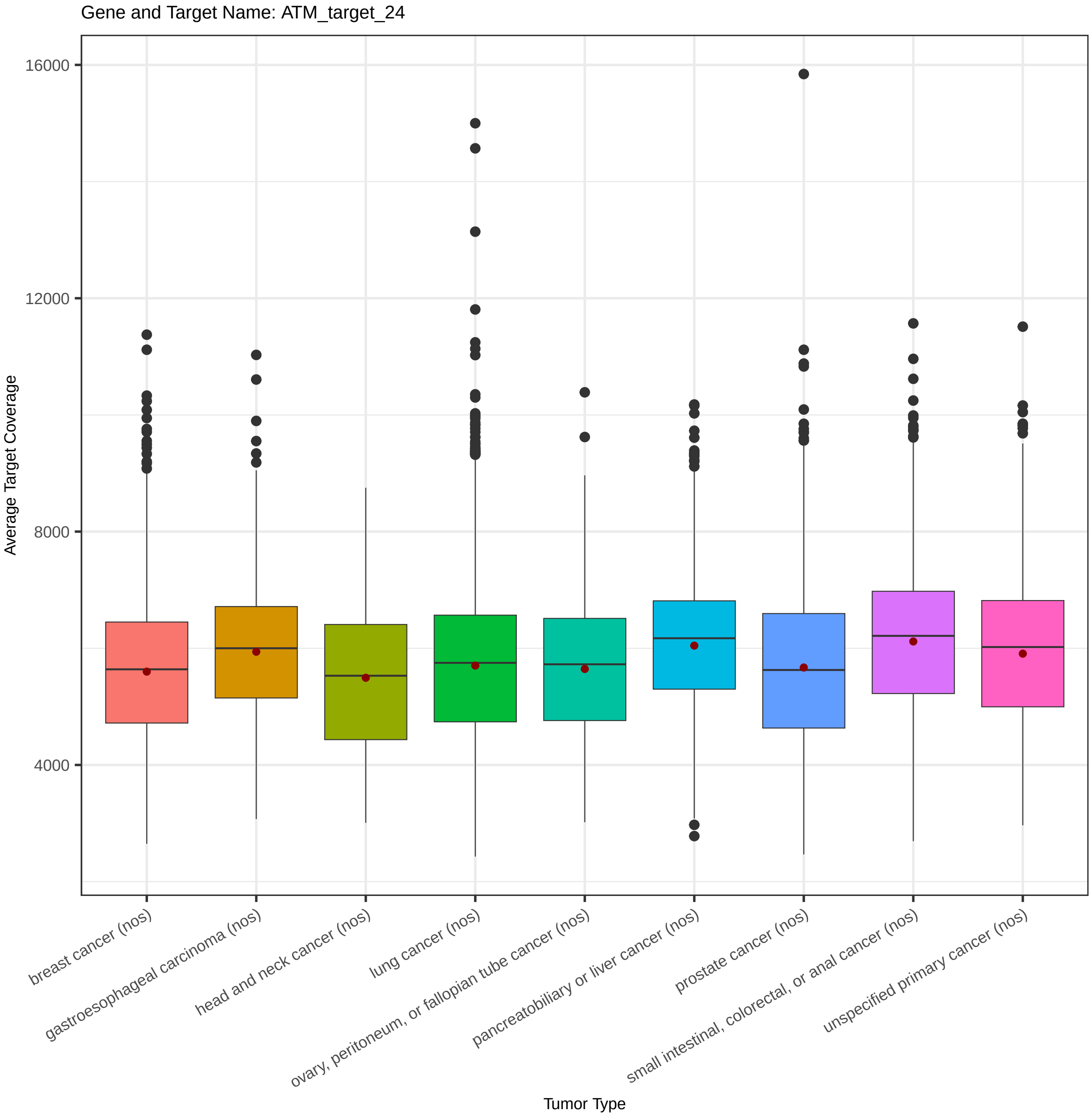

Gene and Target Name: ATM_target_24
Average Target Coverage
16000
12000
8000
4000
breast cancer (nos)
gastroesophageal carcinoma (nos)
head and neck cancer (nos)
lung cancer (nos)
ovary, peritoneum, or fallopian tube cancer (nos)
pancreatobiliary or liver cancer (nos)
prostate cancer (nos)
small intestinal, colorectal, or anal cancer (nos)
unspecified primary cancer (nos)
Tumor Type

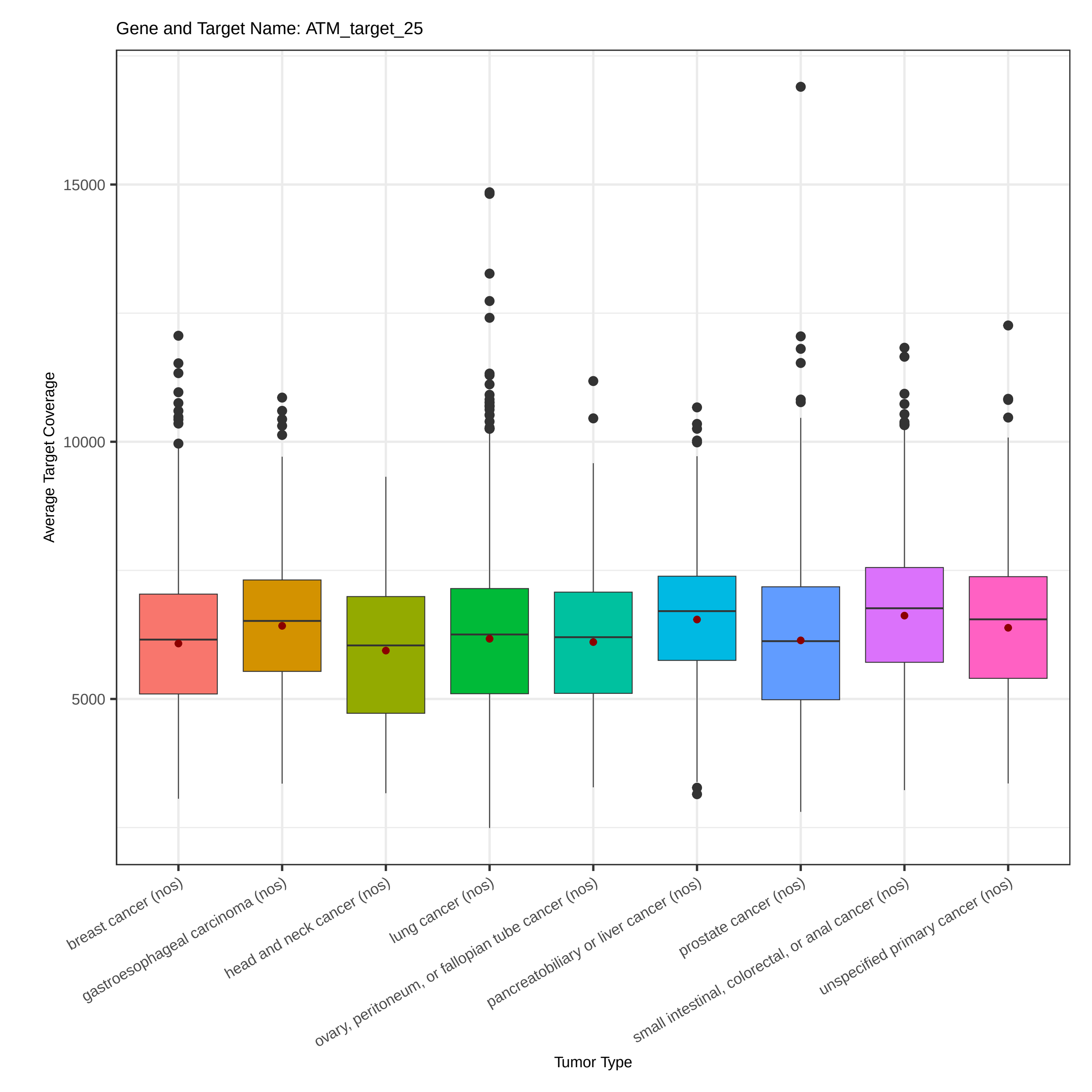

Gene and Target Name: ATM_target_25
Average Target Coverage
15000
10000
5000
breast cancer (nos)
gastroesophageal carcinoma (nos)
head and neck cancer (nos)
lung cancer (nos)
ovary, peritoneum, or fallopian tube cancer (nos)
pancreatobiliary or liver cancer (nos)
prostate cancer (nos)
small intestinal, colorectal, or anal cancer (nos)
unspecified primary cancer (nos)
Tumor Type

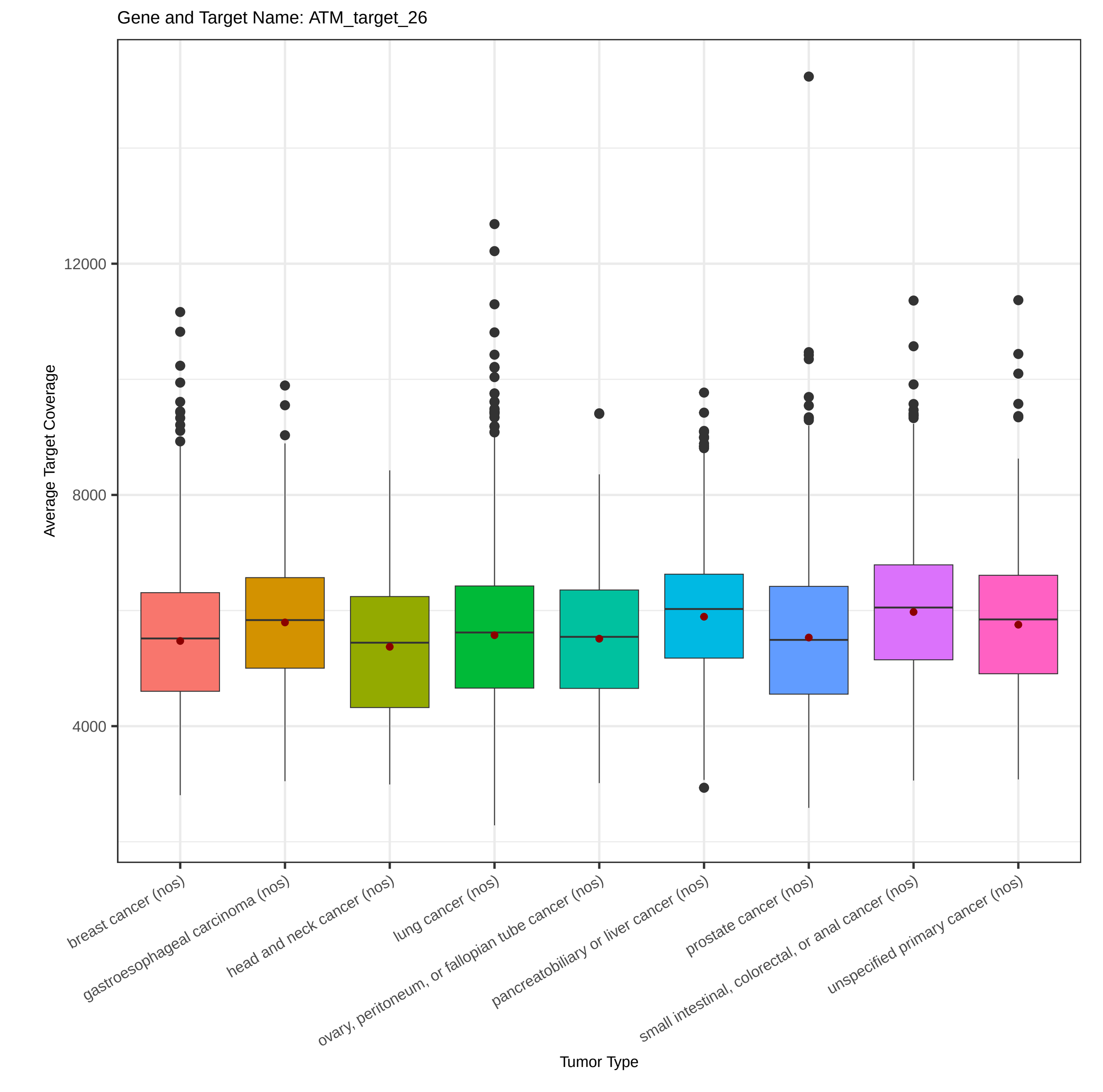

Gene and Target Name: ATM_target_26
Average Target Coverage
12000
8000
4000
breast cancer (nos)
gastroesophageal carcinoma (nos)
head and neck cancer (nos)
lung cancer (nos)
ovary, peritoneum, or fallopian tube cancer (nos)
pancreatobiliary or liver cancer (nos)
prostate cancer (nos)
small intestinal, colorectal, or anal cancer (nos)
unspecified primary cancer (nos)
Tumor Type

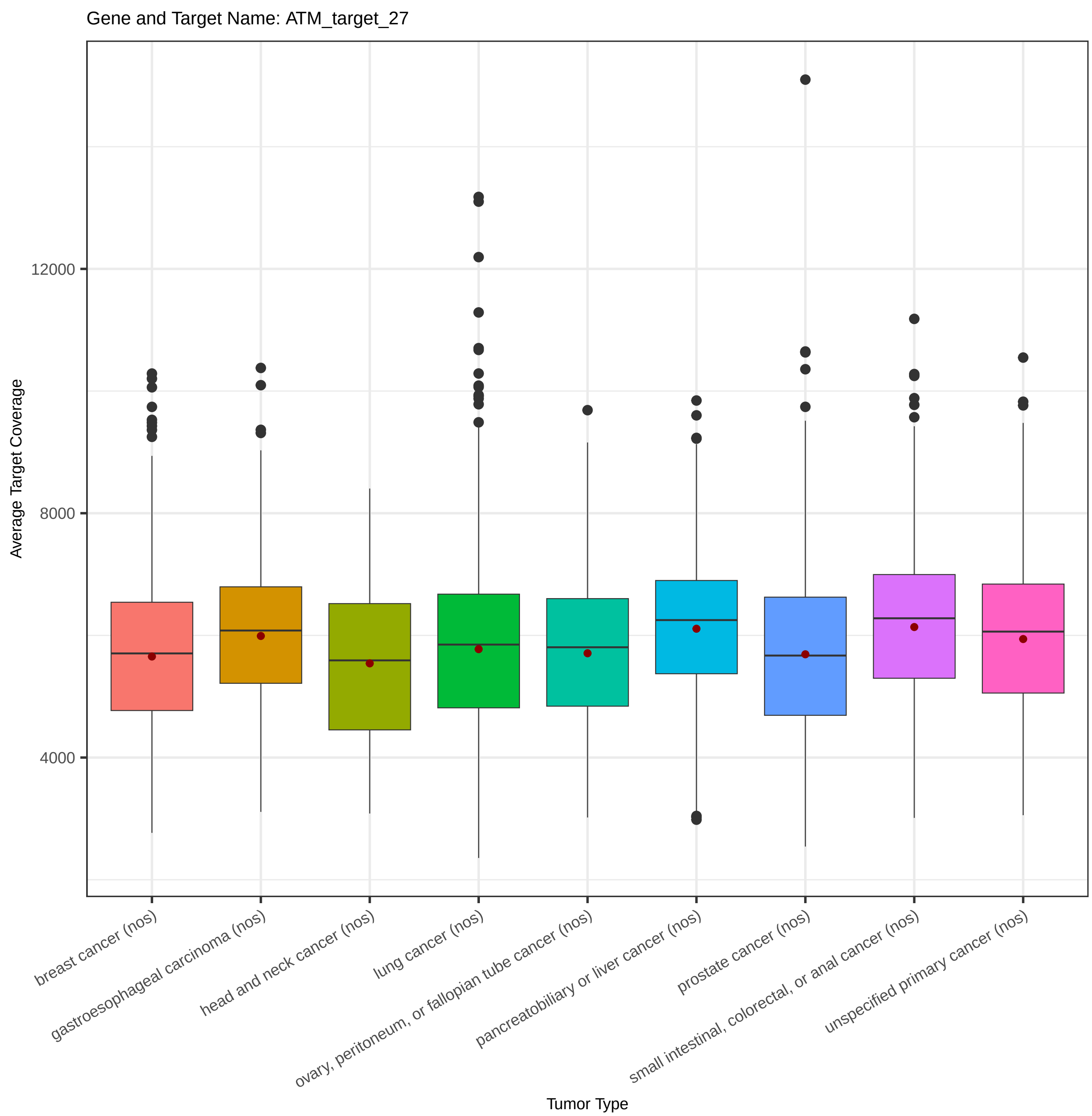
Gene and Target Name: ATM_target_27
Average Target Coverage
12000
8000
4000
breast cancer (nos)
gastroesophageal carcinoma (nos)
head and neck cancer (nos)
lung cancer (nos)
ovary, peritoneum, or fallopian tube cancer (nos)
pancreatobiliary or liver cancer (nos)
prostate cancer (nos)
small intestinal, colorectal, or anal cancer (nos)
unspecified primary cancer (nos)
Tumor Type

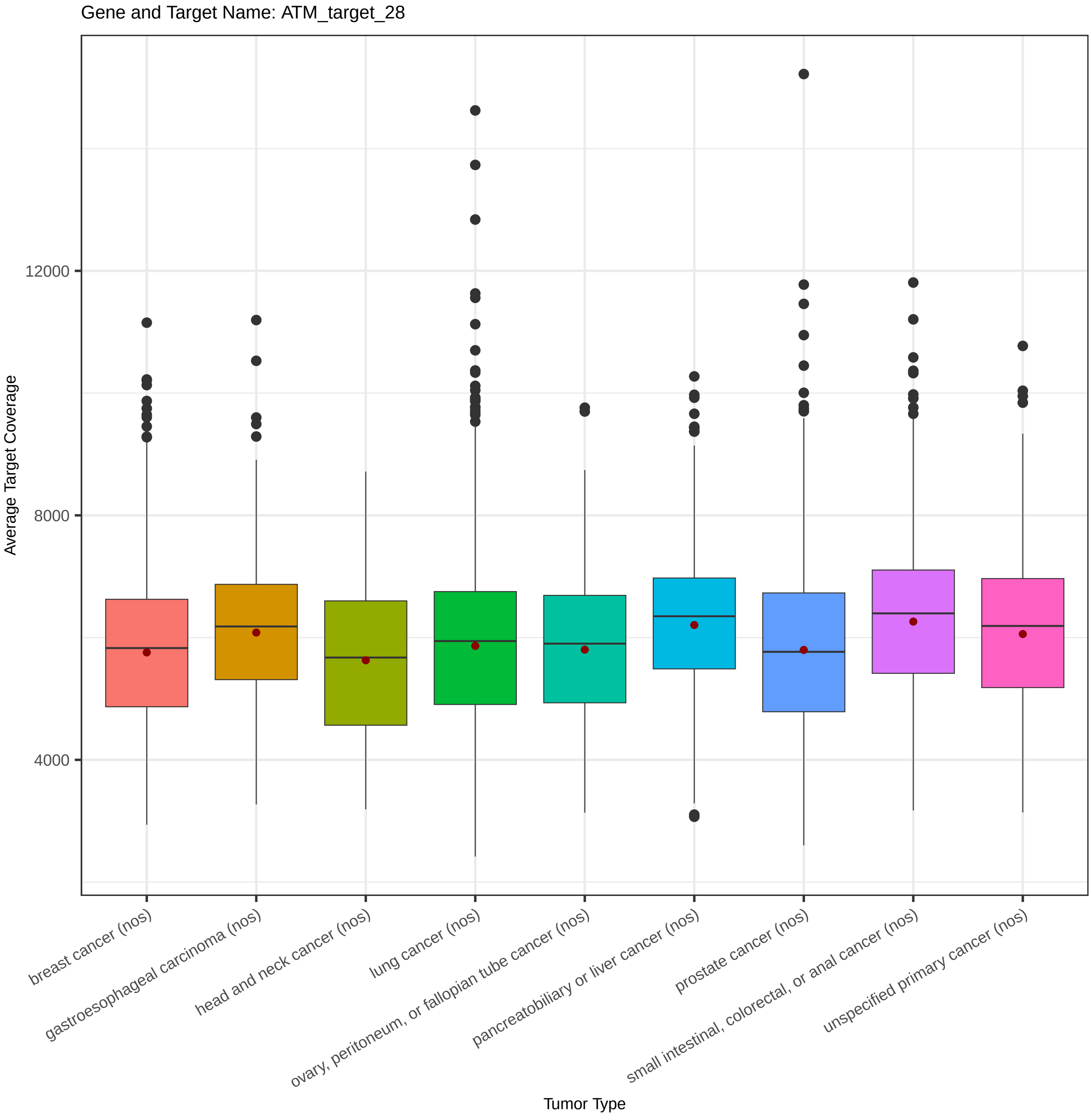
Gene and Target Name: ATM_target_28
Average Target Coverage
12000
8000
4000
breast cancer (nos)
gastroesophageal carcinoma (nos)
head and neck cancer (nos)
lung cancer (nos)
ovary, peritoneum, or fallopian tube cancer (nos)
pancreatobiliary or liver cancer (nos)
prostate cancer (nos)
small intestinal, colorectal, or anal cancer (nos)
unspecified primary cancer (nos)
Tumor Type

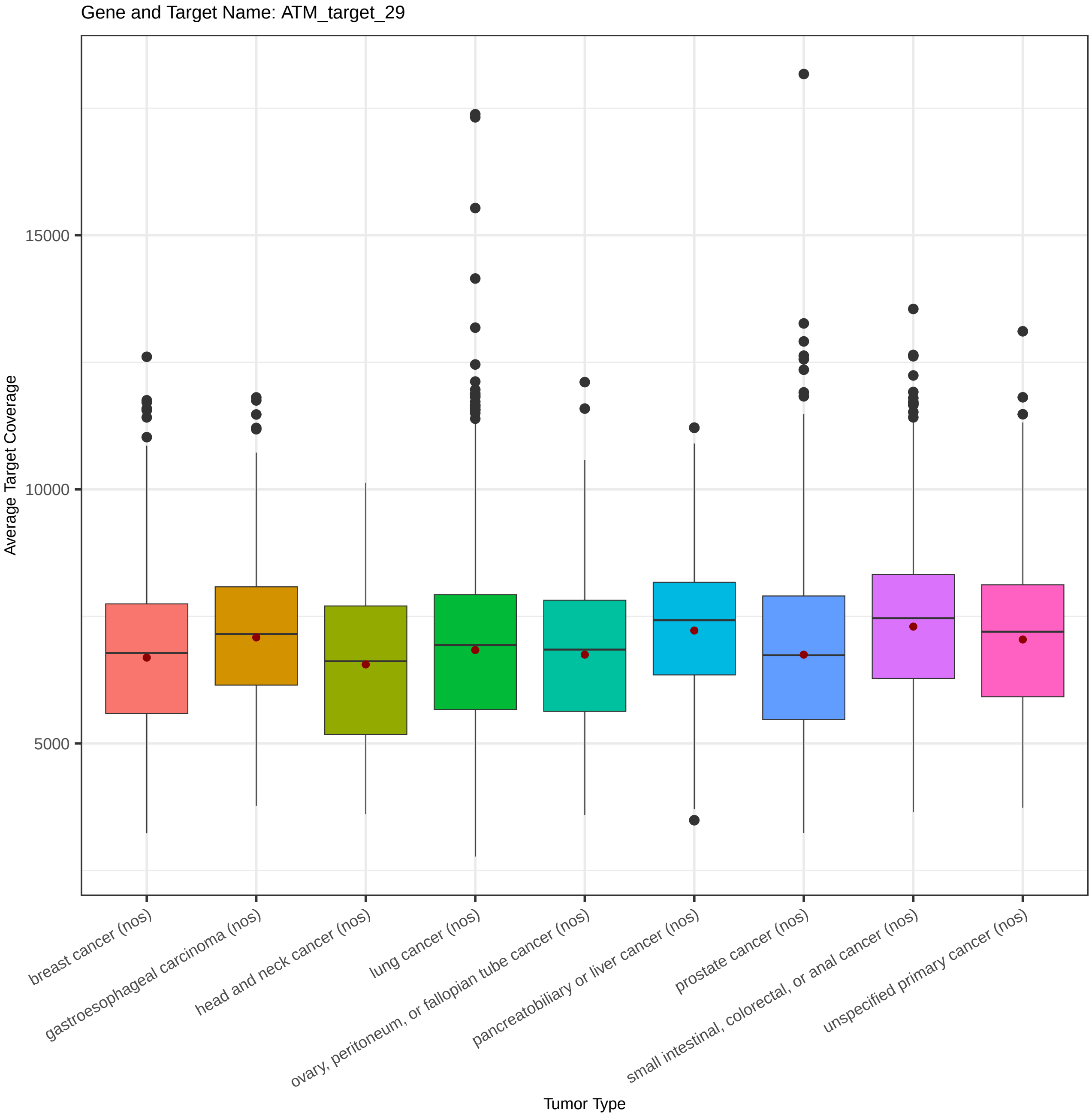
Gene and Target Name: ATM_target_29
Average Target Coverage
15000
10000
5000
breast cancer (nos)
gastroesophageal carcinoma (nos)
head and neck cancer (nos)
lung cancer (nos)
ovary, peritoneum, or fallopian tube cancer (nos)
pancreatobiliary or liver cancer (nos)
prostate cancer (nos)
small intestinal, colorectal, or anal cancer (nos)
unspecified primary cancer (nos)
Tumor Type

Gene and Target Name: ATM_target_30

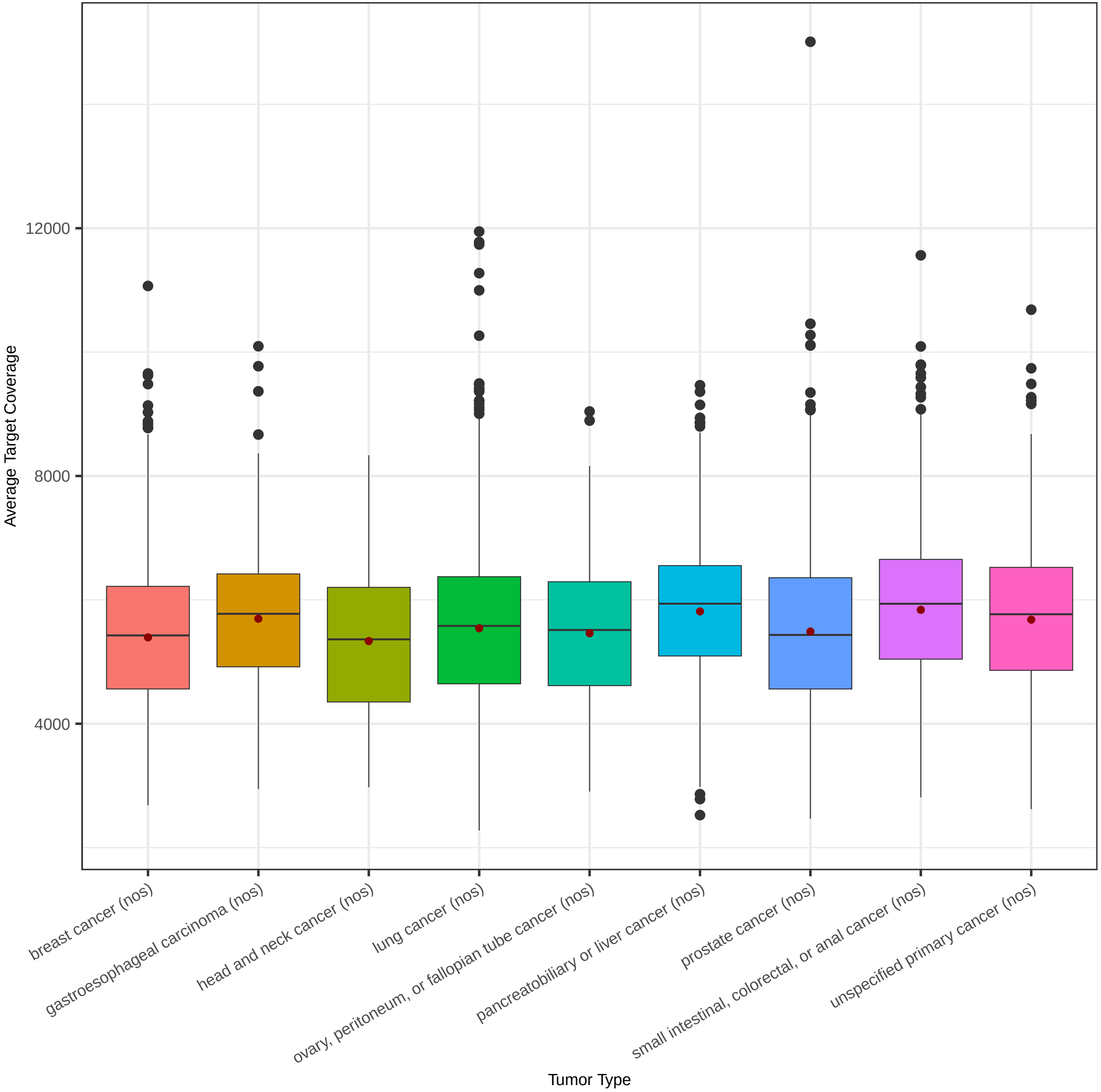

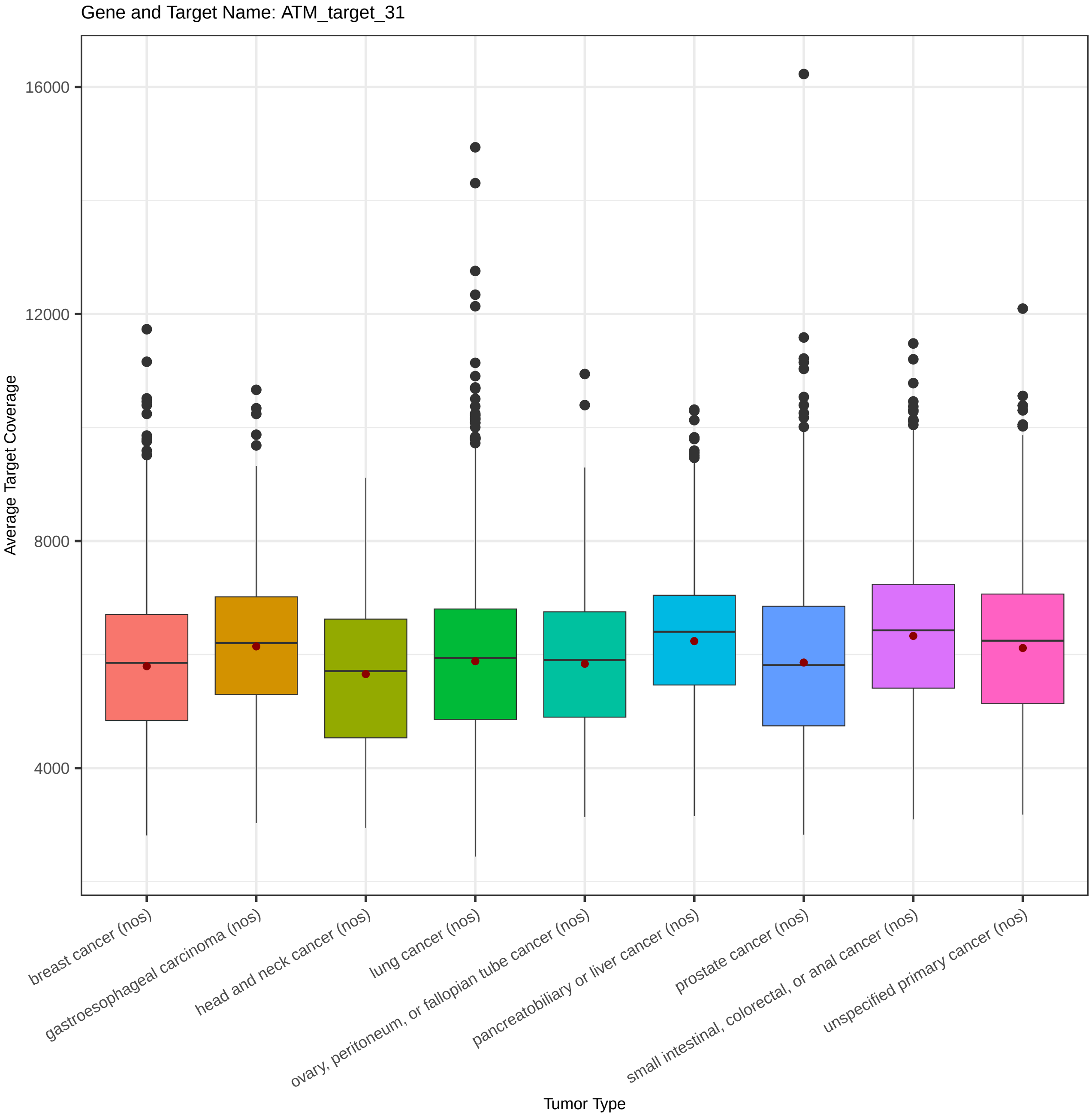

Gene and Target Name: ATM_target_31
Average Target Coverage
16000
12000
8000
4000
breast cancer (nos)
gastroesophageal carcinoma (nos)
head and neck cancer (nos)
lung cancer (nos)
ovary, peritoneum, or fallopian tube cancer (nos)
pancreatobiliary or liver cancer (nos)
prostate cancer (nos)
small intestinal, colorectal, or anal cancer (nos)
unspecified primary cancer (nos)
Tumor Type

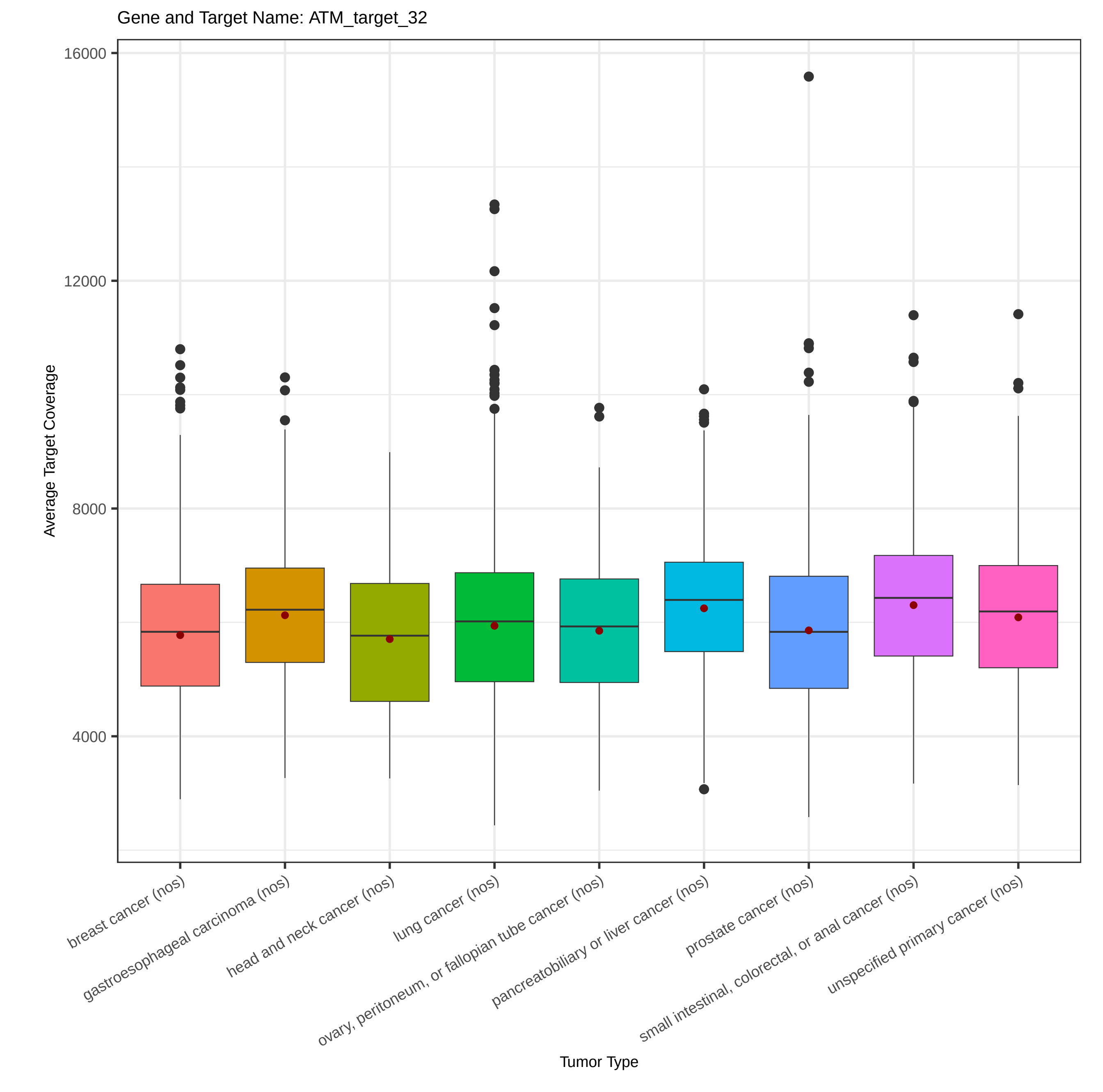
Gene and Target Name: ATM_target_32
Average Target Coverage
16000
12000
8000
4000
breast cancer (nos)
gastroesophageal carcinoma (nos)
head and neck cancer (nos)
lung cancer (nos)
ovary, peritoneum, or fallopian tube cancer (nos)
pancreatobiliary or liver cancer (nos)
prostate cancer (nos)
small intestinal, colorectal, or anal cancer (nos)
unspecified primary cancer (nos)
Tumor Type

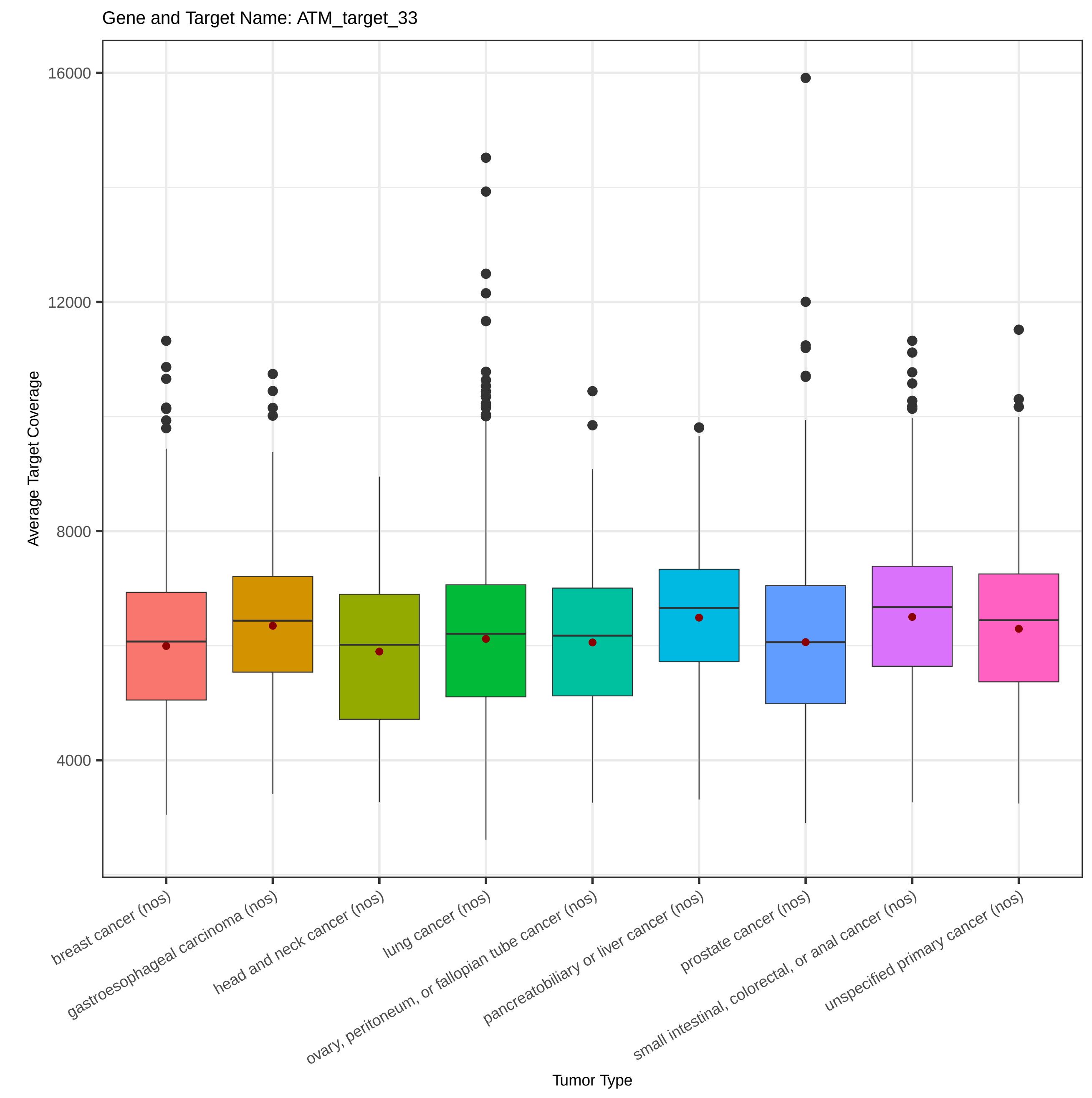

Gene and Target Name: ATM_target_33
16000
12000
8000
4000
Average Target Coverage
breast cancer (nos)
gastroesophageal carcinoma (nos)
head and neck cancer (nos)
lung cancer (nos)
ovary, peritoneum, or fallopian tube cancer (nos)
pancreatobiliary or liver cancer (nos)
prostate cancer (nos)
small intestinal, colorectal, or anal cancer (nos)
unspecified primary cancer (nos)
Tumor Type

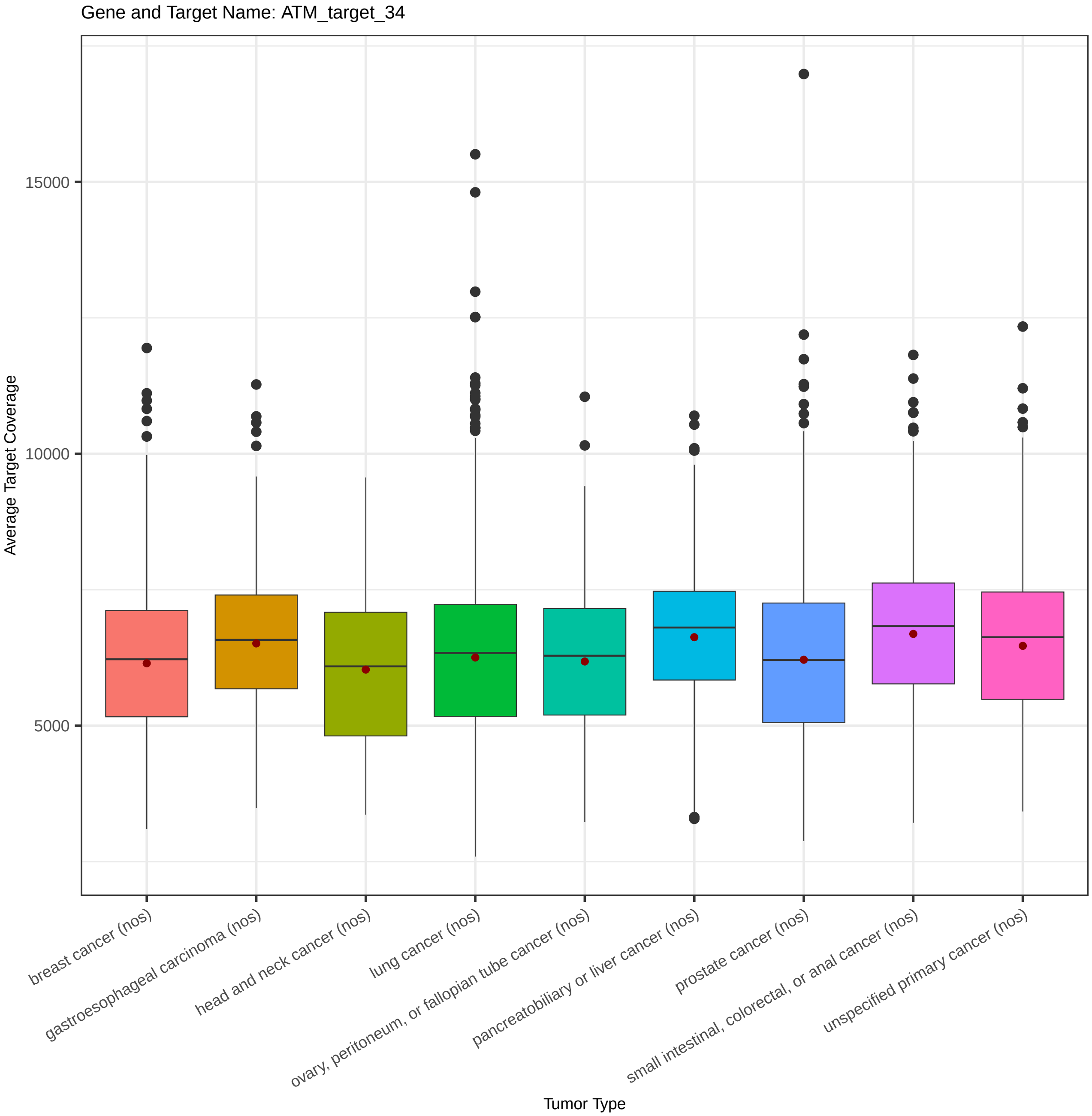
Gene and Target Name: ATM_target_34
Average Target Coverage
15000
10000
5000
breast cancer (nos)
gastroesophageal carcinoma (nos)
head and neck cancer (nos)
lung cancer (nos)
ovary, peritoneum, or fallopian tube cancer (nos)
pancreatobiliary or liver cancer (nos)
prostate cancer (nos)
small intestinal, colorectal, or anal cancer (nos)
unspecified primary cancer (nos)
Tumor Type

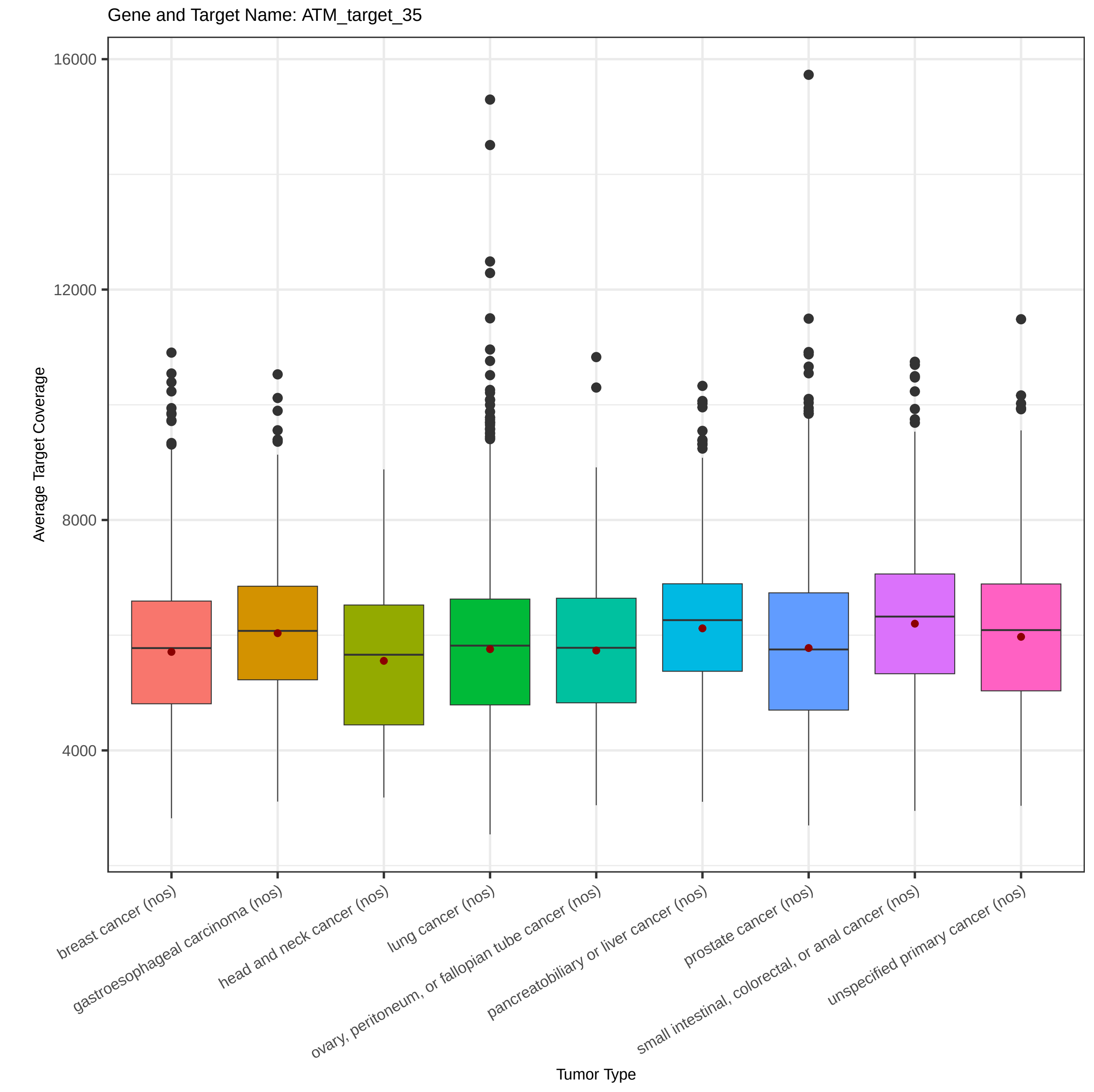

Gene and Target Name: ATM_target_35
16000
12000
8000
4000
Average Target Coverage
breast cancer (nos)
gastroesophageal carcinoma (nos)
head and neck cancer (nos)
lung cancer (nos)
ovary, peritoneum, or fallopian tube cancer (nos)
pancreatobiliary or liver cancer (nos)
prostate cancer (nos)
small intestinal, colorectal, or anal cancer (nos)
unspecified primary cancer (nos)
Tumor Type

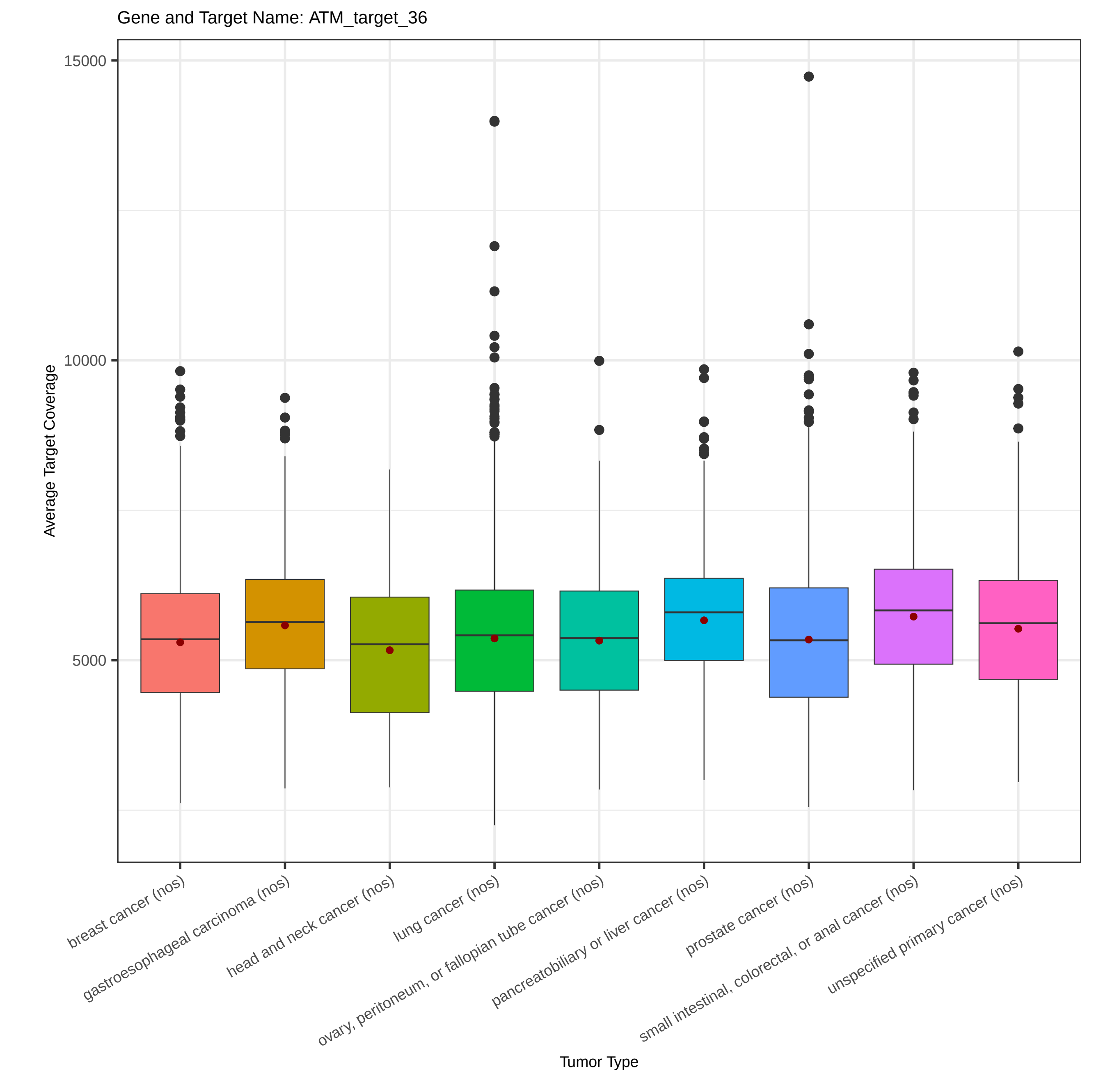

Gene and Target Name: ATM_target_36
15000
10000
5000
Average Target Coverage
breast cancer (nos)
gastroesophageal carcinoma (nos)
head and neck cancer (nos)
lung cancer (nos)
ovary, peritoneum, or fallopian tube cancer (nos)
pancreatobiliary or liver cancer (nos)
prostate cancer (nos)
small intestinal, colorectal, or anal cancer (nos)
unspecified primary cancer (nos)
Tumor Type

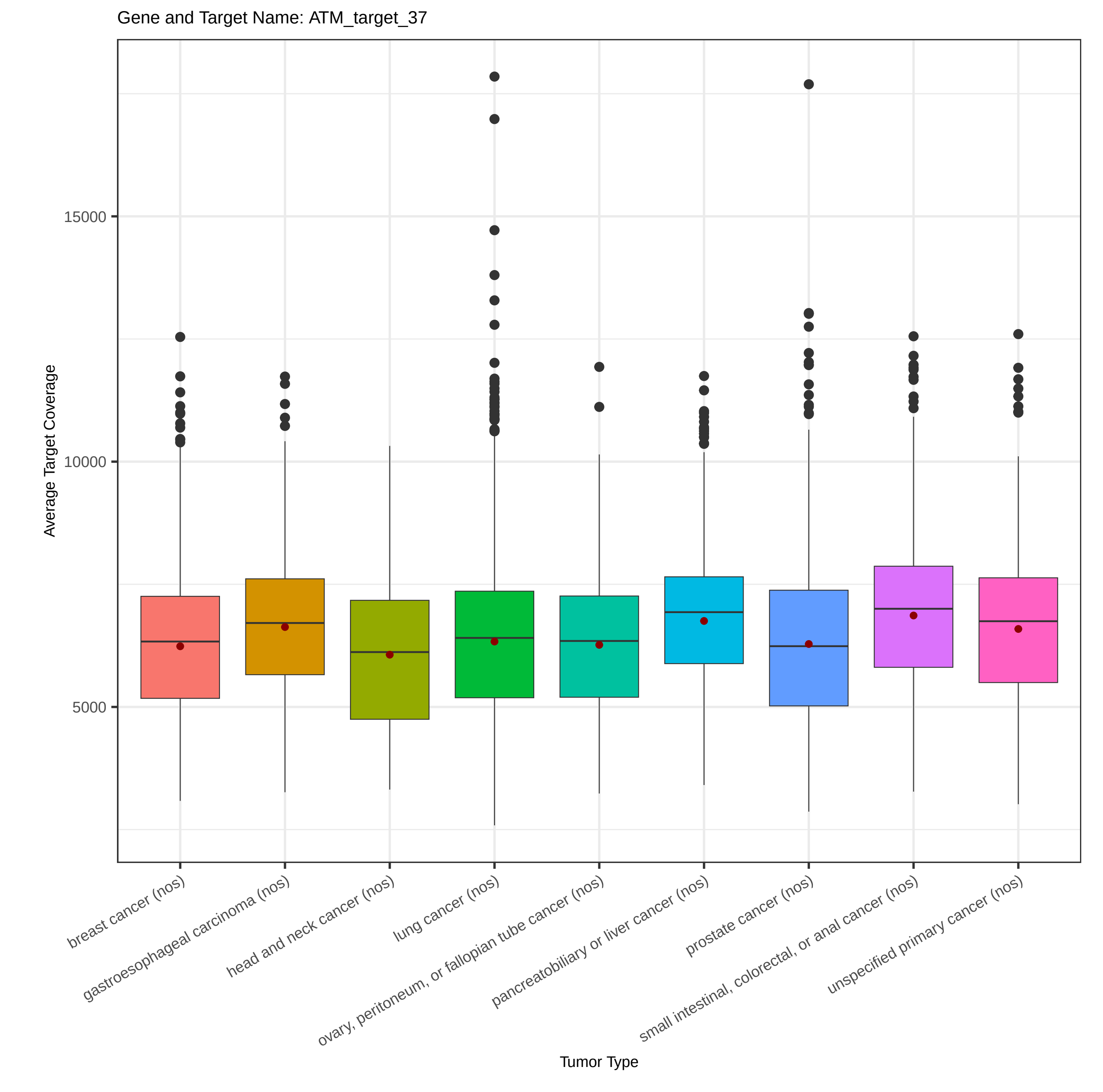

Gene and Target Name: ATM_target_37
15000
10000
5000
Average Target Coverage
breast cancer (nos)
gastroesophageal carcinoma (nos)
head and neck cancer (nos)
lung cancer (nos)
ovary, peritoneum, or fallopian tube cancer (nos)
pancreatobiliary or liver cancer (nos)
prostate cancer (nos)
small intestinal, colorectal, or anal cancer (nos)
unspecified primary cancer (nos)
Tumor Type

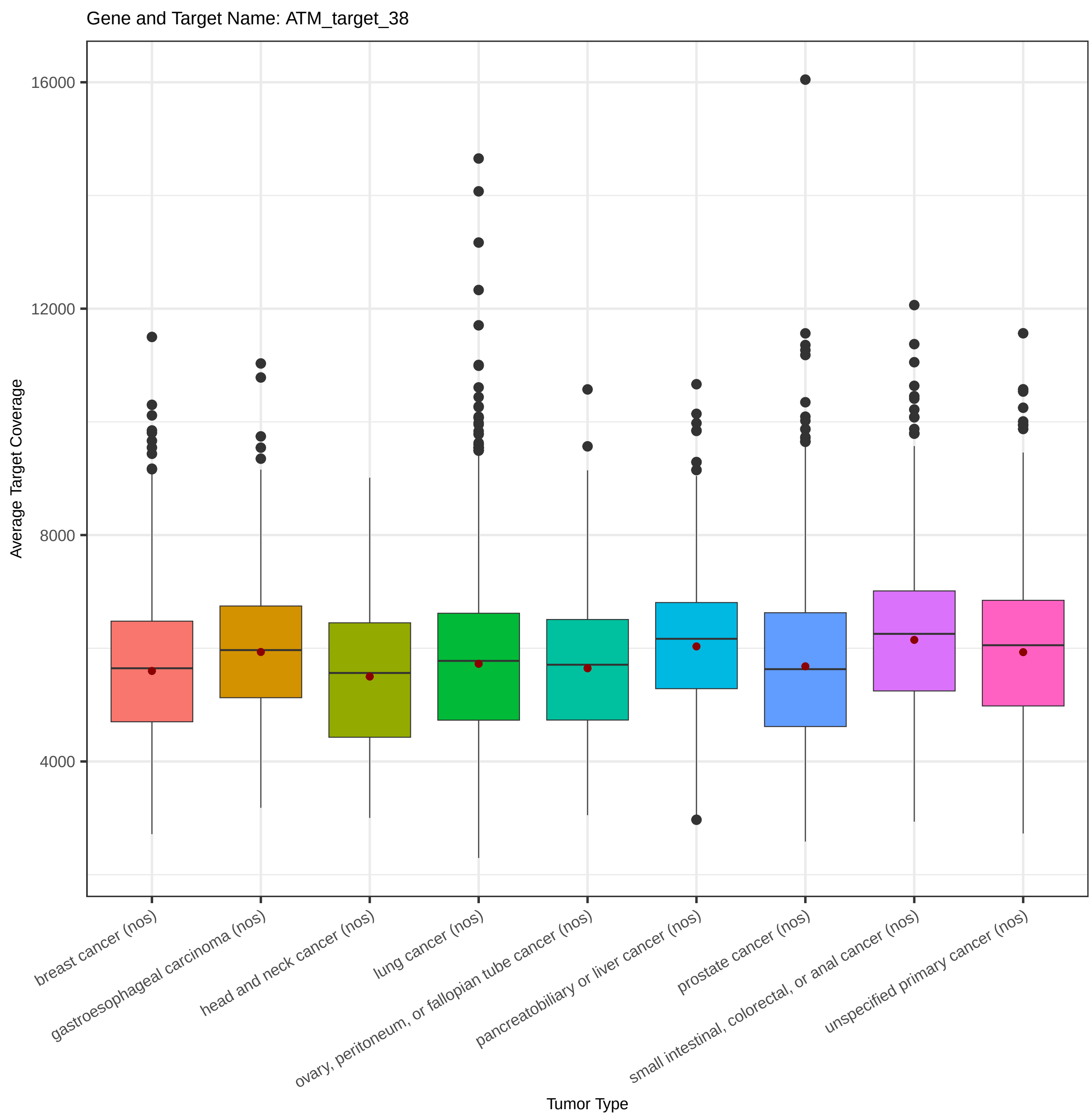

Gene and Target Name: ATM_target_38
Average Target Coverage
16000
12000
8000
4000
breast cancer (nos)
gastroesophageal carcinoma (nos)
head and neck cancer (nos)
lung cancer (nos)
ovary, peritoneum, or fallopian tube cancer (nos)
pancreatobiliary or liver cancer (nos)
prostate cancer (nos)
small intestinal, colorectal, or anal cancer (nos)
unspecified primary cancer (nos)
Tumor Type

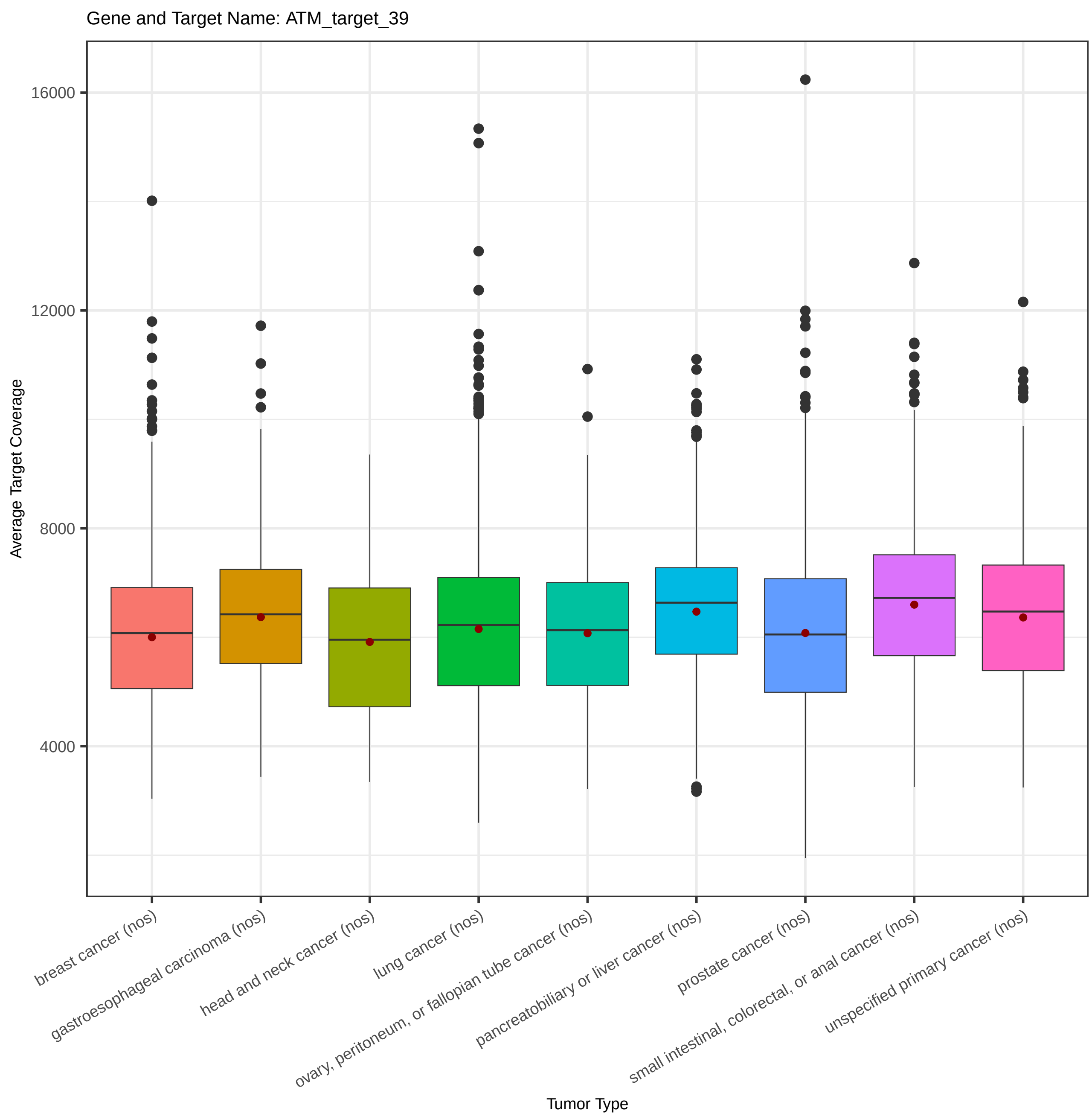

Gene and Target Name: ATM_target_39
Average Target Coverage
16000
12000
8000
4000
breast cancer (nos)
gastroesophageal carcinoma (nos)
head and neck cancer (nos)
lung cancer (nos)
ovary, peritoneum, or fallopian tube cancer (nos)
pancreatobiliary or liver cancer (nos)
prostate cancer (nos)
small intestinal, colorectal, or anal cancer (nos)
unspecified primary cancer (nos)
Tumor Type

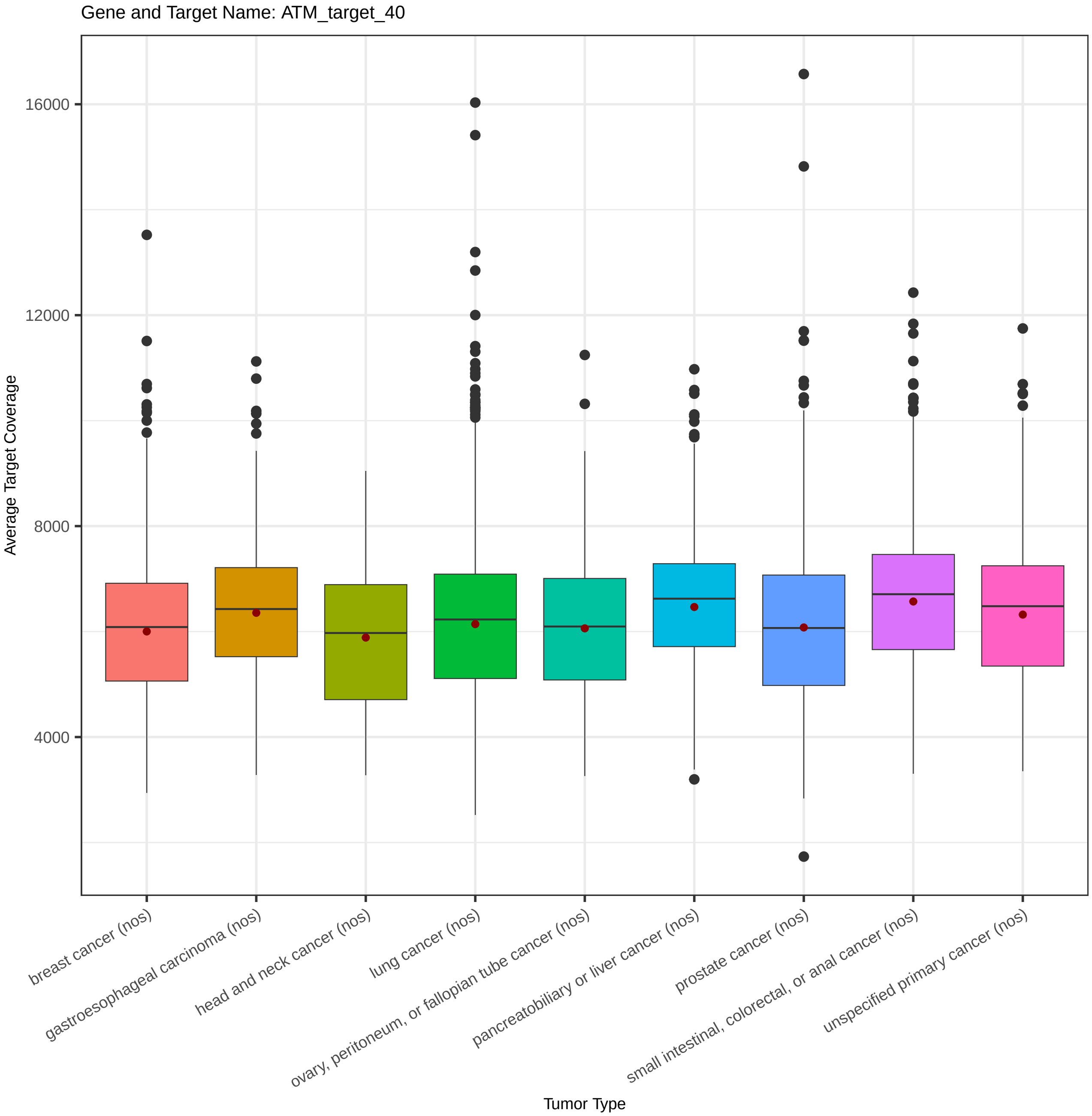

Gene and Target Name: ATM_target_40
Average Target Coverage
16000
12000
8000
4000
breast cancer (nos)
gastroesophageal carcinoma (nos)
head and neck cancer (nos)
lung cancer (nos)
ovary, peritoneum, or fallopian tube cancer (nos)
pancreatobiliary or liver cancer (nos)
prostate cancer (nos)
small intestinal, colorectal, or anal cancer (nos)
unspecified primary cancer (nos)
Tumor Type

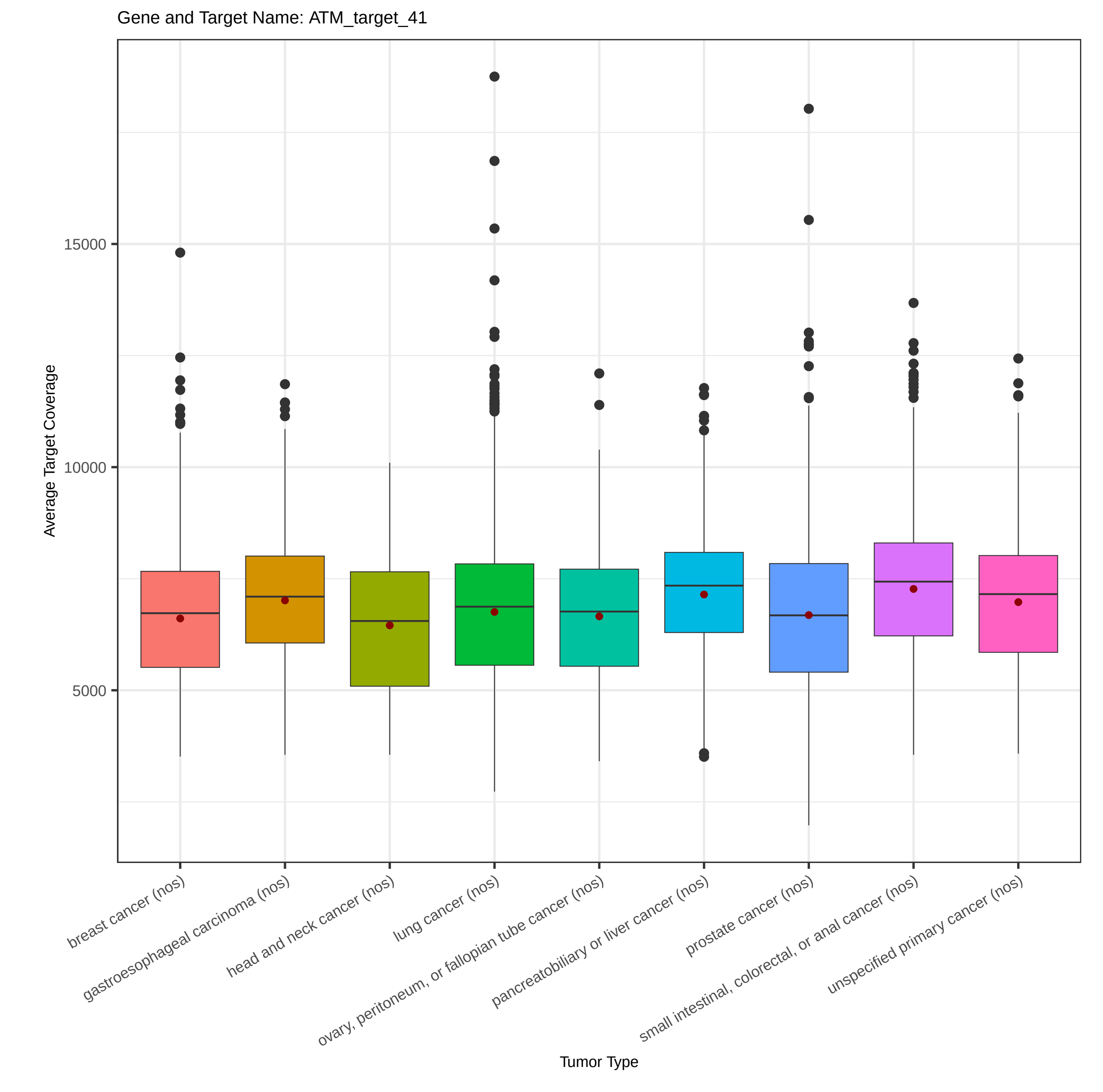

Gene and Target Name: ATM_target_41
Average Target Coverage
15000
10000
5000
breast cancer (nos)
gastroesophageal carcinoma (nos)
head and neck cancer (nos)
lung cancer (nos)
ovary, peritoneum, or fallopian tube cancer (nos)
pancreatobiliary or liver cancer (nos)
prostate cancer (nos)
small intestinal, colorectal, or anal cancer (nos)
unspecified primary cancer (nos)
Tumor Type

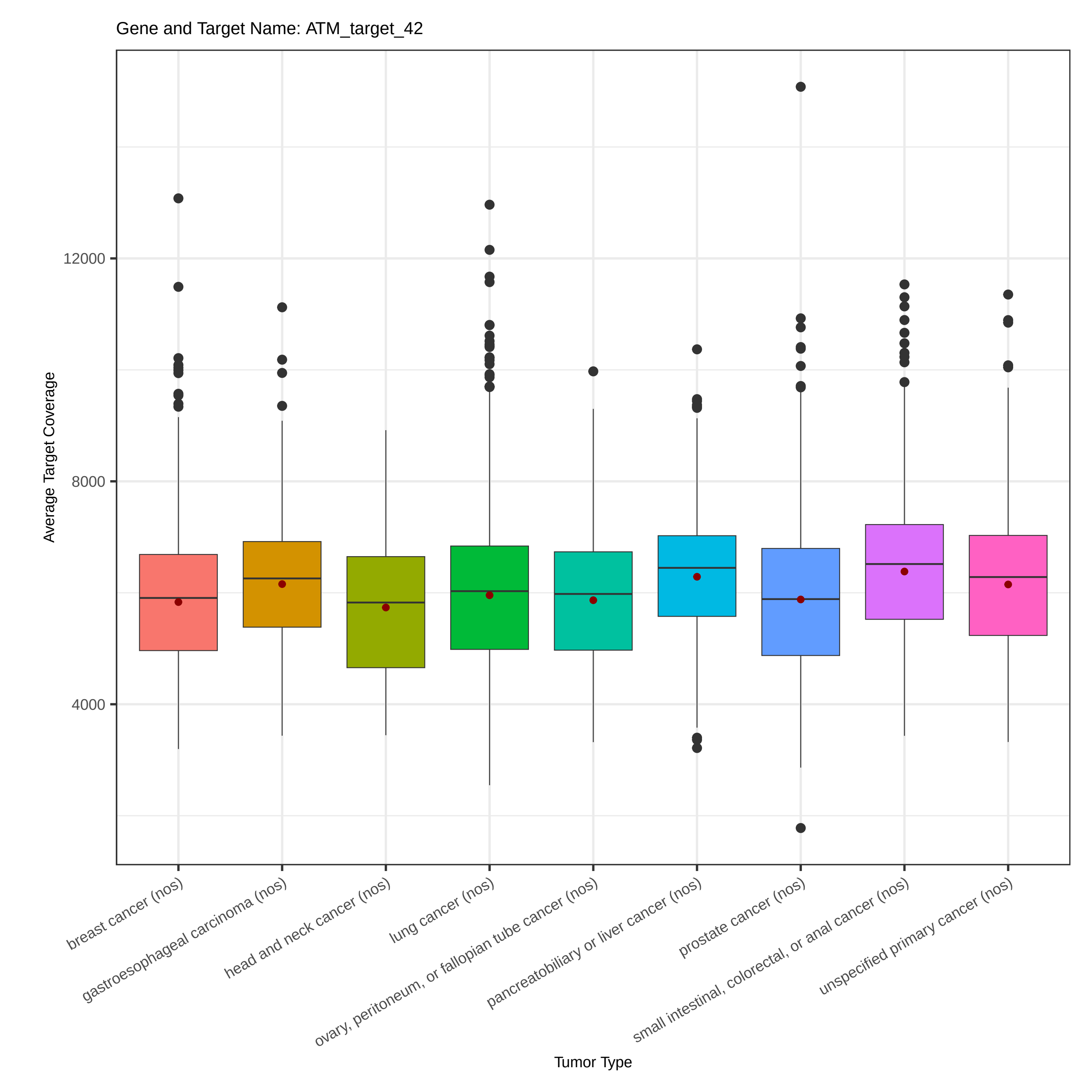

Gene and Target Name: ATM_target_42
Average Target Coverage
12000
8000
4000
breast cancer (nos)
gastroesophageal carcinoma (nos)
head and neck cancer (nos)
lung cancer (nos)
ovary, peritoneum, or fallopian tube cancer (nos)
pancreatobiliary or liver cancer (nos)
prostate cancer (nos)
small intestinal, colorectal, or anal cancer (nos)
unspecified primary cancer (nos)
Tumor Type

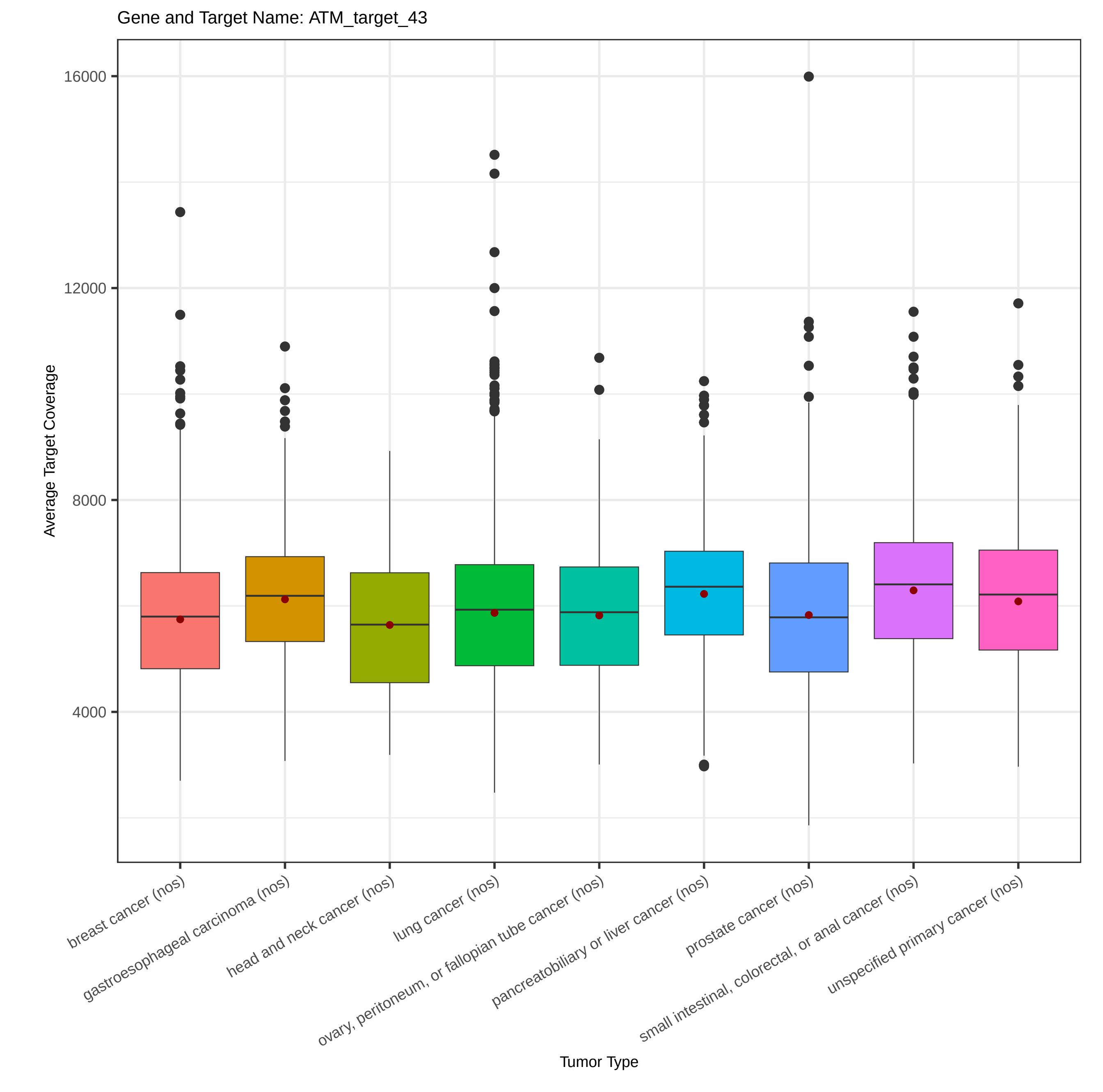

Gene and Target Name: ATM_target_43
Average Target Coverage
16000
12000
8000
4000
breast cancer (nos)
gastroesophageal carcinoma (nos)
head and neck cancer (nos)
lung cancer (nos)
ovary, peritoneum, or fallopian tube cancer (nos)
pancreatobiliary or liver cancer (nos)
prostate cancer (nos)
small intestinal, colorectal, or anal cancer (nos)
unspecified primary cancer (nos)
Tumor Type

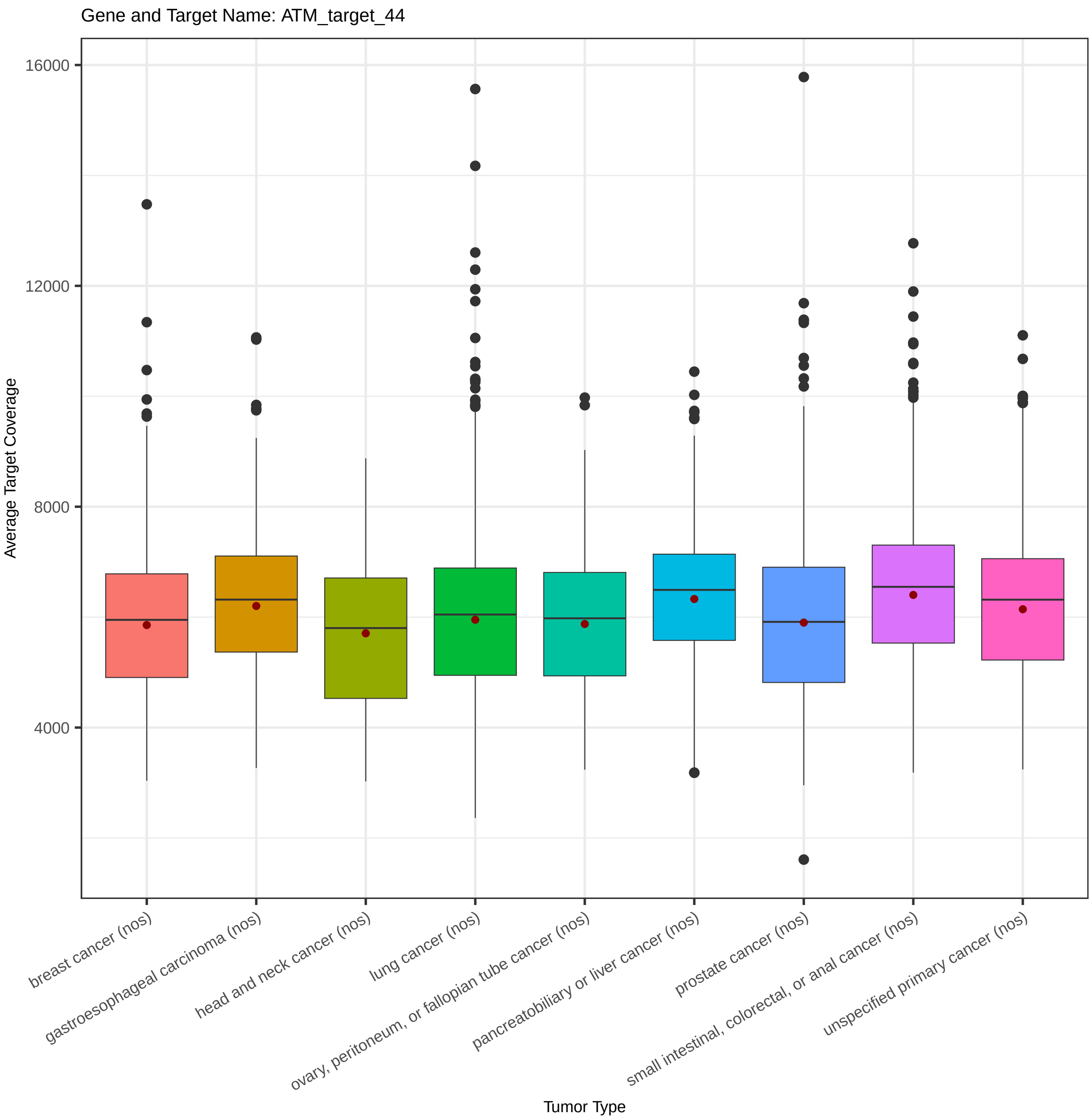
Gene and Target Name: ATM_target_44
Average Target Coverage
16000
12000
8000
4000
breast cancer (nos)
gastroesophageal carcinoma (nos)
head and neck cancer (nos)
lung cancer (nos)
ovary, peritoneum, or fallopian tube cancer (nos)
pancreatobiliary or liver cancer (nos)
prostate cancer (nos)
small intestinal, colorectal, or anal cancer (nos)
unspecified primary cancer (nos)
Tumor Type

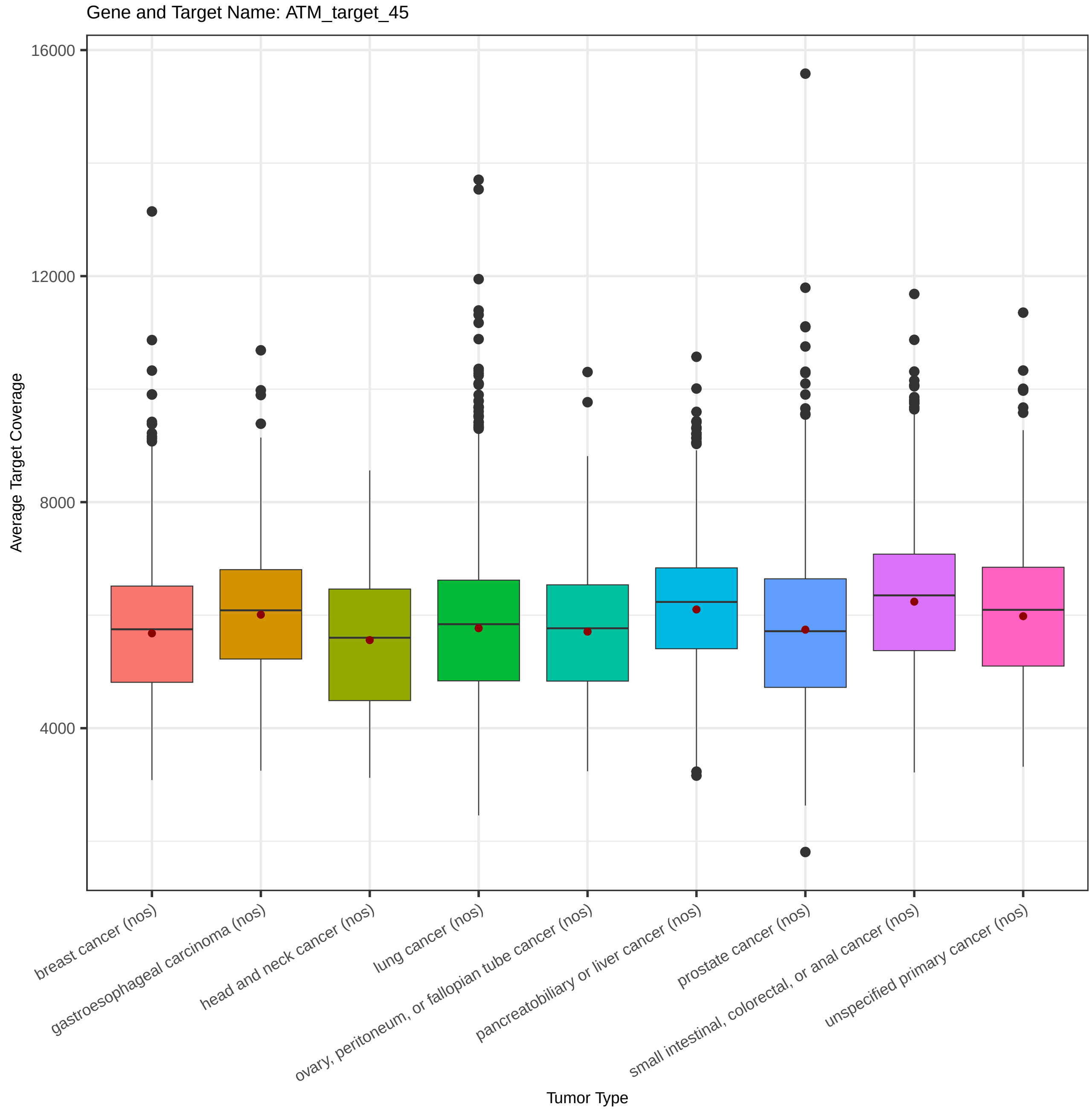
Gene and Target Name: ATM_target_45
Average Target Coverage
16000
12000
8000
4000
breast cancer (nos)
gastroesophageal carcinoma (nos)
head and neck cancer (nos)
lung cancer (nos)
ovary, peritoneum, or fallopian tube cancer (nos)
pancreatobiliary or liver cancer (nos)
prostate cancer (nos)
small intestinal, colorectal, or anal cancer (nos)
unspecified primary cancer (nos)
Tumor Type

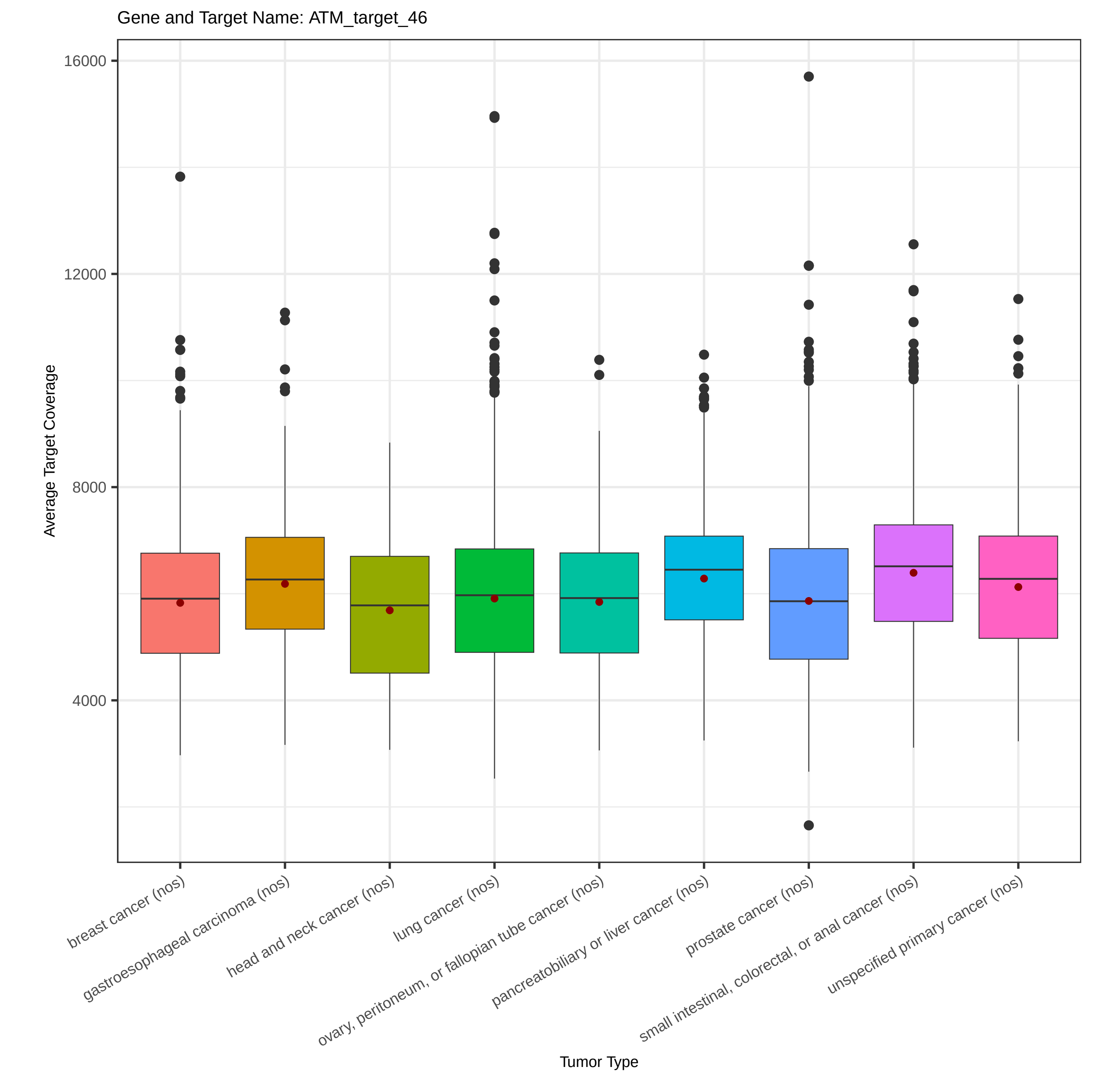

Gene and Target Name: ATM_target_46
Average Target Coverage
16000
12000
8000
4000
breast cancer (nos)
gastroesophageal carcinoma (nos)
head and neck cancer (nos)
lung cancer (nos)
ovary, peritoneum, or fallopian tube cancer (nos)
pancreatobiliary or liver cancer (nos)
prostate cancer (nos)
small intestinal, colorectal, or anal cancer (nos)
unspecified primary cancer (nos)
Tumor Type

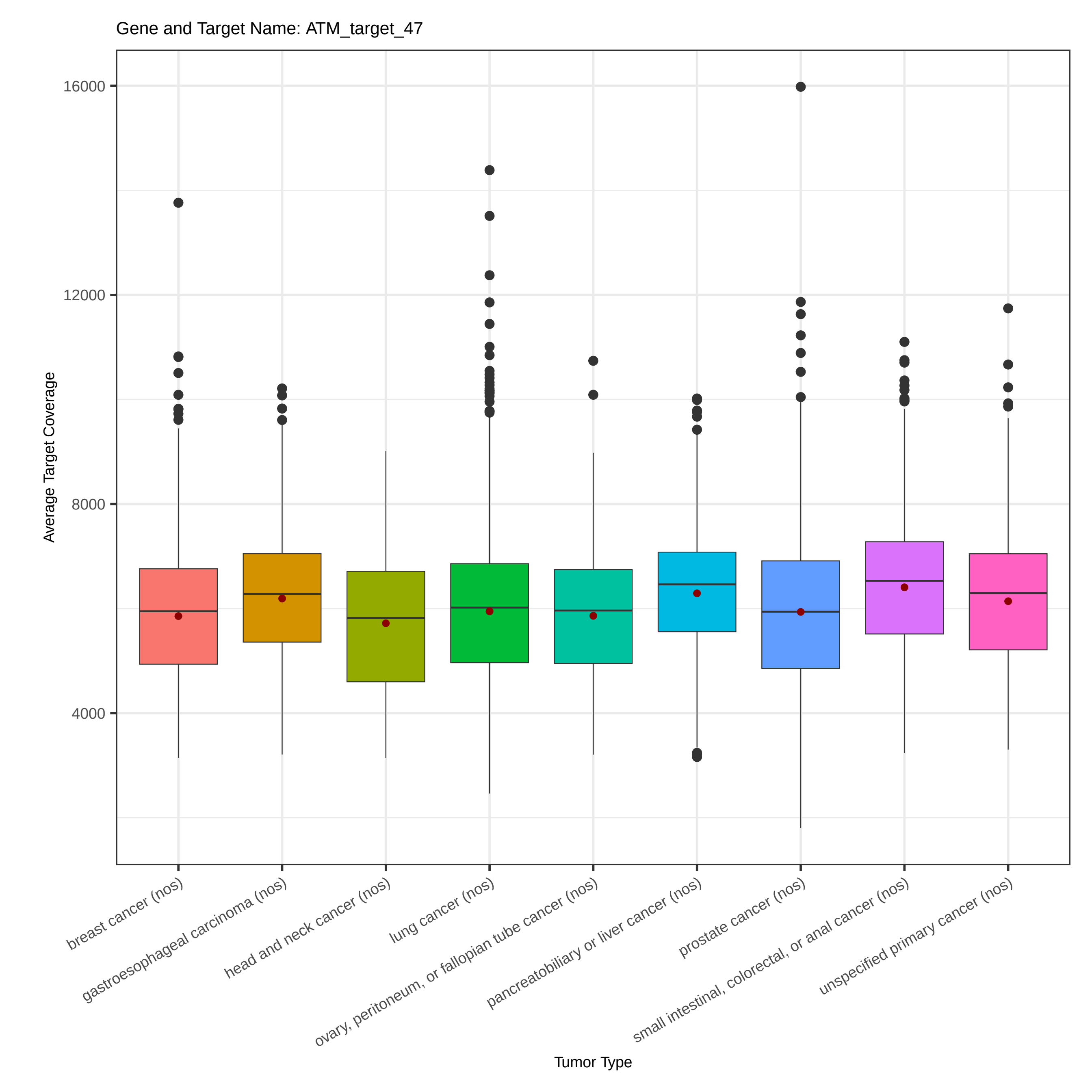

Gene and Target Name: ATM_target_47
Average Target Coverage
16000
12000
8000
4000
breast cancer (nos)
gastroesophageal carcinoma (nos)
head and neck cancer (nos)
lung cancer (nos)
ovary, peritoneum, or fallopian tube cancer (nos)
pancreatobiliary or liver cancer (nos)
prostate cancer (nos)
small intestinal, colorectal, or anal cancer (nos)
unspecified primary cancer (nos)
Tumor Type

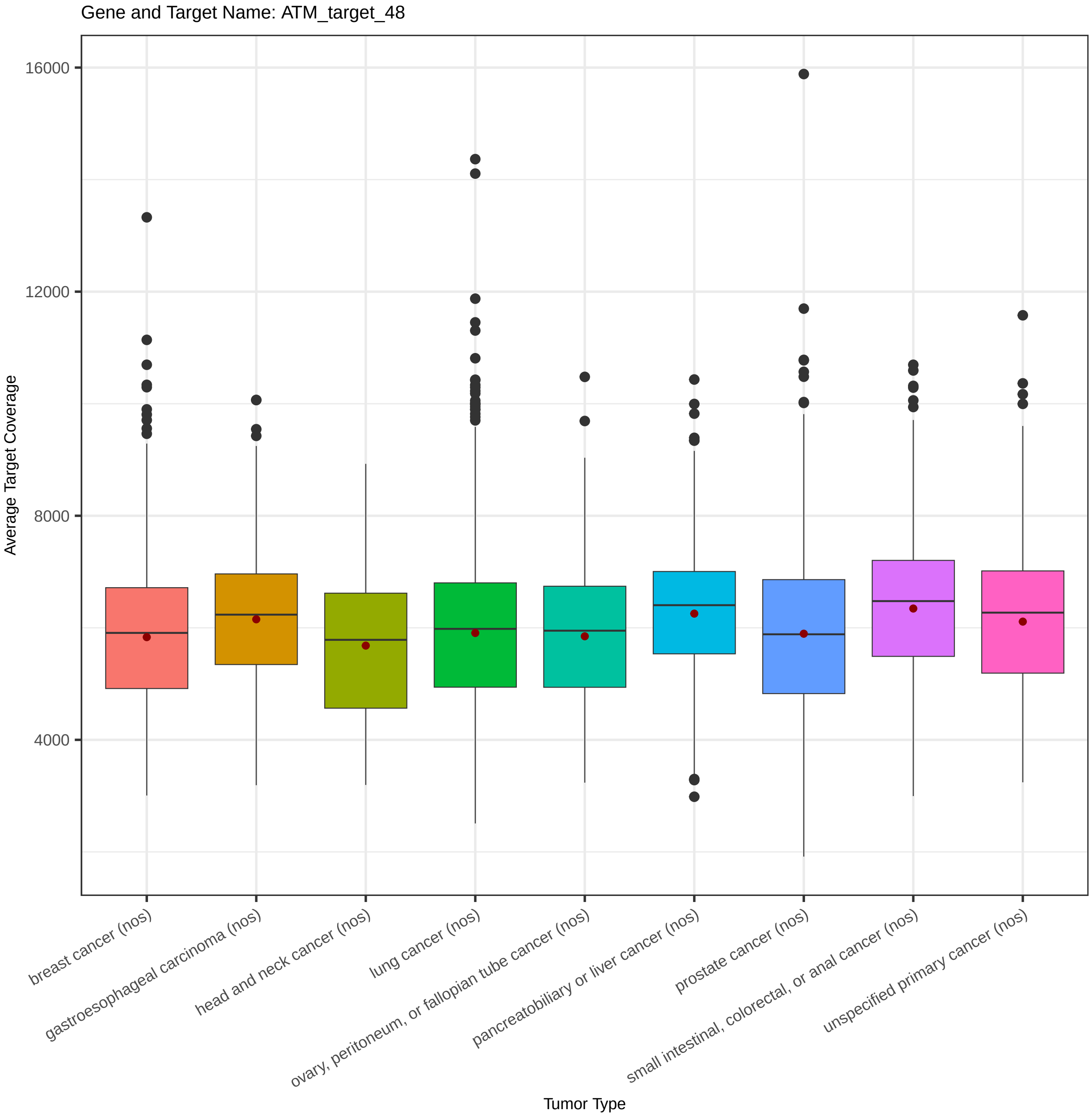

Gene and Target Name: ATM_target_48
Average Target Coverage
16000
12000
8000
4000
Tumor Type
breast cancer (nos)
gastroesophageal carcinoma (nos)
head and neck cancer (nos)
lung cancer (nos)
ovary, peritoneum, or fallopian tube cancer (nos)
pancreatobiliary or liver cancer (nos)
prostate cancer (nos)
small intestinal, colorectal, or anal cancer (nos)
unspecified primary cancer (nos)

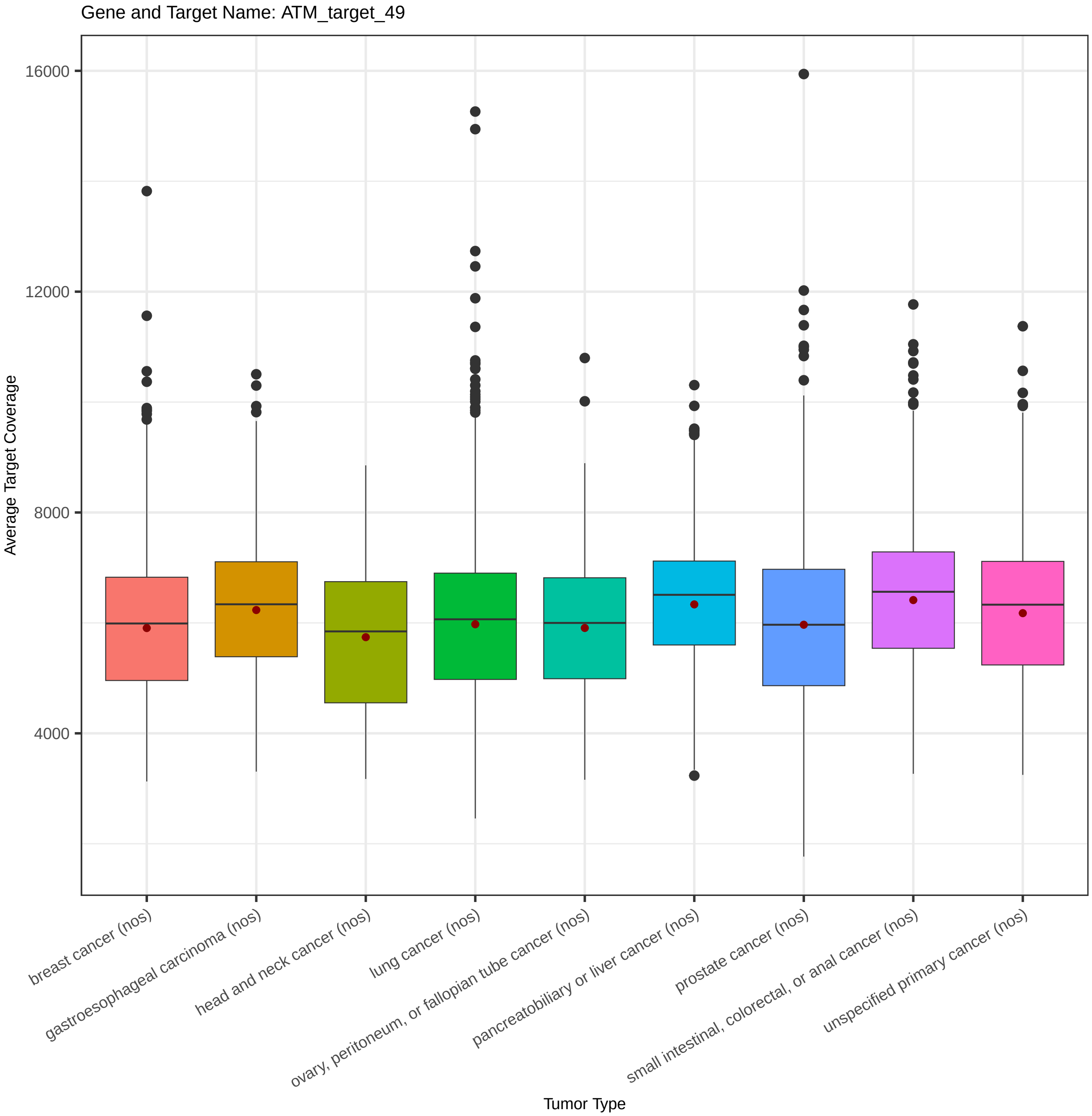
Gene and Target Name: ATM_target_49
Average Target Coverage
16000
12000
8000
4000
breast cancer (nos)
gastroesophageal carcinoma (nos)
head and neck cancer (nos)
lung cancer (nos)
ovary, peritoneum, or fallopian tube cancer (nos)
pancreatobiliary or liver cancer (nos)
prostate cancer (nos)
small intestinal, colorectal, or anal cancer (nos)
unspecified primary cancer (nos)
Tumor Type

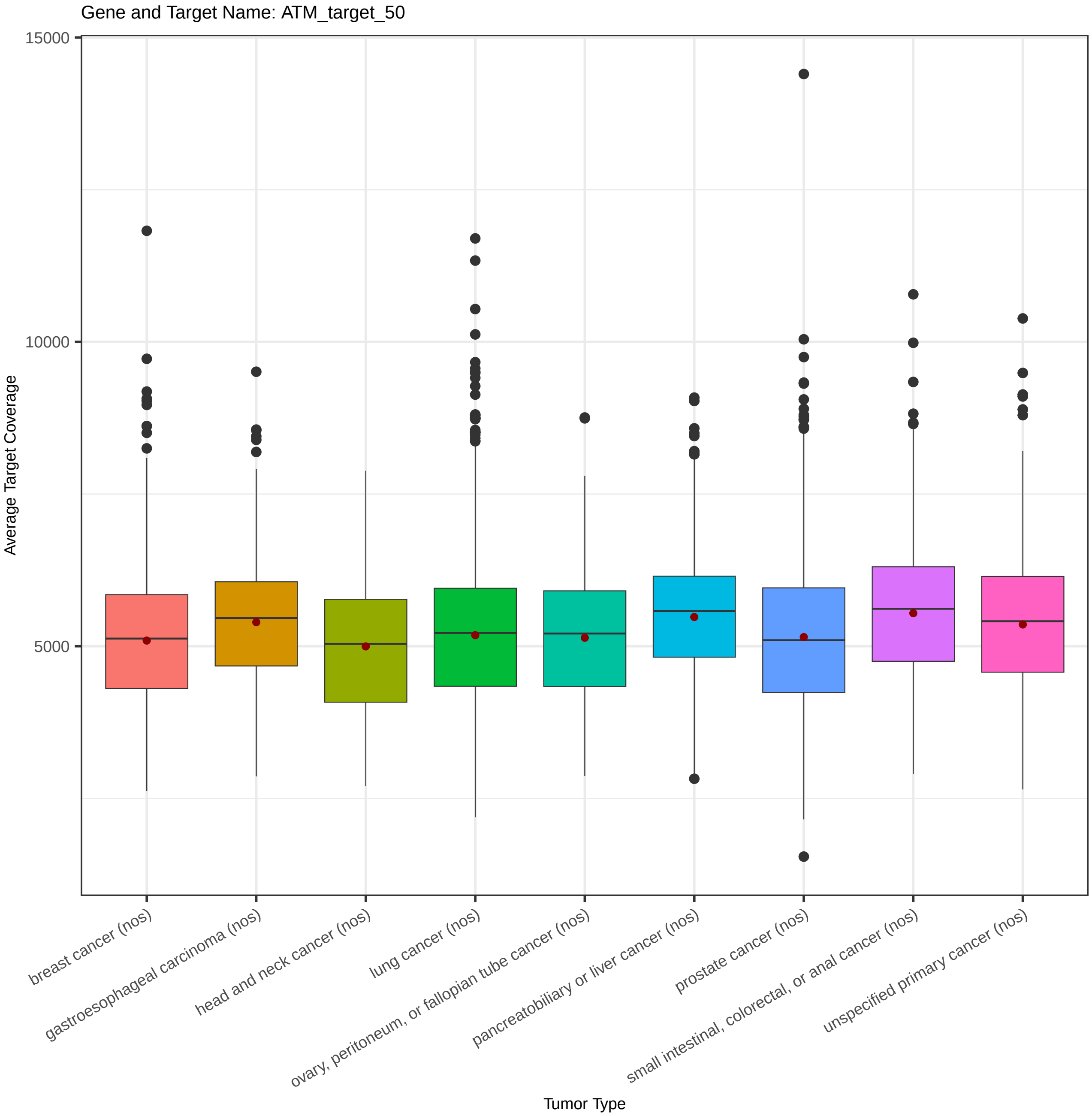

Gene and Target Name: ATM_target_50
15000
10000
5000
Average Target Coverage
breast cancer (nos)
gastroesophageal carcinoma (nos)
head and neck cancer (nos)
lung cancer (nos)
ovary, peritoneum, or fallopian tube cancer (nos)
pancreatobiliary or liver cancer (nos)
prostate cancer (nos)
small intestinal, colorectal, or anal cancer (nos)
unspecified primary cancer (nos)
Tumor Type

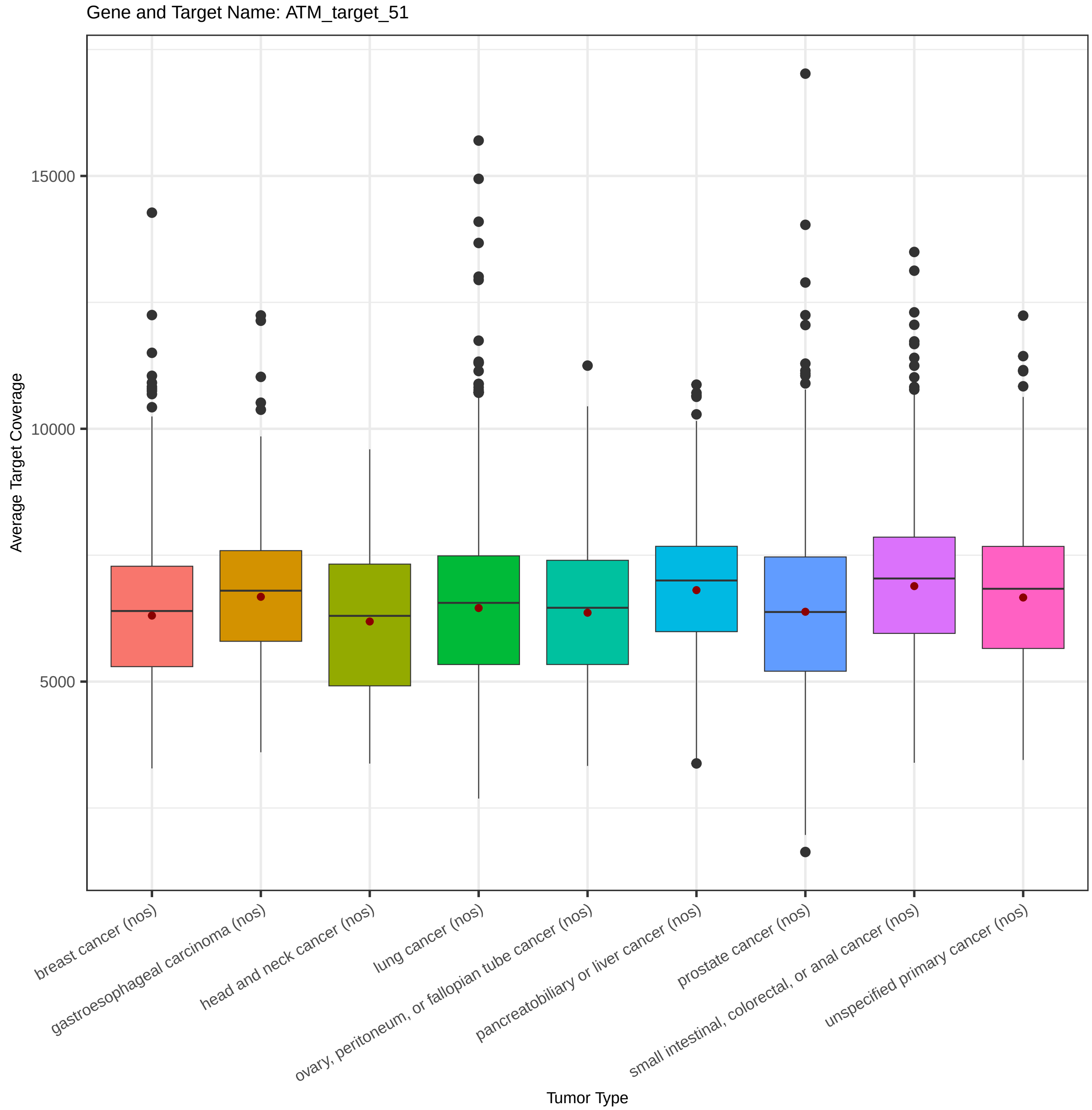

Gene and Target Name: ATM_target_51
Average Target Coverage
15000
10000
5000
breast cancer (nos)
gastroesophageal carcinoma (nos)
head and neck cancer (nos)
lung cancer (nos)
ovary, peritoneum, or fallopian tube cancer (nos)
pancreatobiliary or liver cancer (nos)
prostate cancer (nos)
small intestinal, colorectal, or anal cancer (nos)
unspecified primary cancer (nos)
Tumor Type

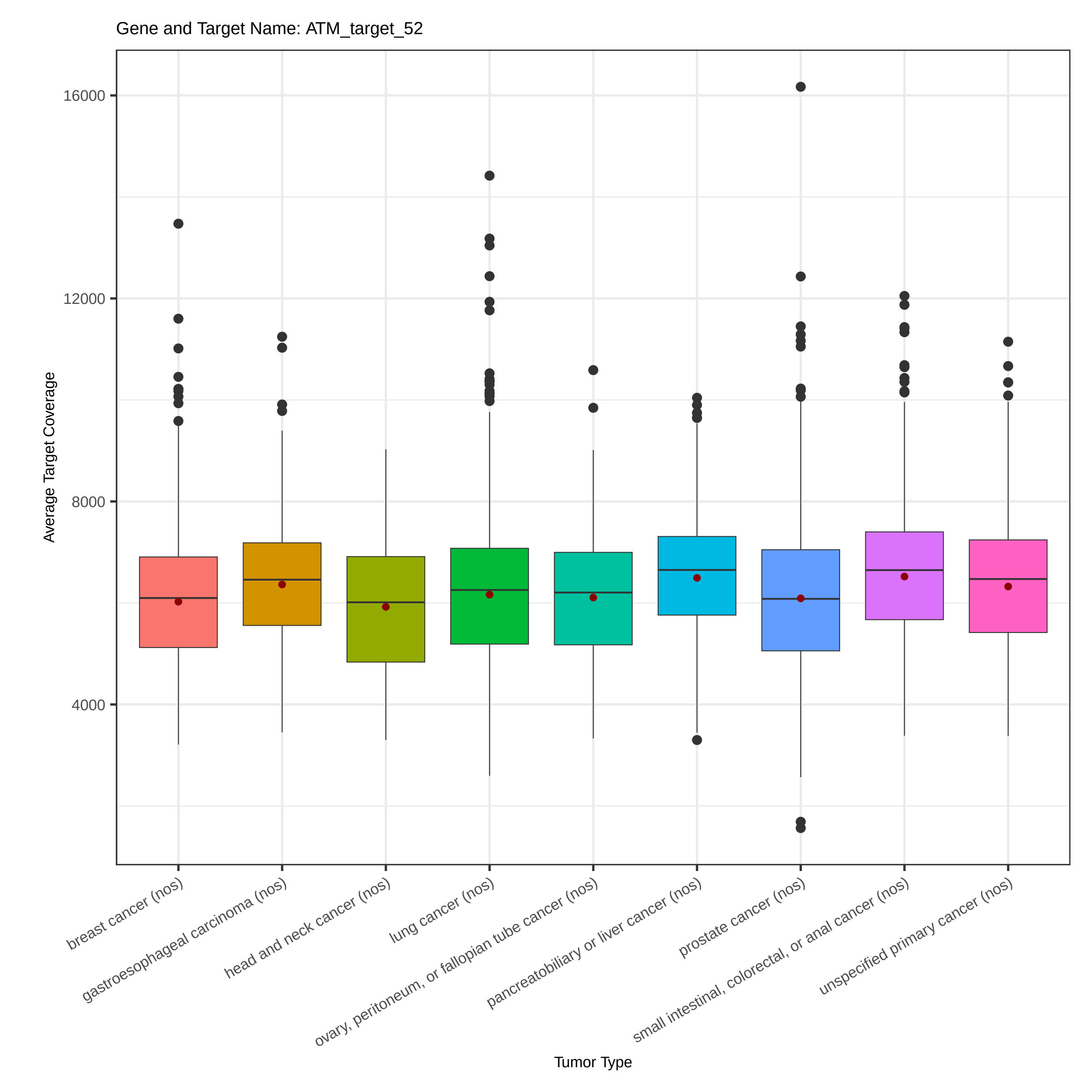

Gene and Target Name: ATM_target_52
Average Target Coverage
16000
12000
8000
4000
breast cancer (nos)
gastroesophageal carcinoma (nos)
head and neck cancer (nos)
lung cancer (nos)
ovary, peritoneum, or fallopian tube cancer (nos)
pancreatobiliary or liver cancer (nos)
prostate cancer (nos)
small intestinal, colorectal, or anal cancer (nos)
unspecified primary cancer (nos)
Tumor Type

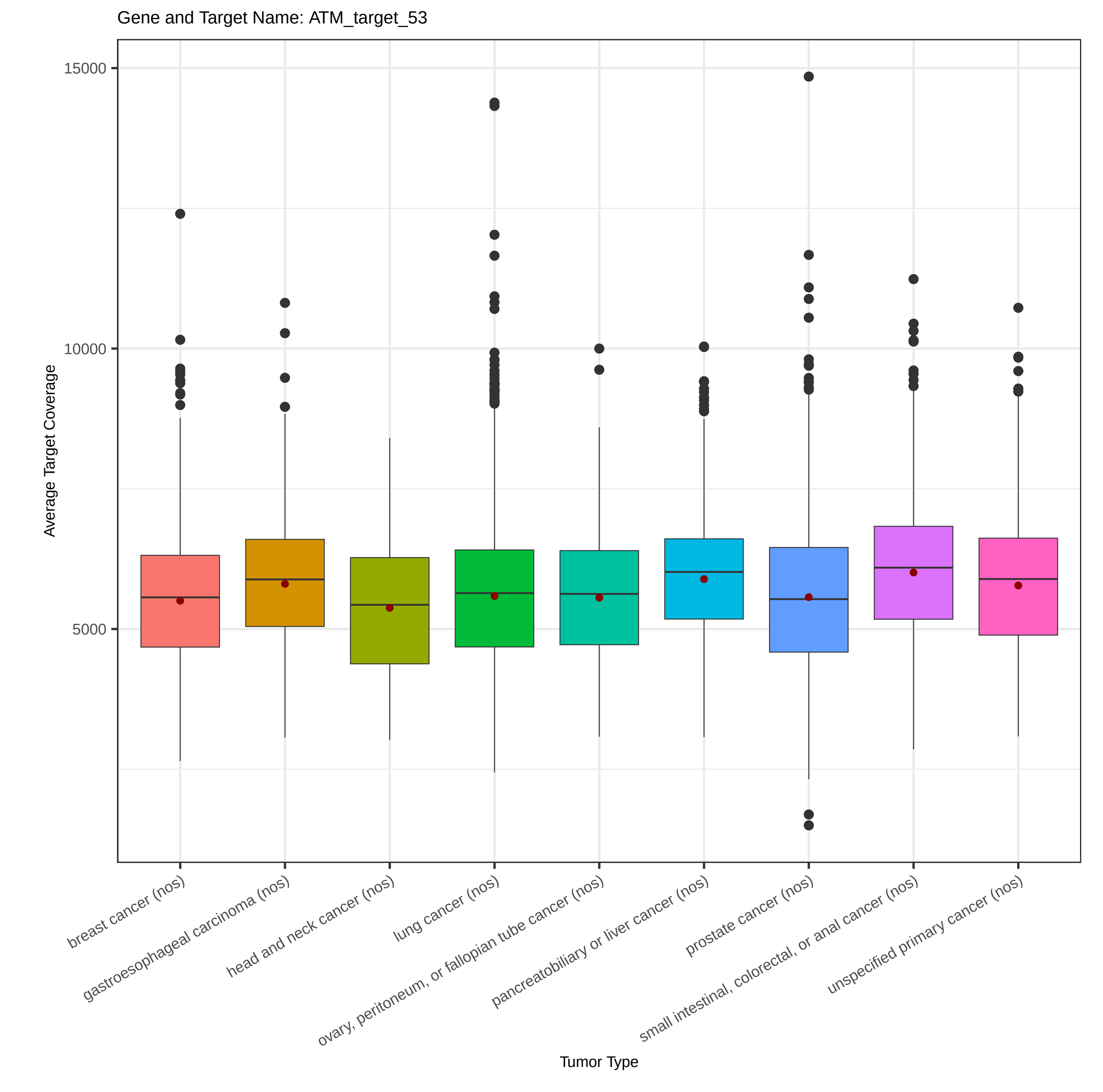
Gene and Target Name: ATM_target_53
Average Target Coverage
15000
10000
5000
Tumor Type
breast cancer (nos)
gastroesophageal carcinoma (nos)
head and neck cancer (nos)
lung cancer (nos)
ovary, peritoneum, or fallopian tube cancer (nos)
pancreatobiliary or liver cancer (nos)
prostate cancer (nos)
small intestinal, colorectal, or anal cancer (nos)
unspecified primary cancer (nos)

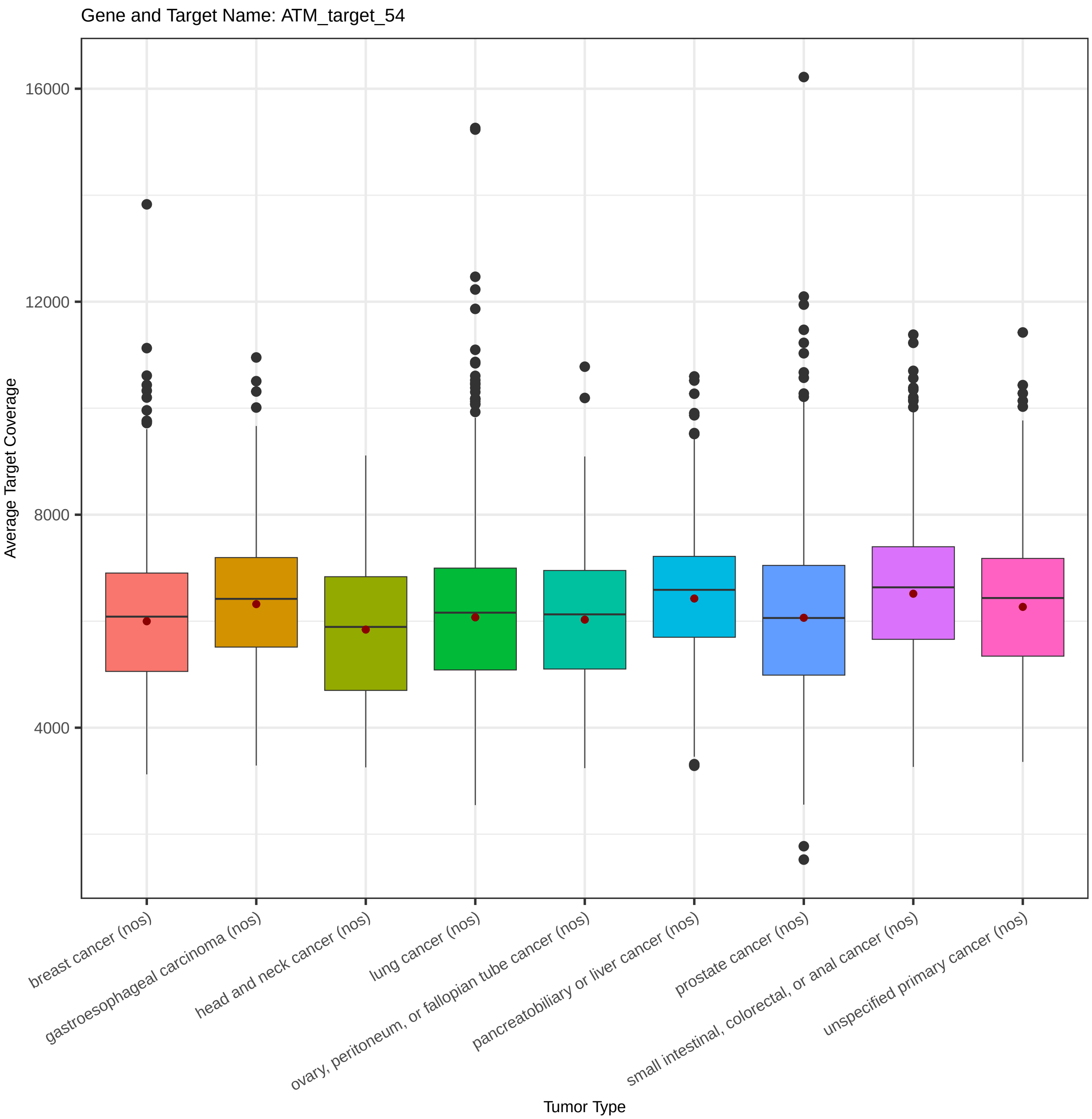
Gene and Target Name: ATM_target_54
Average Target Coverage
16000
12000
8000
4000
breast cancer (nos)
gastroesophageal carcinoma (nos)
head and neck cancer (nos)
lung cancer (nos)
ovary, peritoneum, or fallopian tube cancer (nos)
pancreatobiliary or liver cancer (nos)
prostate cancer (nos)
small intestinal, colorectal, or anal cancer (nos)
unspecified primary cancer (nos)
Tumor Type

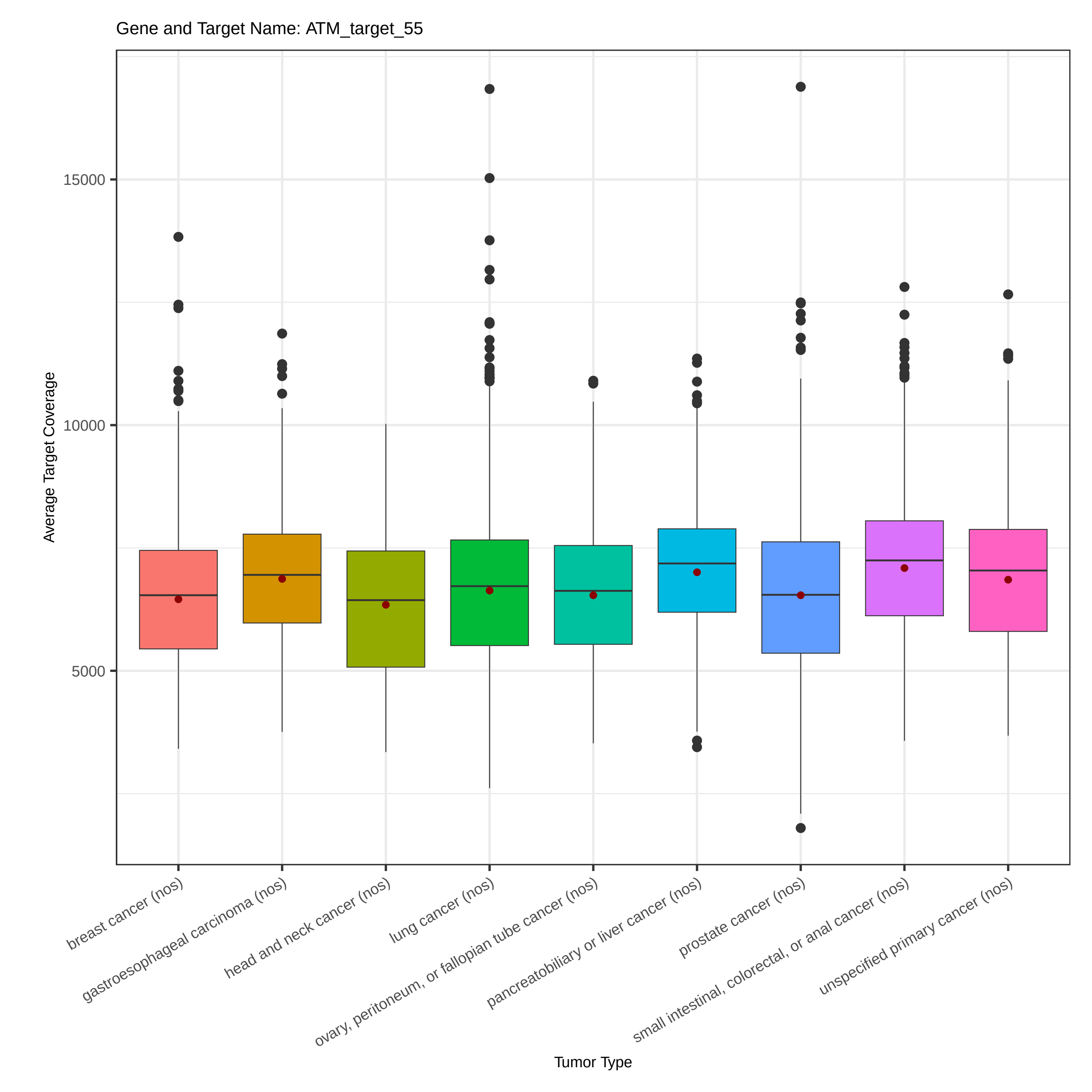

Gene and Target Name: ATM_target_55
Average Target Coverage
15000
10000
5000
breast cancer (nos)
gastroesophageal carcinoma (nos)
head and neck cancer (nos)
lung cancer (nos)
ovary, peritoneum, or fallopian tube cancer (nos)
pancreatobiliary or liver cancer (nos)
prostate cancer (nos)
small intestinal, colorectal, or anal cancer (nos)
unspecified primary cancer (nos)
Tumor Type

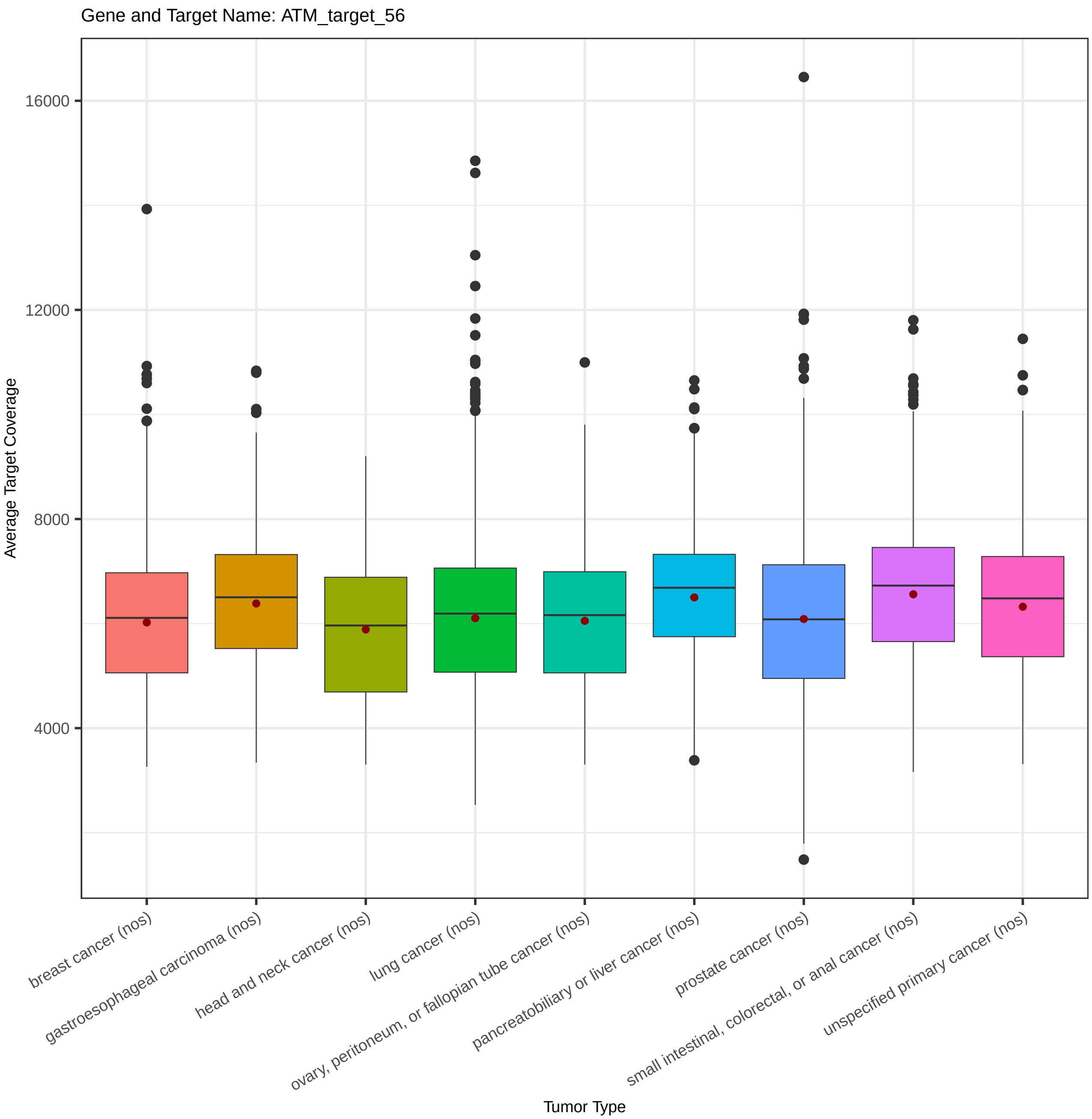

Gene and Target Name: ATM_target_56
16000
12000
8000
4000
Average Target Coverage
breast cancer (nos)
gastroesophageal carcinoma (nos)
head and neck cancer (nos)
lung cancer (nos)
ovary, peritoneum, or fallopian tube cancer (nos)
pancreatobiliary or liver cancer (nos)
prostate cancer (nos)
small intestinal, colorectal, or anal cancer (nos)
unspecified primary cancer (nos)
Tumor Type

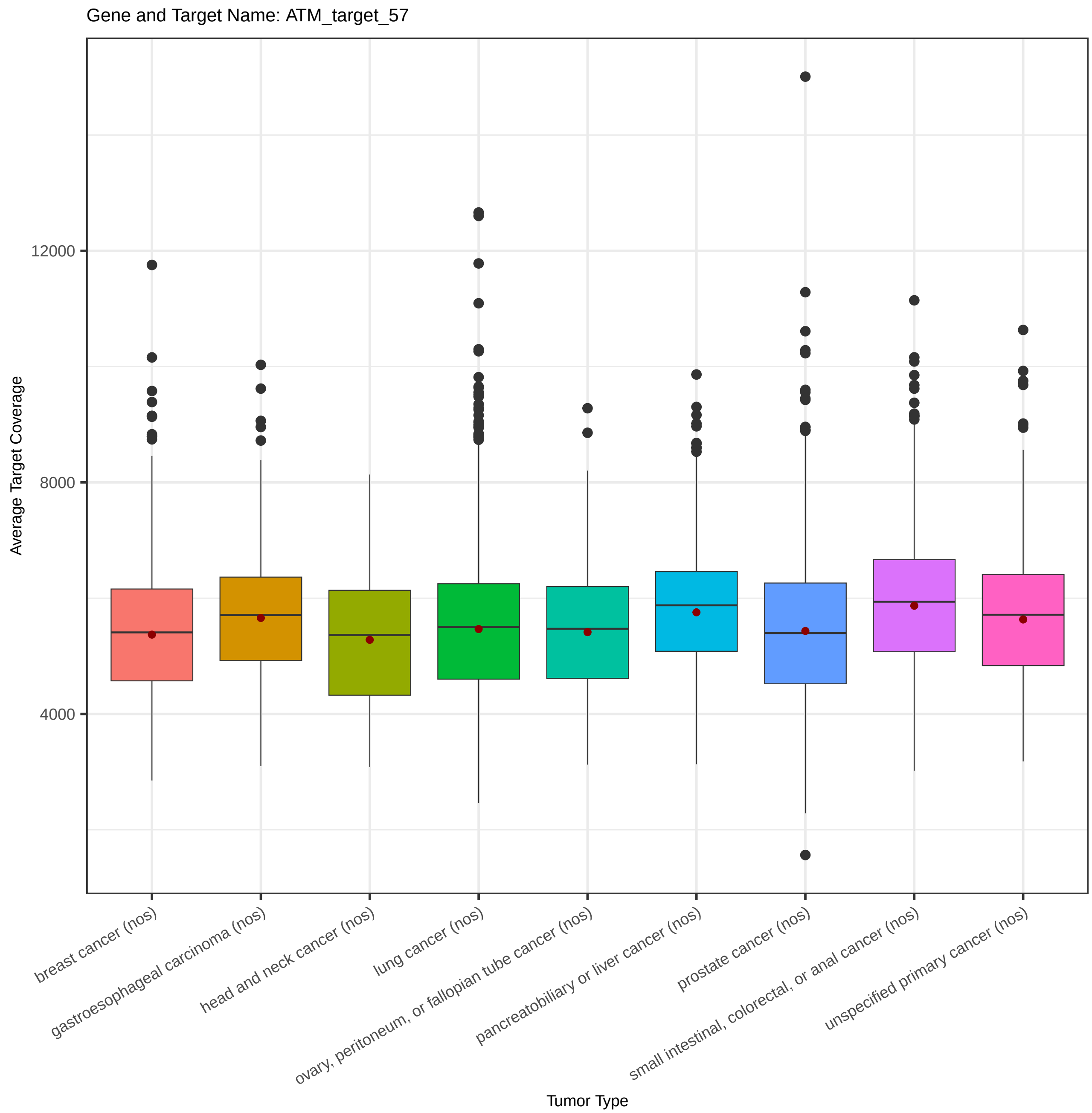
Gene and Target Name: ATM_target_57
Average Target Coverage
12000
8000
4000
breast cancer (nos)
gastroesophageal carcinoma (nos)
head and neck cancer (nos)
lung cancer (nos)
ovary, peritoneum, or fallopian tube cancer (nos)
pancreatobiliary or liver cancer (nos)
prostate cancer (nos)
small intestinal, colorectal, or anal cancer (nos)
unspecified primary cancer (nos)
Tumor Type

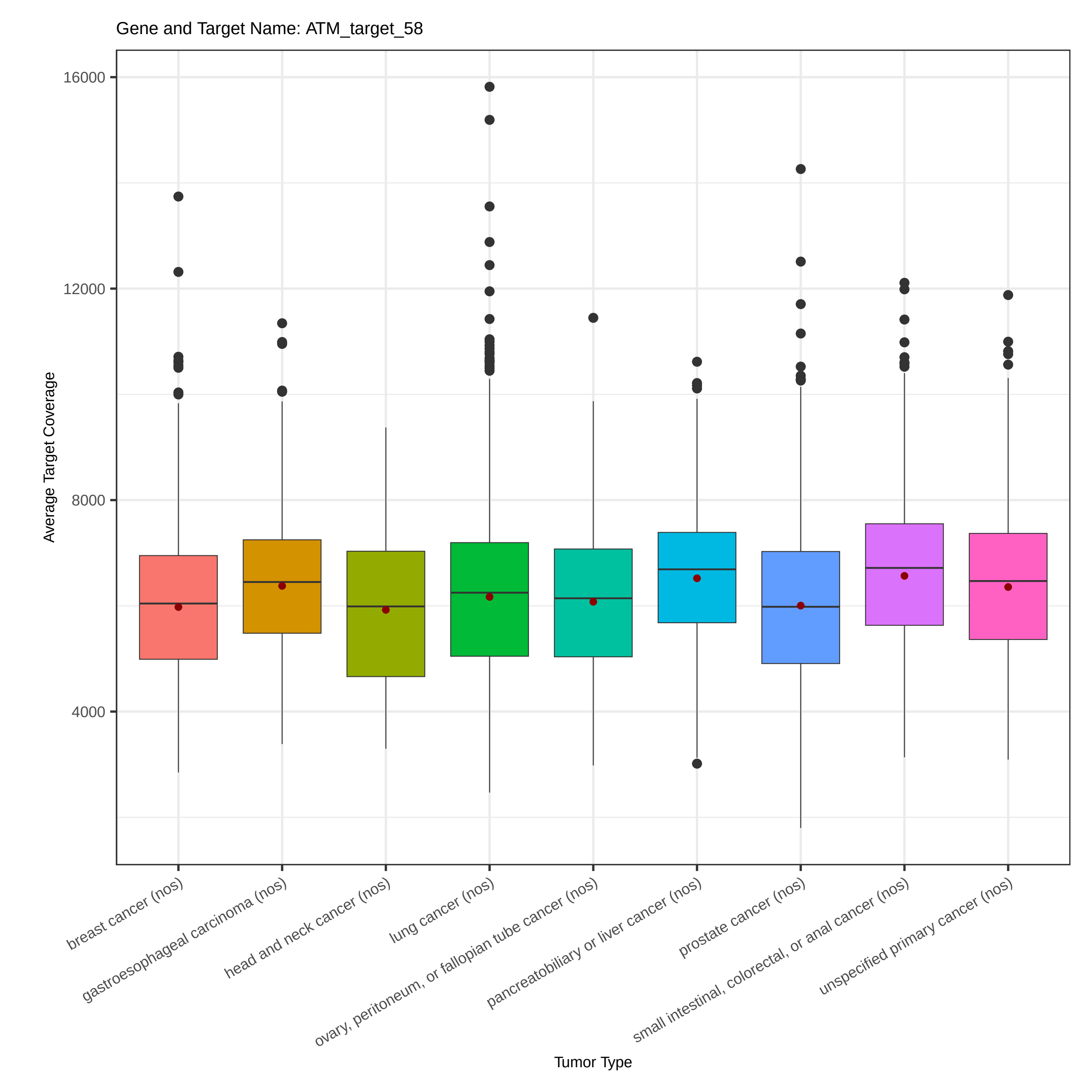
Gene and Target Name: ATM_target_58
Average Target Coverage
16000
12000
8000
4000
breast cancer (nos)
gastroesophageal carcinoma (nos)
head and neck cancer (nos)
lung cancer (nos)
ovary, peritoneum, or fallopian tube cancer (nos)
pancreatobiliary or liver cancer (nos)
prostate cancer (nos)
small intestinal, colorectal, or anal cancer (nos)
unspecified primary cancer (nos)
Tumor Type

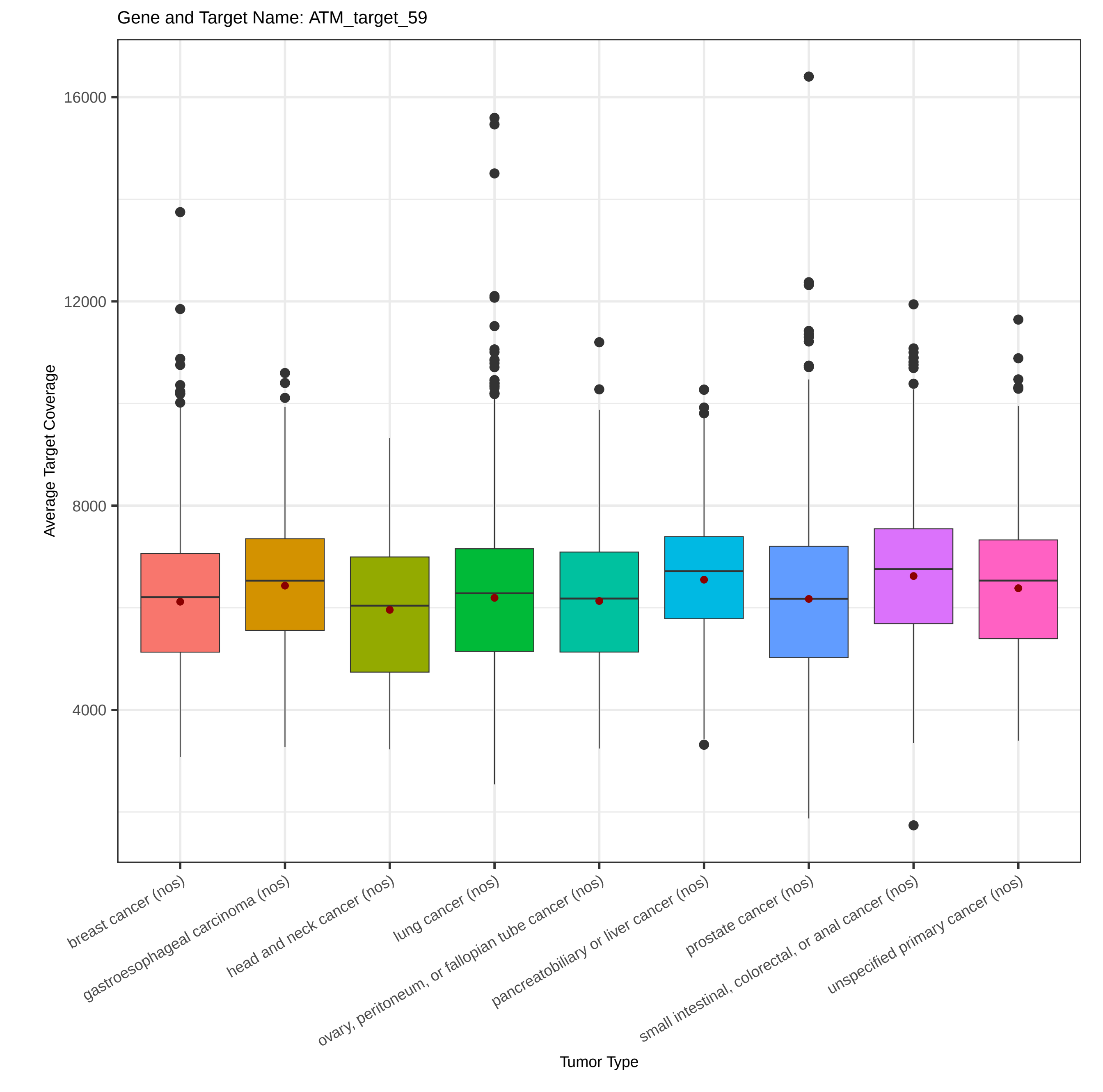
Gene and Target Name: ATM_target_59
Average Target Coverage
16000
12000
8000
4000
Tumor Type
breast cancer (nos)
gastroesophageal carcinoma (nos)
head and neck cancer (nos)
lung cancer (nos)
ovary, peritoneum, or fallopian tube cancer (nos)
pancreatobiliary or liver cancer (nos)
prostate cancer (nos)
small intestinal, colorectal, or anal cancer (nos)
unspecified primary cancer (nos)

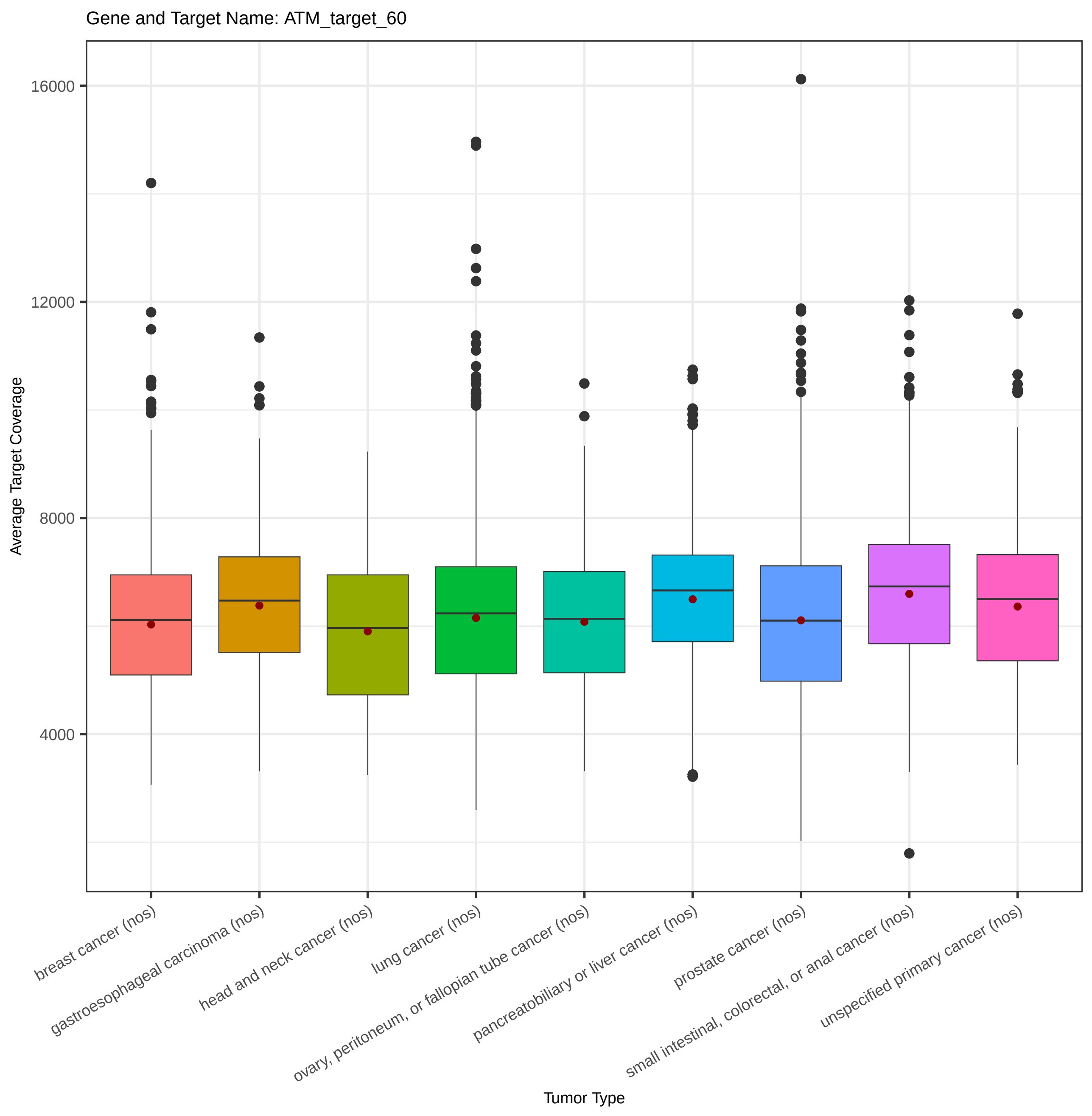

Gene and Target Name: ATM_target_60
16000
12000
8000
4000
Average Target Coverage
breast cancer (nos)
gastroesophageal carcinoma (nos)
head and neck cancer (nos)
lung cancer (nos)
ovary, peritoneum, or fallopian tube cancer (nos)
pancreatobiliary or liver cancer (nos)
prostate cancer (nos)
small intestinal, colorectal, or anal cancer (nos)
unspecified primary cancer (nos)
Tumor Type

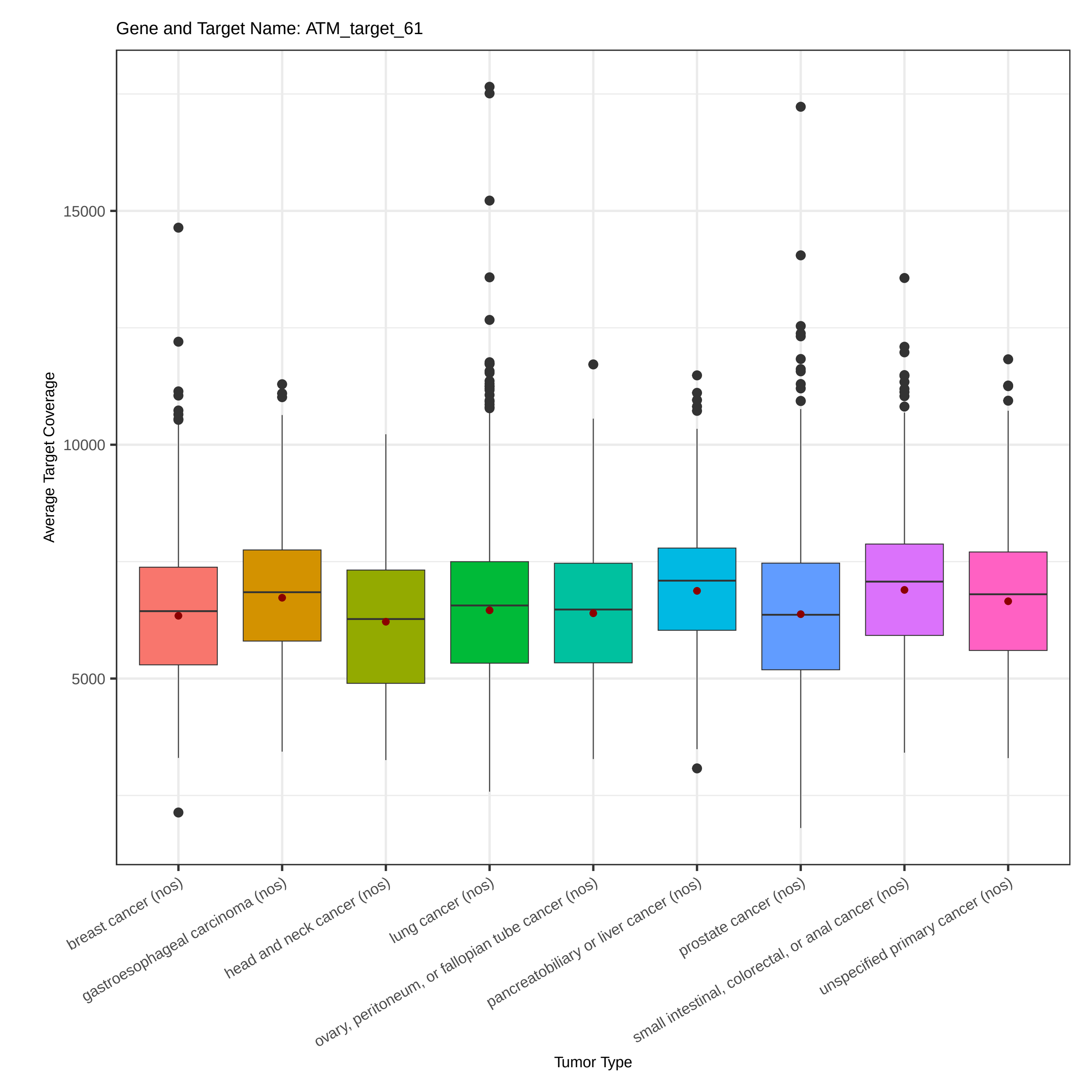

Gene and Target Name: ATM_target_61
Average Target Coverage
15000
10000
5000
breast cancer (nos)
gastroesophageal carcinoma (nos)
head and neck cancer (nos)
lung cancer (nos)
ovary, peritoneum, or fallopian tube cancer (nos)
pancreatobiliary or liver cancer (nos)
prostate cancer (nos)
small intestinal, colorectal, or anal cancer (nos)
unspecified primary cancer (nos)
Tumor Type

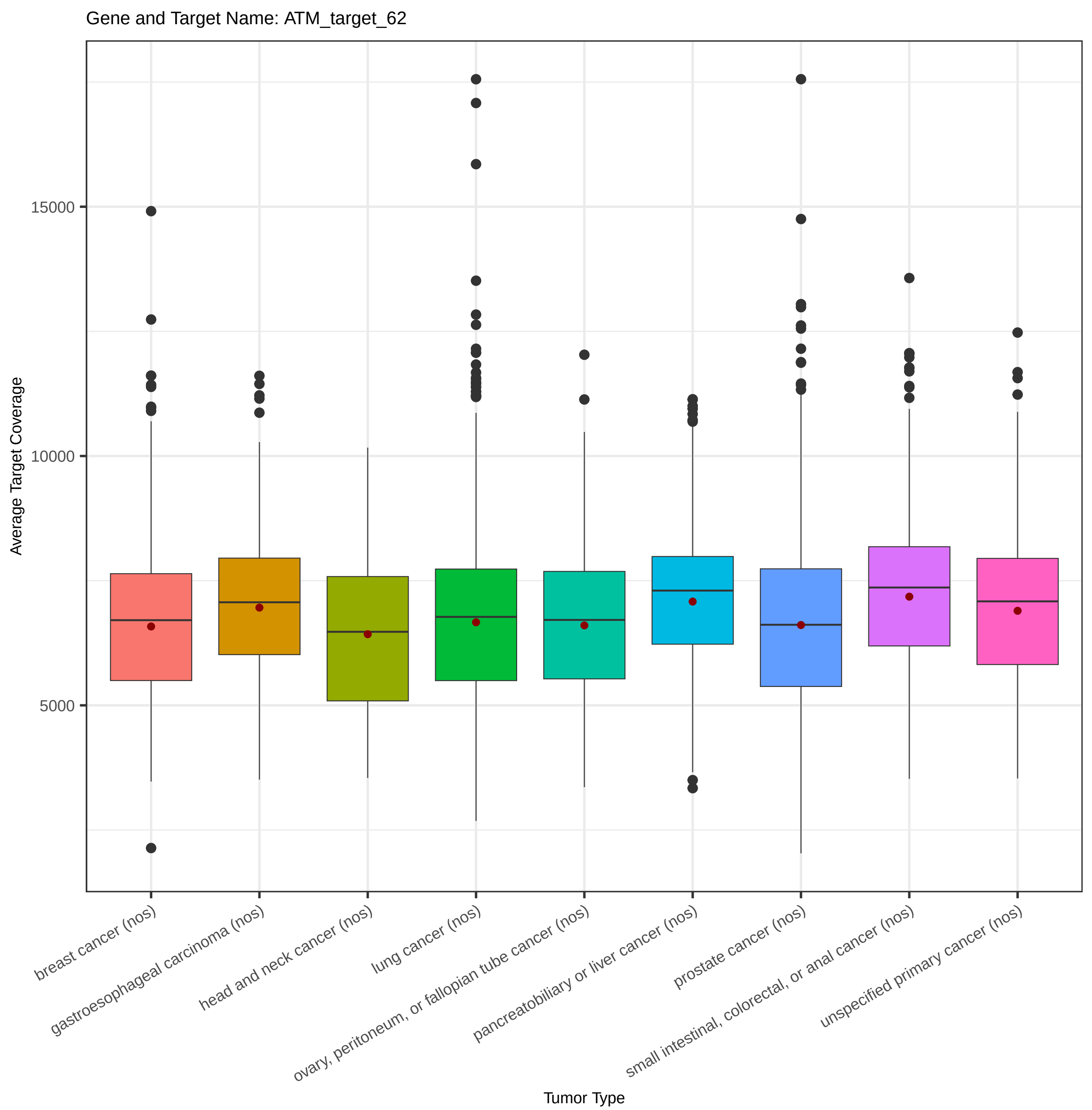

Gene and Target Name: ATM_target_62
Average Target Coverage
15000
10000
5000
Tumor Type
breast cancer (nos)
gastroesophageal carcinoma (nos)
head and neck cancer (nos)
lung cancer (nos)
ovary, peritoneum, or fallopian tube cancer (nos)
pancreatobiliary or liver cancer (nos)
prostate cancer (nos)
small intestinal, colorectal, or anal cancer (nos)
unspecified primary cancer (nos)

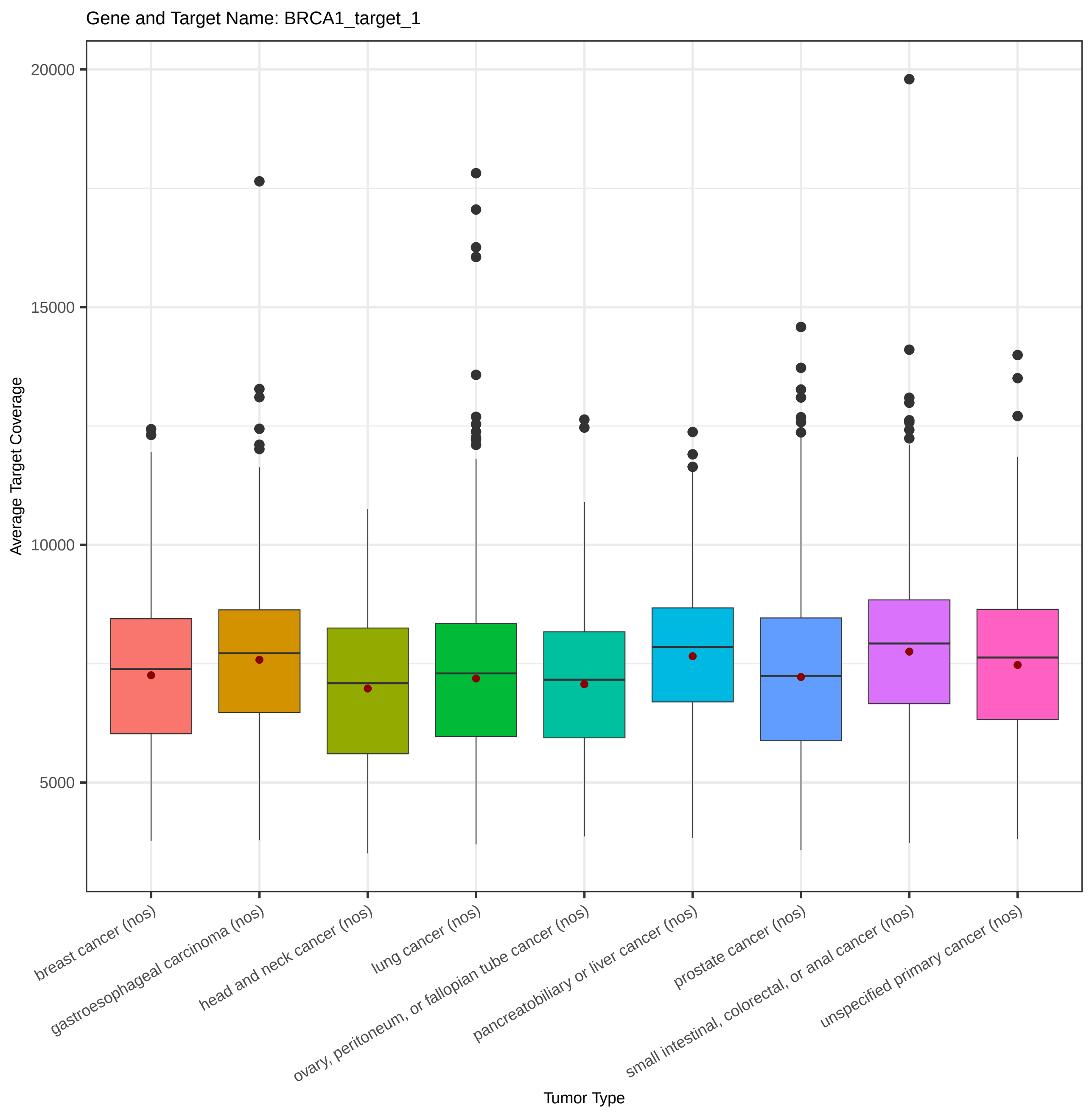

Gene and Target Name: BRCA1_target_1
Average Target Coverage
20000
15000
10000
5000
breast cancer (nos)
gastroesophageal carcinoma (nos)
head and neck cancer (nos)
lung cancer (nos)
ovary, peritoneum, or fallopian tube cancer (nos)
pancreatobiliary or liver cancer (nos)
prostate cancer (nos)
small intestinal, colorectal, or anal cancer (nos)
unspecified primary cancer (nos)
Tumor Type

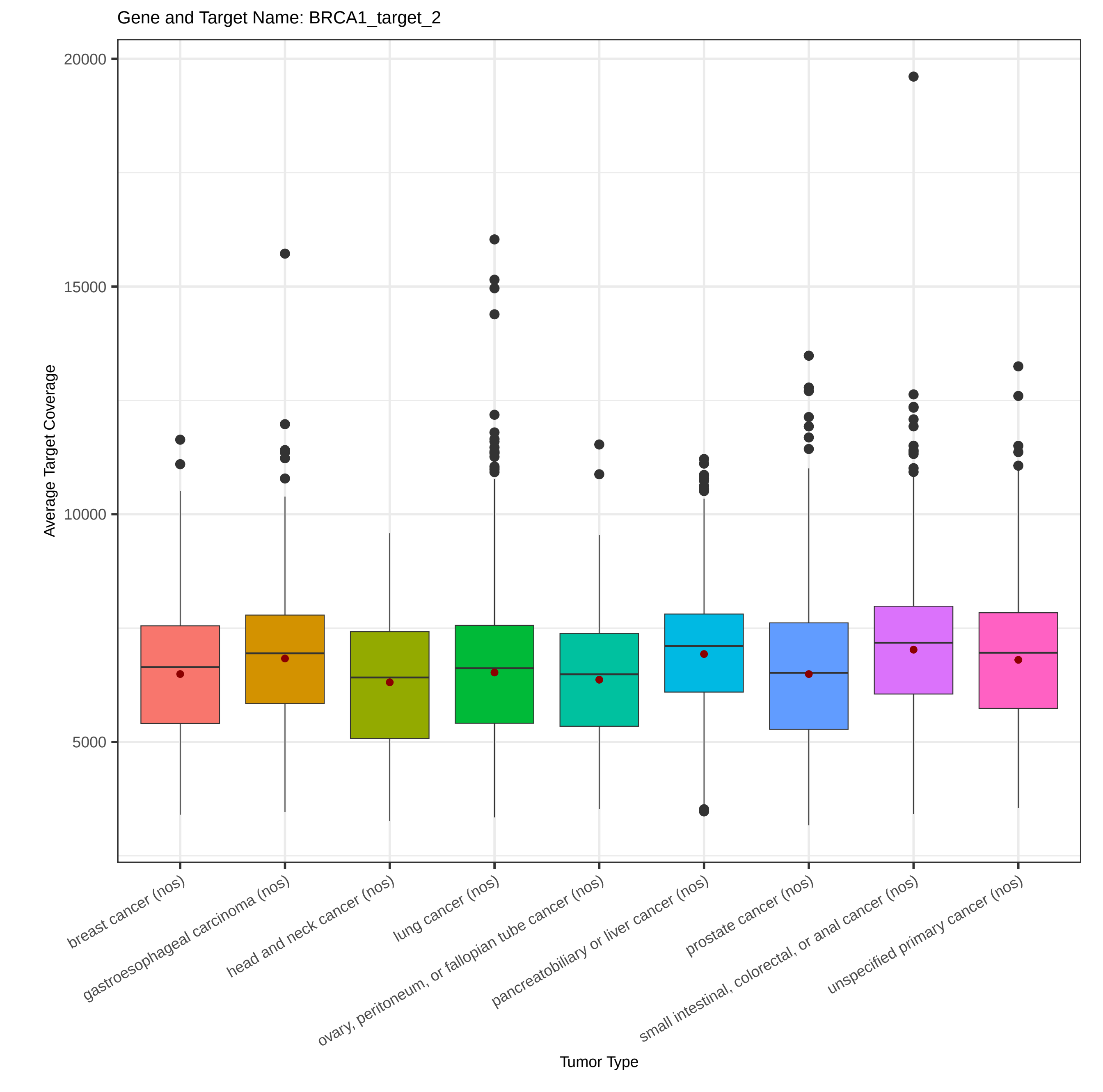

Gene and Target Name: BRCA1_target_2
20000
15000
10000
5000
Average Target Coverage
breast cancer (nos)
gastroesophageal carcinoma (nos)
head and neck cancer (nos)
lung cancer (nos)
ovary, peritoneum, or fallopian tube cancer (nos)
pancreatobiliary or liver cancer (nos)
prostate cancer (nos)
small intestinal, colorectal, or anal cancer (nos)
unspecified primary cancer (nos)
Tumor Type

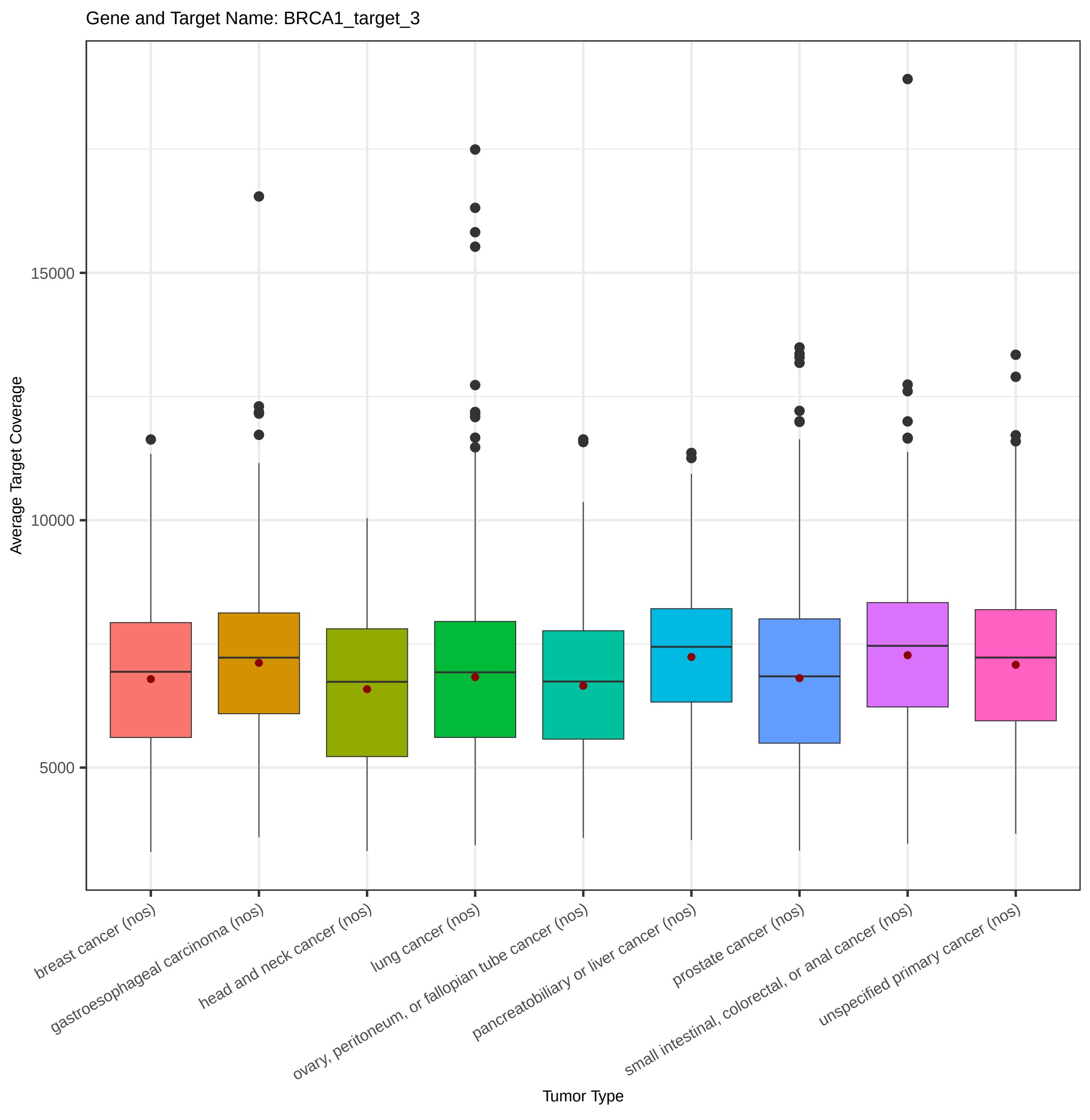

Gene and Target Name: BRCA1_target_3
Average Target Coverage
15000
10000
5000
breast cancer (nos)
gastroesophageal carcinoma (nos)
head and neck cancer (nos)
lung cancer (nos)
ovary, peritoneum, or fallopian tube cancer (nos)
pancreatobiliary or liver cancer (nos)
prostate cancer (nos)
small intestinal, colorectal, or anal cancer (nos)
unspecified primary cancer (nos)
Tumor Type

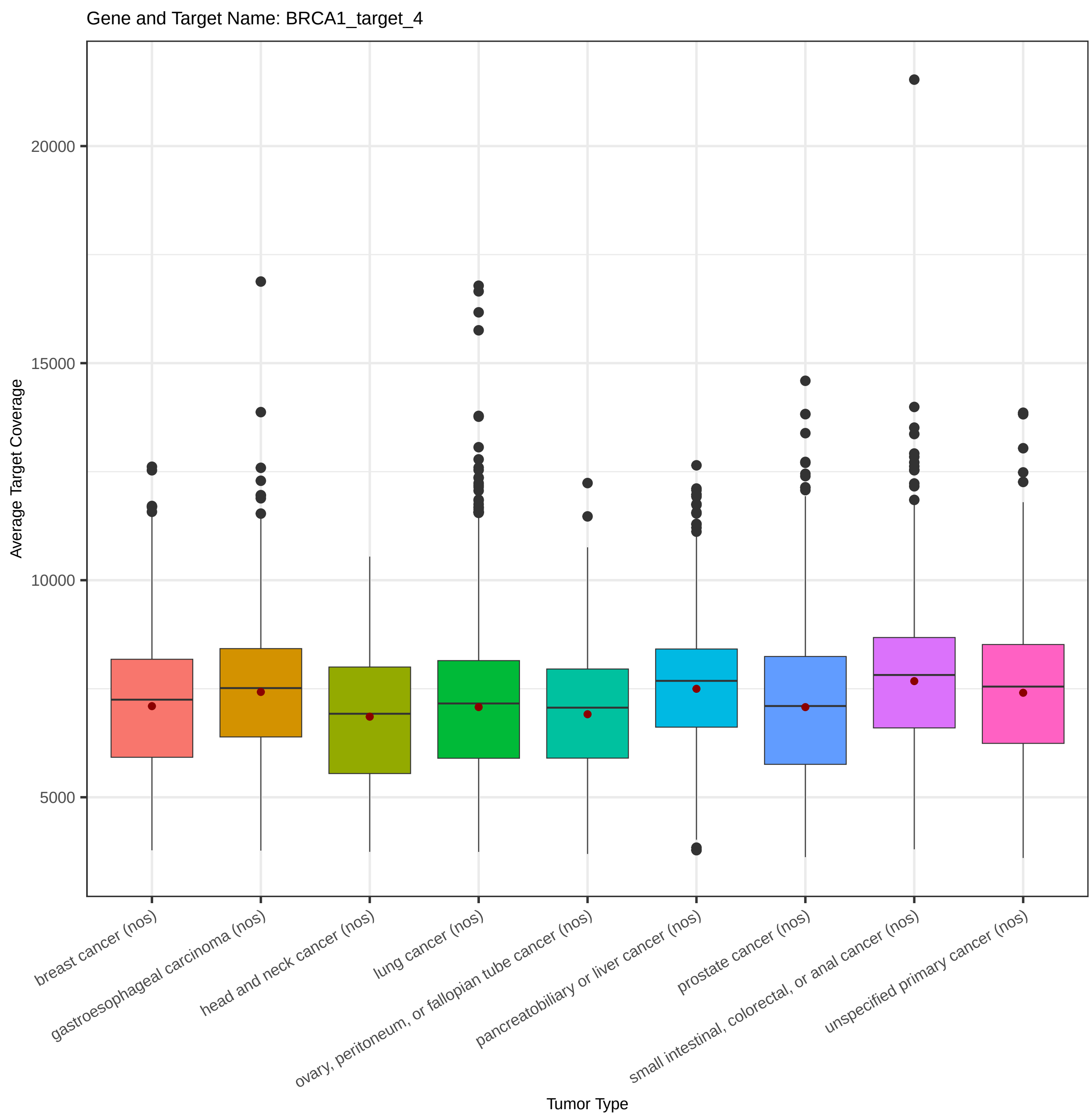
Gene and Target Name: BRCA1_target_4
Average Target Coverage
20000
15000
10000
5000
breast cancer (nos)
gastroesophageal carcinoma (nos)
head and neck cancer (nos)
lung cancer (nos)
ovary, peritoneum, or fallopian tube cancer (nos)
pancreatobiliary or liver cancer (nos)
prostate cancer (nos)
small intestinal, colorectal, or anal cancer (nos)
unspecified primary cancer (nos)
Tumor Type

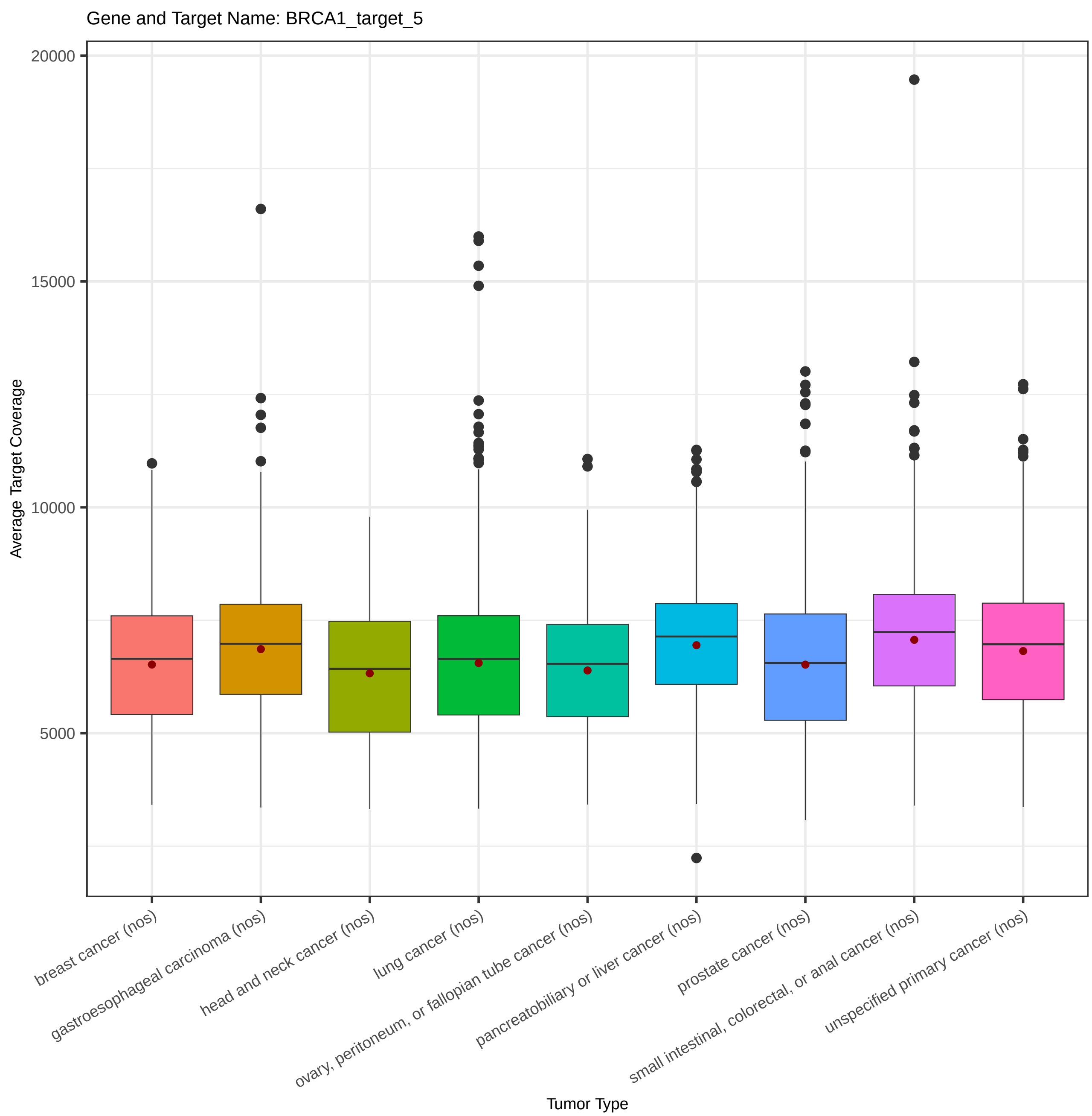
Gene and Target Name: BRCA1_target_5
Average Target Coverage
20000
15000
10000
5000
breast cancer (nos)
gastroesophageal carcinoma (nos)
head and neck cancer (nos)
lung cancer (nos)
ovary, peritoneum, or fallopian tube cancer (nos)
pancreatobiliary or liver cancer (nos)
prostate cancer (nos)
small intestinal, colorectal, or anal cancer (nos)
unspecified primary cancer (nos)
Tumor Type

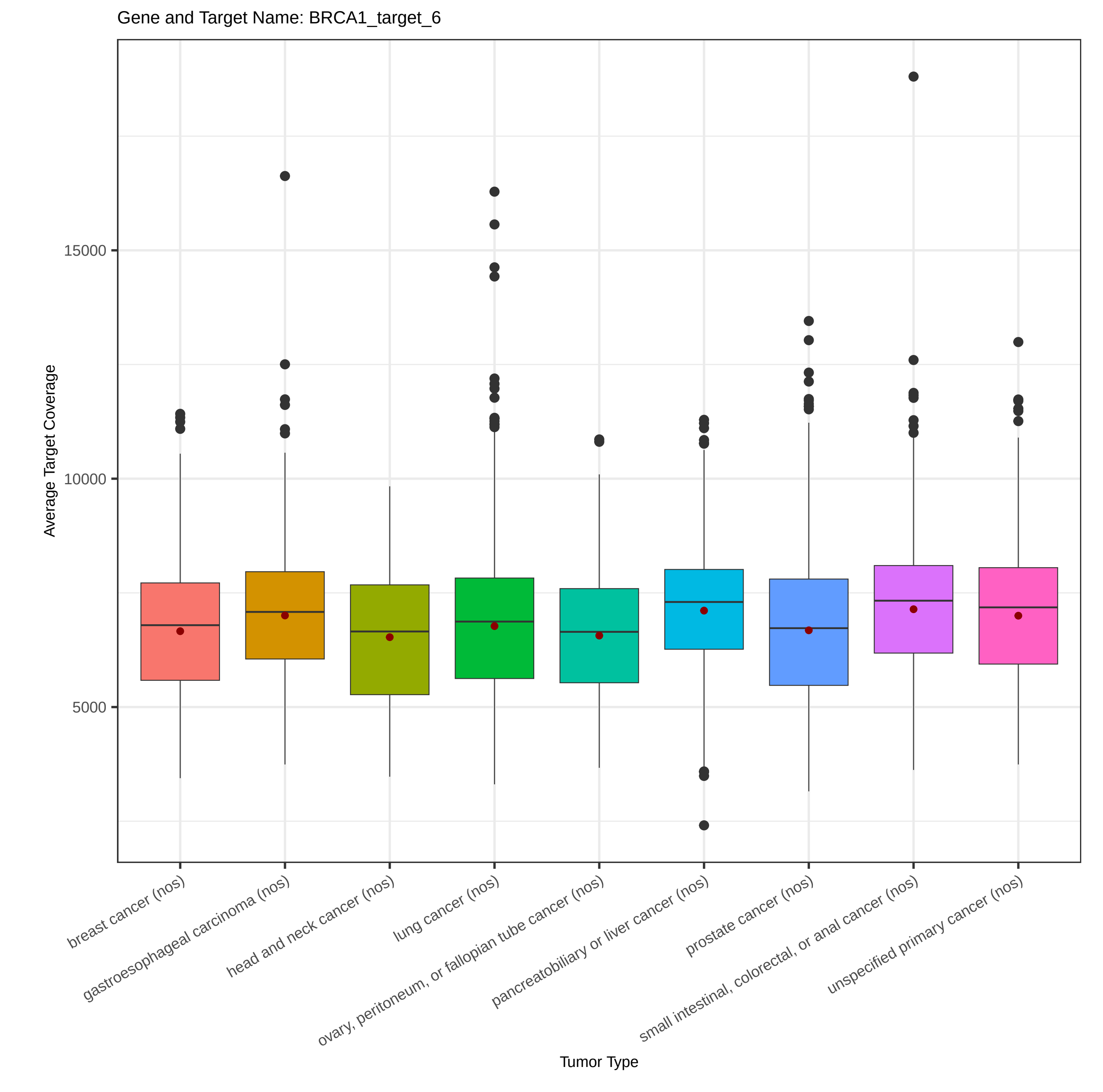
Gene and Target Name: BRCA1_target_6
Average Target Coverage
15000
10000
5000
breast cancer (nos)
gastroesophageal carcinoma (nos)
head and neck cancer (nos)
lung cancer (nos)
ovary, peritoneum, or fallopian tube cancer (nos)
pancreatobiliary or liver cancer (nos)
prostate cancer (nos)
small intestinal, colorectal, or anal cancer (nos)
unspecified primary cancer (nos)
Tumor Type

Gene and Target Name: BRCA1_target_7

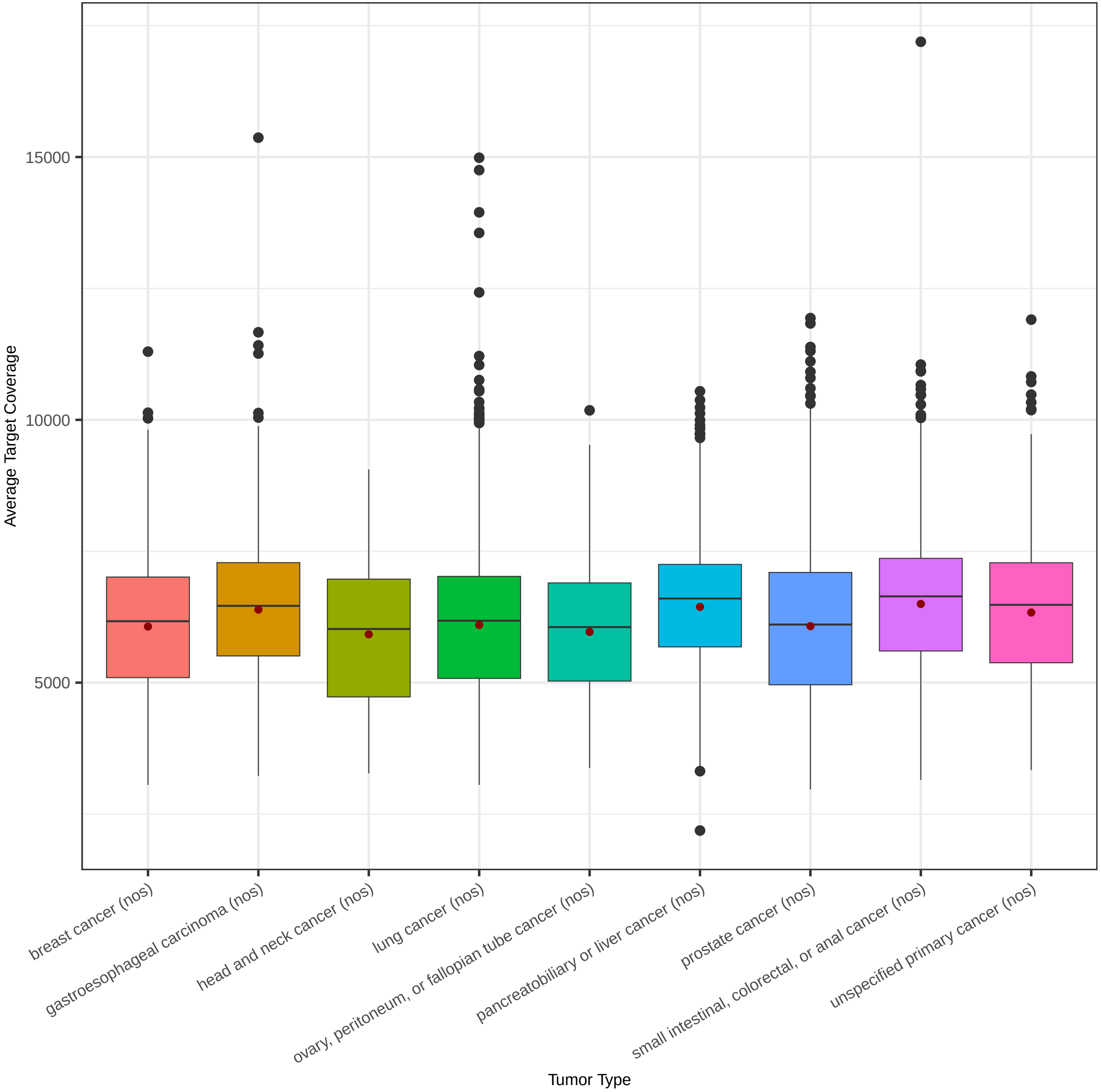

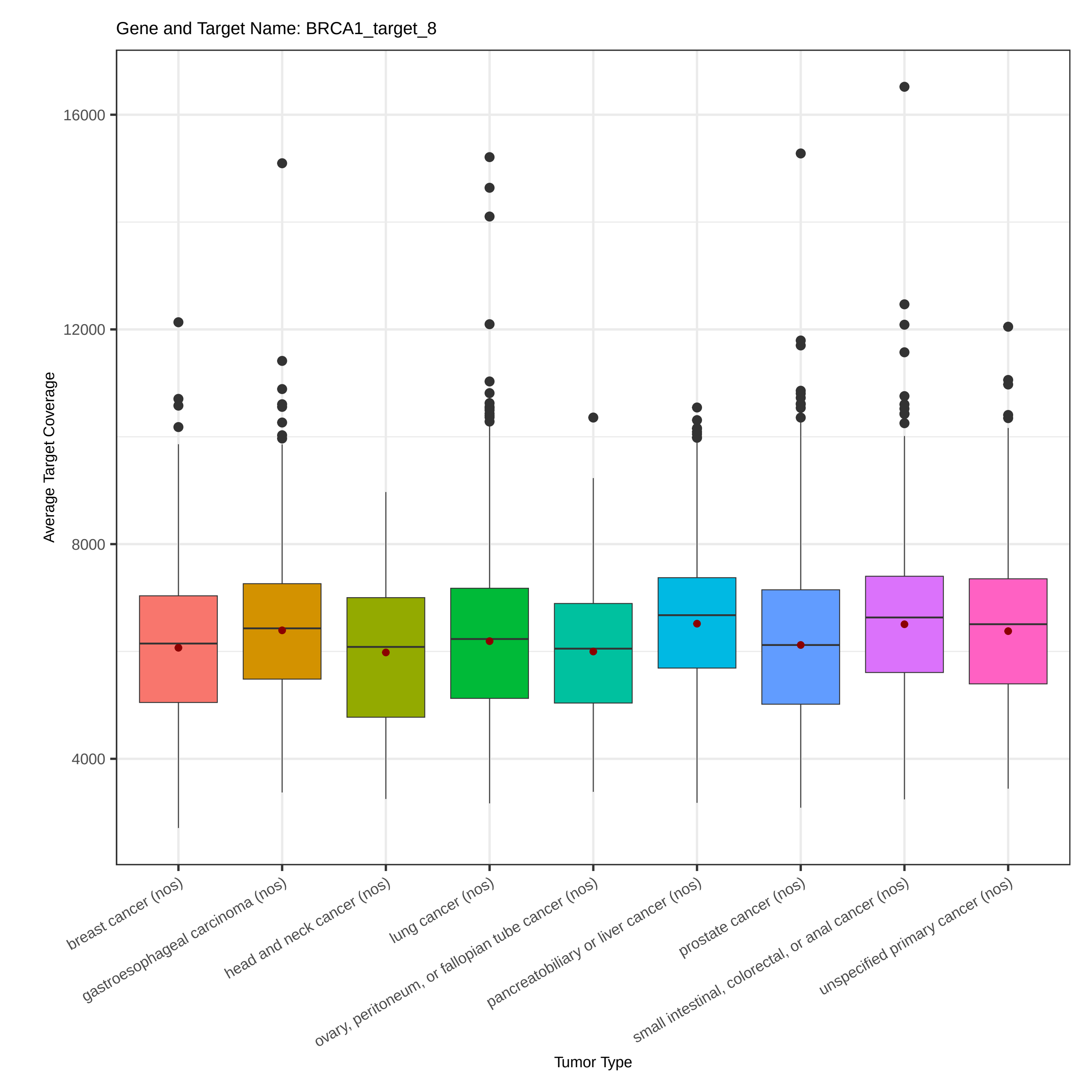
Gene and Target Name: BRCA1_target_8
16000
12000
8000
4000
Average Target Coverage
breast cancer (nos)
gastroesophageal carcinoma (nos)
head and neck cancer (nos)
lung cancer (nos)
ovary, peritoneum, or fallopian tube cancer (nos)
pancreatobiliary or liver cancer (nos)
prostate cancer (nos)
small intestinal, colorectal, or anal cancer (nos)
unspecified primary cancer (nos)
Tumor Type

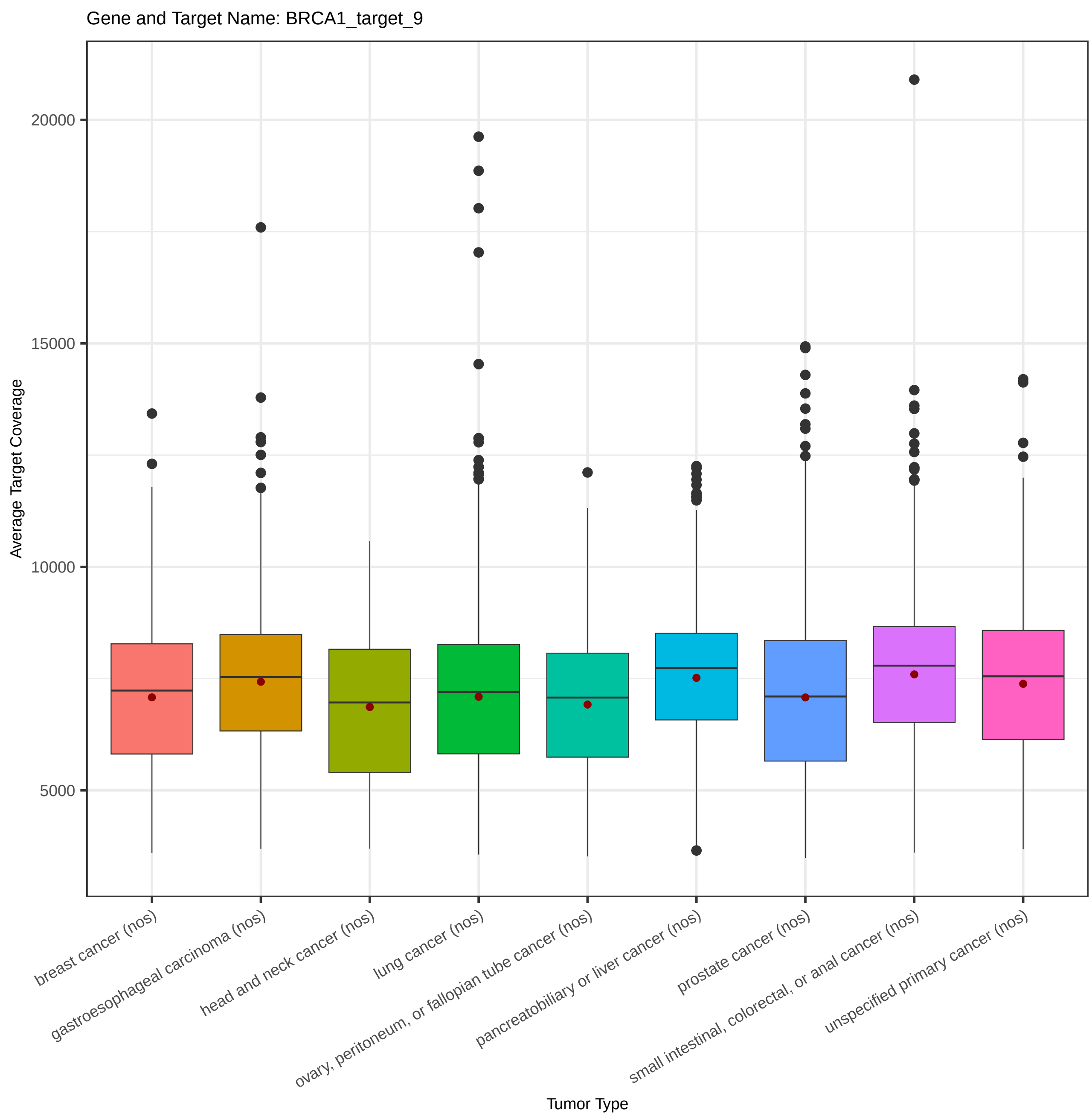

Gene and Target Name: BRCA1_target_9
Average Target Coverage
20000
15000
10000
5000
breast cancer (nos)
gastroesophageal carcinoma (nos)
head and neck cancer (nos)
lung cancer (nos)
ovary, peritoneum, or fallopian tube cancer (nos)
pancreatobiliary or liver cancer (nos)
prostate cancer (nos)
small intestinal, colorectal, or anal cancer (nos)
unspecified primary cancer (nos)
Tumor Type

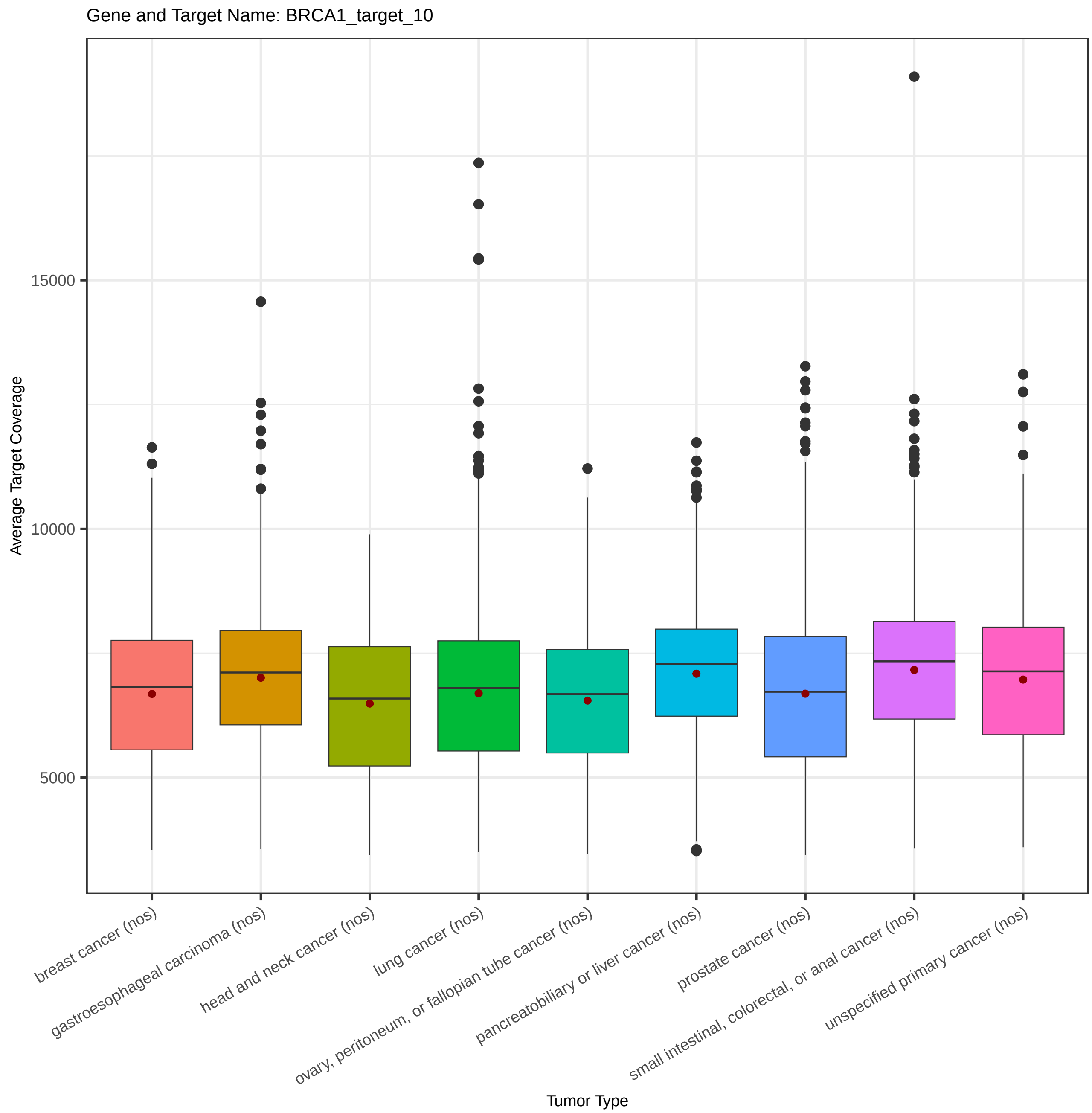
Gene and Target Name: BRCA1_target_10
Average Target Coverage
15000
10000
5000
Tumor Type
breast cancer (nos)
gastroesophageal carcinoma (nos)
head and neck cancer (nos)
lung cancer (nos)
ovary, peritoneum, or fallopian tube cancer (nos)
pancreatobiliary or liver cancer (nos)
prostate cancer (nos)
small intestinal, colorectal, or anal cancer (nos)
unspecified primary cancer (nos)

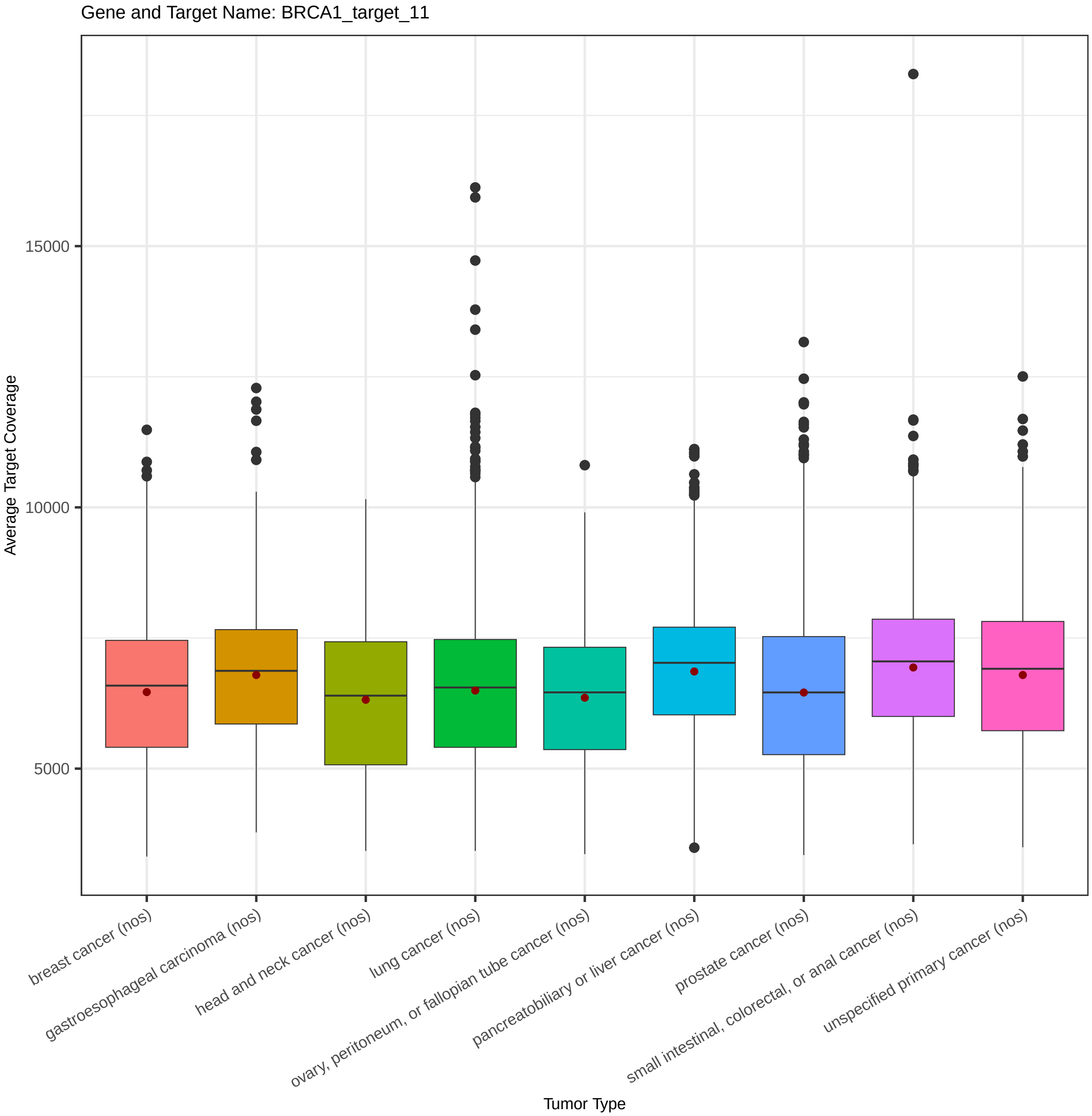
Gene and Target Name: BRCA1_target_11
Average Target Coverage
15000
10000
5000
breast cancer (nos)
gastroesophageal carcinoma (nos)
head and neck cancer (nos)
lung cancer (nos)
ovary, peritoneum, or fallopian tube cancer (nos)
pancreatobiliary or liver cancer (nos)
prostate cancer (nos)
small intestinal, colorectal, or anal cancer (nos)
unspecified primary cancer (nos)
Tumor Type

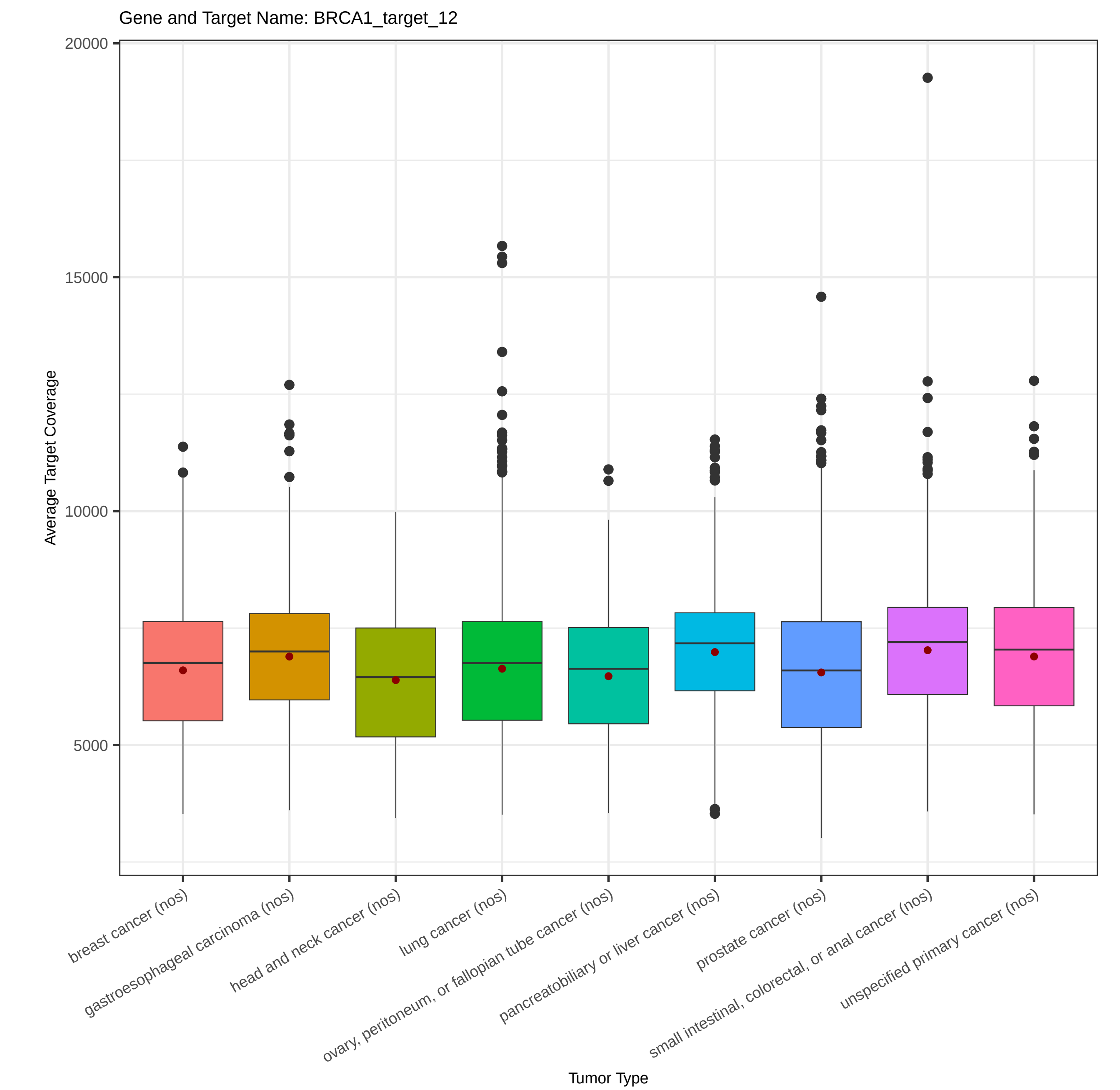
Gene and Target Name: BRCA1_target_12
Average Target Coverage
20000
15000
10000
5000
breast cancer (nos)
gastroesophageal carcinoma (nos)
head and neck cancer (nos)
lung cancer (nos)
ovary, peritoneum, or fallopian tube cancer (nos)
pancreatobiliary or liver cancer (nos)
prostate cancer (nos)
small intestinal, colorectal, or anal cancer (nos)
unspecified primary cancer (nos)
Tumor Type

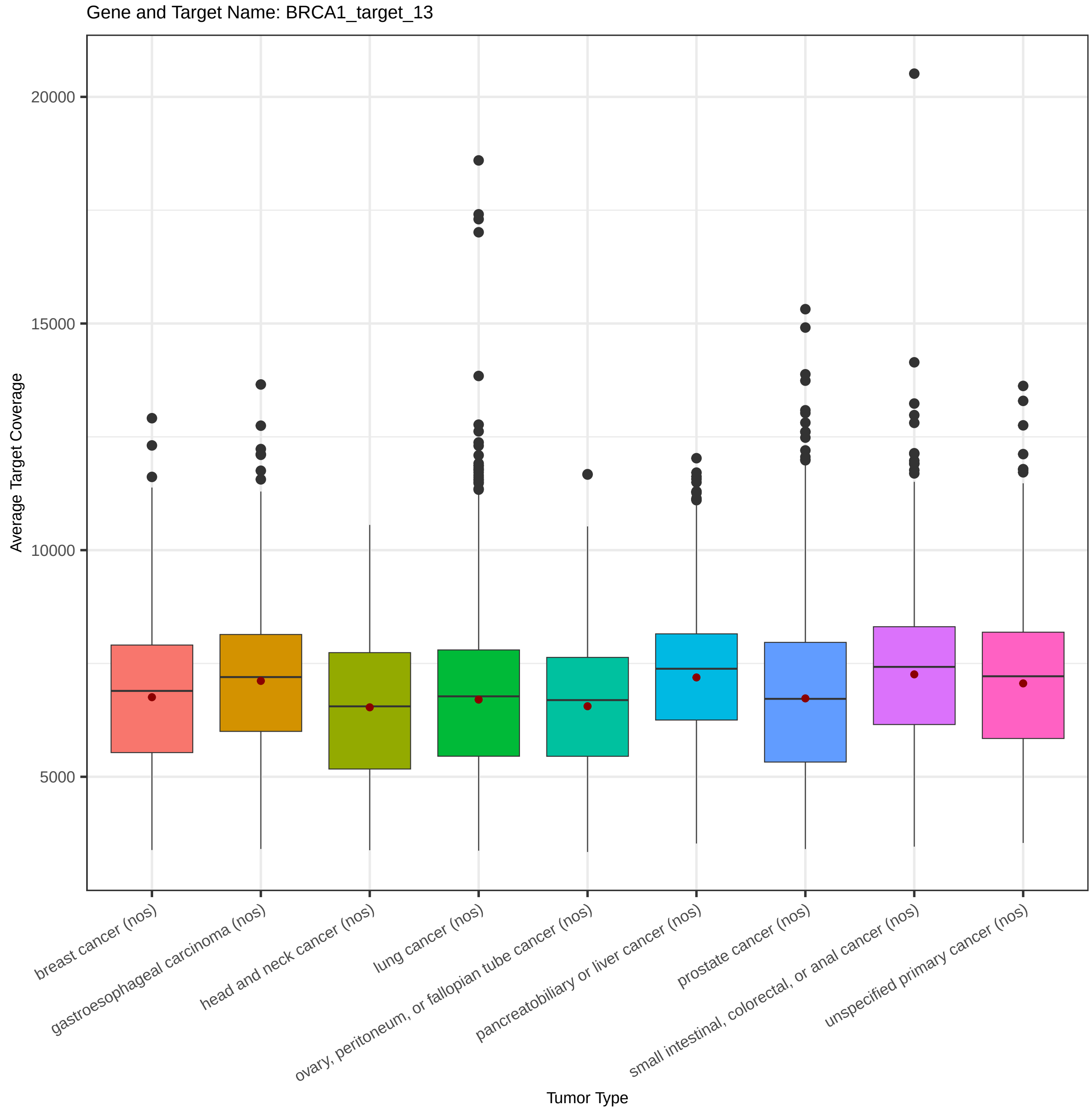
Gene and Target Name: BRCA1_target_13
Average Target Coverage
20000
15000
10000
5000
breast cancer (nos)
gastroesophageal carcinoma (nos)
head and neck cancer (nos)
lung cancer (nos)
ovary, peritoneum, or fallopian tube cancer (nos)
pancreatobiliary or liver cancer (nos)
prostate cancer (nos)
small intestinal, colorectal, or anal cancer (nos)
unspecified primary cancer (nos)
Tumor Type

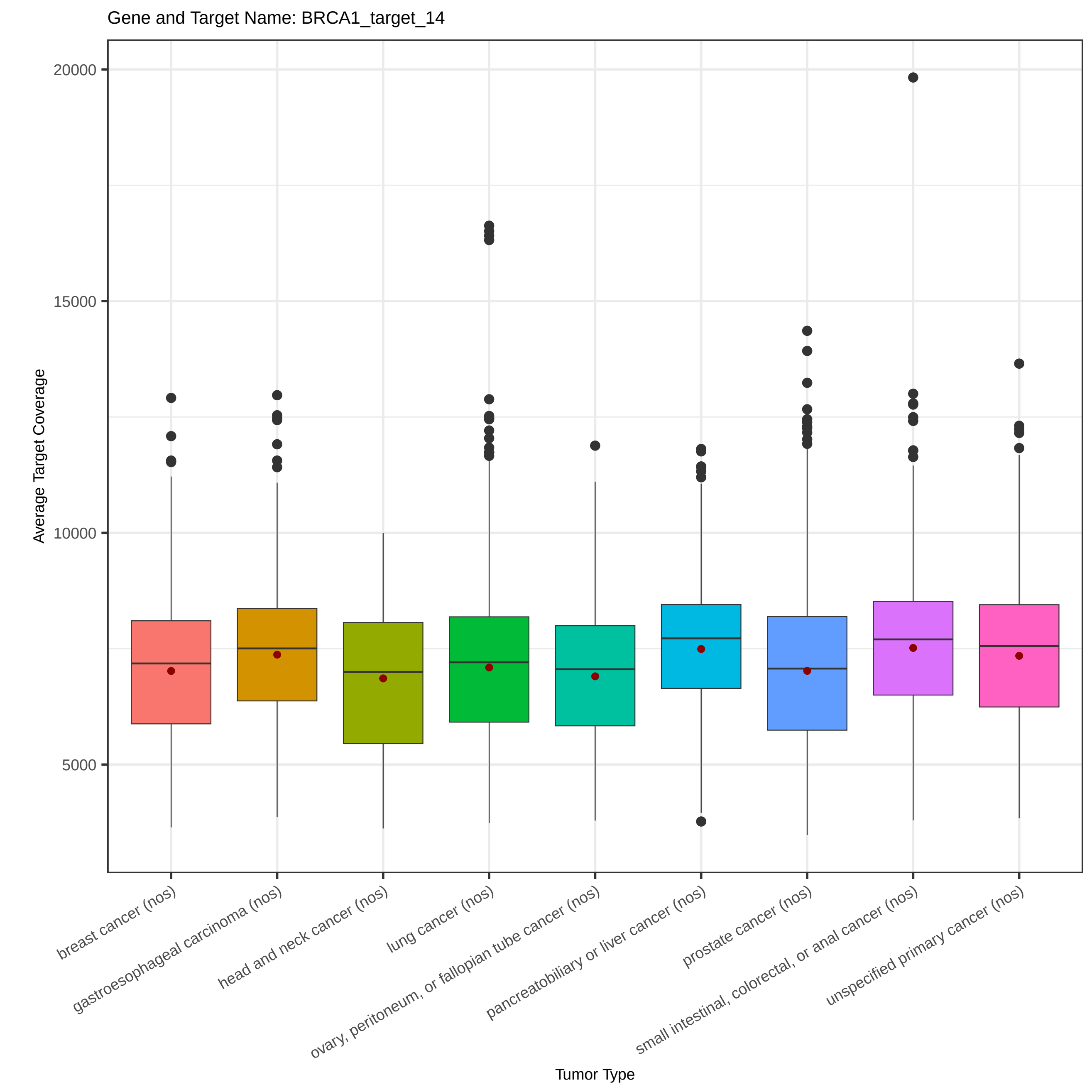

Gene and Target Name: BRCA1_target_14
Average Target Coverage
20000
15000
10000
5000
breast cancer (nos)
gastroesophageal carcinoma (nos)
head and neck cancer (nos)
lung cancer (nos)
ovary, peritoneum, or fallopian tube cancer (nos)
pancreatobiliary or liver cancer (nos)
prostate cancer (nos)
small intestinal, colorectal, or anal cancer (nos)
unspecified primary cancer (nos)
Tumor Type

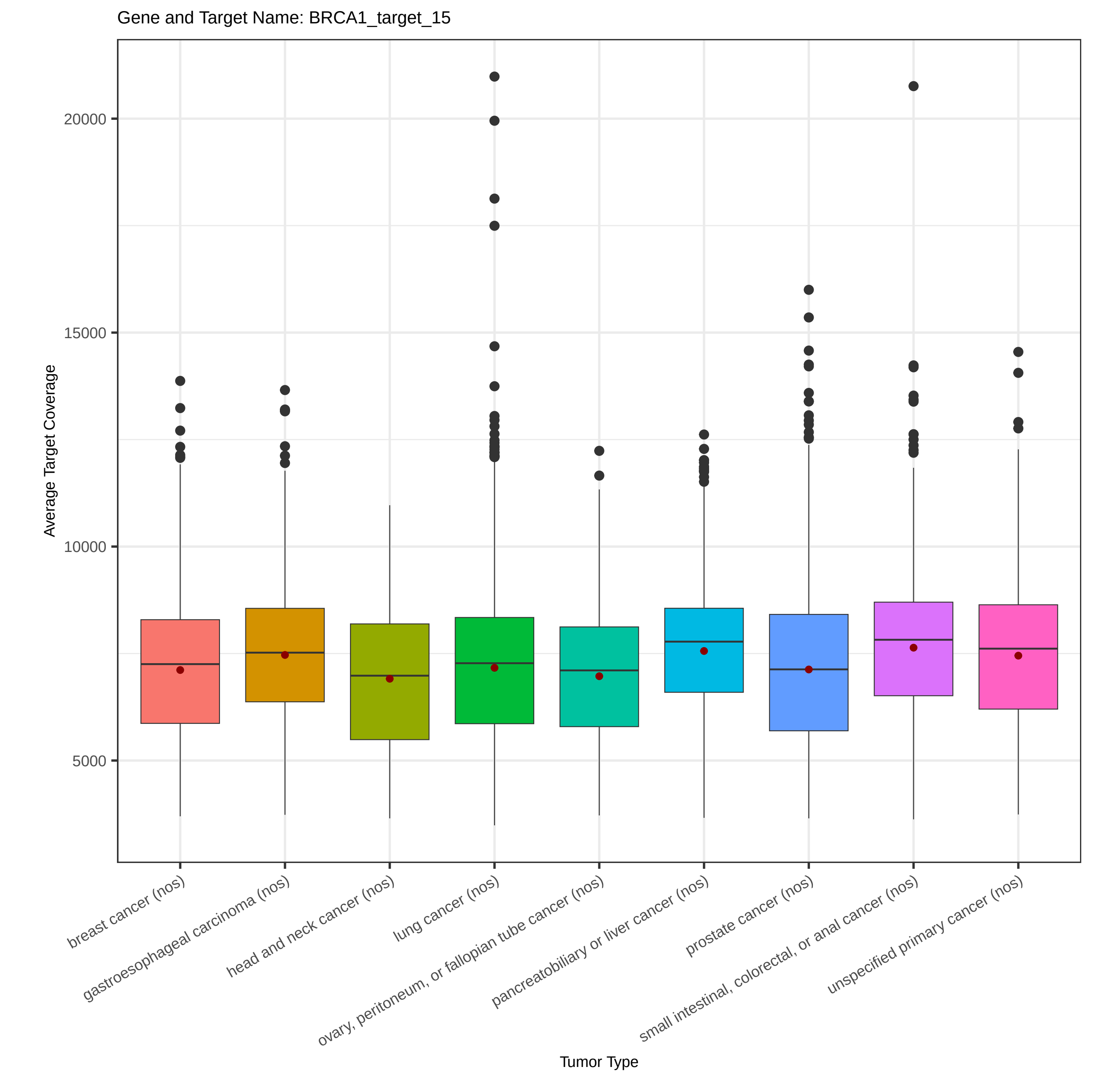

Gene and Target Name: BRCA1_target_15
Average Target Coverage
20000
15000
10000
5000
breast cancer (nos)
gastroesophageal carcinoma (nos)
head and neck cancer (nos)
lung cancer (nos)
ovary, peritoneum, or fallopian tube cancer (nos)
pancreatobiliary or liver cancer (nos)
prostate cancer (nos)
small intestinal, colorectal, or anal cancer (nos)
unspecified primary cancer (nos)
Tumor Type

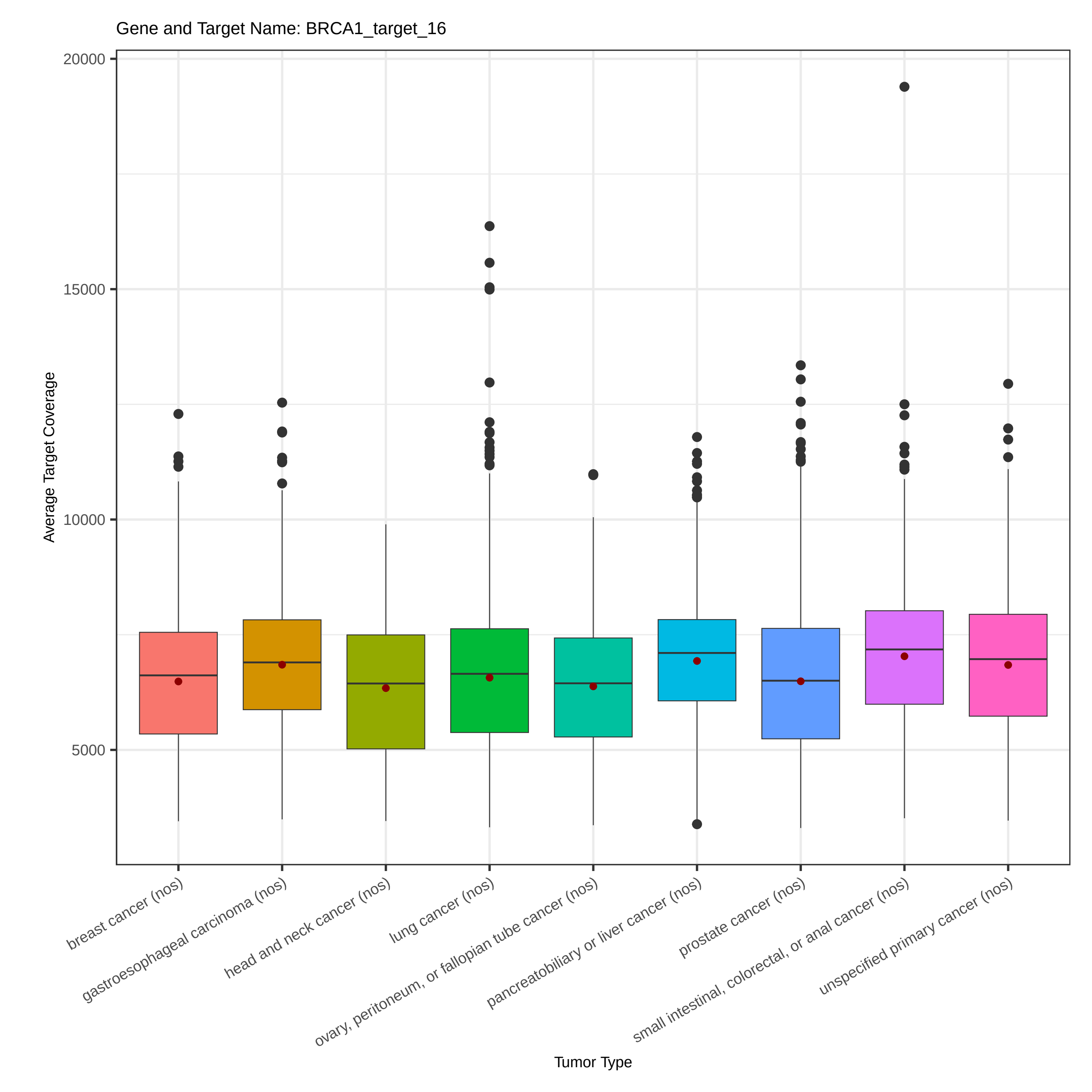
Gene and Target Name: BRCA1_target_16
Average Target Coverage
20000
15000
10000
5000
breast cancer (nos)
gastroesophageal carcinoma (nos)
head and neck cancer (nos)
lung cancer (nos)
ovary, peritoneum, or fallopian tube cancer (nos)
pancreatobiliary or liver cancer (nos)
prostate cancer (nos)
small intestinal, colorectal, or anal cancer (nos)
unspecified primary cancer (nos)
Tumor Type

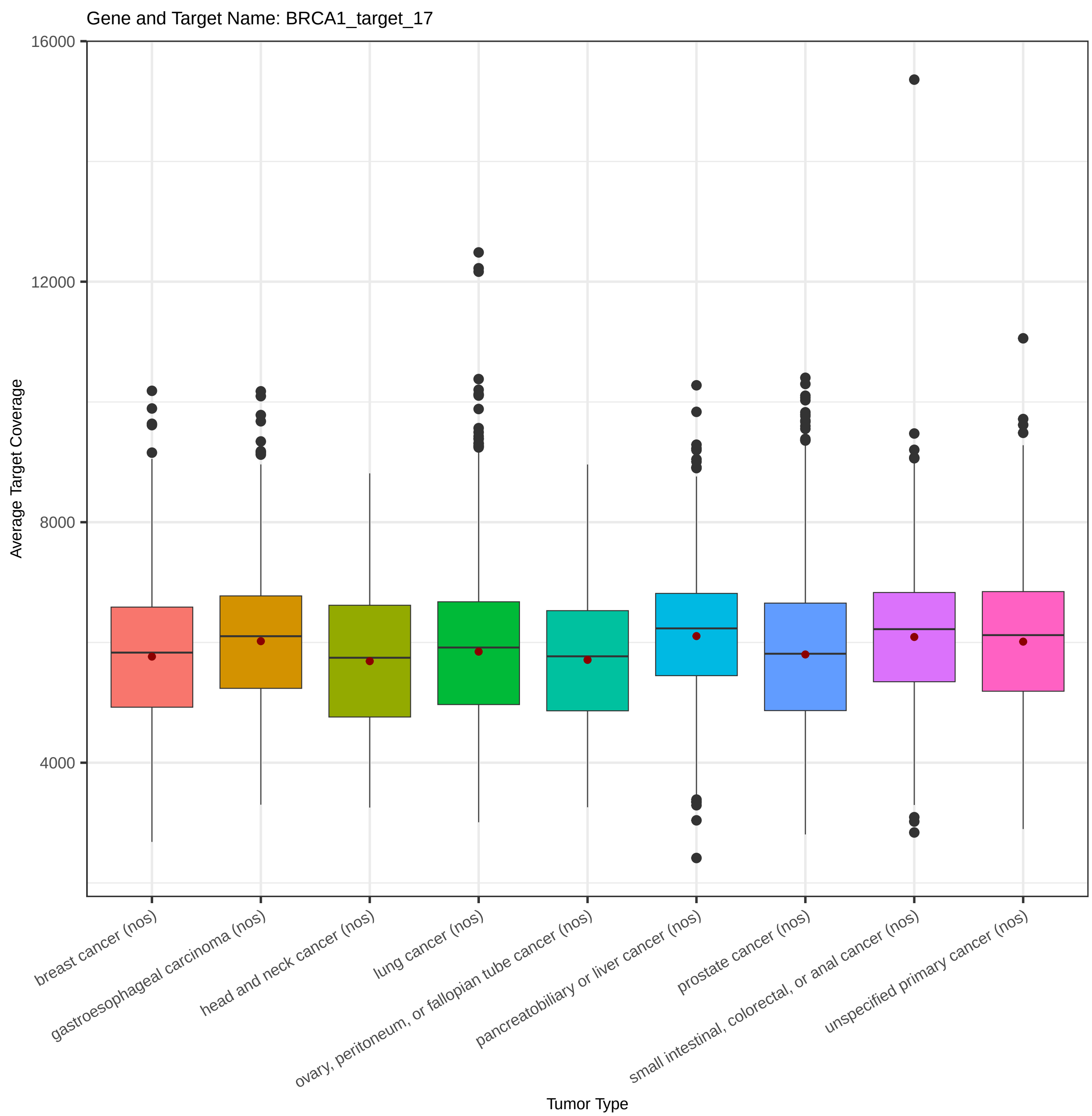

Gene and Target Name: BRCA1_target_17
Average Target Coverage
16000
12000
8000
4000
Tumor Type
breast cancer (nos)
gastroesophageal carcinoma (nos)
head and neck cancer (nos)
lung cancer (nos)
ovary, peritoneum, or fallopian tube cancer (nos)
pancreatobiliary or liver cancer (nos)
prostate cancer (nos)
small intestinal, colorectal, or anal cancer (nos)
unspecified primary cancer (nos)

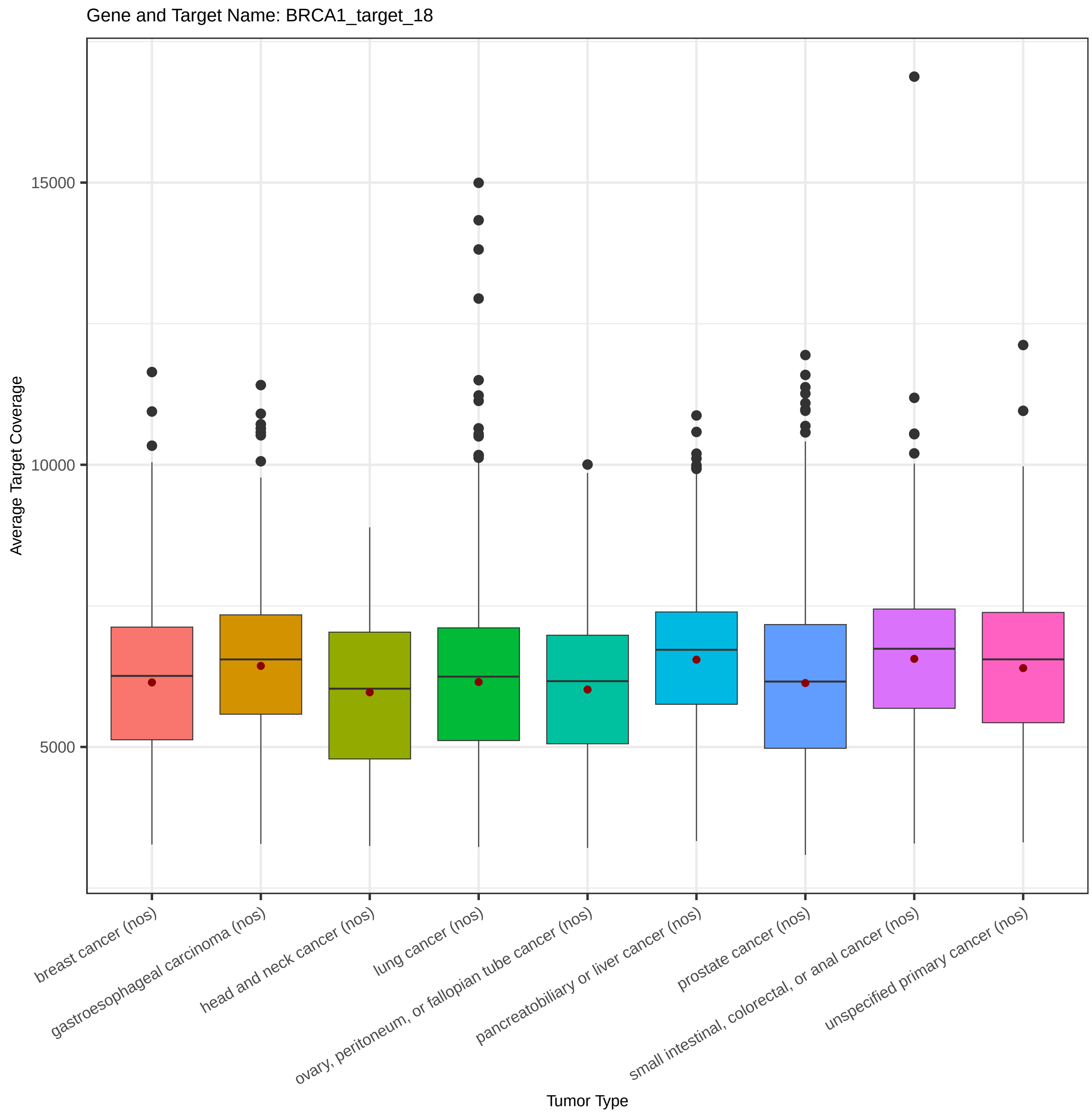
Gene and Target Name: BRCA1_target_18
Average Target Coverage
15000
10000
5000
breast cancer (nos)
gastroesophageal carcinoma (nos)
head and neck cancer (nos)
lung cancer (nos)
ovary, peritoneum, or fallopian tube cancer (nos)
pancreatobiliary or liver cancer (nos)
prostate cancer (nos)
small intestinal, colorectal, or anal cancer (nos)
unspecified primary cancer (nos)
Tumor Type

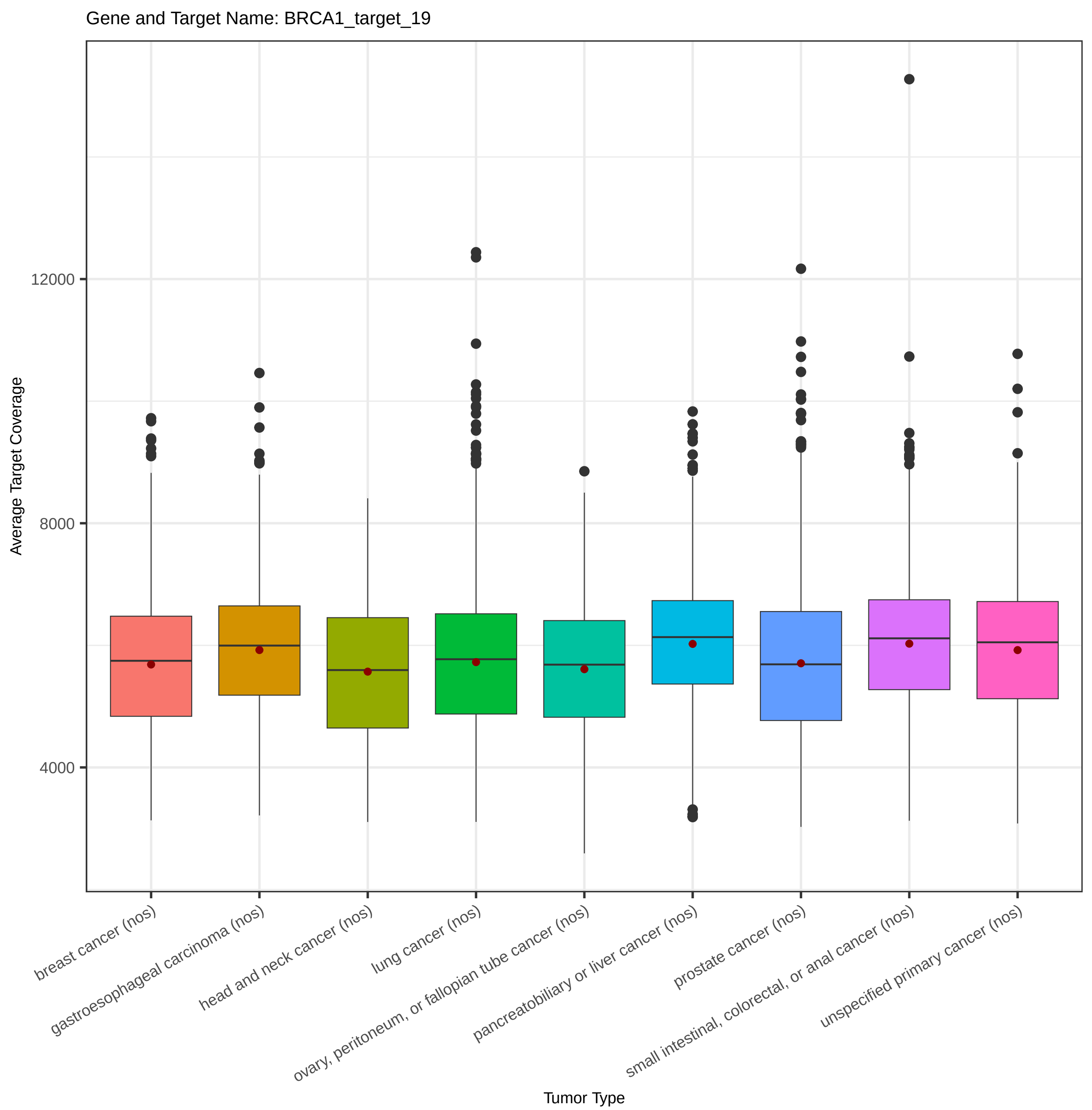

Gene and Target Name: BRCA1_target_19
Average Target Coverage
12000
8000
4000
Tumor Type
breast cancer (nos)
gastroesophageal carcinoma (nos)
head and neck cancer (nos)
lung cancer (nos)
ovary, peritoneum, or fallopian tube cancer (nos)
pancreatobiliary or liver cancer (nos)
prostate cancer (nos)
small intestinal, colorectal, or anal cancer (nos)
unspecified primary cancer (nos)

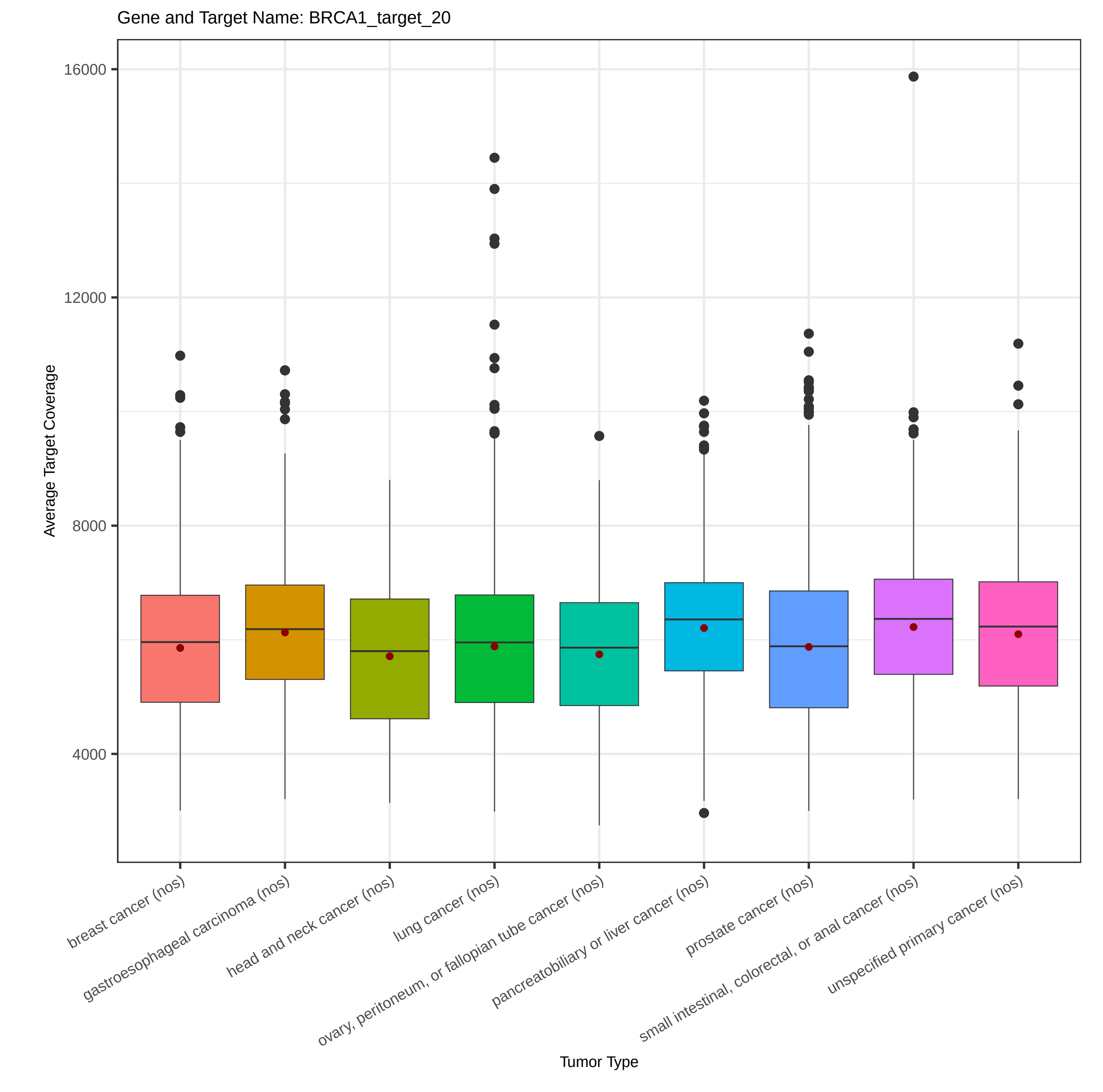
Gene and Target Name: BRCA1_target_20
Average Target Coverage
16000
12000
8000
4000
breast cancer (nos)
gastroesophageal carcinoma (nos)
head and neck cancer (nos)
lung cancer (nos)
ovary, peritoneum, or fallopian tube cancer (nos)
pancreatobiliary or liver cancer (nos)
prostate cancer (nos)
small intestinal, colorectal, or anal cancer (nos)
unspecified primary cancer (nos)
Tumor Type

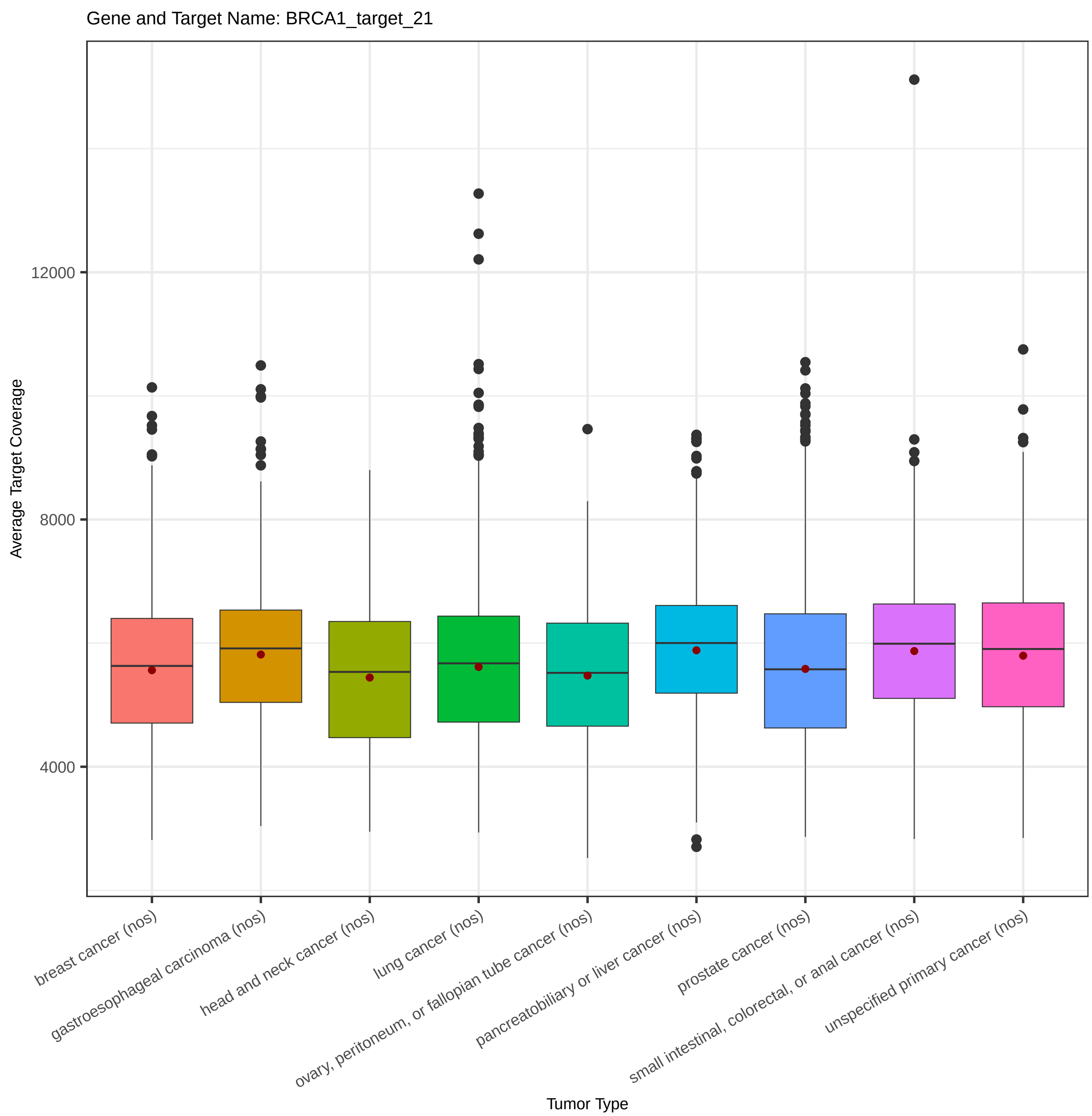
Gene and Target Name: BRCA1_target_21
Average Target Coverage
12000
8000
4000
breast cancer (nos)
gastroesophageal carcinoma (nos)
head and neck cancer (nos)
lung cancer (nos)
ovary, peritoneum, or fallopian tube cancer (nos)
pancreatobiliary or liver cancer (nos)
prostate cancer (nos)
small intestinal, colorectal, or anal cancer (nos)
unspecified primary cancer (nos)
Tumor Type

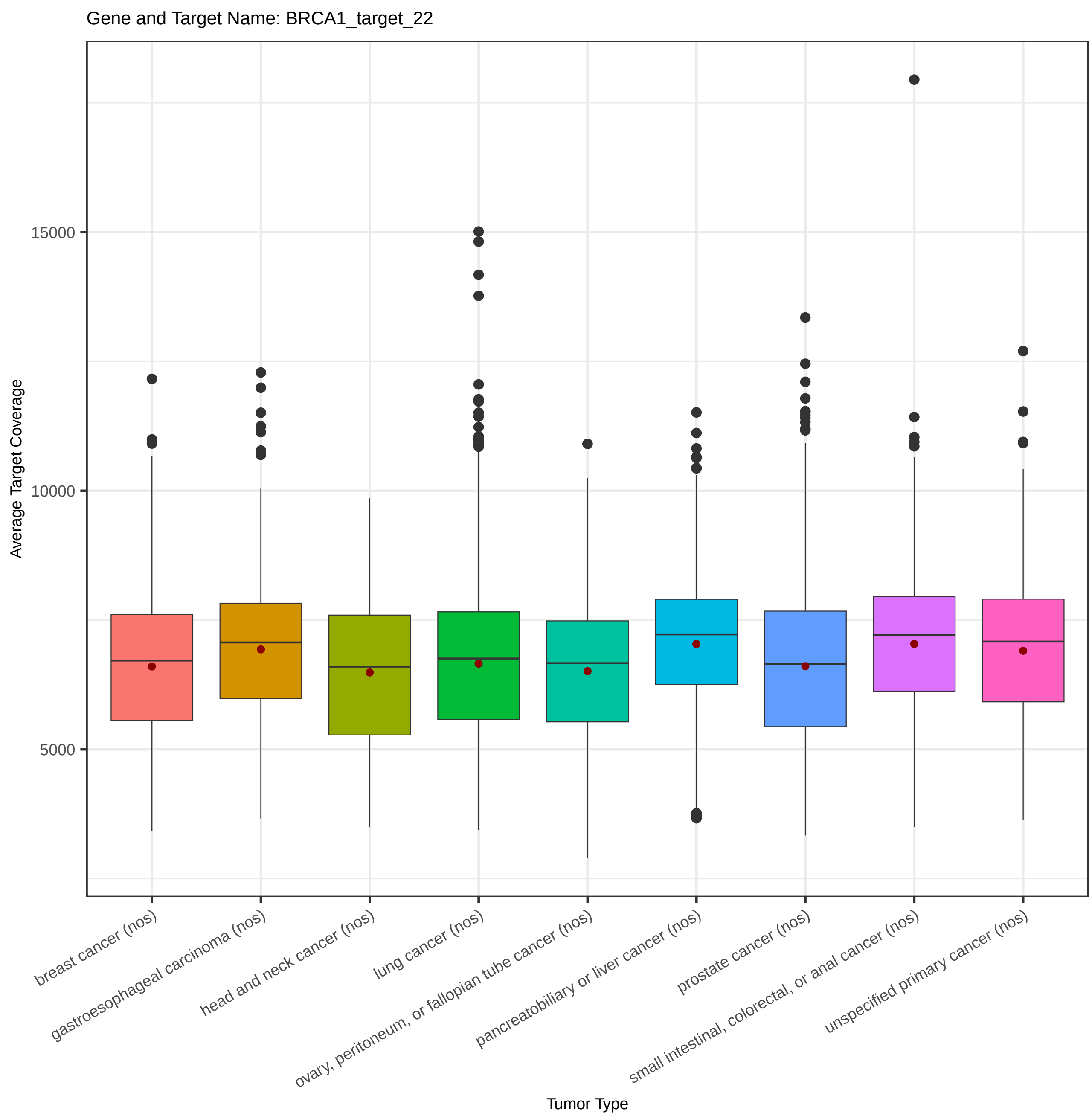

Gene and Target Name: BRCA1_target_22
Average Target Coverage
15000
10000
5000
breast cancer (nos)
gastroesophageal carcinoma (nos)
head and neck cancer (nos)
lung cancer (nos)
ovary, peritoneum, or fallopian tube cancer (nos)
pancreatobiliary or liver cancer (nos)
prostate cancer (nos)
small intestinal, colorectal, or anal cancer (nos)
unspecified primary cancer (nos)
Tumor Type

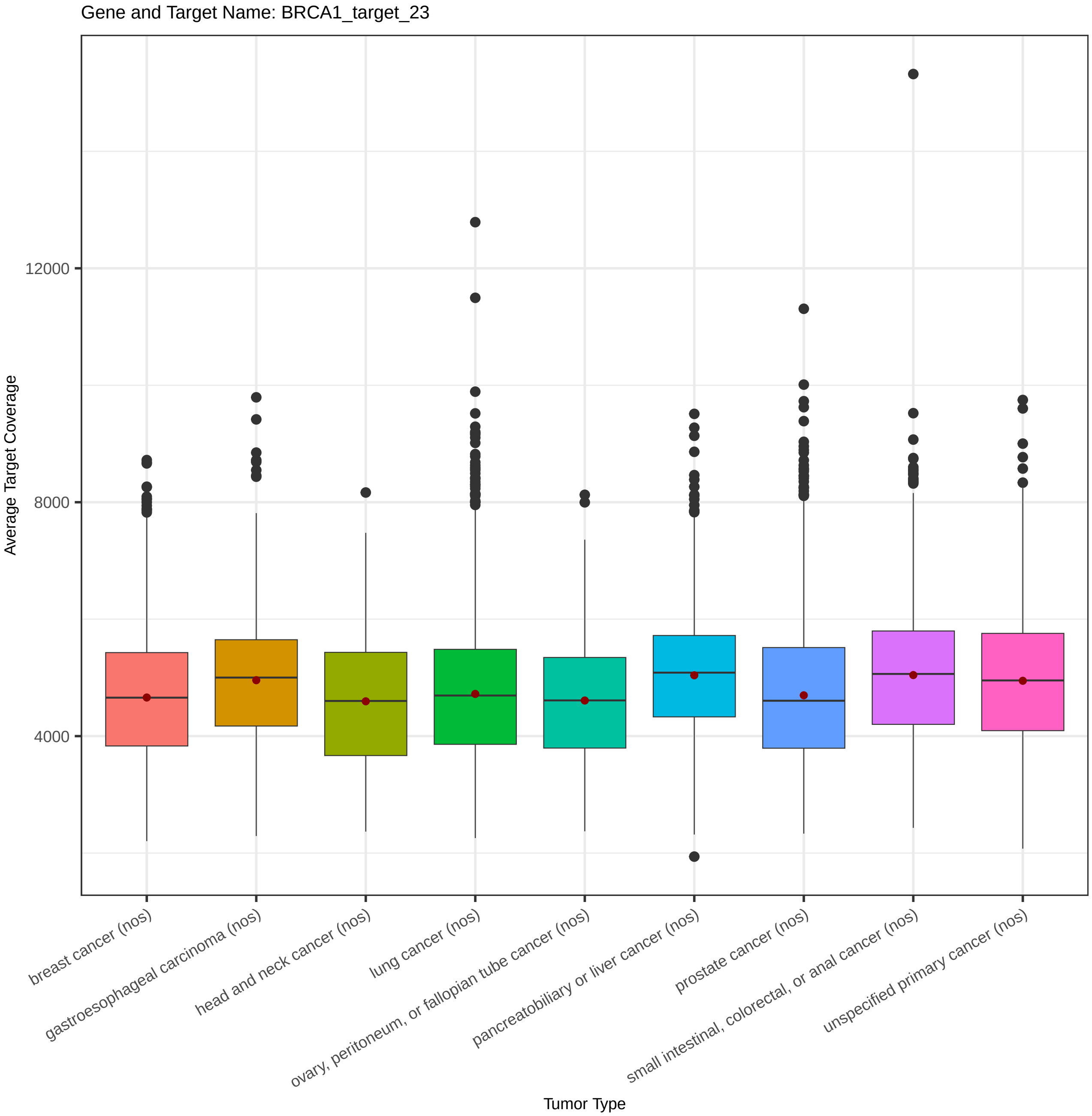

Gene and Target Name: BRCA1_target_23
Average Target Coverage
12000
8000
4000
breast cancer (nos)
gastroesophageal carcinoma (nos)
head and neck cancer (nos)
lung cancer (nos)
ovary, peritoneum, or fallopian tube cancer (nos)
pancreatobiliary or liver cancer (nos)
prostate cancer (nos)
small intestinal, colorectal, or anal cancer (nos)
unspecified primary cancer (nos)
Tumor Type

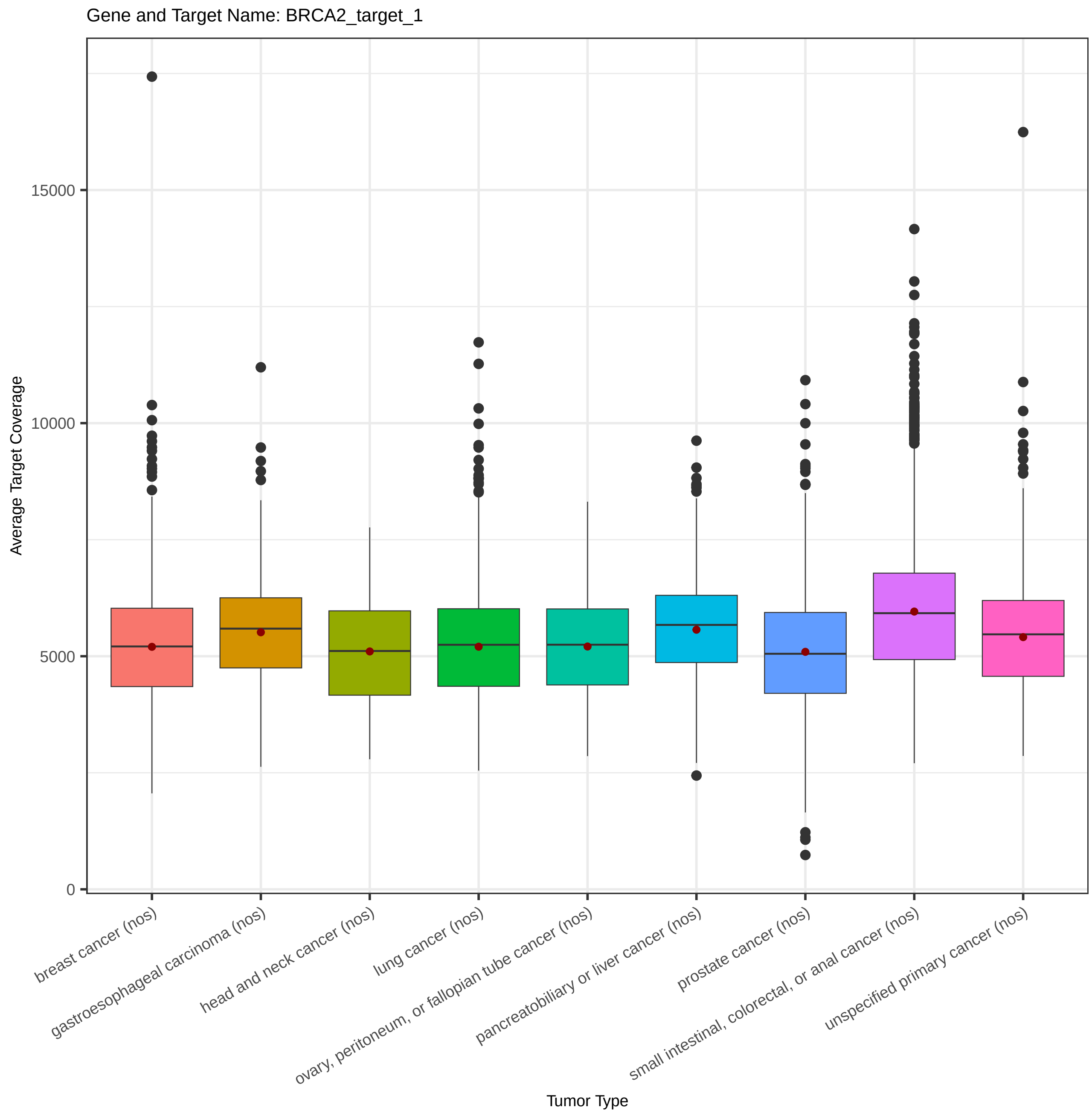

Gene and Target Name: BRCA2_target_1
Average Target Coverage
15000
10000
5000
0
breast cancer (nos)
gastroesophageal carcinoma (nos)
head and neck cancer (nos)
lung cancer (nos)
ovary, peritoneum, or fallopian tube cancer (nos)
pancreatobiliary or liver cancer (nos)
prostate cancer (nos)
small intestinal, colorectal, or anal cancer (nos)
unspecified primary cancer (nos)
Tumor Type

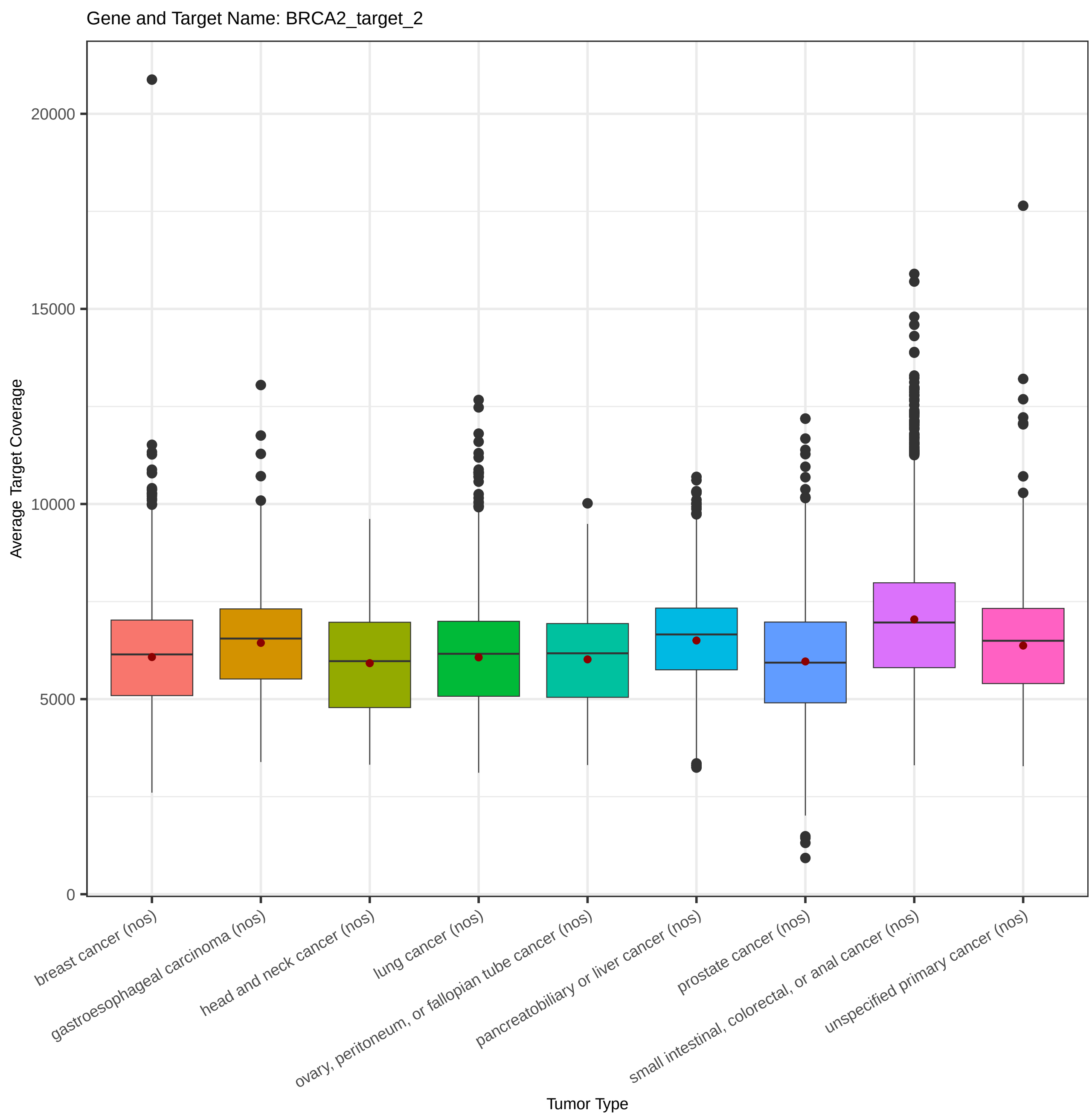

Gene and Target Name: BRCA2_target_2
Average Target Coverage
0
5000
10000
15000
20000
breast cancer (nos)
gastroesophageal carcinoma (nos)
head and neck cancer (nos)
lung cancer (nos)
ovary, peritoneum, or fallopian tube cancer (nos)
pancreatobiliary or liver cancer (nos)
prostate cancer (nos)
small intestinal, colorectal, or anal cancer (nos)
unspecified primary cancer (nos)
Tumor Type

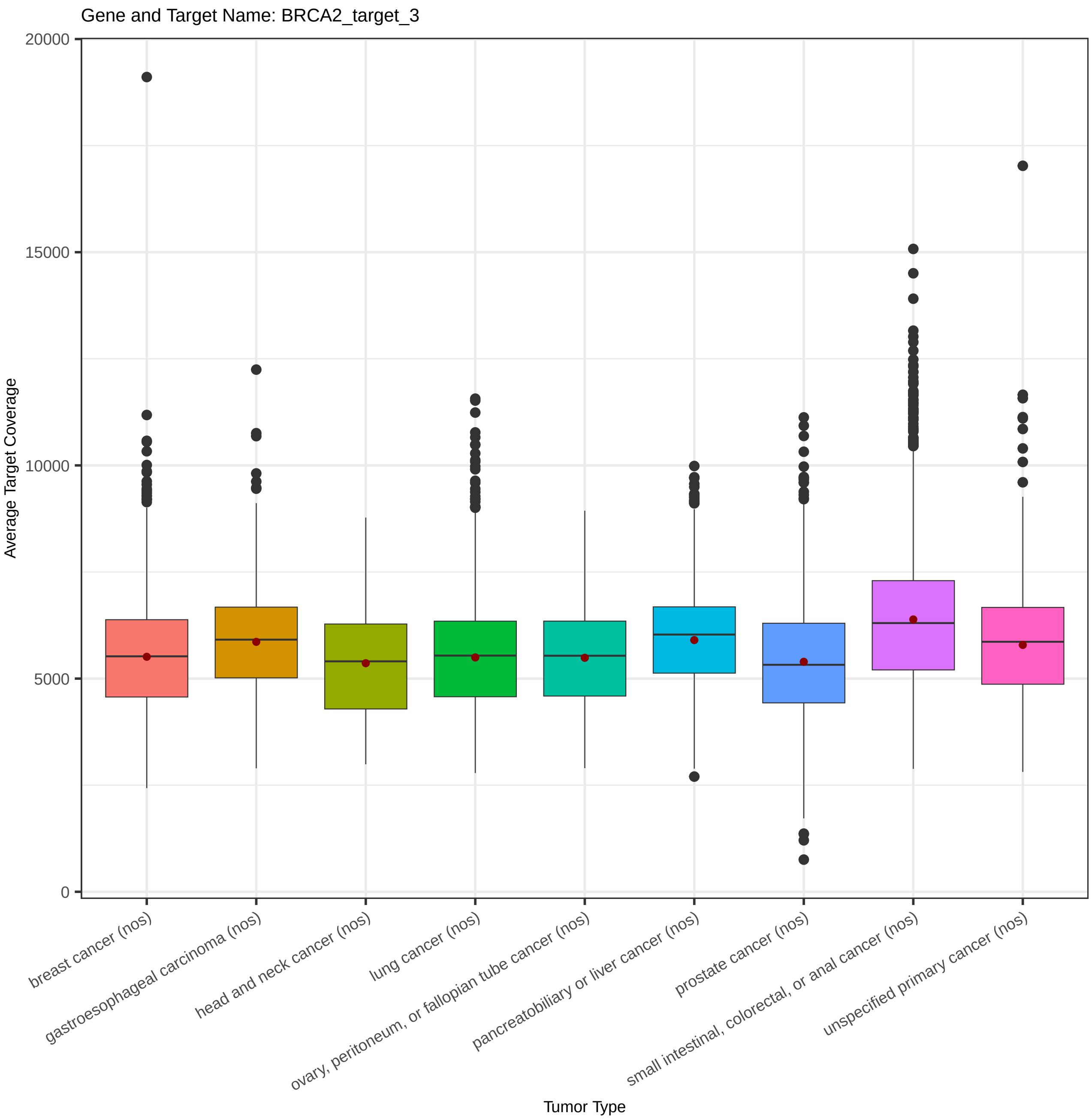

Gene and Target Name: BRCA2_target_3
20000
15000
10000
5000
0
Average Target Coverage
breast cancer (nos)
gastroesophageal carcinoma (nos)
head and neck cancer (nos)
lung cancer (nos)
ovary, peritoneum, or fallopian tube cancer (nos)
pancreatobiliary or liver cancer (nos)
prostate cancer (nos)
small intestinal, colorectal, or anal cancer (nos)
unspecified primary cancer (nos)
Tumor Type

Gene and Target Name: BRCA2_target_4

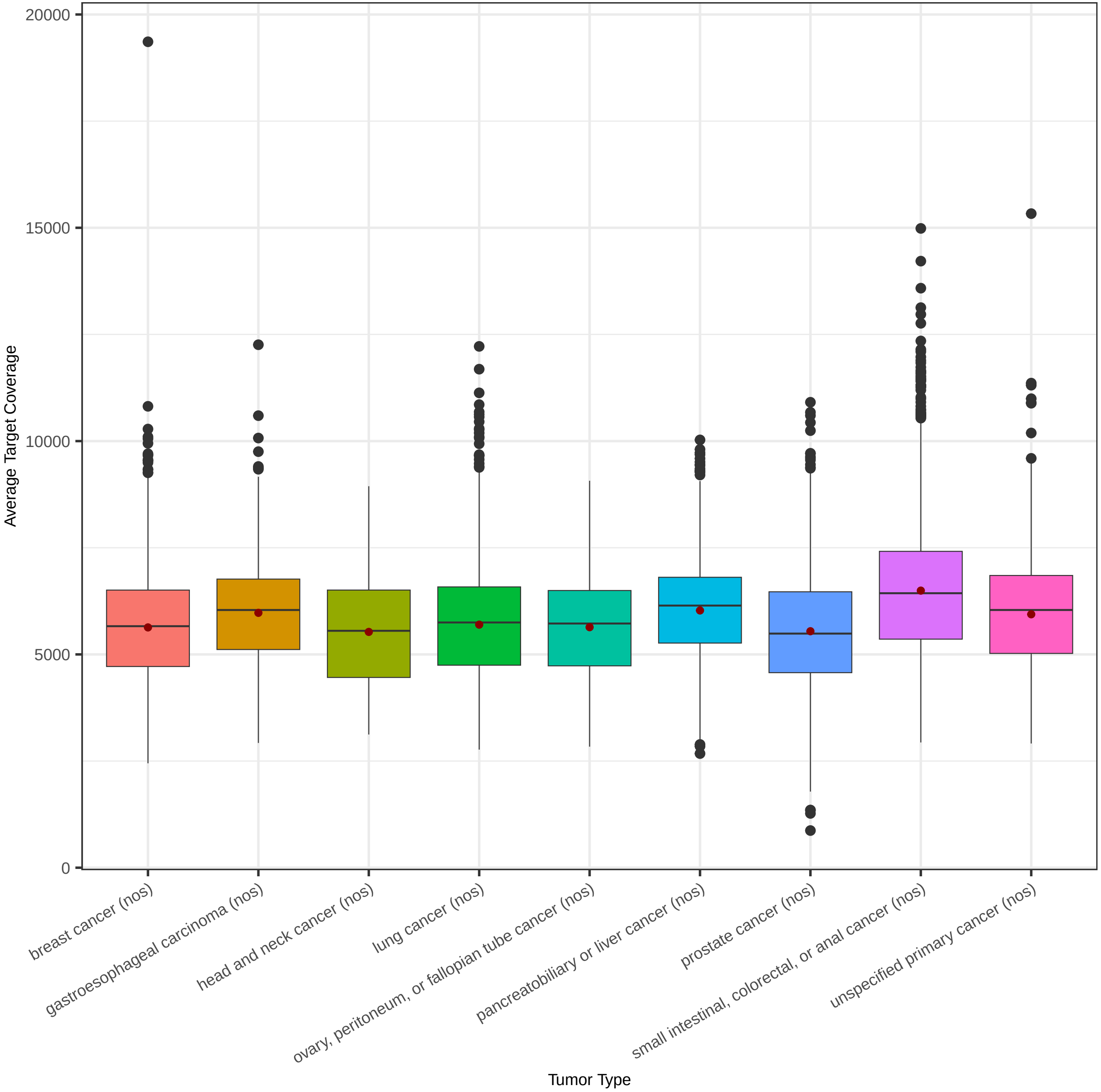

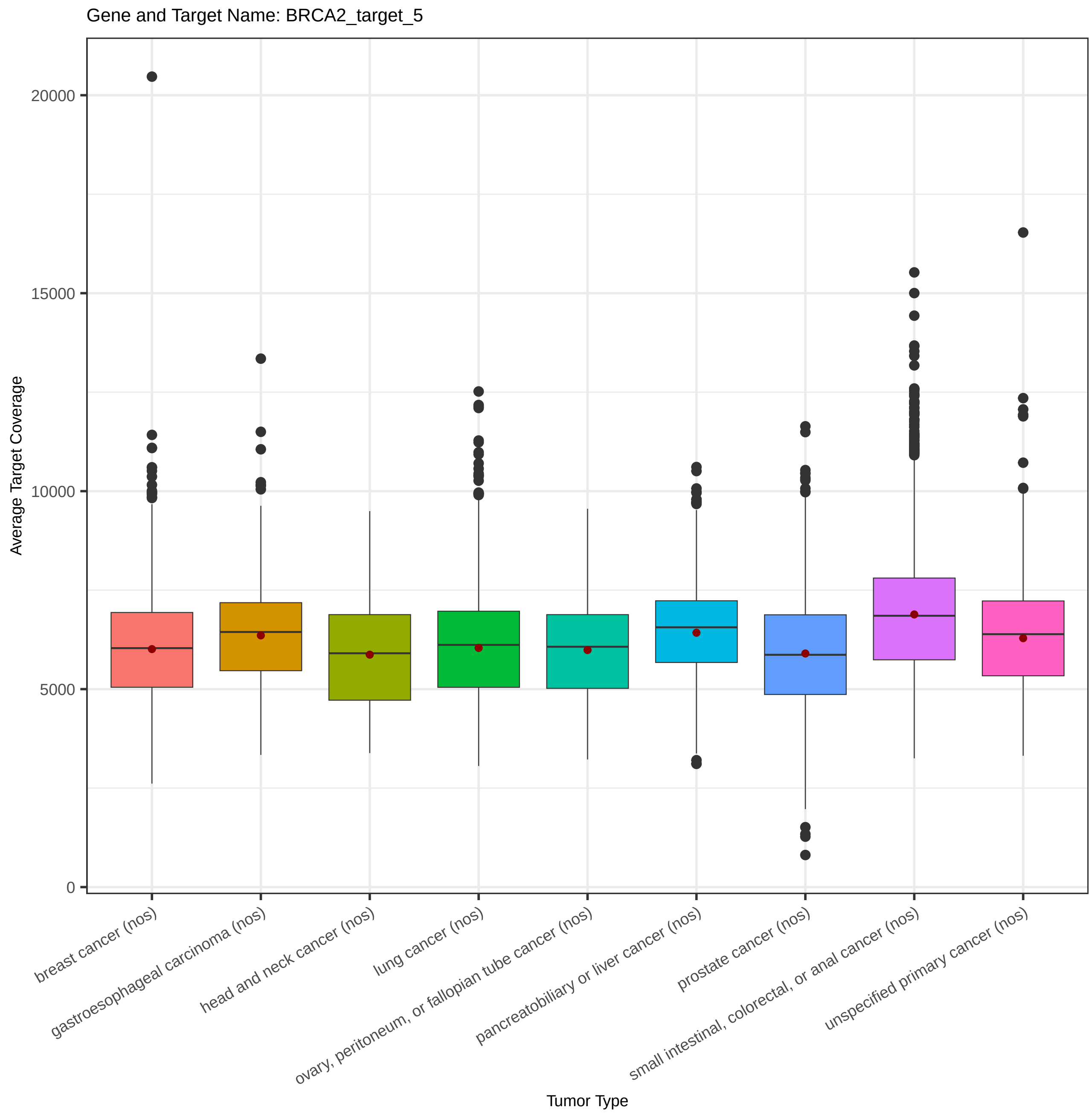
Gene and Target Name: BRCA2_target_5
Average Target Coverage
20000
15000
10000
5000
0
breast cancer (nos)
gastroesophageal carcinoma (nos)
head and neck cancer (nos)
lung cancer (nos)
ovary, peritoneum, or fallopian tube cancer (nos)
pancreatobiliary or liver cancer (nos)
prostate cancer (nos)
small intestinal, colorectal, or anal cancer (nos)
unspecified primary cancer (nos)
Tumor Type

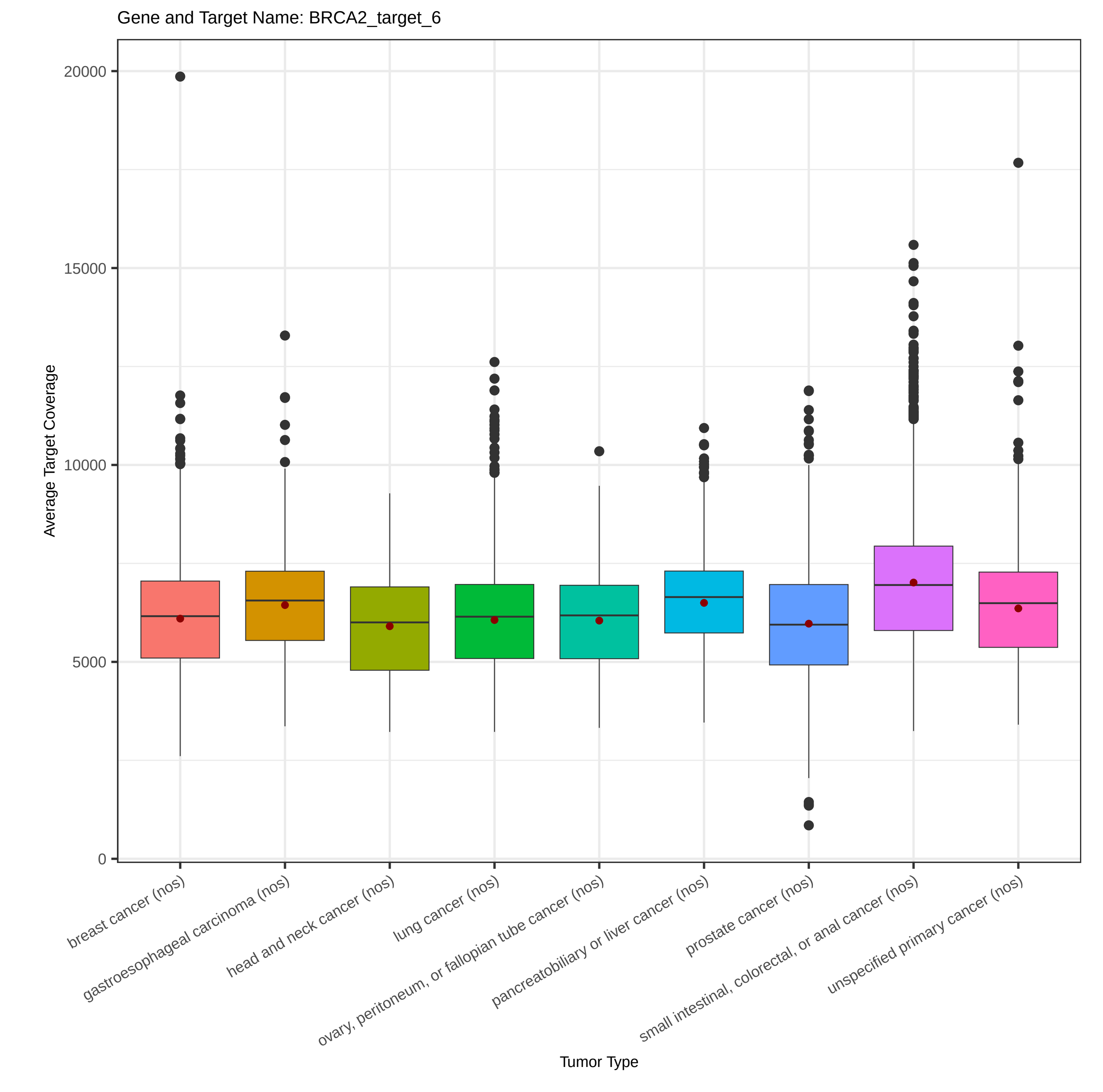

Gene and Target Name: BRCA2_target_6
Average Target Coverage
20000
15000
10000
5000
0
breast cancer (nos)
gastroesophageal carcinoma (nos)
head and neck cancer (nos)
lung cancer (nos)
ovary, peritoneum, or fallopian tube cancer (nos)
pancreatobiliary or liver cancer (nos)
prostate cancer (nos)
small intestinal, colorectal, or anal cancer (nos)
unspecified primary cancer (nos)
Tumor Type

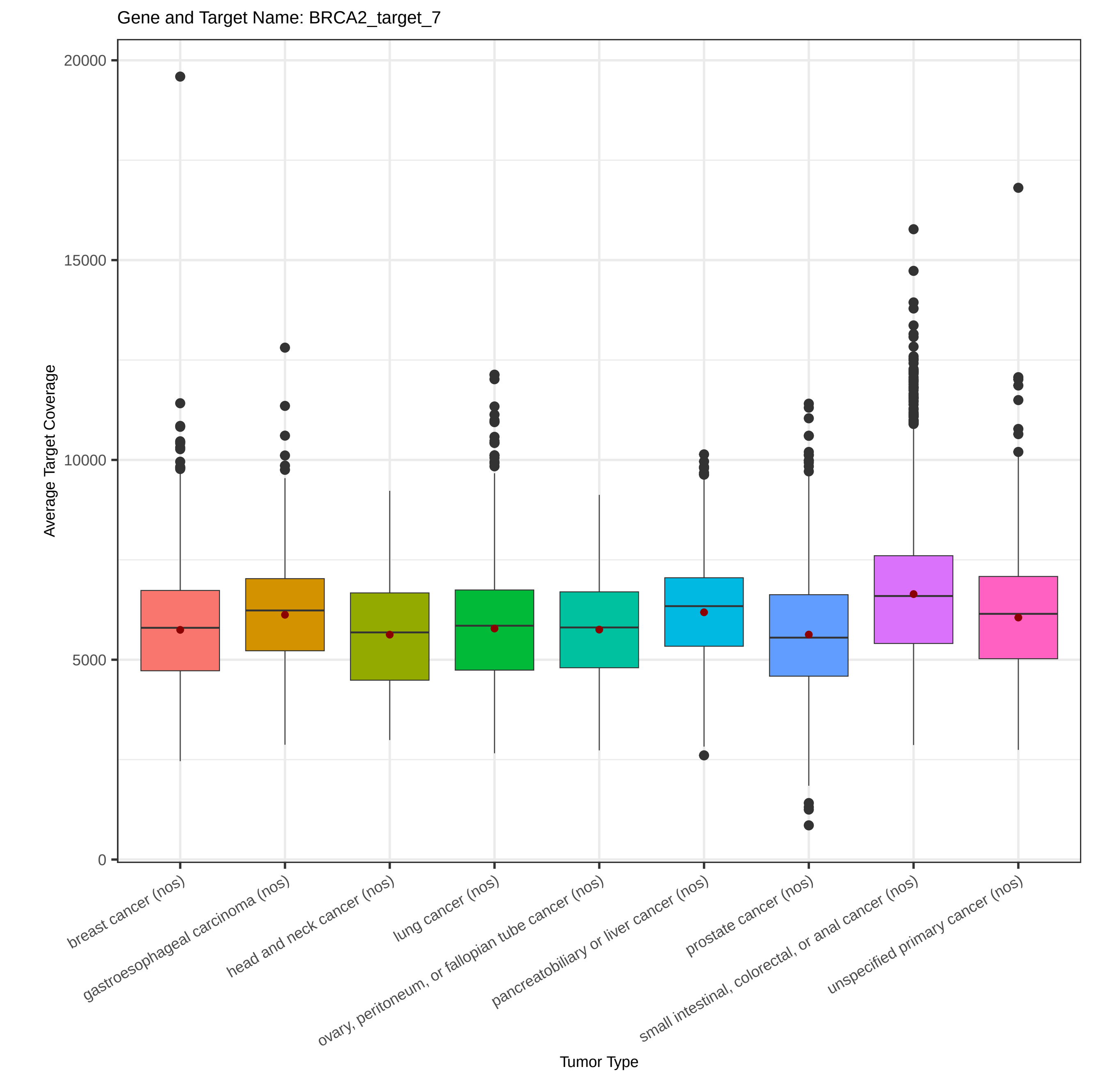

Gene and Target Name: BRCA2_target_7
Average Target Coverage
20000
15000
10000
5000
0
breast cancer (nos)
gastroesophageal carcinoma (nos)
head and neck cancer (nos)
lung cancer (nos)
ovary, peritoneum, or fallopian tube cancer (nos)
pancreatobiliary or liver cancer (nos)
prostate cancer (nos)
small intestinal, colorectal, or anal cancer (nos)
unspecified primary cancer (nos)
Tumor Type

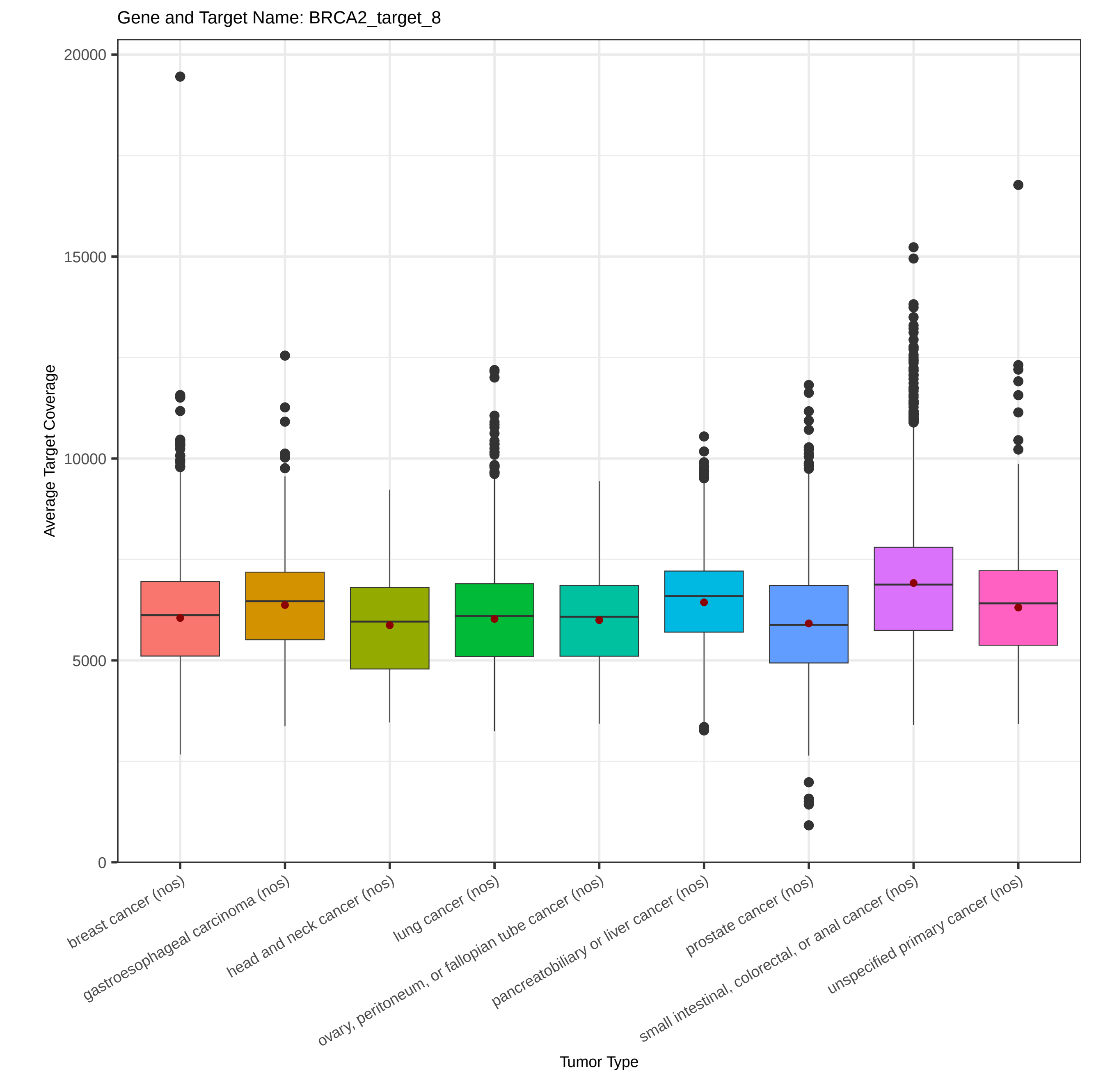
Gene and Target Name: BRCA2_target_8
Average Target Coverage
0
5000
10000
15000
20000
breast cancer (nos)
gastroesophageal carcinoma (nos)
head and neck cancer (nos)
lung cancer (nos)
ovary, peritoneum, or fallopian tube cancer (nos)
pancreatobiliary or liver cancer (nos)
prostate cancer (nos)
small intestinal, colorectal, or anal cancer (nos)
unspecified primary cancer (nos)
Tumor Type

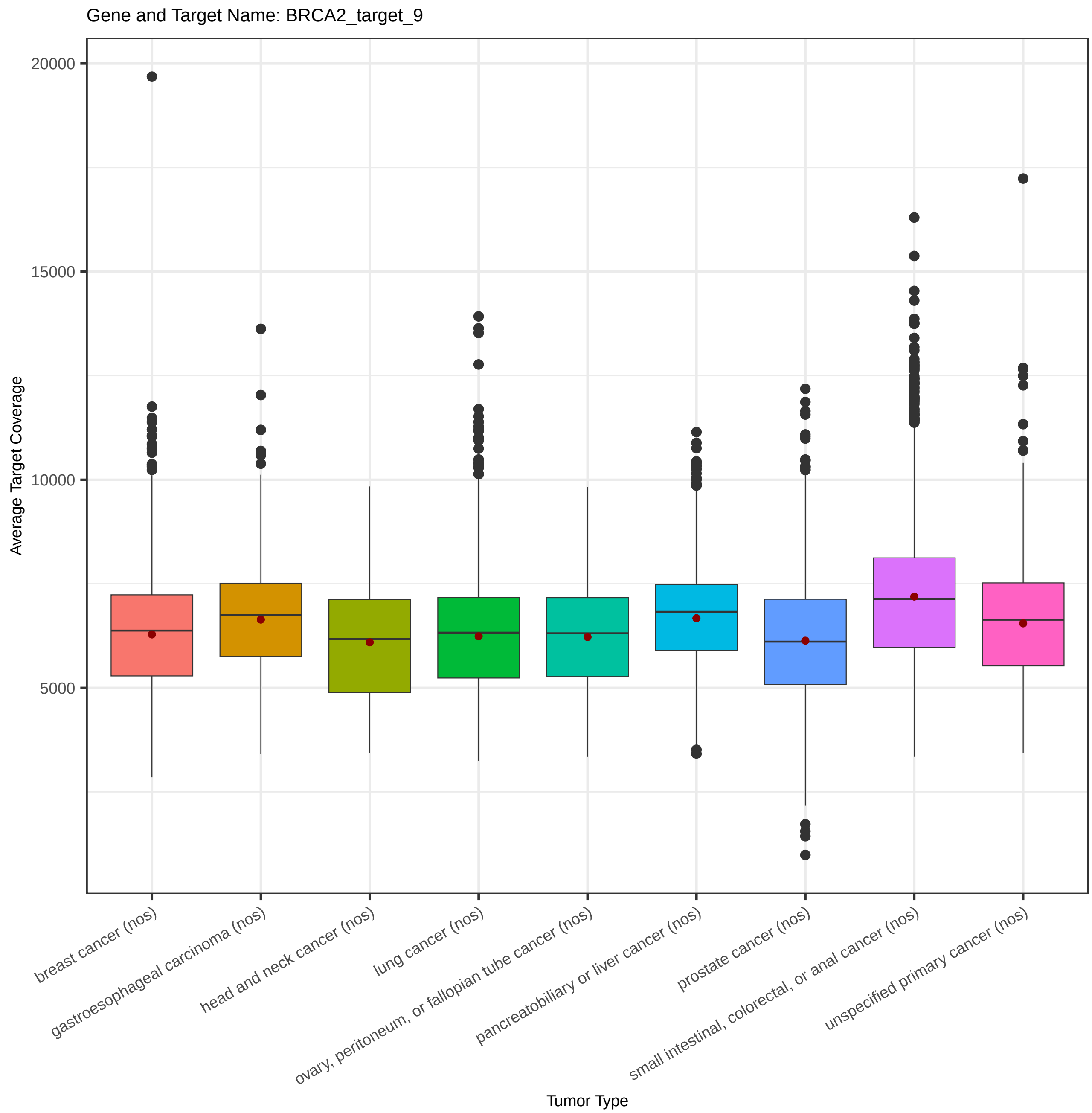
Gene and Target Name: BRCA2_target_9
20000
15000
10000
5000
Average Target Coverage
breast cancer (nos)
gastroesophageal carcinoma (nos)
head and neck cancer (nos)
lung cancer (nos)
ovary, peritoneum, or fallopian tube cancer (nos)
pancreatobiliary or liver cancer (nos)
prostate cancer (nos)
small intestinal, colorectal, or anal cancer (nos)
unspecified primary cancer (nos)
Tumor Type

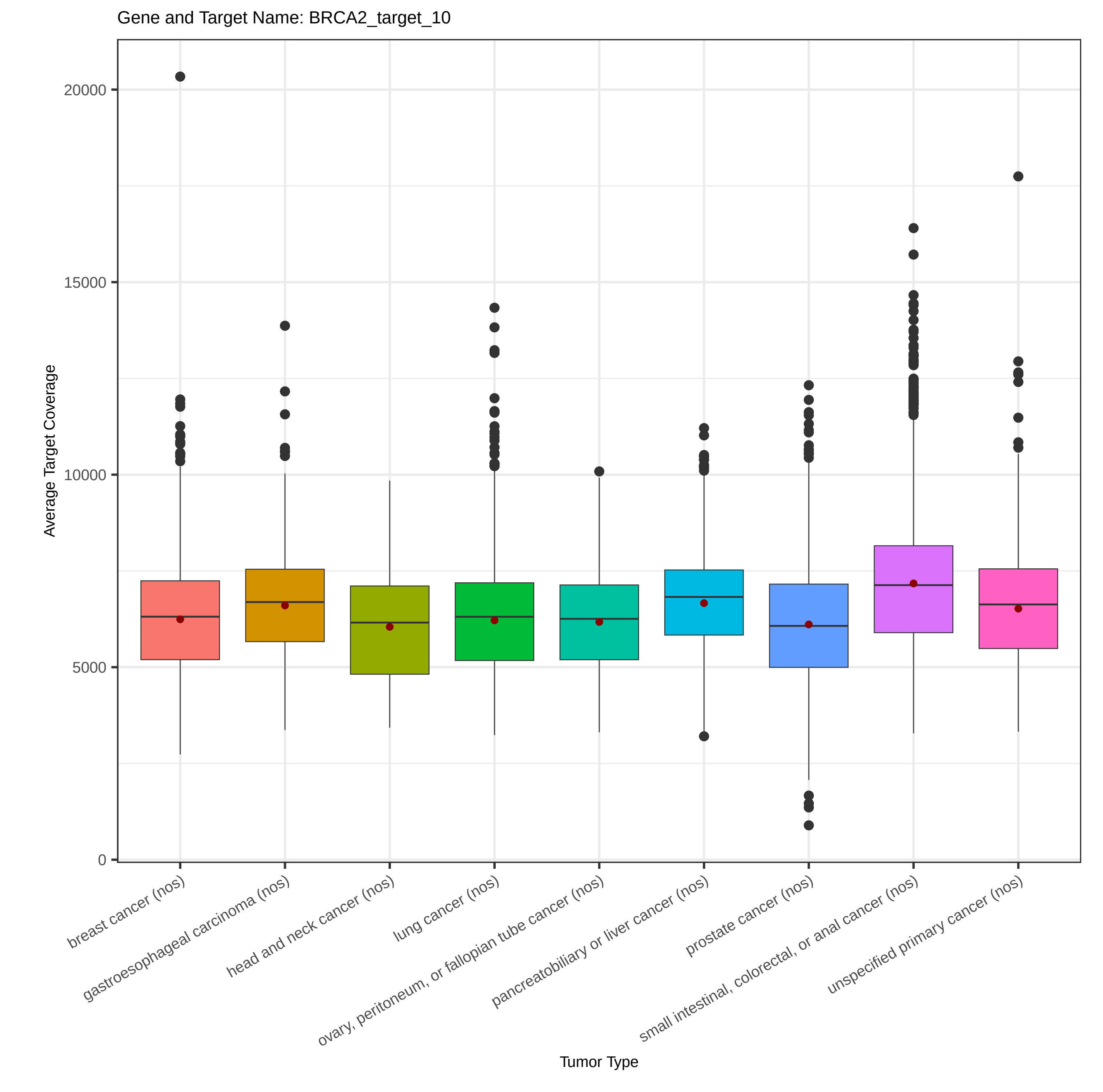

Gene and Target Name: BRCA2_target_10
Average Target Coverage
20000
15000
10000
5000
0
breast cancer (nos)
gastroesophageal carcinoma (nos)
head and neck cancer (nos)
lung cancer (nos)
ovary, peritoneum, or fallopian tube cancer (nos)
pancreatobiliary or liver cancer (nos)
prostate cancer (nos)
small intestinal, colorectal, or anal cancer (nos)
unspecified primary cancer (nos)
Tumor Type

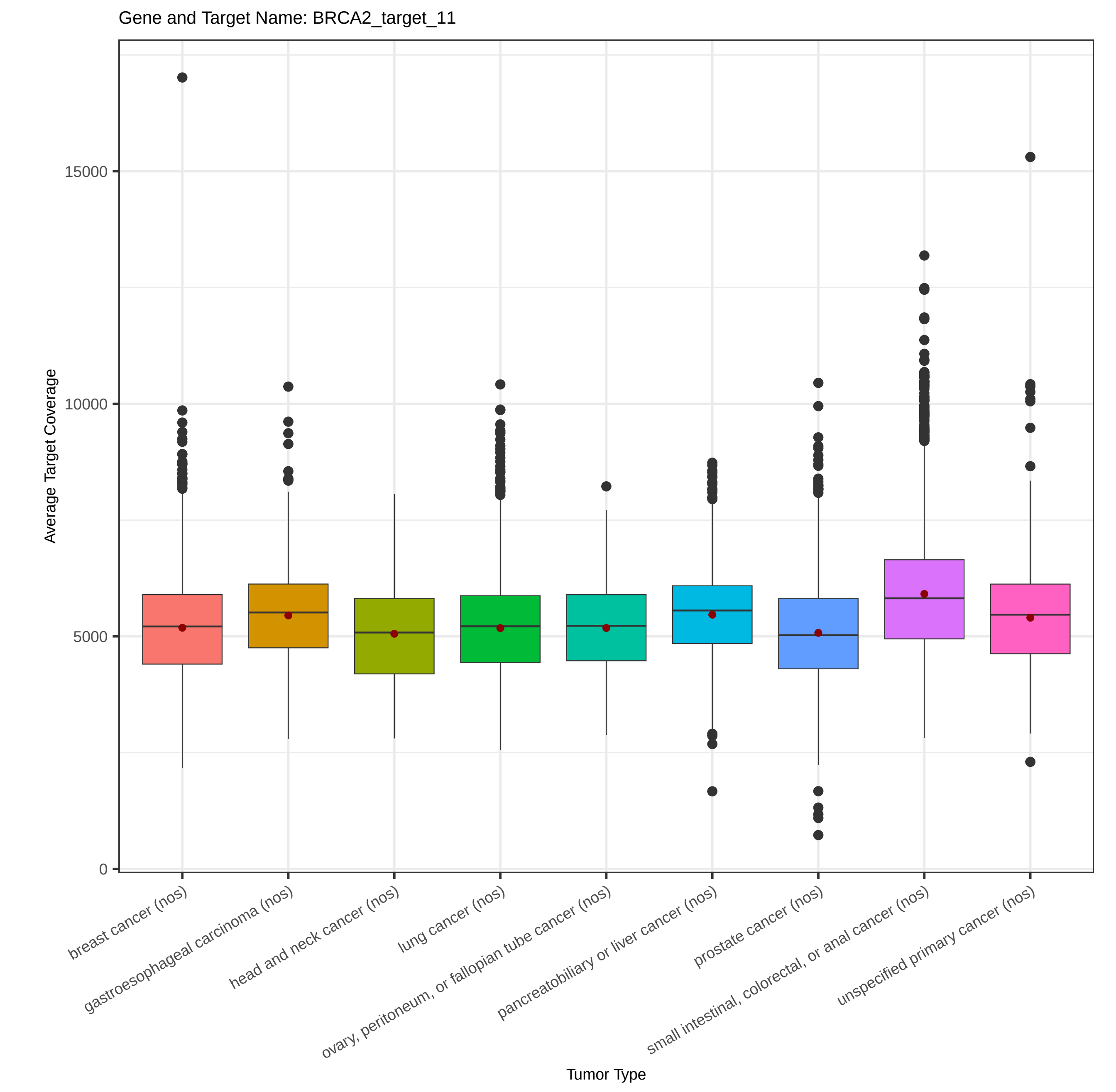

Gene and Target Name: BRCA2_target_11
Average Target Coverage
15000
10000
5000
0
breast cancer (nos)
gastroesophageal carcinoma (nos)
head and neck cancer (nos)
lung cancer (nos)
ovary, peritoneum, or fallopian tube cancer (nos)
pancreatobiliary or liver cancer (nos)
prostate cancer (nos)
small intestinal, colorectal, or anal cancer (nos)
unspecified primary cancer (nos)
Tumor Type

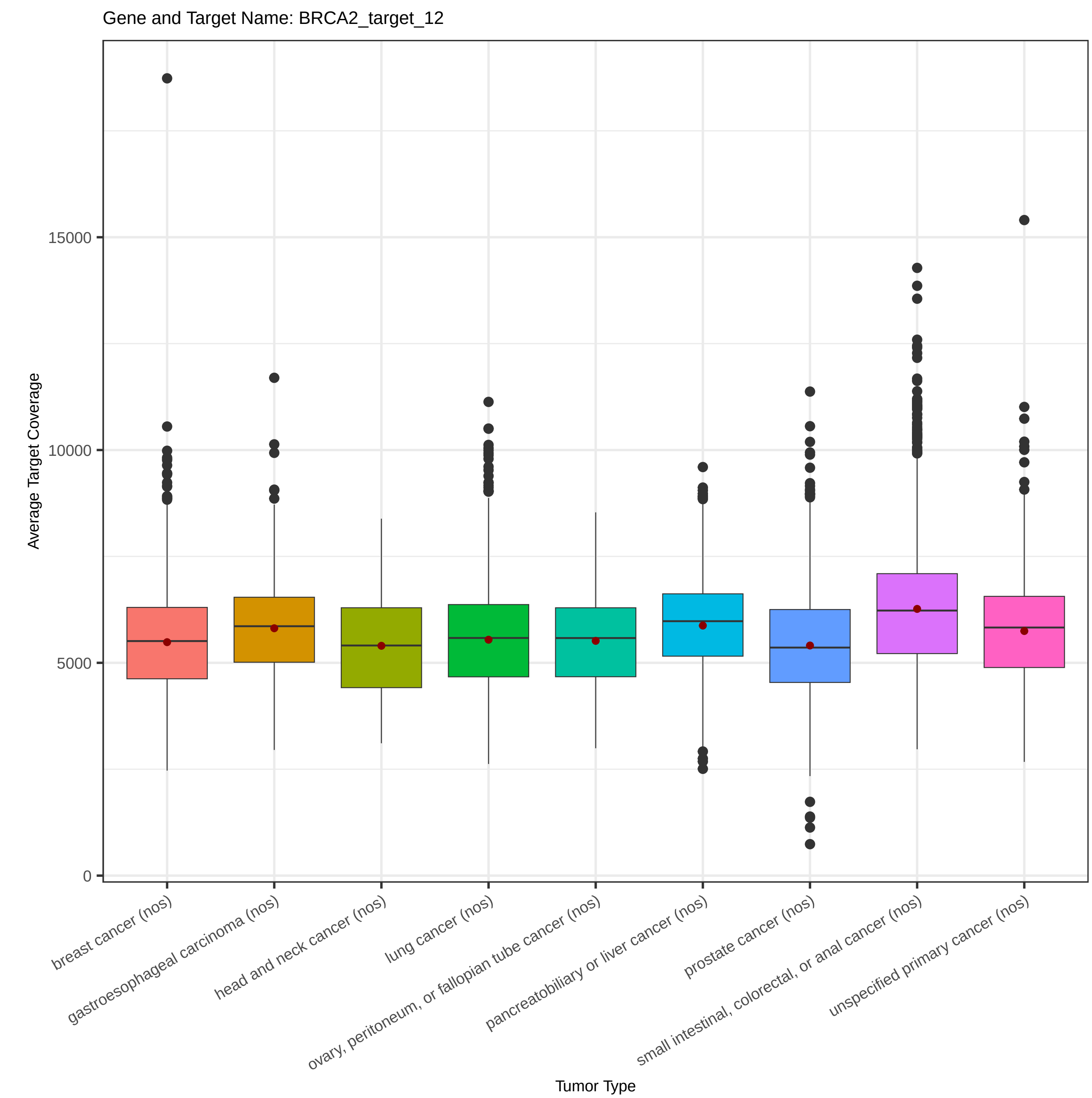

Gene and Target Name: BRCA2_target_12
Average Target Coverage
15000
10000
5000
0
breast cancer (nos)
gastroesophageal carcinoma (nos)
head and neck cancer (nos)
lung cancer (nos)
ovary, peritoneum, or fallopian tube cancer (nos)
pancreatobiliary or liver cancer (nos)
prostate cancer (nos)
small intestinal, colorectal, or anal cancer (nos)
unspecified primary cancer (nos)
Tumor Type

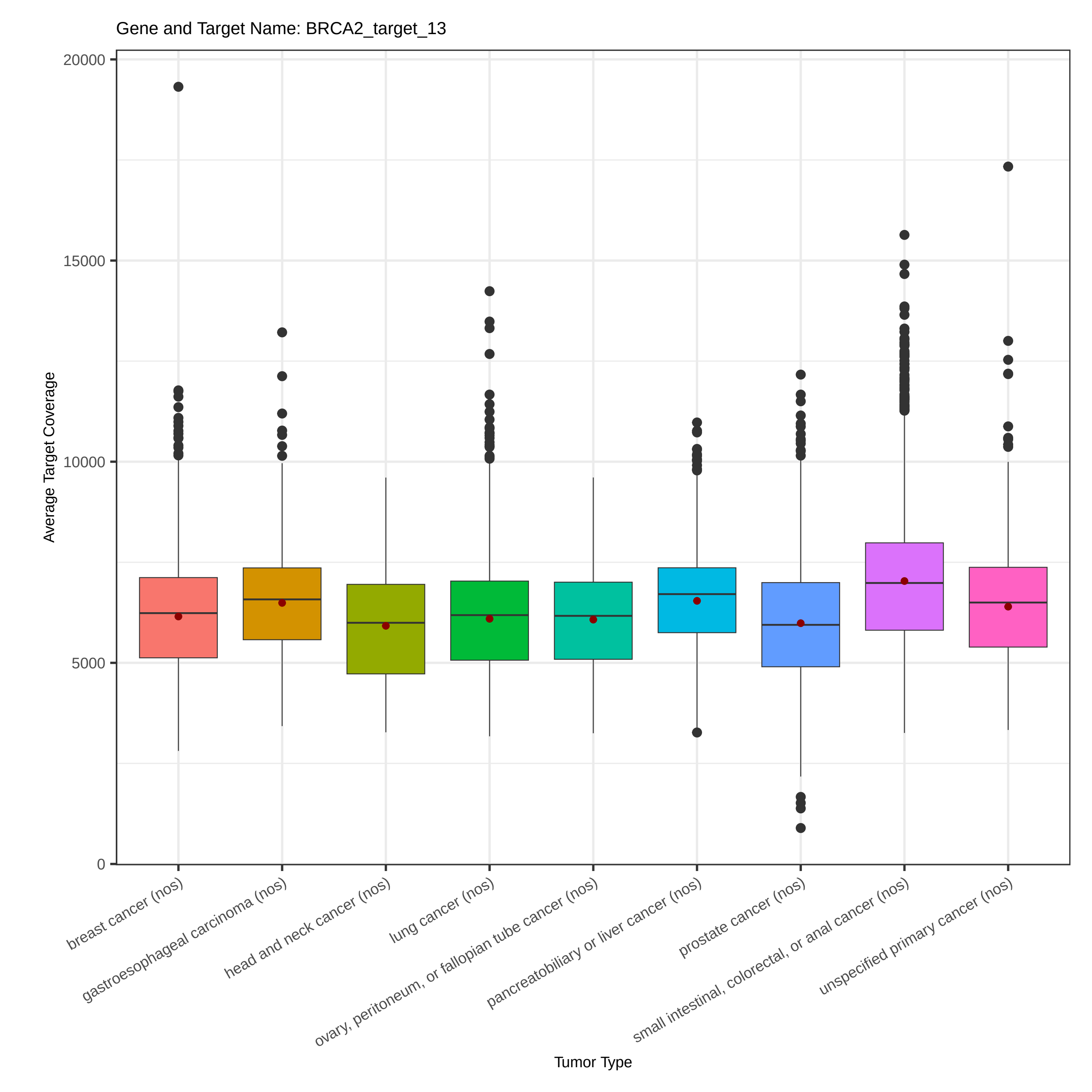
Gene and Target Name: BRCA2_target_13
Average Target Coverage
20000
15000
10000
5000
0
breast cancer (nos)
gastroesophageal carcinoma (nos)
head and neck cancer (nos)
lung cancer (nos)
ovary, peritoneum, or fallopian tube cancer (nos)
pancreatobiliary or liver cancer (nos)
prostate cancer (nos)
small intestinal, colorectal, or anal cancer (nos)
unspecified primary cancer (nos)
Tumor Type

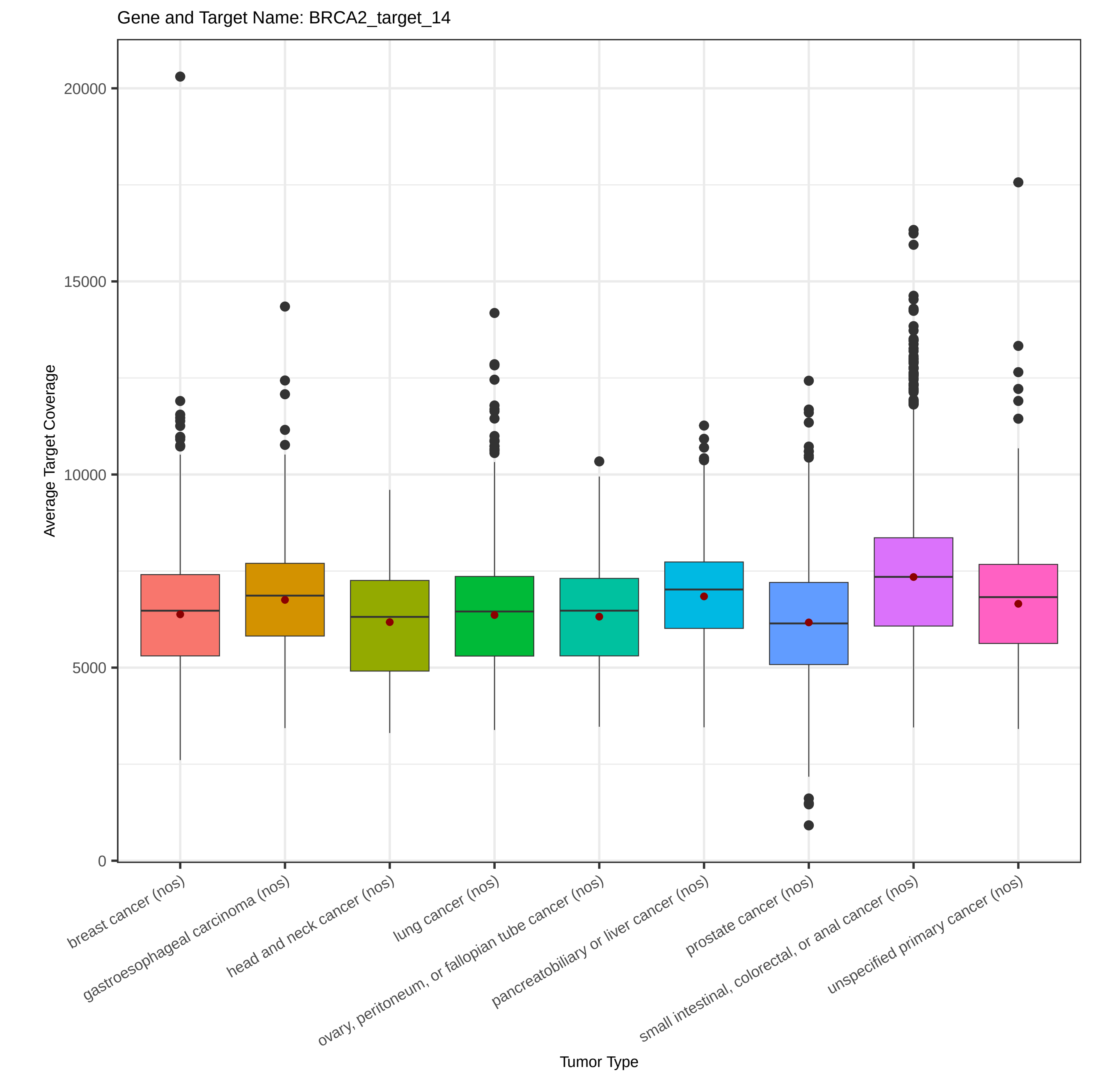
Gene and Target Name: BRCA2_target_14
Average Target Coverage
20000
15000
10000
5000
0
breast cancer (nos)
gastroesophageal carcinoma (nos)
head and neck cancer (nos)
lung cancer (nos)
ovary, peritoneum, or fallopian tube cancer (nos)
pancreatobiliary or liver cancer (nos)
prostate cancer (nos)
small intestinal, colorectal, or anal cancer (nos)
unspecified primary cancer (nos)
Tumor Type

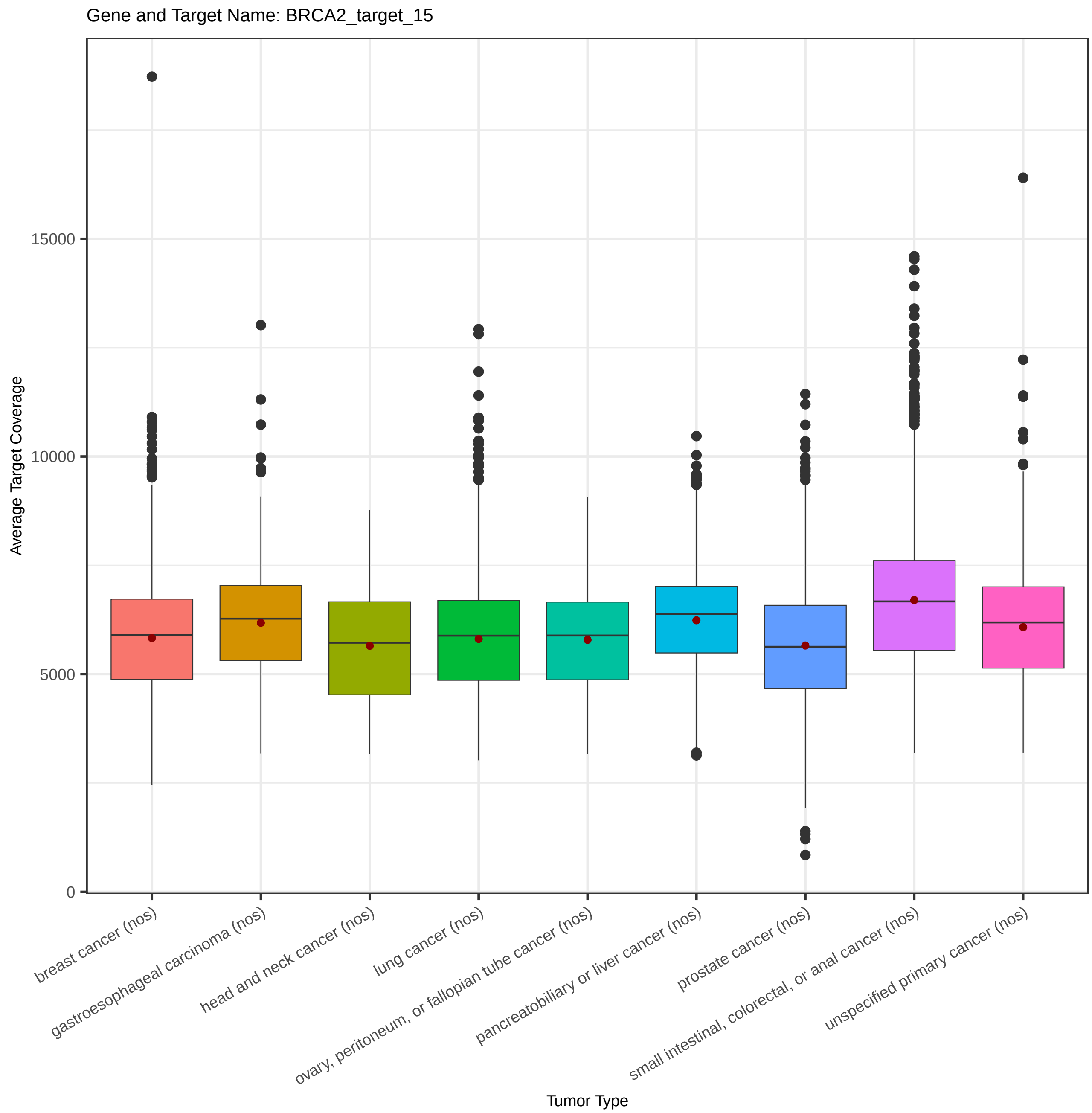

Gene and Target Name: BRCA2_target_15
Average Target Coverage
0
5000
10000
15000
breast cancer (nos)
gastroesophageal carcinoma (nos)
head and neck cancer (nos)
lung cancer (nos)
ovary, peritoneum, or fallopian tube cancer (nos)
pancreatobiliary or liver cancer (nos)
prostate cancer (nos)
small intestinal, colorectal, or anal cancer (nos)
unspecified primary cancer (nos)
Tumor Type

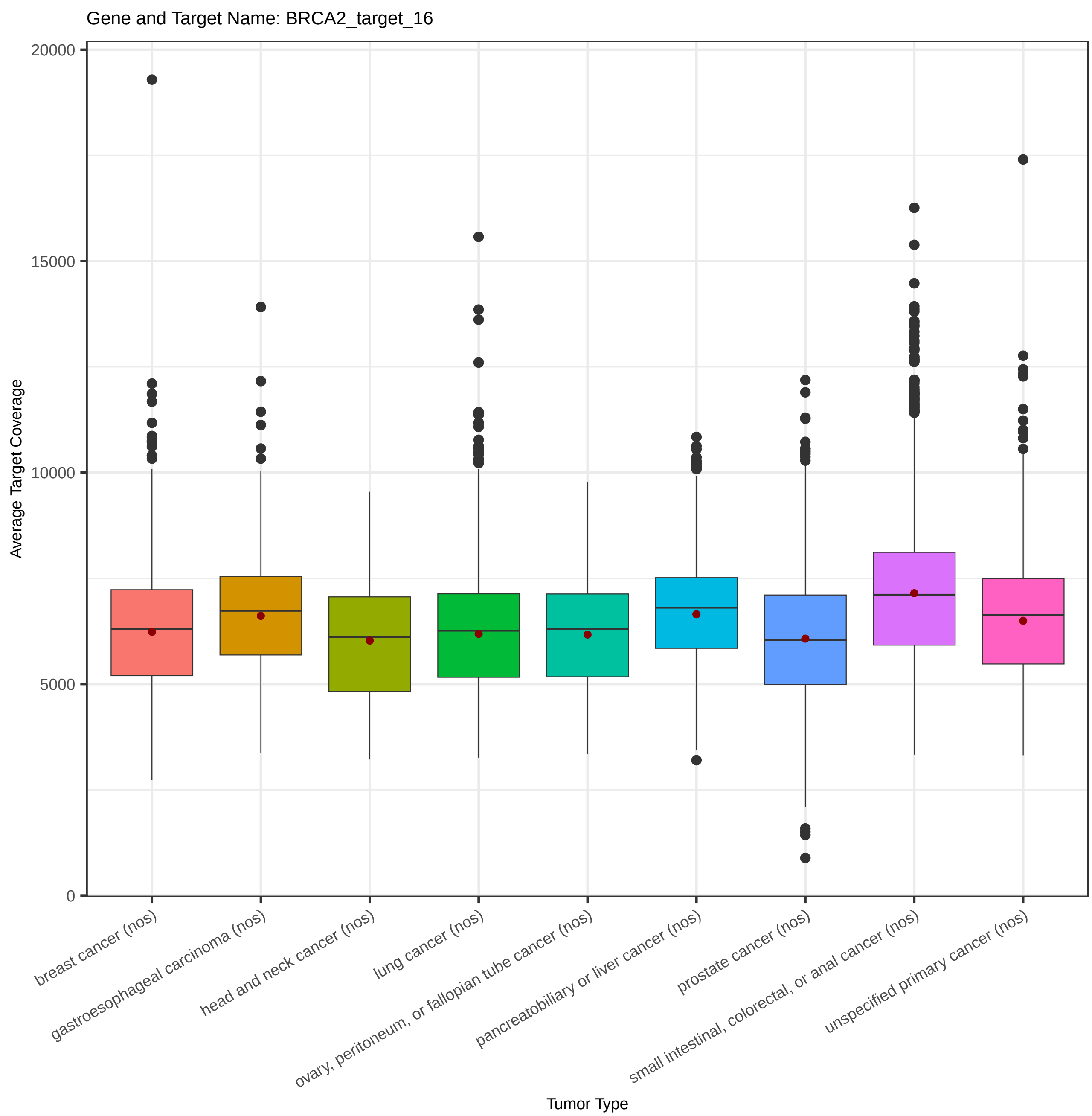
Gene and Target Name: BRCA2_target_16
Average Target Coverage
20000
15000
10000
5000
0
breast cancer (nos)
gastroesophageal carcinoma (nos)
head and neck cancer (nos)
lung cancer (nos)
ovary, peritoneum, or fallopian tube cancer (nos)
pancreatobiliary or liver cancer (nos)
prostate cancer (nos)
small intestinal, colorectal, or anal cancer (nos)
unspecified primary cancer (nos)
Tumor Type

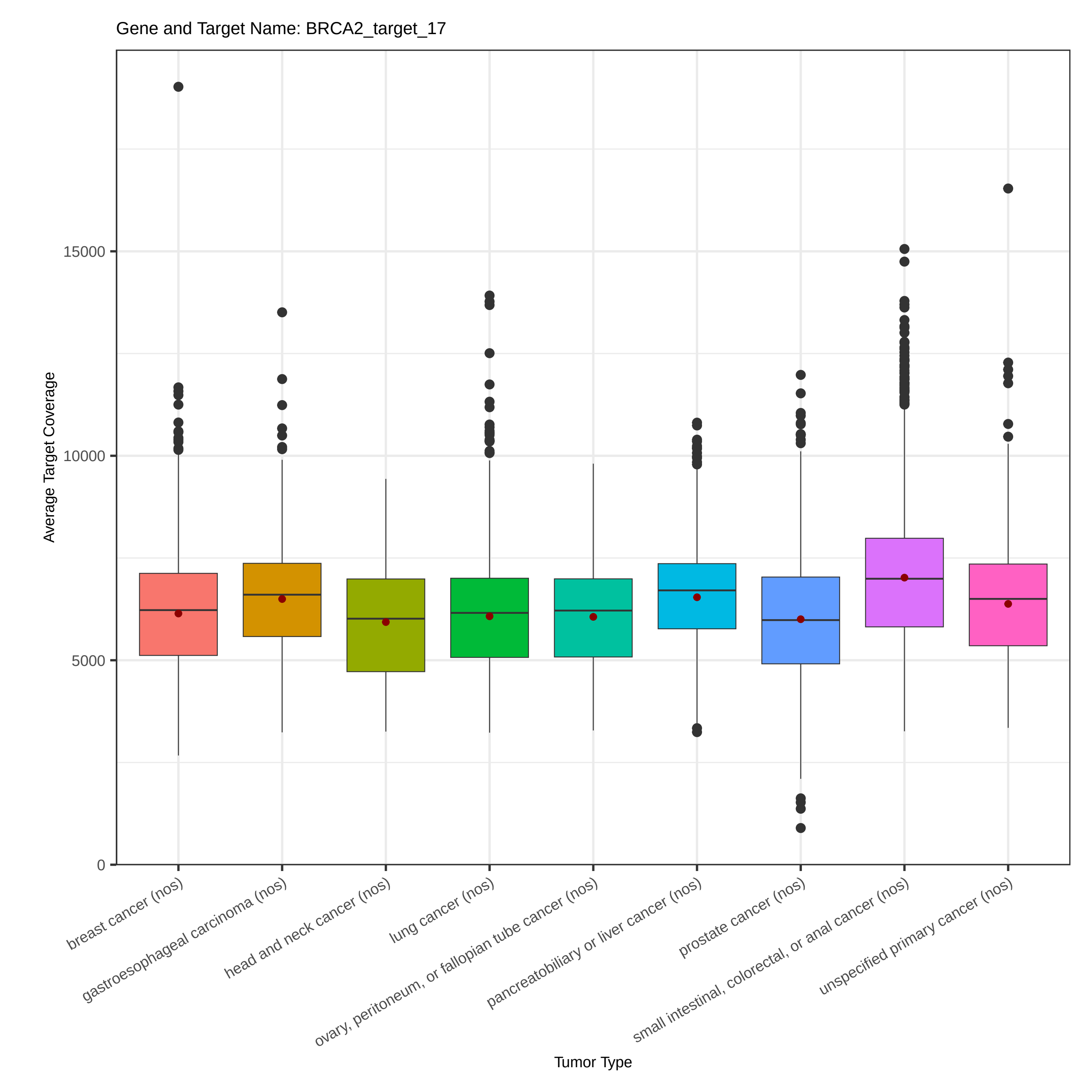

Gene and Target Name: BRCA2_target_17
Average Target Coverage
15000
10000
5000
0
breast cancer (nos)
gastroesophageal carcinoma (nos)
head and neck cancer (nos)
lung cancer (nos)
ovary, peritoneum, or fallopian tube cancer (nos)
pancreatobiliary or liver cancer (nos)
prostate cancer (nos)
small intestinal, colorectal, or anal cancer (nos)
unspecified primary cancer (nos)
Tumor Type

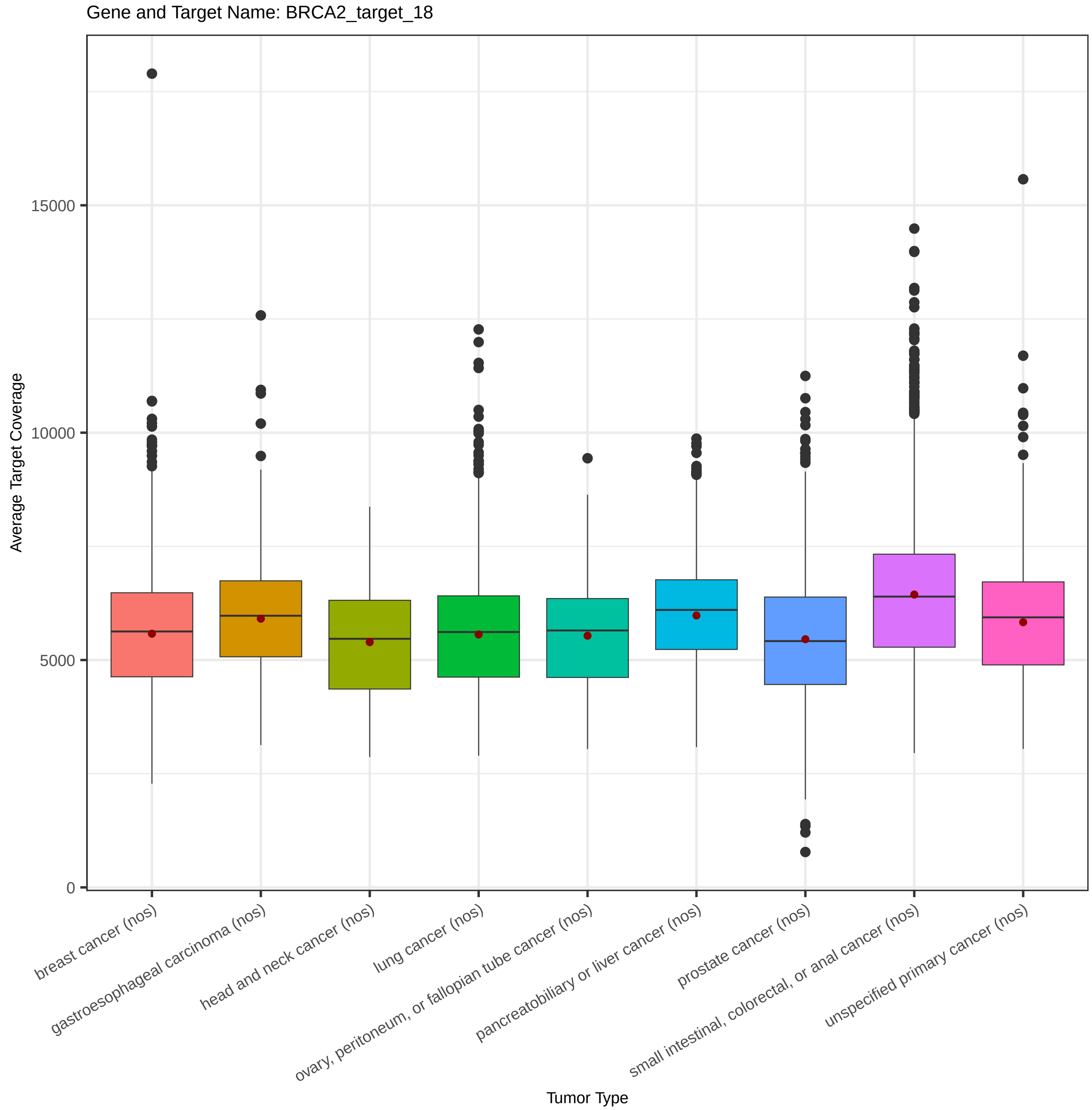

Gene and Target Name: BRCA2_target_18
Average Target Coverage
15000
10000
5000
0
breast cancer (nos)
gastroesophageal carcinoma (nos)
head and neck cancer (nos)
lung cancer (nos)
ovary, peritoneum, or fallopian tube cancer (nos)
pancreatobiliary or liver cancer (nos)
prostate cancer (nos)
small intestinal, colorectal, or anal cancer (nos)
unspecified primary cancer (nos)
Tumor Type

Gene and Target Name: BRCA2_target_19

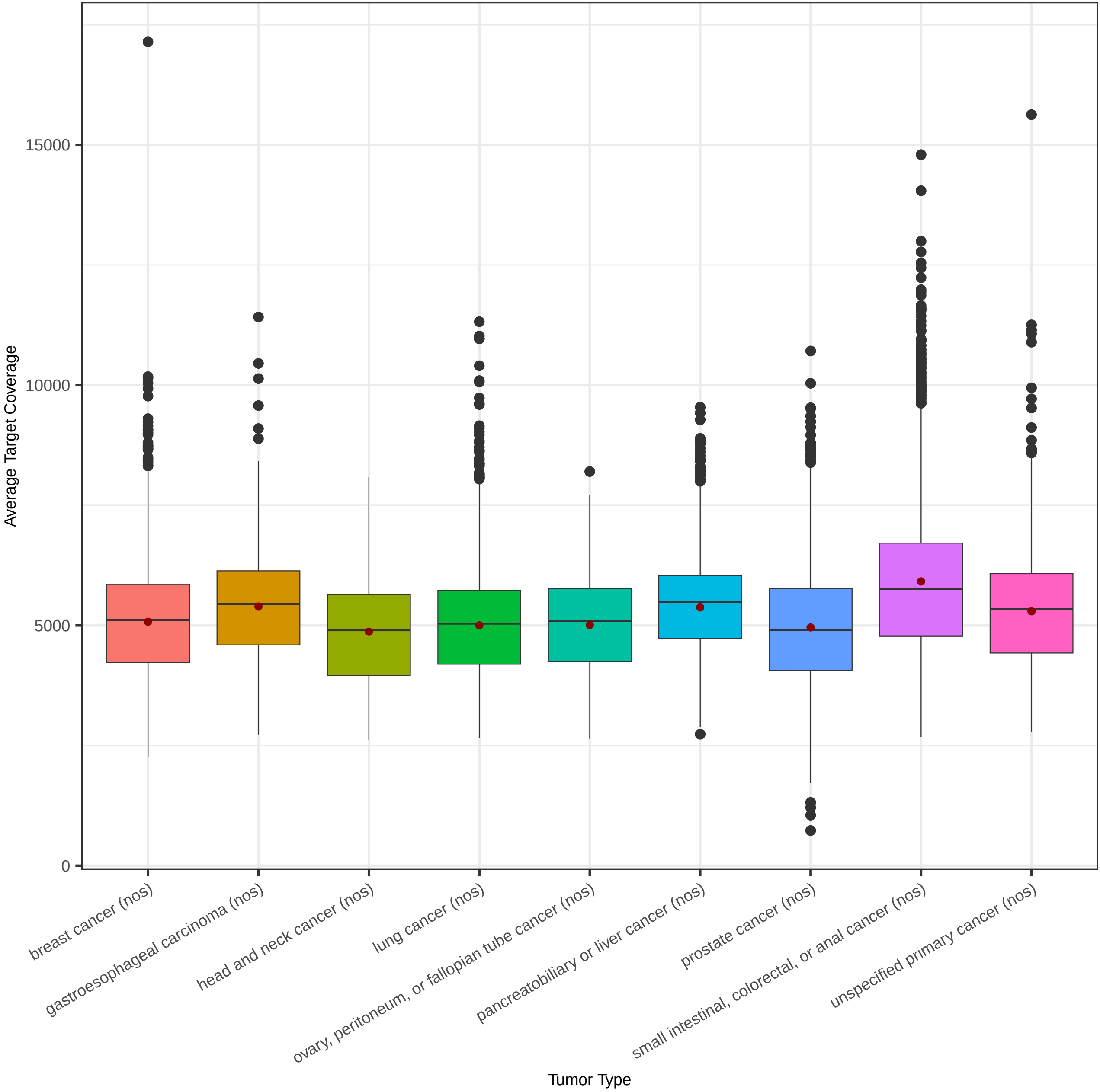

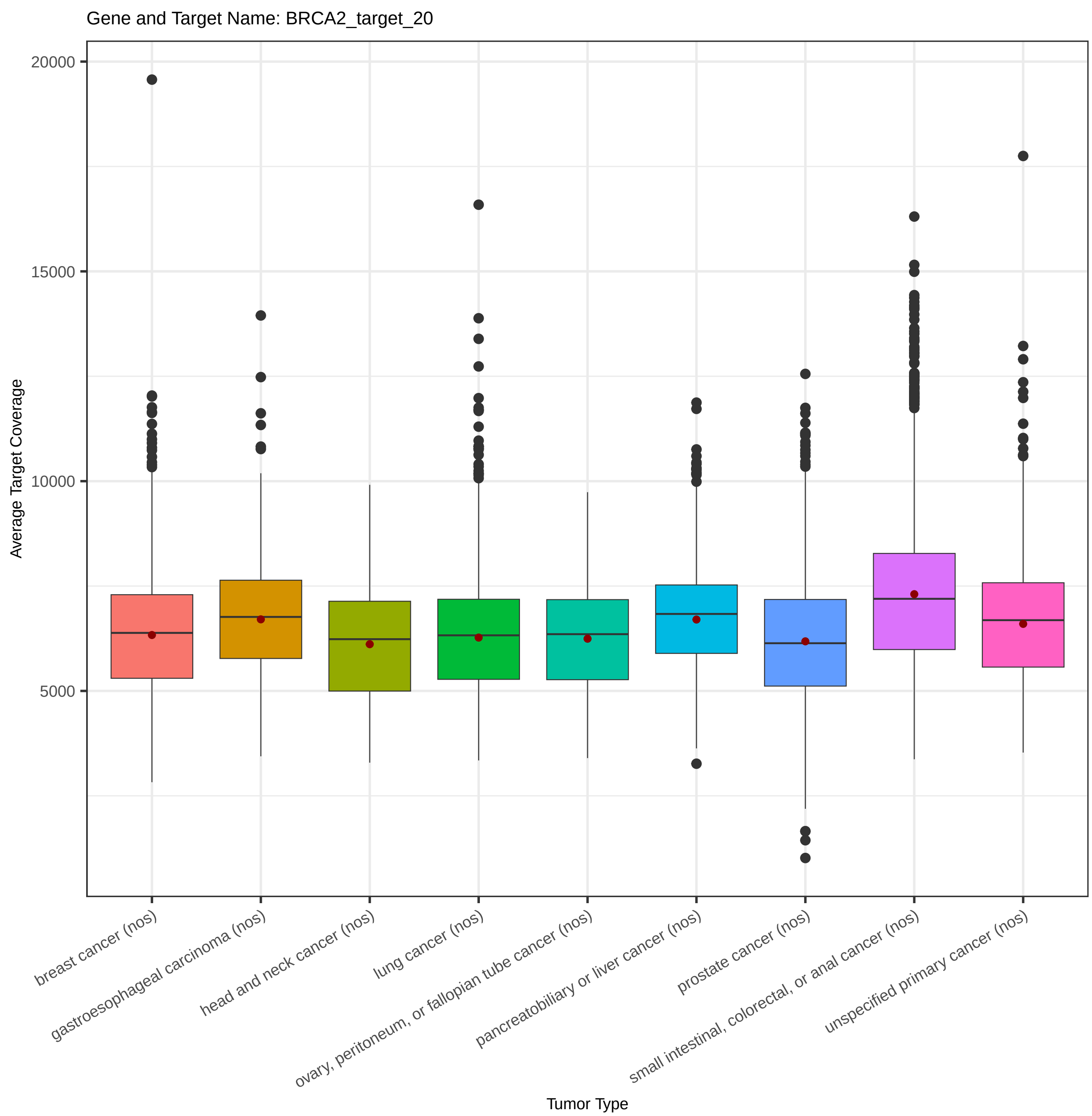
Gene and Target Name: BRCA2_target_20
Average Target Coverage
20000
15000
10000
5000
Tumor Type
breast cancer (nos)
gastroesophageal carcinoma (nos)
head and neck cancer (nos)
lung cancer (nos)
ovary, peritoneum, or fallopian tube cancer (nos)
pancreatobiliary or liver cancer (nos)
prostate cancer (nos)
small intestinal, colorectal, or anal cancer (nos)
unspecified primary cancer (nos)

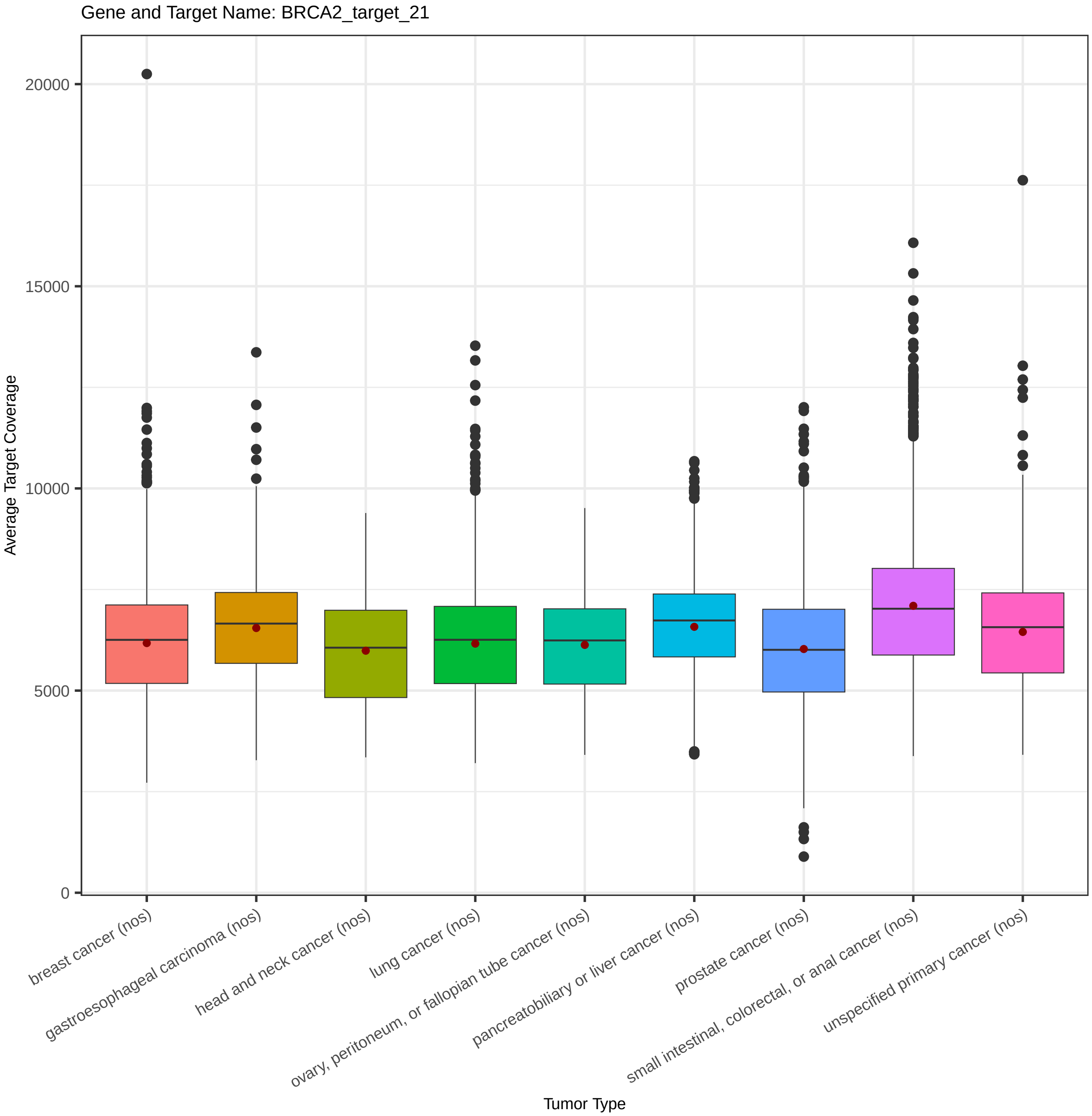
Gene and Target Name: BRCA2_target_21
Average Target Coverage
20000
15000
10000
5000
0
breast cancer (nos)
gastroesophageal carcinoma (nos)
head and neck cancer (nos)
lung cancer (nos)
ovary, peritoneum, or fallopian tube cancer (nos)
pancreatobiliary or liver cancer (nos)
prostate cancer (nos)
small intestinal, colorectal, or anal cancer (nos)
unspecified primary cancer (nos)
Tumor Type

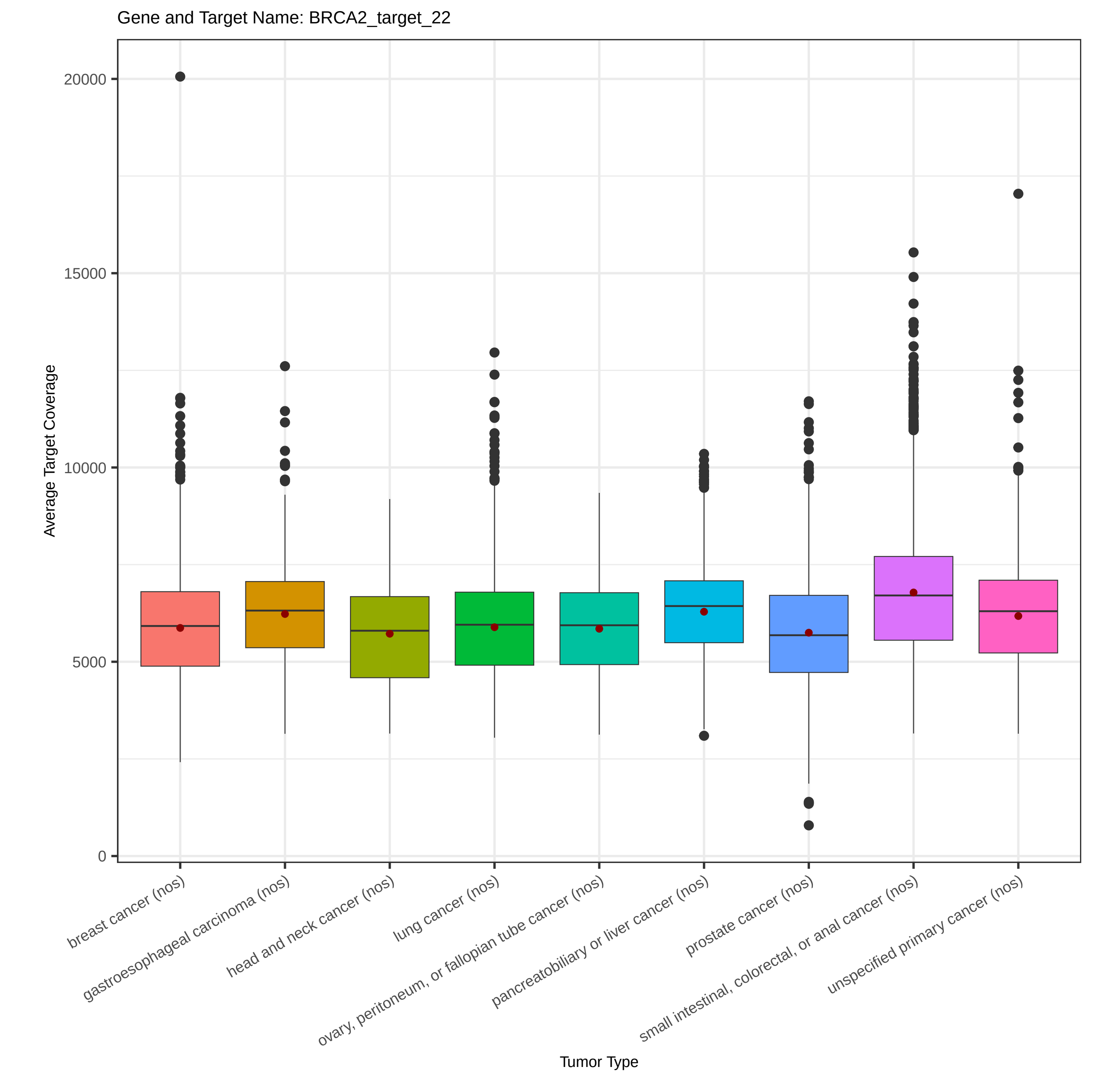
Gene and Target Name: BRCA2_target_22
Average Target Coverage
20000
15000
10000
5000
0
breast cancer (nos)
gastroesophageal carcinoma (nos)
head and neck cancer (nos)
lung cancer (nos)
ovary, peritoneum, or fallopian tube cancer (nos)
pancreatobiliary or liver cancer (nos)
prostate cancer (nos)
small intestinal, colorectal, or anal cancer (nos)
unspecified primary cancer (nos)
Tumor Type

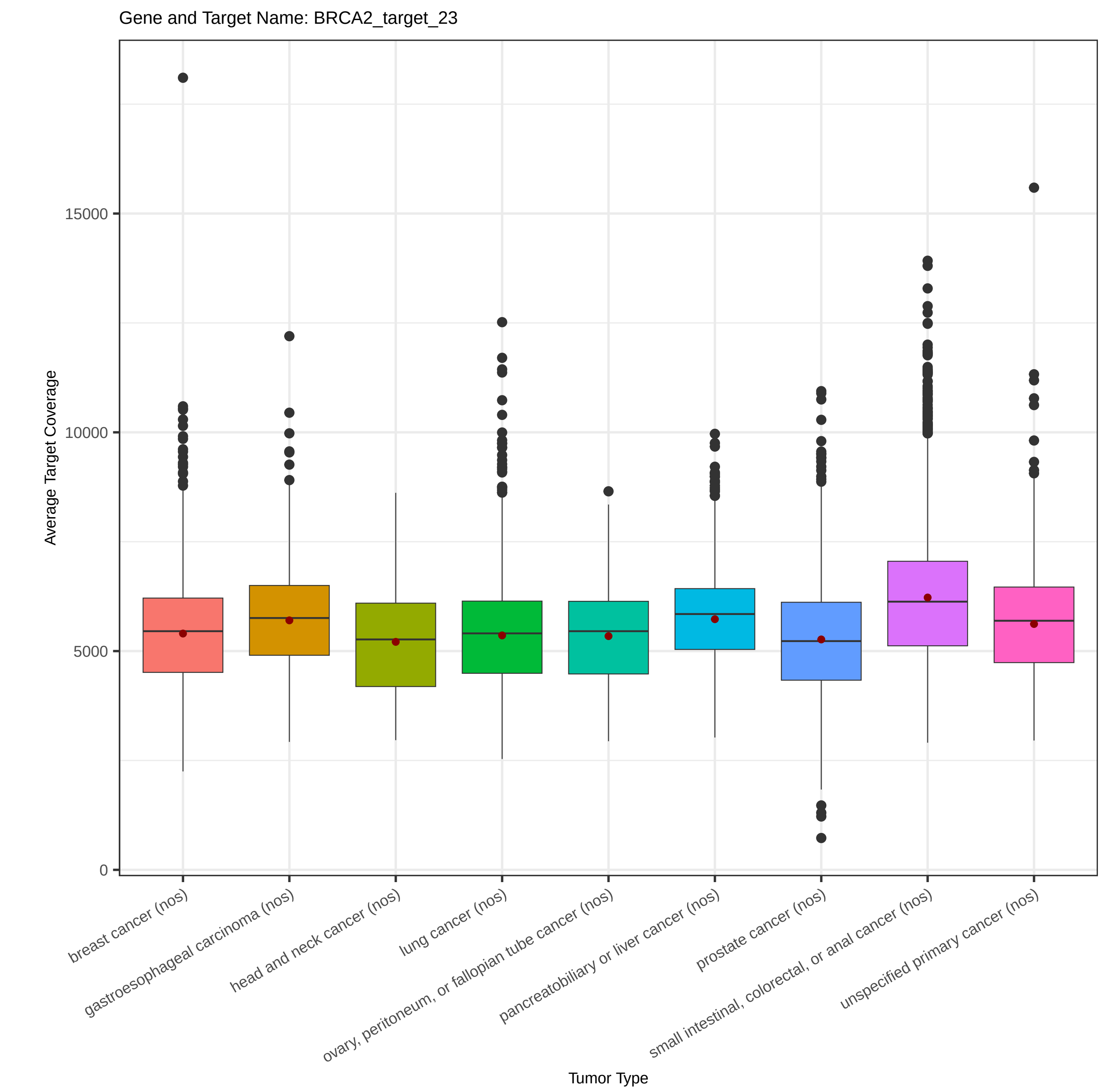

Gene and Target Name: BRCA2_target_23
Average Target Coverage
0
5000
10000
15000
breast cancer (nos)
gastroesophageal carcinoma (nos)
head and neck cancer (nos)
lung cancer (nos)
ovary, peritoneum, or fallopian tube cancer (nos)
pancreatobiliary or liver cancer (nos)
prostate cancer (nos)
small intestinal, colorectal, or anal cancer (nos)
unspecified primary cancer (nos)
Tumor Type

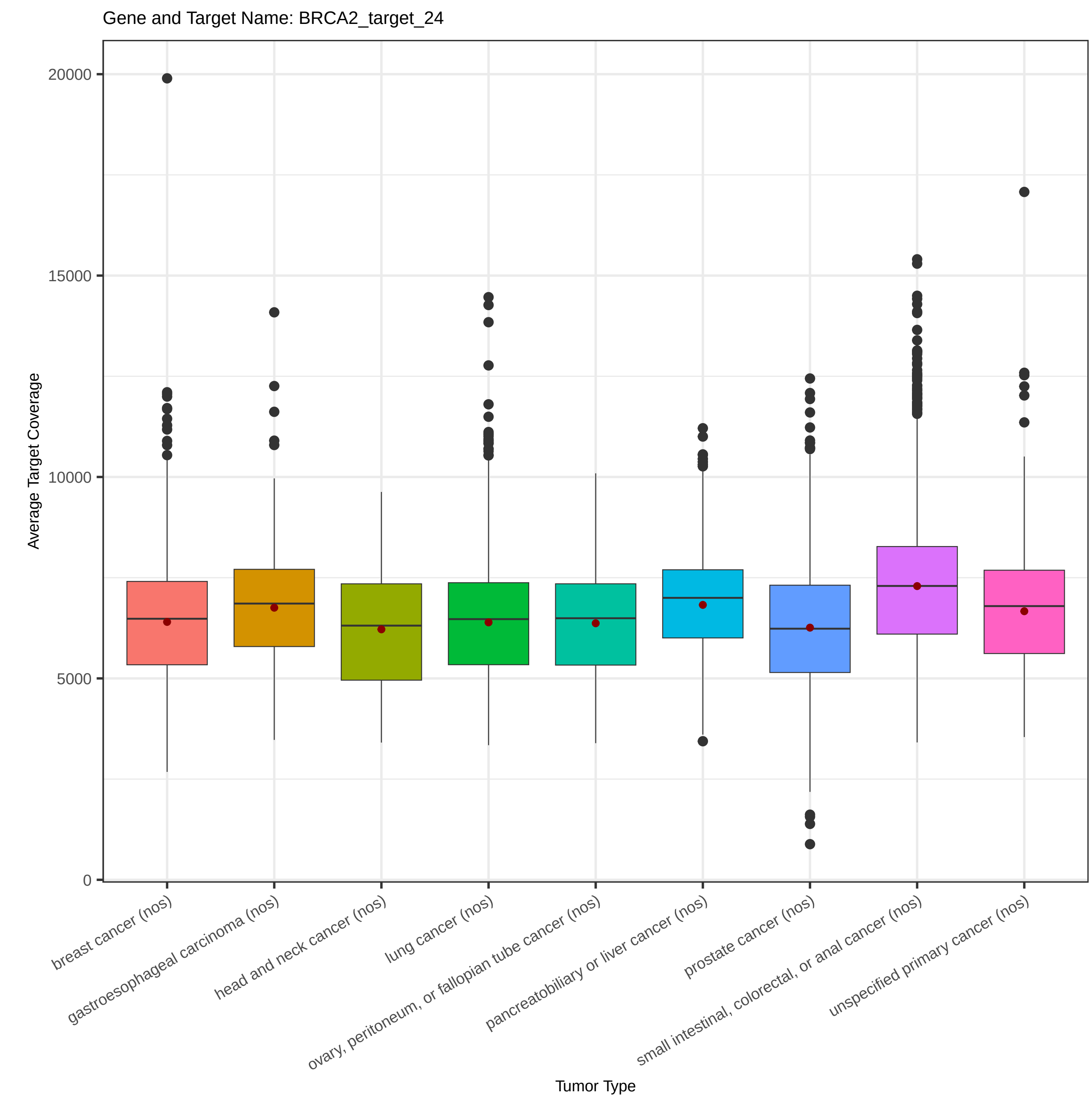

Gene and Target Name: BRCA2_target_24
Average Target Coverage
20000
15000
10000
5000
0
breast cancer (nos)
gastroesophageal carcinoma (nos)
head and neck cancer (nos)
lung cancer (nos)
ovary, peritoneum, or fallopian tube cancer (nos)
pancreatobiliary or liver cancer (nos)
prostate cancer (nos)
small intestinal, colorectal, or anal cancer (nos)
unspecified primary cancer (nos)
Tumor Type

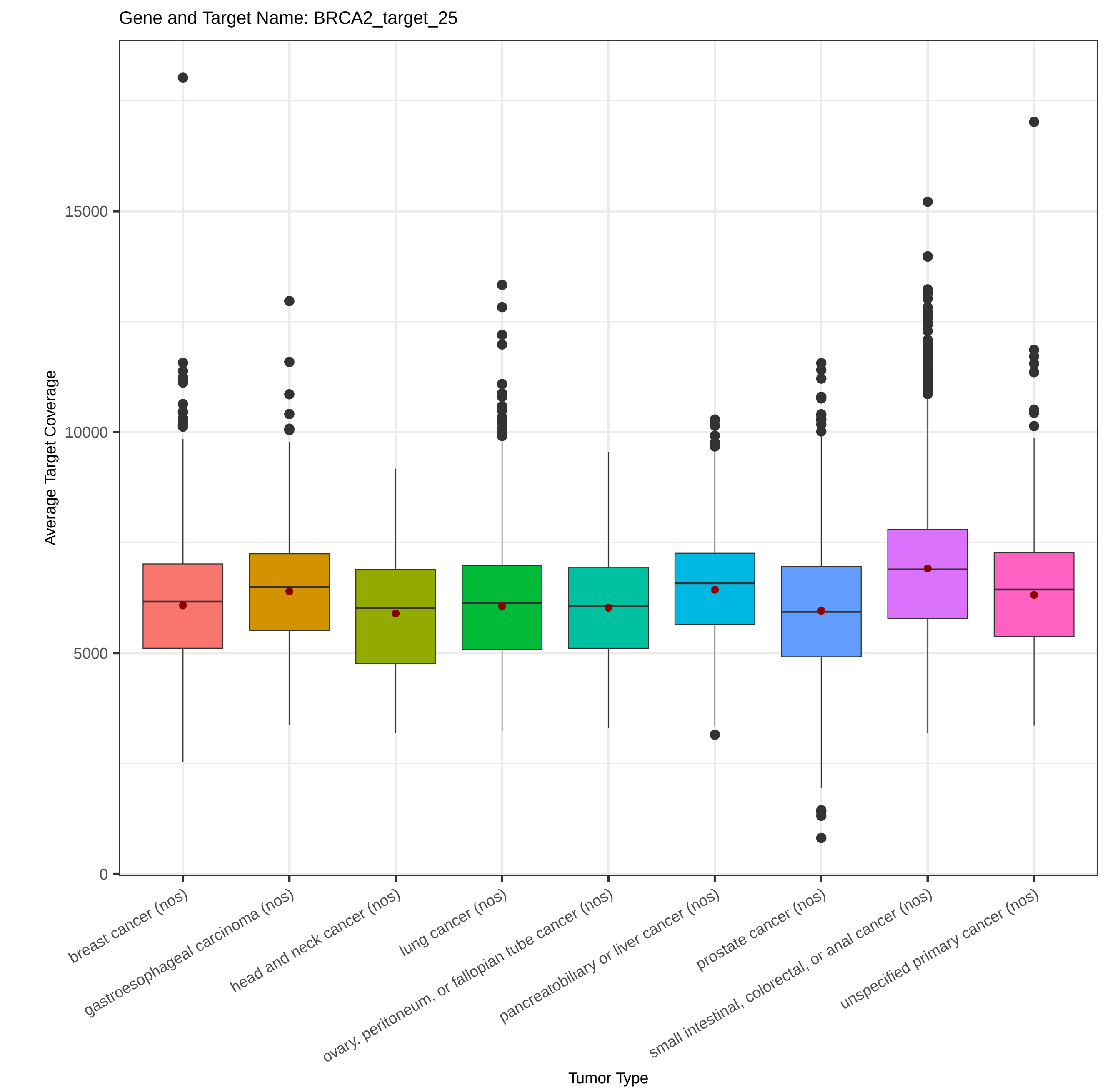
Gene and Target Name: BRCA2_target_25
Average Target Coverage
15000
10000
5000
0
breast cancer (nos)
gastroesophageal carcinoma (nos)
head and neck cancer (nos)
lung cancer (nos)
ovary, peritoneum, or fallopian tube cancer (nos)
pancreatobiliary or liver cancer (nos)
prostate cancer (nos)
small intestinal, colorectal, or anal cancer (nos)
unspecified primary cancer (nos)
Tumor Type

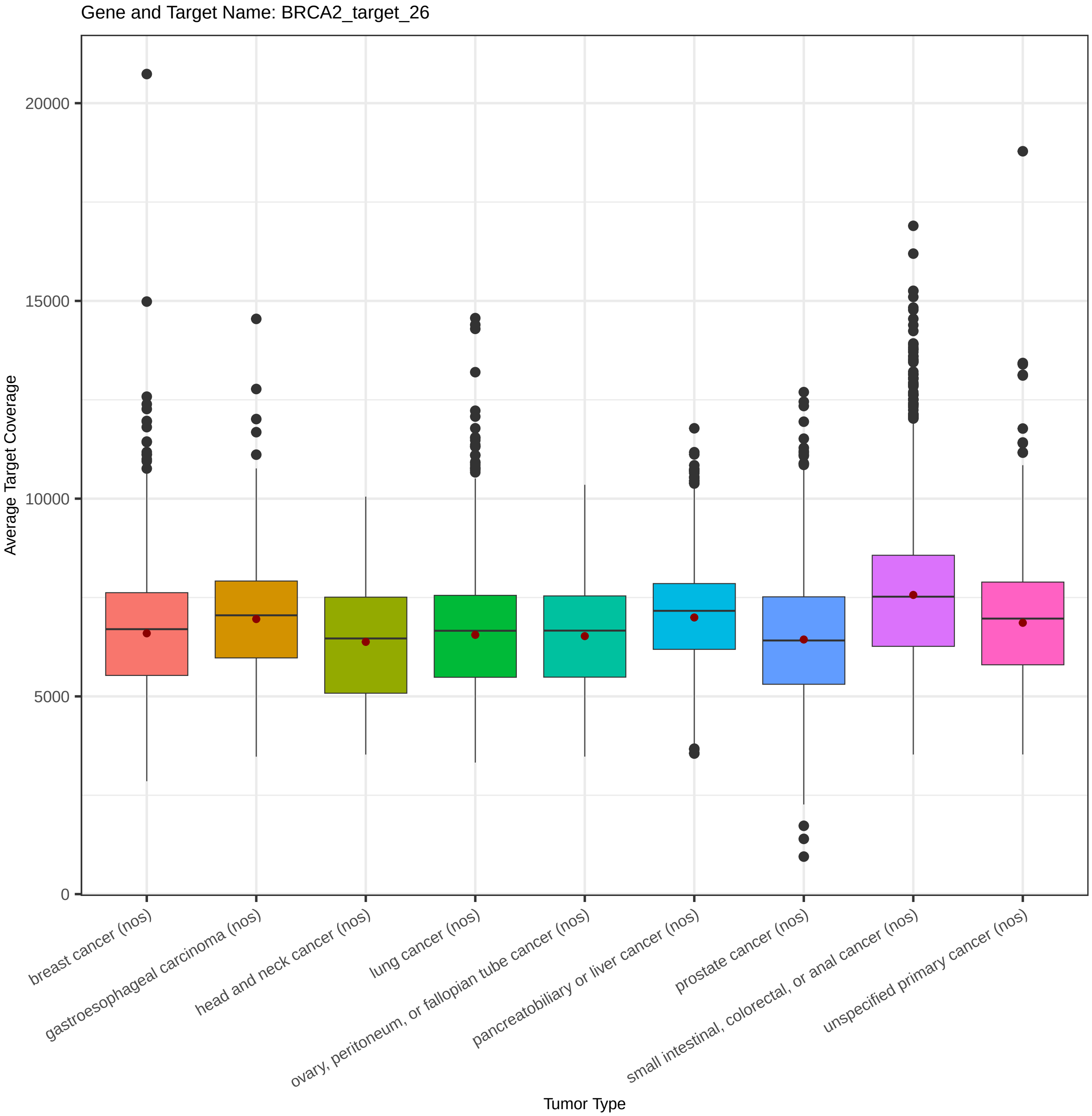

Gene and Target Name: BRCA2_target_26
Average Target Coverage
20000
15000
10000
5000
0
breast cancer (nos)
gastroesophageal carcinoma (nos)
head and neck cancer (nos)
lung cancer (nos)
ovary, peritoneum, or fallopian tube cancer (nos)
pancreatobiliary or liver cancer (nos)
prostate cancer (nos)
small intestinal, colorectal, or anal cancer (nos)
unspecified primary cancer (nos)
Tumor Type

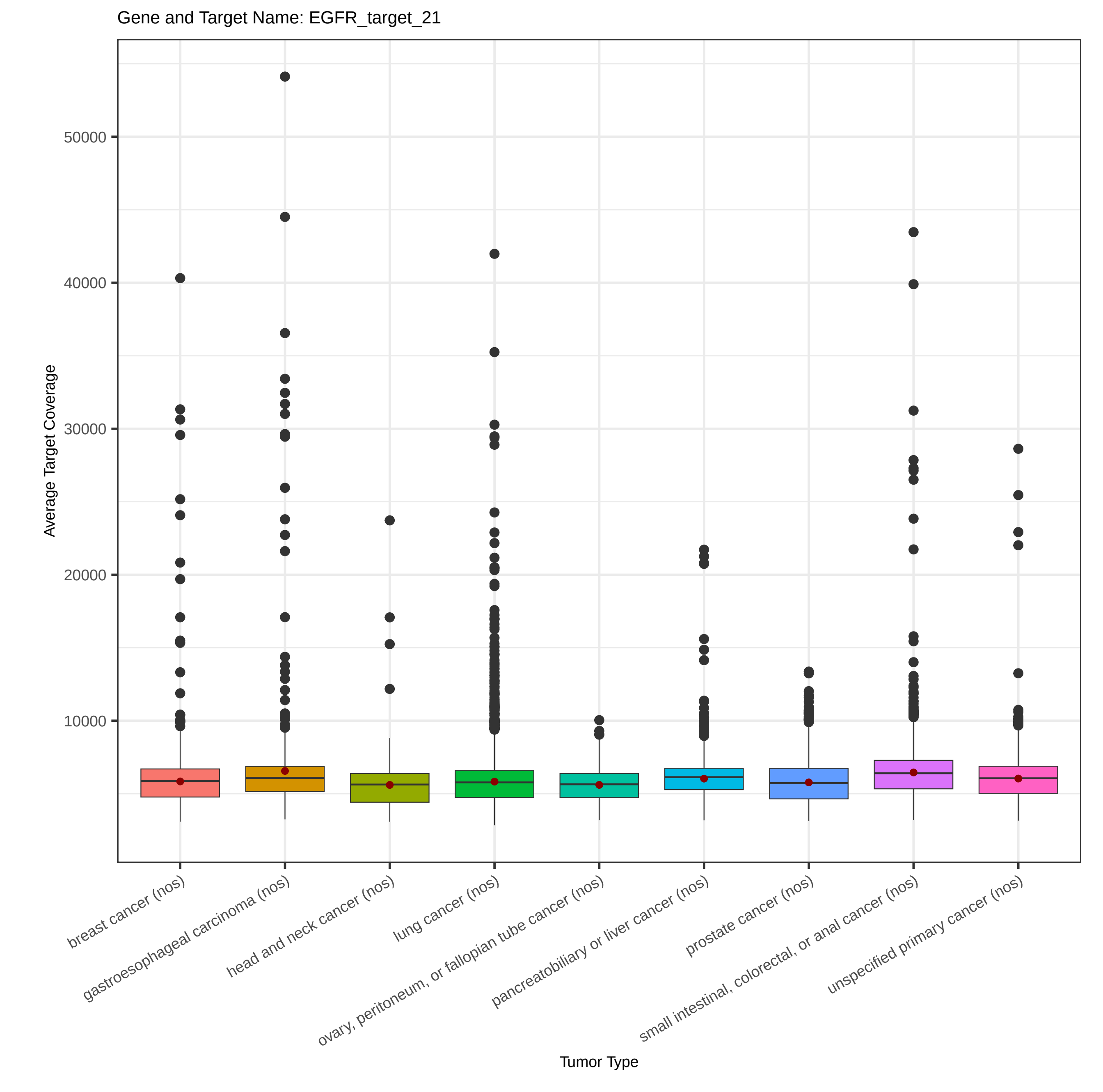
Gene and Target Name: EGFR_target_21
Average Target Coverage
50000
40000
30000
20000
10000
breast cancer (nos)
gastroesophageal carcinoma (nos)
head and neck cancer (nos)
lung cancer (nos)
ovary, peritoneum, or fallopian tube cancer (nos)
pancreatobiliary or liver cancer (nos)
prostate cancer (nos)
small intestinal, colorectal, or anal cancer (nos)
unspecified primary cancer (nos)
Tumor Type

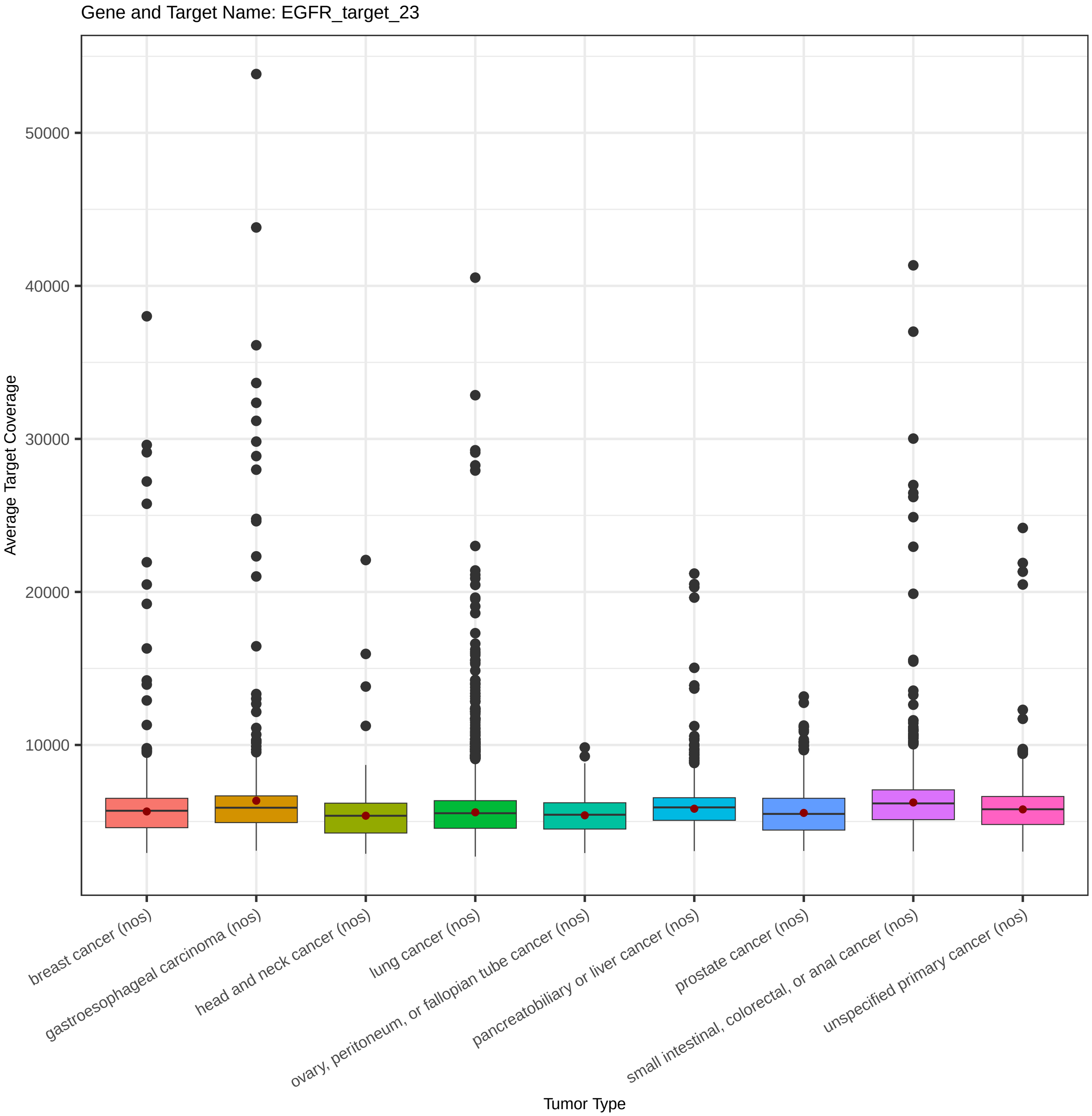

Gene and Target Name: EGFR_target_23
Average Target Coverage
50000
40000
30000
20000
10000
breast cancer (nos)
gastroesophageal carcinoma (nos)
head and neck cancer (nos)
lung cancer (nos)
ovary, peritoneum, or fallopian tube cancer (nos)
pancreatobiliary or liver cancer (nos)
prostate cancer (nos)
small intestinal, colorectal, or anal cancer (nos)
unspecified primary cancer (nos)
Tumor Type

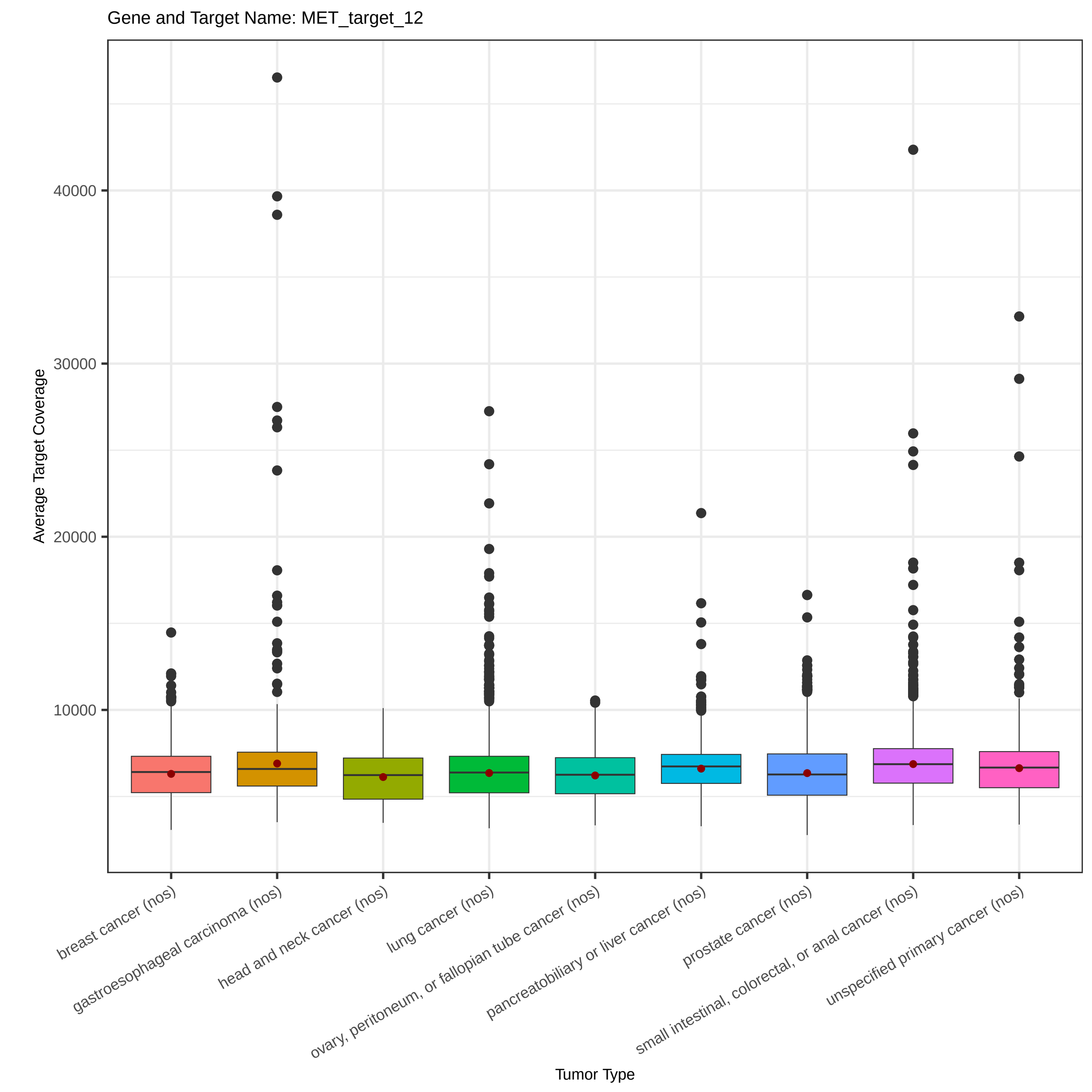

Gene and Target Name: MET_target_12
Average Target Coverage
40000
30000
20000
10000
breast cancer (nos)
gastroesophageal carcinoma (nos)
head and neck cancer (nos)
lung cancer (nos)
ovary, peritoneum, or fallopian tube cancer (nos)
pancreatobiliary or liver cancer (nos)
prostate cancer (nos)
small intestinal, colorectal, or anal cancer (nos)
unspecified primary cancer (nos)
Tumor Type

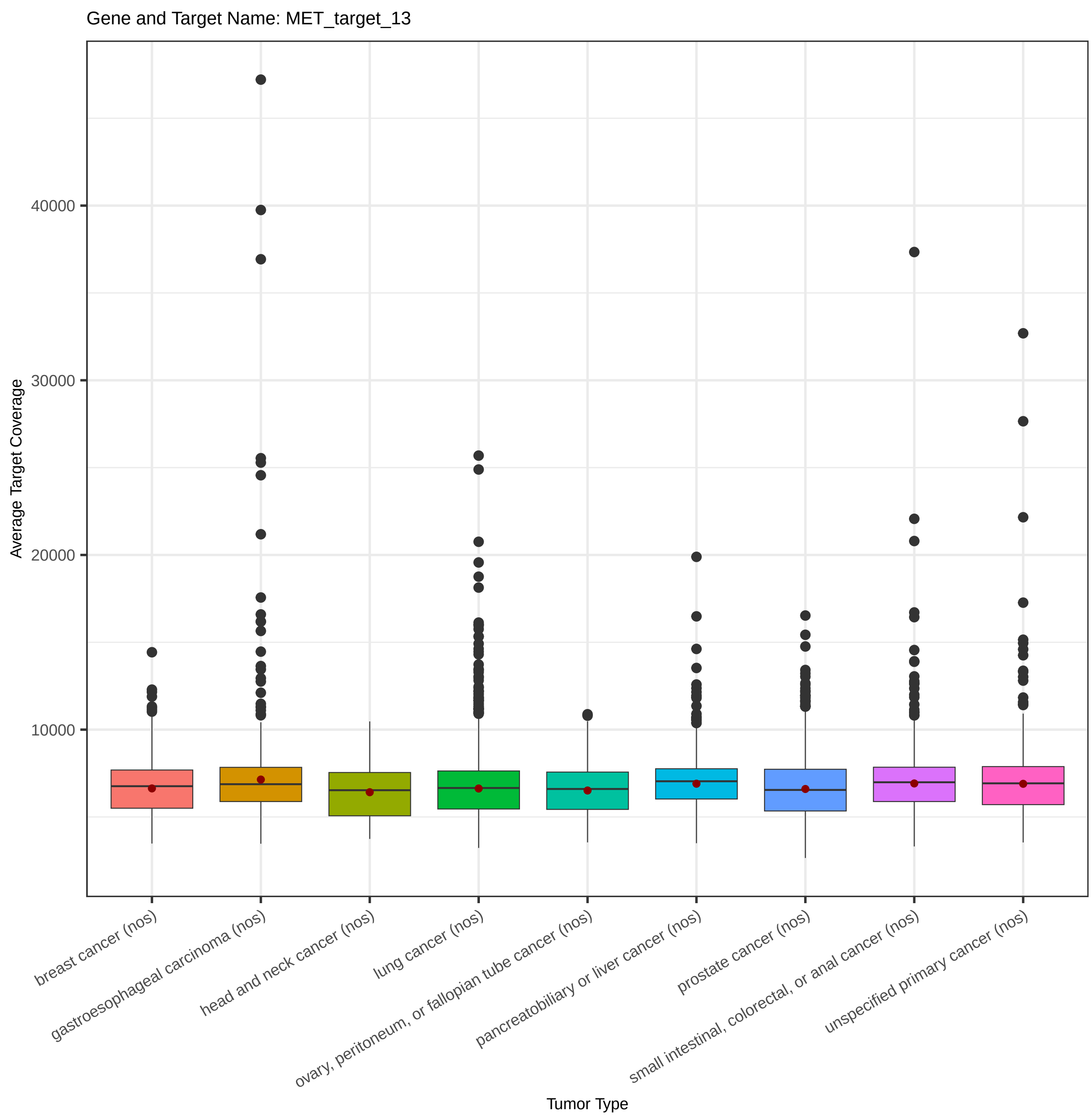

Gene and Target Name: MET_target_13
Average Target Coverage
40000
30000
20000
10000
breast cancer (nos)
gastroesophageal carcinoma (nos)
head and neck cancer (nos)
lung cancer (nos)
ovary, peritoneum, or fallopian tube cancer (nos)
pancreatobiliary or liver cancer (nos)
prostate cancer (nos)
small intestinal, colorectal, or anal cancer (nos)
unspecified primary cancer (nos)
Tumor Type

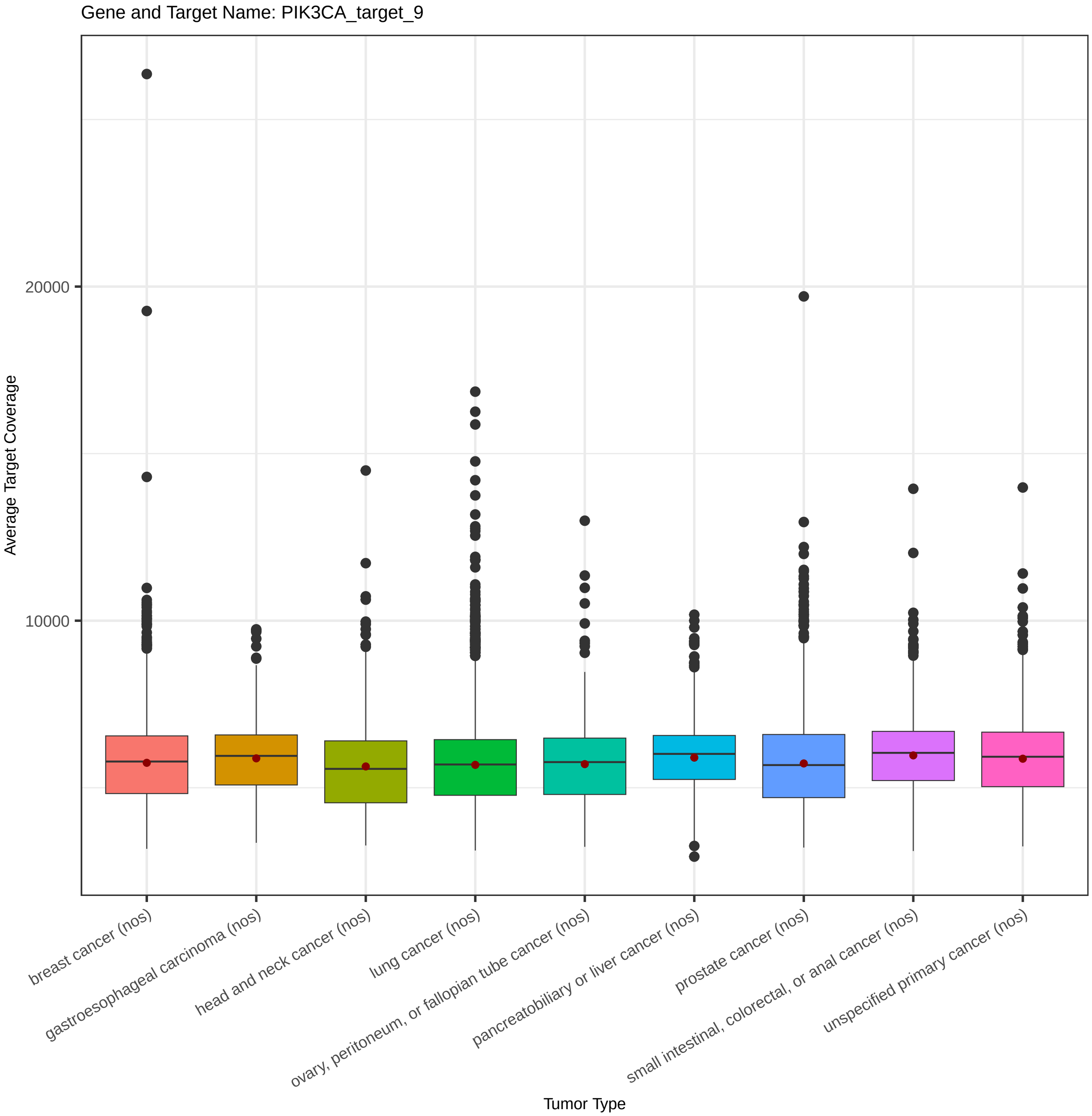
Gene and Target Name: PIK3CA_target_9
Average Target Coverage
20000
10000
breast cancer (nos)
gastroesophageal carcinoma (nos)
head and neck cancer (nos)
lung cancer (nos)
ovary, peritoneum, or fallopian tube cancer (nos)
pancreatobiliary or liver cancer (nos)
prostate cancer (nos)
small intestinal, colorectal, or anal cancer (nos)
unspecified primary cancer (nos)
Tumor Type

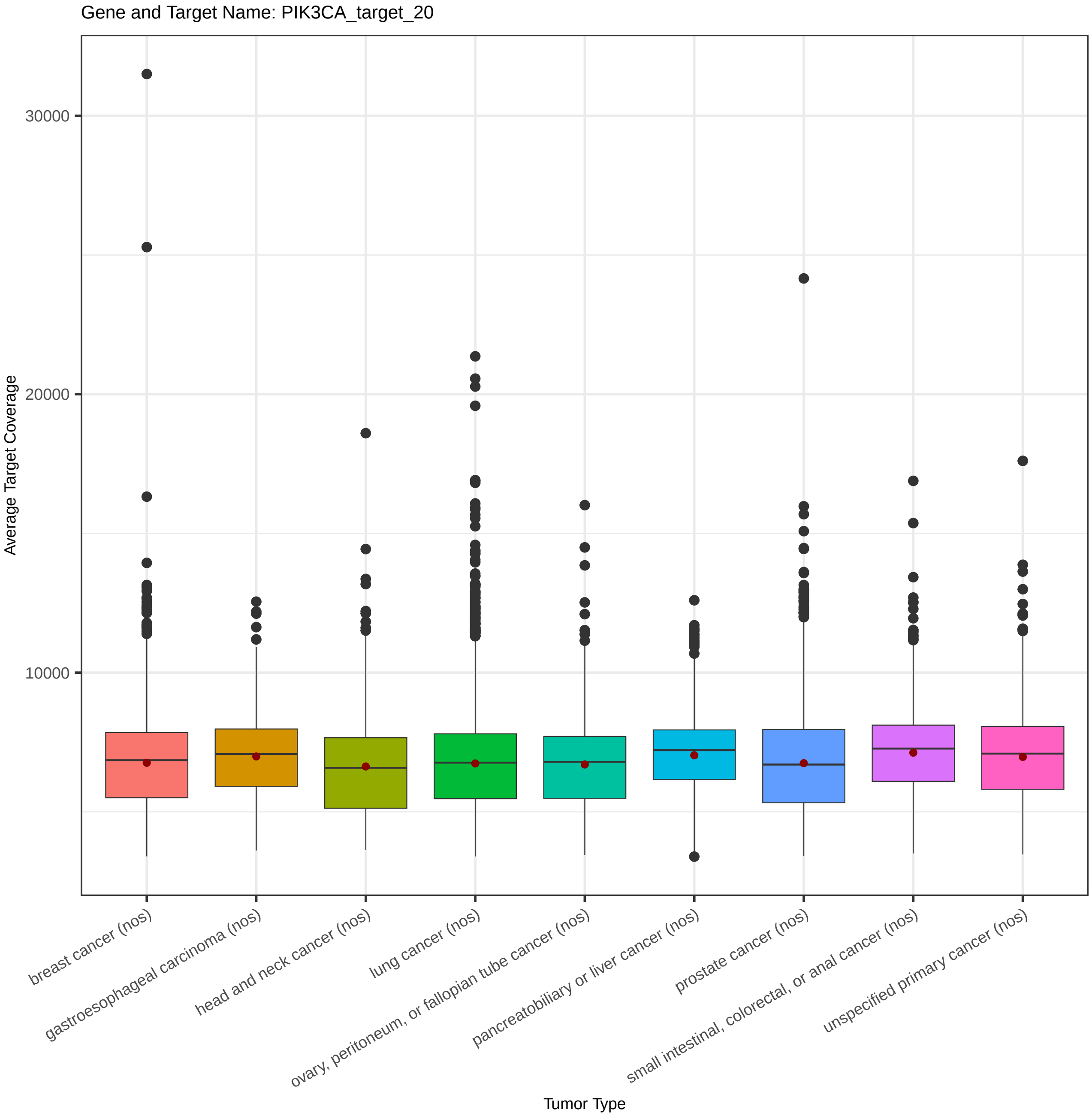
Gene and Target Name: PIK3CA_target_20
Average Target Coverage
30000
20000
10000
Tumor Type
breast cancer (nos)
gastroesophageal carcinoma (nos)
head and neck cancer (nos)
lung cancer (nos)
ovary, peritoneum, or fallopian tube cancer (nos)
pancreatobiliary or liver cancer (nos)
prostate cancer (nos)
small intestinal, colorectal, or anal cancer (nos)
unspecified primary cancer (nos)

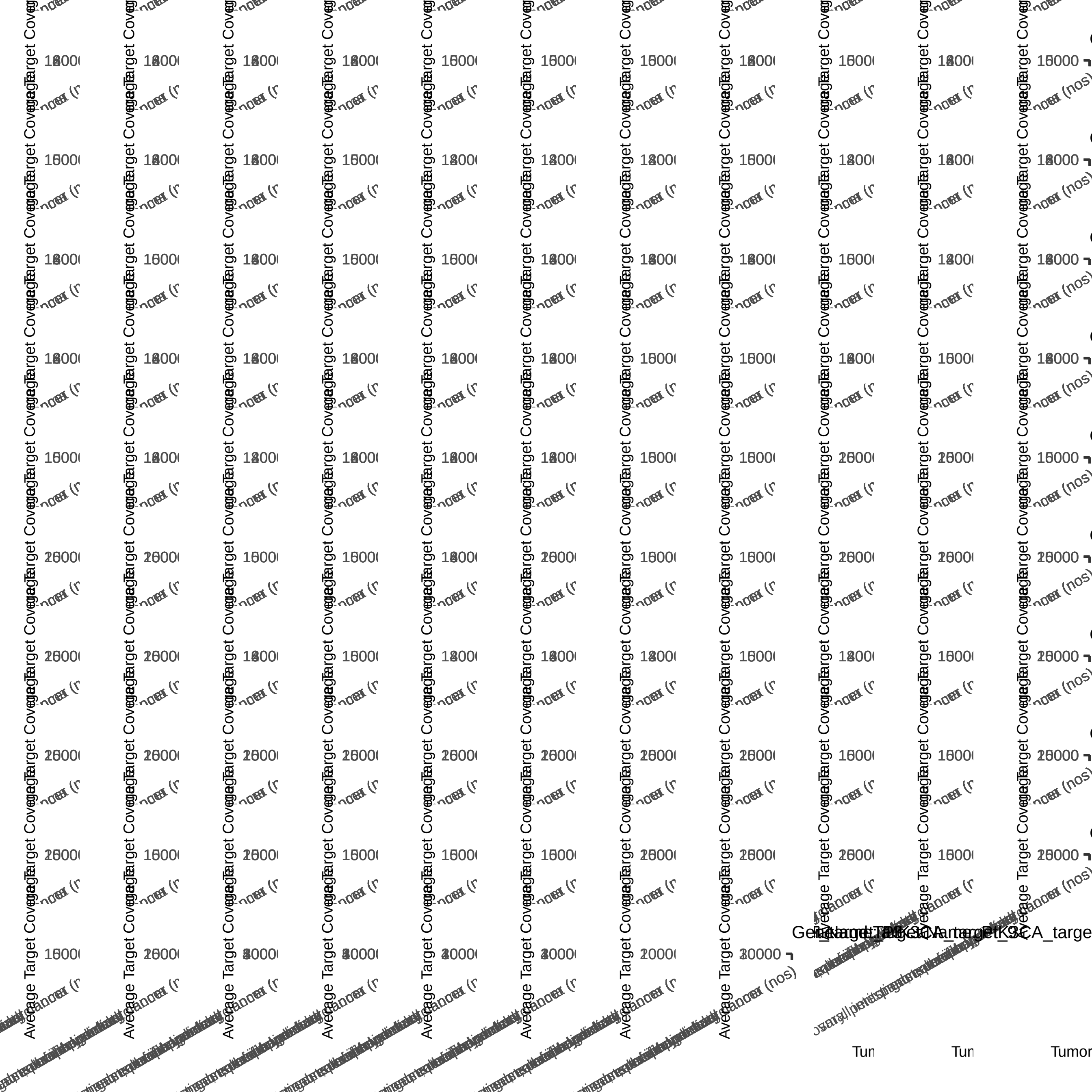

Supplement: S2 File — (ZIP) [file pone.0329392.s002.zip › Supplement Figures/S4 Fig.pdf]
