# Supplementary material for: Analytical validation and sequencing coverage studies suggest that performance of a liquid biopsy assay is tumor agnostic (DNA-is-DNA)
Source: PLoS One. 2025 Aug 1;20(8):e0329392. doi: 10.1371/journal.pone.0329392 (PMC12316276; doi:10.1371/journal.pone.0329392)
Supplement: S2 File — (ZIP) [file pone.0329392.s002.zip › Supplement Figures/S5 Fig.pdf]

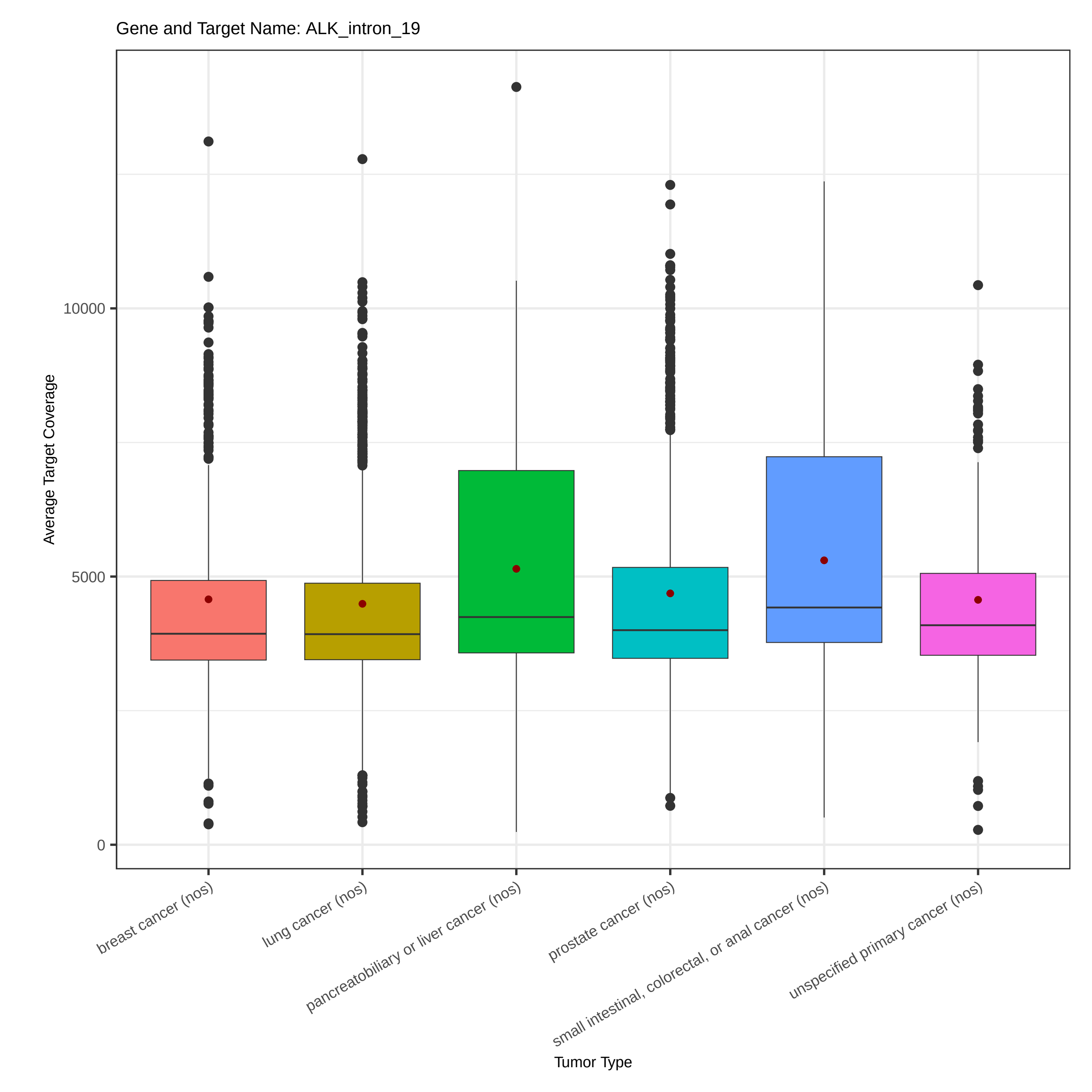
Gene and Target Name: ALK_intron_19
Average Target Coverage
10000
5000
0
breast cancer (nos)
lung cancer (nos)
pancreatobiliary or liver cancer (nos)
prostate cancer (nos)
small intestinal, colorectal, or anal cancer (nos)
unspecified primary cancer (nos)
Tumor Type

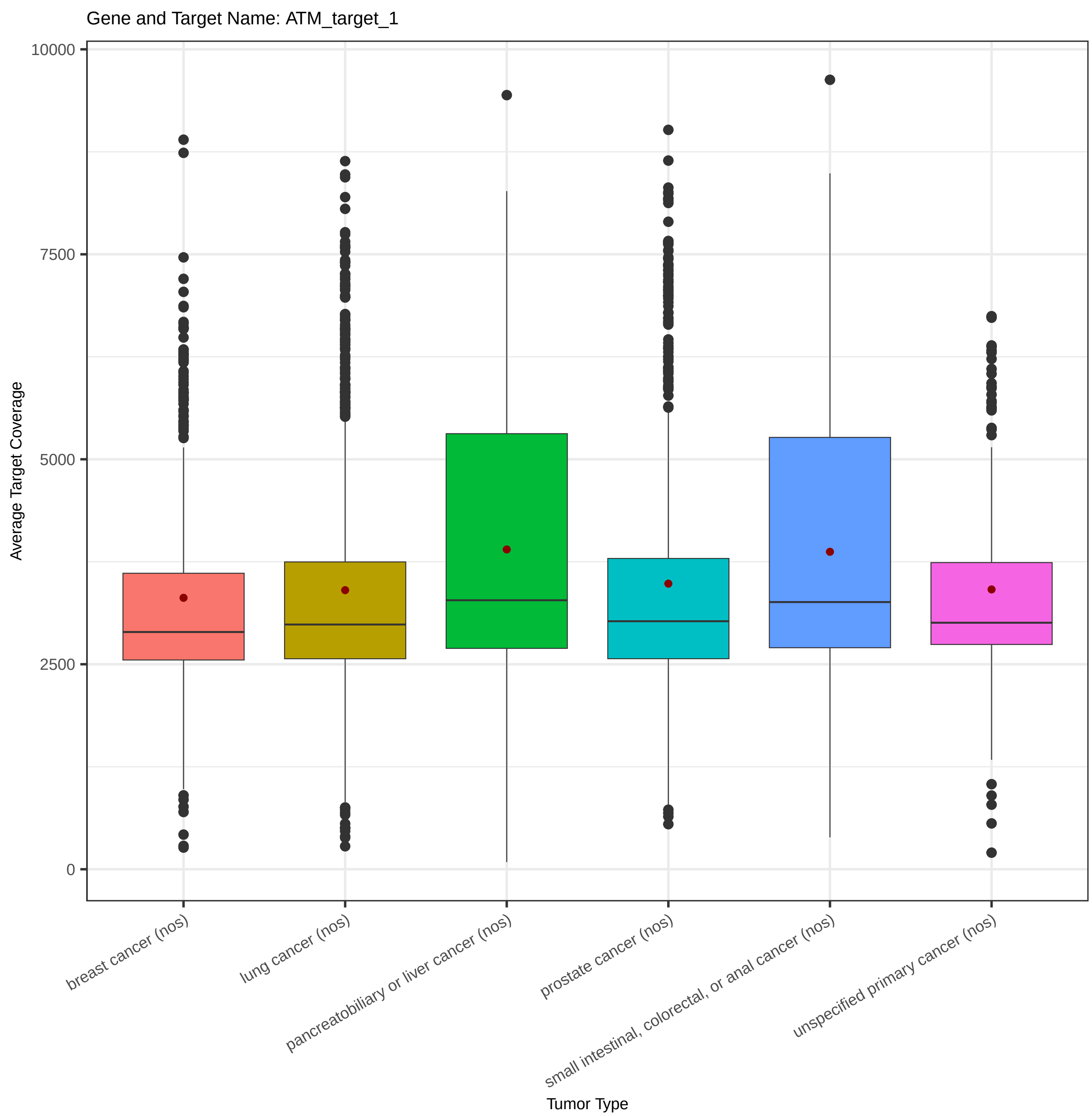

Gene and Target Name: ATM_target_1
Average Target Coverage
10000
7500
5000
2500
0
breast cancer (nos)
lung cancer (nos)
pancreatobiliary or liver cancer (nos)
prostate cancer (nos)
small intestinal, colorectal, or anal cancer (nos)
unspecified primary cancer (nos)
Tumor Type

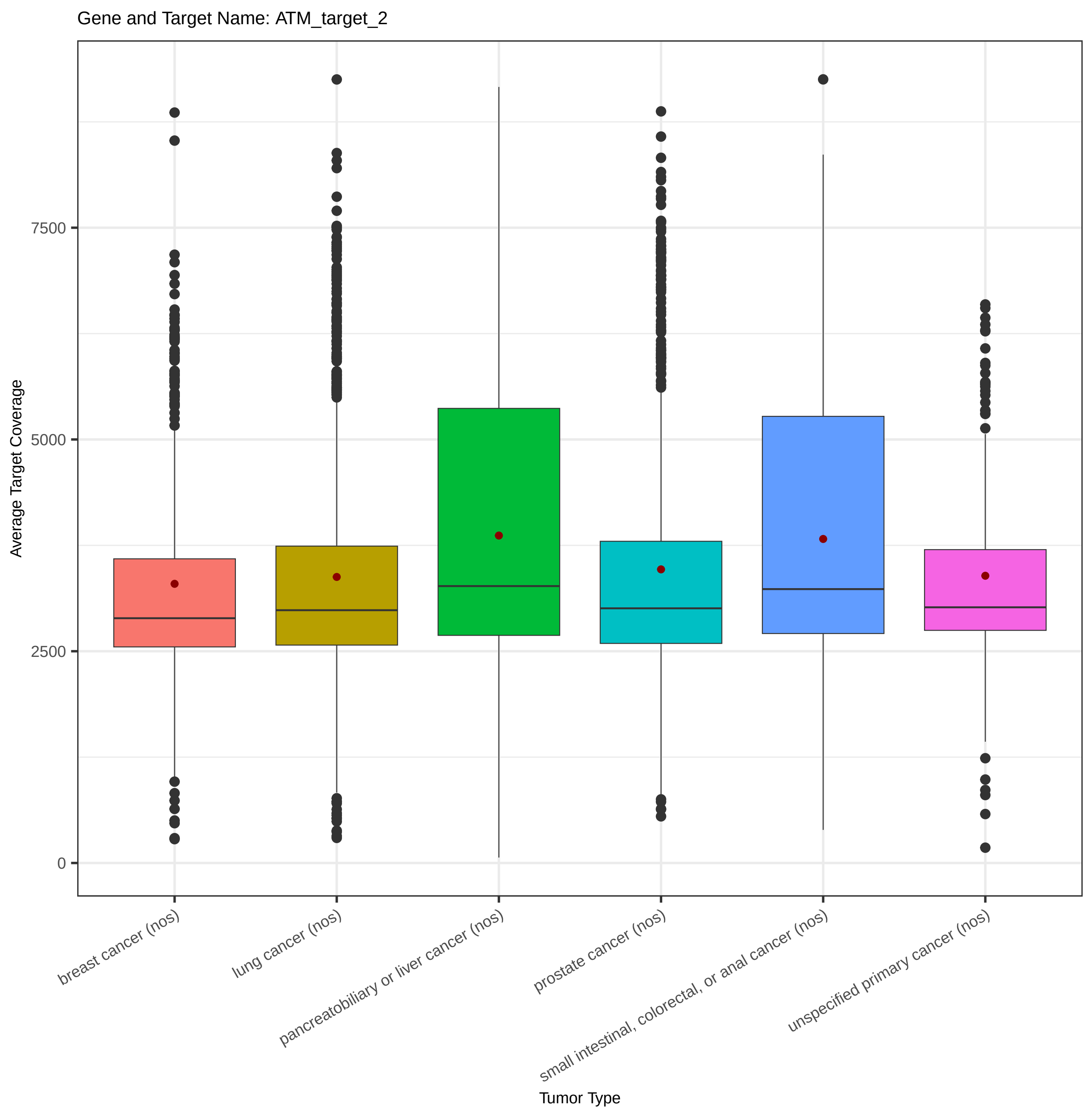

Gene and Target Name: ATM_target_2
Average Target Coverage
7500
5000
2500
0
breast cancer (nos)
lung cancer (nos)
pancreatobiliary or liver cancer (nos)
prostate cancer (nos)
small intestinal, colorectal, or anal cancer (nos)
unspecified primary cancer (nos)
Tumor Type

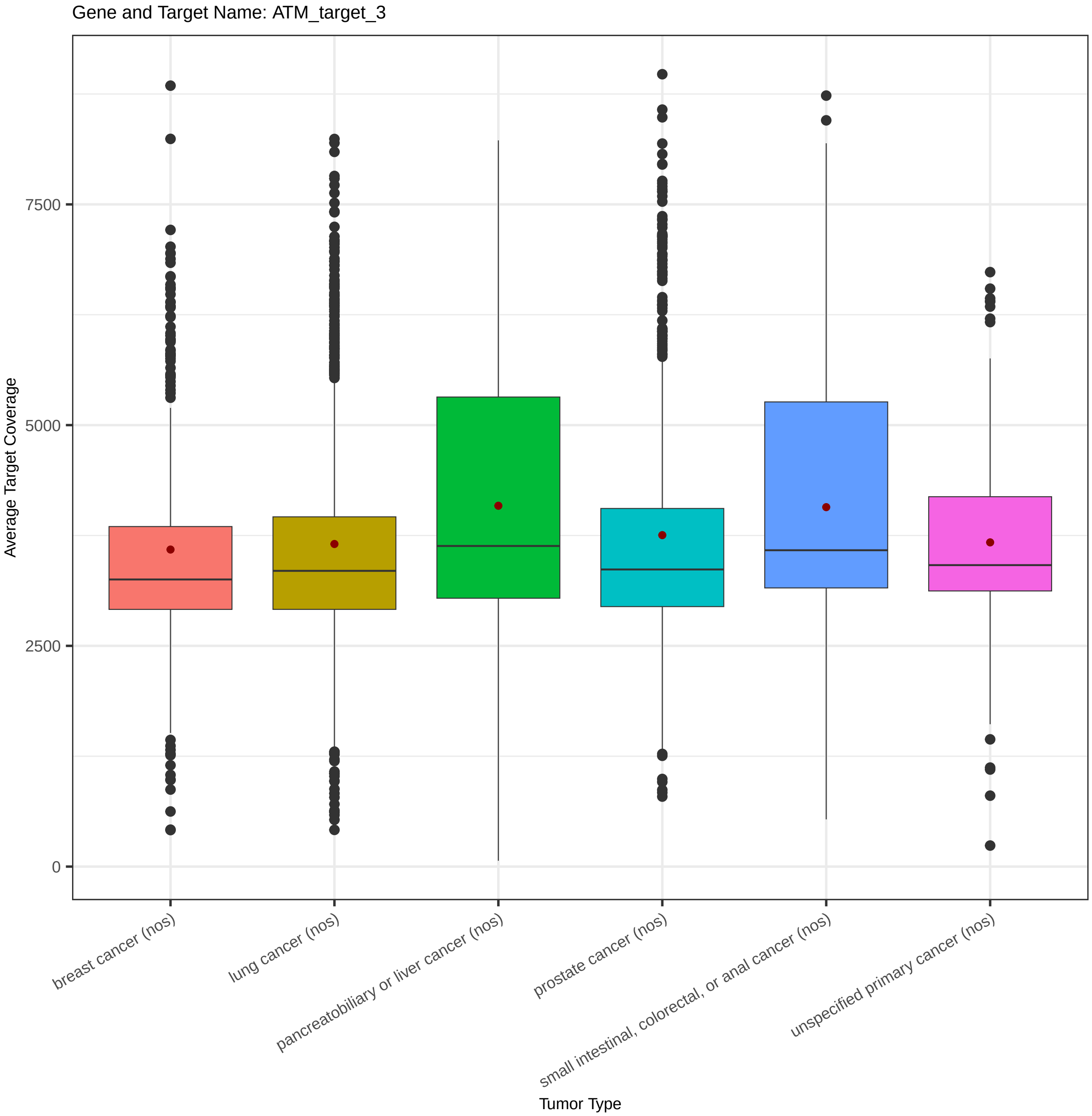
Gene and Target Name: ATM_target_3
Average Target Coverage
0
2500
5000
7500
breast cancer (nos)
lung cancer (nos)
pancreatobiliary or liver cancer (nos)
prostate cancer (nos)
small intestinal, colorectal, or anal cancer (nos)
unspecified primary cancer (nos)
Tumor Type

Gene and Target Name: ATM_target_4

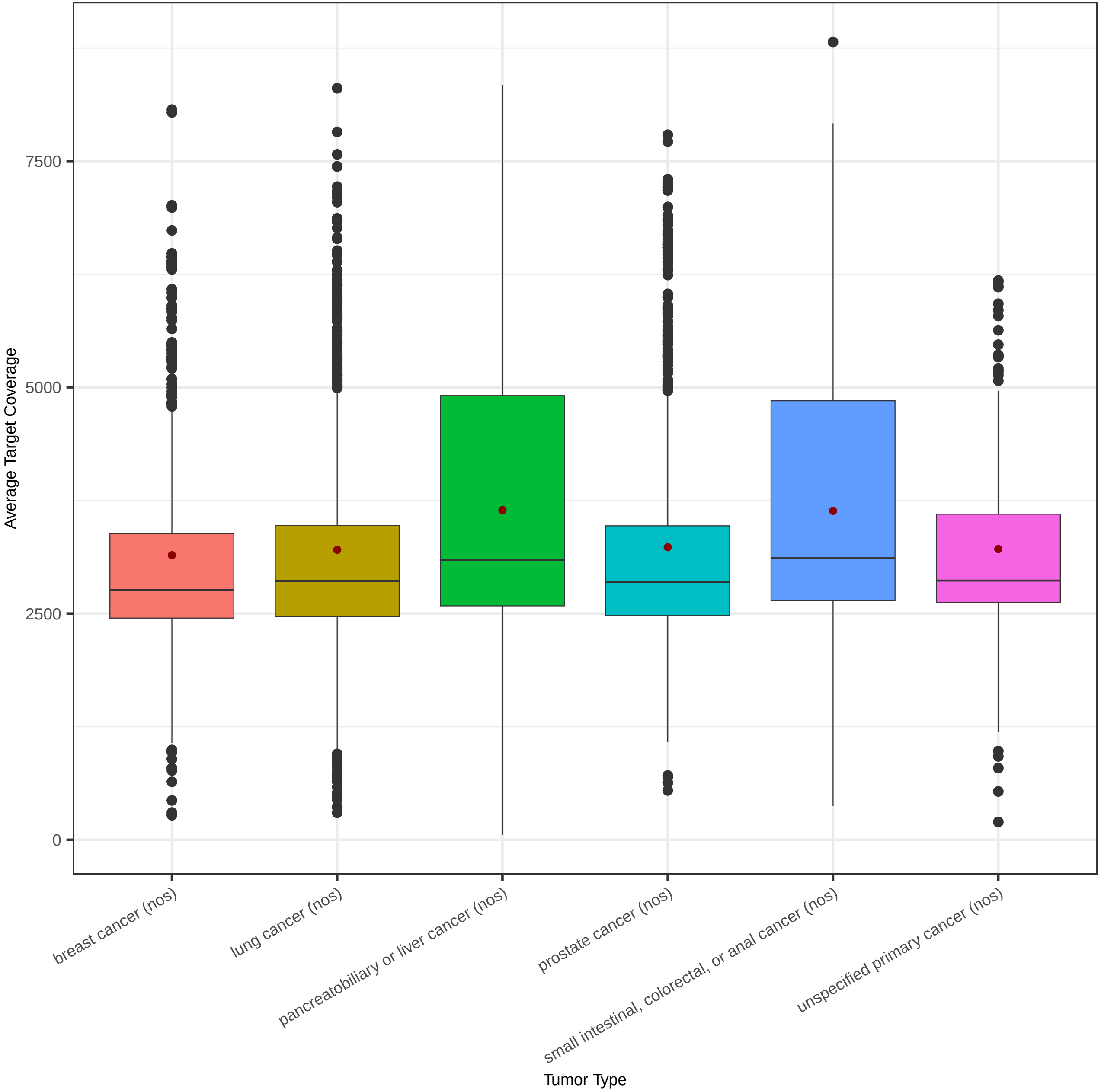

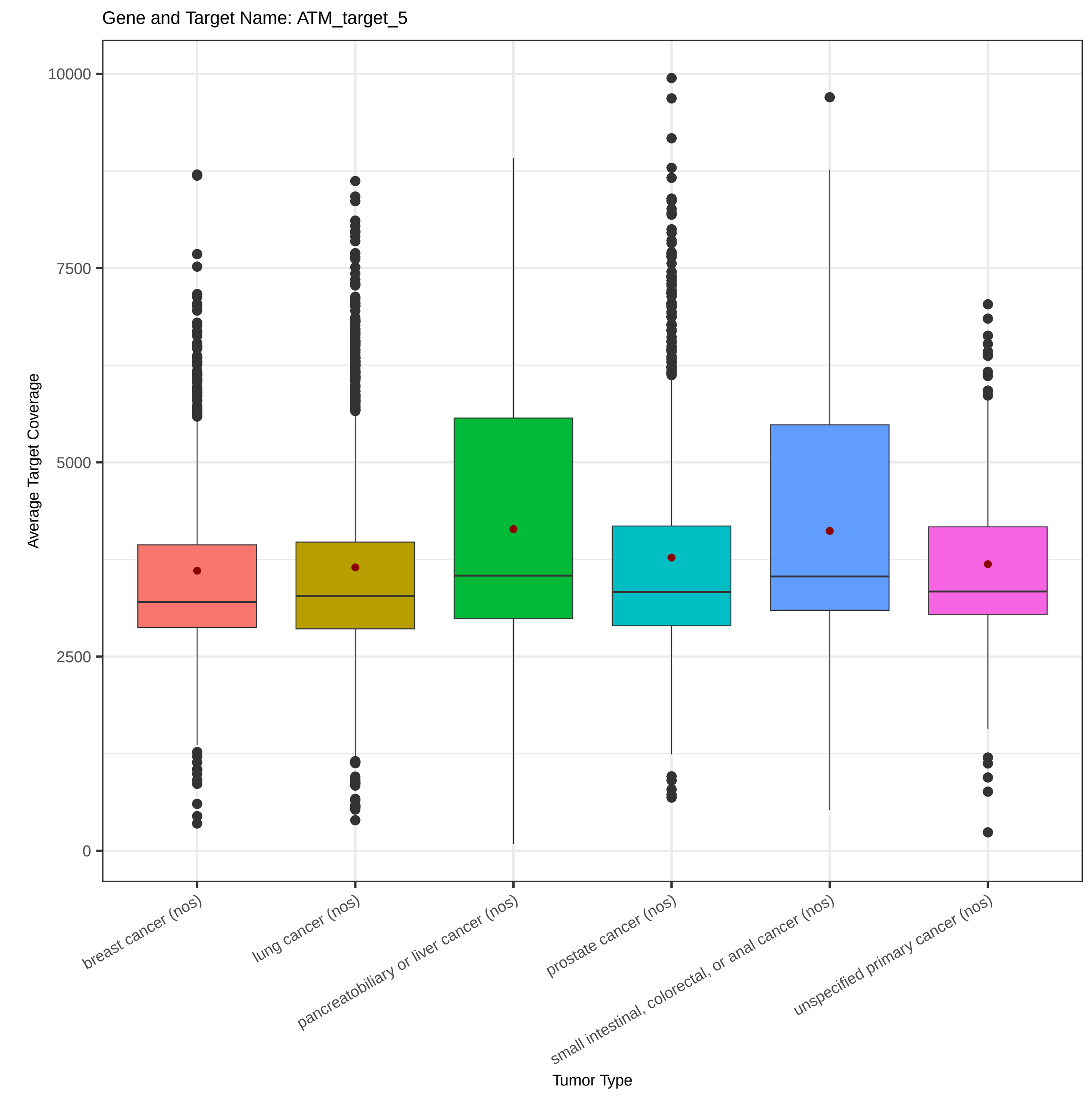

Gene and Target Name: ATM_target_5
Average Target Coverage
10000
7500
5000
2500
0
breast cancer (nos)
lung cancer (nos)
pancreatobiliary or liver cancer (nos)
prostate cancer (nos)
small intestinal, colorectal, or anal cancer (nos)
unspecified primary cancer (nos)
Tumor Type

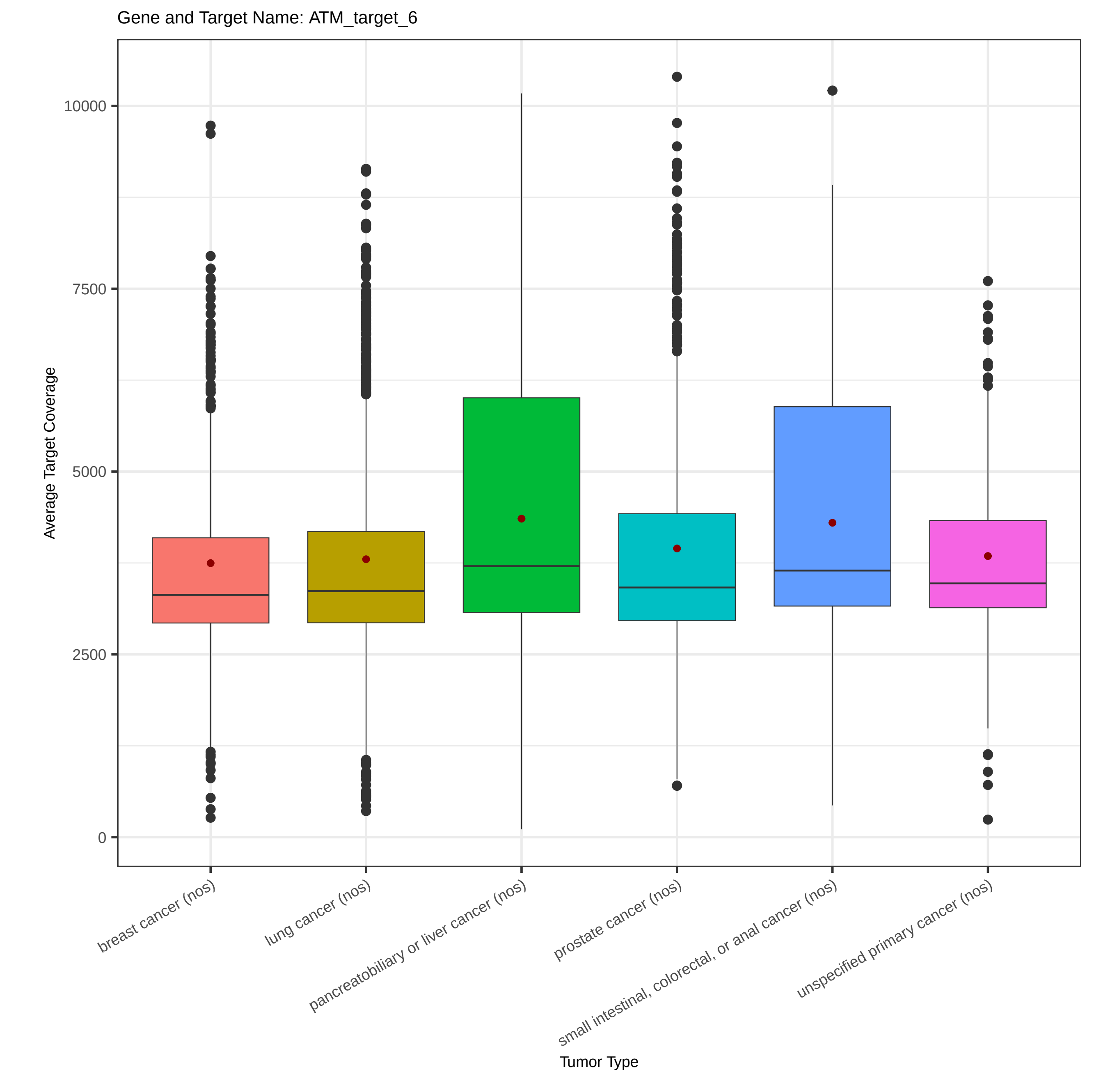
Gene and Target Name: ATM_target_6
Average Target Coverage
10000
7500
5000
2500
0
breast cancer (nos)
lung cancer (nos)
pancreatobiliary or liver cancer (nos)
prostate cancer (nos)
small intestinal, colorectal, or anal cancer (nos)
unspecified primary cancer (nos)
Tumor Type

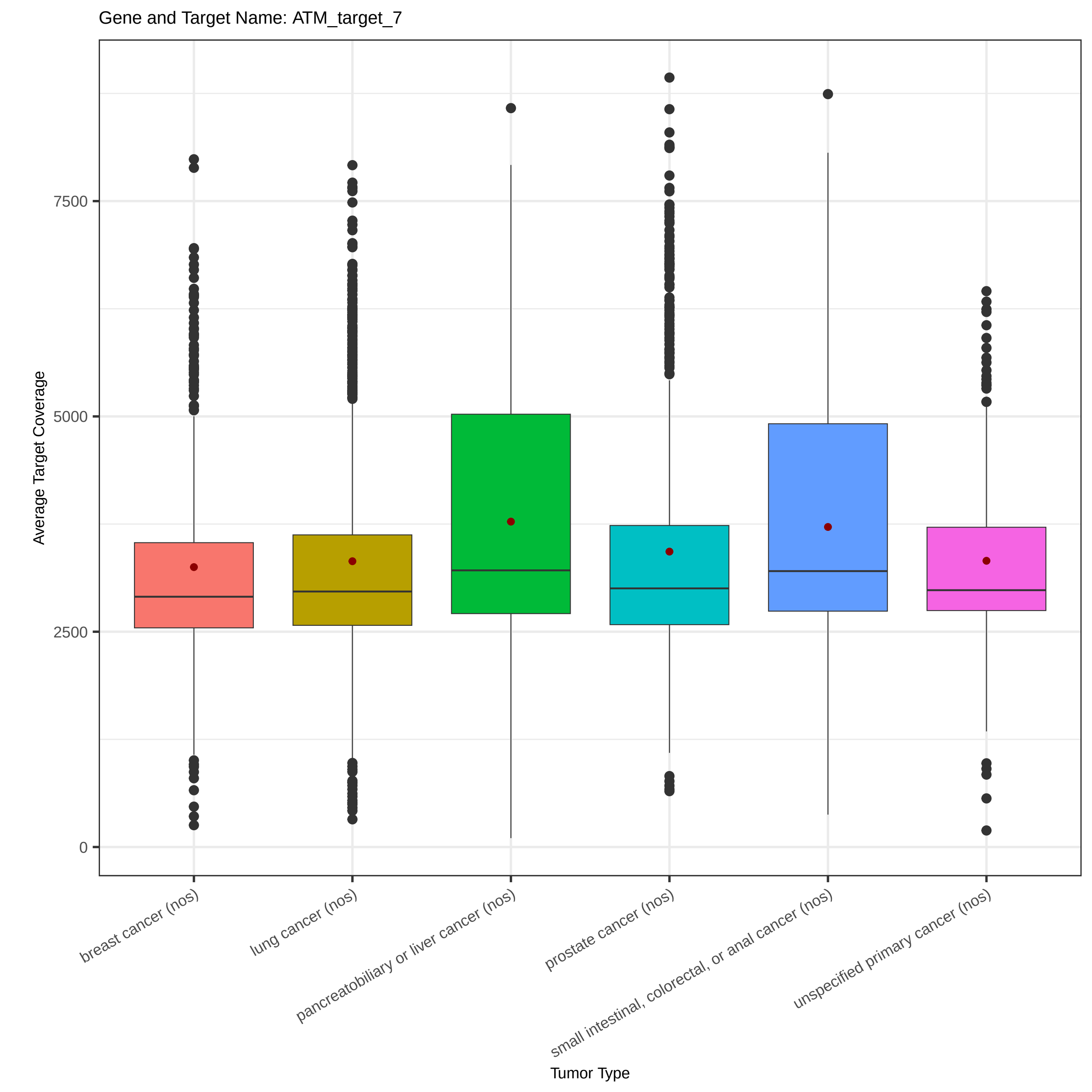

Gene and Target Name: ATM_target_7
Average Target Coverage
7500
5000
2500
0
breast cancer (nos)
lung cancer (nos)
pancreatobiliary or liver cancer (nos)
prostate cancer (nos)
small intestinal, colorectal, or anal cancer (nos)
unspecified primary cancer (nos)
Tumor Type

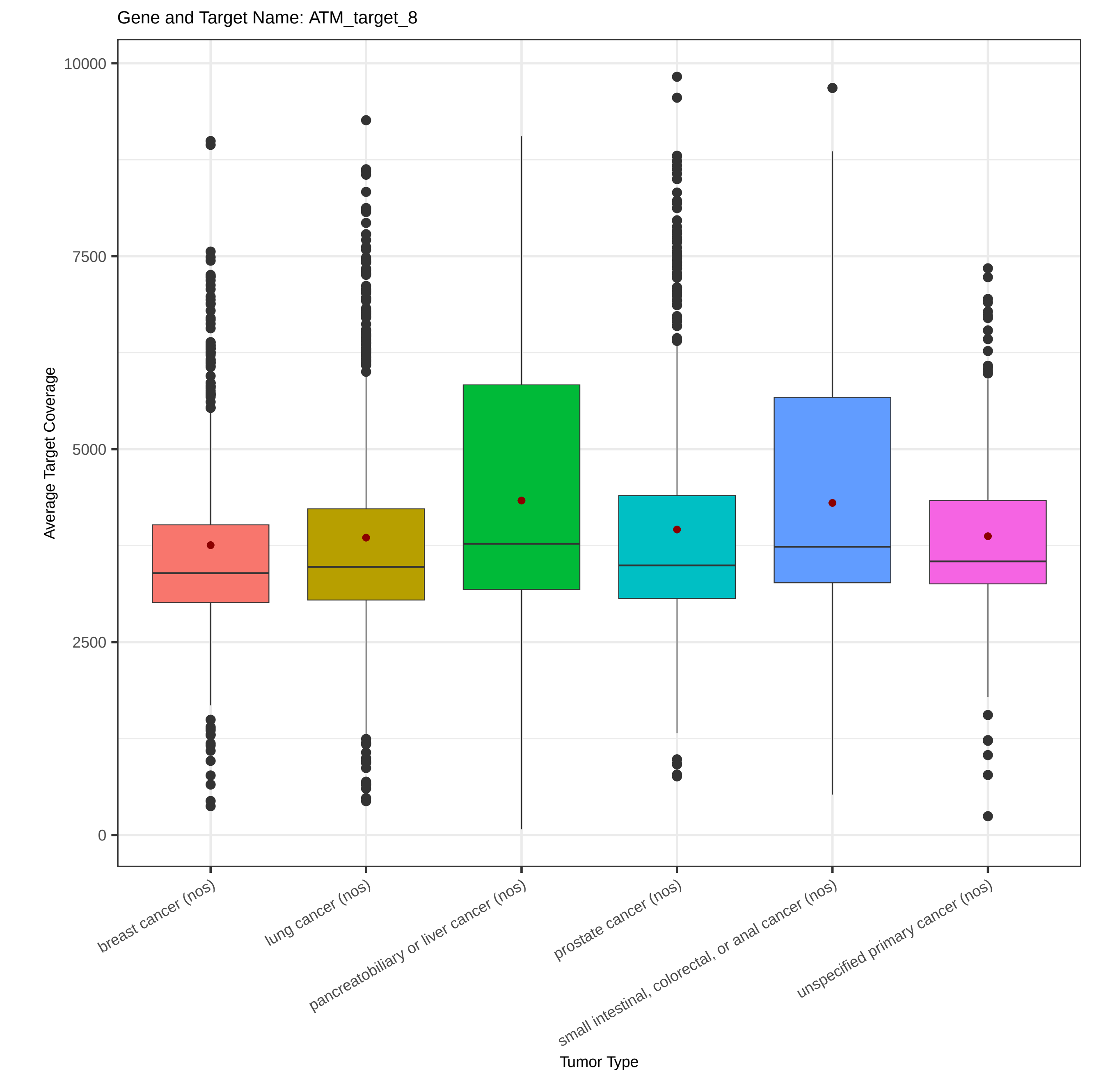
Gene and Target Name: ATM_target_8
Average Target Coverage
10000
7500
5000
2500
0
breast cancer (nos)
lung cancer (nos)
pancreatobiliary or liver cancer (nos)
prostate cancer (nos)
small intestinal, colorectal, or anal cancer (nos)
unspecified primary cancer (nos)
Tumor Type

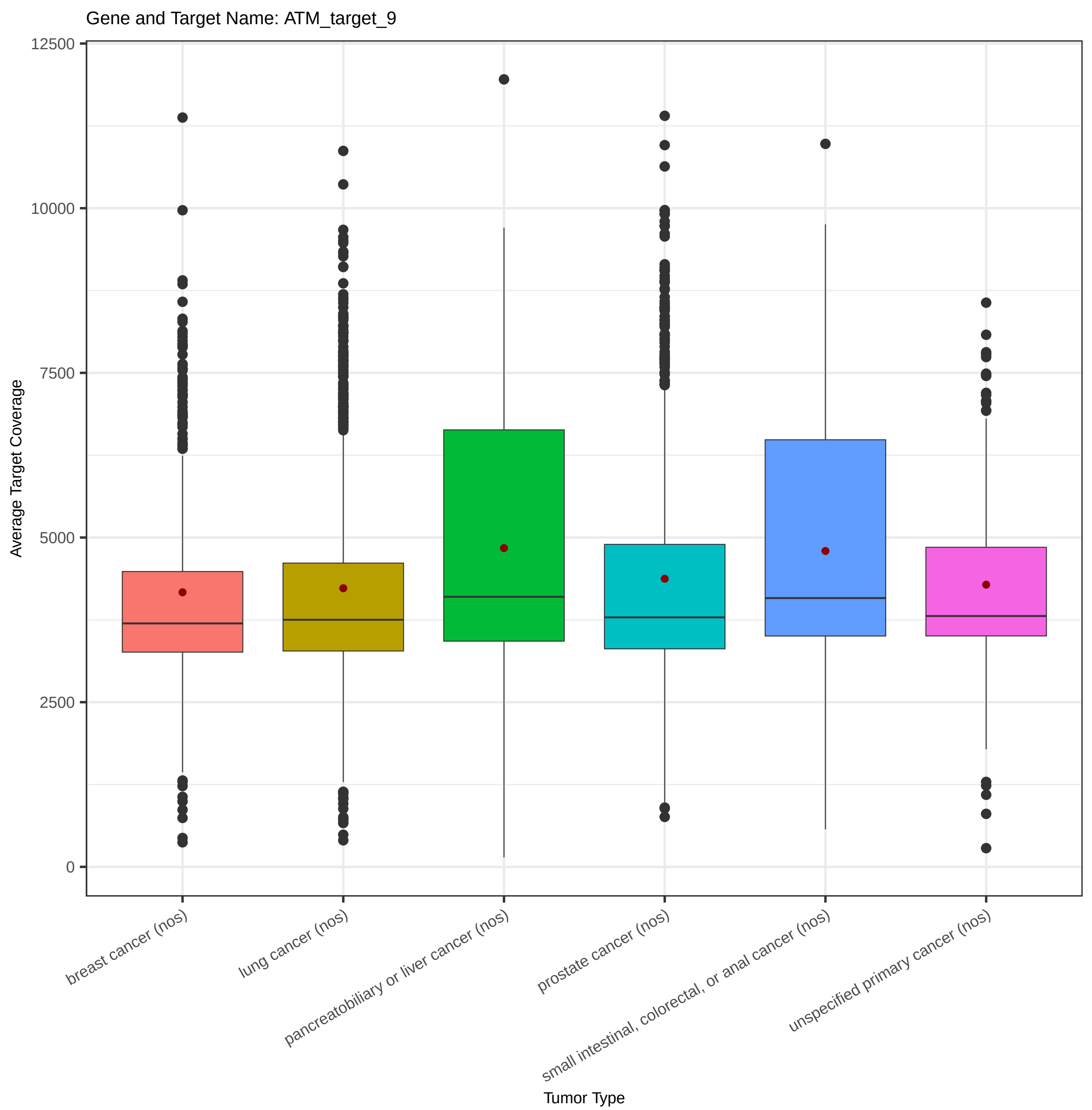
Gene and Target Name: ATM_target_9
Average Target Coverage
12500
10000
7500
5000
2500
0
breast cancer (nos)
lung cancer (nos)
pancreatobiliary or liver cancer (nos)
prostate cancer (nos)
small intestinal, colorectal, or anal cancer (nos)
unspecified primary cancer (nos)
Tumor Type

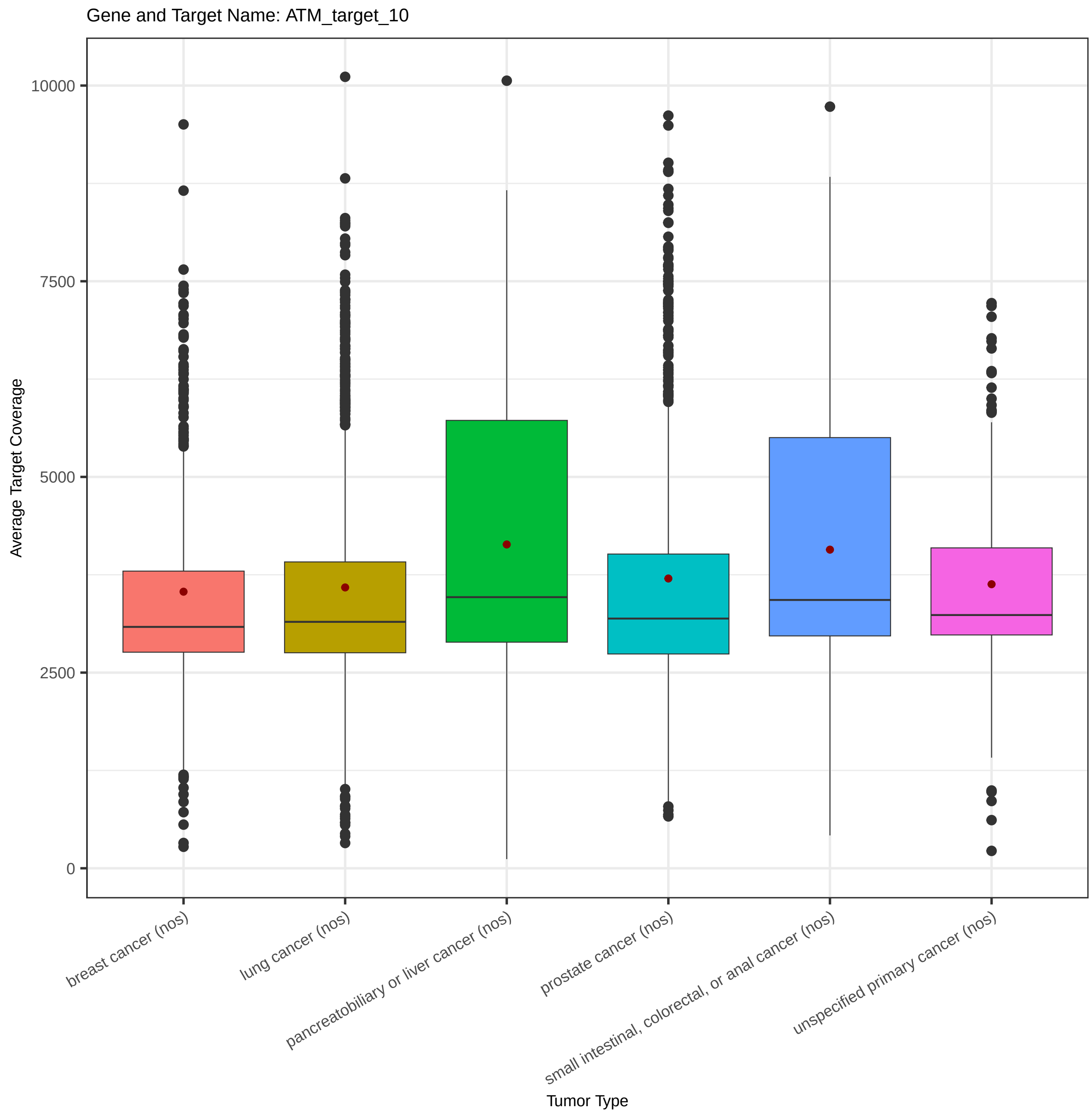

Gene and Target Name: ATM_target_10
Average Target Coverage
10000
7500
5000
2500
0
breast cancer (nos)
lung cancer (nos)
pancreatobiliary or liver cancer (nos)
prostate cancer (nos)
small intestinal, colorectal, or anal cancer (nos)
unspecified primary cancer (nos)
Tumor Type

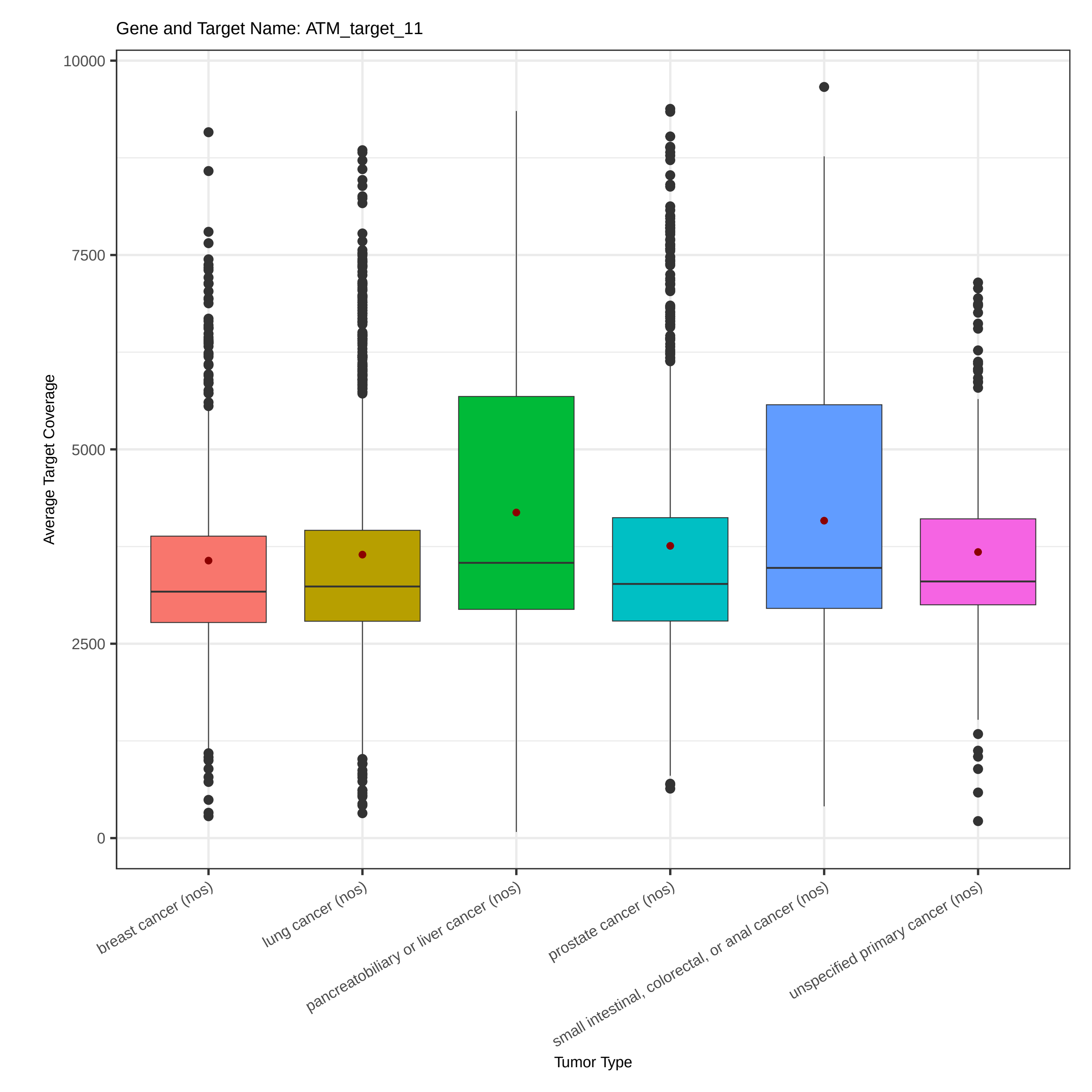

Gene and Target Name: ATM_target_11
10000
7500
5000
2500
0
Average Target Coverage
breast cancer (nos)
lung cancer (nos)
pancreatobiliary or liver cancer (nos)
prostate cancer (nos)
small intestinal, colorectal, or anal cancer (nos)
unspecified primary cancer (nos)
Tumor Type

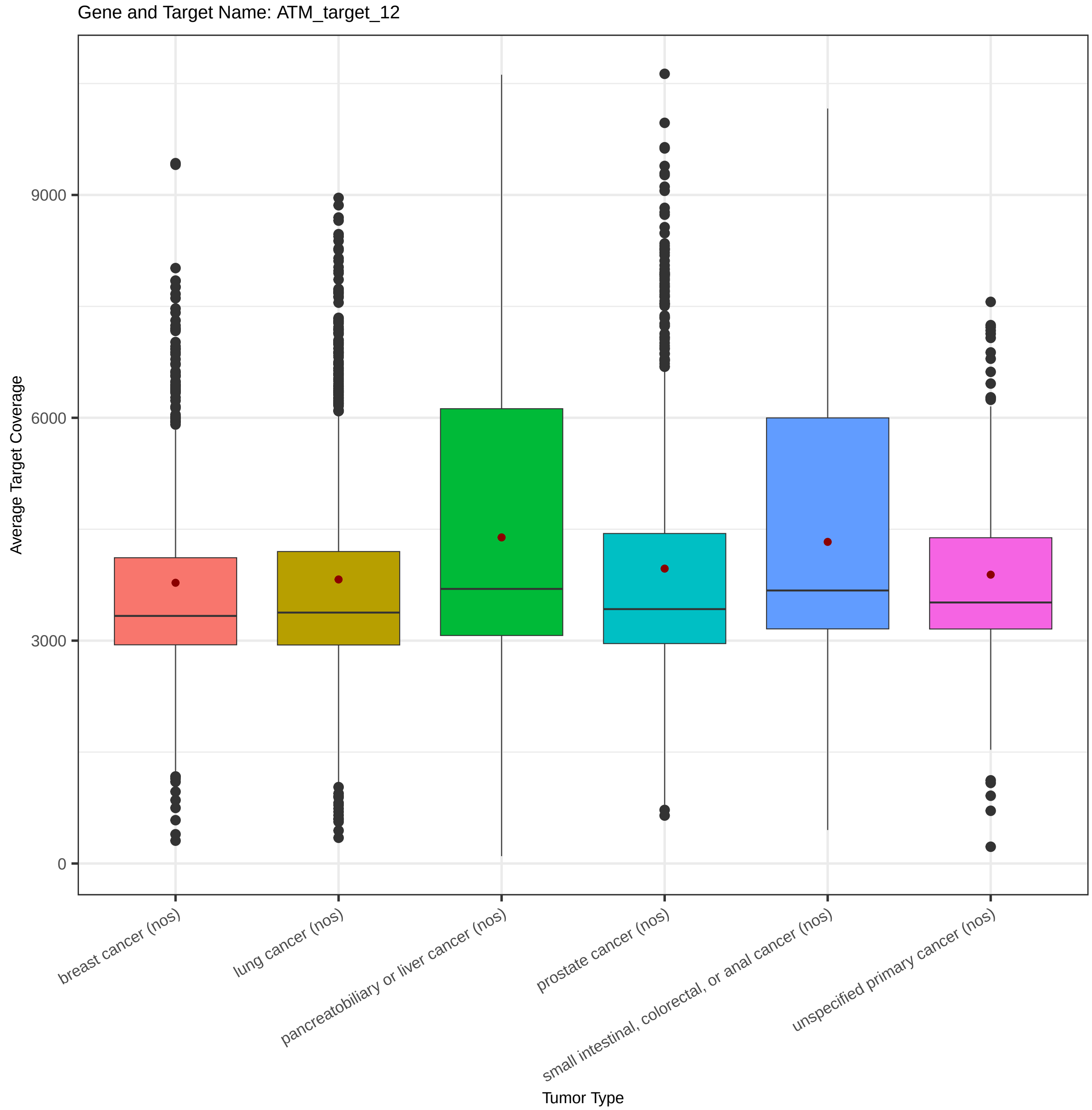
Gene and Target Name: ATM_target_12
Average Target Coverage
0
3000
6000
9000
breast cancer (nos)
lung cancer (nos)
pancreatobiliary or liver cancer (nos)
prostate cancer (nos)
small intestinal, colorectal, or anal cancer (nos)
unspecified primary cancer (nos)
Tumor Type

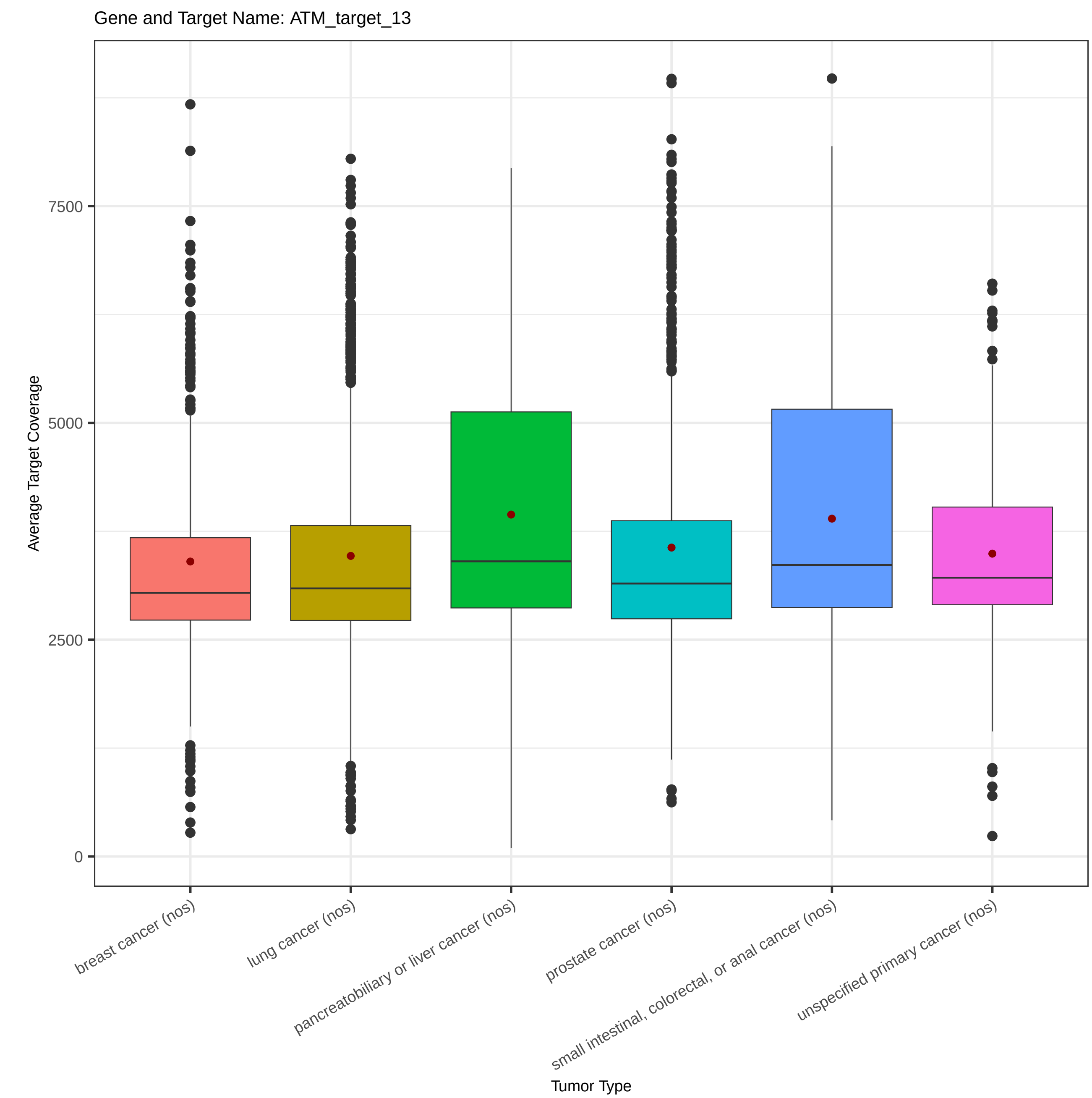

Gene and Target Name: ATM_target_13
Average Target Coverage
7500
5000
2500
0
breast cancer (nos)
lung cancer (nos)
pancreatobiliary or liver cancer (nos)
prostate cancer (nos)
small intestinal, colorectal, or anal cancer (nos)
unspecified primary cancer (nos)
Tumor Type

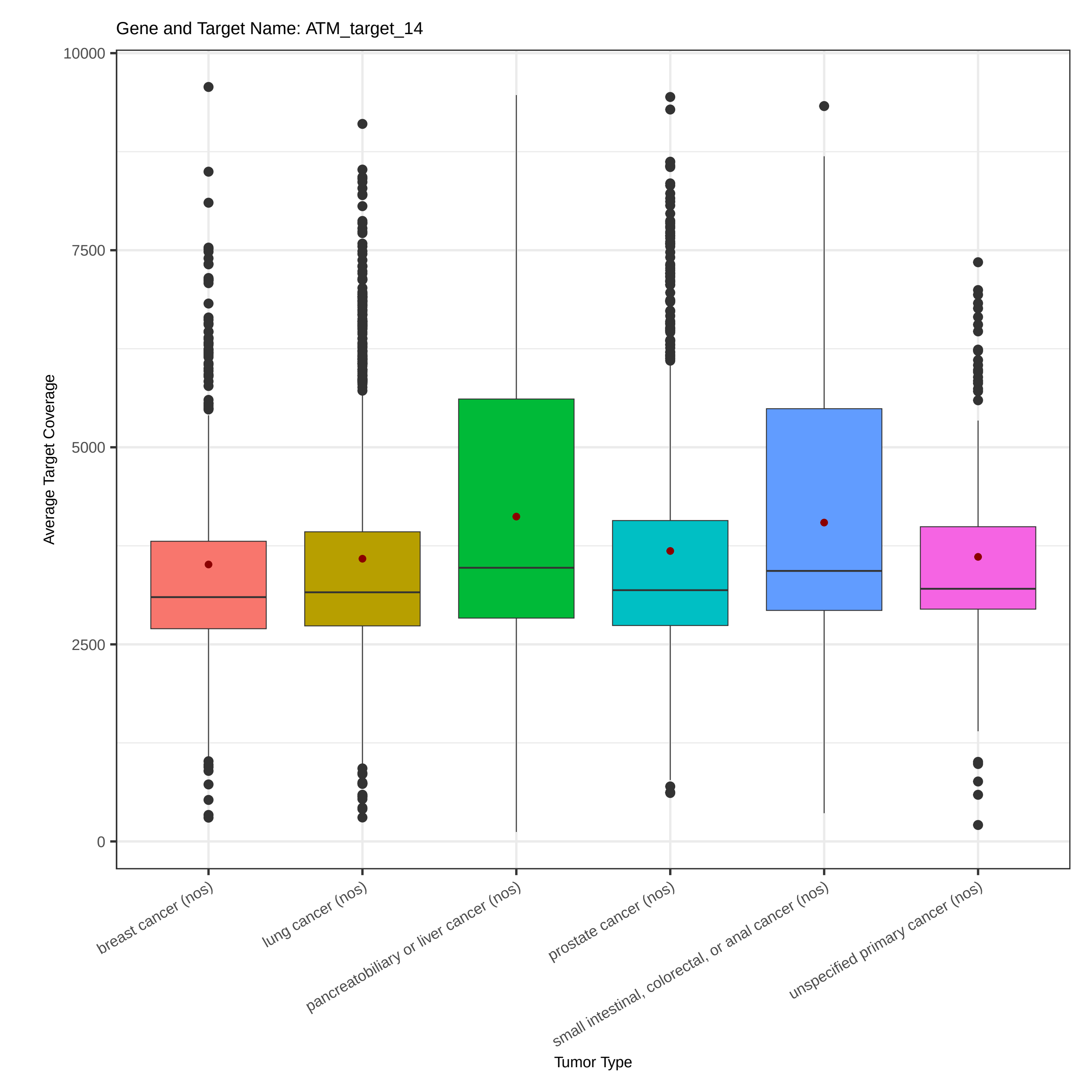
Gene and Target Name: ATM_target_14
Average Target Coverage
10000
7500
5000
2500
0
breast cancer (nos)
lung cancer (nos)
pancreatobiliary or liver cancer (nos)
prostate cancer (nos)
small intestinal, colorectal, or anal cancer (nos)
unspecified primary cancer (nos)
Tumor Type

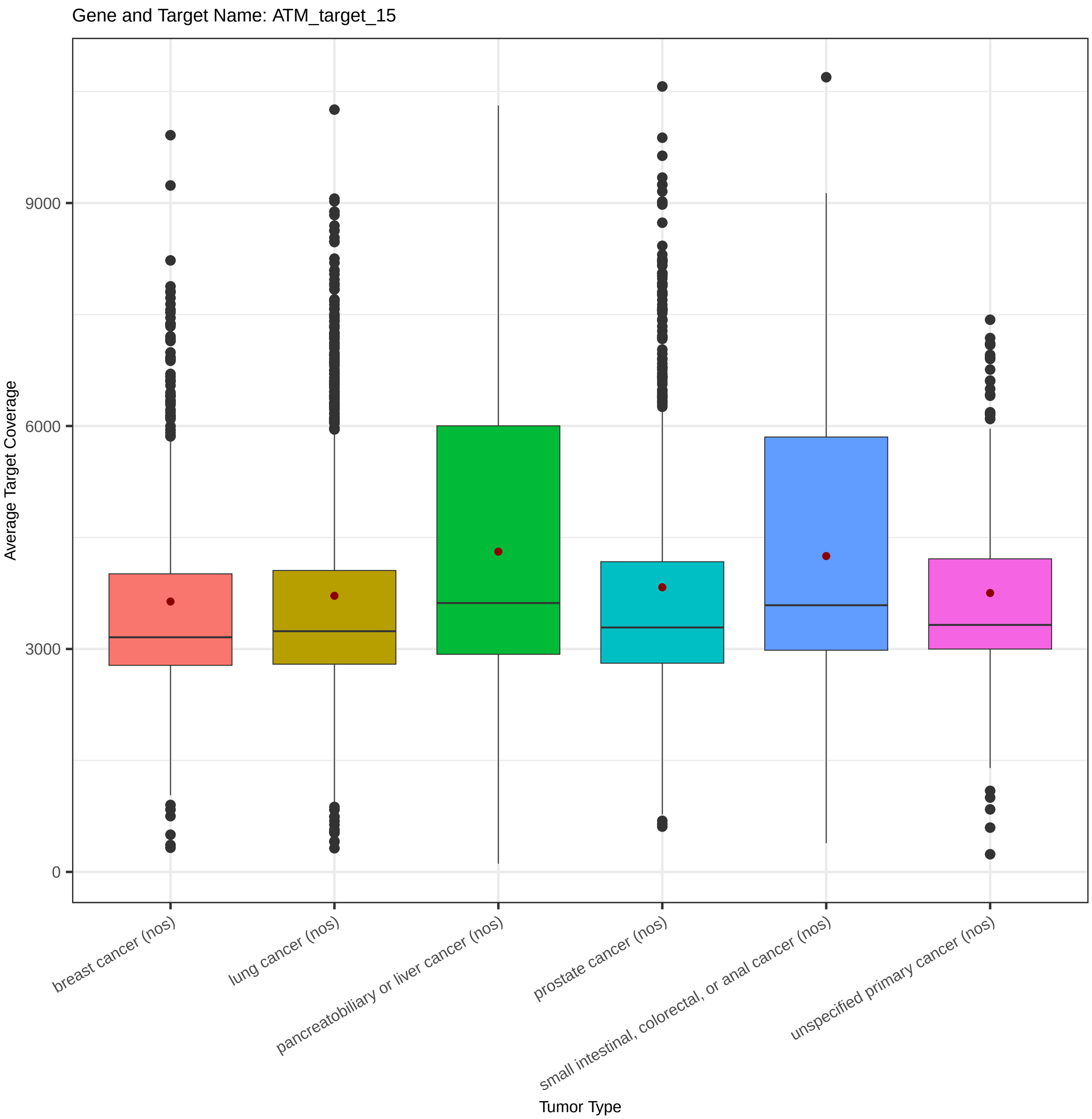
Gene and Target Name: ATM_target_15
Average Target Coverage
0
3000
6000
9000
breast cancer (nos)
lung cancer (nos)
pancreatobiliary or liver cancer (nos)
prostate cancer (nos)
small intestinal, colorectal, or anal cancer (nos)
unspecified primary cancer (nos)
Tumor Type

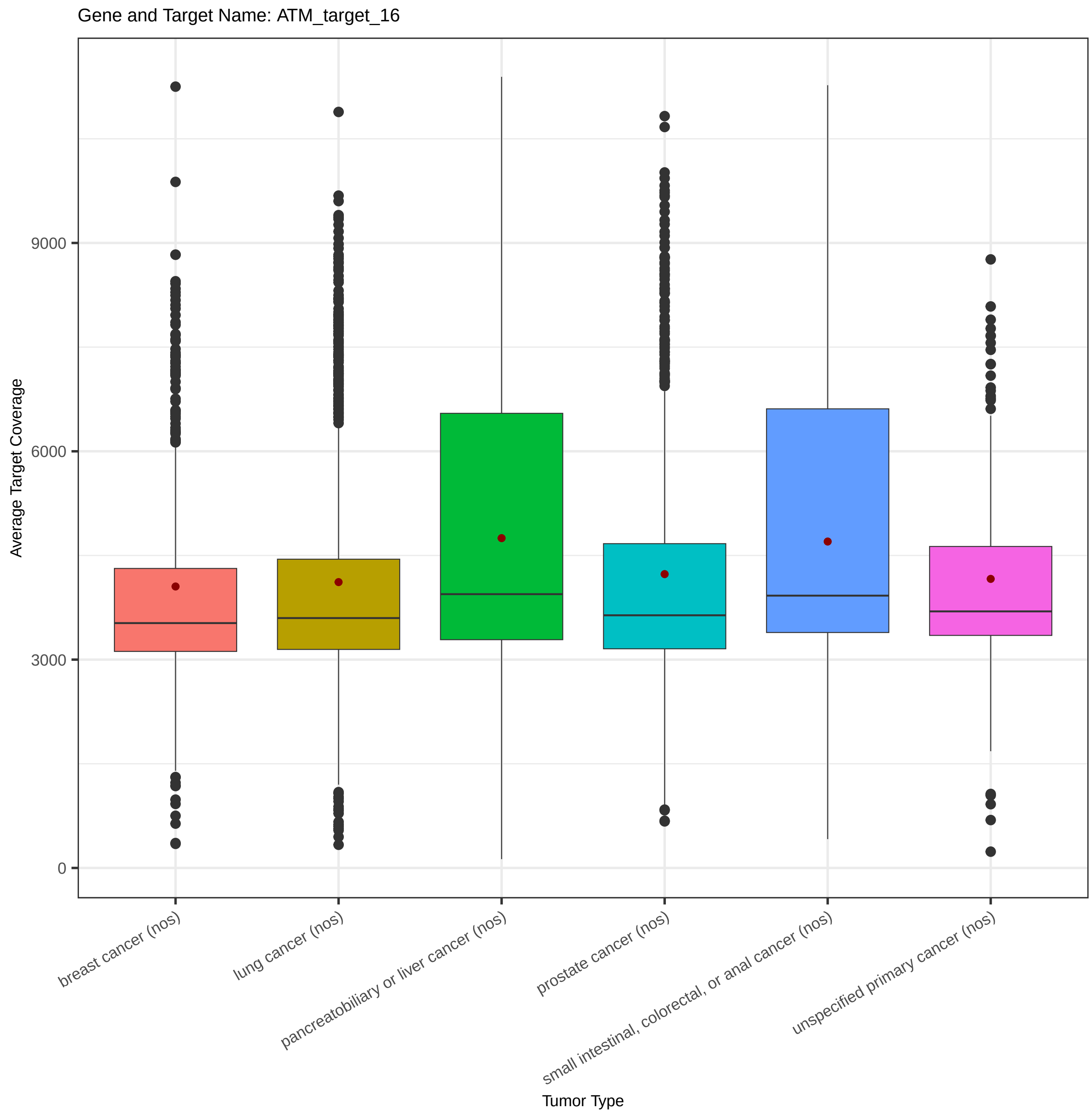
Gene and Target Name: ATM_target_16
Average Target Coverage
9000
6000
3000
0
breast cancer (nos)
lung cancer (nos)
pancreatobiliary or liver cancer (nos)
prostate cancer (nos)
small intestinal, colorectal, or anal cancer (nos)
unspecified primary cancer (nos)
Tumor Type

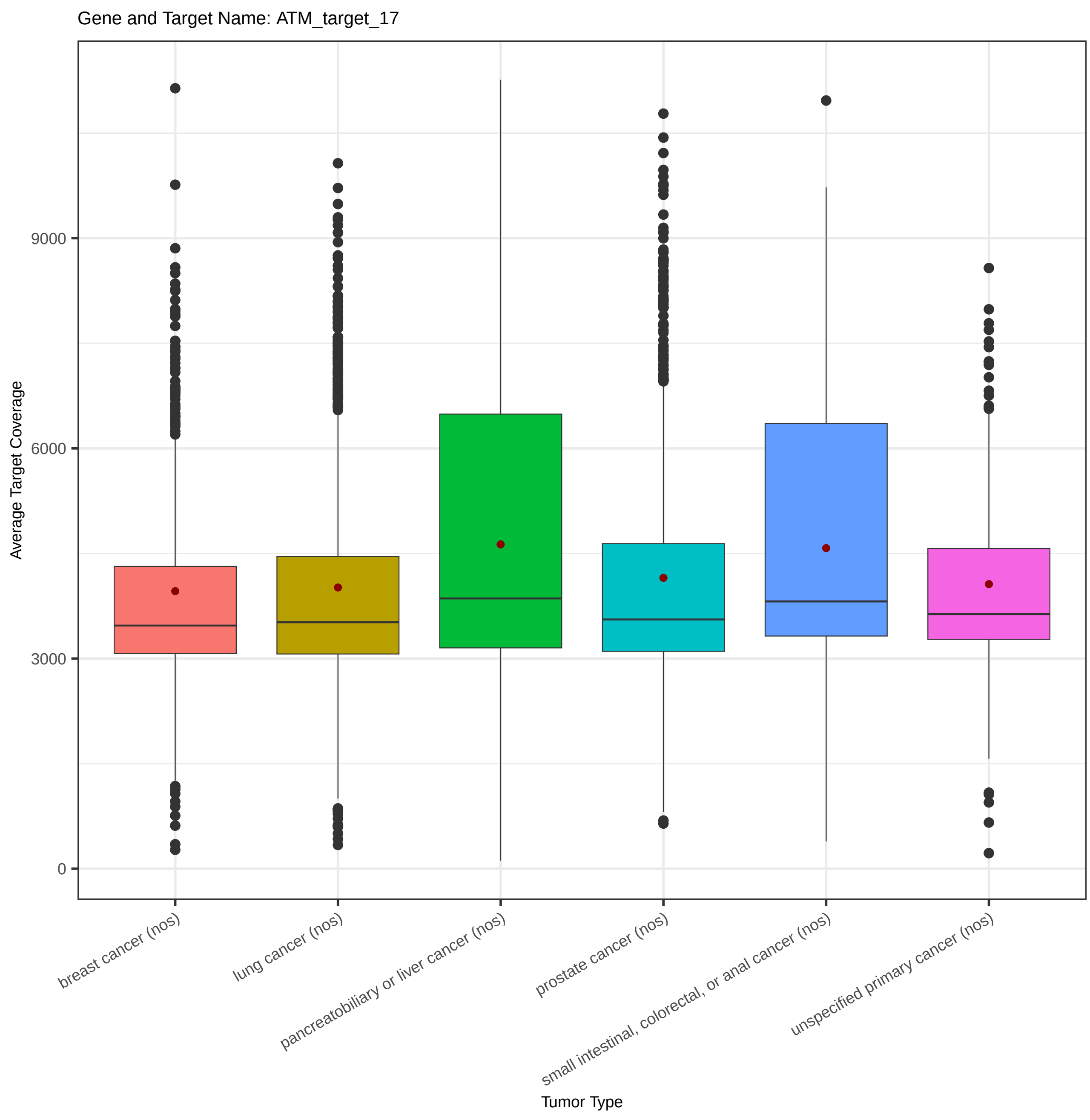
Gene and Target Name: ATM_target_17
Average Target Coverage
9000
6000
3000
0
breast cancer (nos)
lung cancer (nos)
pancreatobiliary or liver cancer (nos)
prostate cancer (nos)
small intestinal, colorectal, or anal cancer (nos)
unspecified primary cancer (nos)
Tumor Type

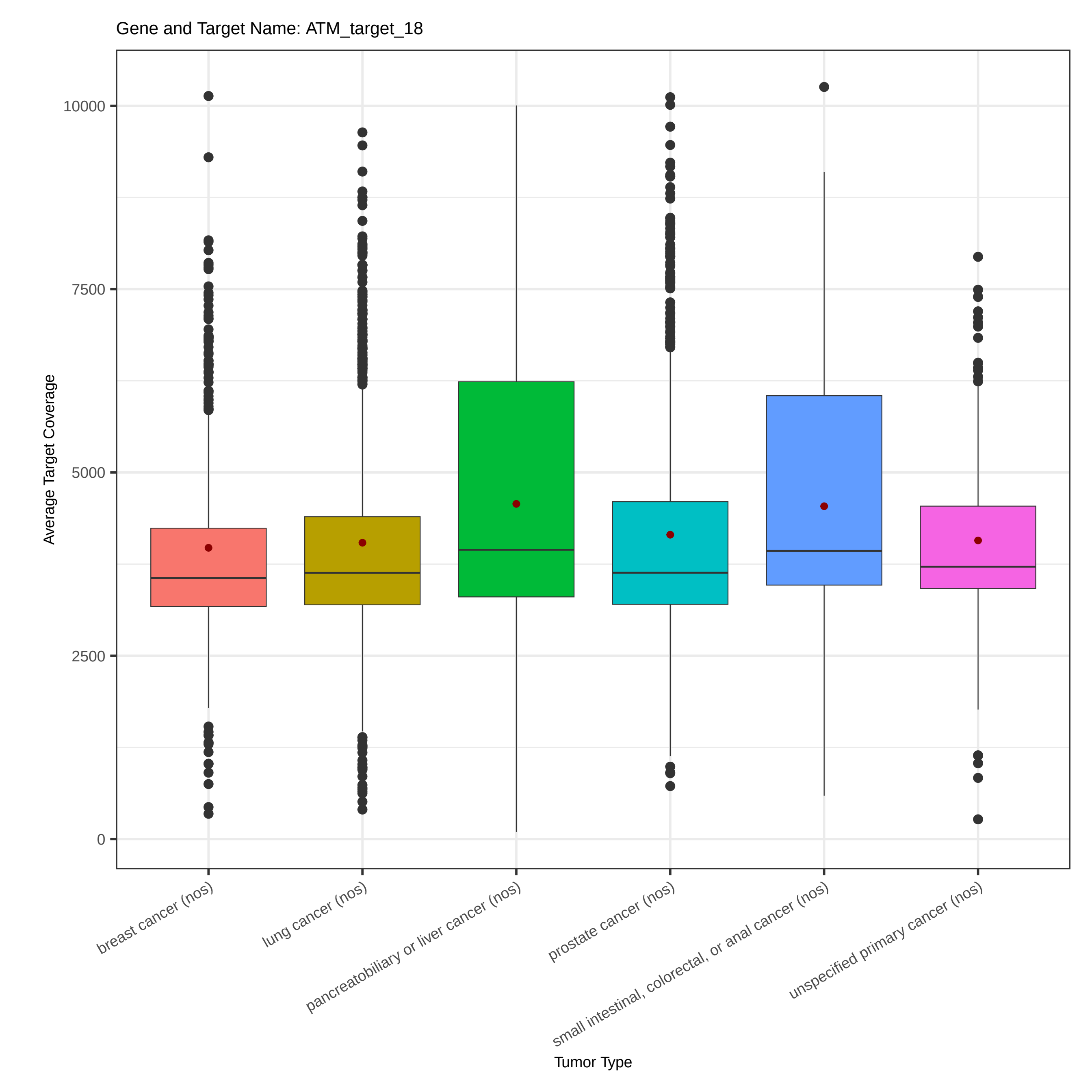

Gene and Target Name: ATM_target_18
Average Target Coverage
10000
7500
5000
2500
0
breast cancer (nos)
lung cancer (nos)
pancreatobiliary or liver cancer (nos)
prostate cancer (nos)
small intestinal, colorectal, or anal cancer (nos)
unspecified primary cancer (nos)
Tumor Type

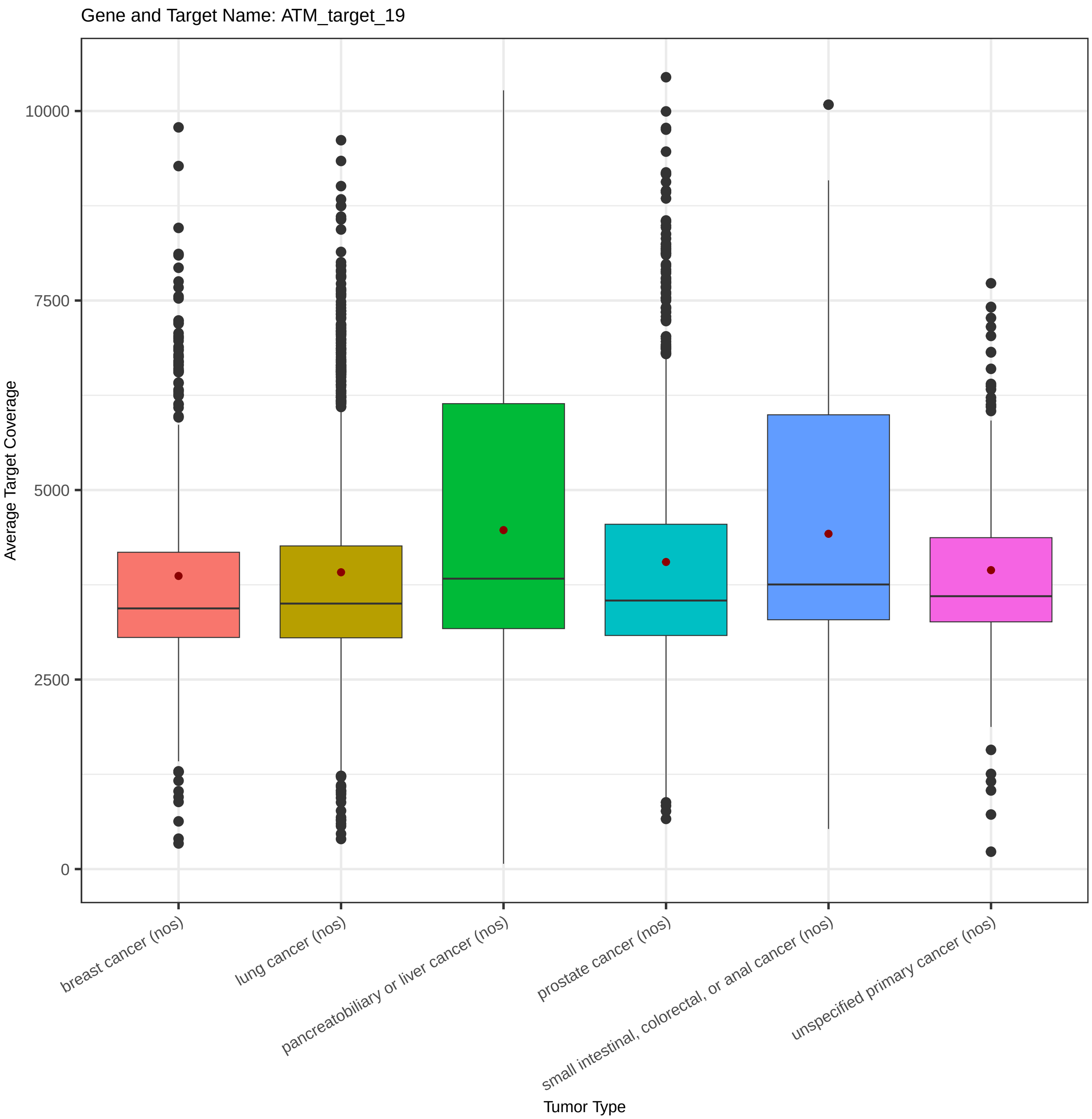
Gene and Target Name: ATM_target_19
Average Target Coverage
10000
7500
5000
2500
0
breast cancer (nos)
lung cancer (nos)
pancreatobiliary or liver cancer (nos)
prostate cancer (nos)
small intestinal, colorectal, or anal cancer (nos)
unspecified primary cancer (nos)
Tumor Type

Gene and Target Name: ATM_target_20

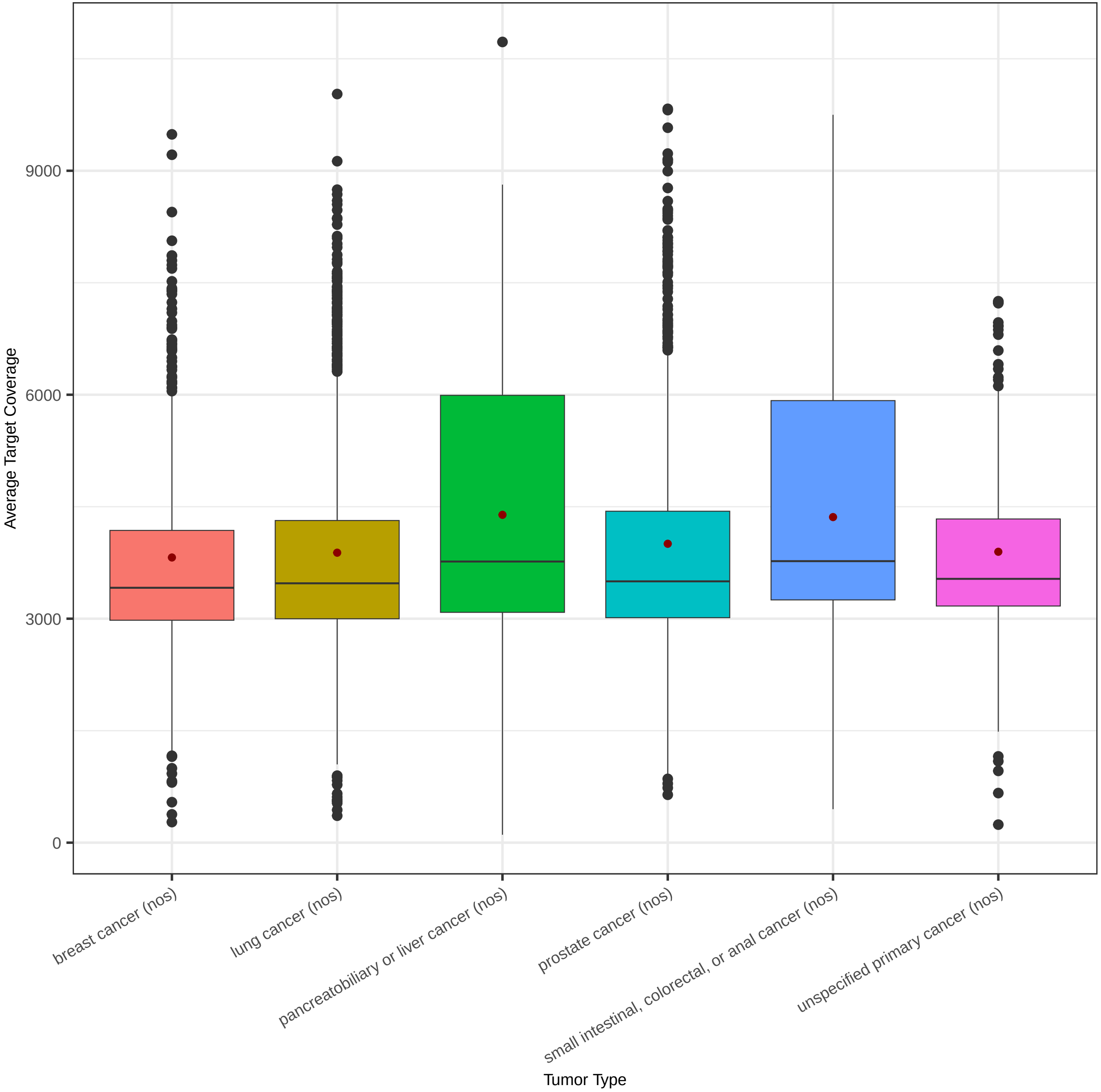

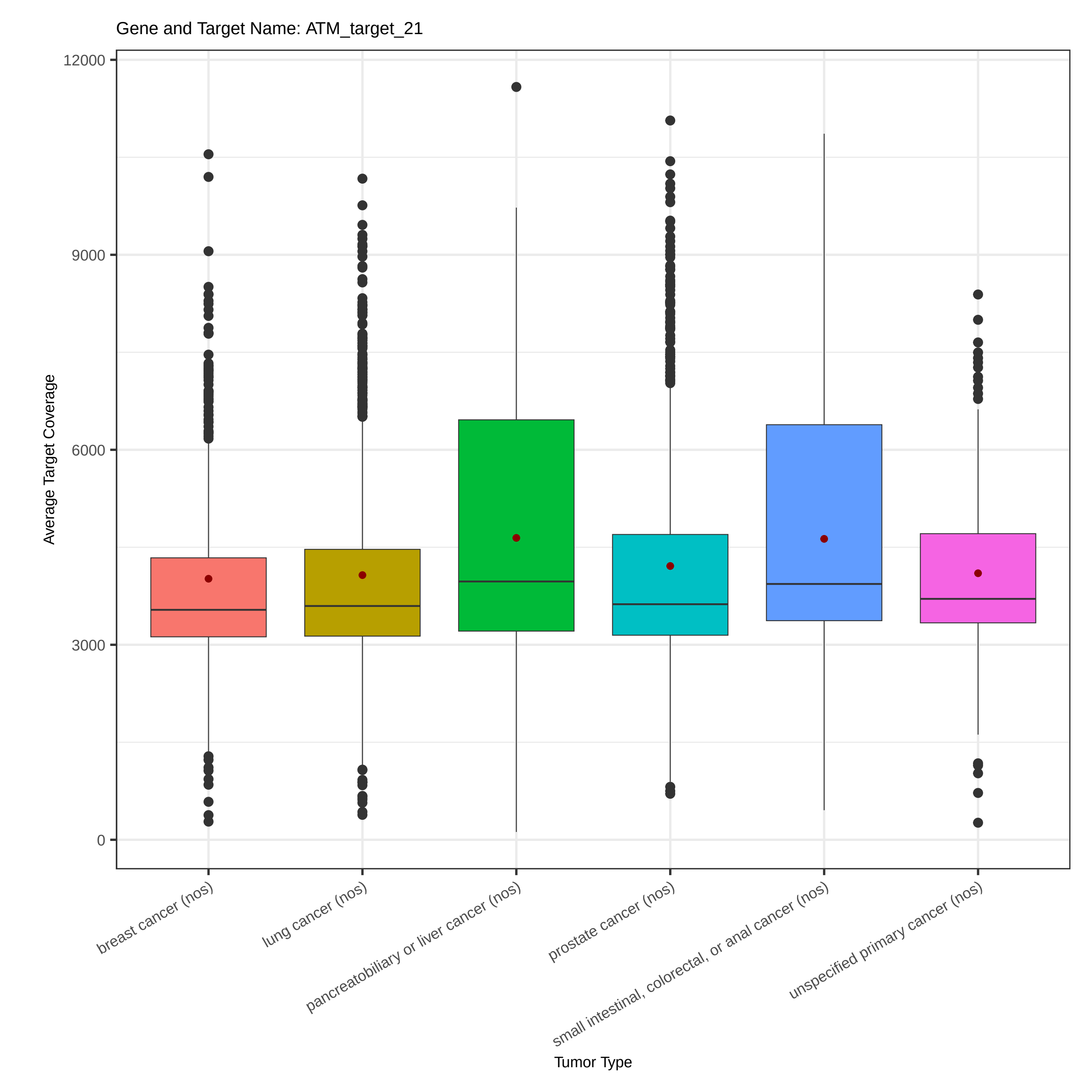
Gene and Target Name: ATM_target_21
12000
9000
6000
3000
0
Average Target Coverage
breast cancer (nos)
lung cancer (nos)
pancreatobiliary or liver cancer (nos)
prostate cancer (nos)
small intestinal, colorectal, or anal cancer (nos)
unspecified primary cancer (nos)
Tumor Type

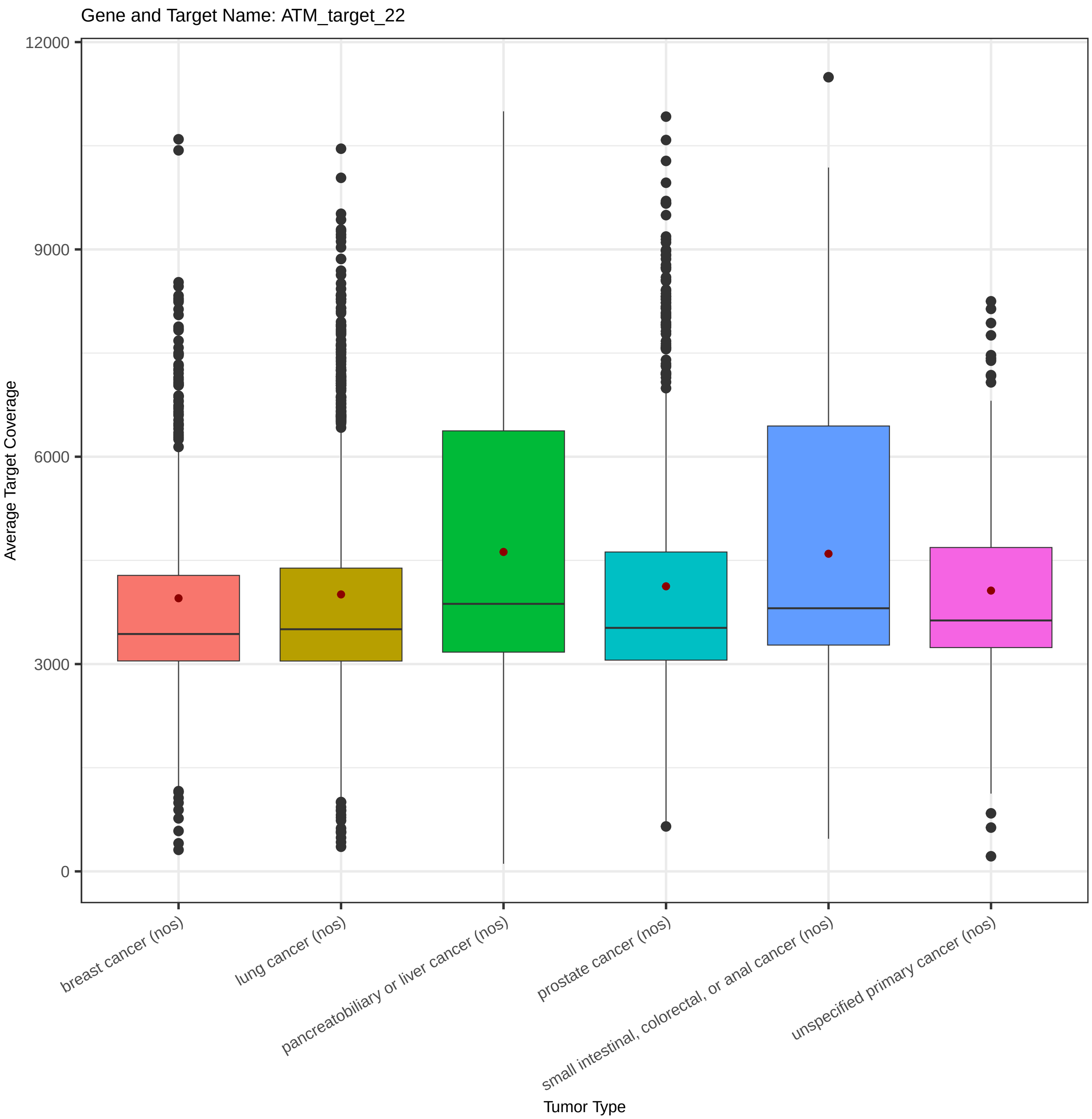

Gene and Target Name: ATM_target_22
Average Target Coverage
12000
9000
6000
3000
0
breast cancer (nos)
lung cancer (nos)
pancreatobiliary or liver cancer (nos)
prostate cancer (nos)
small intestinal, colorectal, or anal cancer (nos)
unspecified primary cancer (nos)
Tumor Type

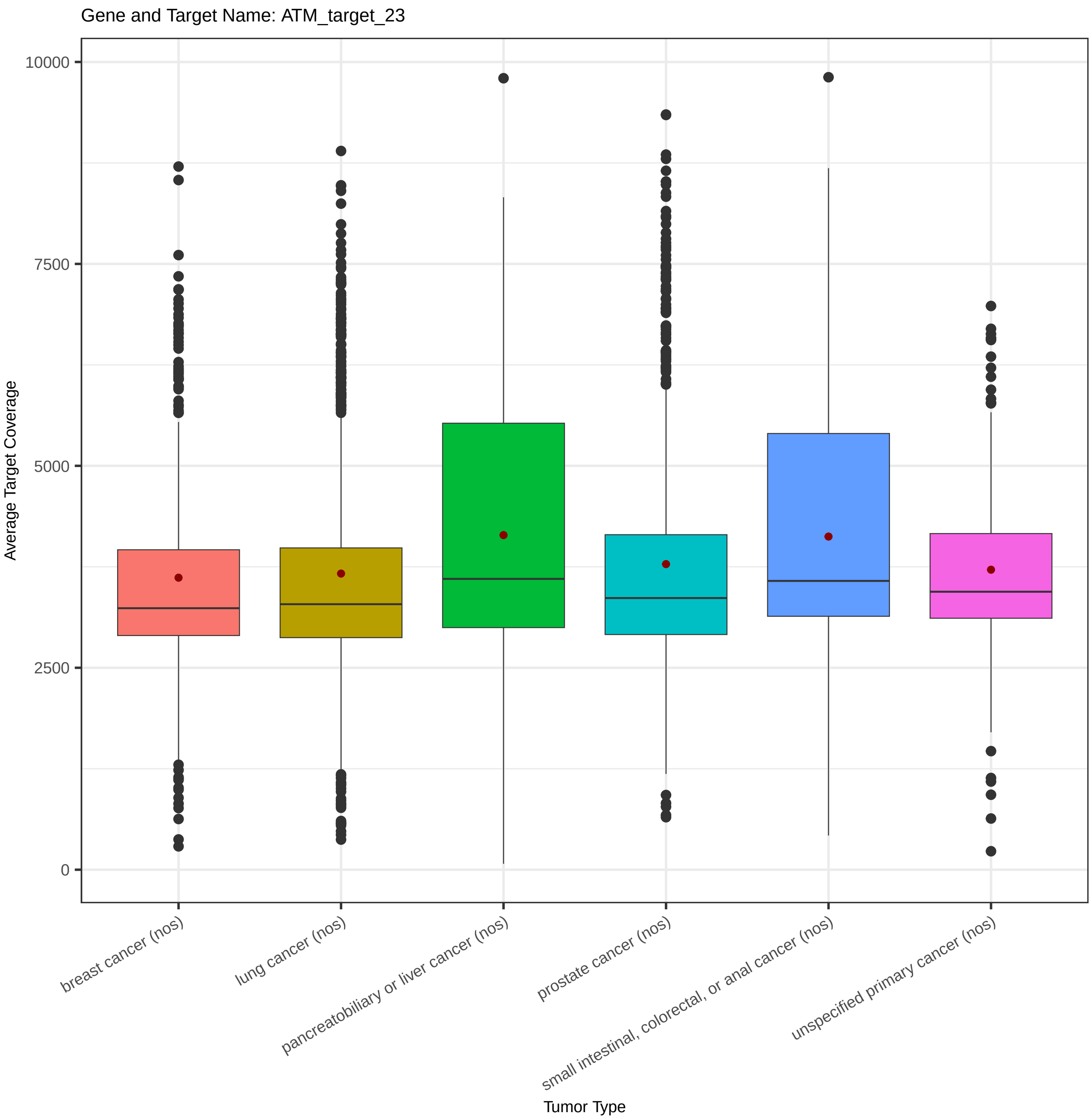
Gene and Target Name: ATM_target_23
Average Target Coverage
10000
7500
5000
2500
0
breast cancer (nos)
lung cancer (nos)
pancreatobiliary or liver cancer (nos)
prostate cancer (nos)
small intestinal, colorectal, or anal cancer (nos)
unspecified primary cancer (nos)
Tumor Type

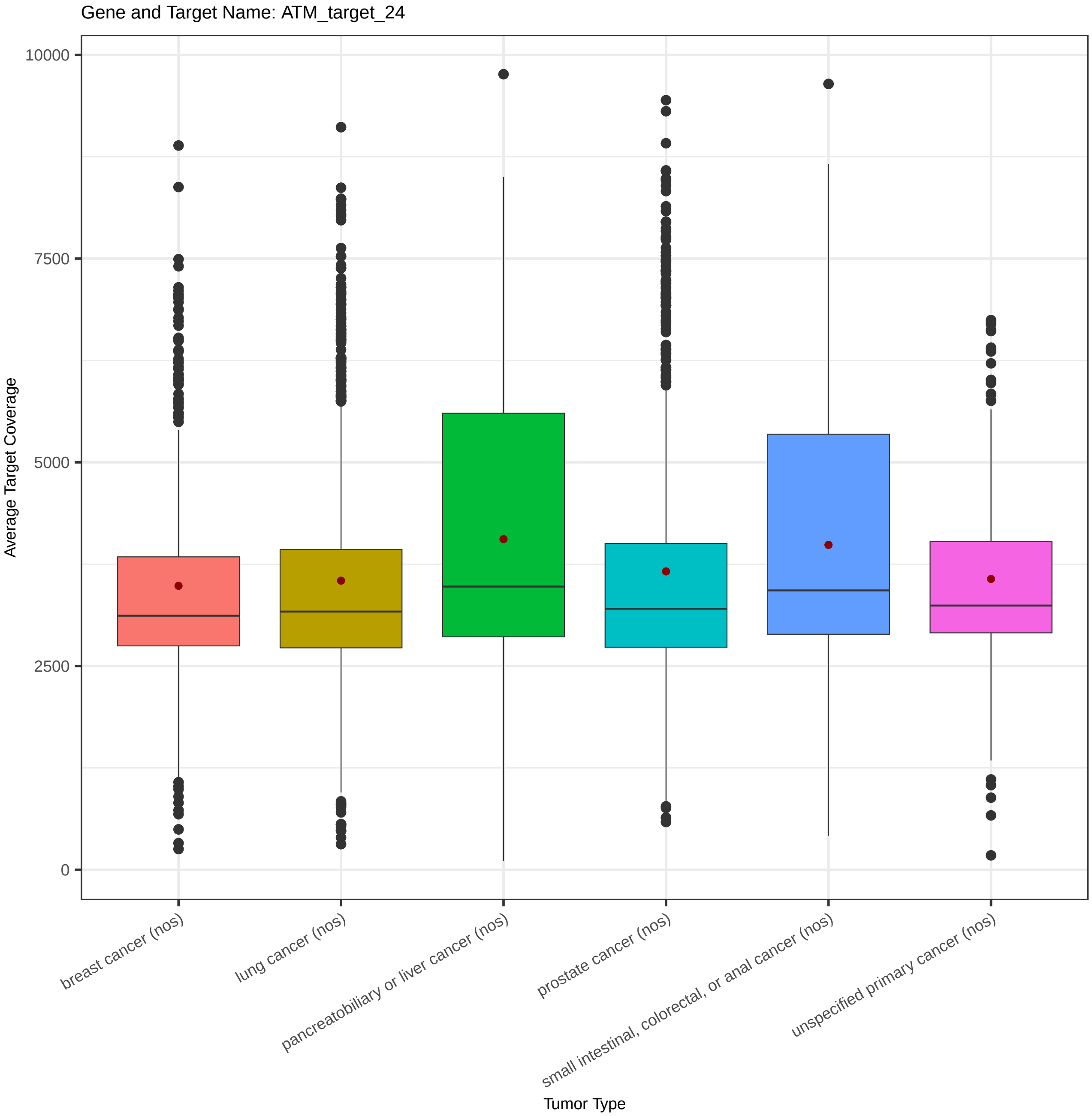

Gene and Target Name: ATM_target_24
Average Target Coverage
10000
7500
5000
2500
0
breast cancer (nos)
lung cancer (nos)
pancreatobiliary or liver cancer (nos)
prostate cancer (nos)
small intestinal, colorectal, or anal cancer (nos)
unspecified primary cancer (nos)
Tumor Type

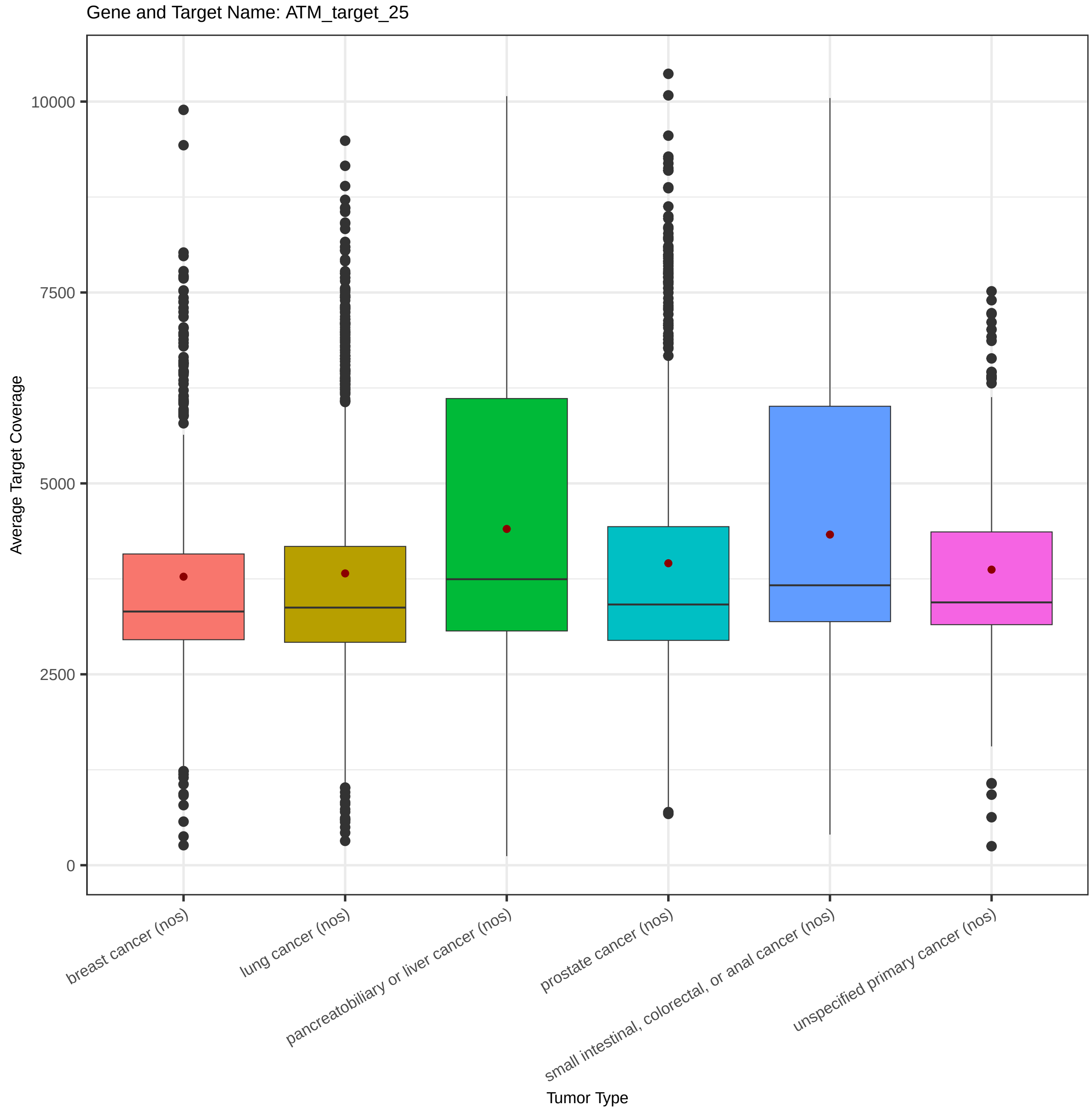
Gene and Target Name: ATM_target_25
Average Target Coverage
10000
7500
5000
2500
0
breast cancer (nos)
lung cancer (nos)
pancreatobiliary or liver cancer (nos)
prostate cancer (nos)
small intestinal, colorectal, or anal cancer (nos)
unspecified primary cancer (nos)
Tumor Type

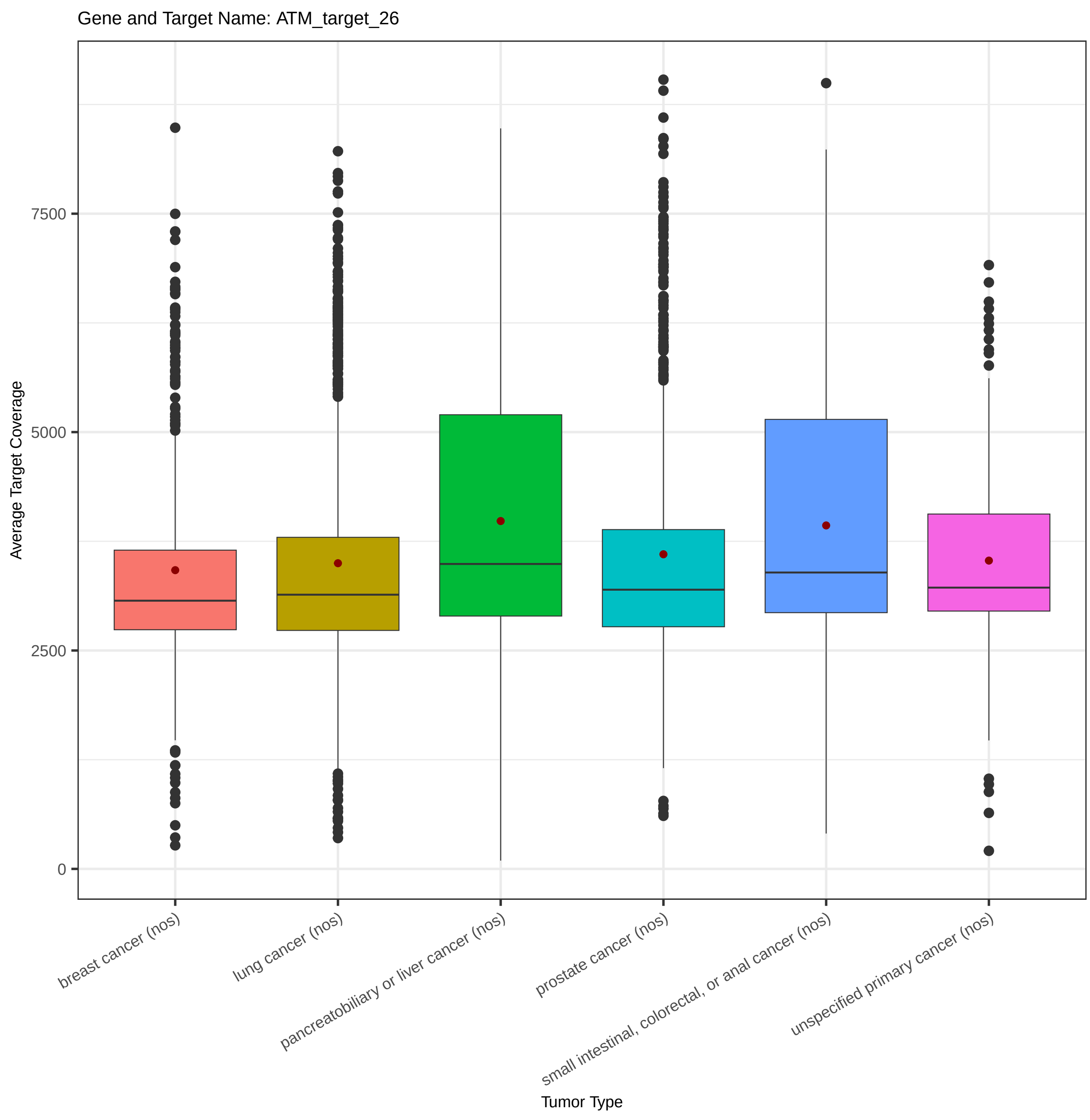

Gene and Target Name: ATM_target_26
Average Target Coverage
7500
5000
2500
0
breast cancer (nos)
lung cancer (nos)
pancreatobiliary or liver cancer (nos)
prostate cancer (nos)
small intestinal, colorectal, or anal cancer (nos)
unspecified primary cancer (nos)
Tumor Type

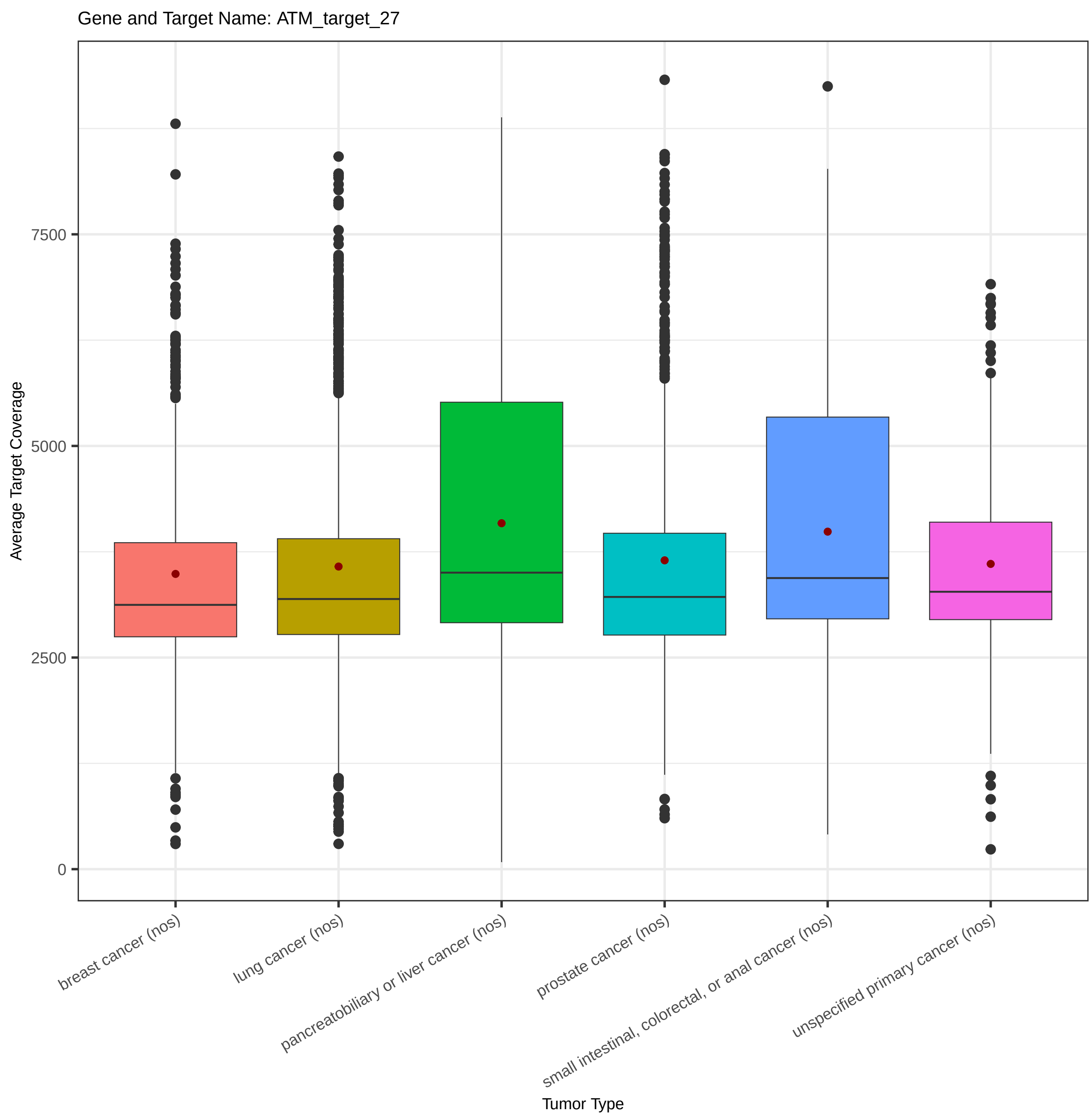
Gene and Target Name: ATM_target_27
Average Target Coverage
7500
5000
2500
0
breast cancer (nos)
lung cancer (nos)
pancreatobiliary or liver cancer (nos)
prostate cancer (nos)
small intestinal, colorectal, or anal cancer (nos)
unspecified primary cancer (nos)
Tumor Type

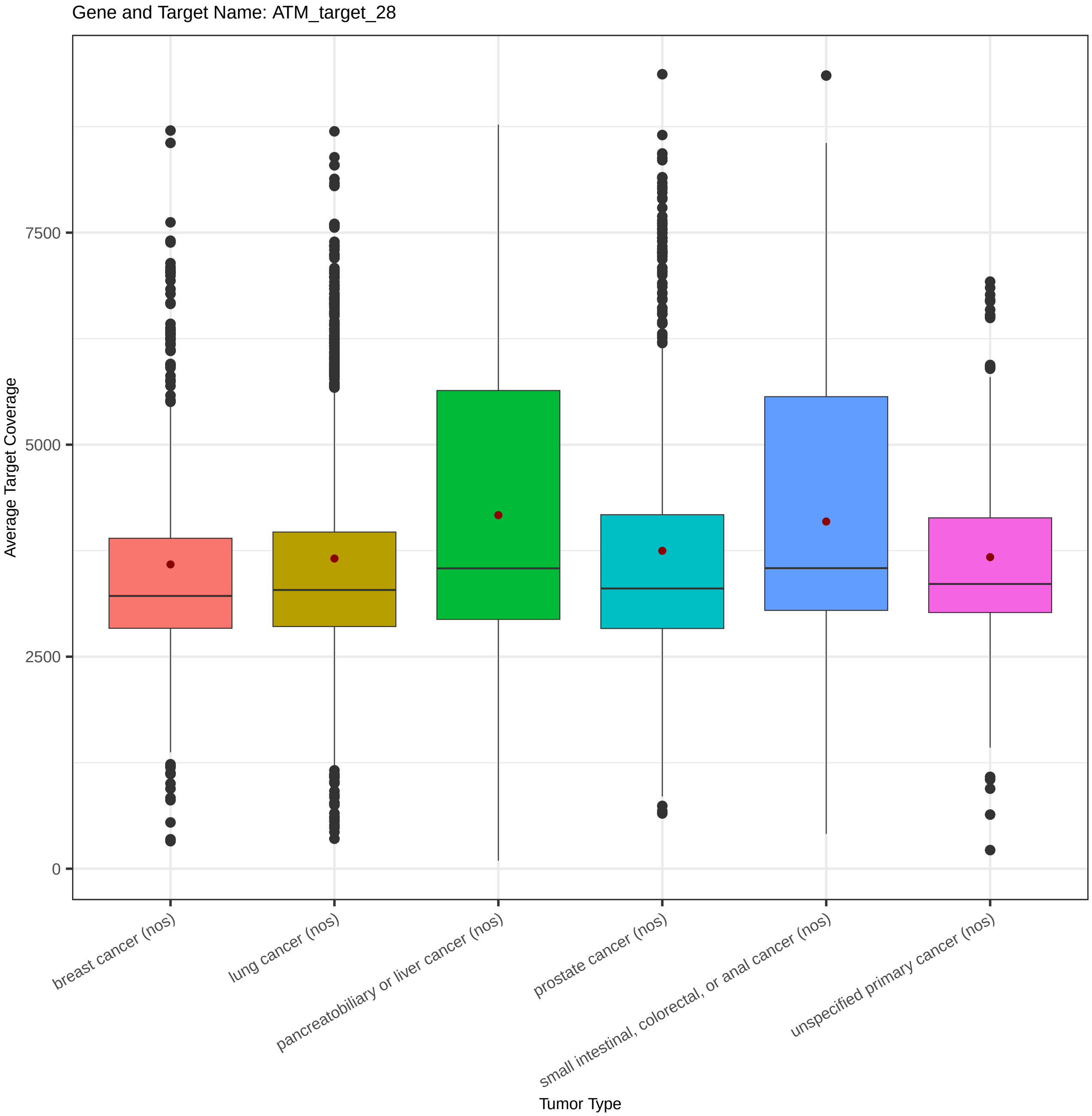

Gene and Target Name: ATM_target_28
Average Target Coverage
7500
5000
2500
0
breast cancer (nos)
lung cancer (nos)
pancreatobiliary or liver cancer (nos)
prostate cancer (nos)
small intestinal, colorectal, or anal cancer (nos)
unspecified primary cancer (nos)
Tumor Type

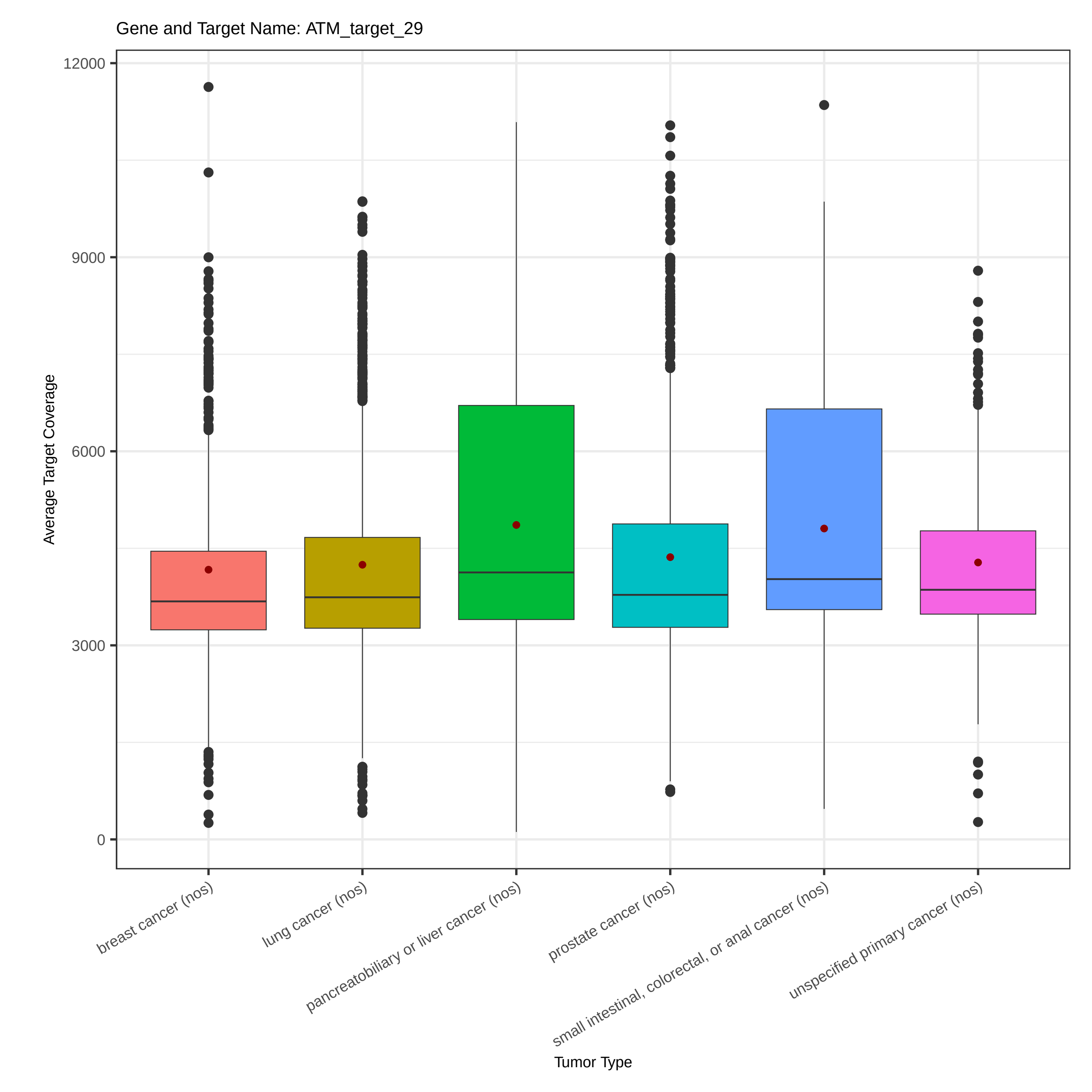

Gene and Target Name: ATM_target_29
Average Target Coverage
12000
9000
6000
3000
0
breast cancer (nos)
lung cancer (nos)
pancreatobiliary or liver cancer (nos)
prostate cancer (nos)
small intestinal, colorectal, or anal cancer (nos)
unspecified primary cancer (nos)
Tumor Type

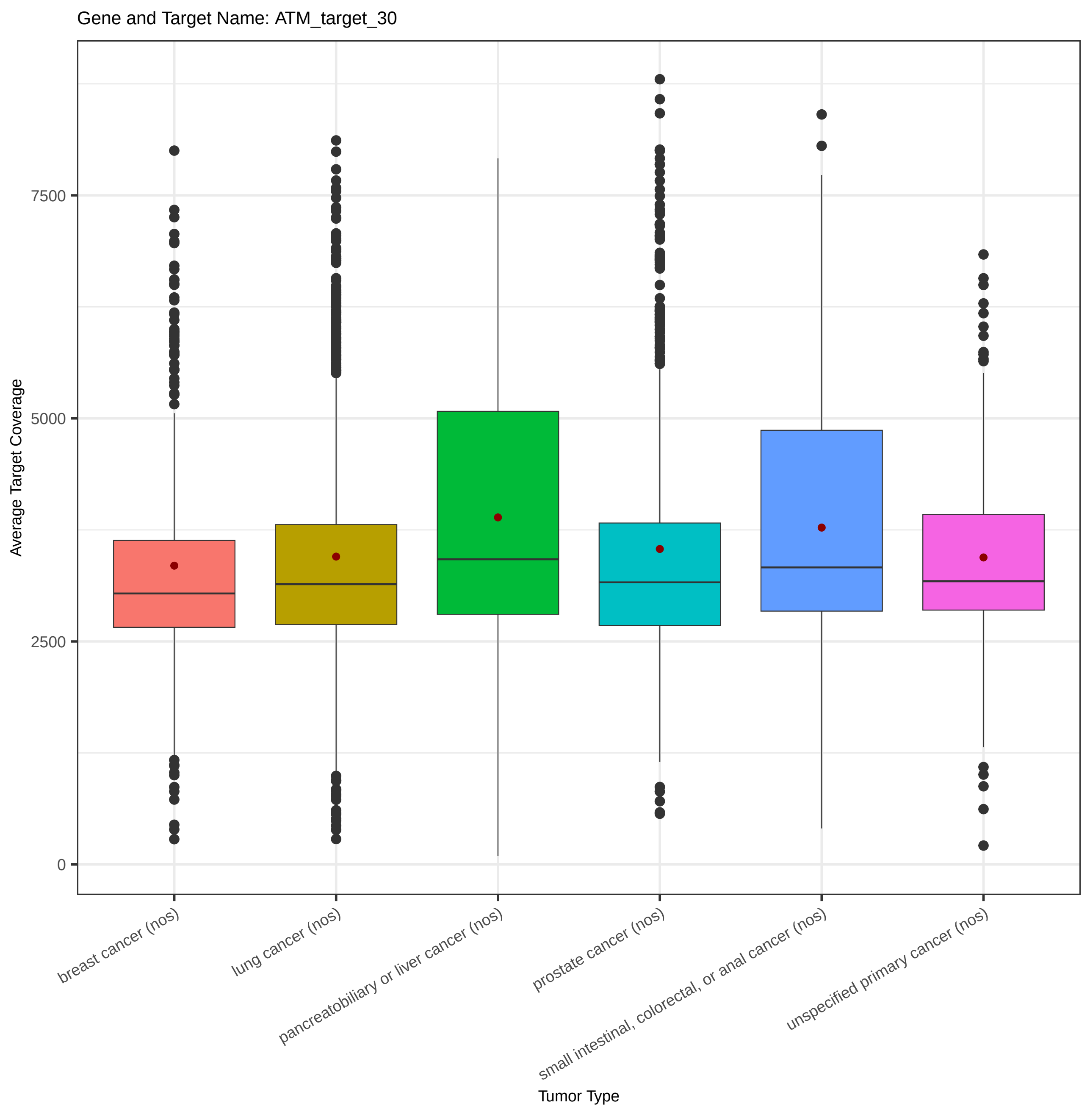

Gene and Target Name: ATM_target_30
Average Target Coverage
7500
5000
2500
0
breast cancer (nos)
lung cancer (nos)
pancreatobiliary or liver cancer (nos)
prostate cancer (nos)
small intestinal, colorectal, or anal cancer (nos)
unspecified primary cancer (nos)
Tumor Type

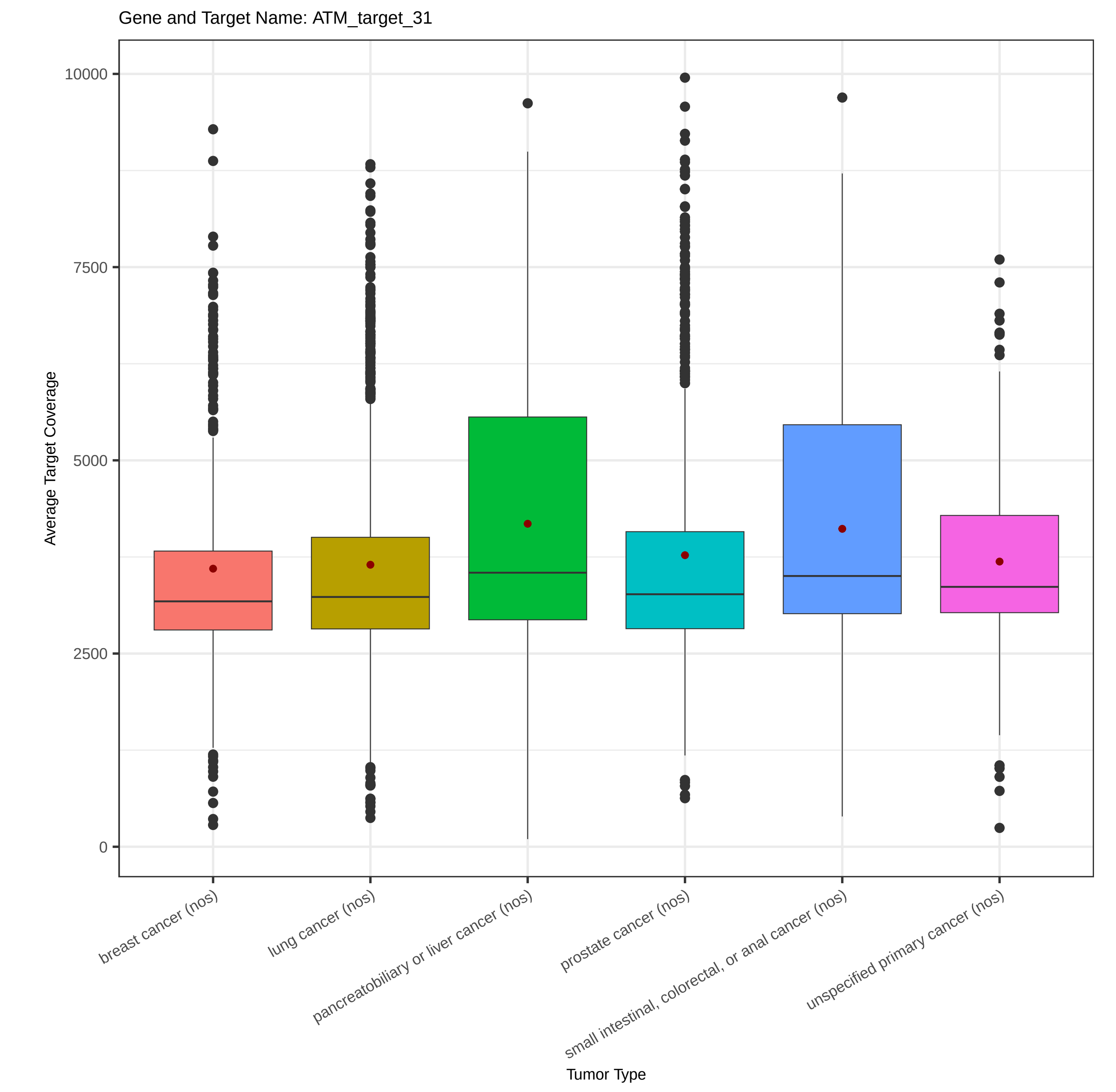
Gene and Target Name: ATM_target_31
Average Target Coverage
10000
7500
5000
2500
0
breast cancer (nos)
lung cancer (nos)
pancreatobiliary or liver cancer (nos)
prostate cancer (nos)
small intestinal, colorectal, or anal cancer (nos)
unspecified primary cancer (nos)
Tumor Type

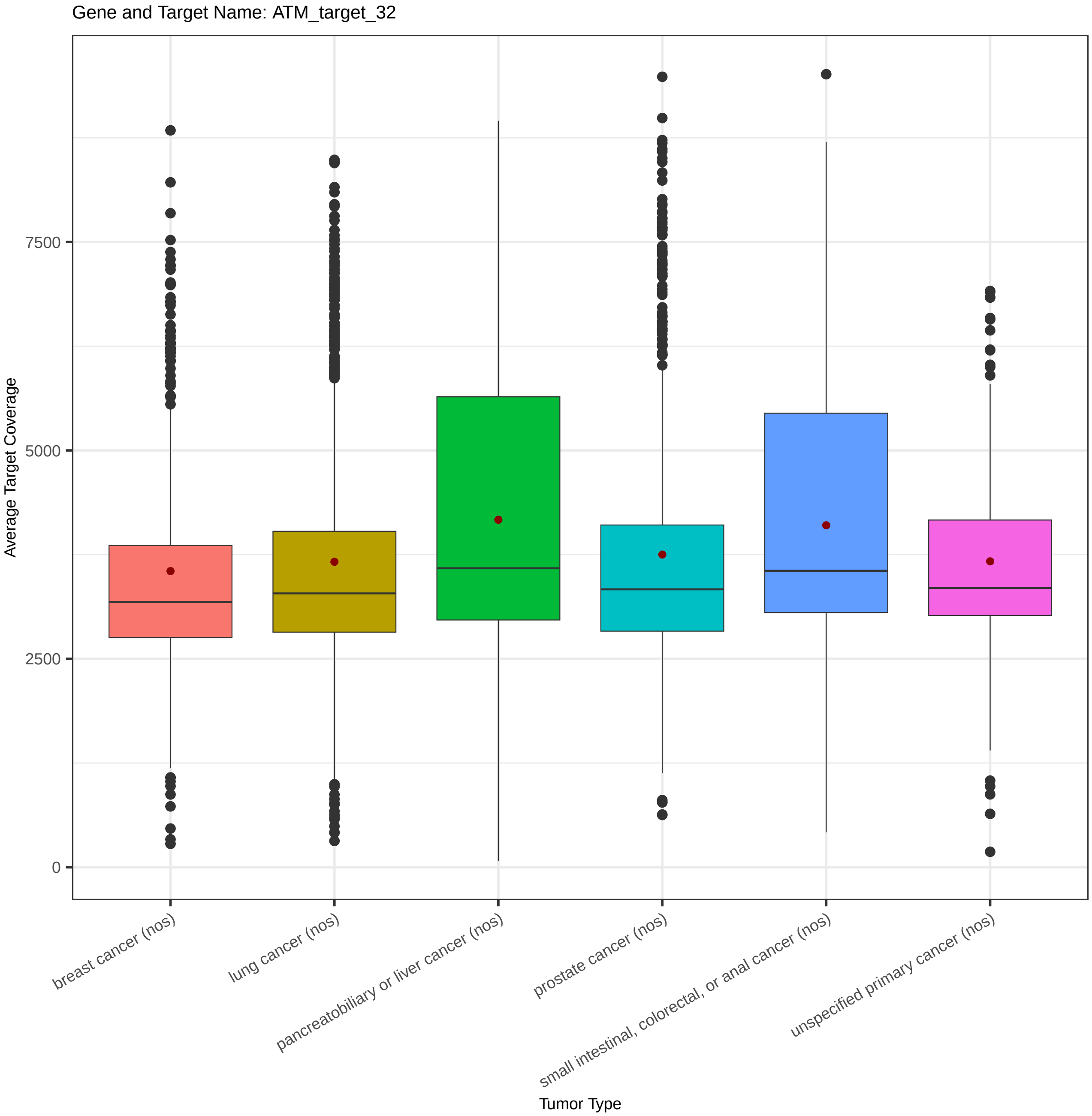
Gene and Target Name: ATM_target_32
Average Target Coverage
0
2500
5000
7500
breast cancer (nos)
lung cancer (nos)
pancreatobiliary or liver cancer (nos)
prostate cancer (nos)
small intestinal, colorectal, or anal cancer (nos)
unspecified primary cancer (nos)
Tumor Type

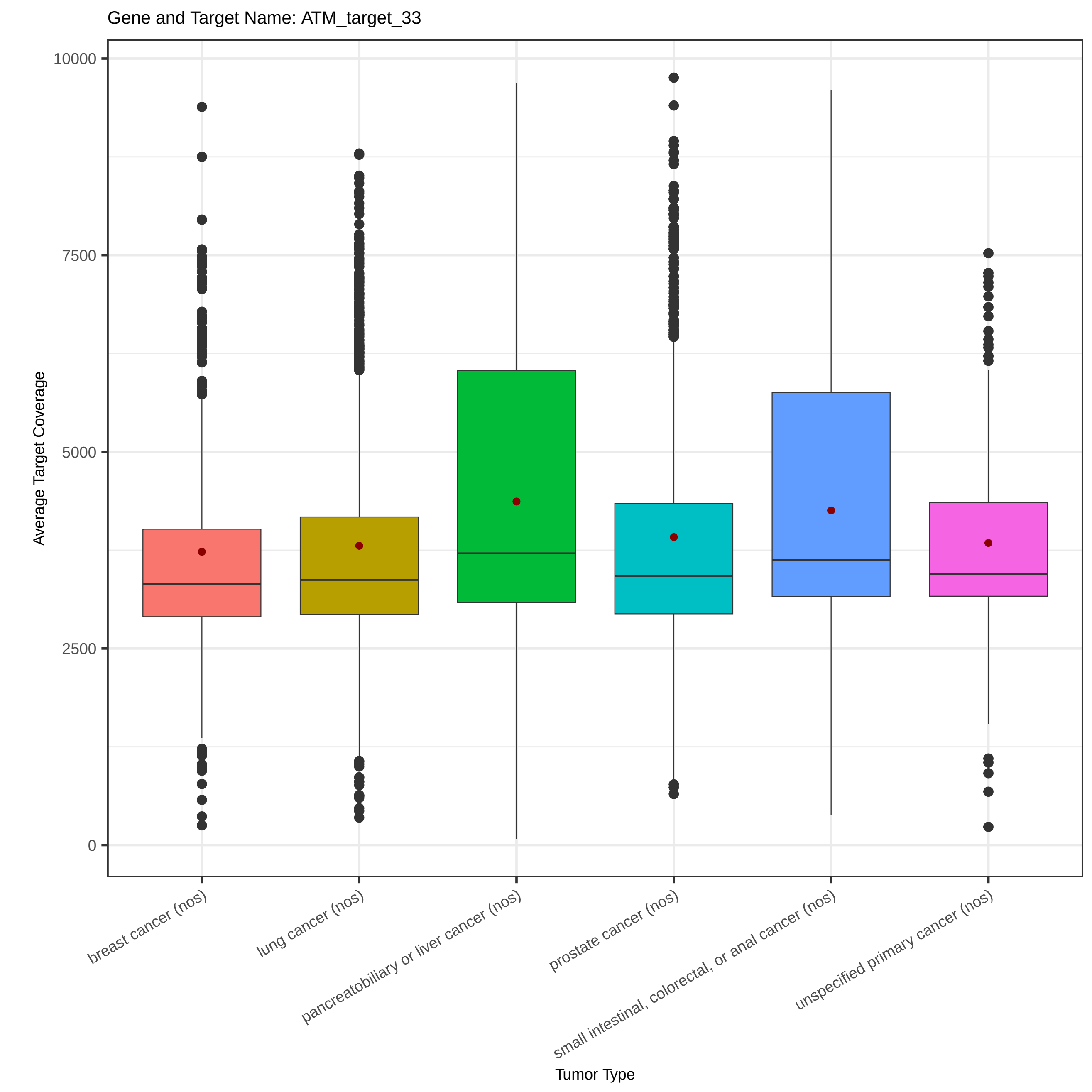
Gene and Target Name: ATM_target_33
Average Target Coverage
10000
7500
5000
2500
0
breast cancer (nos)
lung cancer (nos)
pancreatobiliary or liver cancer (nos)
prostate cancer (nos)
small intestinal, colorectal, or anal cancer (nos)
unspecified primary cancer (nos)
Tumor Type

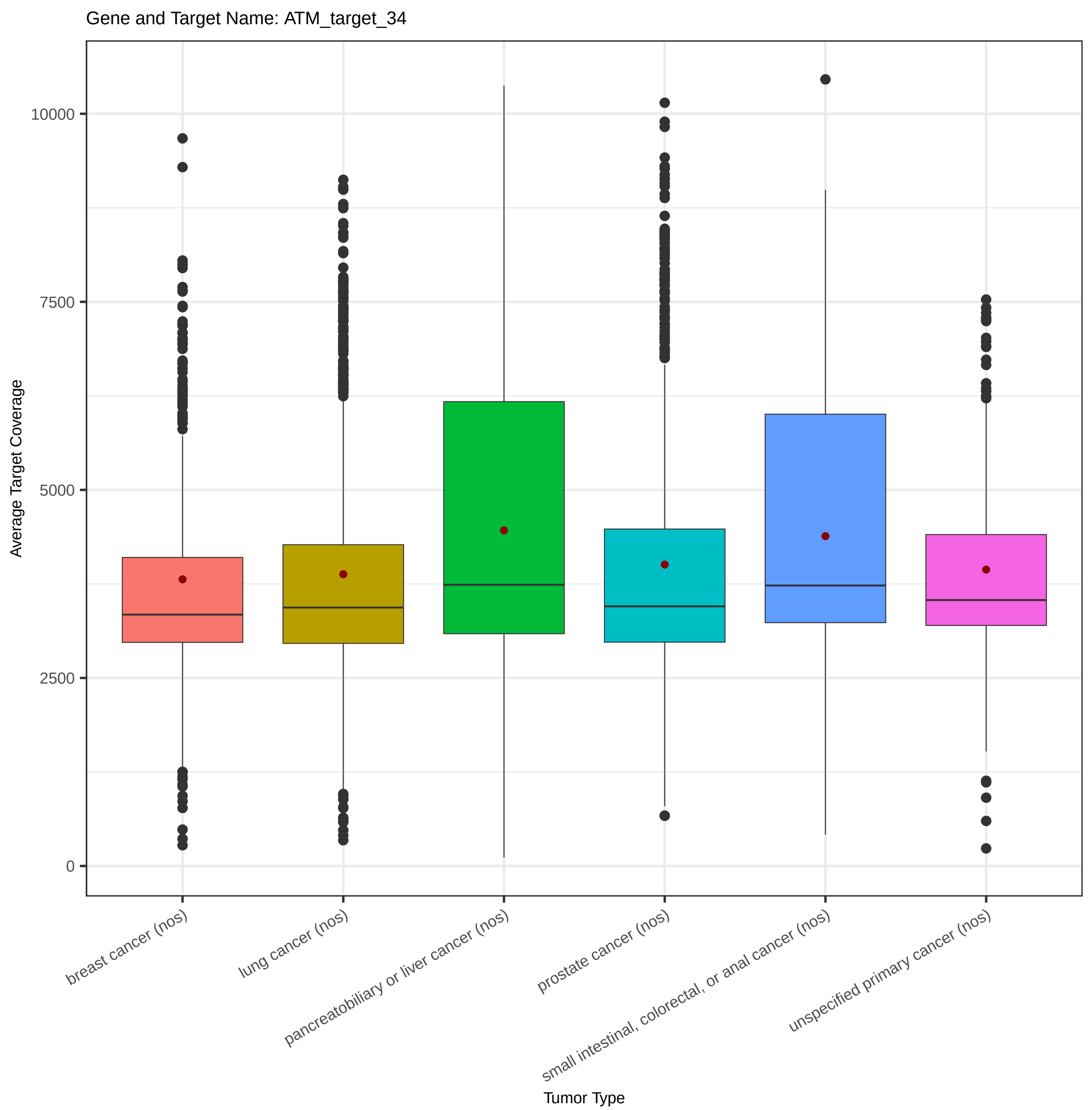
Gene and Target Name: ATM_target_34
Average Target Coverage
10000
7500
5000
2500
0
breast cancer (nos)
lung cancer (nos)
pancreatobiliary or liver cancer (nos)
prostate cancer (nos)
small intestinal, colorectal, or anal cancer (nos)
unspecified primary cancer (nos)
Tumor Type

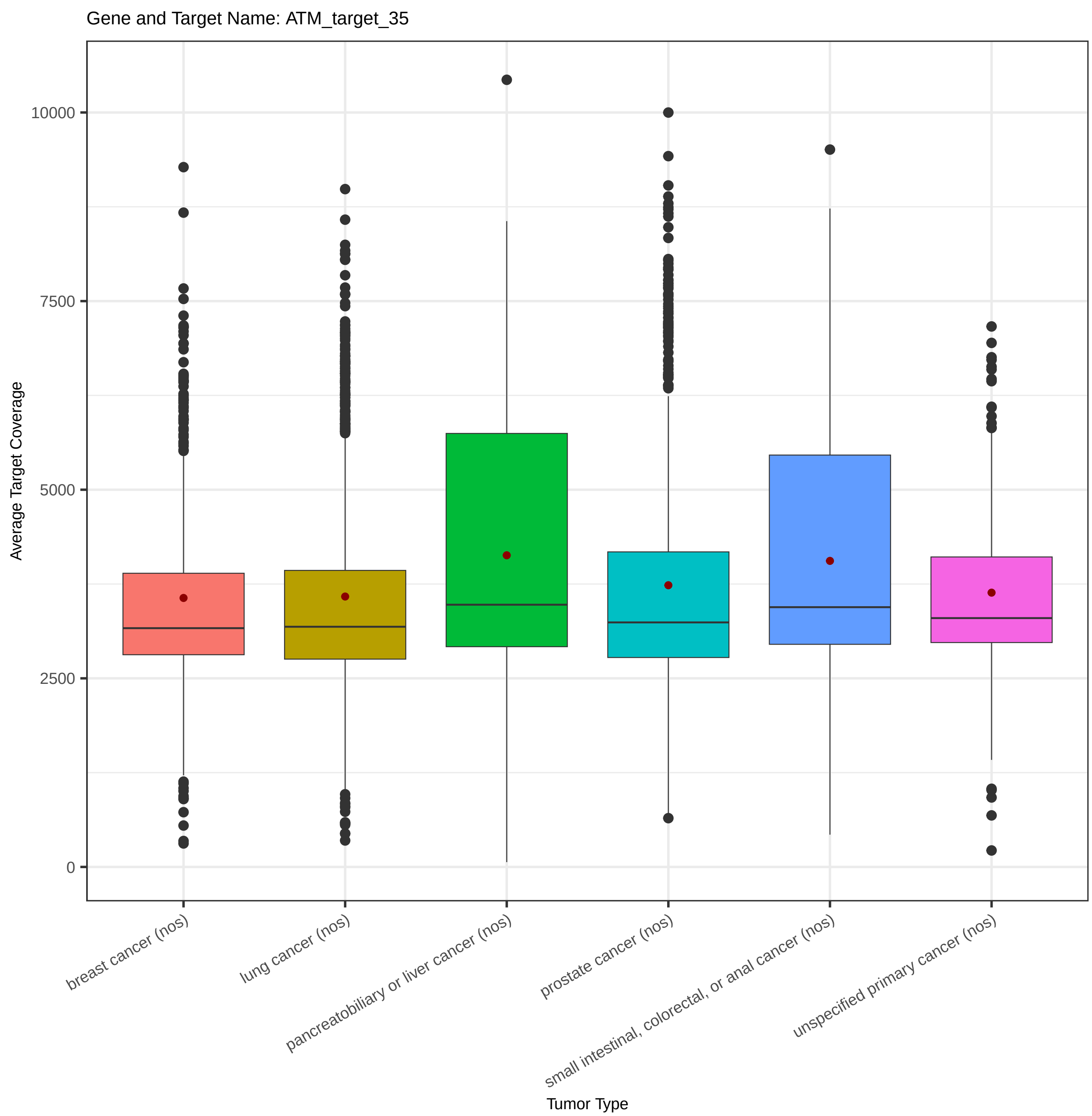

Gene and Target Name: ATM_target_35
Average Target Coverage
10000
7500
5000
2500
0
breast cancer (nos)
lung cancer (nos)
pancreatobiliary or liver cancer (nos)
prostate cancer (nos)
small intestinal, colorectal, or anal cancer (nos)
unspecified primary cancer (nos)
Tumor Type

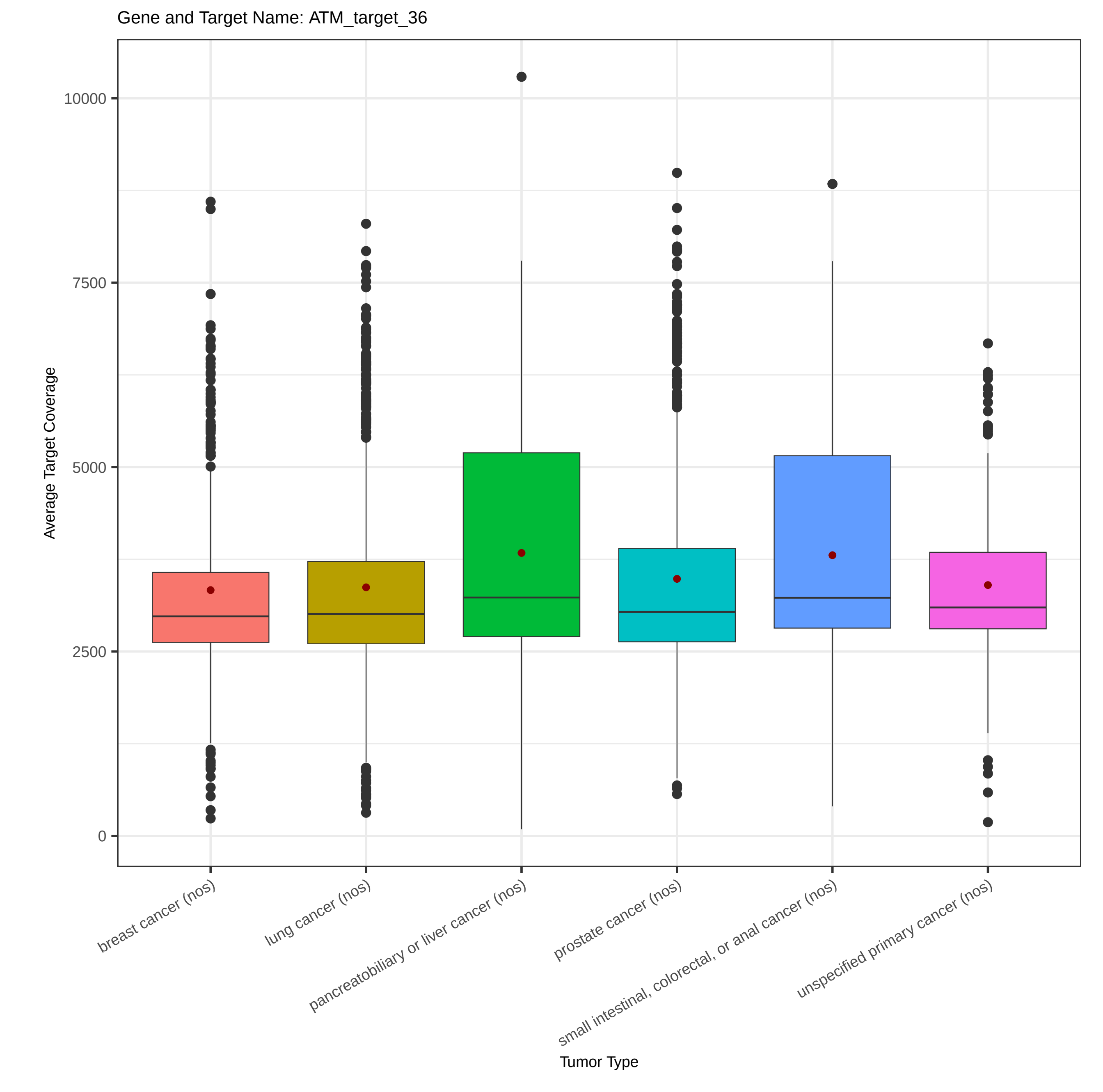
Gene and Target Name: ATM_target_36
Average Target Coverage
10000
7500
5000
2500
0
breast cancer (nos)
lung cancer (nos)
pancreatobiliary or liver cancer (nos)
prostate cancer (nos)
small intestinal, colorectal, or anal cancer (nos)
unspecified primary cancer (nos)
Tumor Type

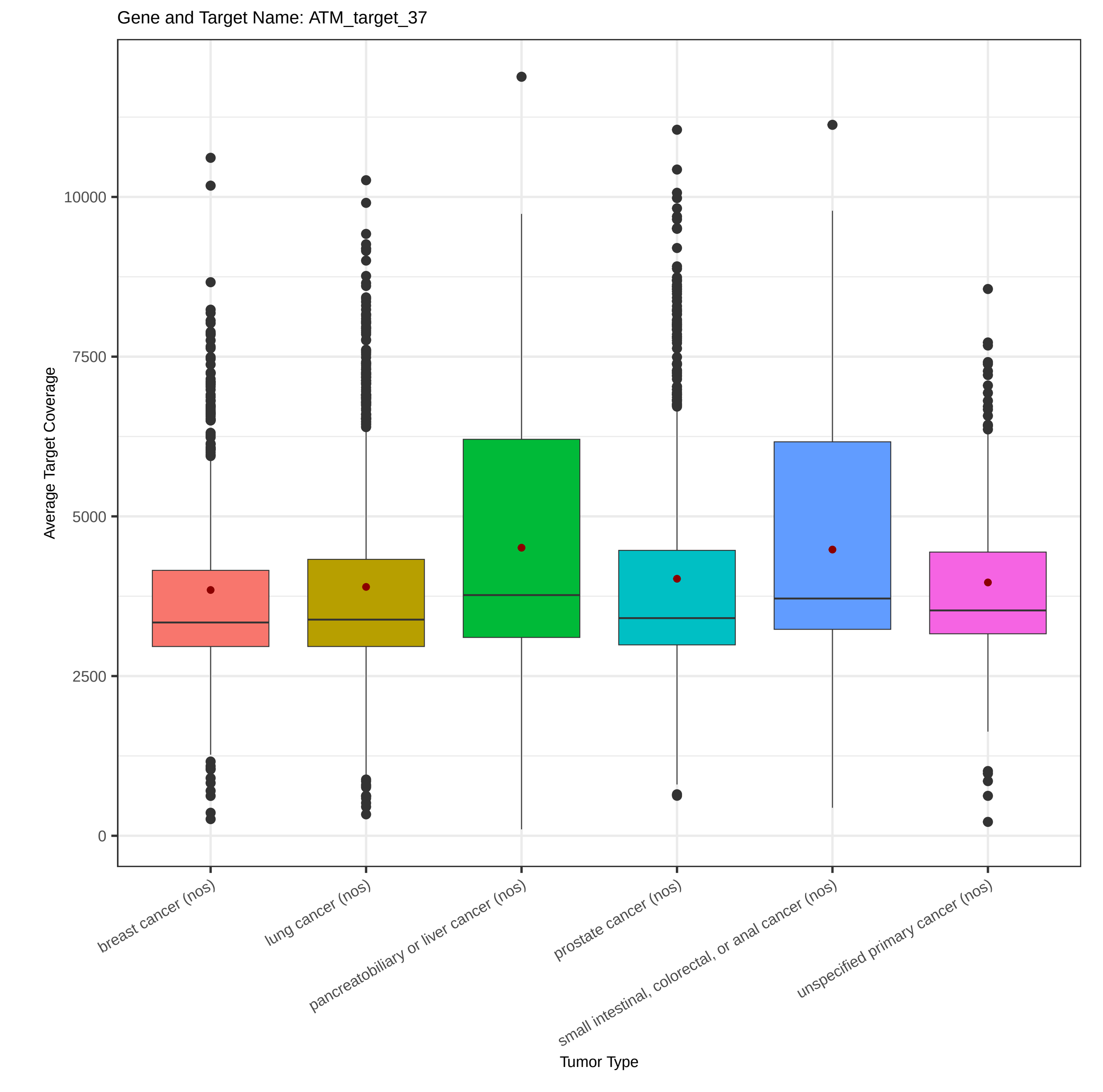

Gene and Target Name: ATM_target_37
Average Target Coverage
0
2500
5000
7500
10000
breast cancer (nos)
lung cancer (nos)
pancreatobiliary or liver cancer (nos)
prostate cancer (nos)
small intestinal, colorectal, or anal cancer (nos)
unspecified primary cancer (nos)
Tumor Type

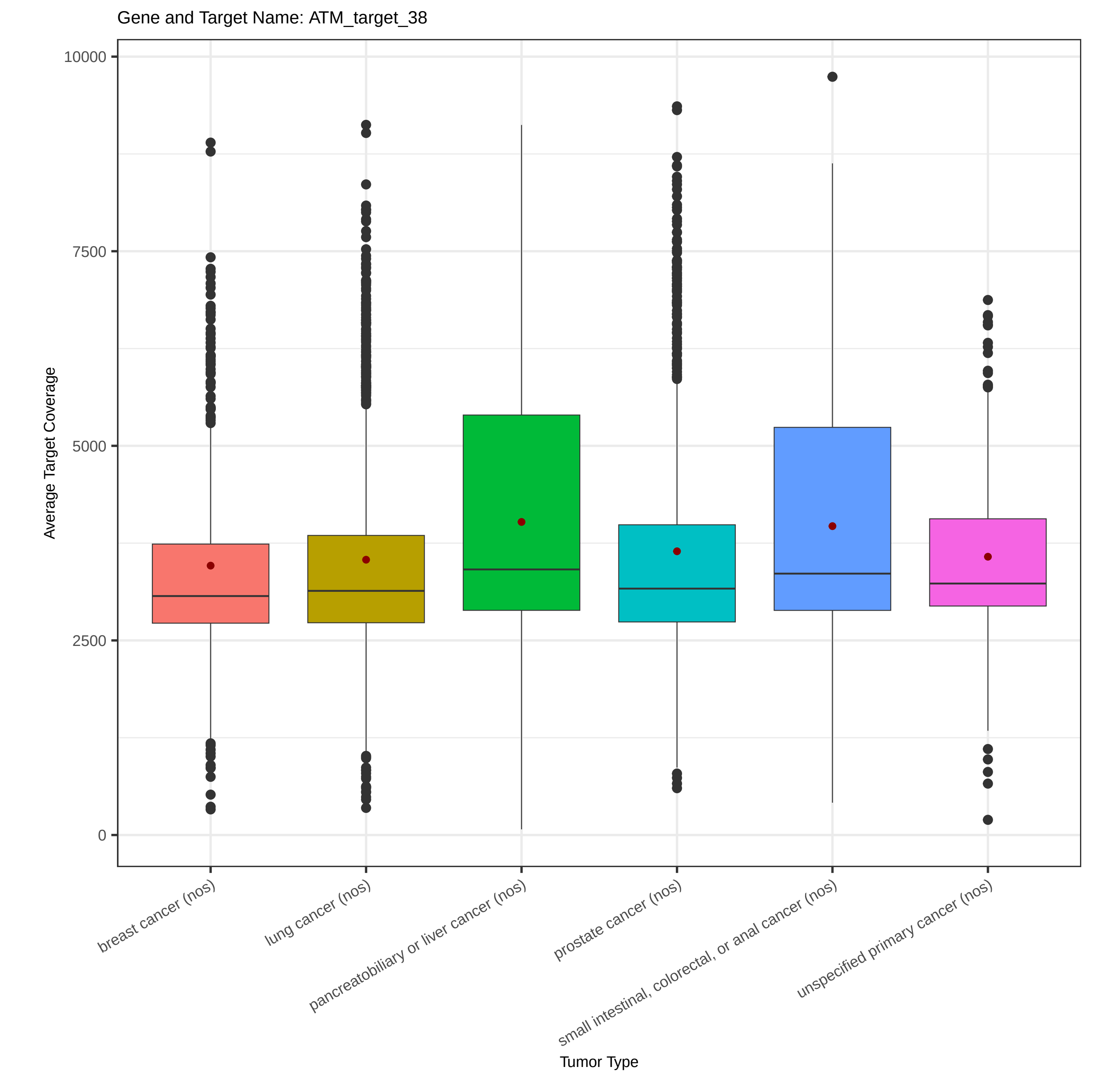

Gene and Target Name: ATM_target_38
Average Target Coverage
10000
7500
5000
2500
0
breast cancer (nos)
lung cancer (nos)
pancreatobiliary or liver cancer (nos)
prostate cancer (nos)
small intestinal, colorectal, or anal cancer (nos)
unspecified primary cancer (nos)
Tumor Type

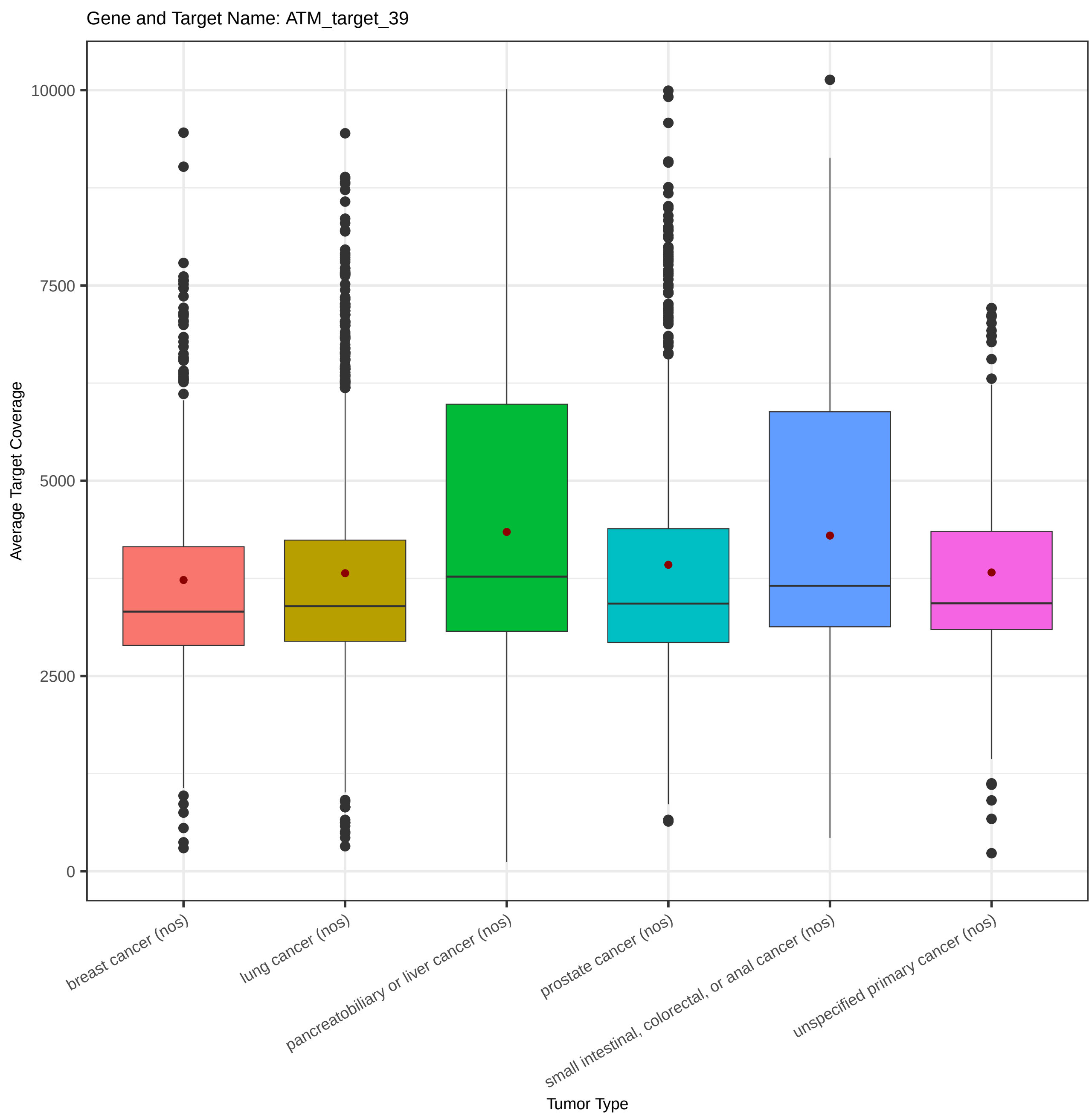

Gene and Target Name: ATM_target_39
Average Target Coverage
10000
7500
5000
2500
0
breast cancer (nos)
lung cancer (nos)
pancreatobiliary or liver cancer (nos)
prostate cancer (nos)
small intestinal, colorectal, or anal cancer (nos)
unspecified primary cancer (nos)
Tumor Type

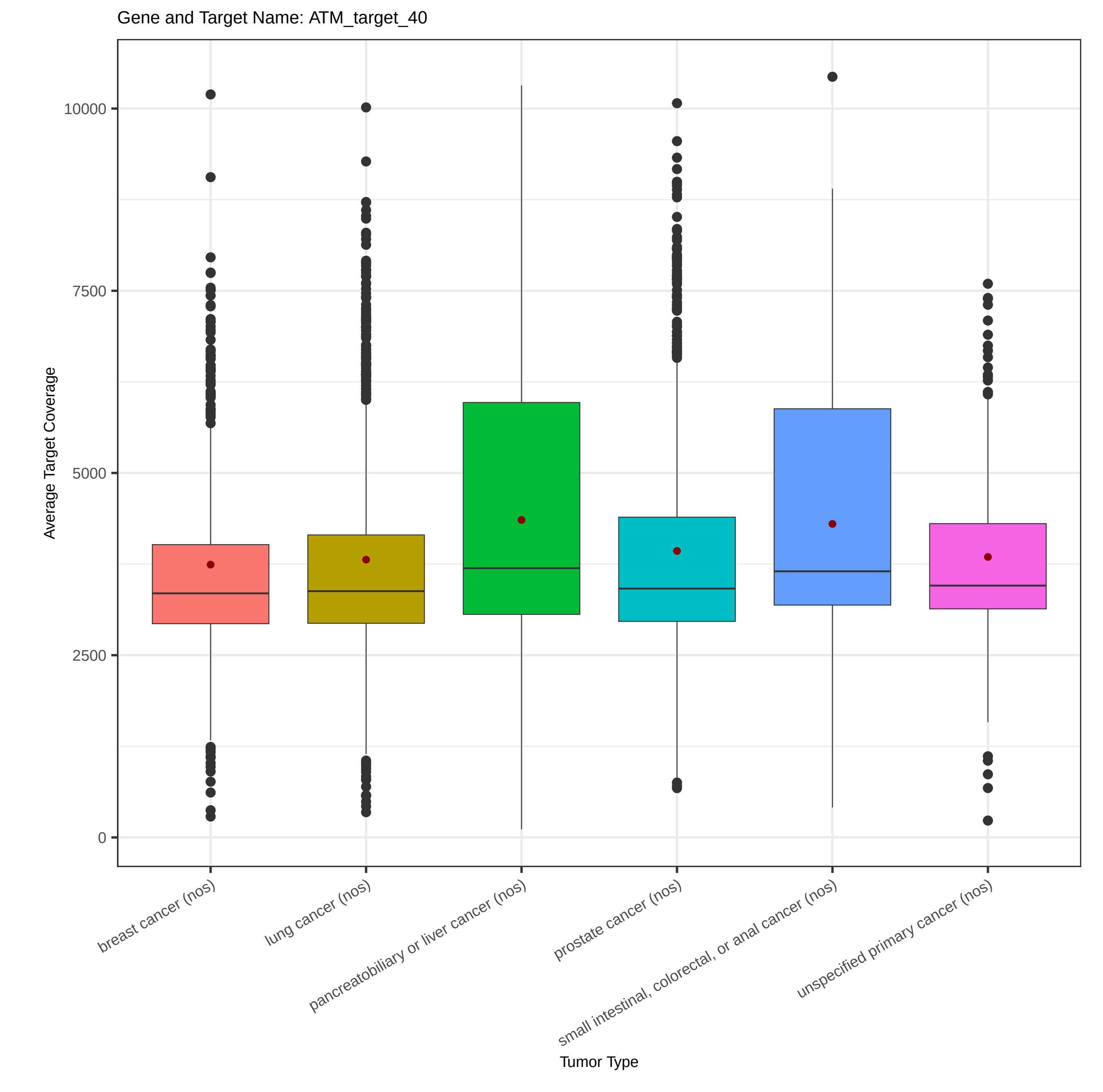

Gene and Target Name: ATM_target_40
Average Target Coverage
10000
7500
5000
2500
0
breast cancer (nos)
lung cancer (nos)
pancreatobiliary or liver cancer (nos)
prostate cancer (nos)
small intestinal, colorectal, or anal cancer (nos)
unspecified primary cancer (nos)
Tumor Type

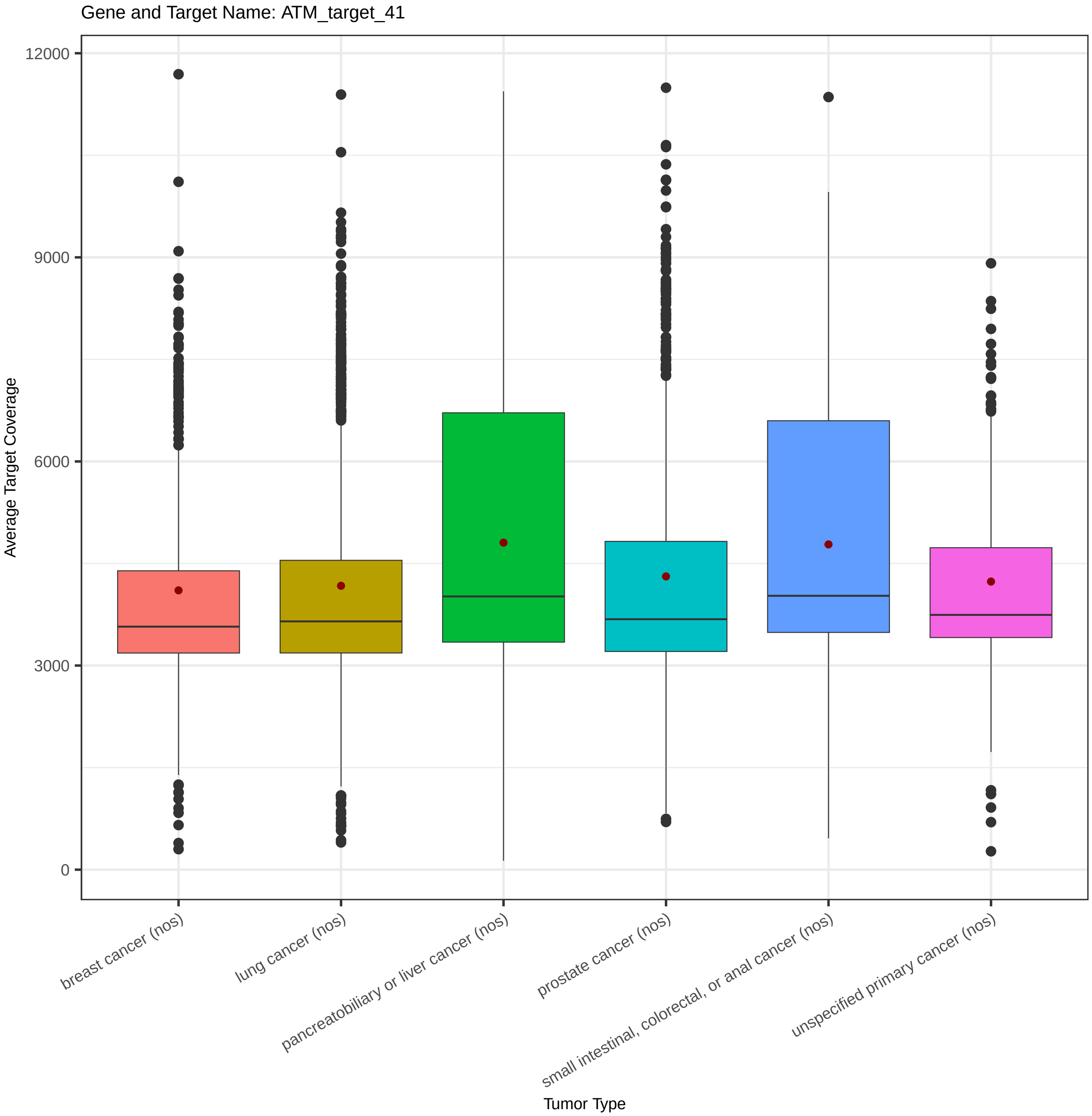

Gene and Target Name: ATM_target_41
Average Target Coverage
12000
9000
6000
3000
0
breast cancer (nos)
lung cancer (nos)
pancreatobiliary or liver cancer (nos)
prostate cancer (nos)
small intestinal, colorectal, or anal cancer (nos)
unspecified primary cancer (nos)
Tumor Type

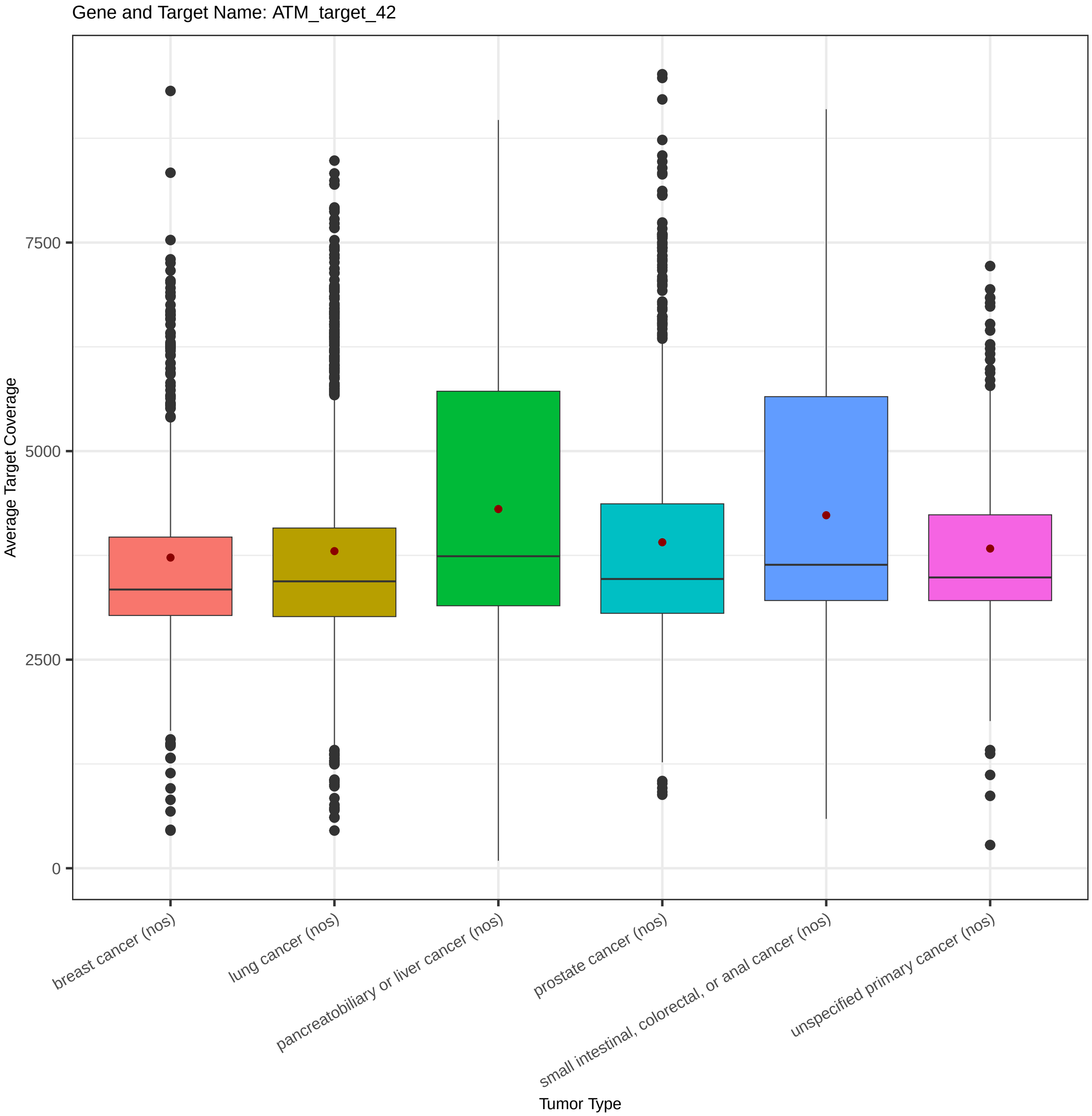
Gene and Target Name: ATM_target_42
Average Target Coverage
7500
5000
2500
0
breast cancer (nos)
lung cancer (nos)
pancreatobiliary or liver cancer (nos)
prostate cancer (nos)
small intestinal, colorectal, or anal cancer (nos)
unspecified primary cancer (nos)
Tumor Type

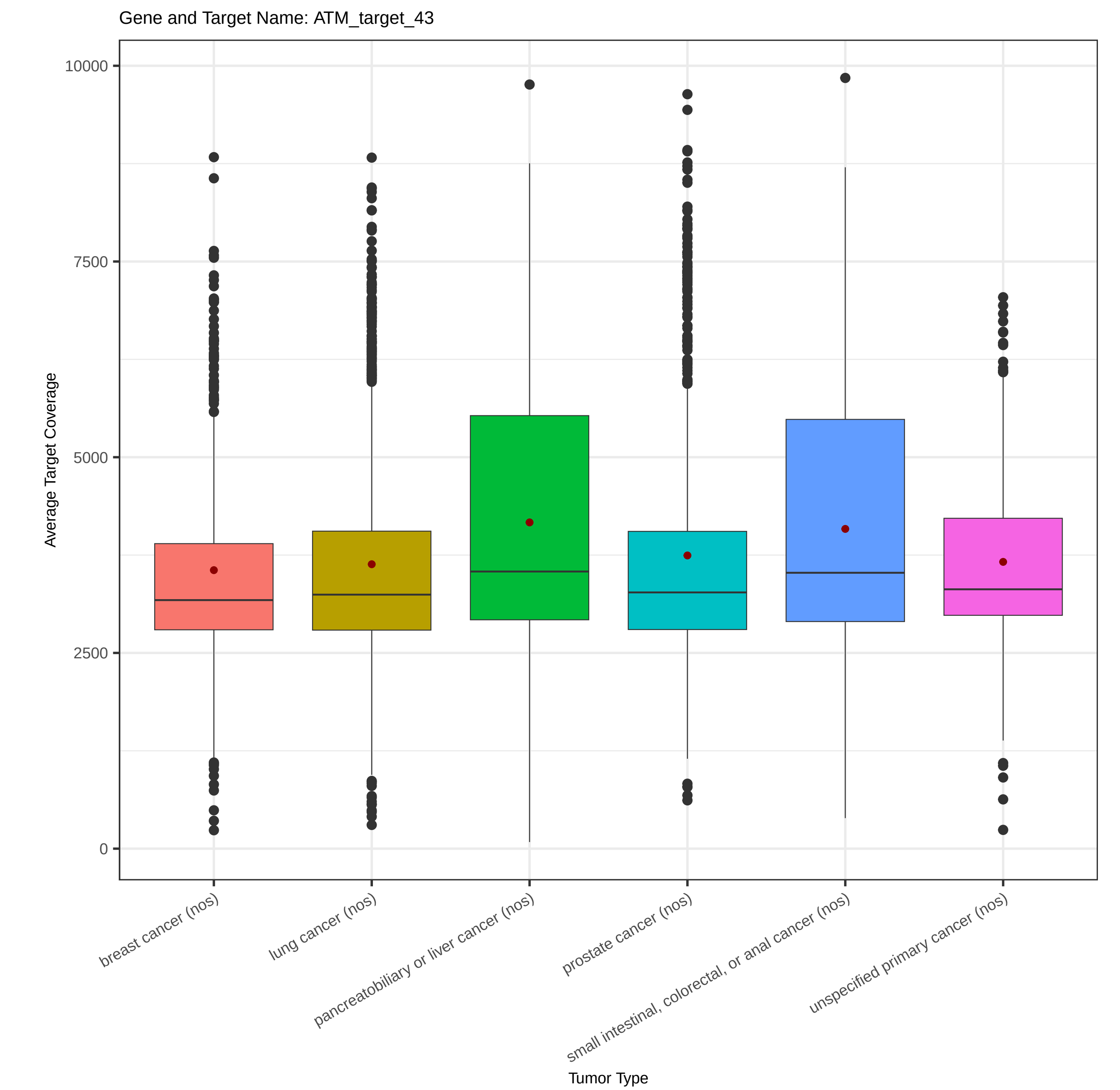

Gene and Target Name: ATM_target_43
Average Target Coverage
10000
7500
5000
2500
0
breast cancer (nos)
lung cancer (nos)
pancreatobiliary or liver cancer (nos)
prostate cancer (nos)
small intestinal, colorectal, or anal cancer (nos)
unspecified primary cancer (nos)
Tumor Type

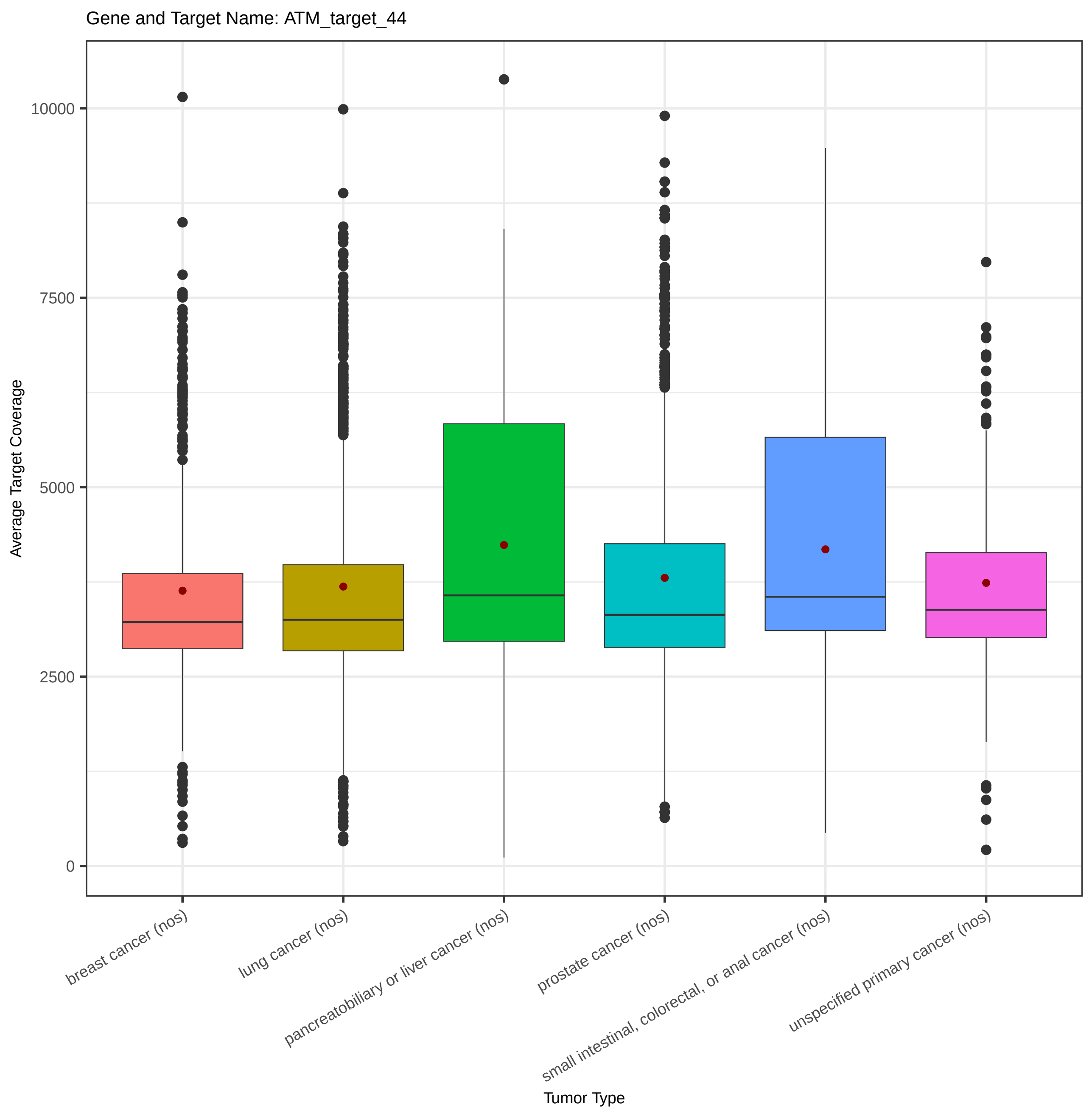
Gene and Target Name: ATM_target_44
Average Target Coverage
10000
7500
5000
2500
0
breast cancer (nos)
lung cancer (nos)
pancreatobiliary or liver cancer (nos)
prostate cancer (nos)
small intestinal, colorectal, or anal cancer (nos)
unspecified primary cancer (nos)
Tumor Type

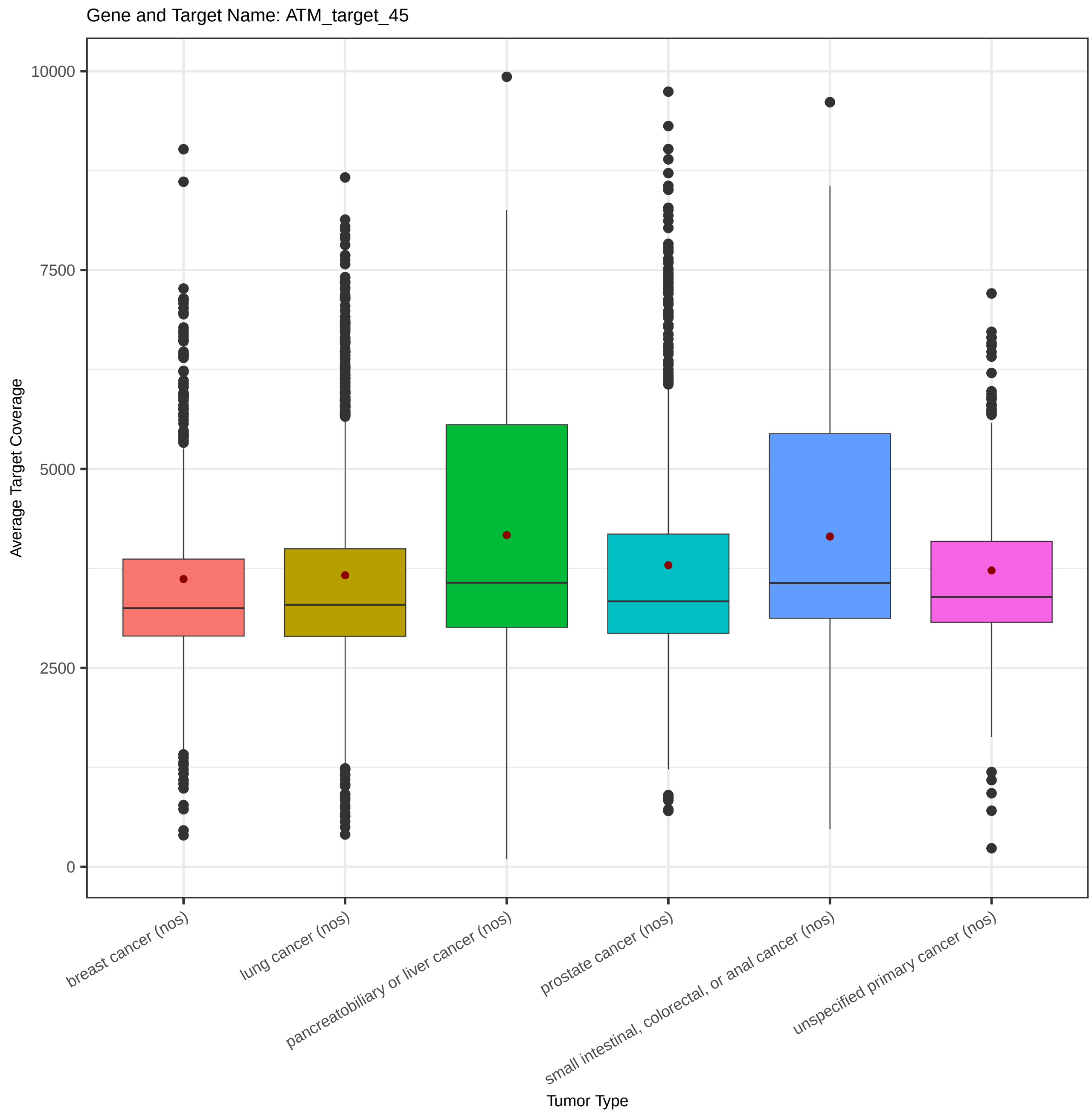
Gene and Target Name: ATM_target_45
Average Target Coverage
10000
7500
5000
2500
0
breast cancer (nos)
lung cancer (nos)
pancreatobiliary or liver cancer (nos)
prostate cancer (nos)
small intestinal, colorectal, or anal cancer (nos)
unspecified primary cancer (nos)
Tumor Type

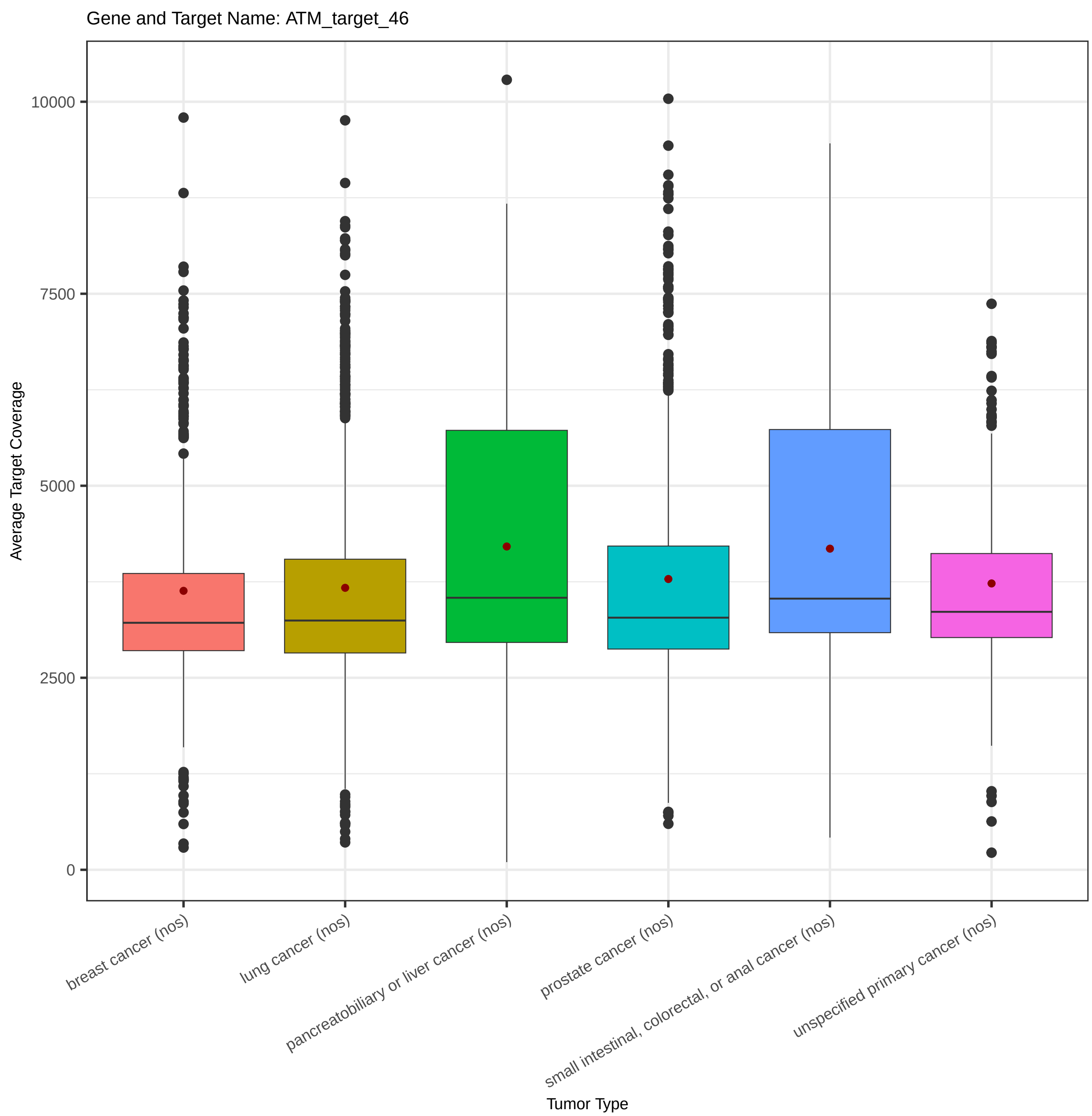
Gene and Target Name: ATM_target_46
Average Target Coverage
10000
7500
5000
2500
0
breast cancer (nos)
lung cancer (nos)
pancreatobiliary or liver cancer (nos)
prostate cancer (nos)
small intestinal, colorectal, or anal cancer (nos)
unspecified primary cancer (nos)
Tumor Type

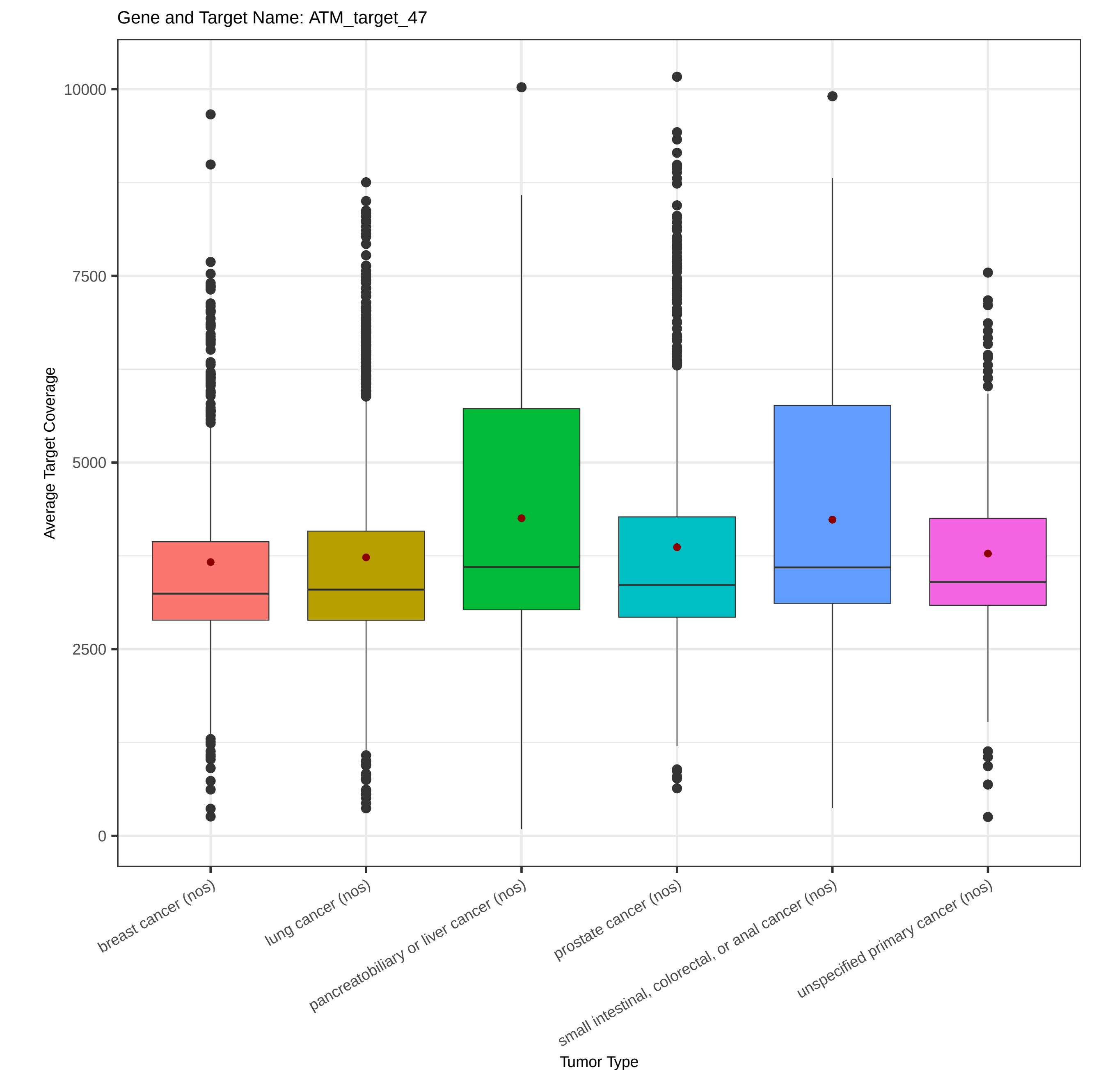

Gene and Target Name: ATM_target_47
Average Target Coverage
10000
7500
5000
2500
0
breast cancer (nos)
lung cancer (nos)
pancreatobiliary or liver cancer (nos)
prostate cancer (nos)
small intestinal, colorectal, or anal cancer (nos)
unspecified primary cancer (nos)
Tumor Type

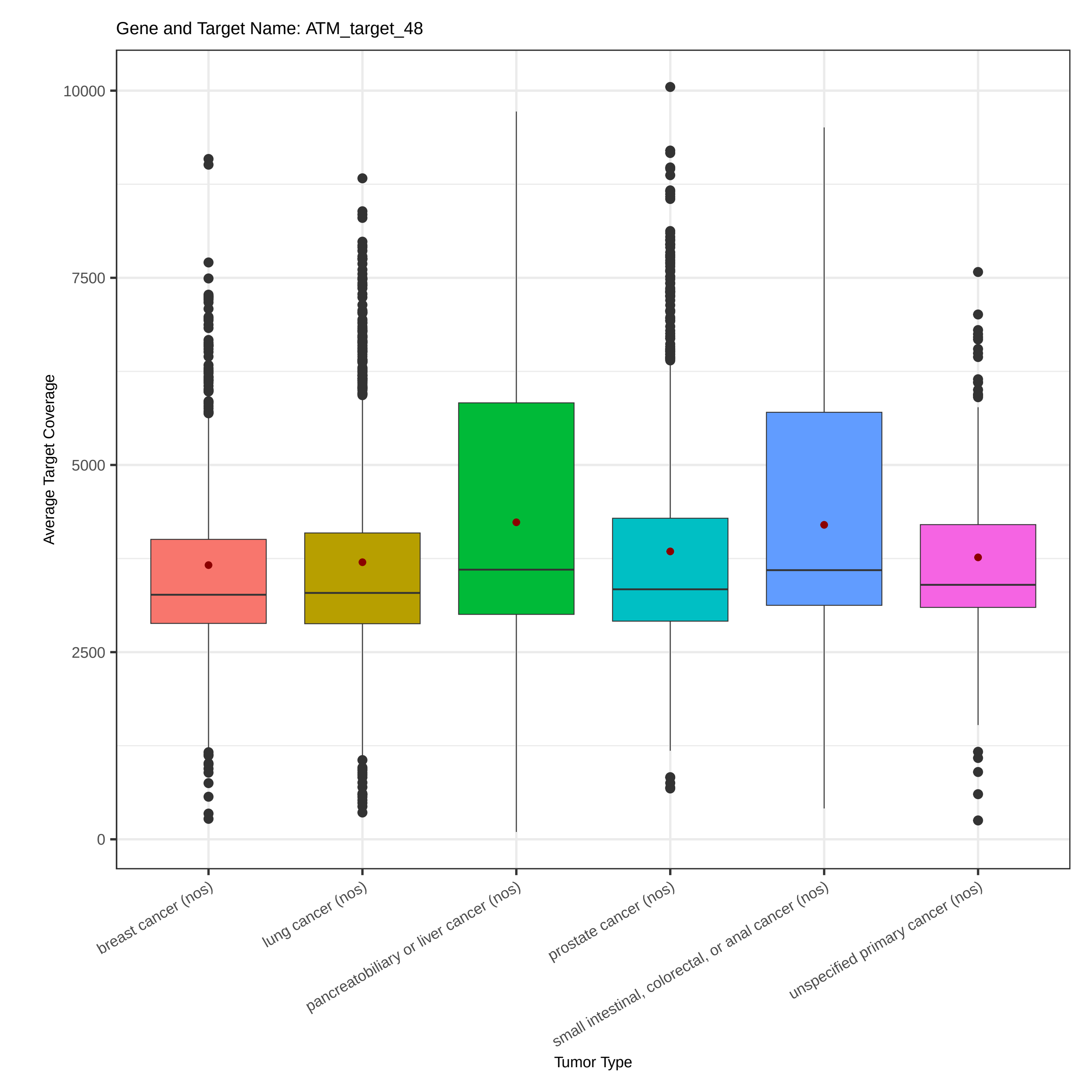

Gene and Target Name: ATM_target_48
Average Target Coverage
10000
7500
5000
2500
0
breast cancer (nos)
lung cancer (nos)
pancreatobiliary or liver cancer (nos)
prostate cancer (nos)
small intestinal, colorectal, or anal cancer (nos)
unspecified primary cancer (nos)
Tumor Type

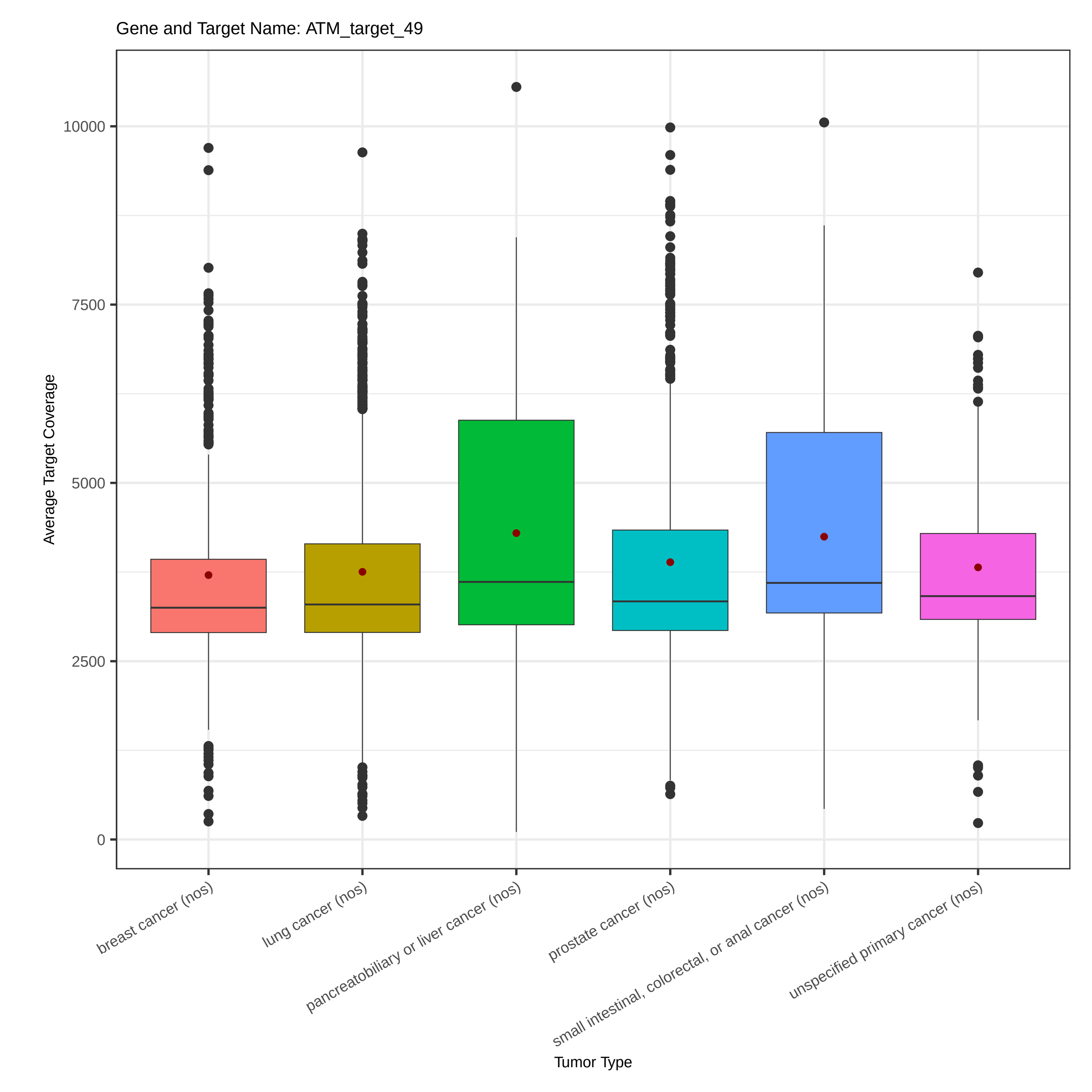
Gene and Target Name: ATM_target_49
Average Target Coverage
10000
7500
5000
2500
0
breast cancer (nos)
lung cancer (nos)
pancreatobiliary or liver cancer (nos)
prostate cancer (nos)
small intestinal, colorectal, or anal cancer (nos)
unspecified primary cancer (nos)
Tumor Type

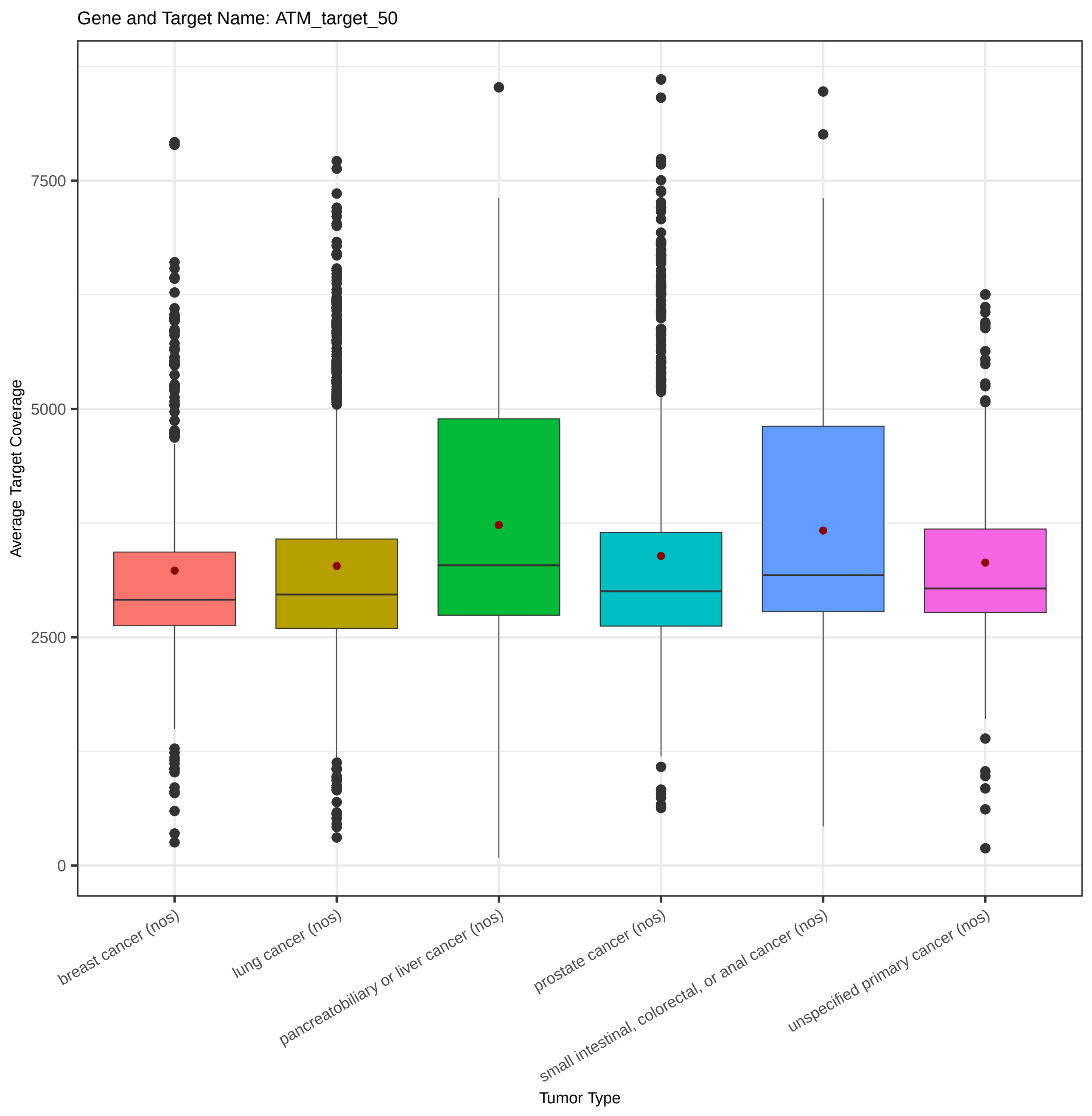

Gene and Target Name: ATM_target_50
Average Target Coverage
7500
5000
2500
0
breast cancer (nos)
lung cancer (nos)
pancreatobiliary or liver cancer (nos)
prostate cancer (nos)
small intestinal, colorectal, or anal cancer (nos)
unspecified primary cancer (nos)
Tumor Type

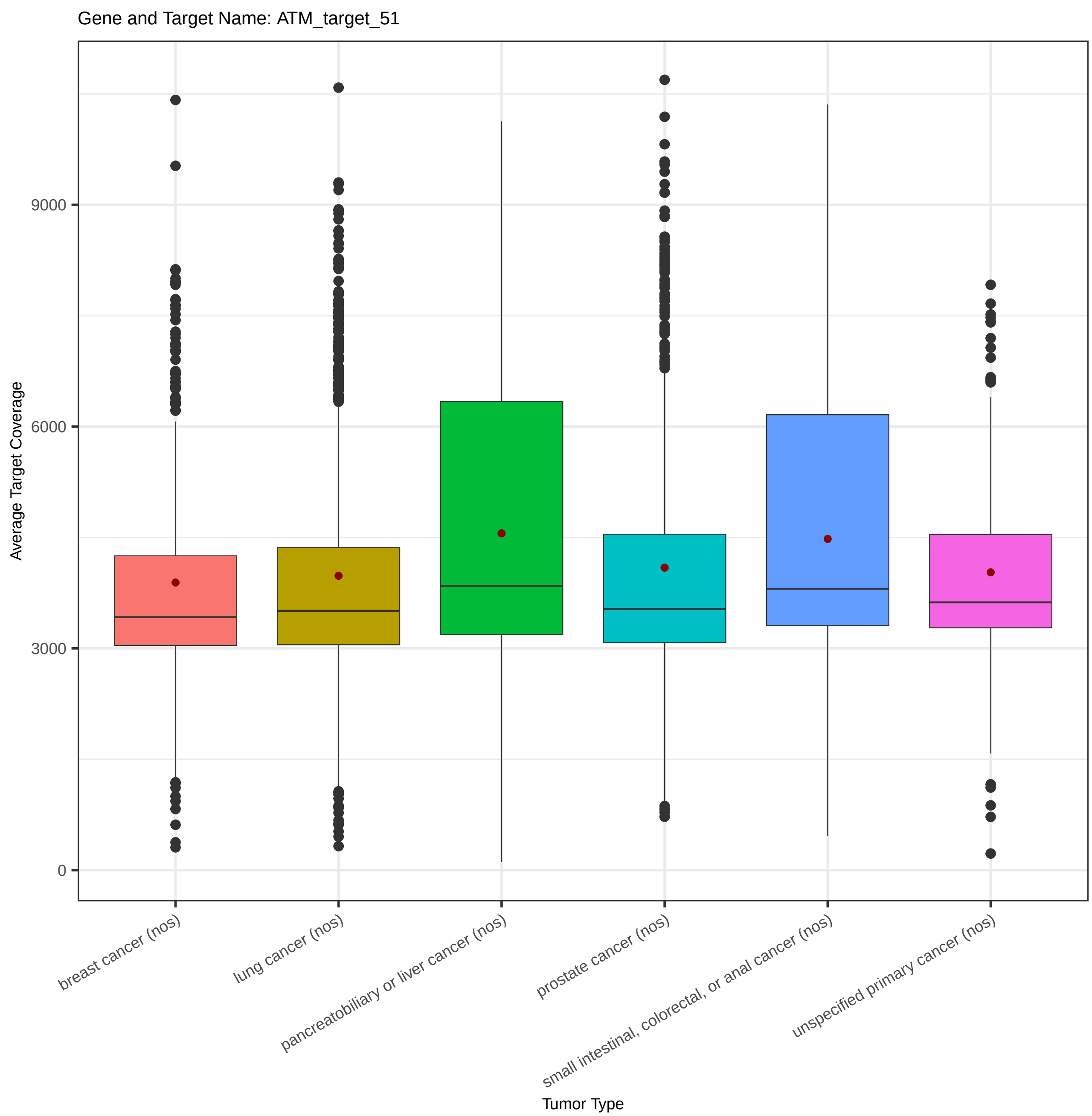

Gene and Target Name: ATM_target_51
Average Target Coverage
9000
6000
3000
0
breast cancer (nos)
lung cancer (nos)
pancreatobiliary or liver cancer (nos)
prostate cancer (nos)
small intestinal, colorectal, or anal cancer (nos)
unspecified primary cancer (nos)
Tumor Type

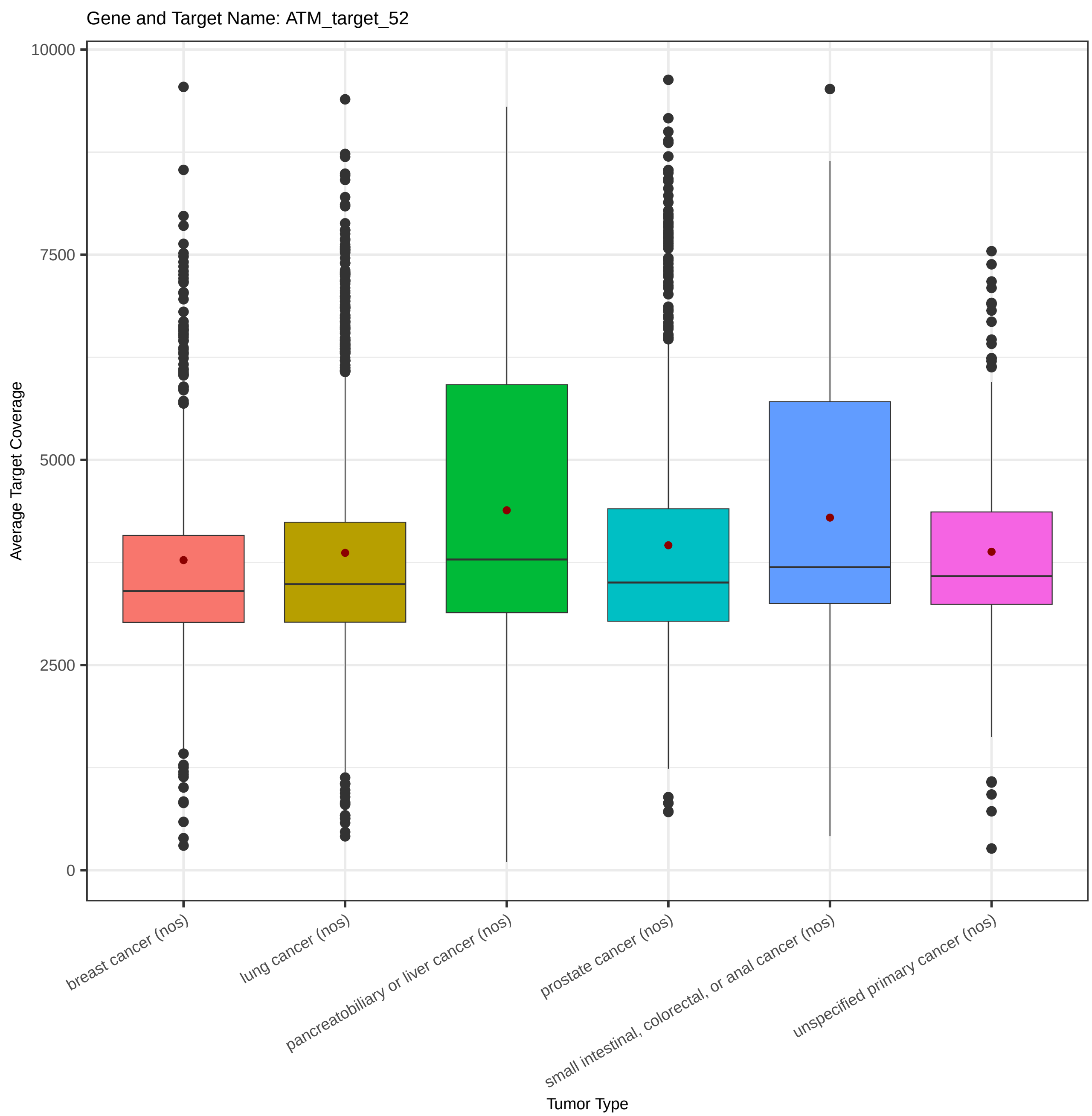
Gene and Target Name: ATM_target_52
Average Target Coverage
10000
7500
5000
2500
0
breast cancer (nos)
lung cancer (nos)
pancreatobiliary or liver cancer (nos)
prostate cancer (nos)
small intestinal, colorectal, or anal cancer (nos)
unspecified primary cancer (nos)
Tumor Type

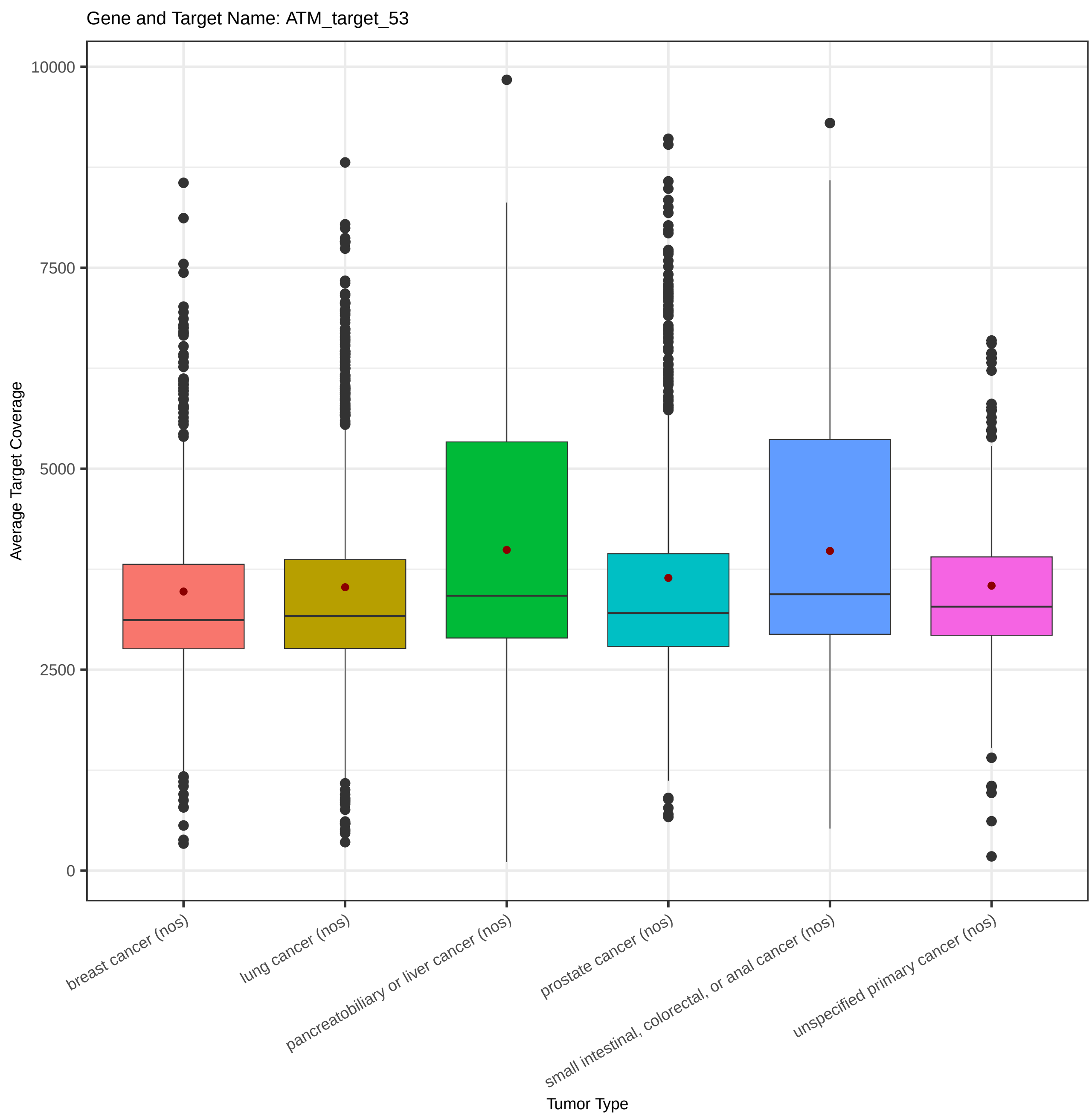

Gene and Target Name: ATM_target_53
10000
7500
5000
2500
0
Average Target Coverage
breast cancer (nos)
lung cancer (nos)
pancreatobiliary or liver cancer (nos)
prostate cancer (nos)
small intestinal, colorectal, or anal cancer (nos)
unspecified primary cancer (nos)
Tumor Type

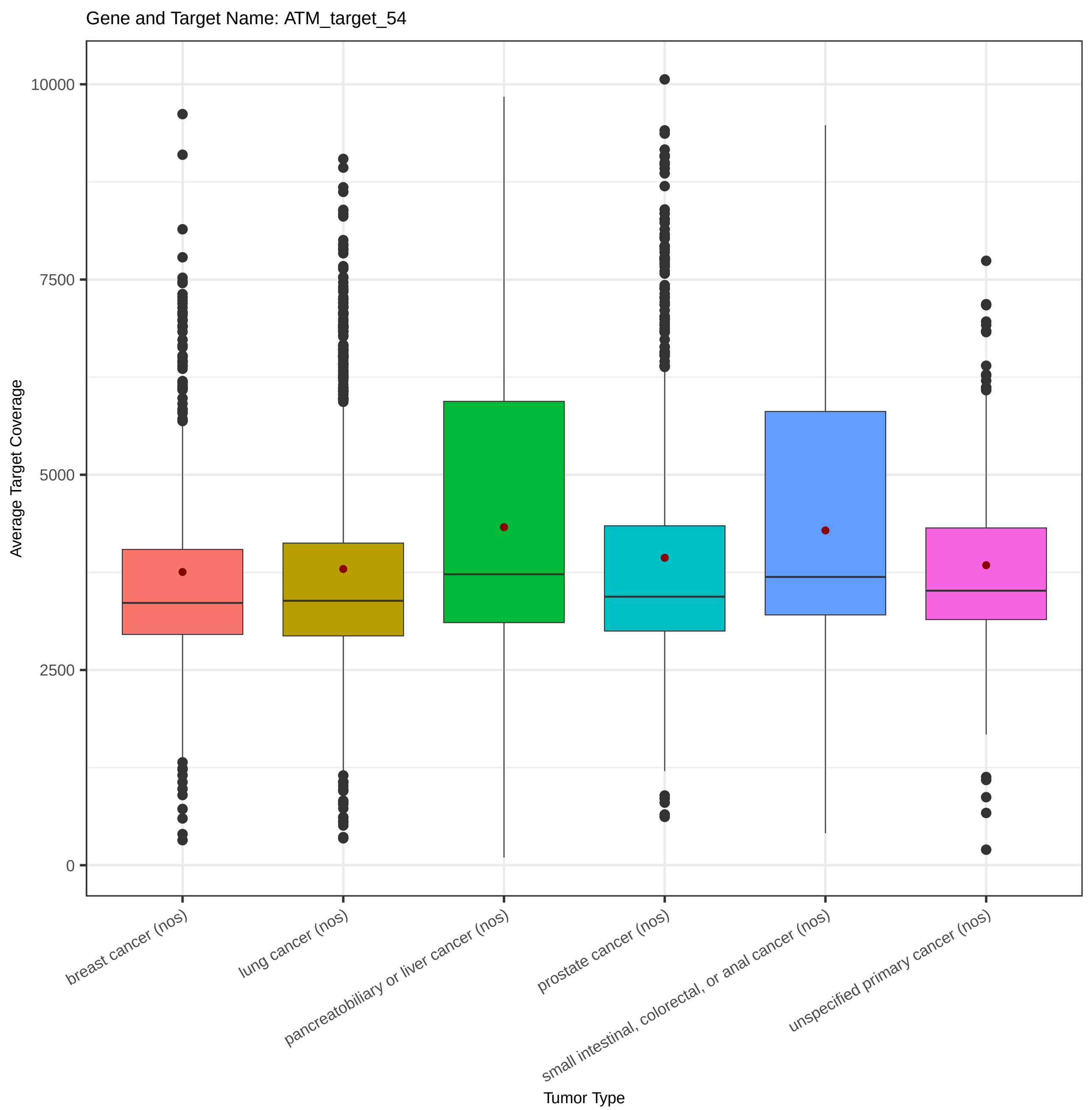

Gene and Target Name: ATM_target_54
Average Target Coverage
10000
7500
5000
2500
0
breast cancer (nos)
lung cancer (nos)
pancreatobiliary or liver cancer (nos)
prostate cancer (nos)
small intestinal, colorectal, or anal cancer (nos)
unspecified primary cancer (nos)
Tumor Type

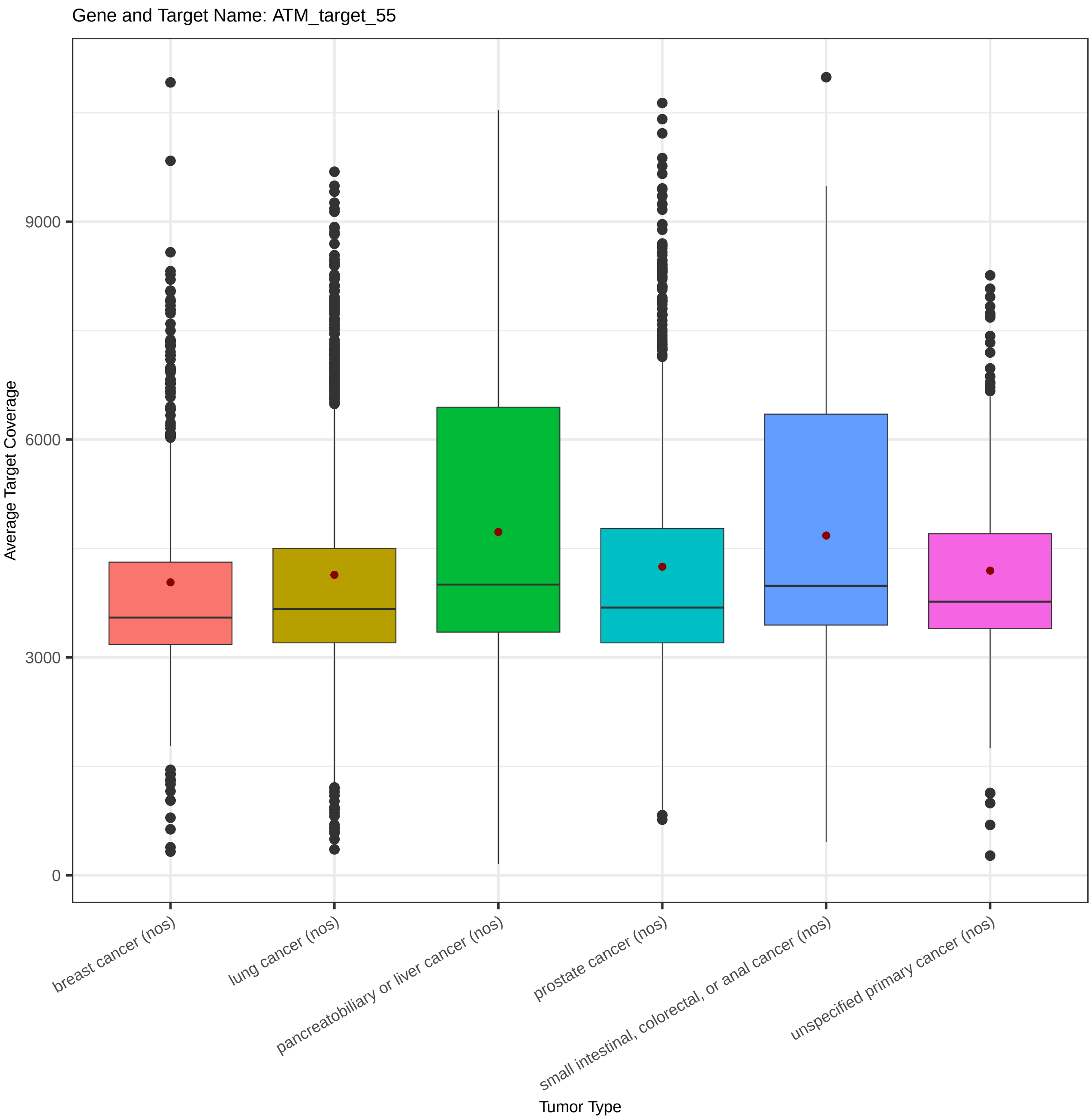

Gene and Target Name: ATM_target_55
Average Target Coverage
9000
6000
3000
0
breast cancer (nos)
lung cancer (nos)
pancreatobiliary or liver cancer (nos)
prostate cancer (nos)
small intestinal, colorectal, or anal cancer (nos)
unspecified primary cancer (nos)
Tumor Type

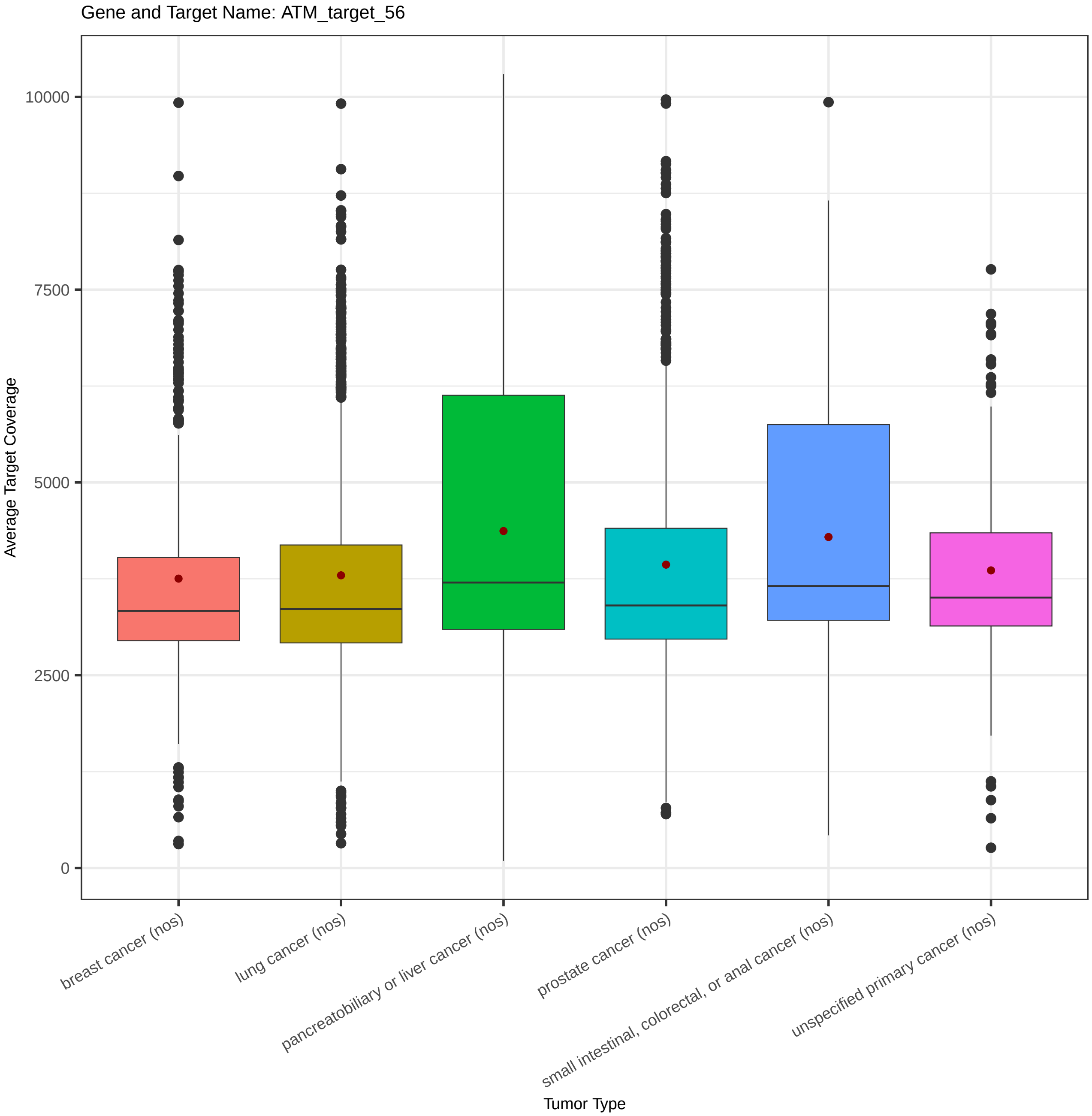

Gene and Target Name: ATM_target_56
Average Target Coverage
10000
7500
5000
2500
0
breast cancer (nos)
lung cancer (nos)
pancreatobiliary or liver cancer (nos)
prostate cancer (nos)
small intestinal, colorectal, or anal cancer (nos)
unspecified primary cancer (nos)
Tumor Type

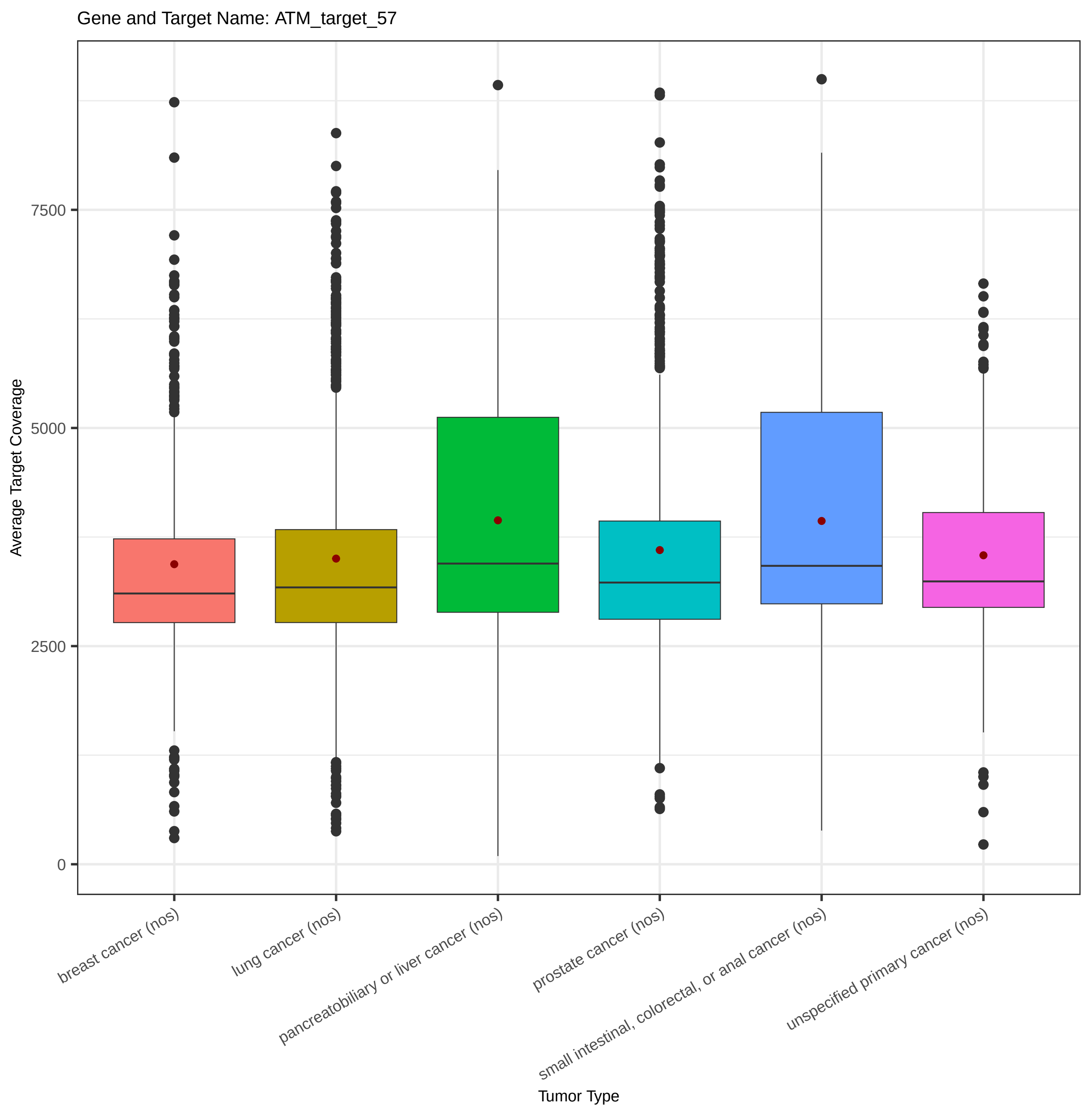

Gene and Target Name: ATM_target_57
Average Target Coverage
0
2500
5000
7500
breast cancer (nos)
lung cancer (nos)
pancreatobiliary or liver cancer (nos)
prostate cancer (nos)
small intestinal, colorectal, or anal cancer (nos)
unspecified primary cancer (nos)
Tumor Type

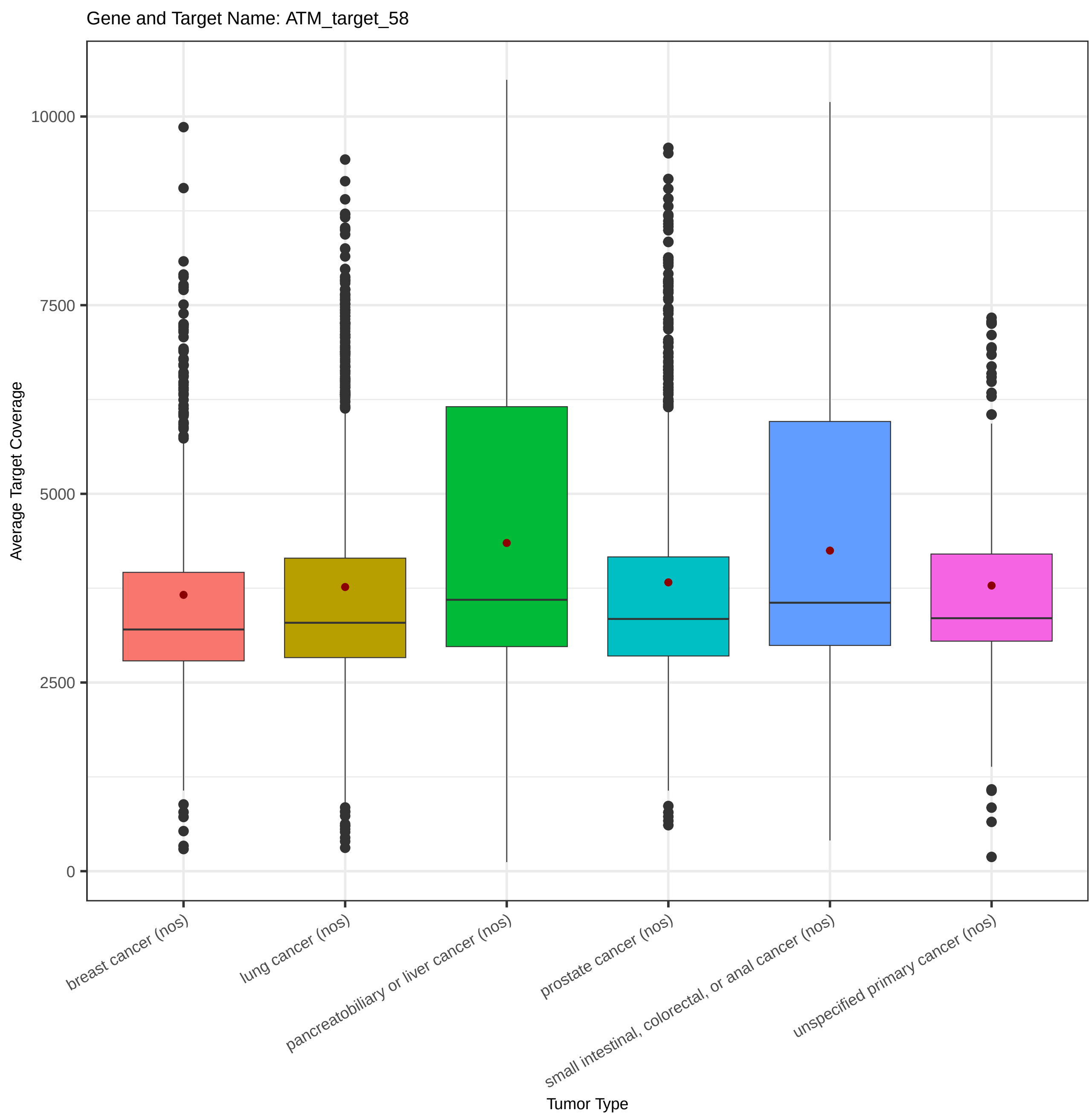
Gene and Target Name: ATM_target_58
Average Target Coverage
10000
7500
5000
2500
0
breast cancer (nos)
lung cancer (nos)
pancreatobiliary or liver cancer (nos)
prostate cancer (nos)
small intestinal, colorectal, or anal cancer (nos)
unspecified primary cancer (nos)
Tumor Type

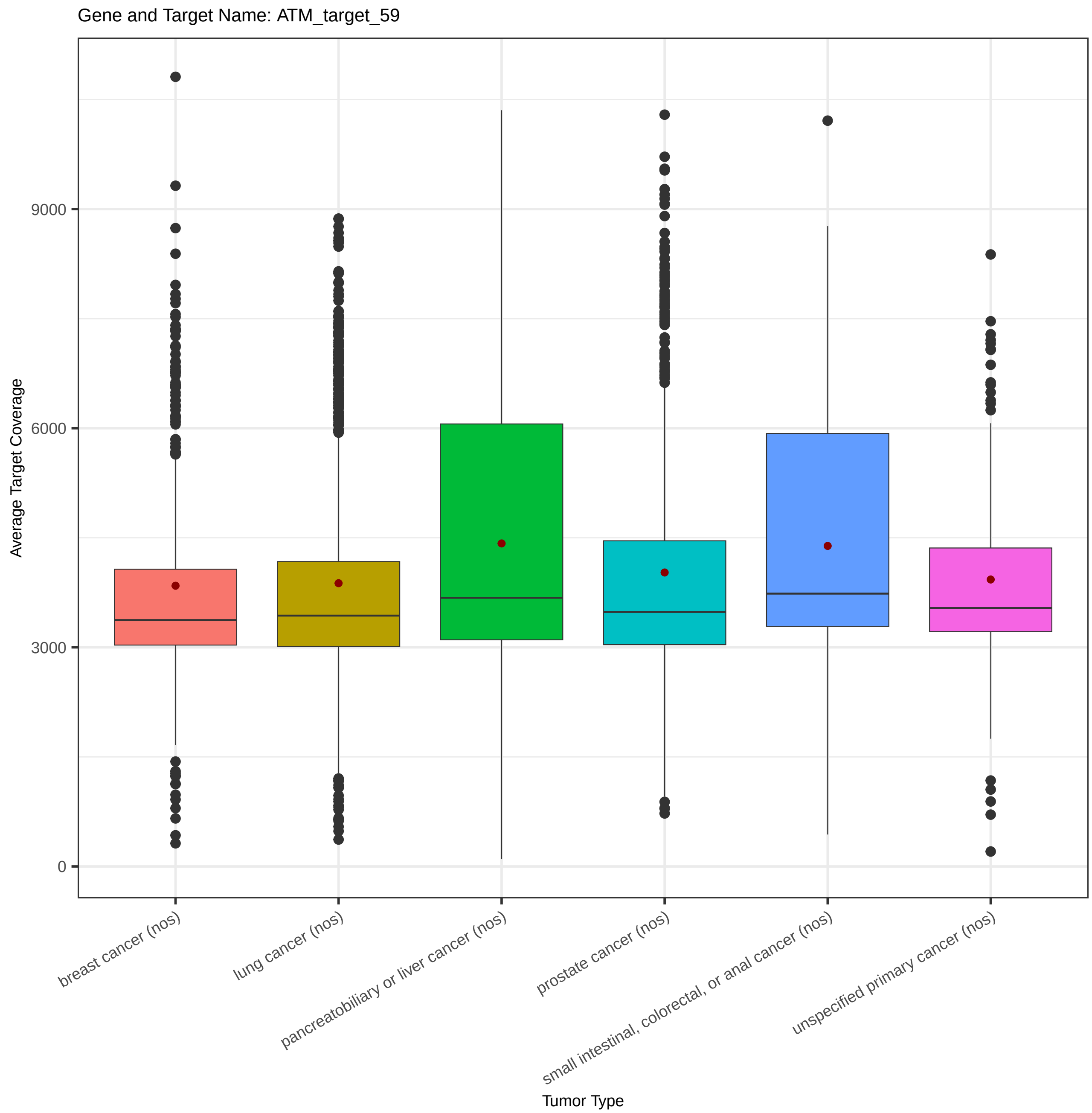

Gene and Target Name: ATM_target_59
Average Target Coverage
9000
6000
3000
0
breast cancer (nos)
lung cancer (nos)
pancreatobiliary or liver cancer (nos)
prostate cancer (nos)
small intestinal, colorectal, or anal cancer (nos)
unspecified primary cancer (nos)
Tumor Type

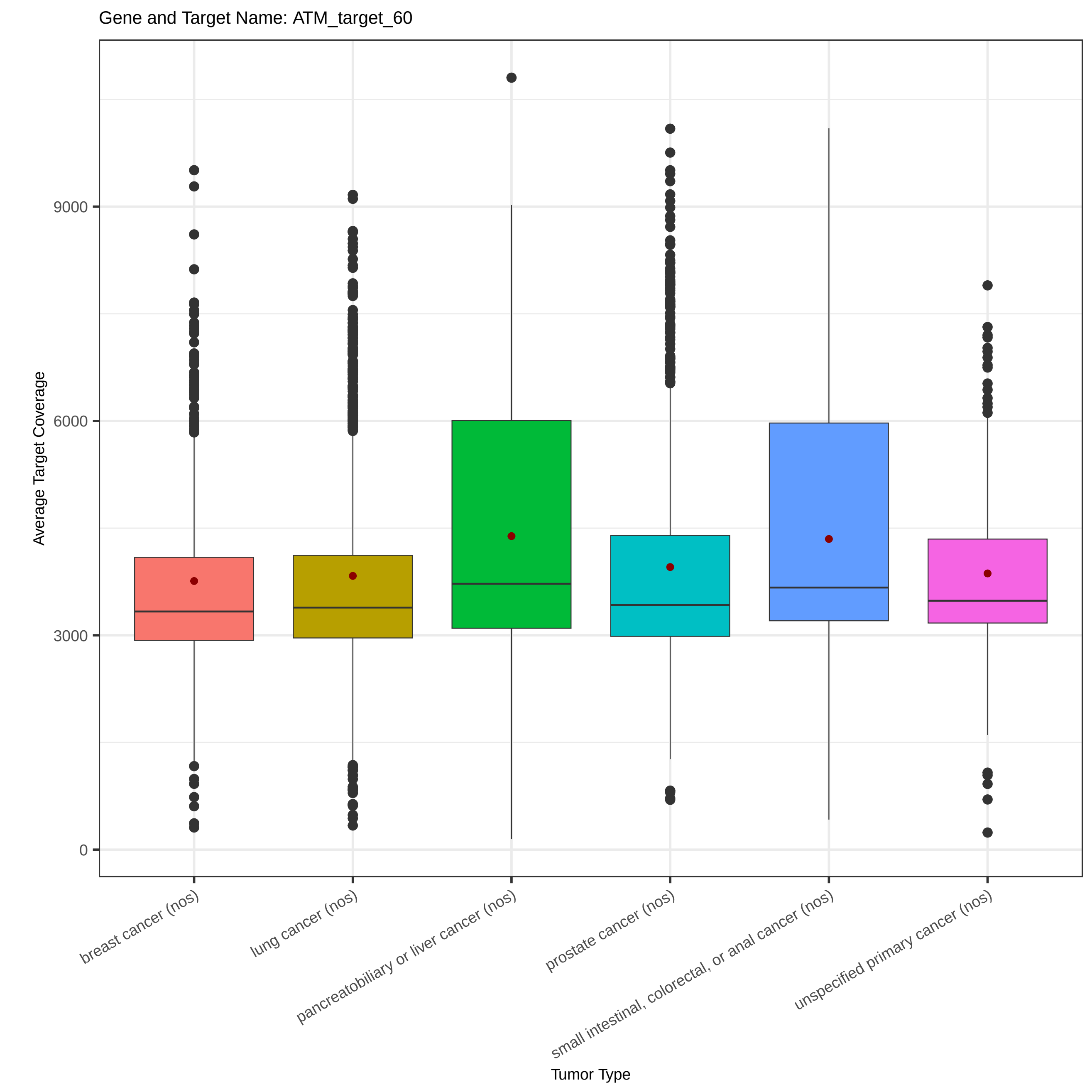

Gene and Target Name: ATM_target_60
Average Target Coverage
9000
6000
3000
0
breast cancer (nos)
lung cancer (nos)
pancreatobiliary or liver cancer (nos)
prostate cancer (nos)
small intestinal, colorectal, or anal cancer (nos)
unspecified primary cancer (nos)
Tumor Type

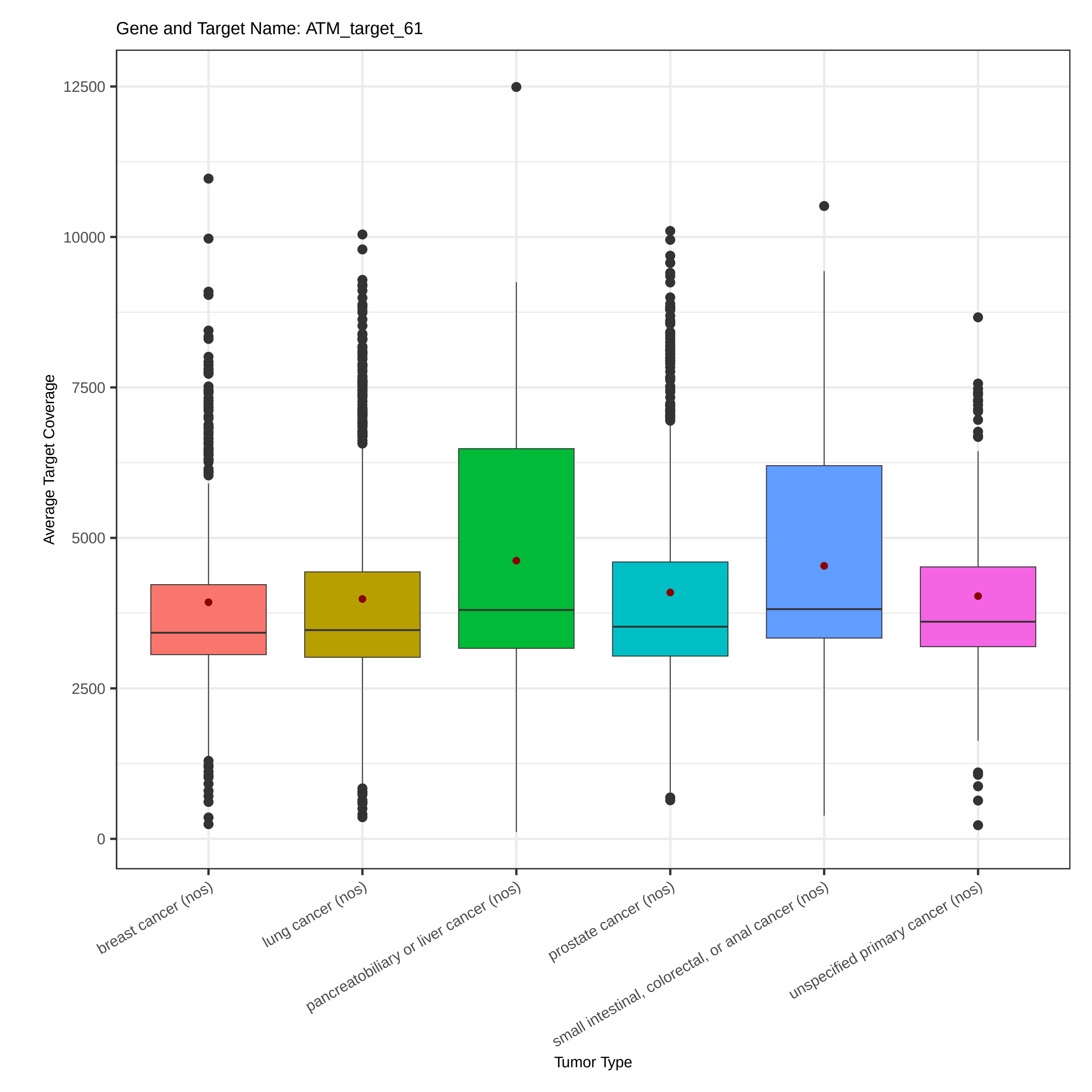

Gene and Target Name: ATM_target_61
Average Target Coverage
12500
10000
7500
5000
2500
0
breast cancer (nos)
lung cancer (nos)
pancreatobiliary or liver cancer (nos)
prostate cancer (nos)
small intestinal, colorectal, or anal cancer (nos)
unspecified primary cancer (nos)
Tumor Type

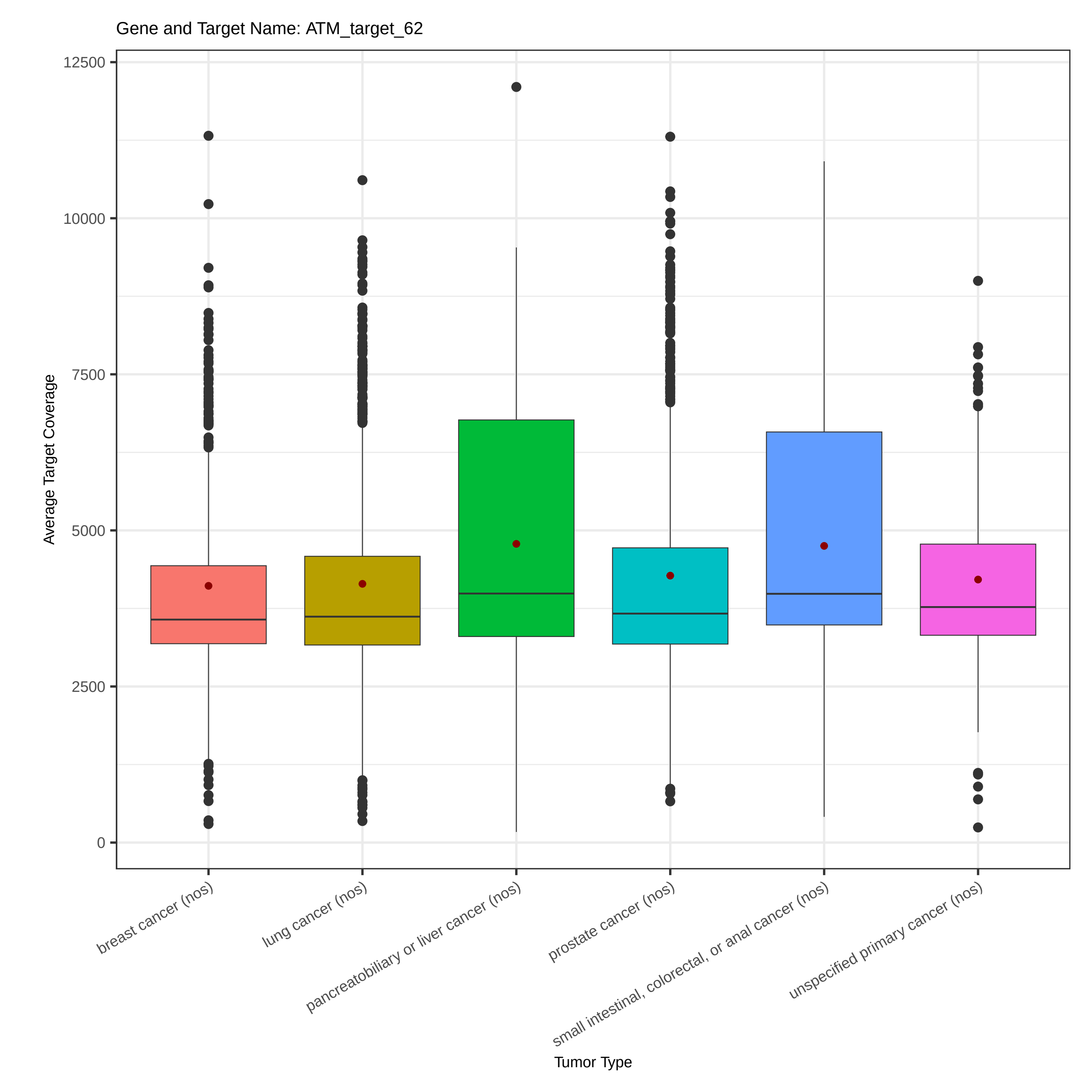

Gene and Target Name: ATM_target_62
12500
10000
7500
5000
2500
0
Average Target Coverage
breast cancer (nos)
lung cancer (nos)
pancreatobiliary or liver cancer (nos)
prostate cancer (nos)
small intestinal, colorectal, or anal cancer (nos)
unspecified primary cancer (nos)
Tumor Type

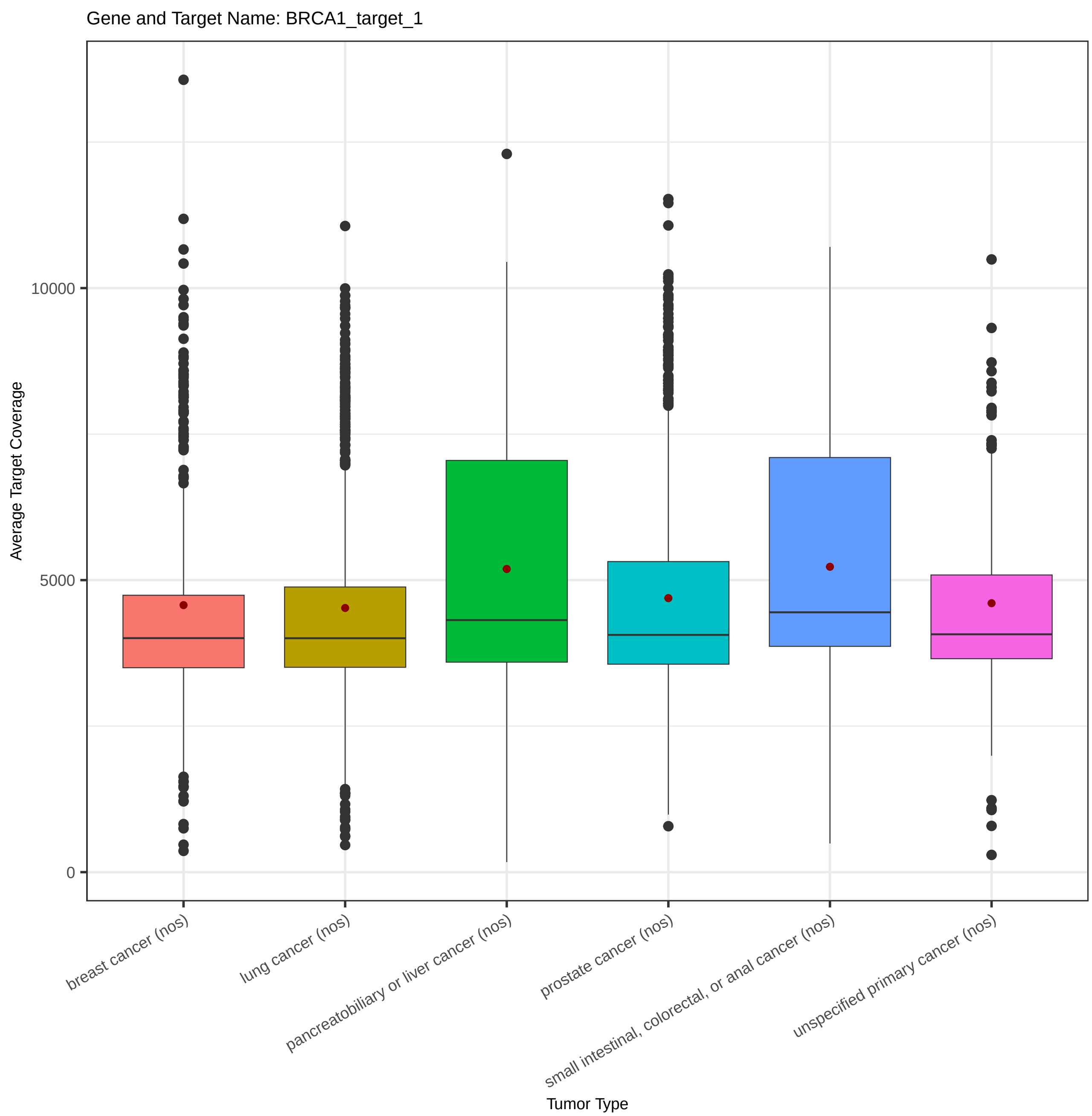

Gene and Target Name: BRCA1_target_1
Average Target Coverage
10000
5000
0
breast cancer (nos)
lung cancer (nos)
pancreatobiliary or liver cancer (nos)
prostate cancer (nos)
small intestinal, colorectal, or anal cancer (nos)
unspecified primary cancer (nos)
Tumor Type

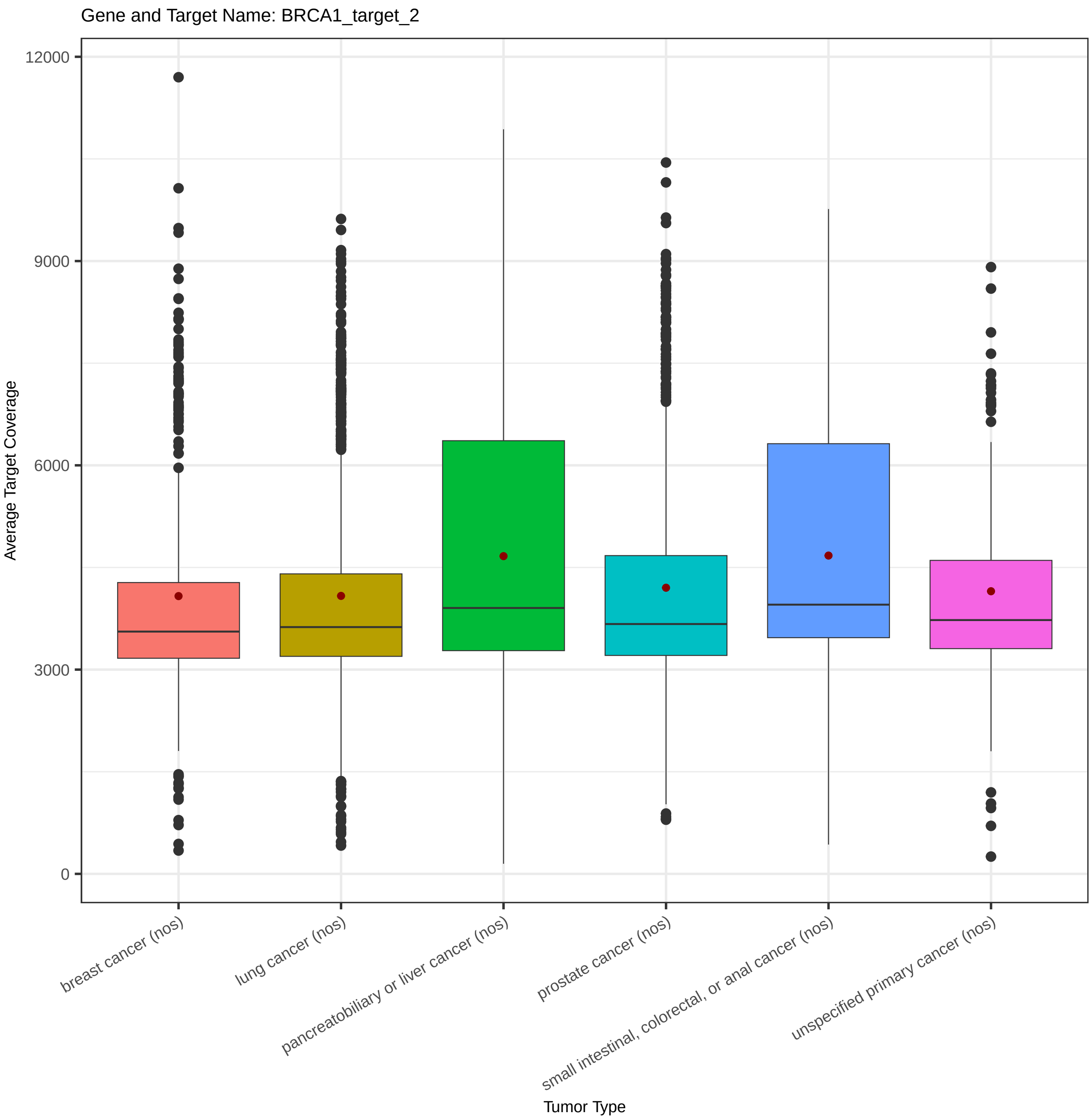
Gene and Target Name: BRCA1_target_2
Average Target Coverage
12000
9000
6000
3000
0
breast cancer (nos)
lung cancer (nos)
pancreatobiliary or liver cancer (nos)
prostate cancer (nos)
small intestinal, colorectal, or anal cancer (nos)
unspecified primary cancer (nos)
Tumor Type

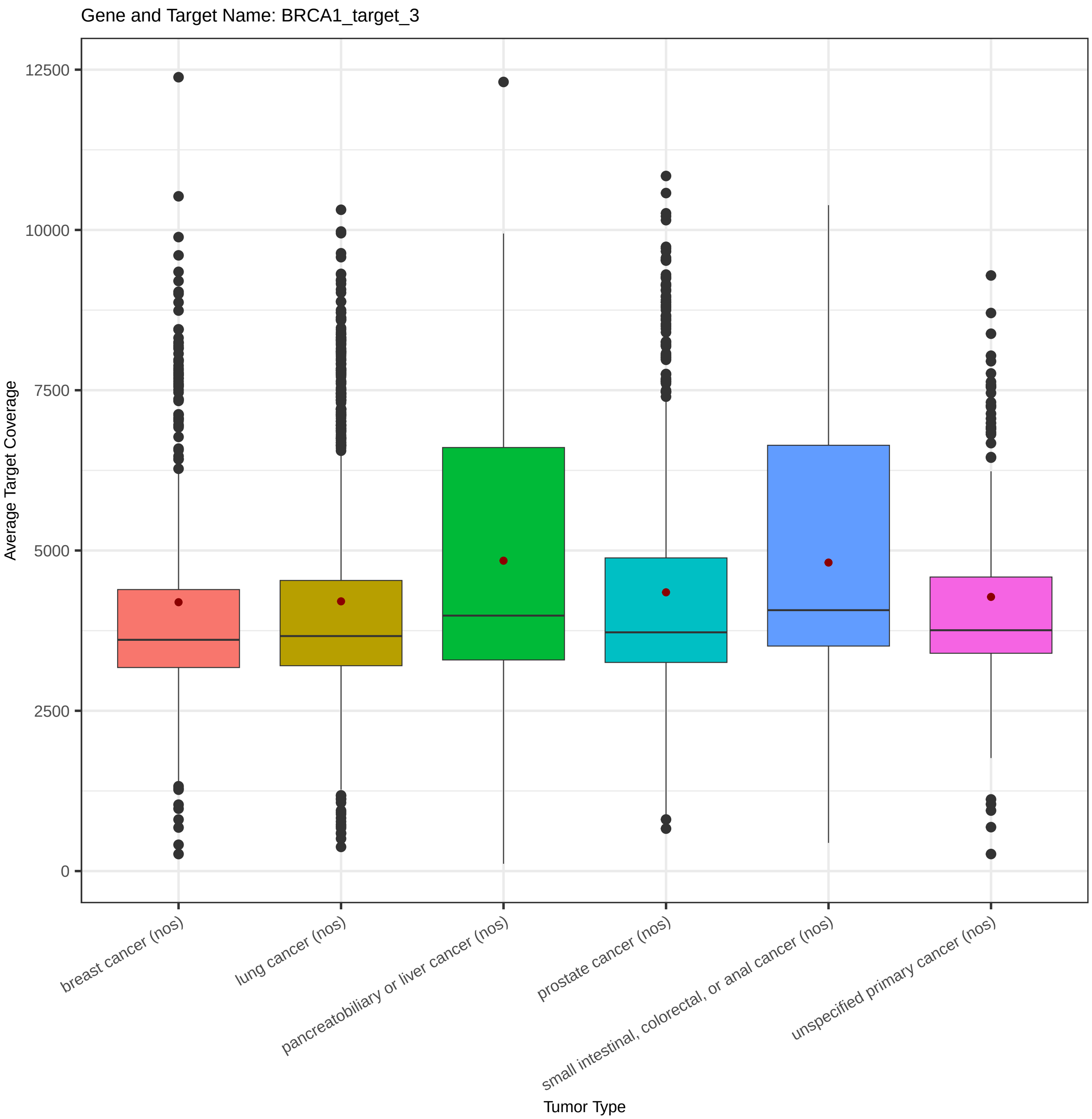

Gene and Target Name: BRCA1_target_3
Average Target Coverage
12500
10000
7500
5000
2500
0
breast cancer (nos)
lung cancer (nos)
pancreatobiliary or liver cancer (nos)
prostate cancer (nos)
small intestinal, colorectal, or anal cancer (nos)
unspecified primary cancer (nos)
Tumor Type

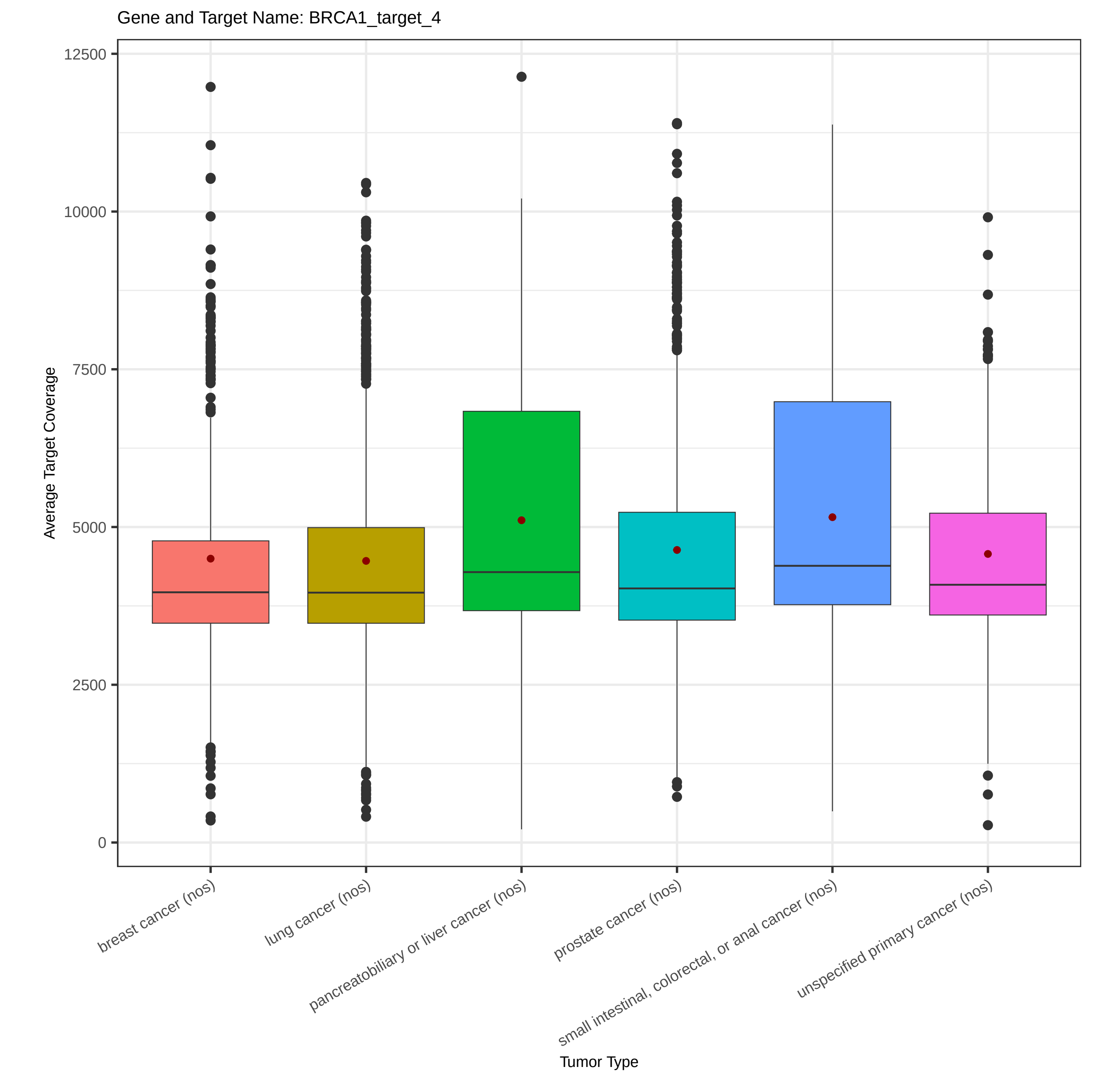

Gene and Target Name: BRCA1_target_4
Average Target Coverage
12500
10000
7500
5000
2500
0
breast cancer (nos)
lung cancer (nos)
pancreatobiliary or liver cancer (nos)
prostate cancer (nos)
small intestinal, colorectal, or anal cancer (nos)
unspecified primary cancer (nos)
Tumor Type

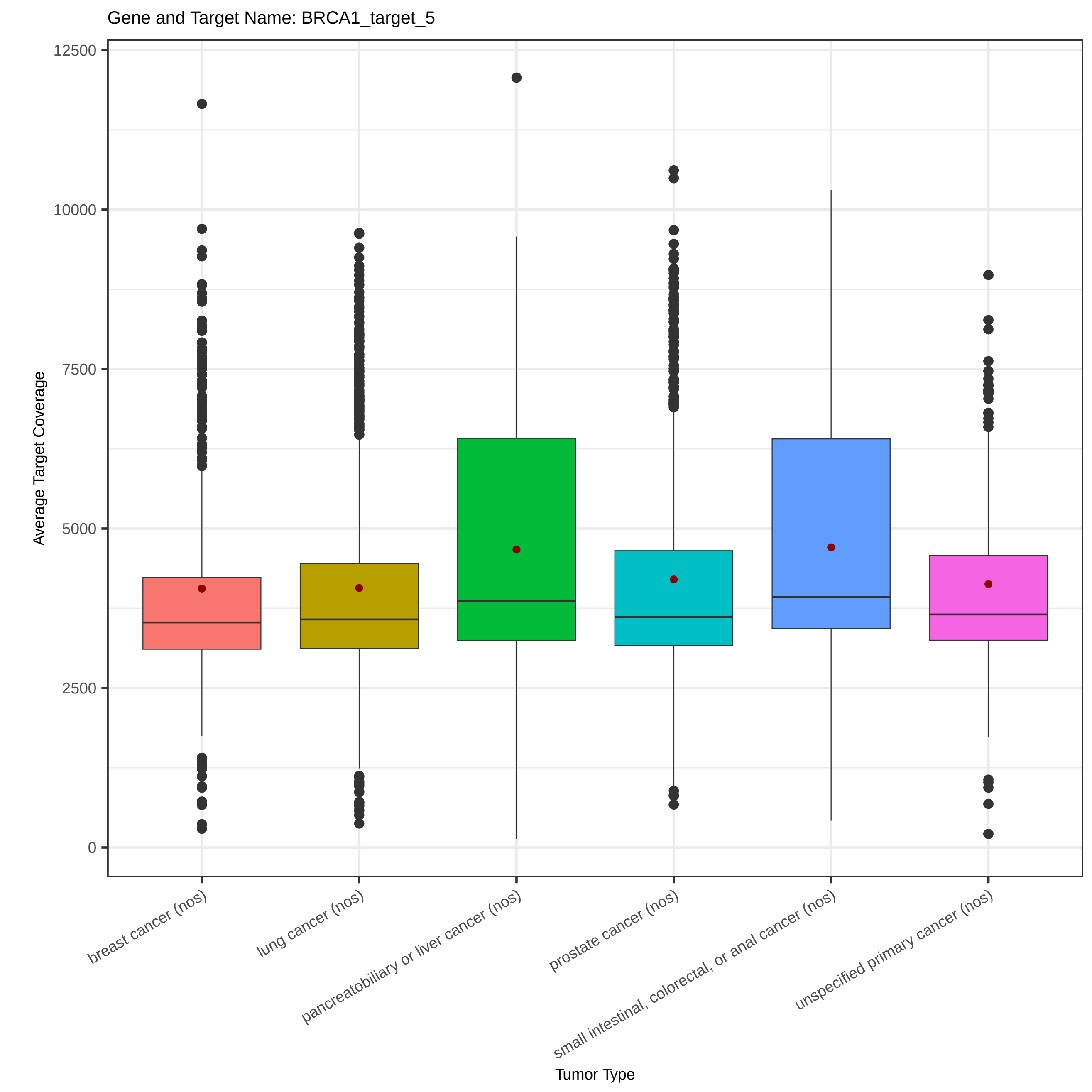
Gene and Target Name: BRCA1_target_5
Average Target Coverage
12500
10000
7500
5000
2500
0
breast cancer (nos)
lung cancer (nos)
pancreatobiliary or liver cancer (nos)
prostate cancer (nos)
small intestinal, colorectal, or anal cancer (nos)
unspecified primary cancer (nos)
Tumor Type

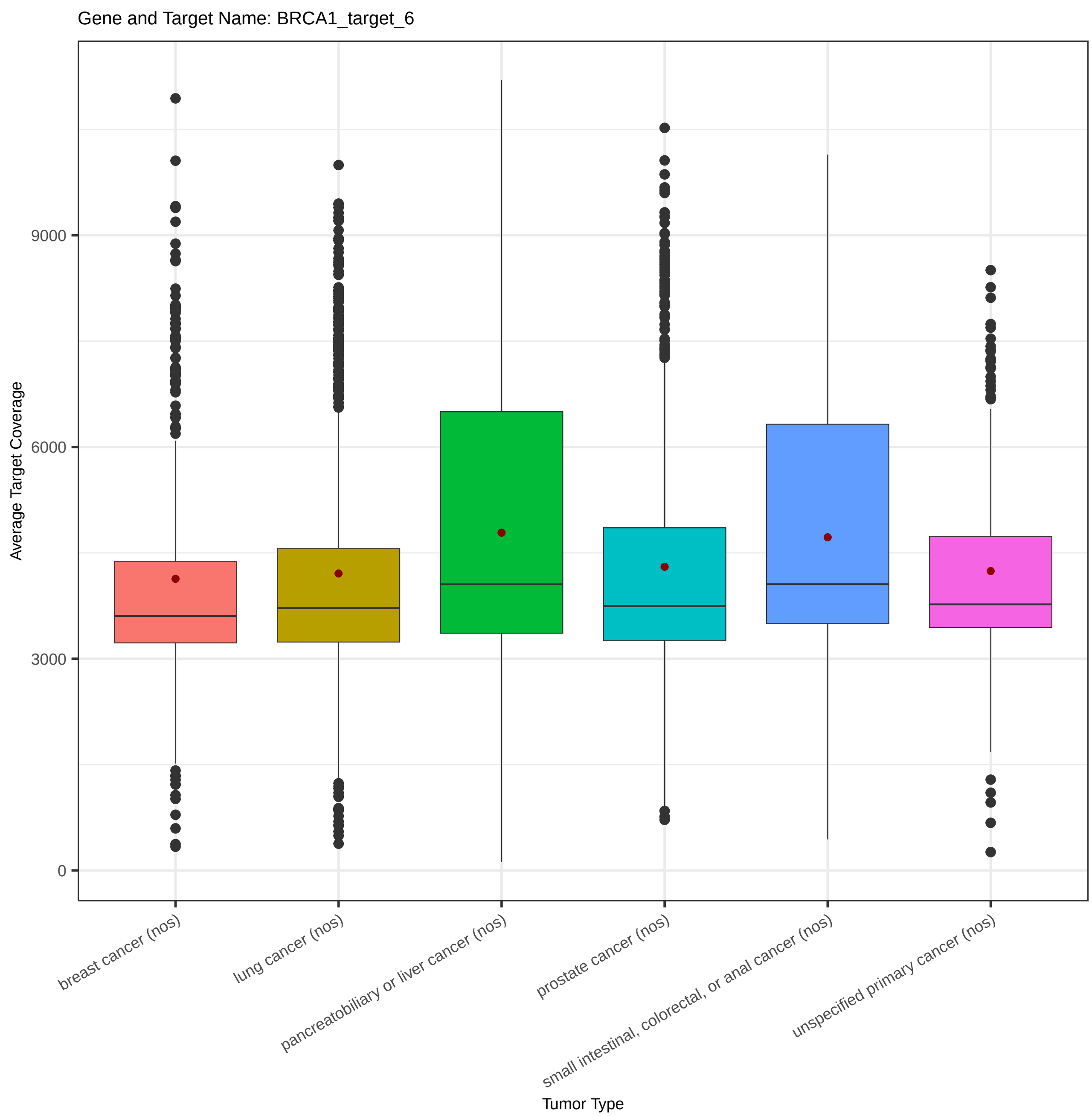

Gene and Target Name: BRCA1_target_6
Average Target Coverage
9000
6000
3000
0
breast cancer (nos)
lung cancer (nos)
pancreatobiliary or liver cancer (nos)
prostate cancer (nos)
small intestinal, colorectal, or anal cancer (nos)
unspecified primary cancer (nos)
Tumor Type

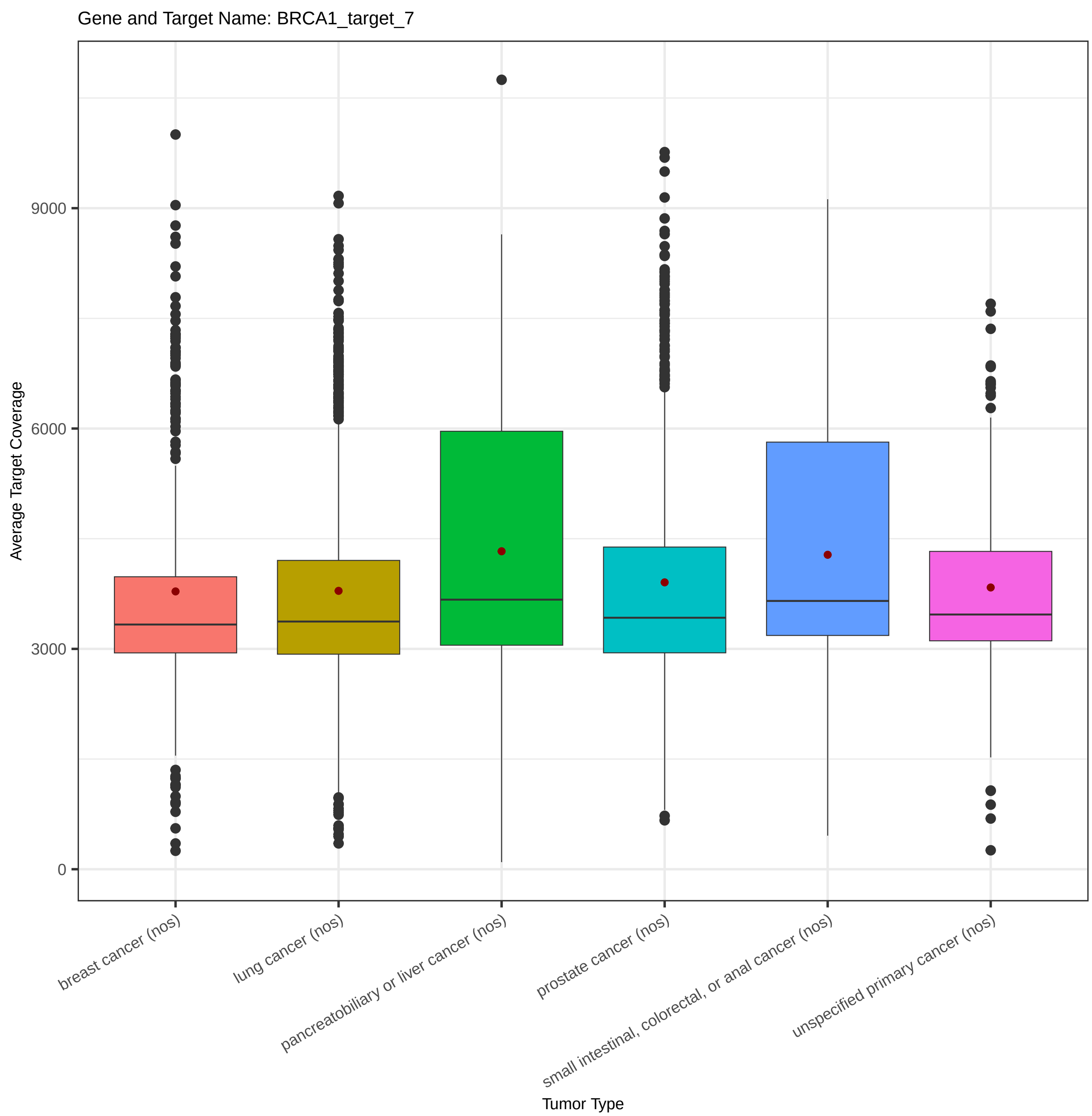

Gene and Target Name: BRCA1_target_7
Average Target Coverage
9000
6000
3000
0
breast cancer (nos)
lung cancer (nos)
pancreatobiliary or liver cancer (nos)
prostate cancer (nos)
small intestinal, colorectal, or anal cancer (nos)
unspecified primary cancer (nos)
Tumor Type

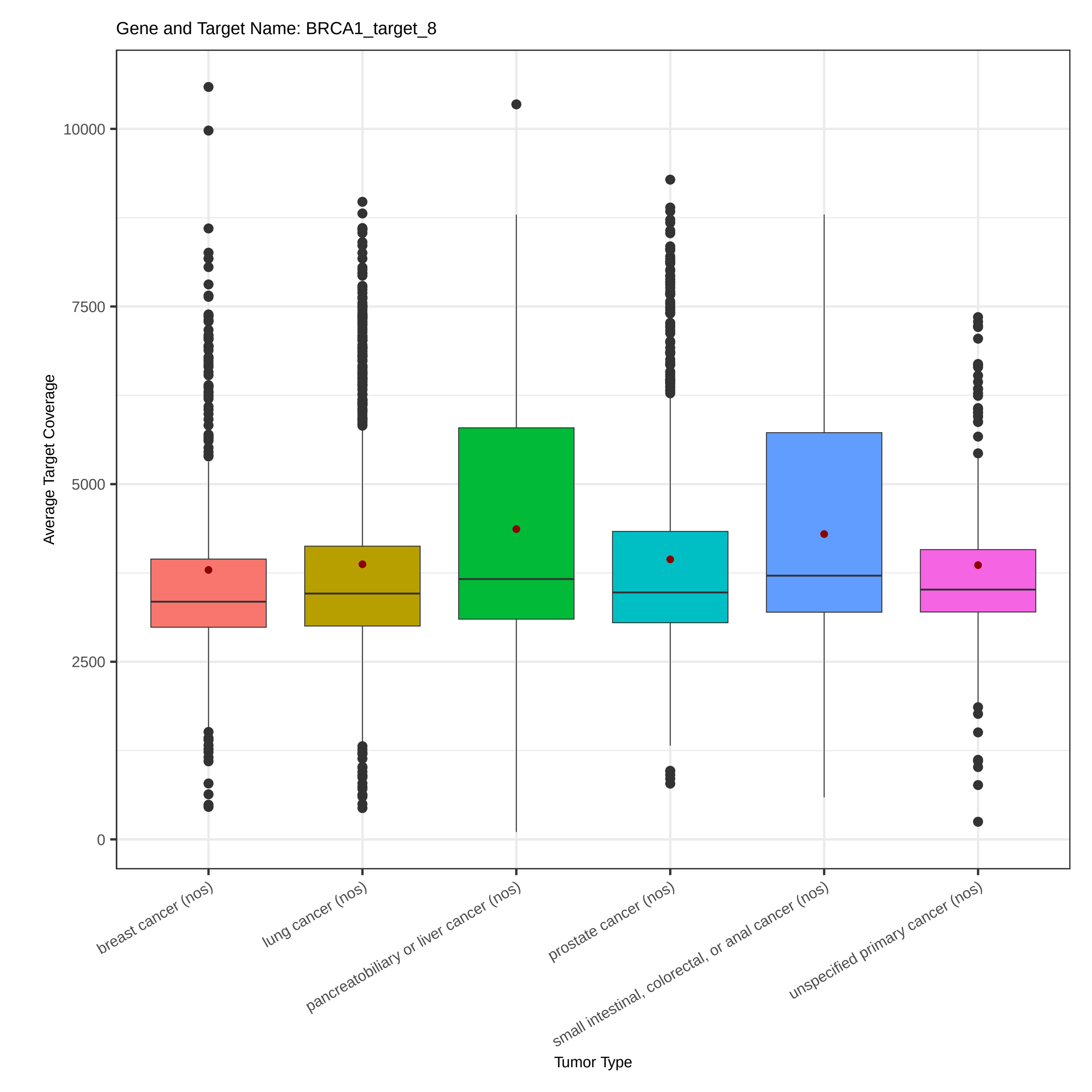

Gene and Target Name: BRCA1_target_8
Average Target Coverage
10000
7500
5000
2500
0
breast cancer (nos)
lung cancer (nos)
pancreatobiliary or liver cancer (nos)
prostate cancer (nos)
small intestinal, colorectal, or anal cancer (nos)
unspecified primary cancer (nos)
Tumor Type

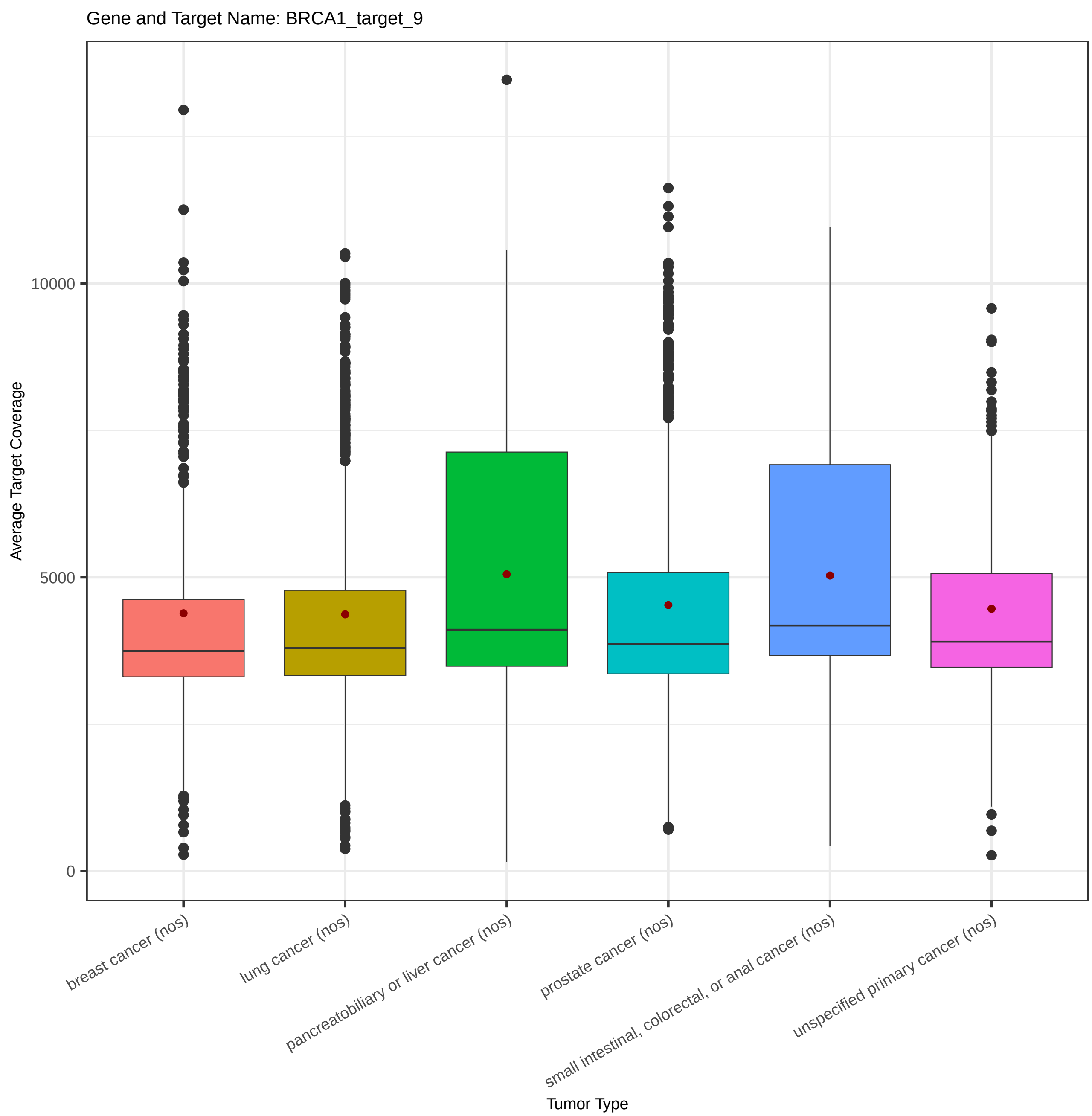

Gene and Target Name: BRCA1_target_9
Average Target Coverage
10000
5000
0
breast cancer (nos)
lung cancer (nos)
pancreatobiliary or liver cancer (nos)
prostate cancer (nos)
small intestinal, colorectal, or anal cancer (nos)
unspecified primary cancer (nos)
Tumor Type

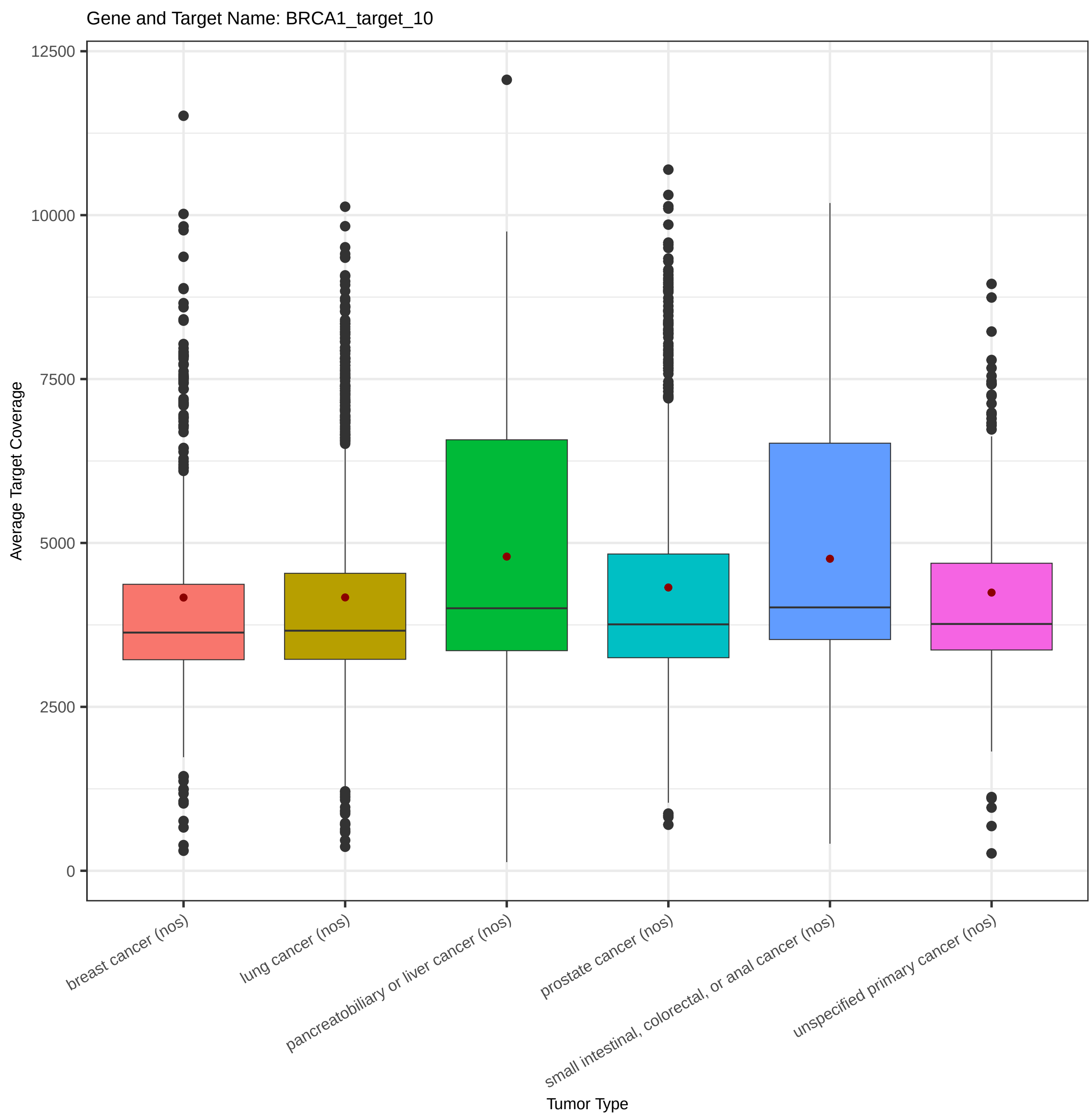
Gene and Target Name: BRCA1_target_10
Average Target Coverage
12500
10000
7500
5000
2500
0
breast cancer (nos)
lung cancer (nos)
pancreatobiliary or liver cancer (nos)
prostate cancer (nos)
small intestinal, colorectal, or anal cancer (nos)
unspecified primary cancer (nos)
Tumor Type

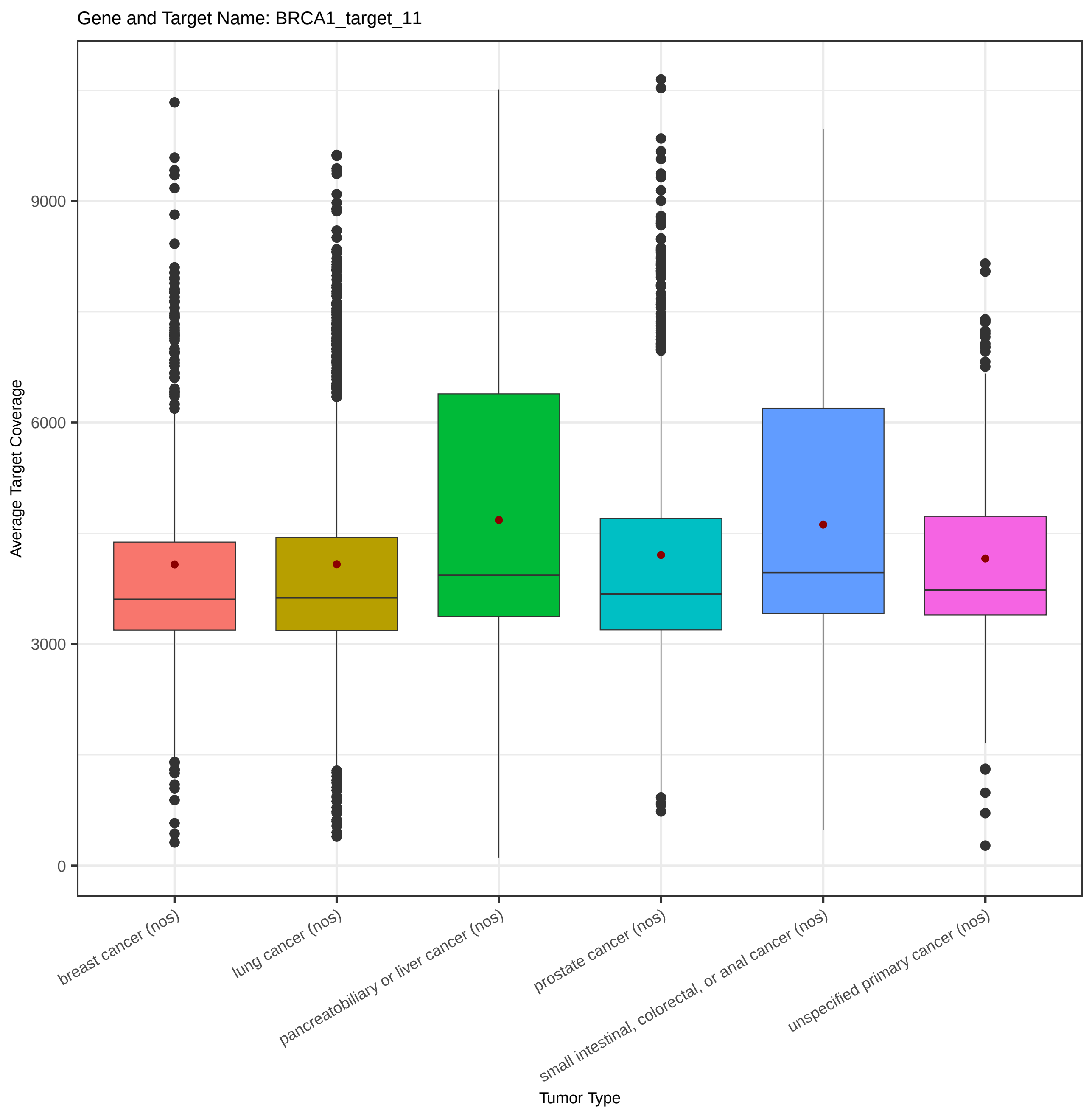

Gene and Target Name: BRCA1_target_11
Average Target Coverage
9000
6000
3000
0
breast cancer (nos)
lung cancer (nos)
pancreatobiliary or liver cancer (nos)
prostate cancer (nos)
small intestinal, colorectal, or anal cancer (nos)
unspecified primary cancer (nos)
Tumor Type

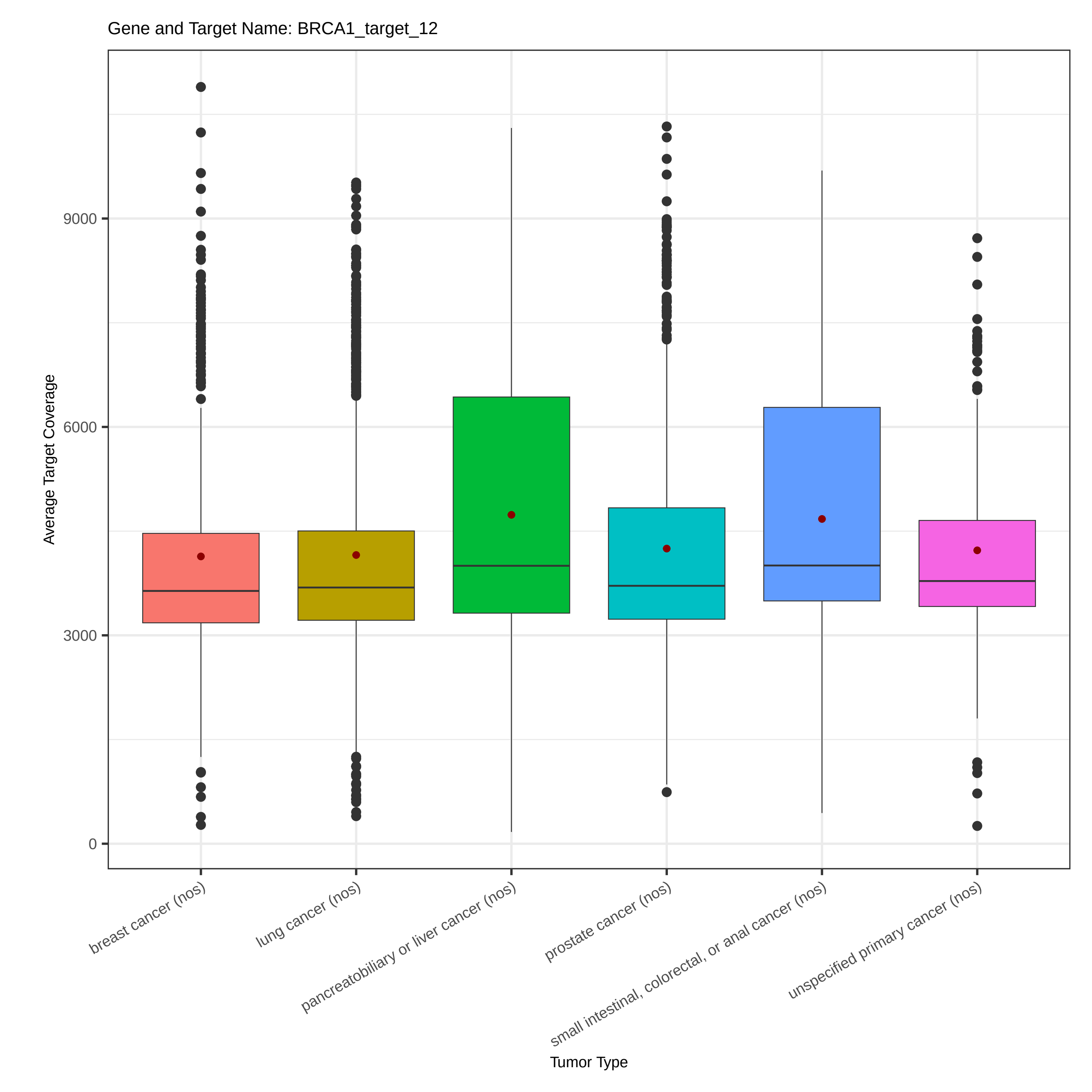
Gene and Target Name: BRCA1_target_12
Average Target Coverage
0
3000
6000
9000
breast cancer (nos)
lung cancer (nos)
pancreatobiliary or liver cancer (nos)
prostate cancer (nos)
small intestinal, colorectal, or anal cancer (nos)
unspecified primary cancer (nos)
Tumor Type

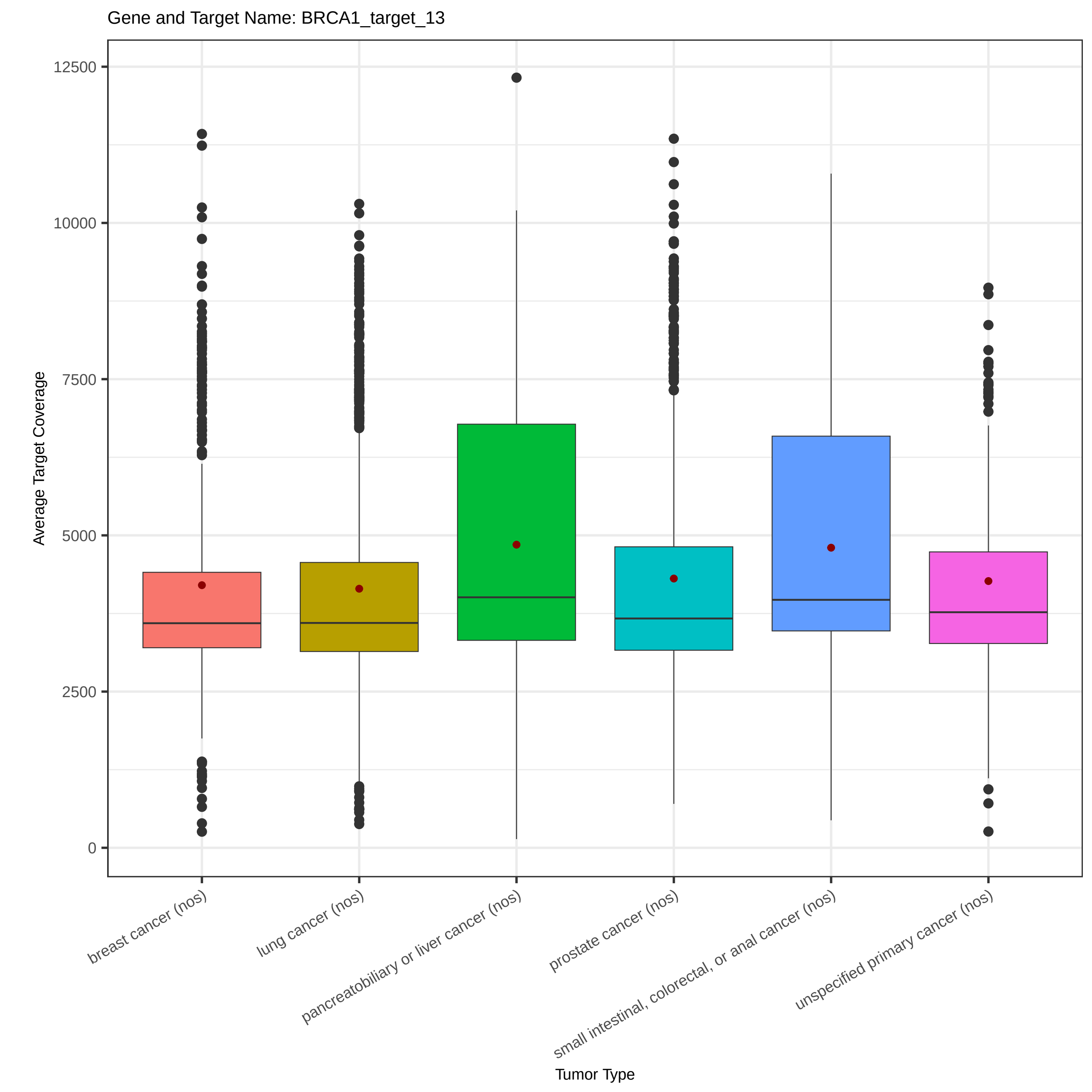

Gene and Target Name: BRCA1_target_13
Average Target Coverage
12500
10000
7500
5000
2500
0
breast cancer (nos)
lung cancer (nos)
pancreatobiliary or liver cancer (nos)
prostate cancer (nos)
small intestinal, colorectal, or anal cancer (nos)
unspecified primary cancer (nos)
Tumor Type

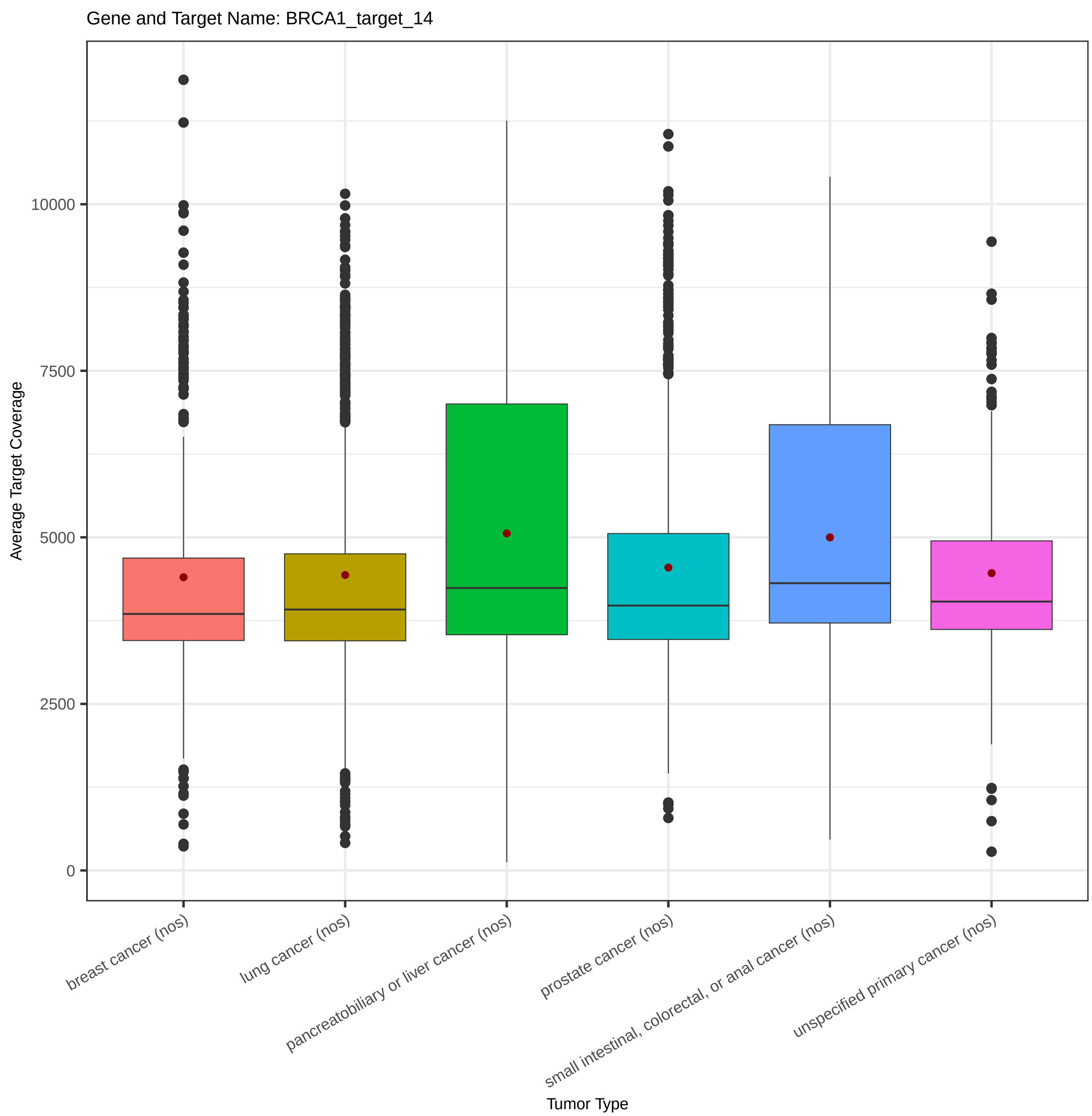

Gene and Target Name: BRCA1_target_14
Average Target Coverage
10000
7500
5000
2500
0
breast cancer (nos)
lung cancer (nos)
pancreatobiliary or liver cancer (nos)
prostate cancer (nos)
small intestinal, colorectal, or anal cancer (nos)
unspecified primary cancer (nos)
Tumor Type

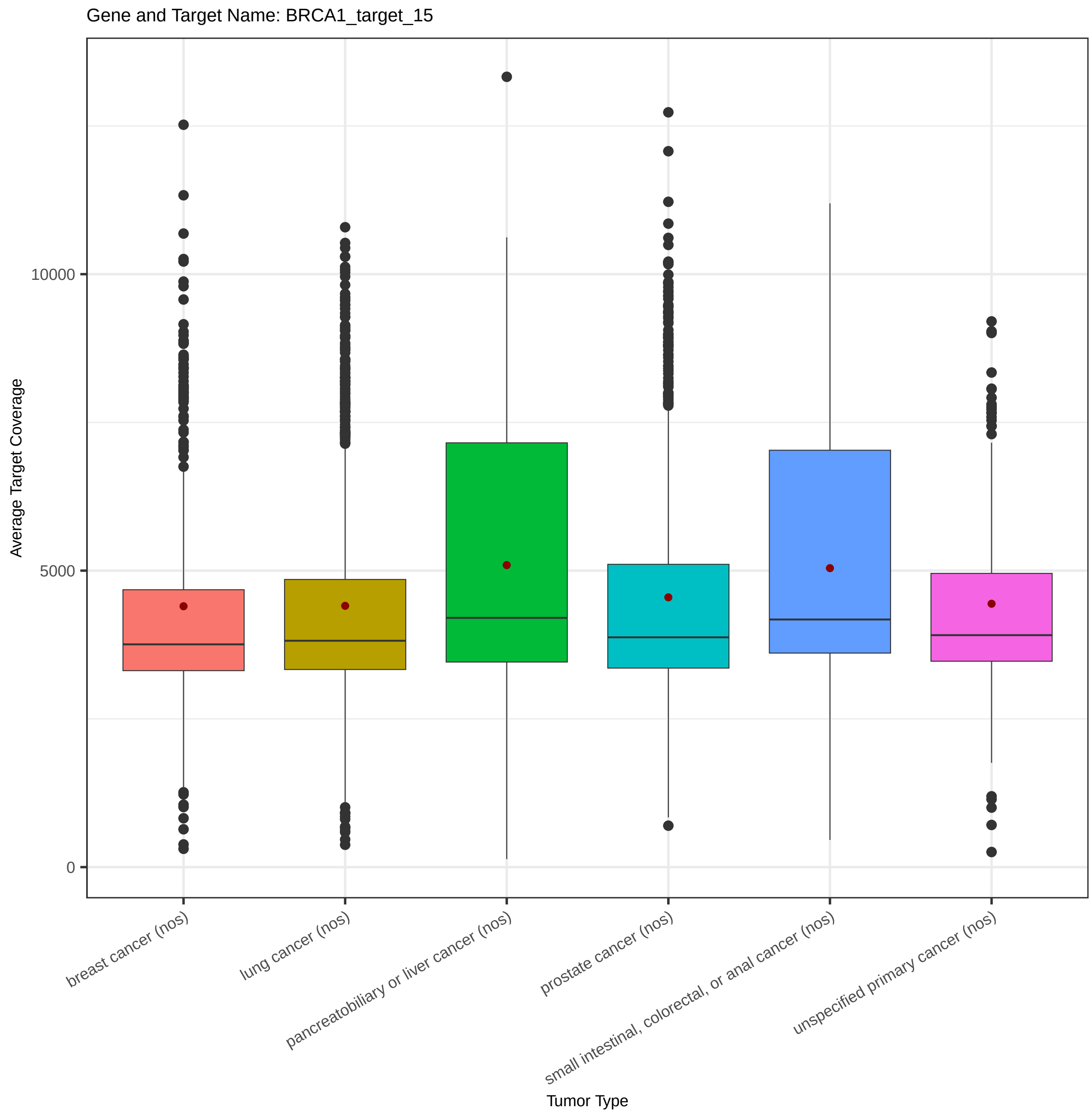

Gene and Target Name: BRCA1_target_15
Average Target Coverage
10000
5000
0
breast cancer (nos)
lung cancer (nos)
pancreatobiliary or liver cancer (nos)
prostate cancer (nos)
small intestinal, colorectal, or anal cancer (nos)
unspecified primary cancer (nos)
Tumor Type

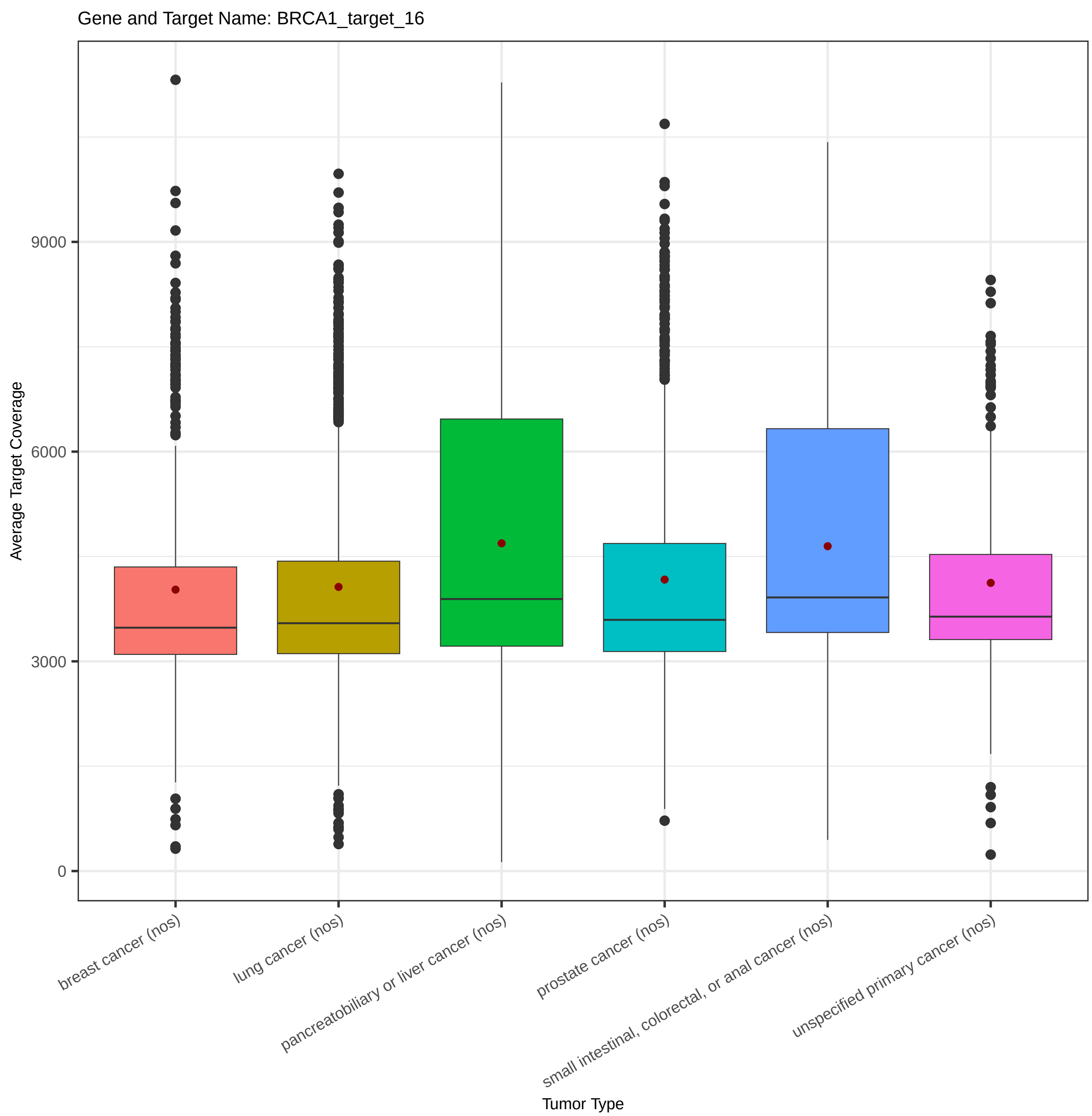

Gene and Target Name: BRCA1_target_16
Average Target Coverage
9000
6000
3000
0
breast cancer (nos)
lung cancer (nos)
pancreatobiliary or liver cancer (nos)
prostate cancer (nos)
small intestinal, colorectal, or anal cancer (nos)
unspecified primary cancer (nos)
Tumor Type

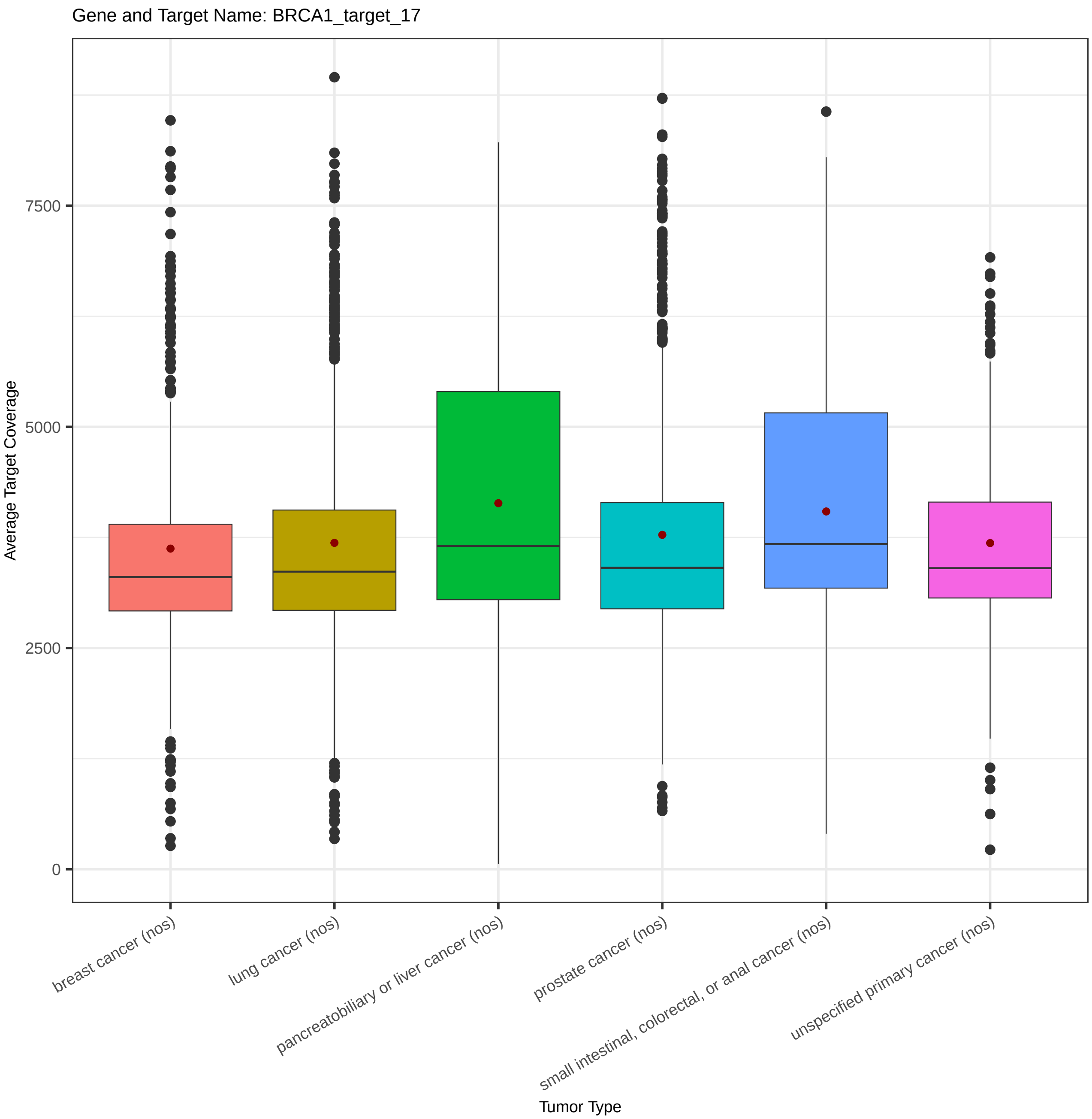

Gene and Target Name: BRCA1_target_17
Average Target Coverage
0
2500
5000
7500
breast cancer (nos)
lung cancer (nos)
pancreatobiliary or liver cancer (nos)
prostate cancer (nos)
small intestinal, colorectal, or anal cancer (nos)
unspecified primary cancer (nos)
Tumor Type

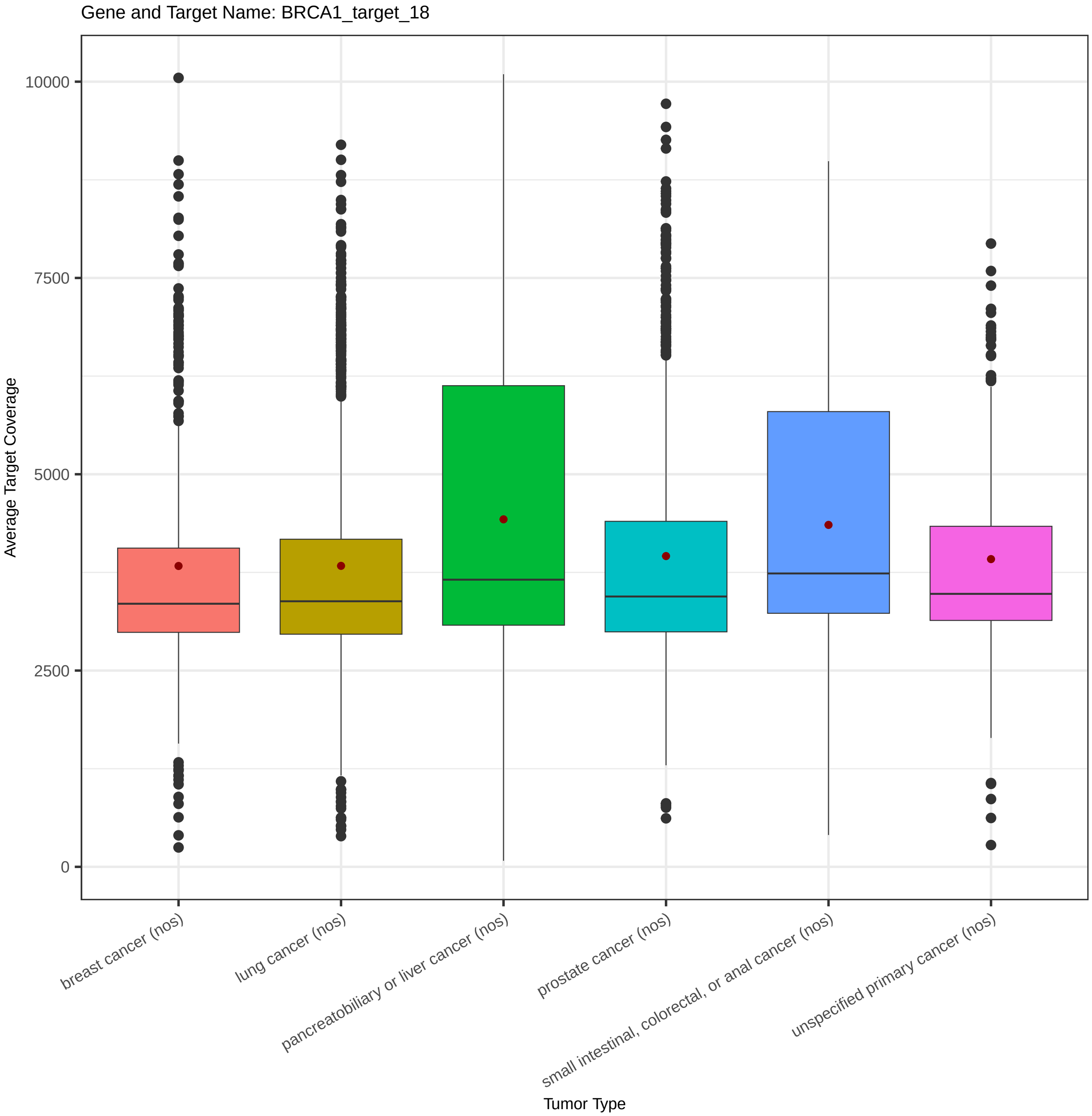
Gene and Target Name: BRCA1_target_18
Average Target Coverage
10000
7500
5000
2500
0
breast cancer (nos)
lung cancer (nos)
pancreatobiliary or liver cancer (nos)
prostate cancer (nos)
small intestinal, colorectal, or anal cancer (nos)
unspecified primary cancer (nos)
Tumor Type

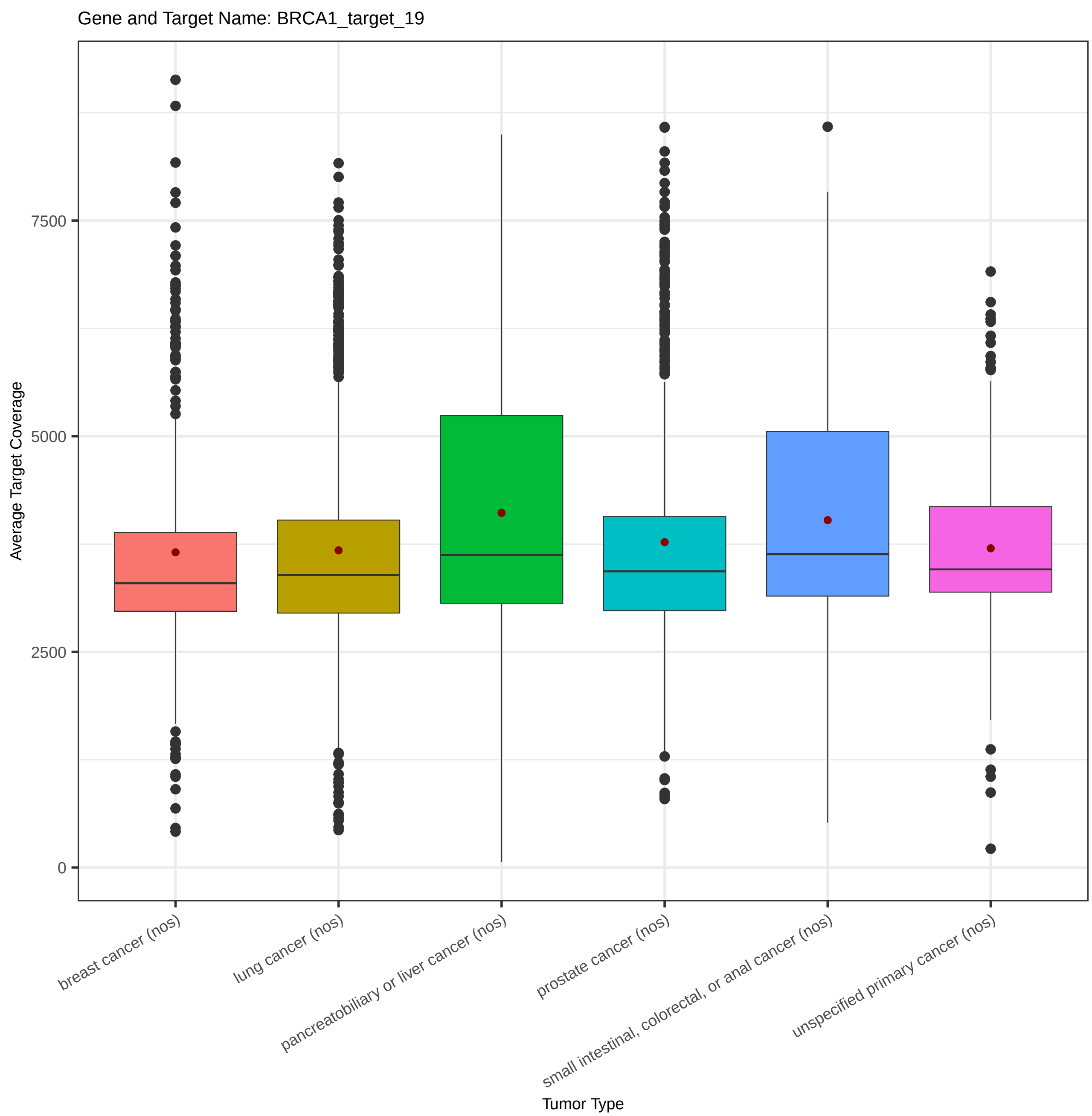

Gene and Target Name: BRCA1_target_19
Average Target Coverage
7500
5000
2500
0
breast cancer (nos)
lung cancer (nos)
pancreatobiliary or liver cancer (nos)
prostate cancer (nos)
small intestinal, colorectal, or anal cancer (nos)
unspecified primary cancer (nos)
Tumor Type

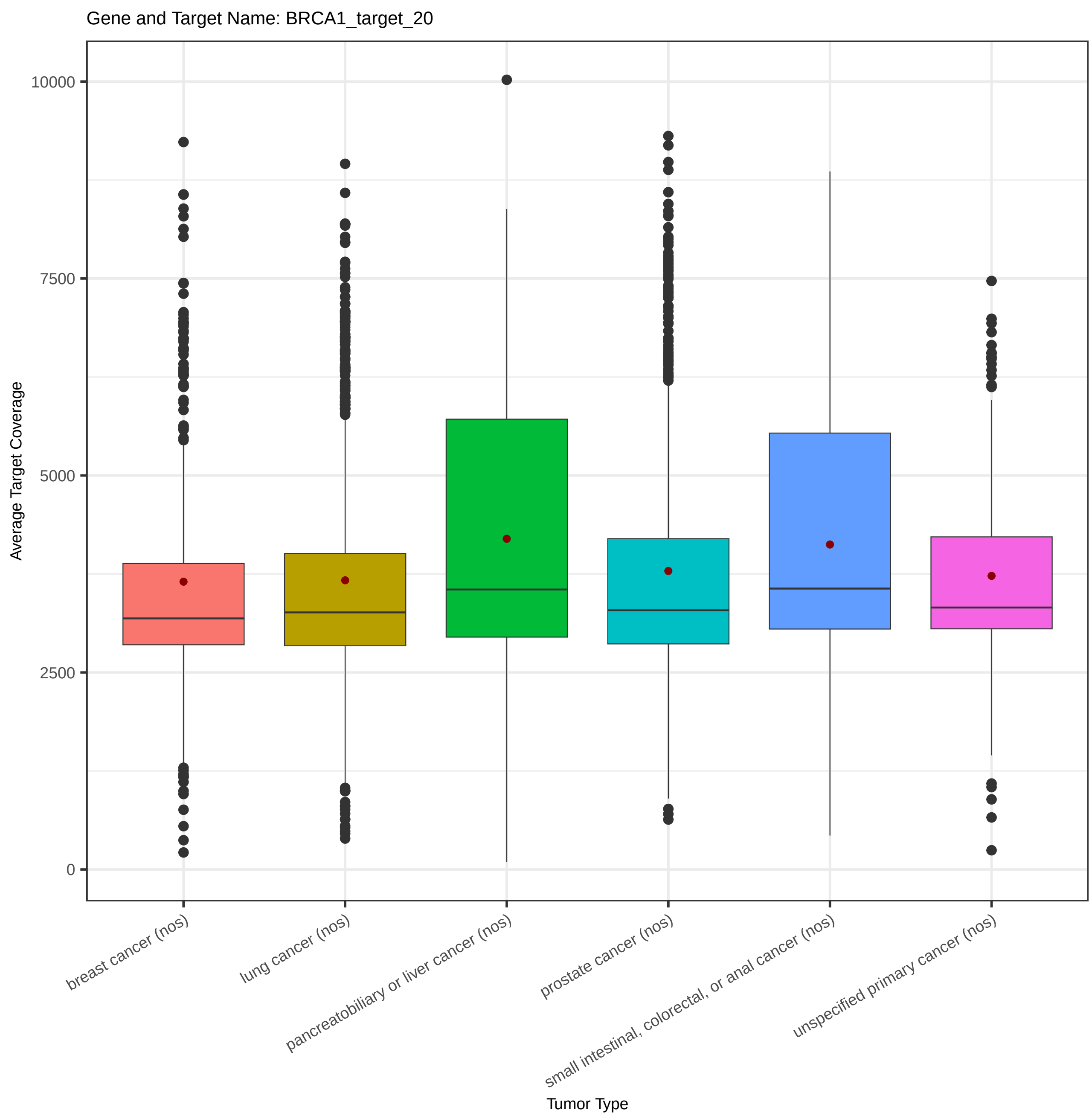
Gene and Target Name: BRCA1_target_20
Average Target Coverage
10000
7500
5000
2500
0
breast cancer (nos)
lung cancer (nos)
pancreatobiliary or liver cancer (nos)
prostate cancer (nos)
small intestinal, colorectal, or anal cancer (nos)
unspecified primary cancer (nos)
Tumor Type

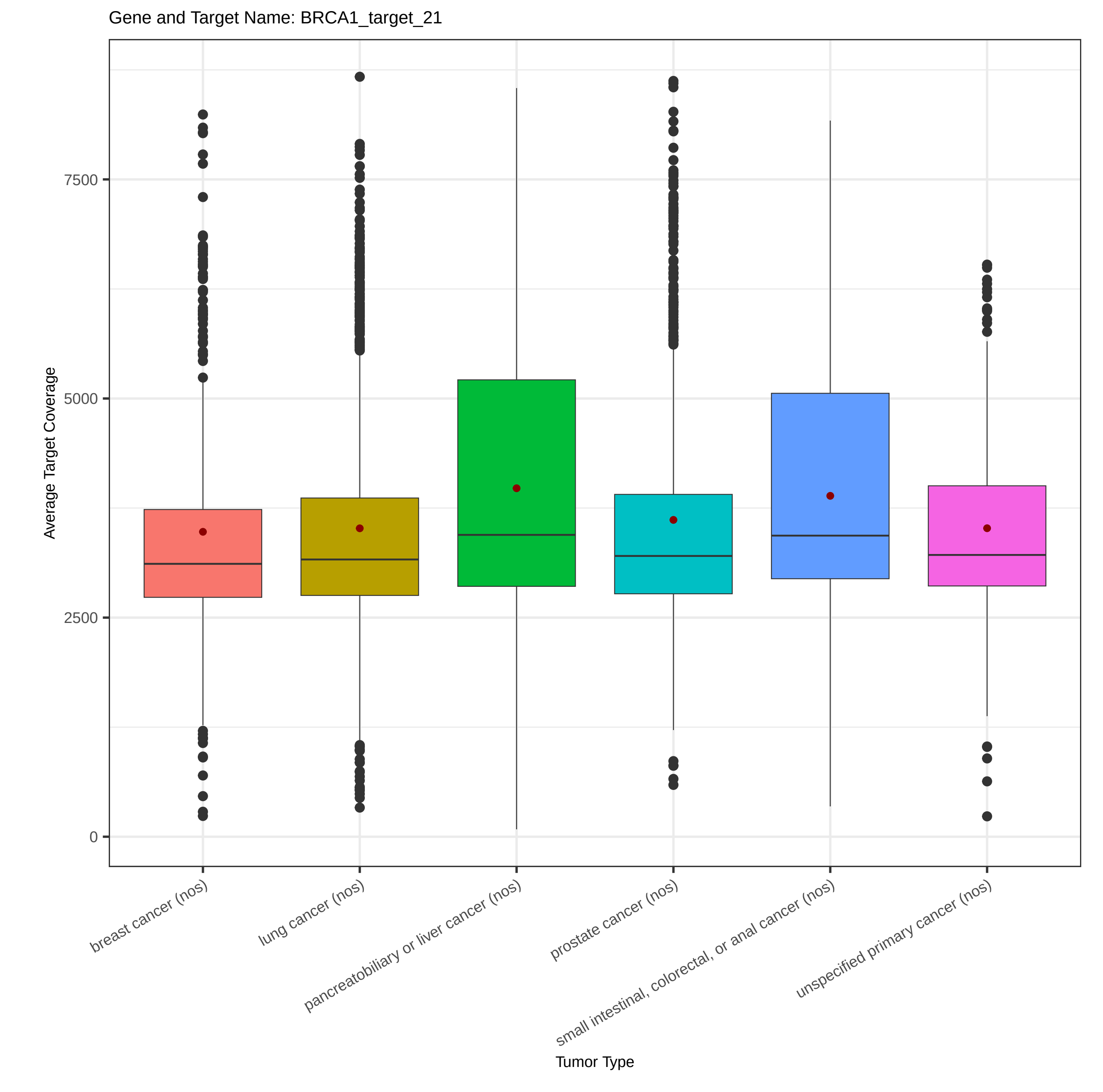
Gene and Target Name: BRCA1_target_21
Average Target Coverage
7500
5000
2500
0
breast cancer (nos)
lung cancer (nos)
pancreatobiliary or liver cancer (nos)
prostate cancer (nos)
small intestinal, colorectal, or anal cancer (nos)
unspecified primary cancer (nos)
Tumor Type

Gene and Target Name: BRCA1_target_22

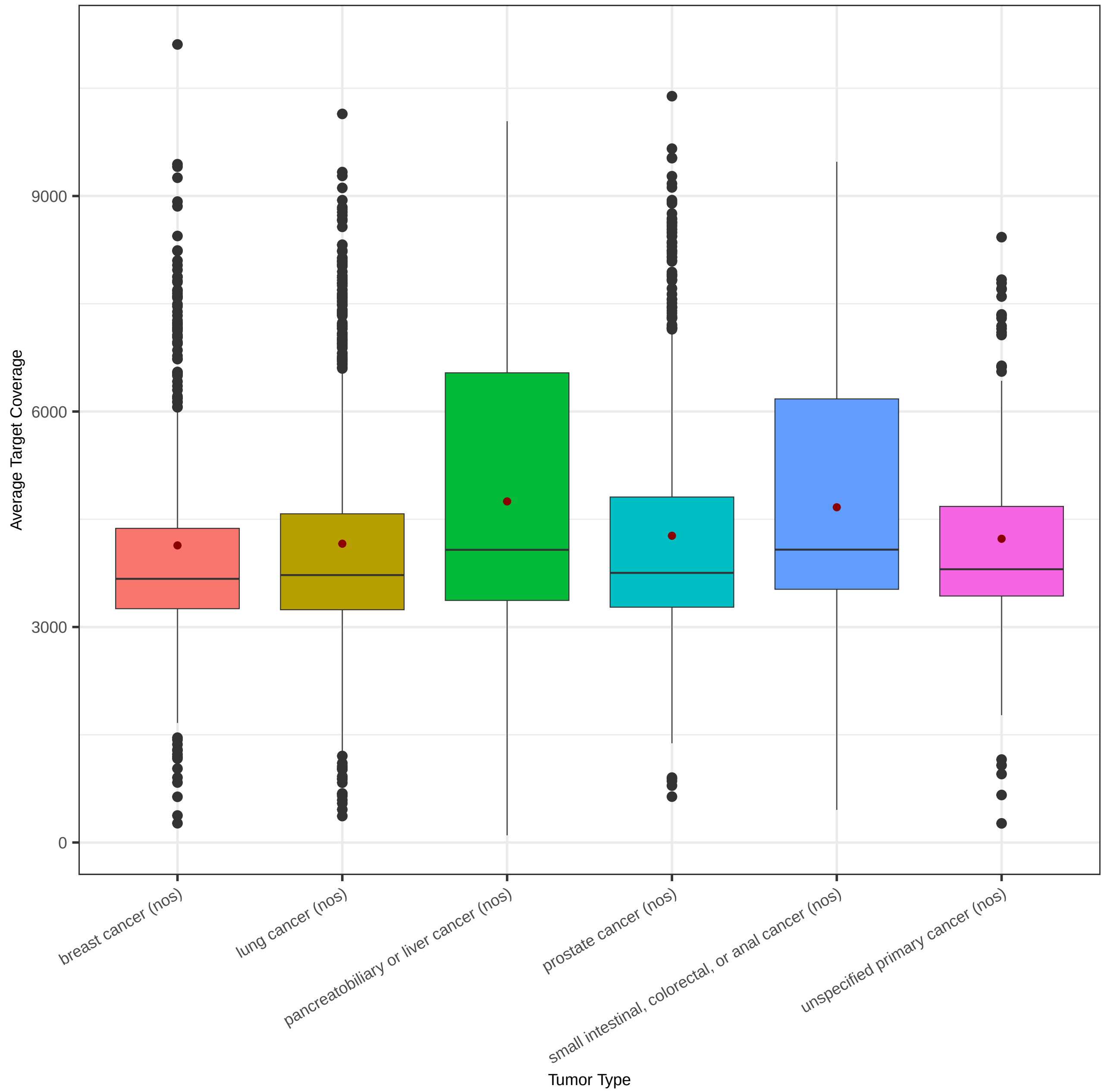

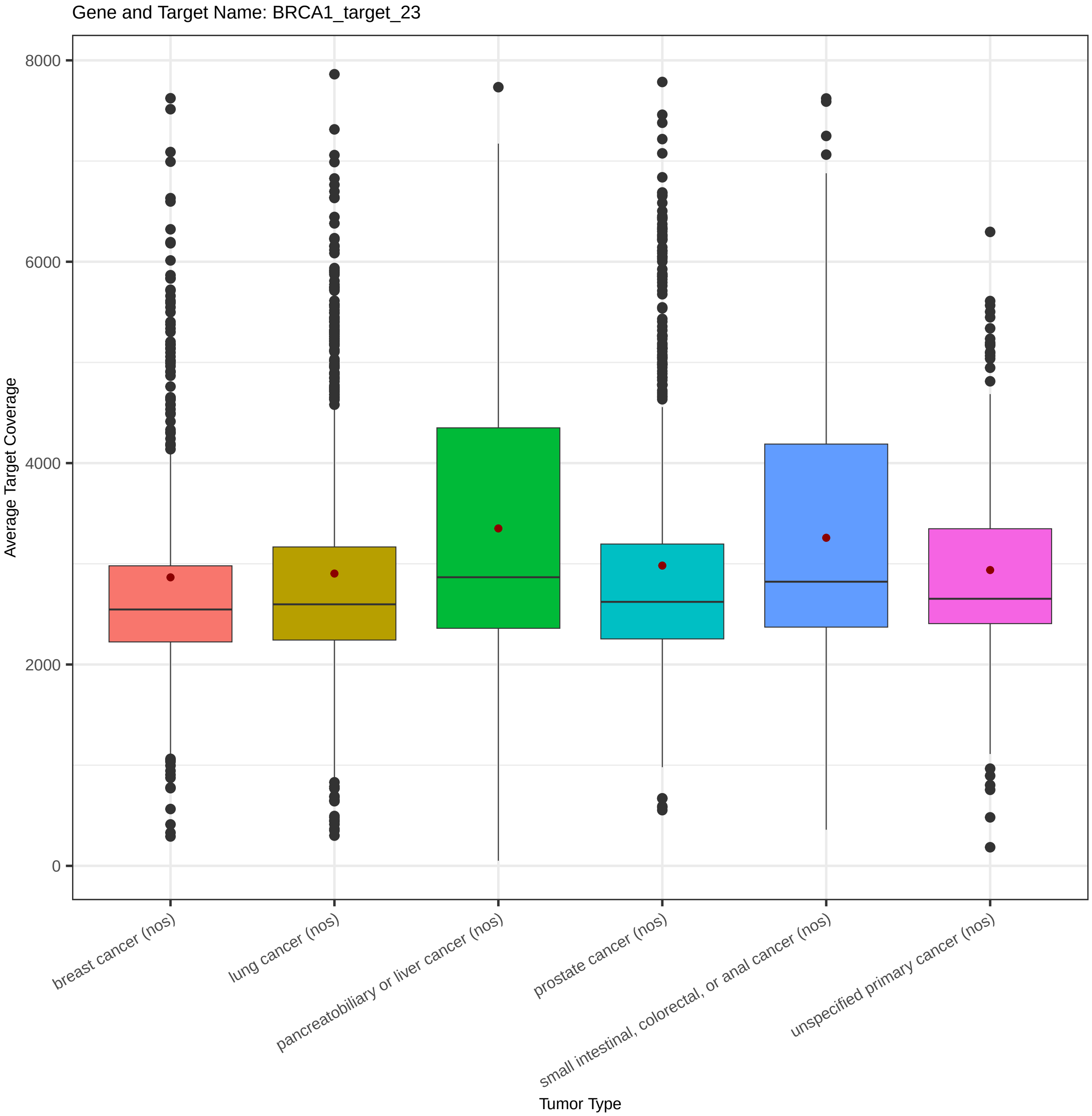

Gene and Target Name: BRCA1_target_23
Average Target Coverage
8000
6000
4000
2000
0
breast cancer (nos)
lung cancer (nos)
pancreatobiliary or liver cancer (nos)
prostate cancer (nos)
small intestinal, colorectal, or anal cancer (nos)
unspecified primary cancer (nos)
Tumor Type

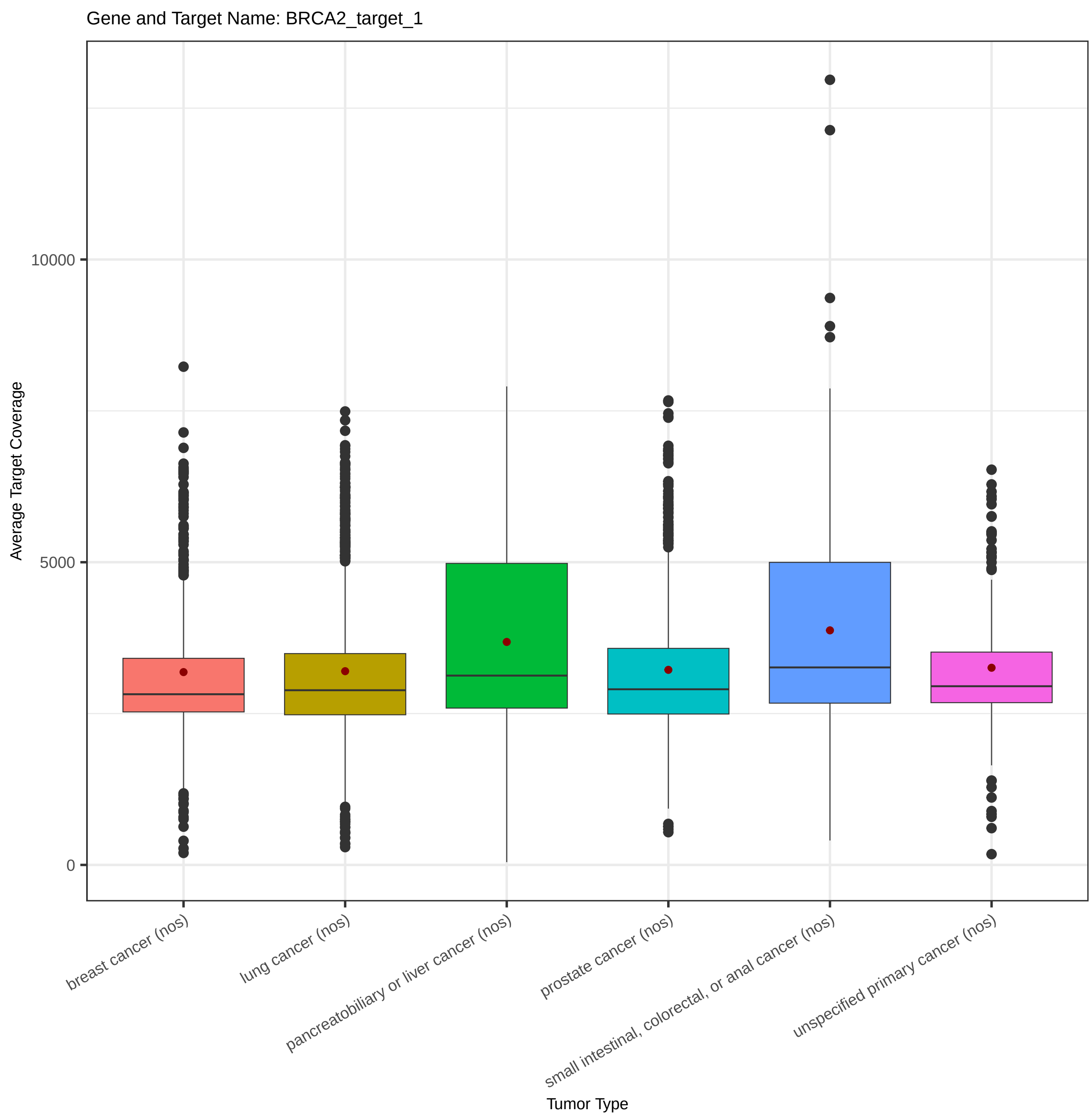

Gene and Target Name: BRCA2_target_1
Average Target Coverage
10000
5000
0
breast cancer (nos)
lung cancer (nos)
pancreatobiliary or liver cancer (nos)
prostate cancer (nos)
small intestinal, colorectal, or anal cancer (nos)
unspecified primary cancer (nos)
Tumor Type

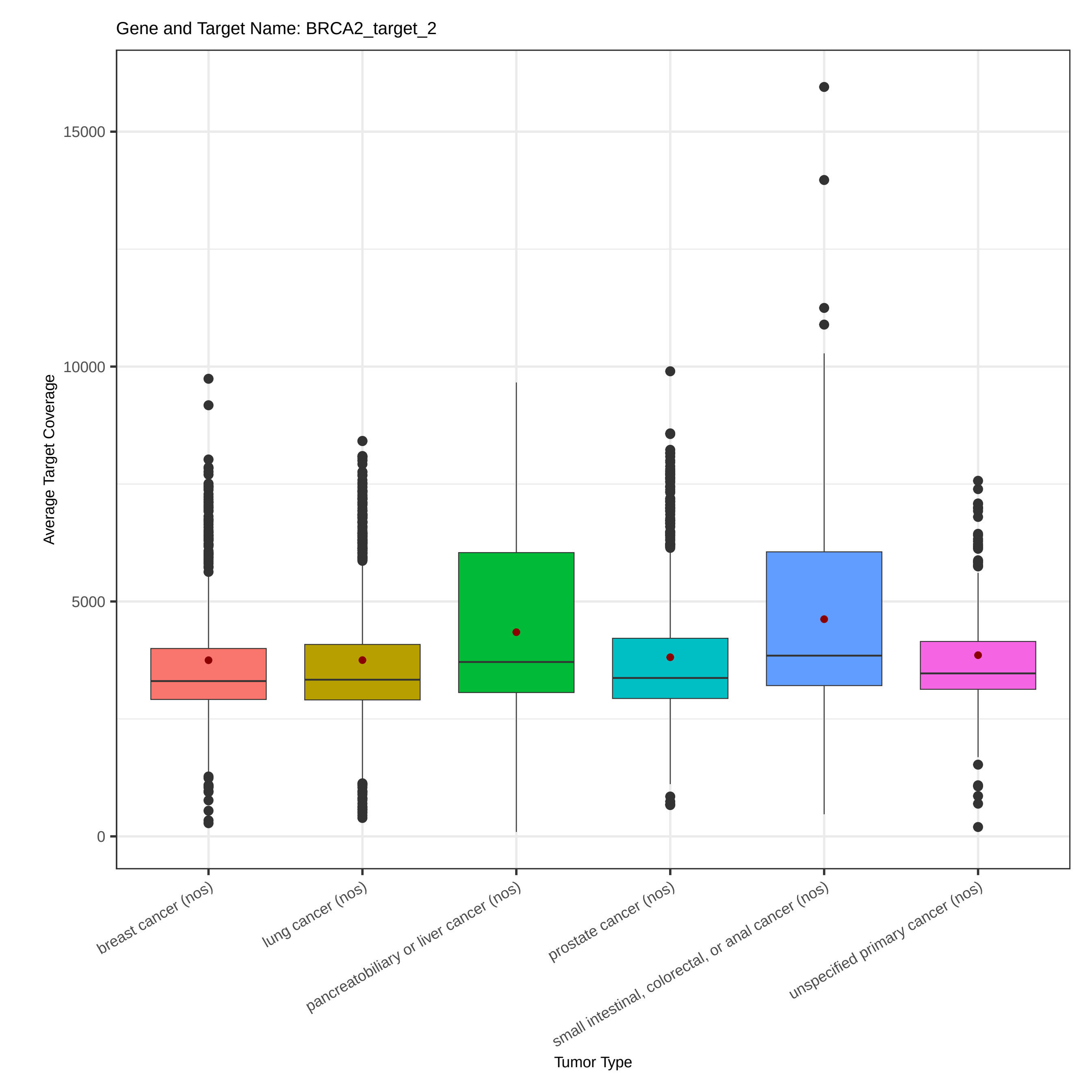

Gene and Target Name: BRCA2_target_2
Average Target Coverage
15000
10000
5000
0
breast cancer (nos)
lung cancer (nos)
pancreatobiliary or liver cancer (nos)
prostate cancer (nos)
small intestinal, colorectal, or anal cancer (nos)
unspecified primary cancer (nos)
Tumor Type

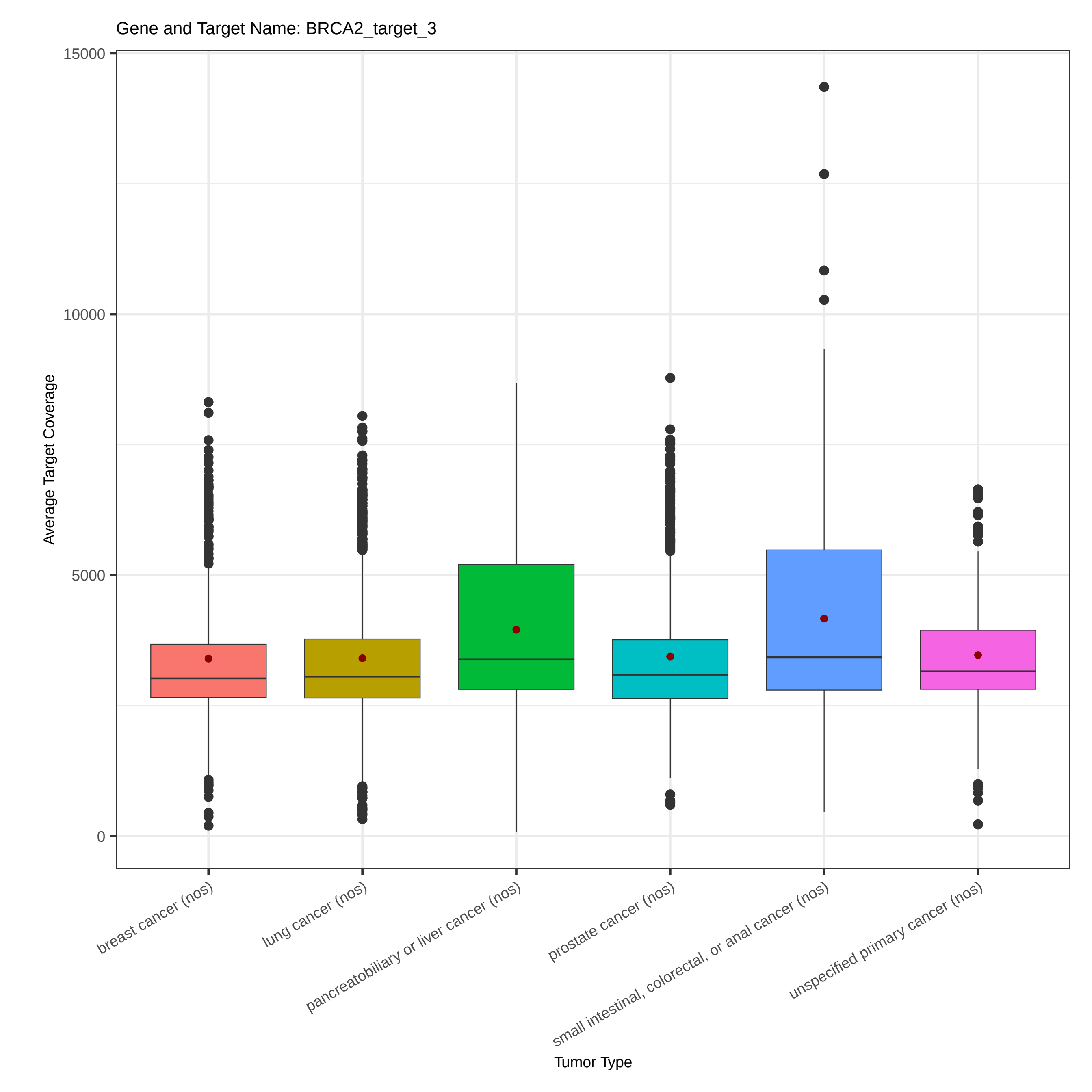

Gene and Target Name: BRCA2_target_3
15000
10000
5000
0
Average Target Coverage
breast cancer (nos)
lung cancer (nos)
pancreatobiliary or liver cancer (nos)
prostate cancer (nos)
small intestinal, colorectal, or anal cancer (nos)
unspecified primary cancer (nos)
Tumor Type

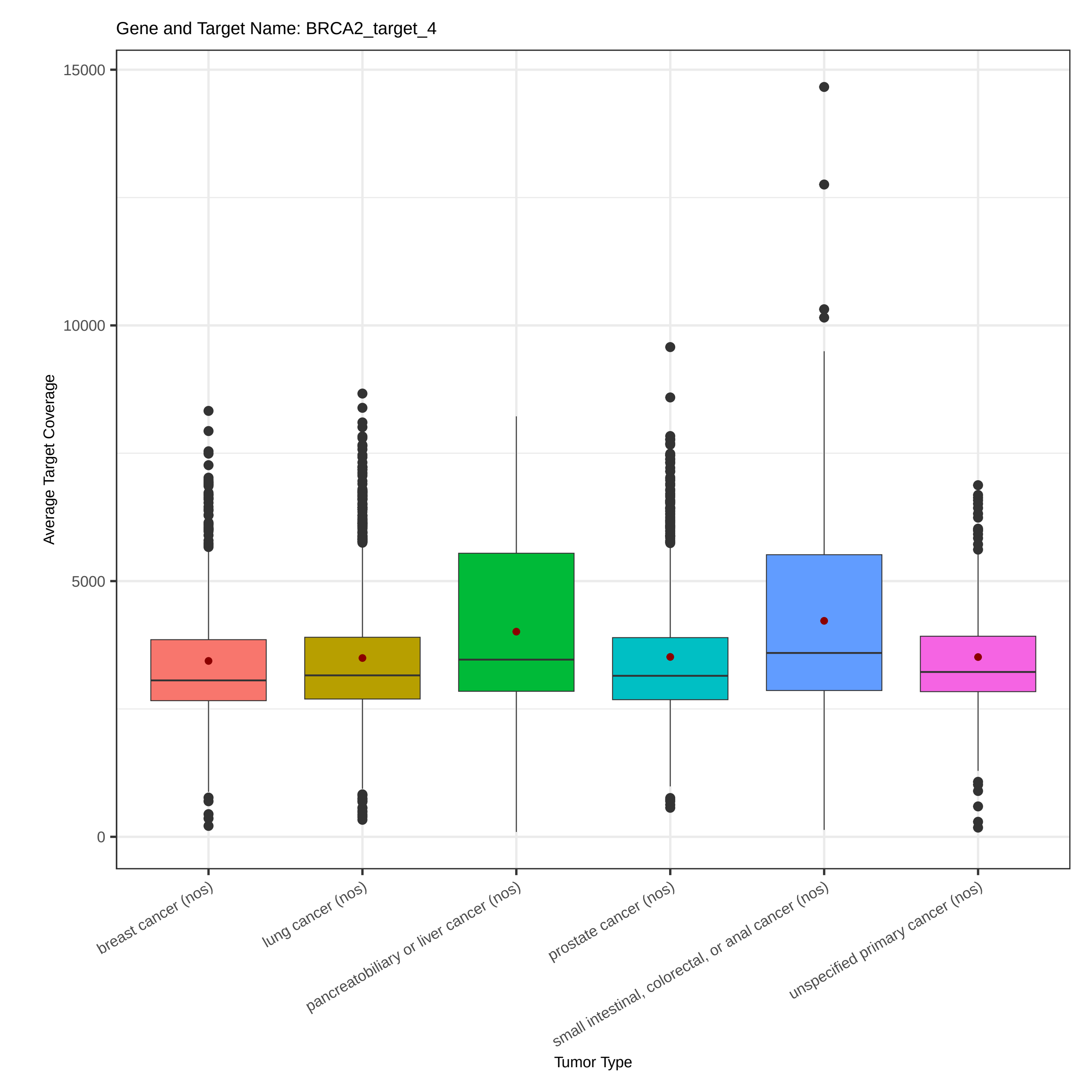
Gene and Target Name: BRCA2_target_4
Average Target Coverage
15000
10000
5000
0
breast cancer (nos)
lung cancer (nos)
pancreatobiliary or liver cancer (nos)
prostate cancer (nos)
small intestinal, colorectal, or anal cancer (nos)
unspecified primary cancer (nos)
Tumor Type

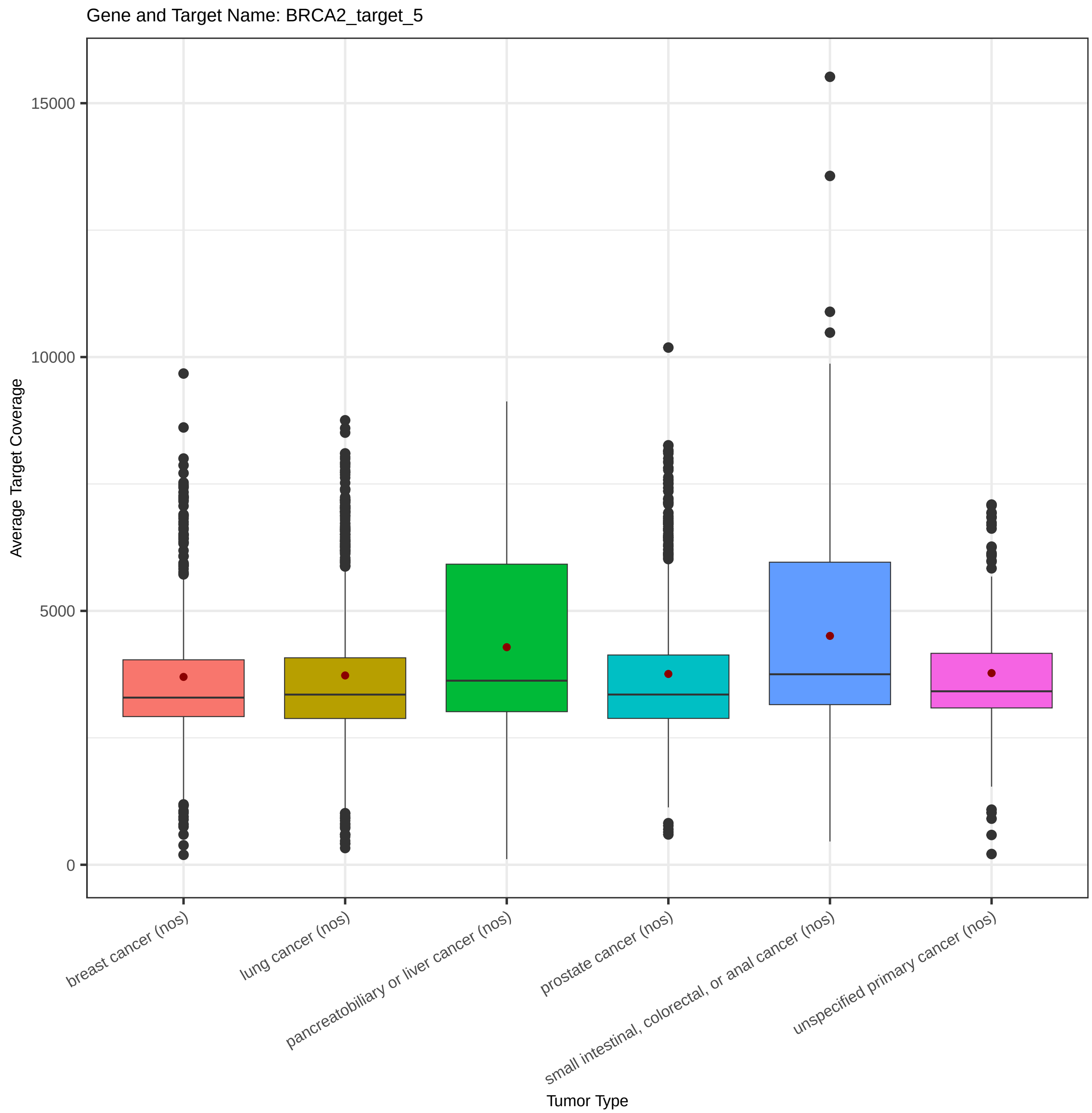

Gene and Target Name: BRCA2_target_5
Average Target Coverage
0
5000
10000
15000
breast cancer (nos)
lung cancer (nos)
pancreatobiliary or liver cancer (nos)
prostate cancer (nos)
small intestinal, colorectal, or anal cancer (nos)
unspecified primary cancer (nos)
Tumor Type

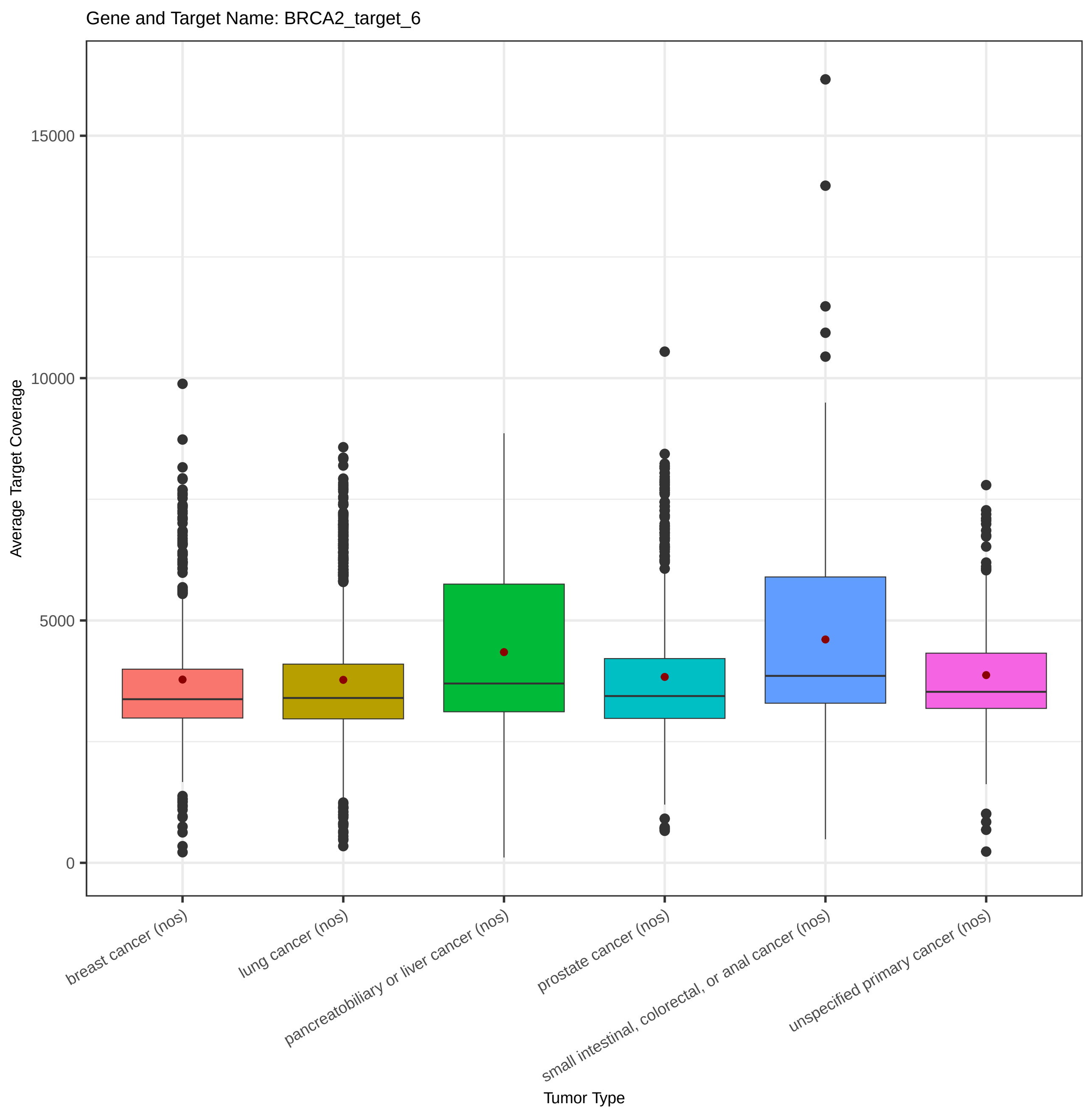

Gene and Target Name: BRCA2_target_6
Average Target Coverage
15000
10000
5000
0
breast cancer (nos)
lung cancer (nos)
pancreatobiliary or liver cancer (nos)
prostate cancer (nos)
small intestinal, colorectal, or anal cancer (nos)
unspecified primary cancer (nos)
Tumor Type

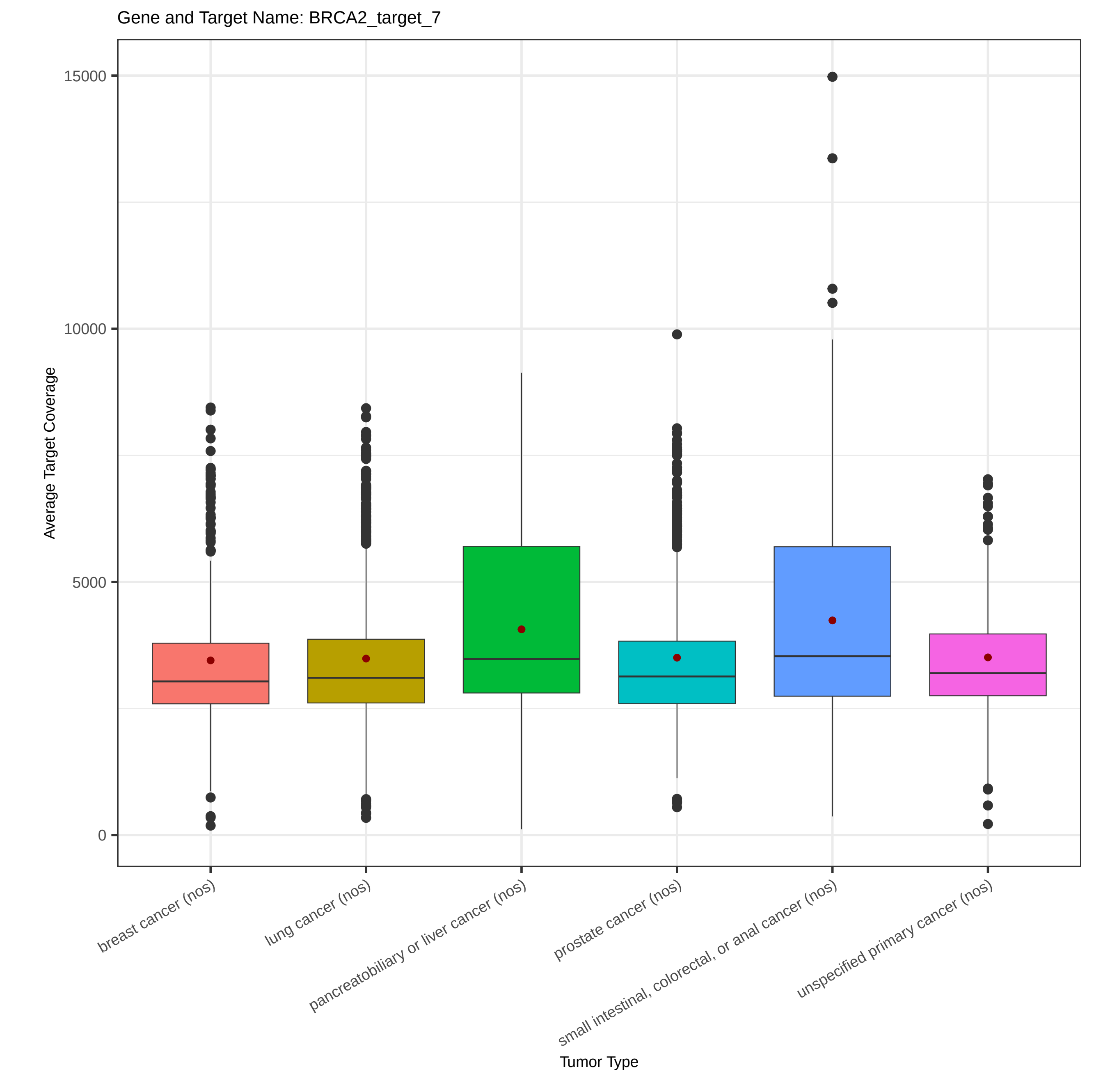

Gene and Target Name: BRCA2_target_7
Average Target Coverage
15000
10000
5000
0
breast cancer (nos)
lung cancer (nos)
pancreatobiliary or liver cancer (nos)
prostate cancer (nos)
small intestinal, colorectal, or anal cancer (nos)
unspecified primary cancer (nos)
Tumor Type

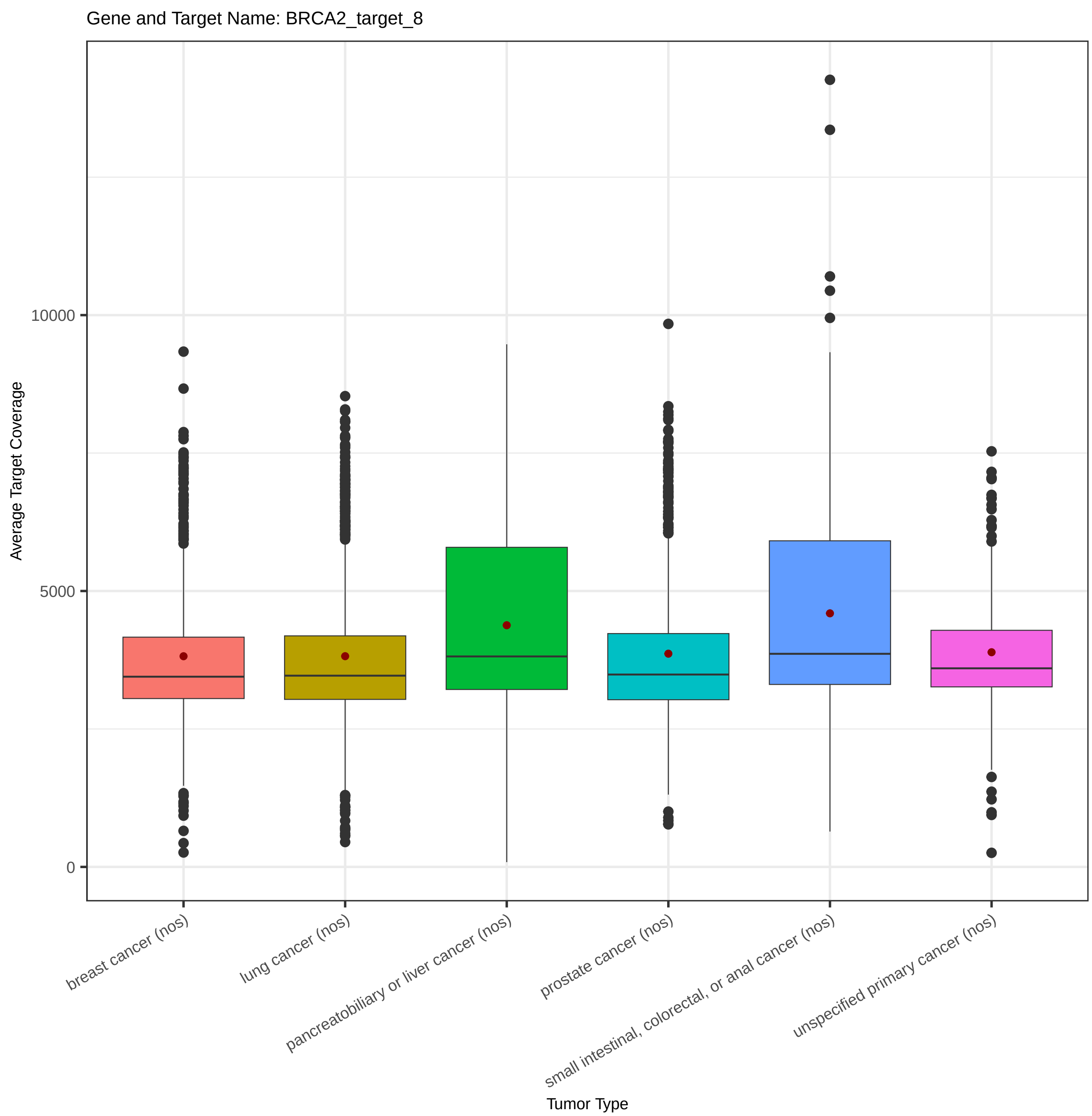
Gene and Target Name: BRCA2_target_8
Average Target Coverage
10000
5000
0
breast cancer (nos)
lung cancer (nos)
pancreatobiliary or liver cancer (nos)
prostate cancer (nos)
small intestinal, colorectal, or anal cancer (nos)
unspecified primary cancer (nos)
Tumor Type

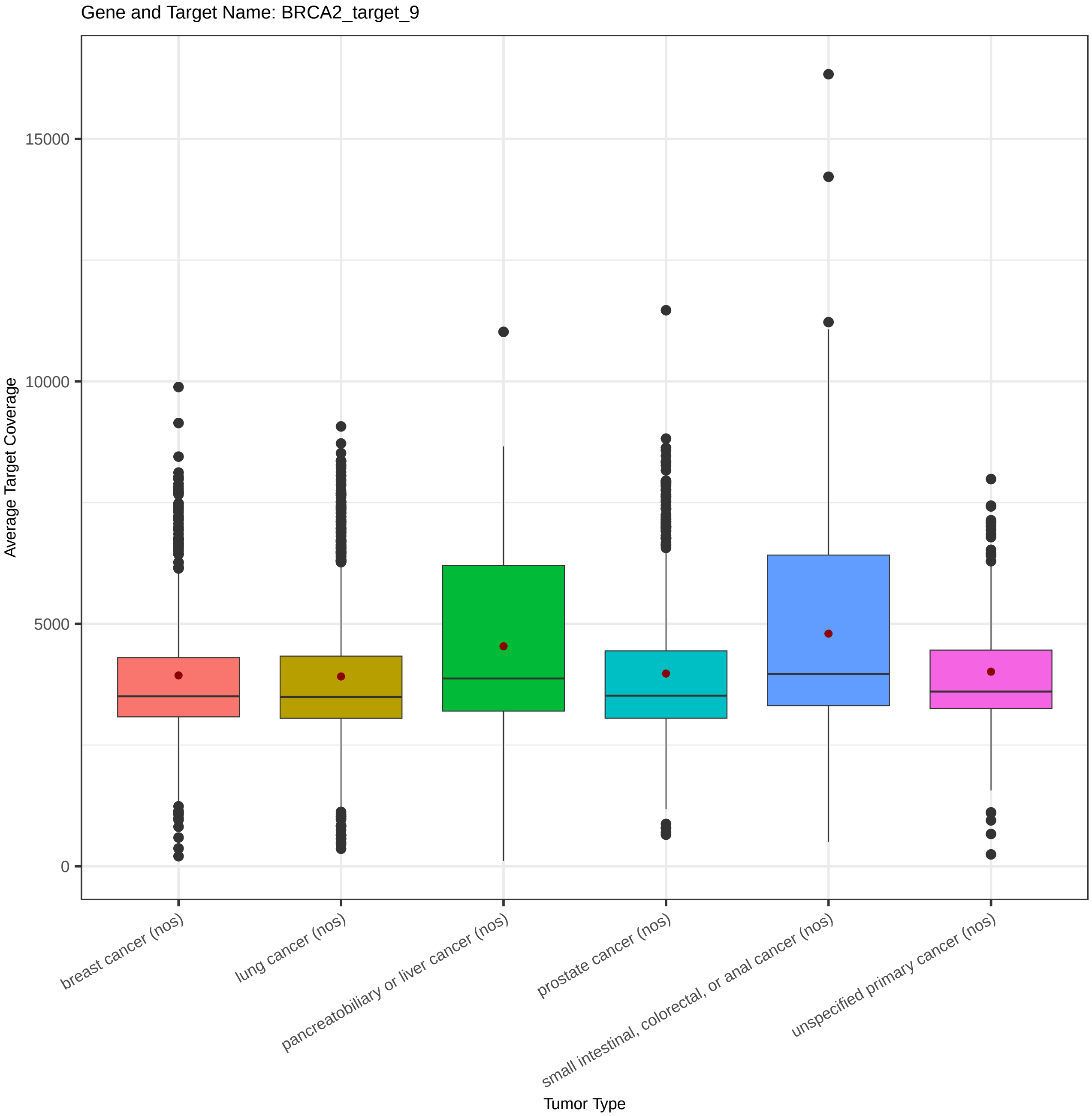

Gene and Target Name: BRCA2_target_9
Average Target Coverage
15000
10000
5000
0
breast cancer (nos)
lung cancer (nos)
pancreatobiliary or liver cancer (nos)
prostate cancer (nos)
small intestinal, colorectal, or anal cancer (nos)
unspecified primary cancer (nos)
Tumor Type

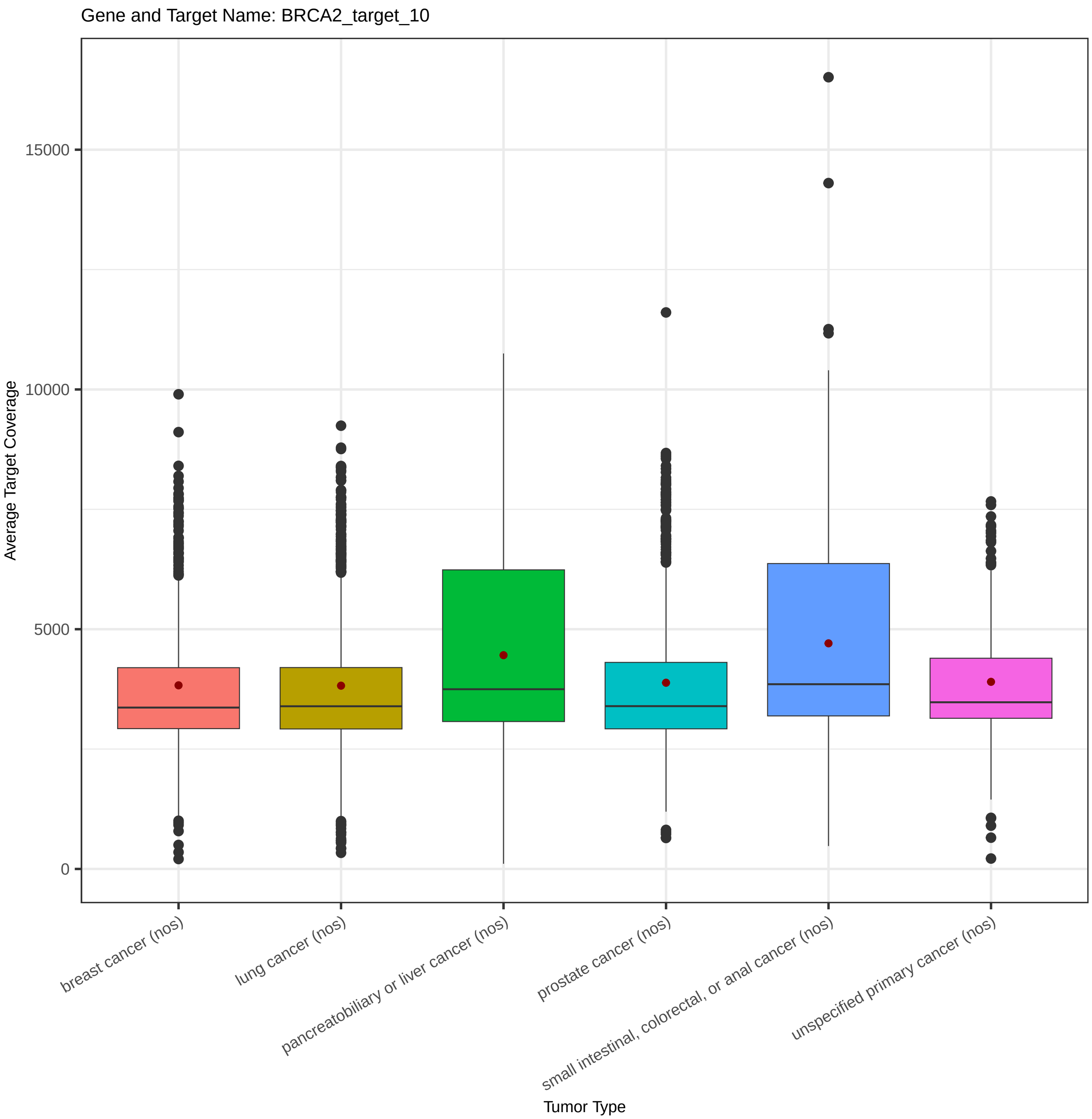

Gene and Target Name: BRCA2_target_10
Average Target Coverage
15000
10000
5000
0
breast cancer (nos)
lung cancer (nos)
pancreatobiliary or liver cancer (nos)
prostate cancer (nos)
small intestinal, colorectal, or anal cancer (nos)
unspecified primary cancer (nos)
Tumor Type

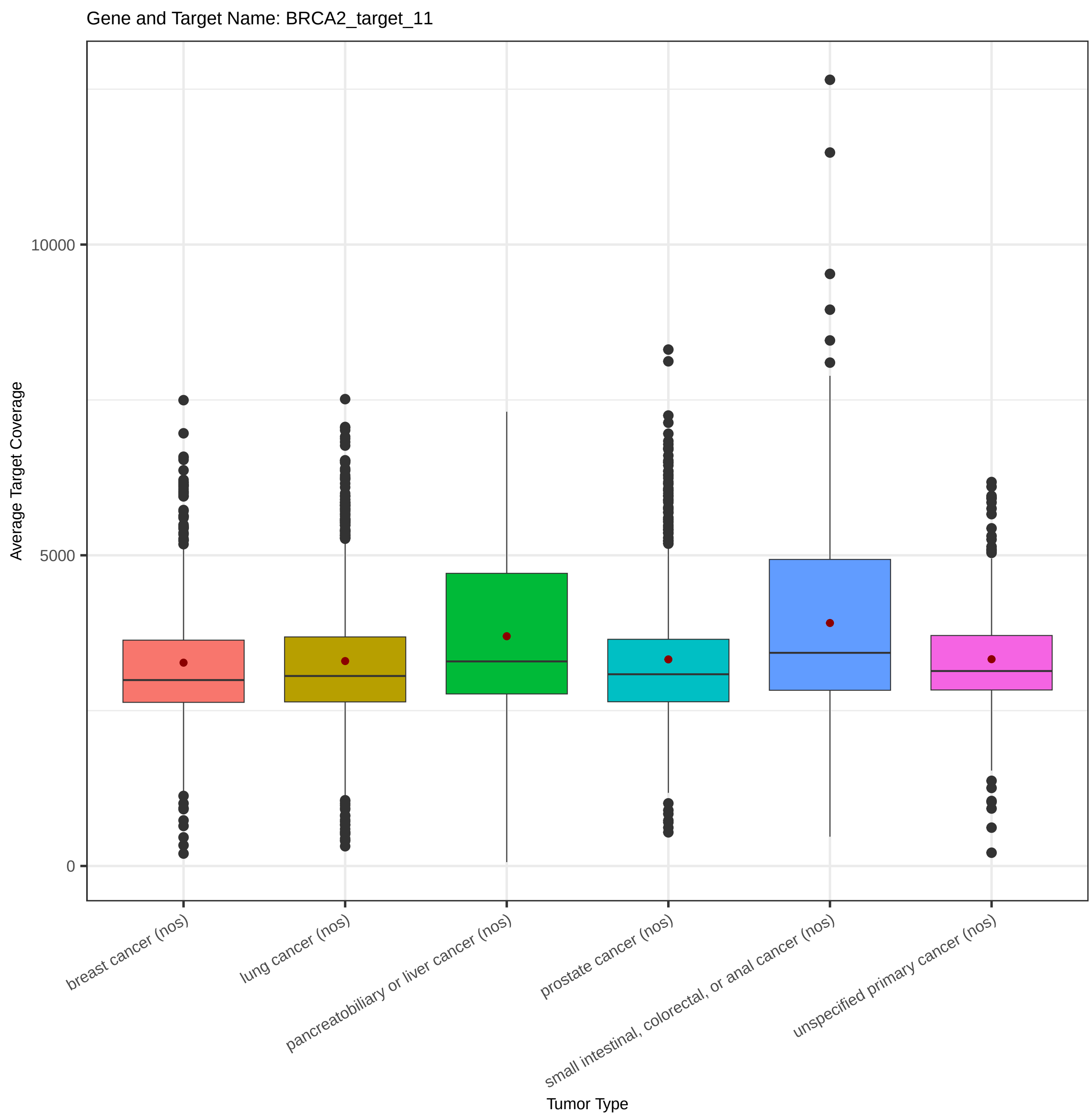
Gene and Target Name: BRCA2_target_11
Average Target Coverage
10000
5000
0
breast cancer (nos)
lung cancer (nos)
pancreatobiliary or liver cancer (nos)
prostate cancer (nos)
small intestinal, colorectal, or anal cancer (nos)
unspecified primary cancer (nos)
Tumor Type

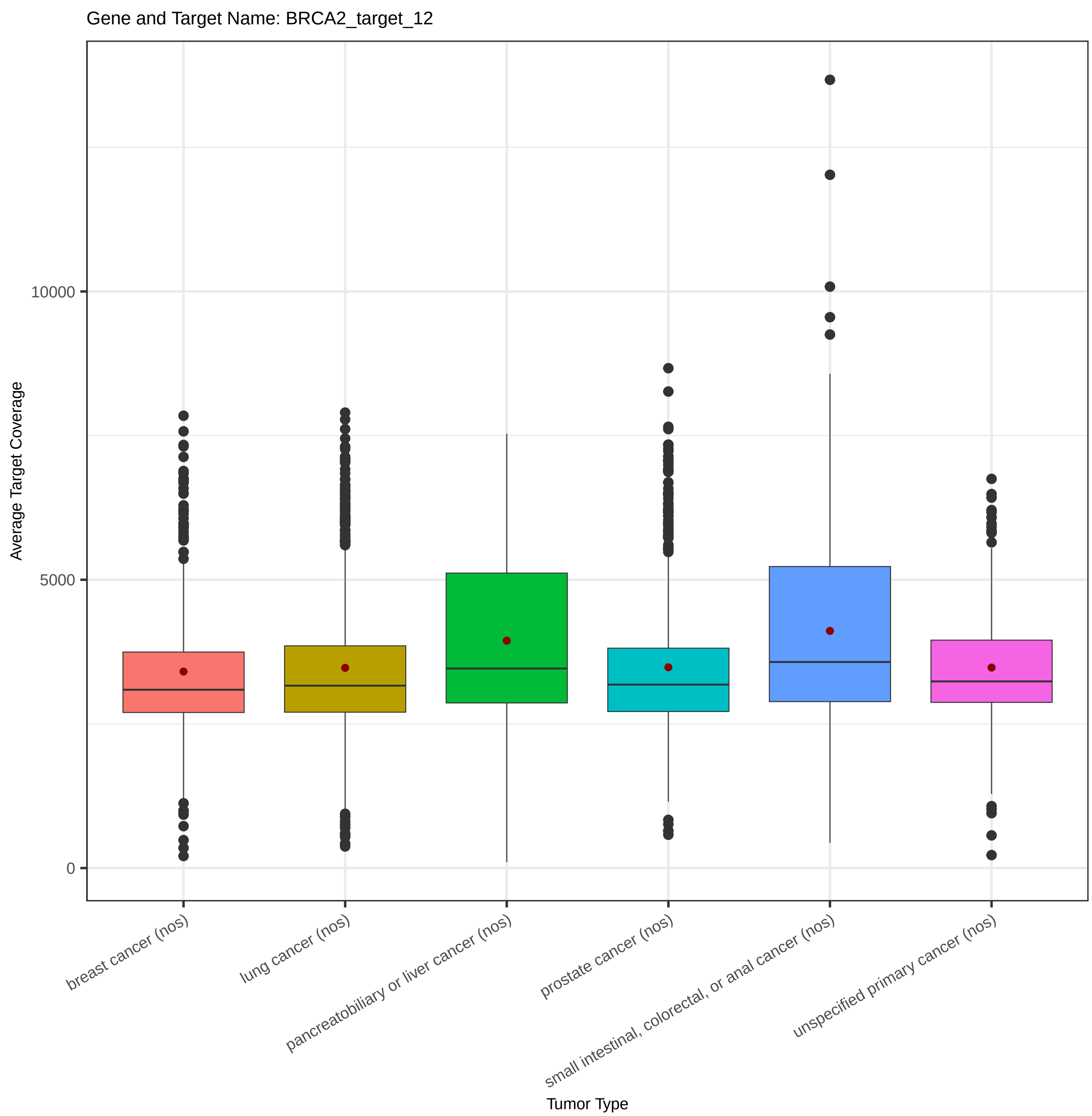

Gene and Target Name: BRCA2_target_12
Average Target Coverage
10000
5000
0
breast cancer (nos)
lung cancer (nos)
pancreatobiliary or liver cancer (nos)
prostate cancer (nos)
small intestinal, colorectal, or anal cancer (nos)
unspecified primary cancer (nos)
Tumor Type

Gene and Target Name: BRCA2_target_13

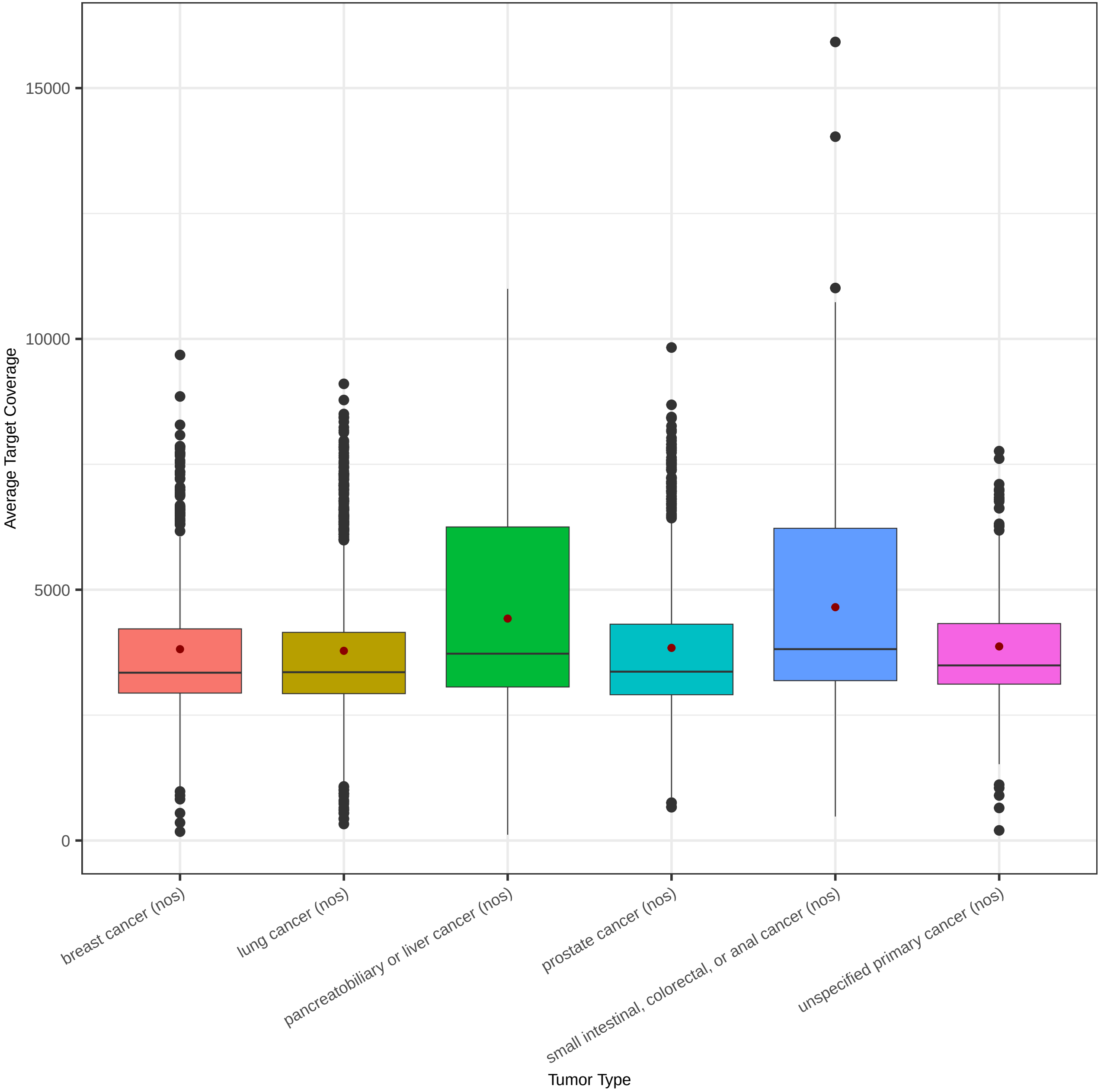

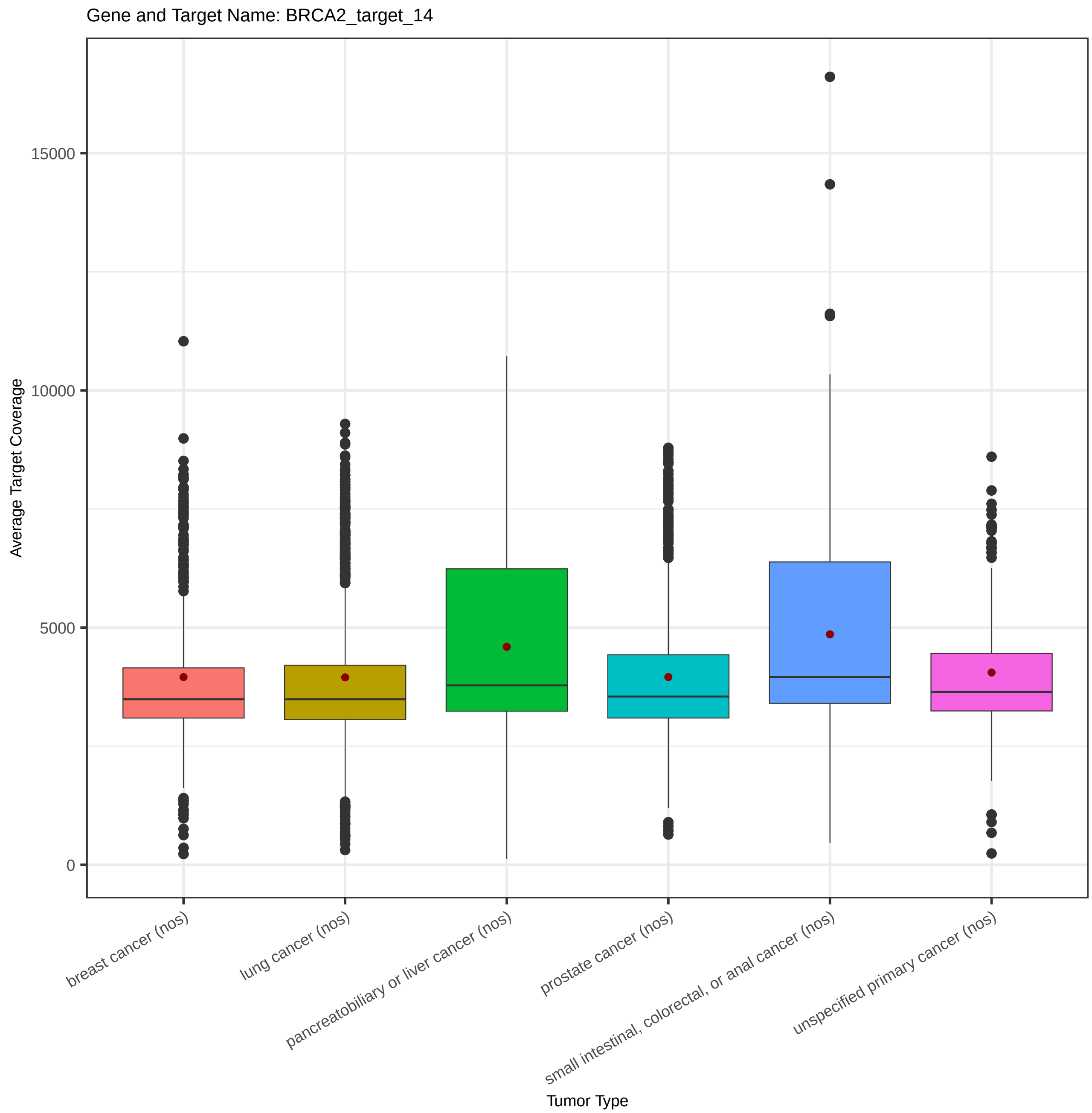
Gene and Target Name: BRCA2_target_14
Average Target Coverage
15000
10000
5000
0
breast cancer (nos)
lung cancer (nos)
pancreatobiliary or liver cancer (nos)
prostate cancer (nos)
small intestinal, colorectal, or anal cancer (nos)
unspecified primary cancer (nos)
Tumor Type

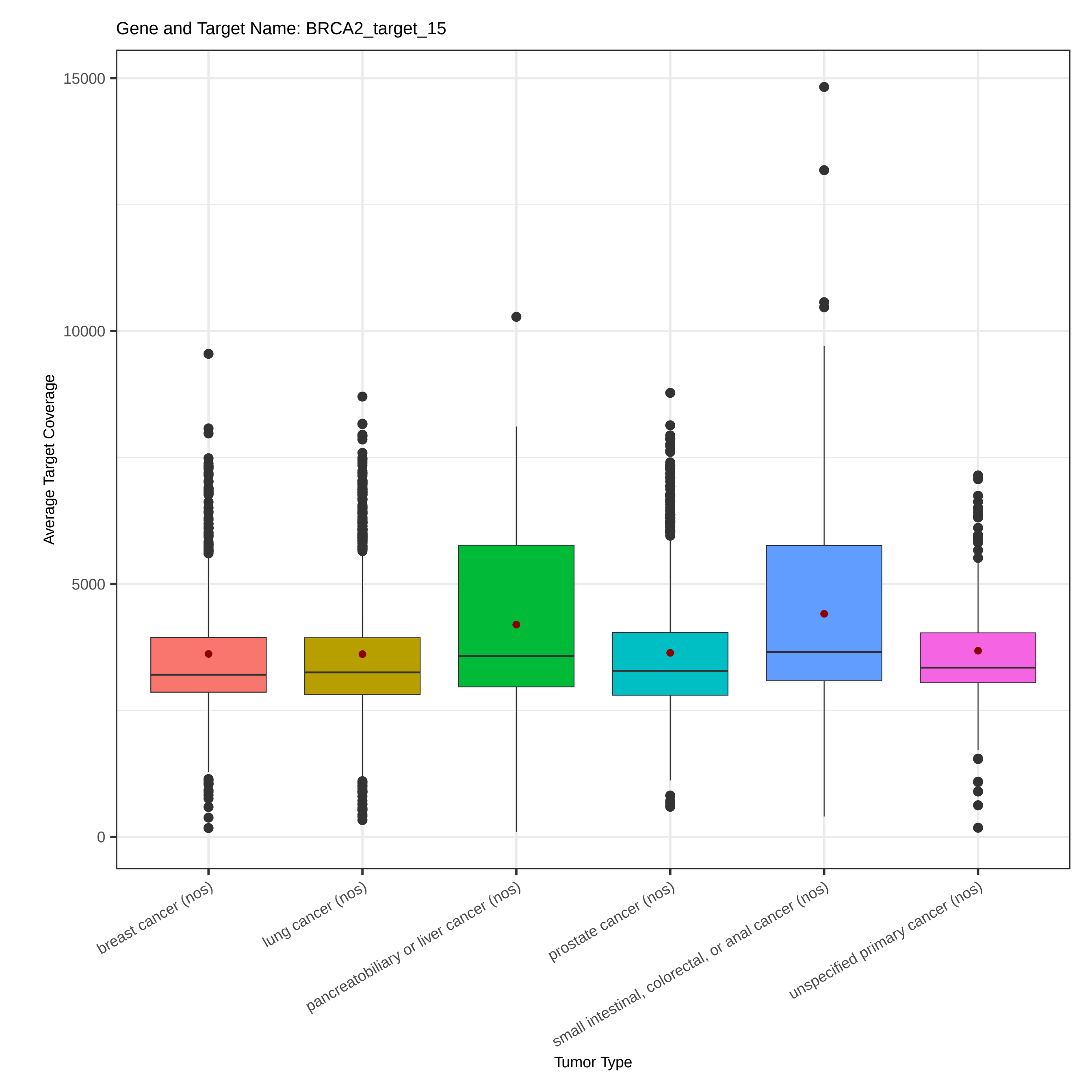

Gene and Target Name: BRCA2_target_15
Average Target Coverage
15000
10000
5000
0
breast cancer (nos)
lung cancer (nos)
pancreatobiliary or liver cancer (nos)
prostate cancer (nos)
small intestinal, colorectal, or anal cancer (nos)
unspecified primary cancer (nos)
Tumor Type

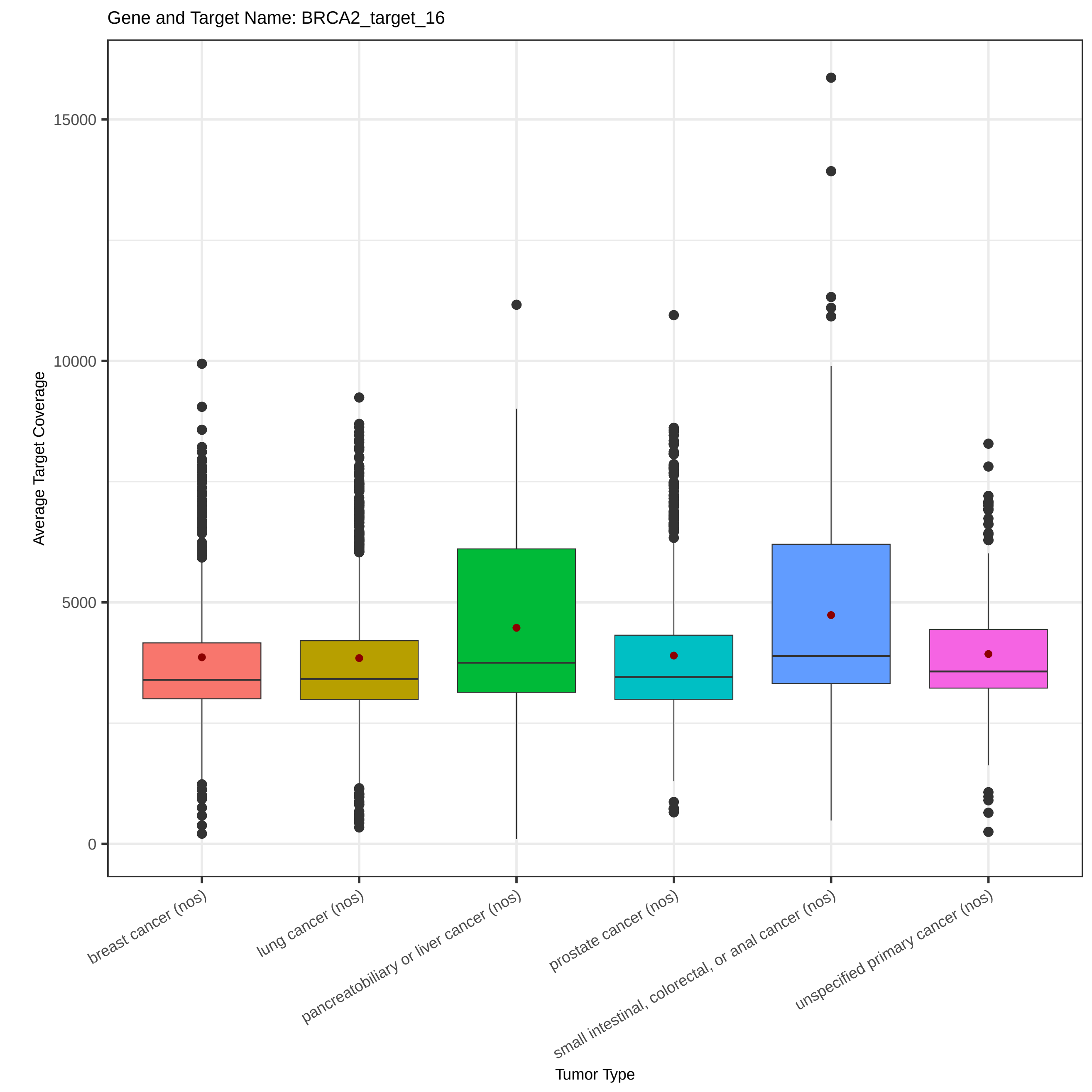
Gene and Target Name: BRCA2_target_16
Average Target Coverage
15000
10000
5000
0
breast cancer (nos)
lung cancer (nos)
pancreatobiliary or liver cancer (nos)
prostate cancer (nos)
small intestinal, colorectal, or anal cancer (nos)
unspecified primary cancer (nos)
Tumor Type

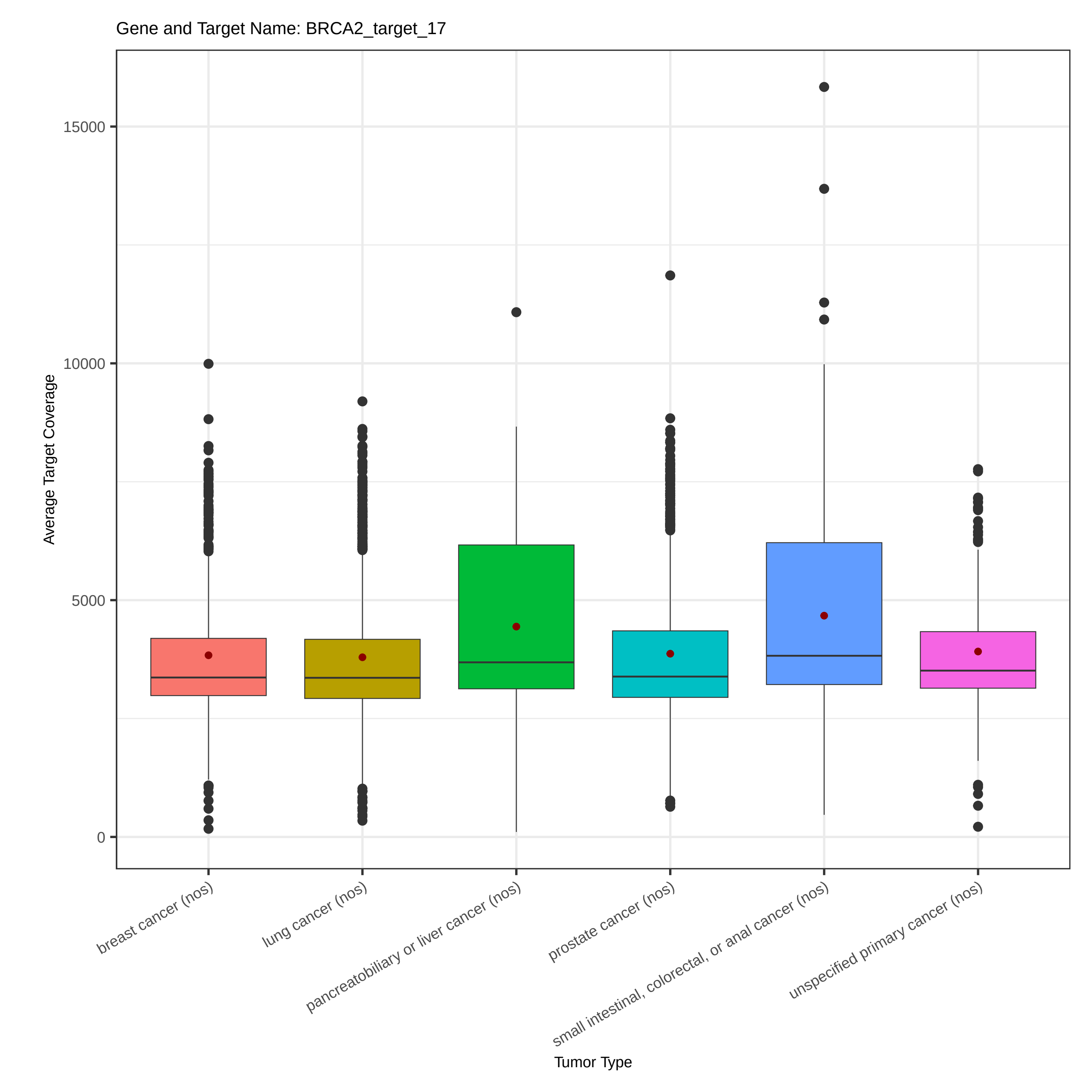
Gene and Target Name: BRCA2_target_17
Average Target Coverage
15000
10000
5000
0
breast cancer (nos)
lung cancer (nos)
pancreatobiliary or liver cancer (nos)
prostate cancer (nos)
small intestinal, colorectal, or anal cancer (nos)
unspecified primary cancer (nos)
Tumor Type

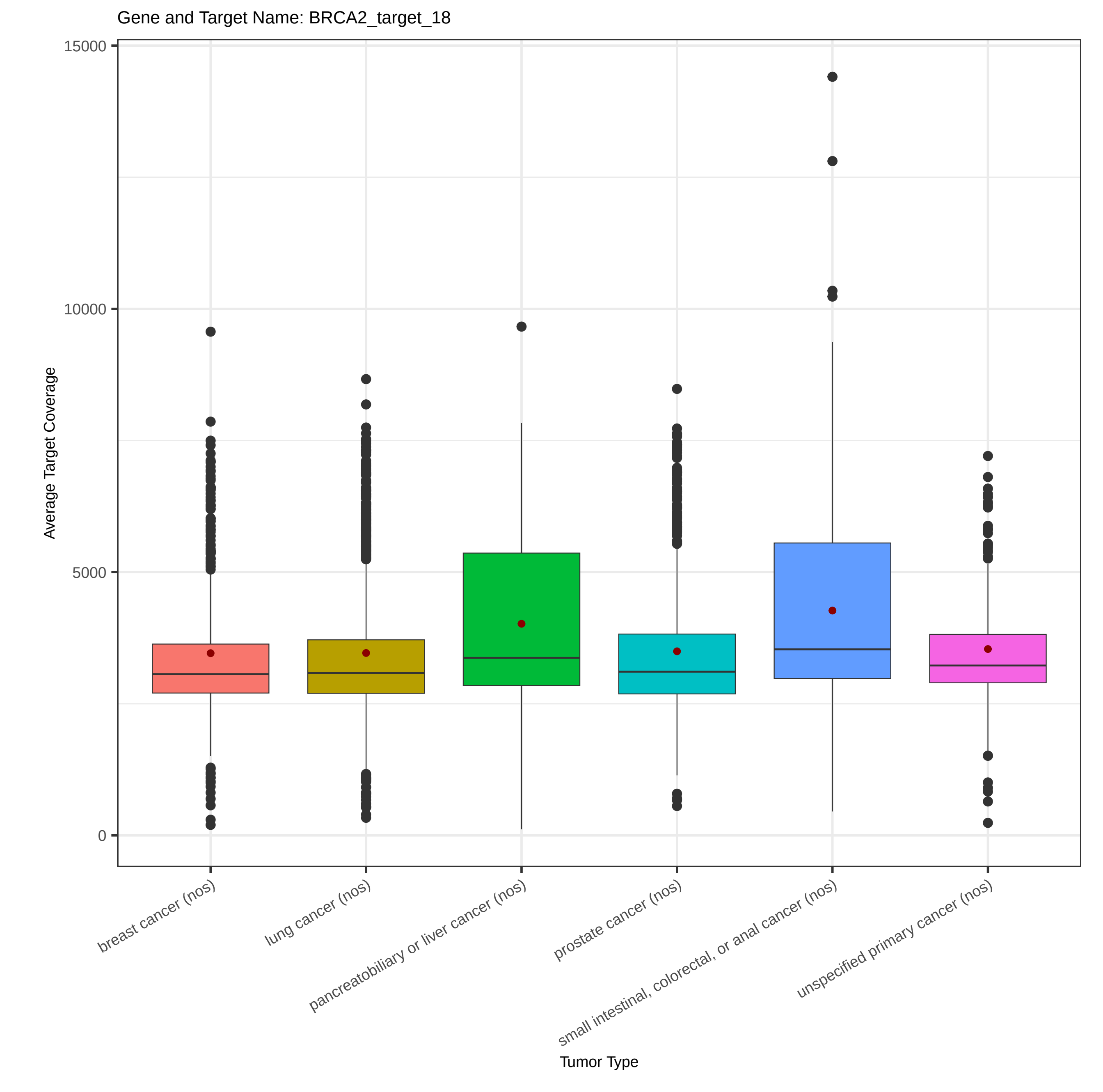
Gene and Target Name: BRCA2_target_18
Average Target Coverage
15000
10000
5000
0
breast cancer (nos)
lung cancer (nos)
pancreatobiliary or liver cancer (nos)
prostate cancer (nos)
small intestinal, colorectal, or anal cancer (nos)
unspecified primary cancer (nos)
Tumor Type

Gene and Target Name: BRCA2_target_19

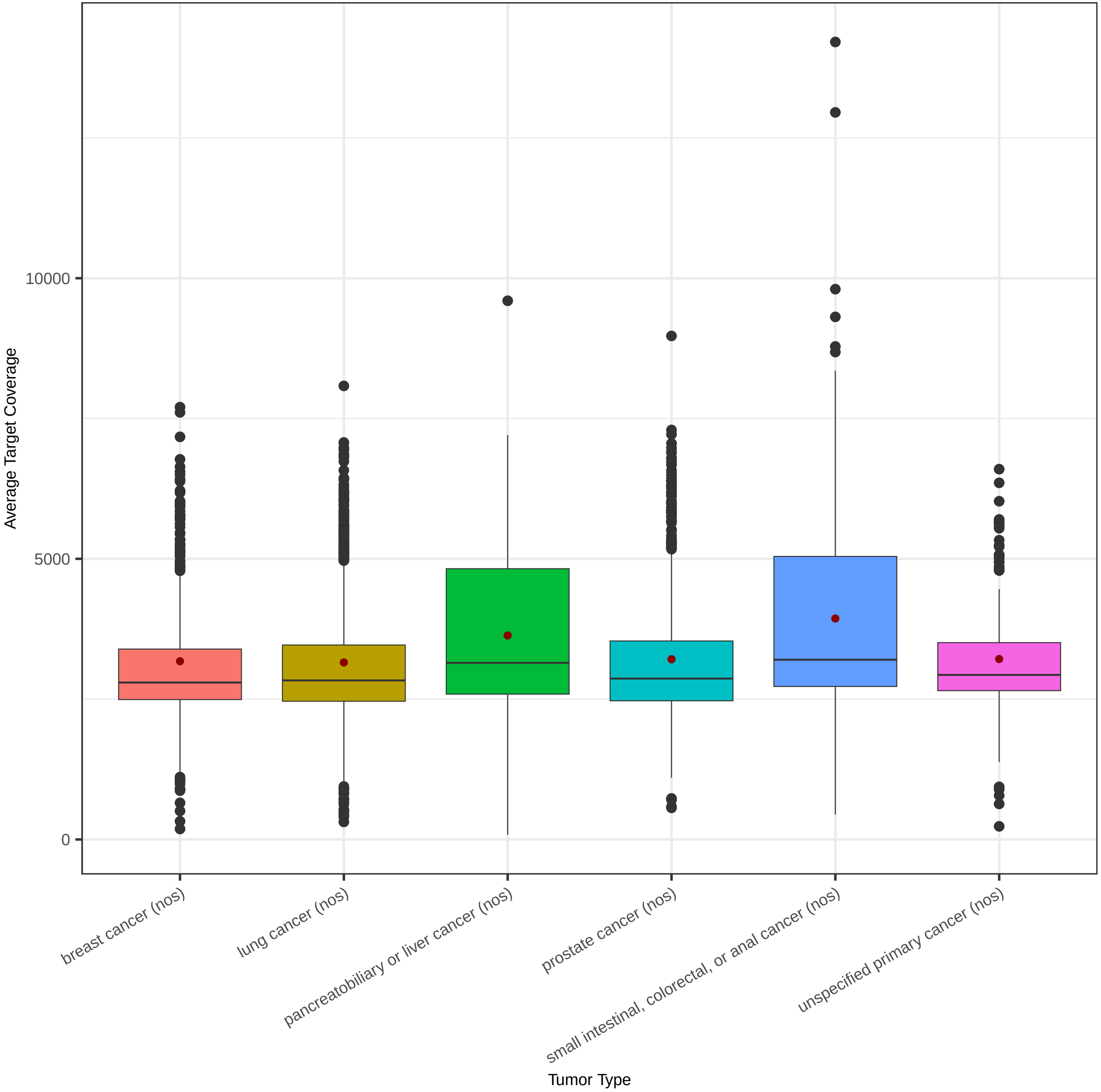

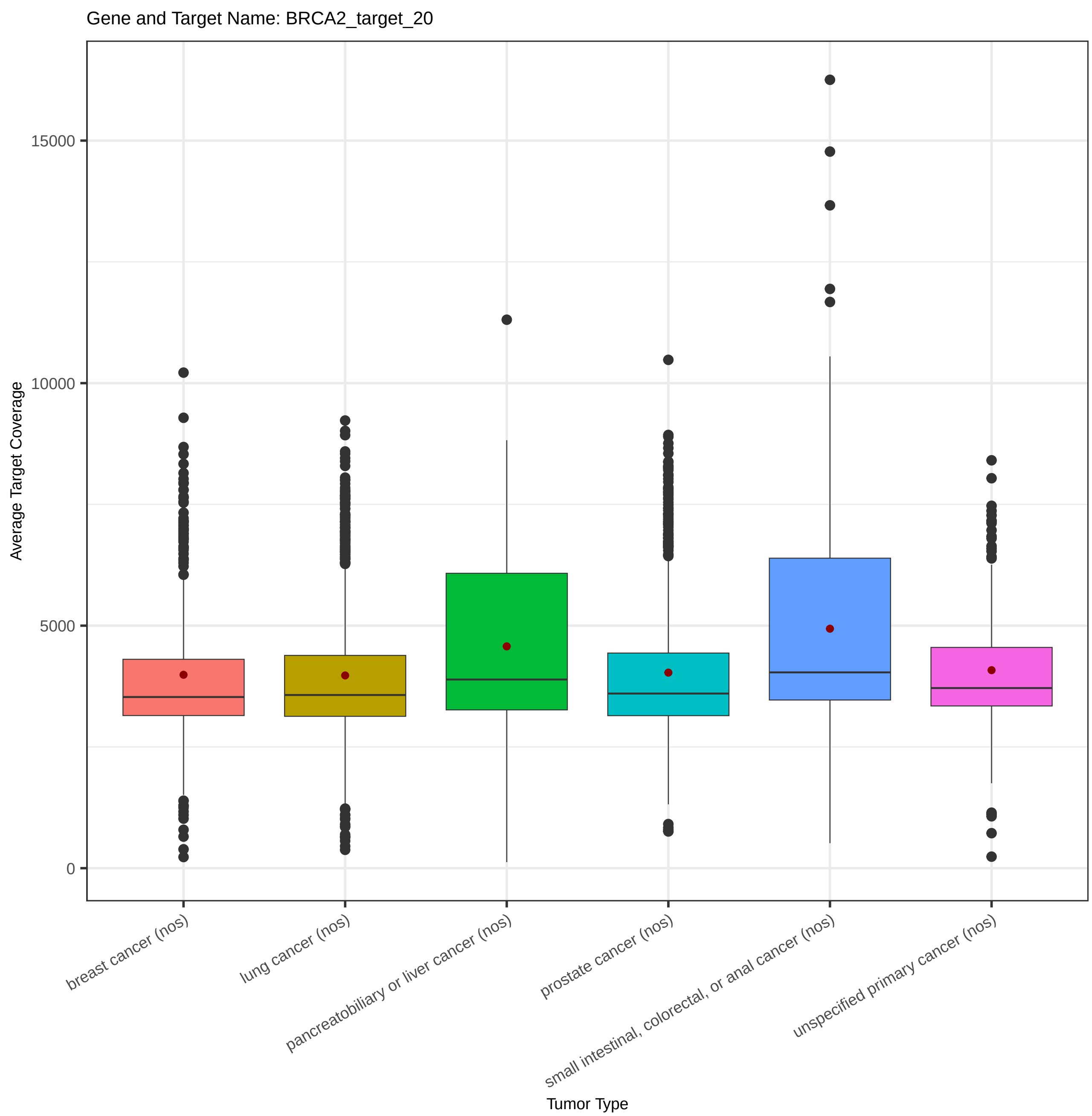
Gene and Target Name: BRCA2_target_20
Average Target Coverage
15000
10000
5000
0
breast cancer (nos)
lung cancer (nos)
pancreatobiliary or liver cancer (nos)
prostate cancer (nos)
small intestinal, colorectal, or anal cancer (nos)
unspecified primary cancer (nos)
Tumor Type

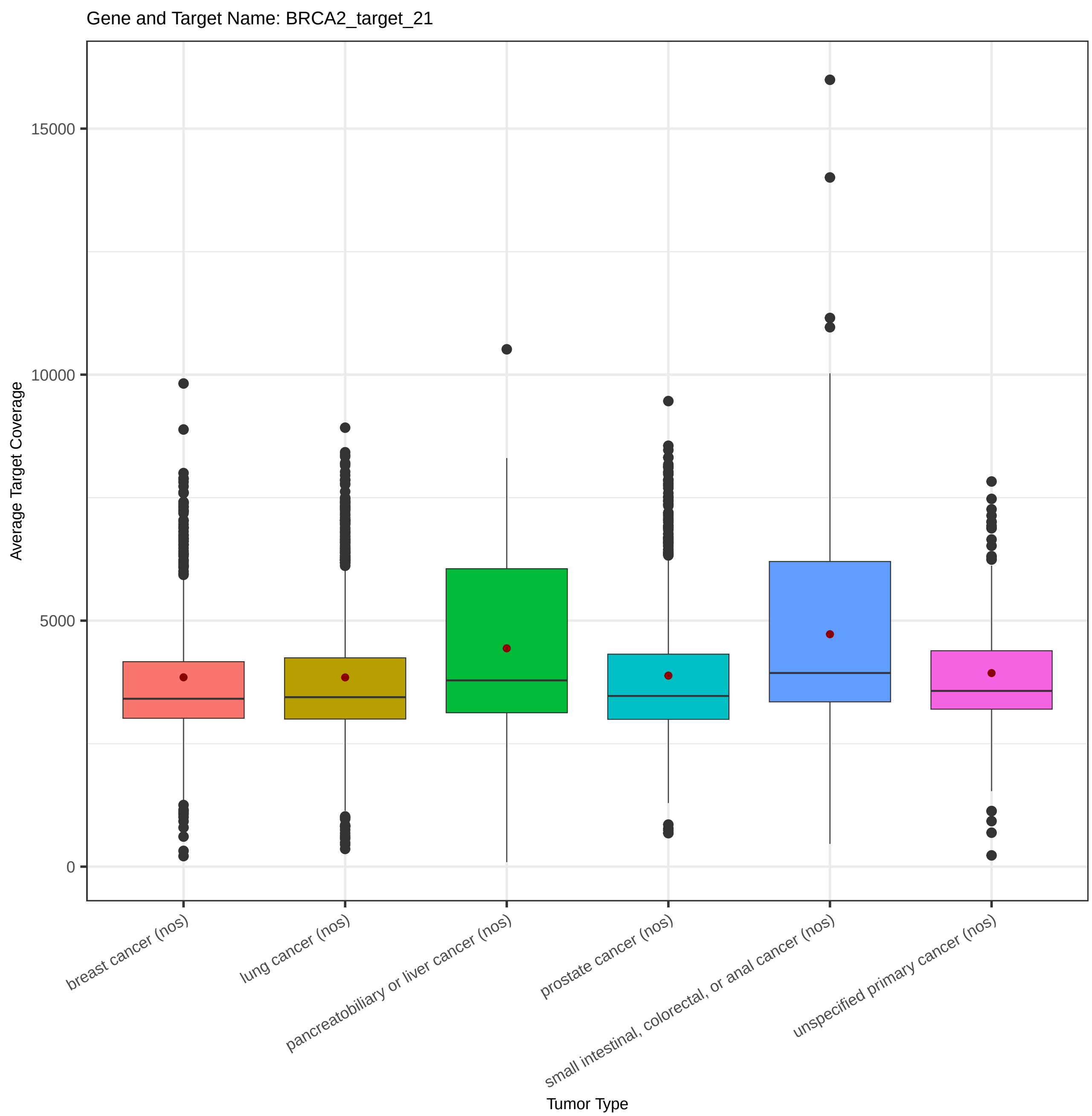
Gene and Target Name: BRCA2_target_21
Average Target Coverage
15000
10000
5000
0
breast cancer (nos)
lung cancer (nos)
pancreatobiliary or liver cancer (nos)
prostate cancer (nos)
small intestinal, colorectal, or anal cancer (nos)
unspecified primary cancer (nos)
Tumor Type

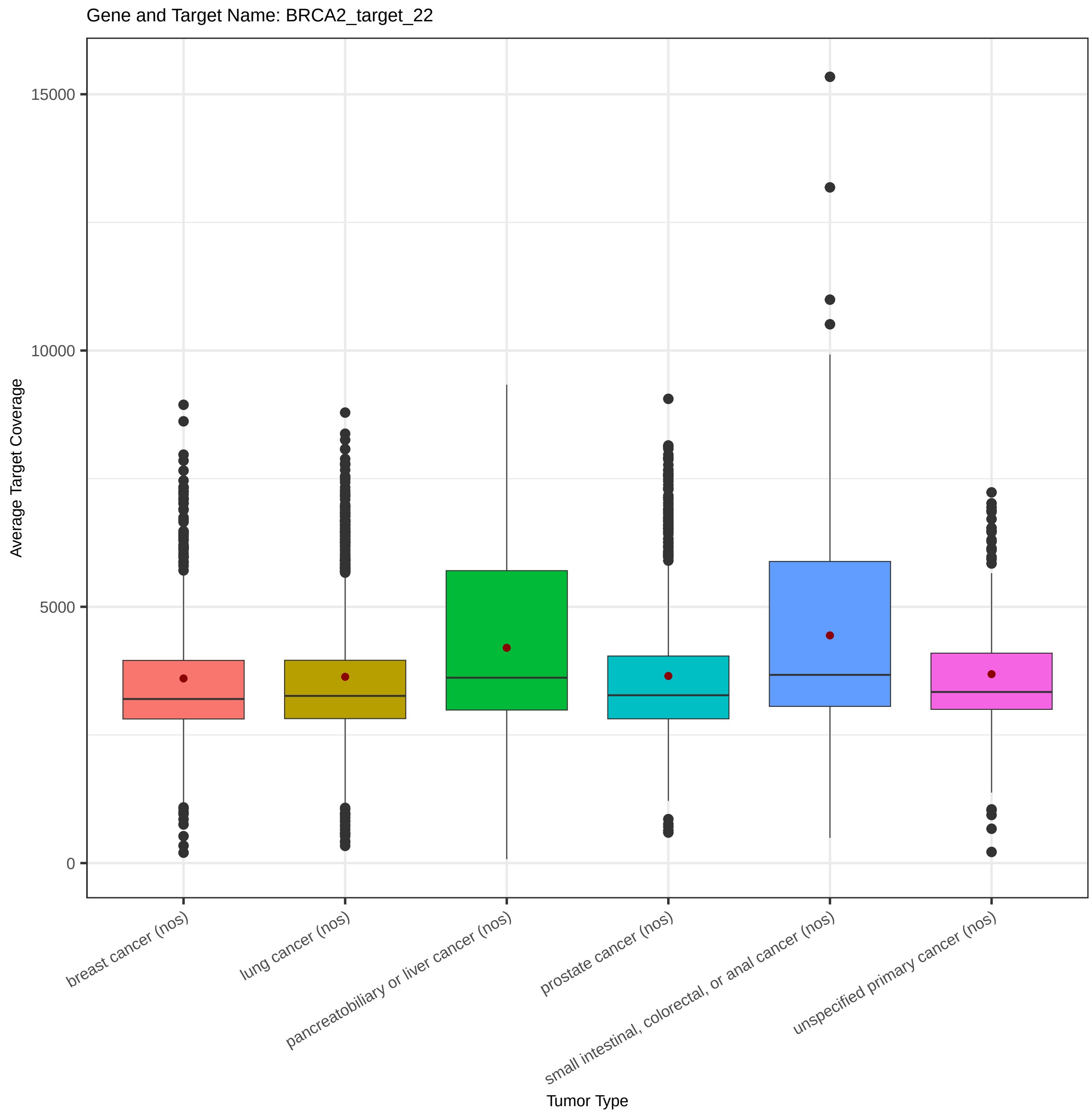
Gene and Target Name: BRCA2_target_22
Average Target Coverage
15000
10000
5000
0
breast cancer (nos)
lung cancer (nos)
pancreatobiliary or liver cancer (nos)
prostate cancer (nos)
small intestinal, colorectal, or anal cancer (nos)
unspecified primary cancer (nos)
Tumor Type

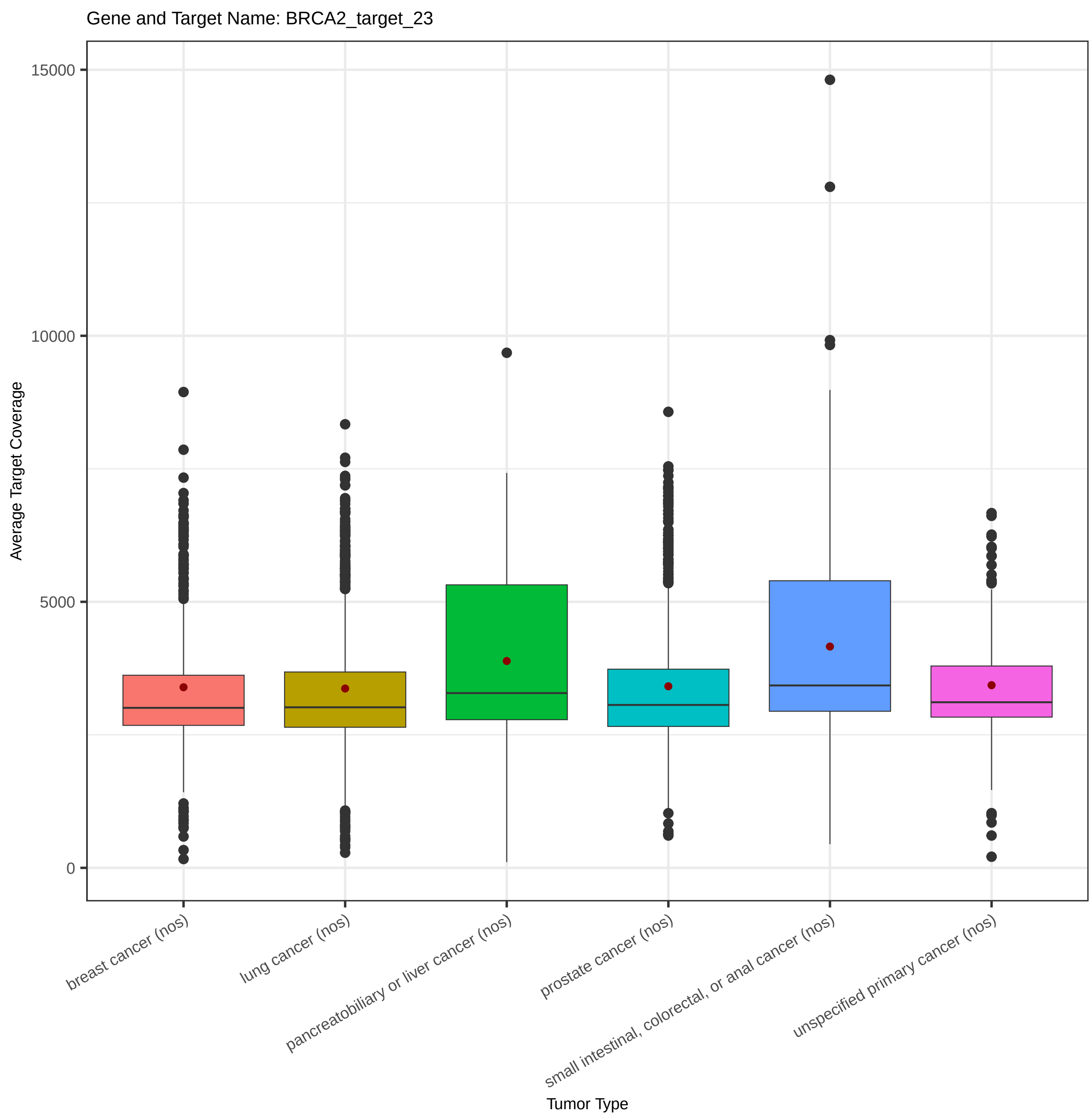

Gene and Target Name: BRCA2_target_23
Average Target Coverage
15000
10000
5000
0
breast cancer (nos)
lung cancer (nos)
pancreatobiliary or liver cancer (nos)
prostate cancer (nos)
small intestinal, colorectal, or anal cancer (nos)
unspecified primary cancer (nos)
Tumor Type

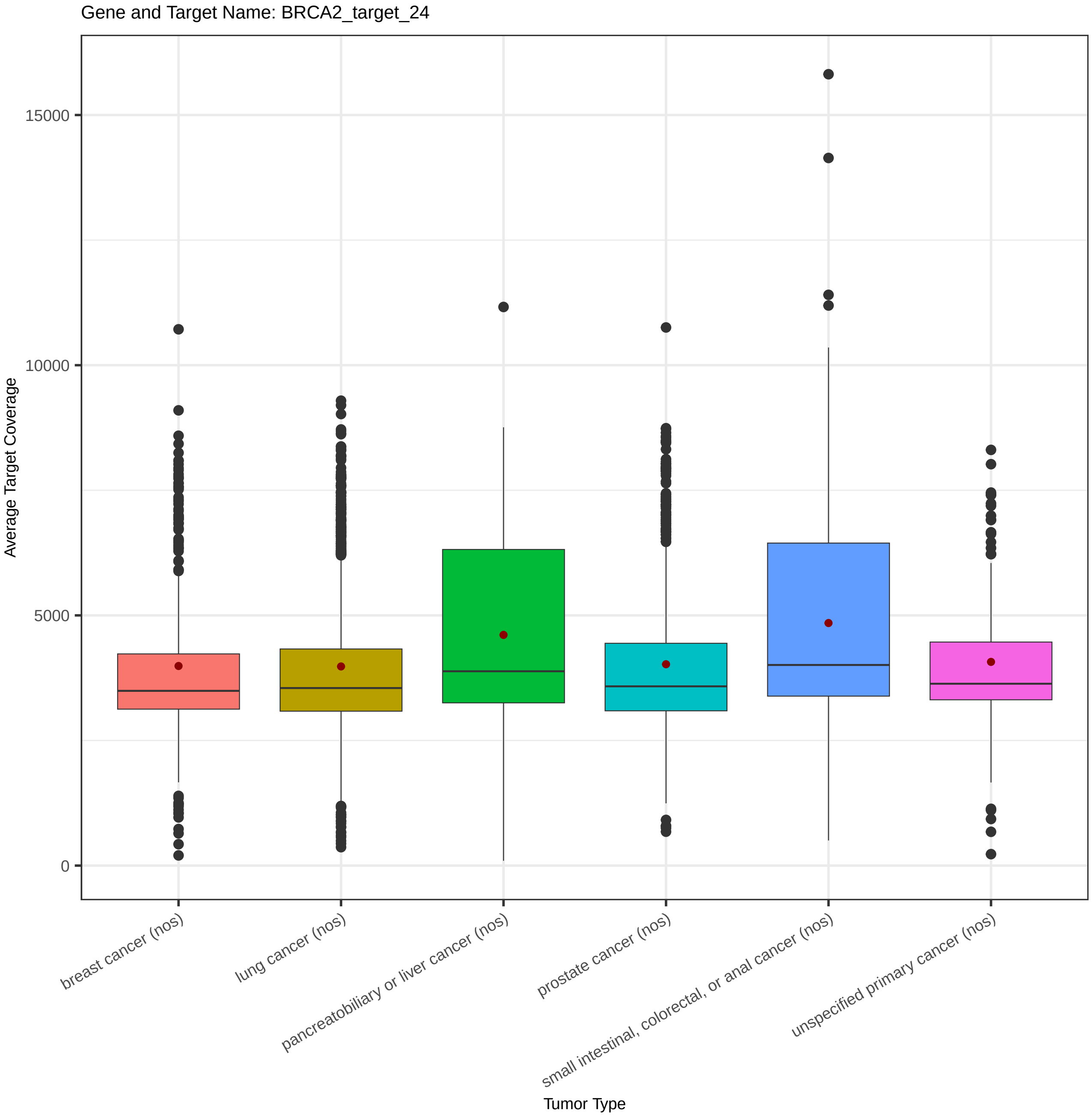

Gene and Target Name: BRCA2_target_24
Average Target Coverage
15000
10000
5000
0
breast cancer (nos)
lung cancer (nos)
pancreatobiliary or liver cancer (nos)
prostate cancer (nos)
small intestinal, colorectal, or anal cancer (nos)
unspecified primary cancer (nos)
Tumor Type

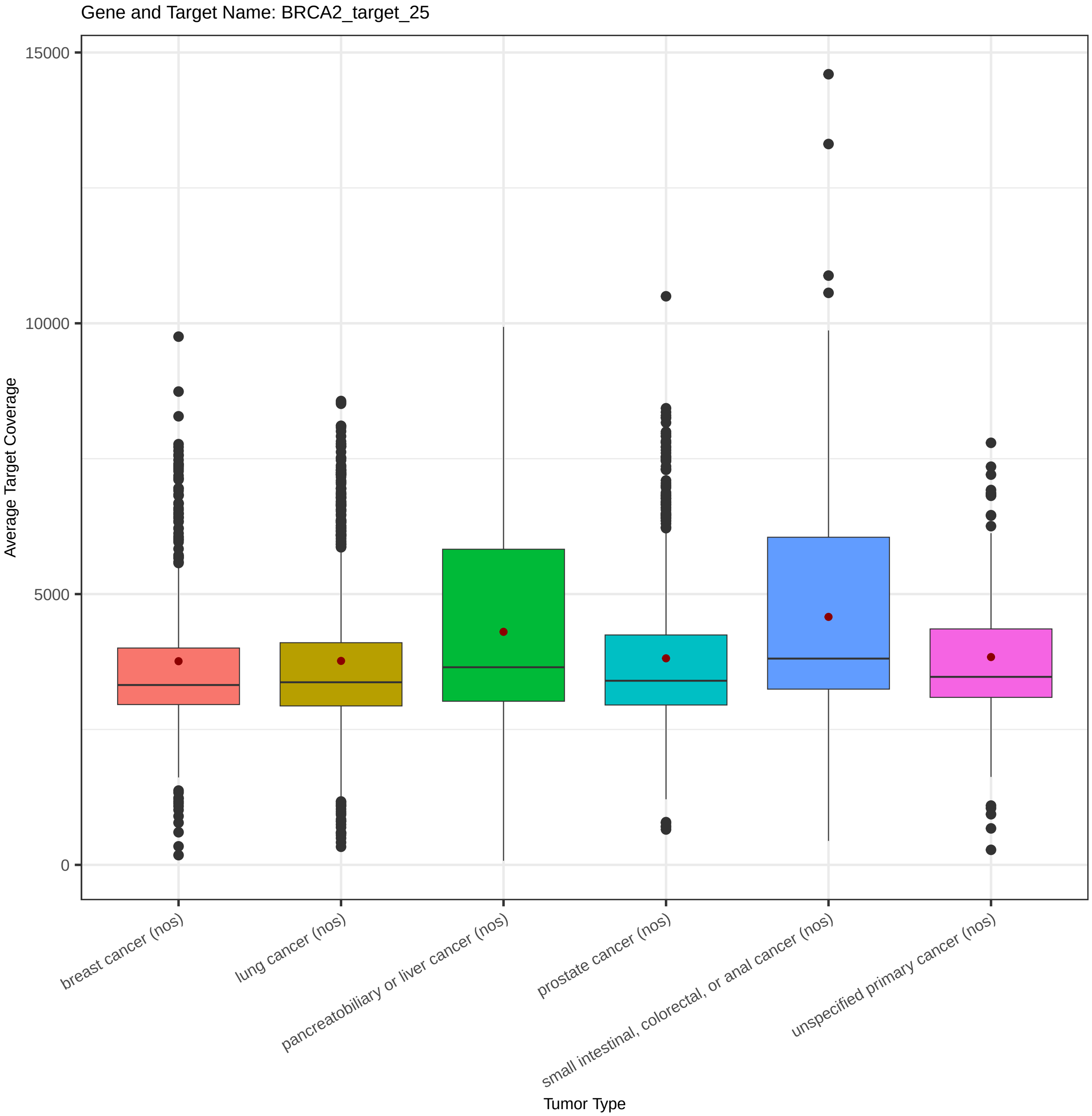

Gene and Target Name: BRCA2_target_25
Average Target Coverage
15000
10000
5000
0
breast cancer (nos)
lung cancer (nos)
pancreatobiliary or liver cancer (nos)
prostate cancer (nos)
small intestinal, colorectal, or anal cancer (nos)
unspecified primary cancer (nos)
Tumor Type

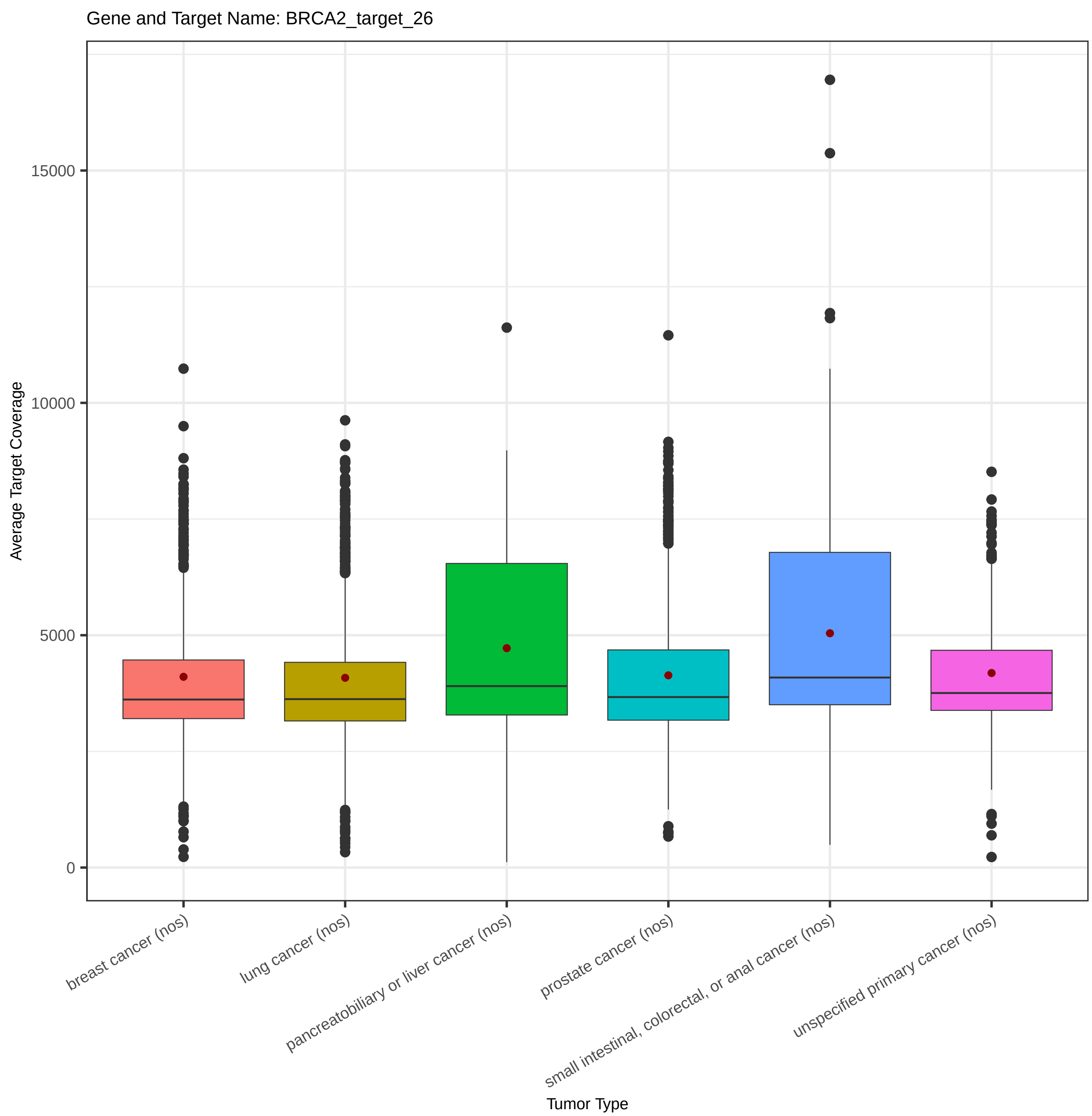

Gene and Target Name: BRCA2_target_26
Average Target Coverage
15000
10000
5000
0
breast cancer (nos)
lung cancer (nos)
pancreatobiliary or liver cancer (nos)
prostate cancer (nos)
small intestinal, colorectal, or anal cancer (nos)
unspecified primary cancer (nos)
Tumor Type

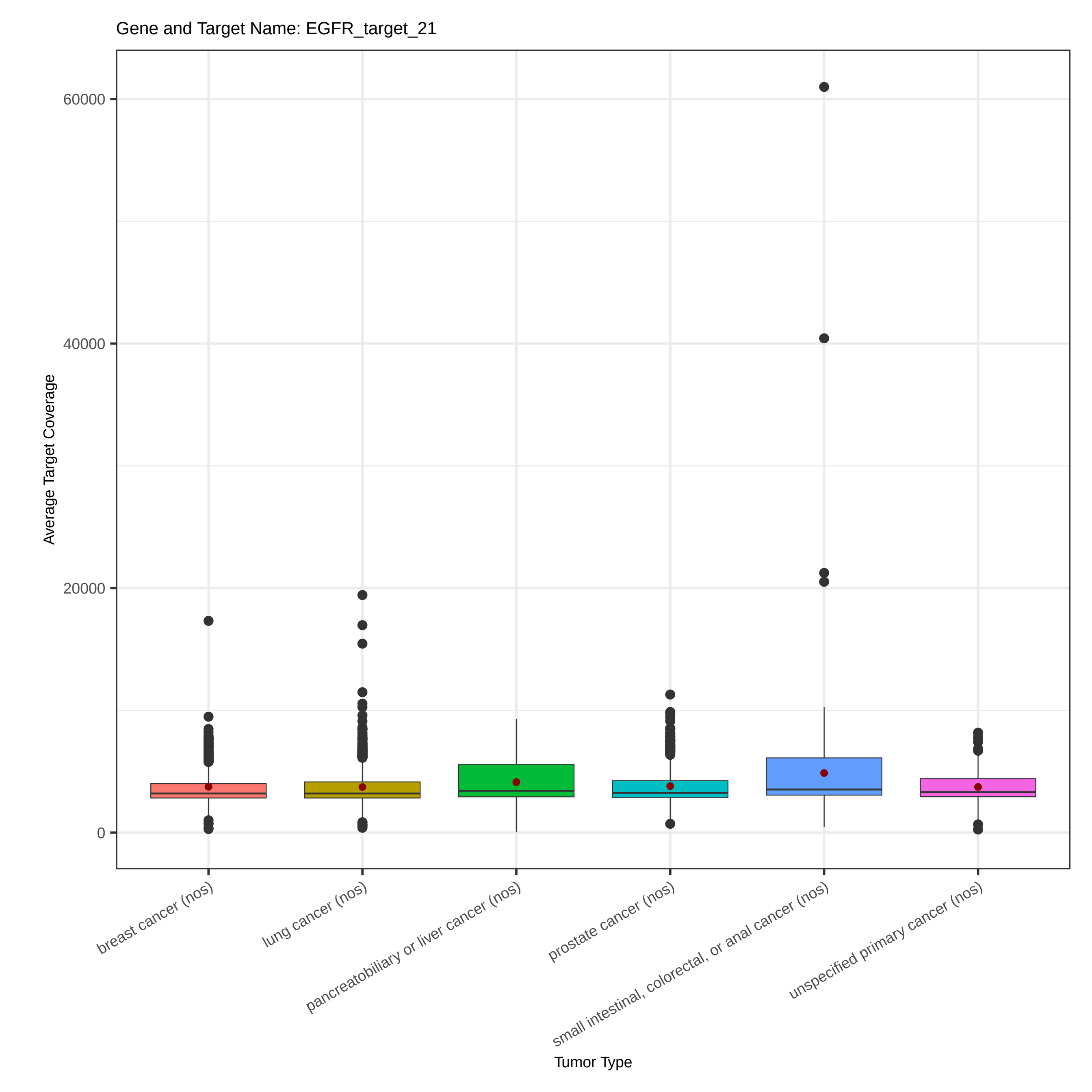

Gene and Target Name: EGFR_target_21
Average Target Coverage
60000
40000
20000
0
breast cancer (nos)
lung cancer (nos)
pancreatobiliary or liver cancer (nos)
prostate cancer (nos)
small intestinal, colorectal, or anal cancer (nos)
unspecified primary cancer (nos)
Tumor Type

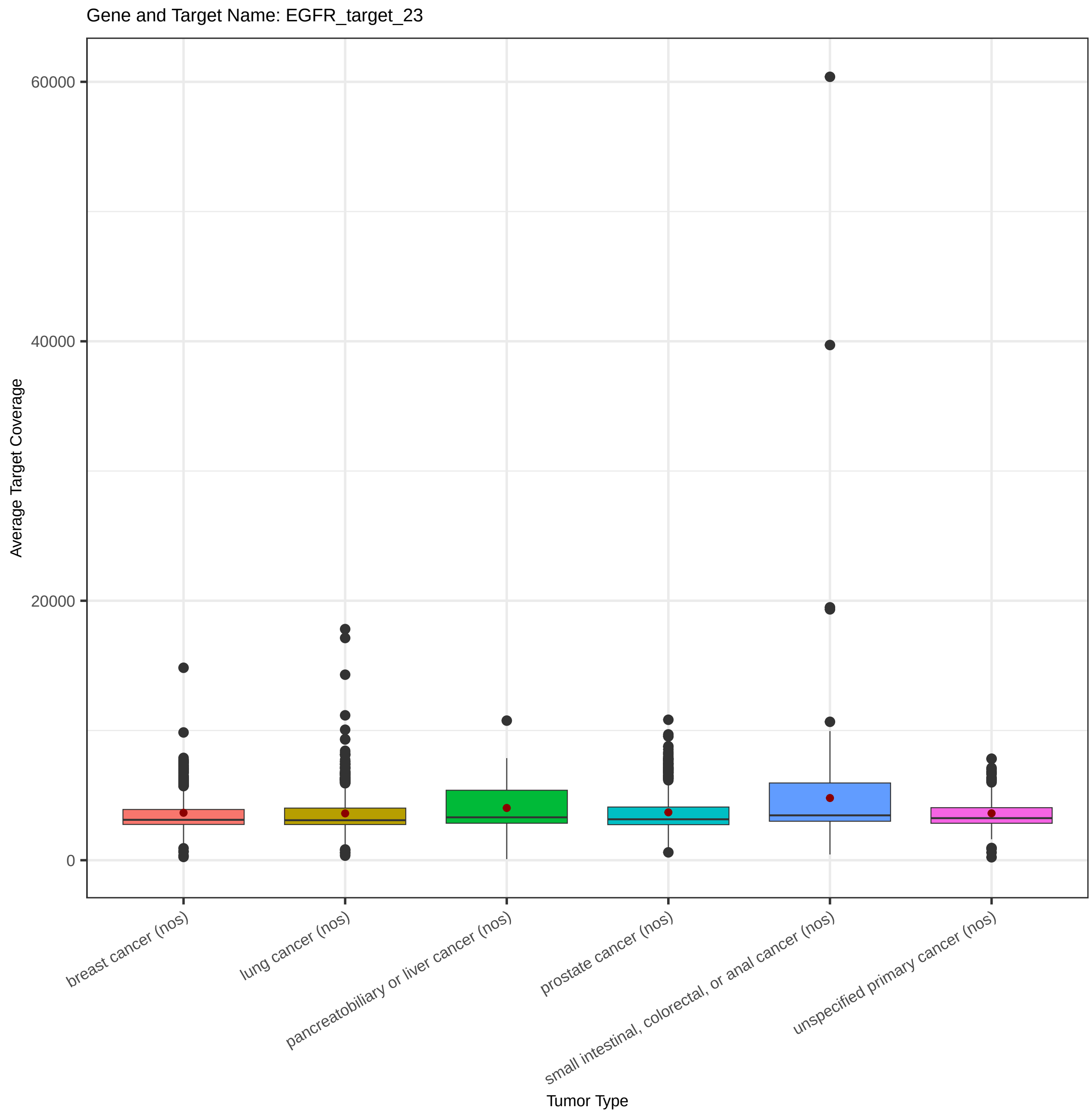

Gene and Target Name: EGFR_target_23
Average Target Coverage
60000
40000
20000
0
breast cancer (nos)
lung cancer (nos)
pancreatobiliary or liver cancer (nos)
prostate cancer (nos)
small intestinal, colorectal, or anal cancer (nos)
unspecified primary cancer (nos)
Tumor Type

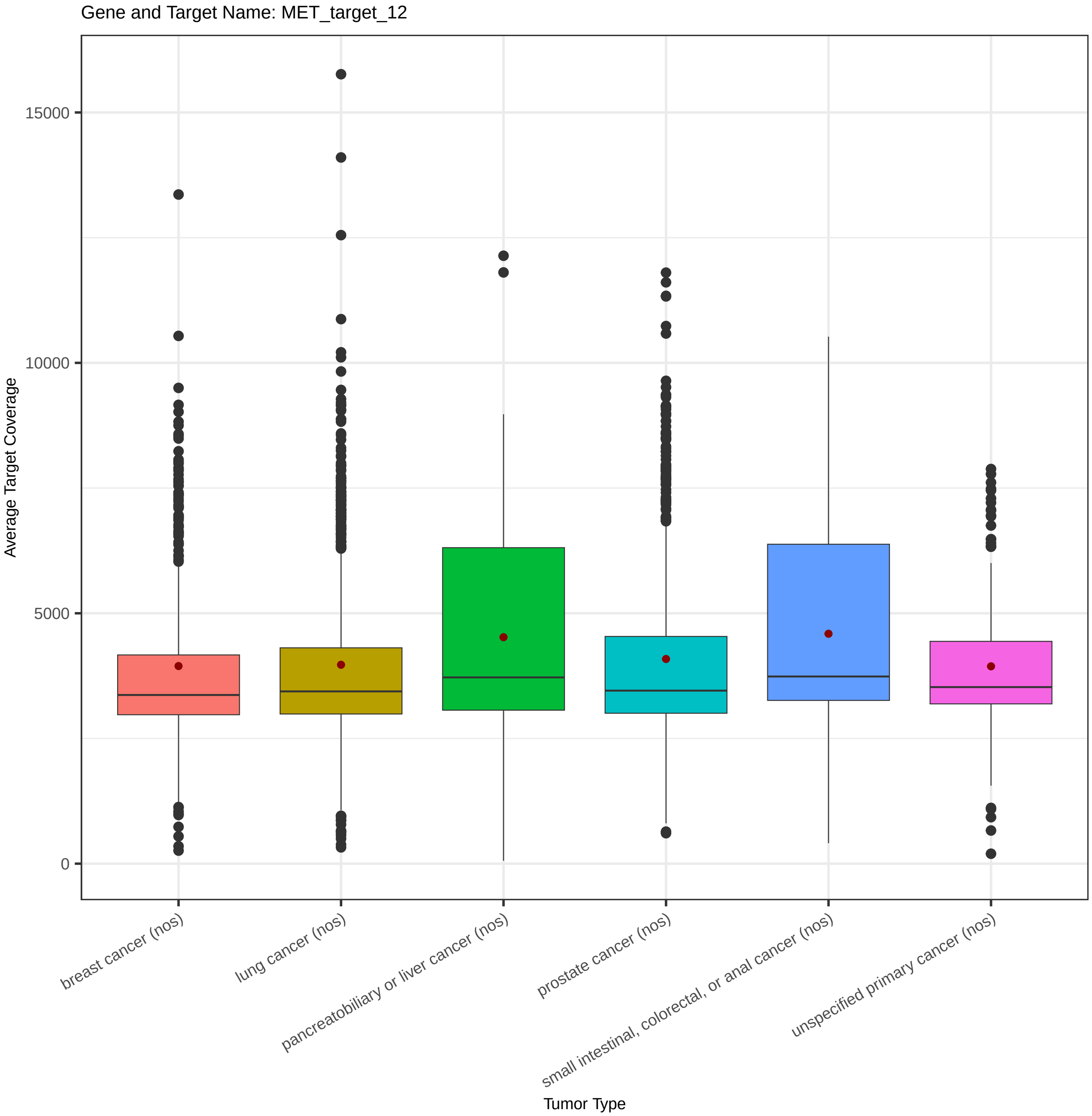

Gene and Target Name: MET_target_12
15000
10000
5000
0
Average Target Coverage
breast cancer (nos)
lung cancer (nos)
pancreatobiliary or liver cancer (nos)
prostate cancer (nos)
small intestinal, colorectal, or anal cancer (nos)
unspecified primary cancer (nos)
Tumor Type

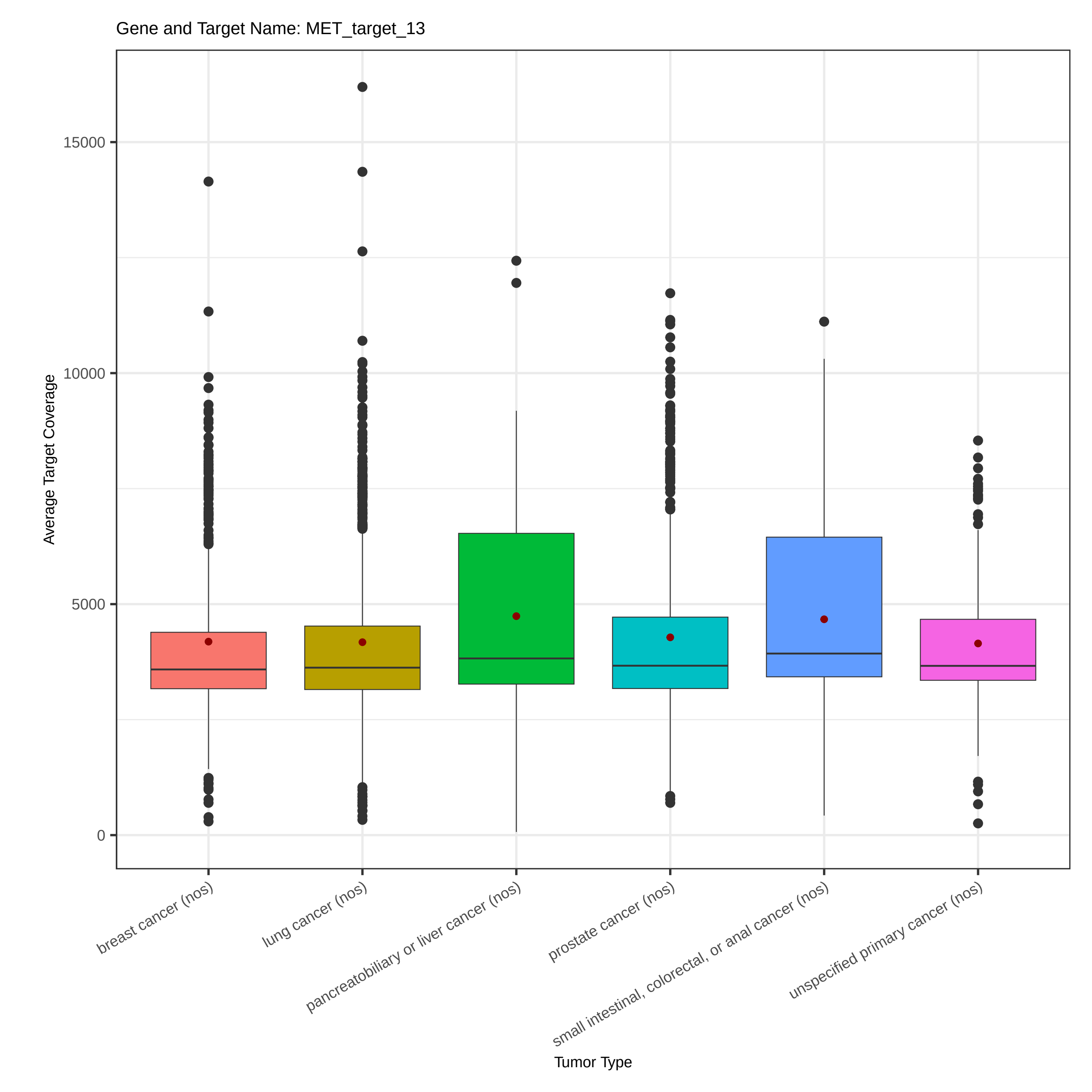
Gene and Target Name: MET_target_13
Average Target Coverage
15000
10000
5000
0
breast cancer (nos)
lung cancer (nos)
pancreatobiliary or liver cancer (nos)
prostate cancer (nos)
small intestinal, colorectal, or anal cancer (nos)
unspecified primary cancer (nos)
Tumor Type

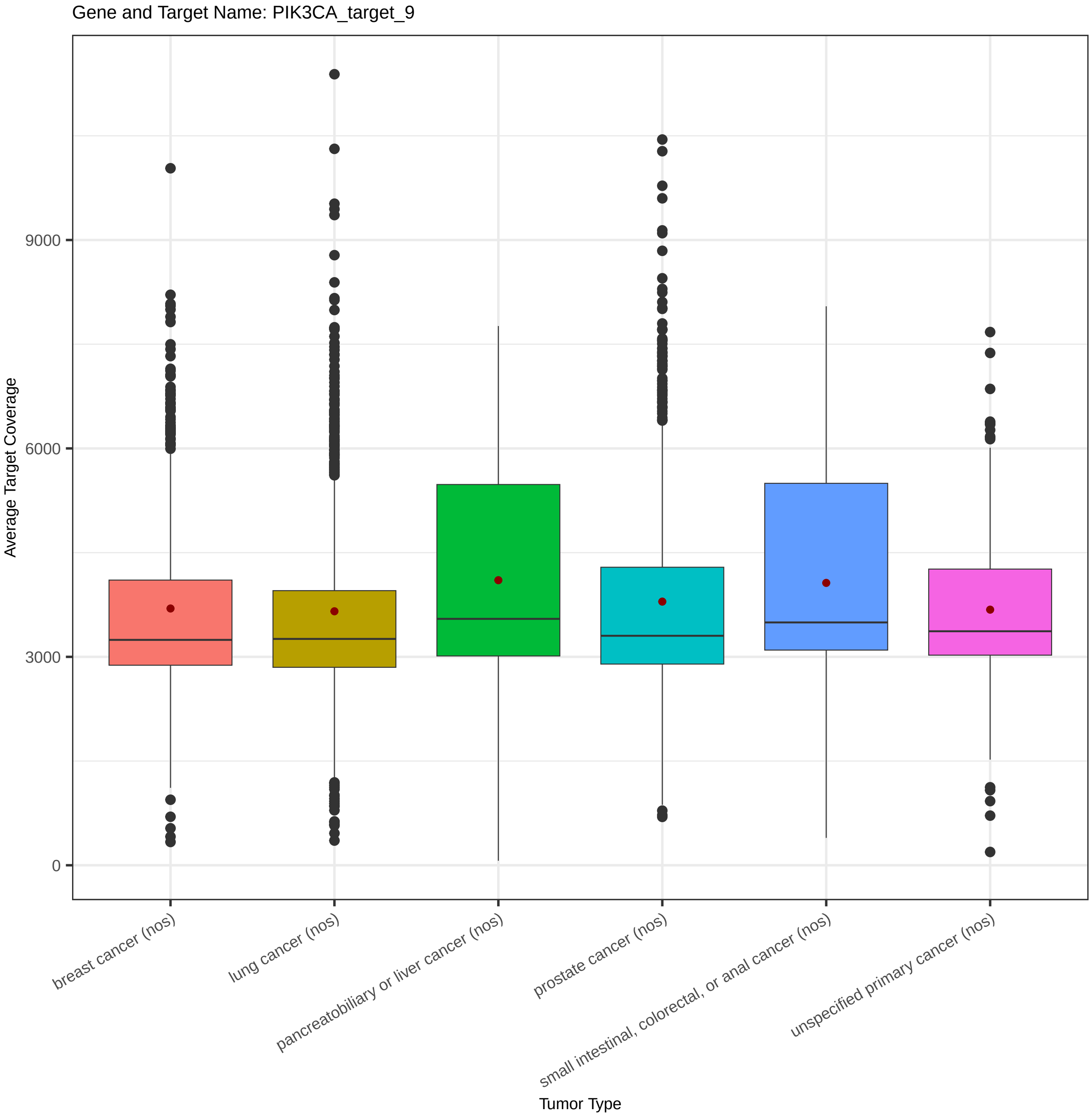

Gene and Target Name: PIK3CA_target_9
Average Target Coverage
9000
6000
3000
0
breast cancer (nos)
lung cancer (nos)
pancreatobiliary or liver cancer (nos)
prostate cancer (nos)
small intestinal, colorectal, or anal cancer (nos)
unspecified primary cancer (nos)
Tumor Type

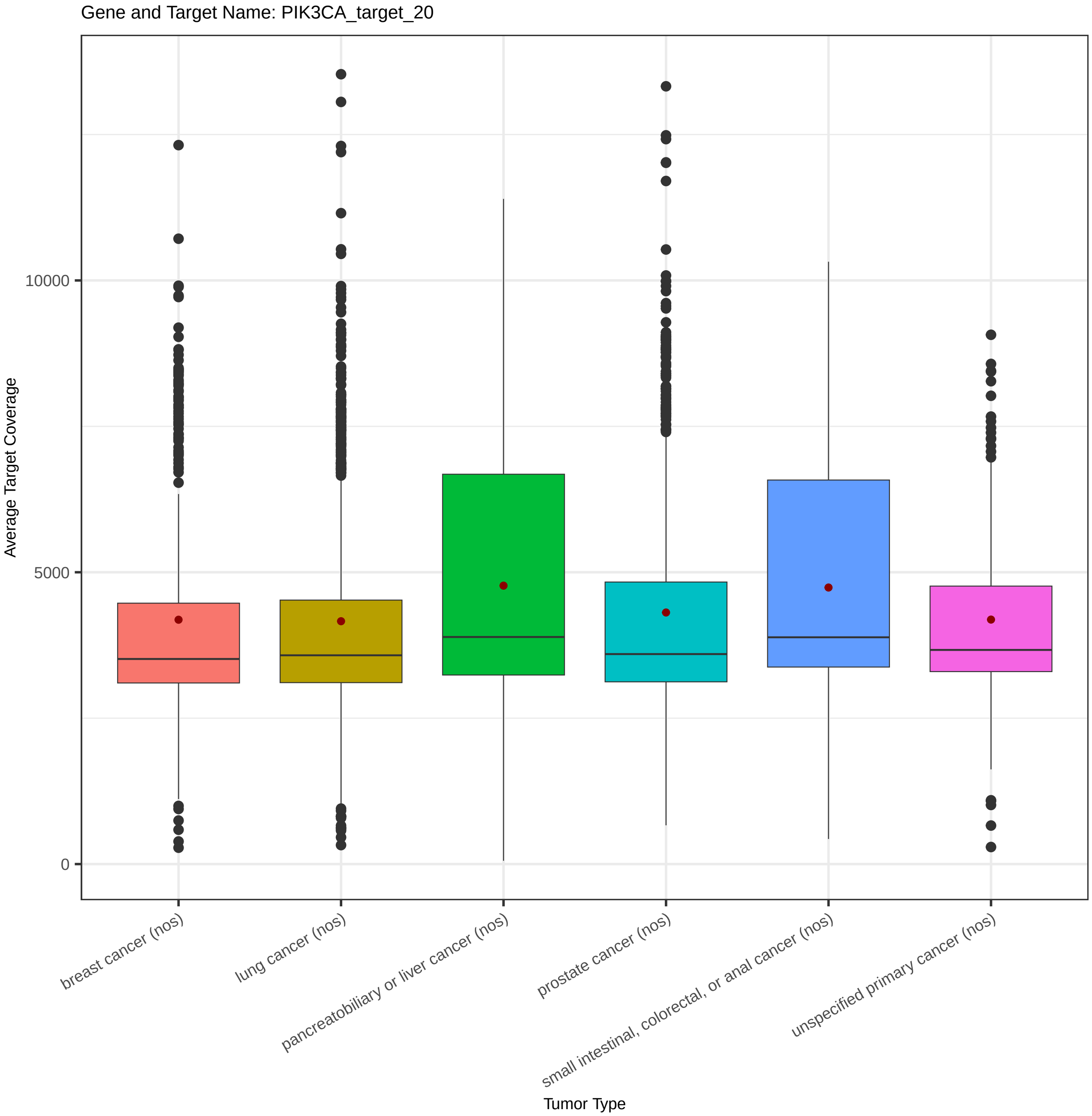
Gene and Target Name: PIK3CA_target_20
Average Target Coverage
10000
5000
0
breast cancer (nos)
lung cancer (nos)
pancreatobiliary or liver cancer (nos)
prostate cancer (nos)
small intestinal, colorectal, or anal cancer (nos)
unspecified primary cancer (nos)
Tumor Type

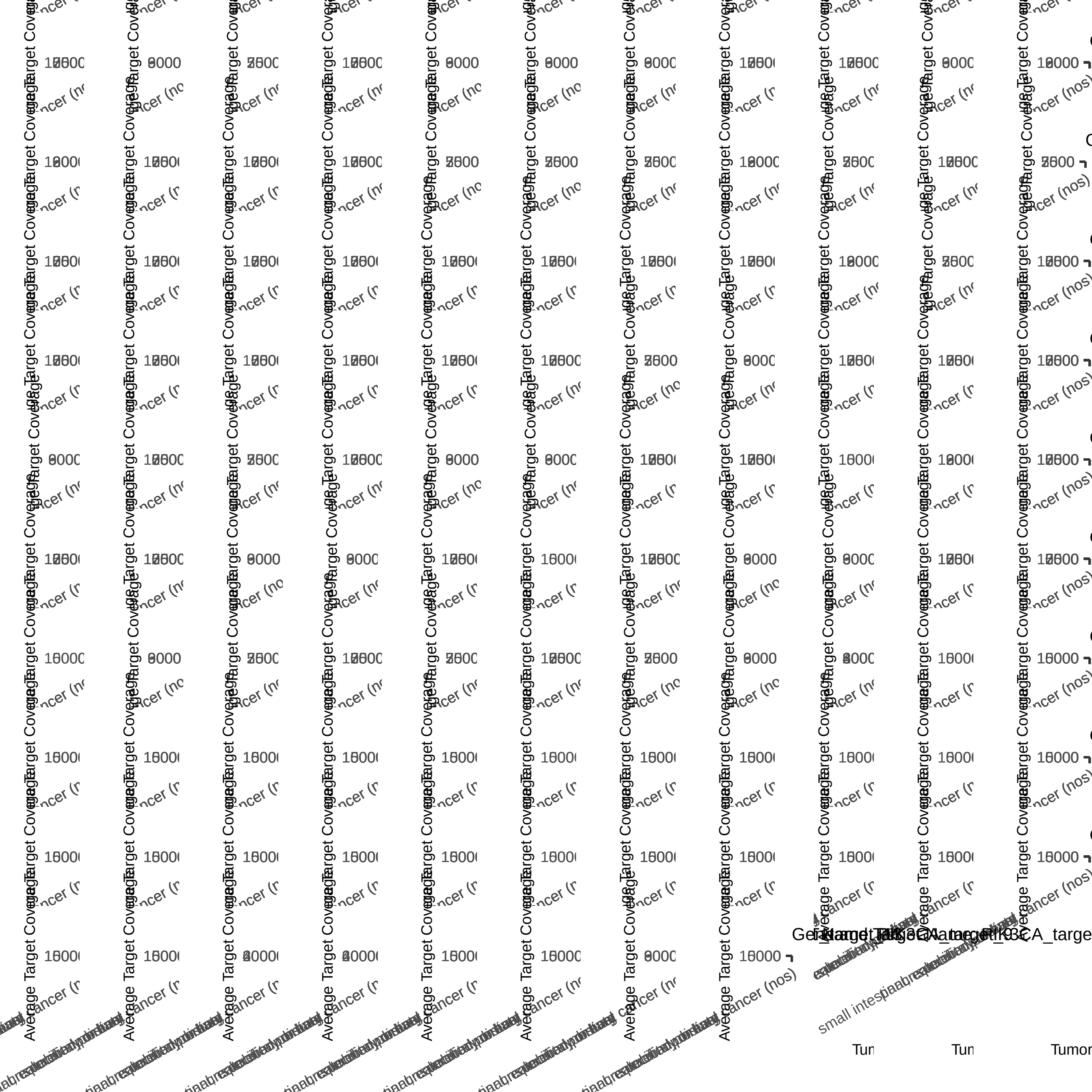

Average Target Coverage
Tumor
