# Supplementary figures and images for: Analytical validation and sequencing coverage studies suggest that performance of a liquid biopsy assay is tumor agnostic (DNA-is-DNA)
Source: PLoS One. 2025 Aug 1;20(8):e0329392. doi: 10.1371/journal.pone.0329392 (PMC12316276; doi:10.1371/journal.pone.0329392)

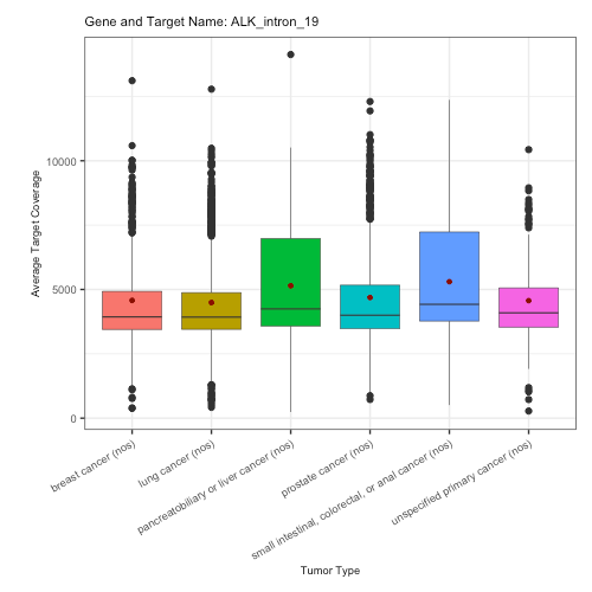

Supplement: S2 File — (ZIP) [file pone.0329392.s002.zip › Supplement Figures/S1 Fig.tif]

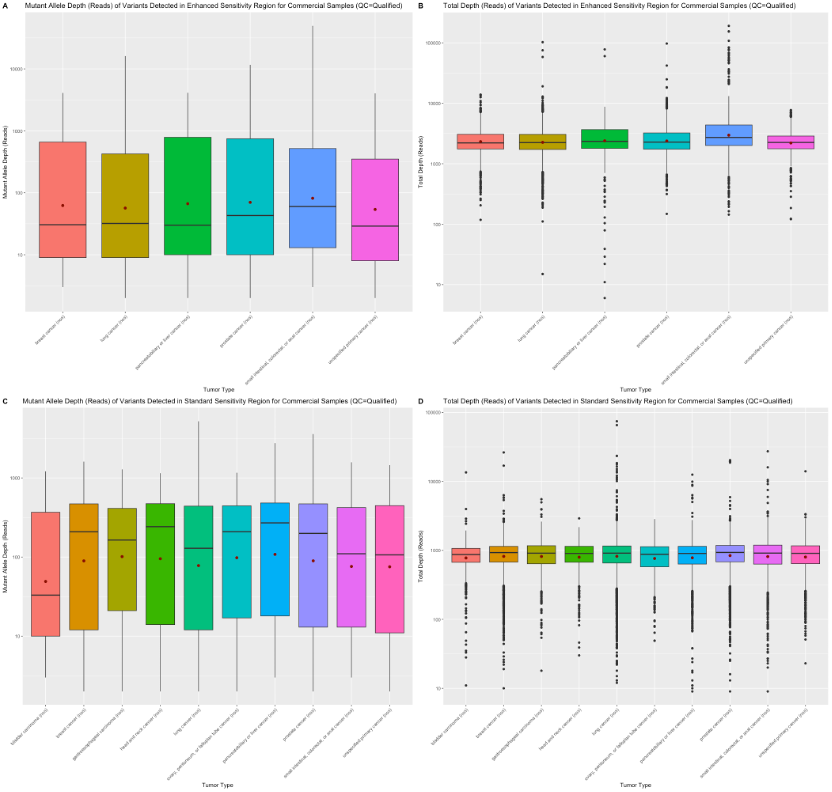

Supplement: S2 File — (ZIP) [file pone.0329392.s002.zip › Supplement Figures/S2 Fig.tif]

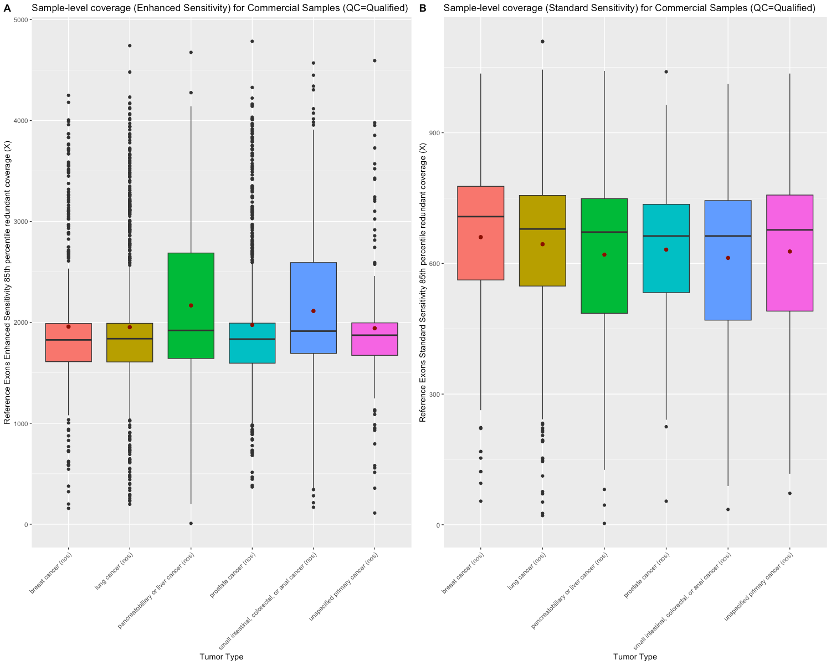

Supplement: S2 File — (ZIP) [file pone.0329392.s002.zip › Supplement Figures/S3 Fig.tif]
